# Supplementary material for: ASMT determines gut microbiota and increases neurobehavioral adaptability to exercise in female mice
Source: Commun Biol. 2023 Nov 7;6:1126. doi: 10.1038/s42003-023-05520-8 (PMC10630421; doi:10.1038/s42003-023-05520-8)
Supplement: Supplementary file 2 — Supplementary Figures and Tables [file 42003_2023_5520_MOESM2_ESM.pdf]

ASMT determines gut microbiota and increases neurobehavioral adaptability to exercise in female mice

Weina Liu[1,2,#*], Zhuochun Huang[1,2], Ye Zhang[1,2], Sen Zhang[1,2], Zhiming Cui[1,2], Wenbin Liu[1,2], Lingxia Li[1,2], Jie Xia[1,2], Yong Zou[1,2], Zhengtang Qi[1,2, #*]

[1] The Key Laboratory of Adolescent Health Assessment and Exercise Intervention (Ministry of Education), East China Normal University, Shanghai 200241, China

[2] College of Physical Education and Health, East China Normal University, Shanghai 200241, China

[#] These authors contributed equally.

*Correspondence: ztqi@tyxx.ecnu.edu.cn (Z.Q.), Tel: +86-21-54342612;

wnliu@tyxx.ecnu.edu.cn (W.L.), Tel: +86-21-54341197.

Keywords: N-acetylserotonin O-methyltransferase; frameshift mutation; depression; anxiety; exercise; gut microbiota

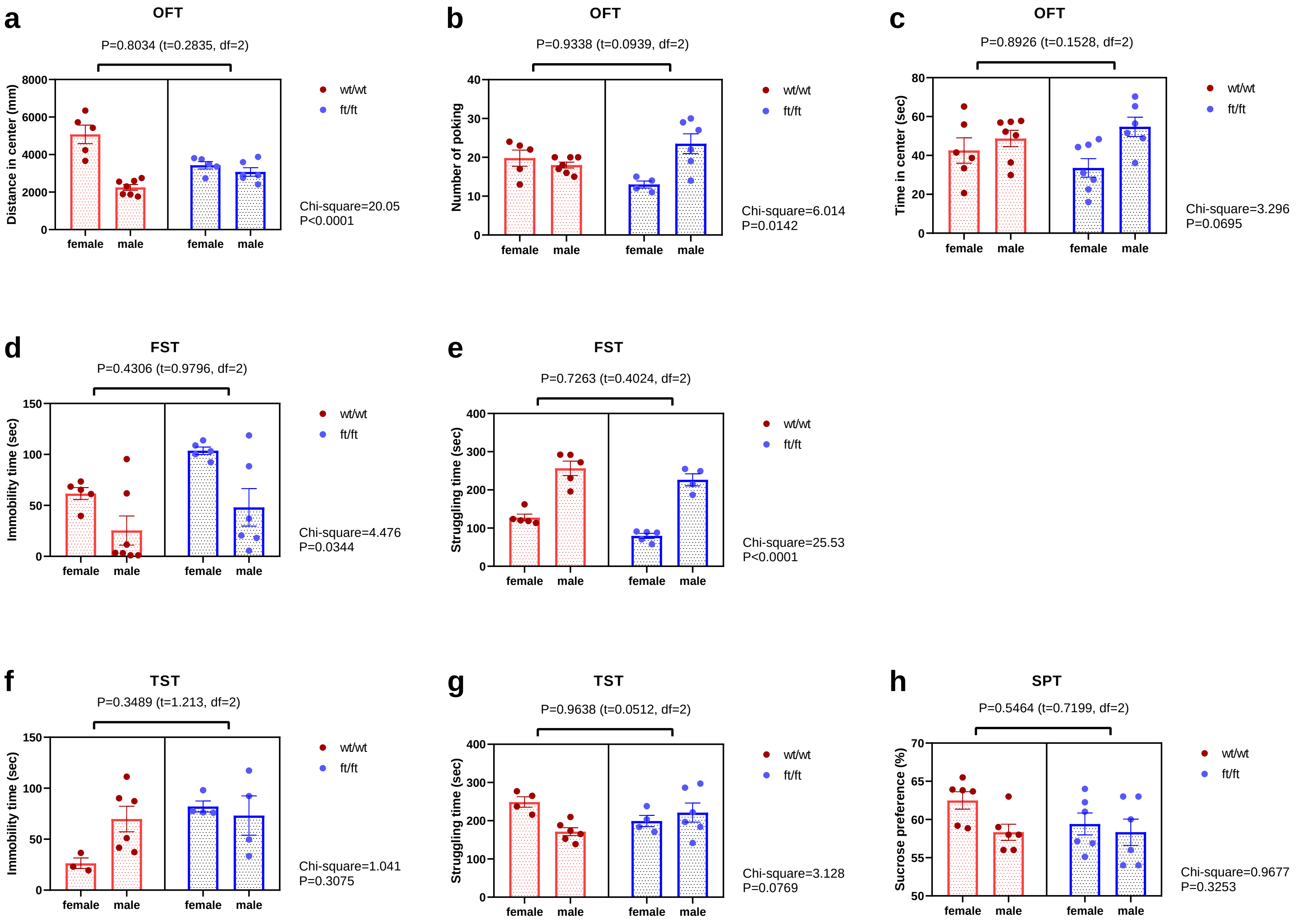

Fig. S1 Nested t-test and Chi-square test for anxiety and depression-like behaviors in mice. a-c Distance in center, number of poking, and time in center in the OFT (n=4~7/group). d, e Immobility time and struggling time in the FST (n=4~7/group). f, g Immobility time and struggling time in the TST (n=4~7/group). h Sucrose preference in the SPT (n=6/group). Data are mean ± S.E.M. The P-value above each panel represents the result of Nested t-test, and the Chi-square value at the lower right represents the result of Chi-square test.

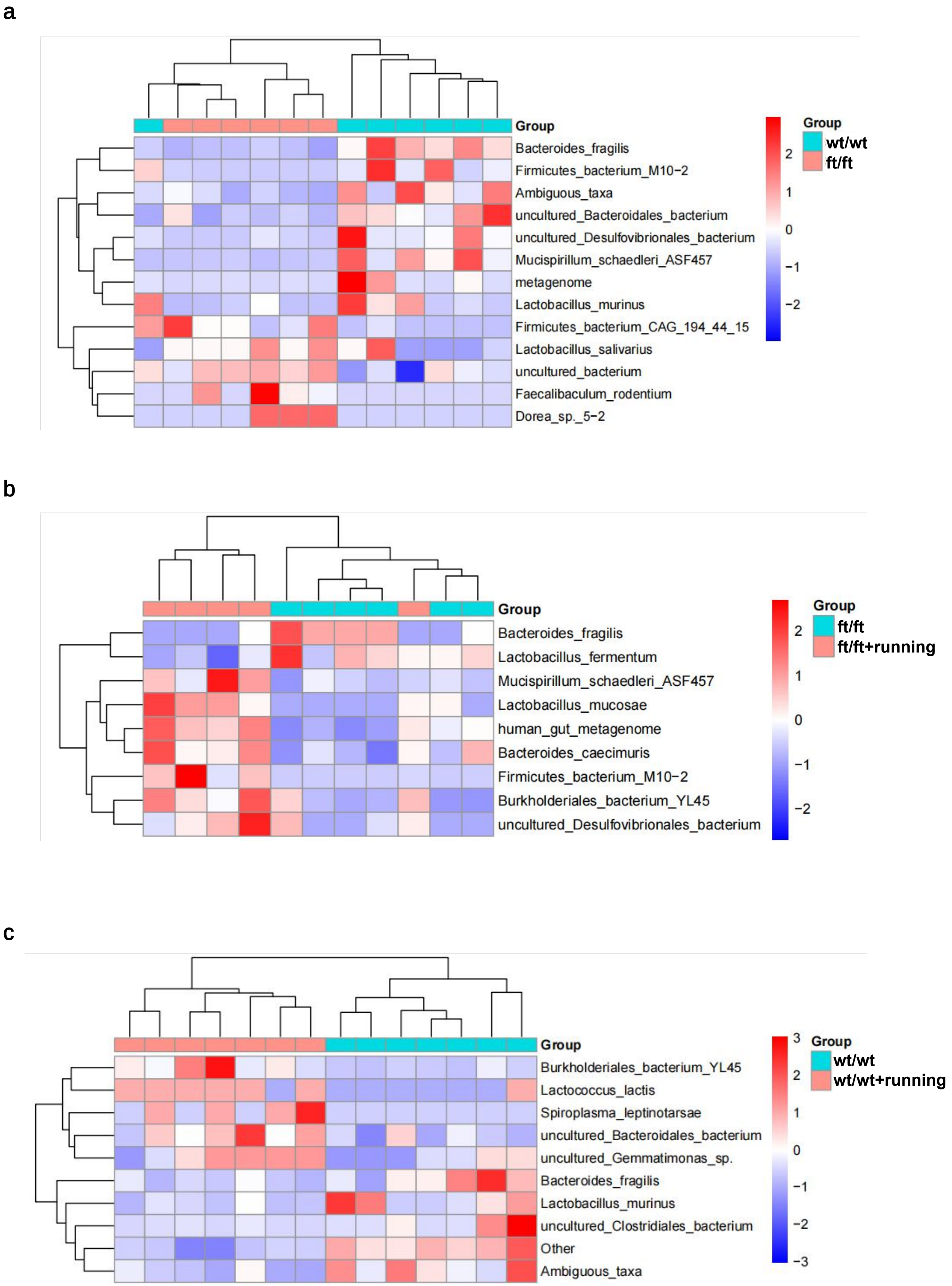

Figure S2. Hierarchical cluster analysis for the structure of gut microbiota after exercise. a, ASMT$^{ft/ft}$ resulted in 13 different species compared with the wild-type. **b,** Exercise resulted in 9 different species in ASMT$^{ft/ft}$ mice. **c,** Exercise resulted in 10 different species in wt/wt mice and a perfect clustering in group (Wilcoxon test).

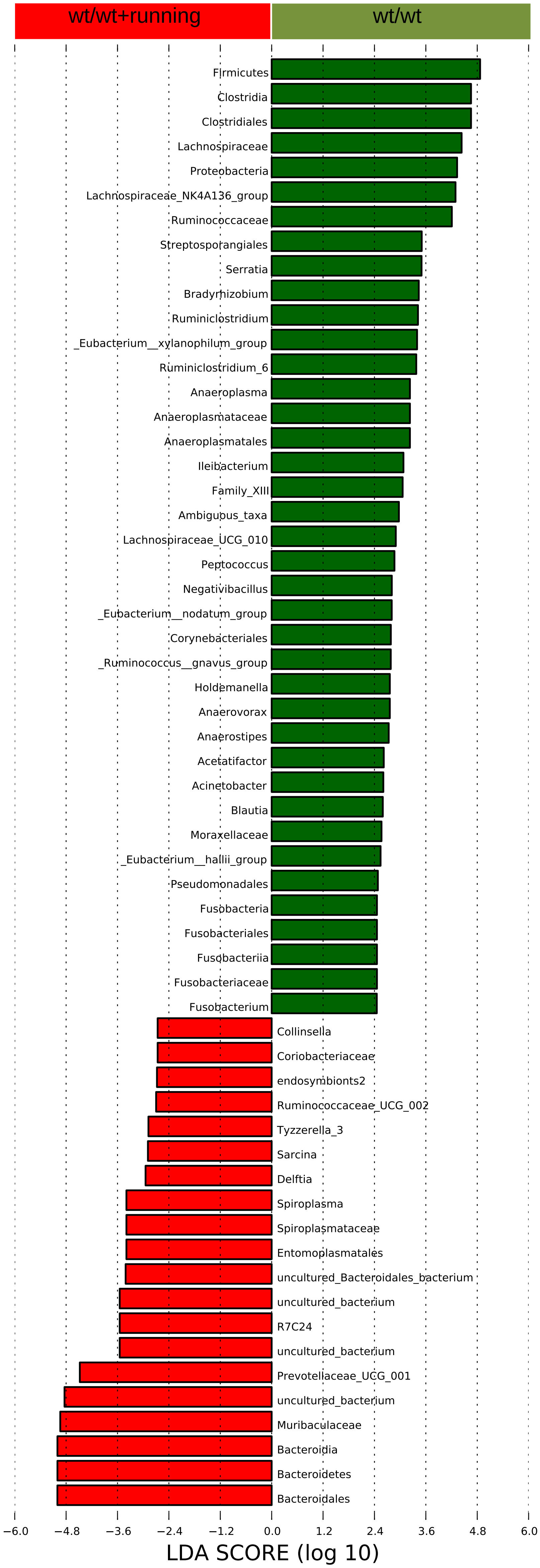

Figure S3. Key species of gut microbiota responding to running identified using LEfSe in wild-type mice. The histogram shows the LDA scores computed for features (on the species level) differentially abundant between sedentary and running group after exercise.

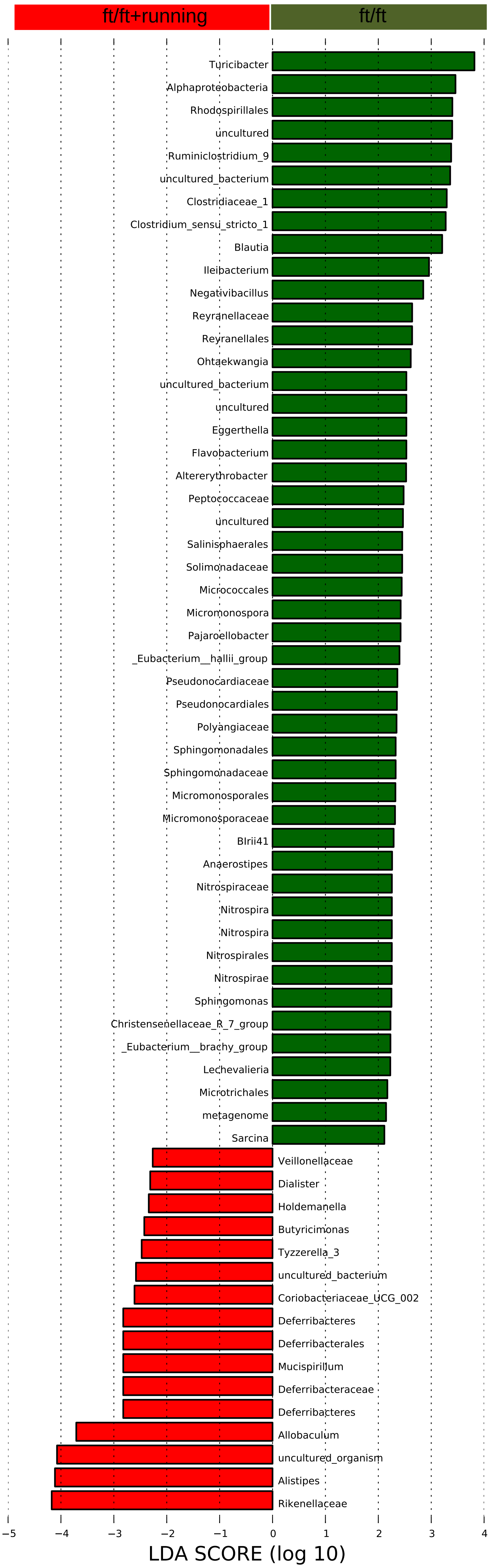

Figure S4. Key species of gut microbiota responding to running identified using LEfSe in ASMT$^{ft/ft}$ mice. The histogram shows the LDA scores computed for features (on the species level) differentially abundant between sedentary and running group after exercise.

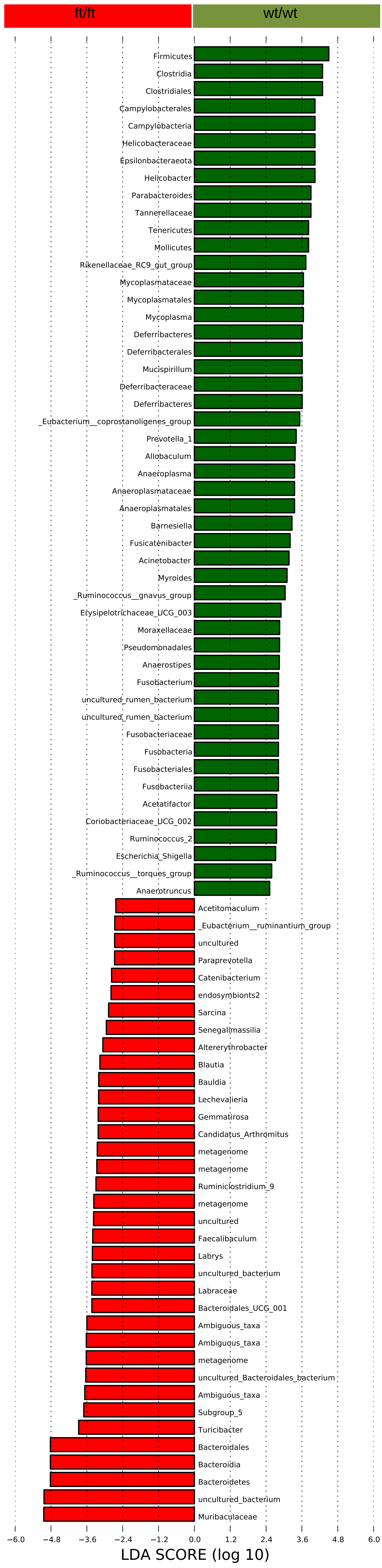

Figure S5. Key species of gut microbiota between genotypes identified using LEfSe. The histogram shows the LDA scores computed for features (on the species level) differentially abundant between wild-type and ASMT$^{ft/ft}$ mice in the third collection of feces.

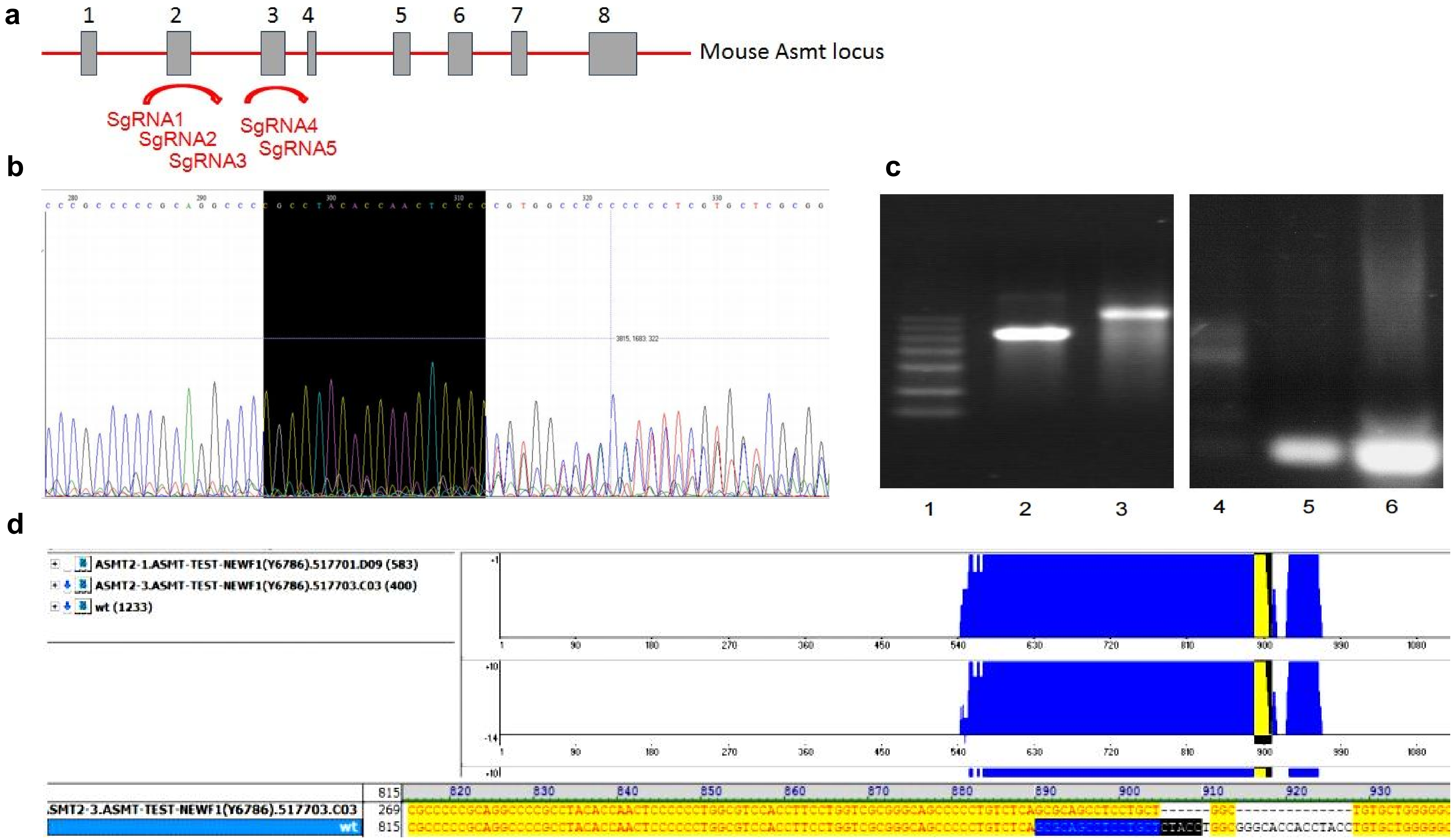

Figure S6. Asmt gene structure and scheme of SgRNA-guided frameshift mutation. a, The Asmt gene contains 8 exons. In order to produce effective frameshift mutations, we designed five SgRNAs targeting exons 2 and 3 in the upstream of the gene (see **Supplementary Table S2**). **b,** Asmt-SgRNAs plasmid was transient transfected into NIH3T3 cells, and the sequencing results showed that SgRNA1 and SgRNA2 had higher efficiency. **c,** Synthesis of Cas9 mRNA and Asmt-SgRNA1/2 transcripts in vitro. Lane 1, 6kD RNA marker; Lane 2, Cas9 mRNA (before adding a poly(A) tail); Lane 3, Cas9 mRNA (after adding a poly(A) tail); Lane 4, 1kD RNA marker; Lane 5, Asmt-SgRNA1; Lane 6, Asmt2-SgRNA2. Electrophoresis showed that Asmt-SgRNA1 and Asmt-SgRNA2 had high transfection efficiency in vitro. **d,** Gene sequencing of the 2# founder mouse showed that Asmt had a 20bp loss of exon 2, resulting in a highly efficient frameshift mutation.

Supplementary Table S1

Intron-exon organization of the murine Asmt, chrX:170,672,644-170,678,054 (base numbering in base pairs from mm10, Sept 2019 assembly of the mouse genome at https://genome.ucsc.edu/cgi-bin/hgGene?hgg_gene=ENSMUST00000178693.1&hgg_prot=uc029xop.1&hgg_chrom=chrX&hgg_start=170672643&hgg_end=170678054&hgg_type=knownGene&db=mm10&hgsid=1134780985_SMbHyvni75QmDiKhDdA8YPv5DSTz).

Feature	Start	End	Size	
Exon 1	170,672,644	170,672,753	110	
Intron 1	170,672,754	170,673,624	871	
Exon2	170,673,625	170,673,796	172	sgRNA target
Intron 2	170,673,797	170,674,621	825	
Exon3	170,674,622	170,674,754	133	sgRNA target
Intron 3	170,674,755	170,674,960	206	
Exon4	170,674,961	170,675,029	69	
Intron 4	170,675,030	170,675,747	718	
Exon5	170,675,748	170,675,866	119	
Intron 5	170,675,867	170,676,337	471	
Exon6	170,676,338	170,676,493	156	
Intron 6	170,676,494	170,677,016	523	
Exon7	170,677,017	170,677,139	123	
Intron 7	170,677,140	170,677,745	606	
Exon8	170,677,746	170,678,054	309	

Supplementary Table S2

SgRNAs designed for gene targeting

SgRNA#	Sequence
Asmt -SgRNA1	5’ -GGGCCGCGGCGTCGAACACG-3’
Asmt -SgRNA2	5’ -GCGGCGCTGGCGAGGTCGTC -3’
Asmt -SgRNA3	5’ -GCCGCGTCCCCCGGGGGCTC -3’
Asmt -SgRNA4*	5’ -gCGCCTACACCAACTCCCCCC -3’
Asmt -SgRNA5	5’ -GCGCAGCCTCCTGCTCTACC-3’

* g was added at the 5 '-terminal for SgRNA4 to facilitate the activation of the hU6 promoter.
