# Supplementary material for: ASMT determines gut microbiota and increases neurobehavioral adaptability to exercise in female mice
Source: Commun Biol. 2023 Nov 7;6:1126. doi: 10.1038/s42003-023-05520-8 (PMC10630421; doi:10.1038/s42003-023-05520-8)
Supplement: Supplementary file 3 — Description of Additional Supplementary Files [file 42003_2023_5520_MOESM3_ESM.pdf]

Description of Additional Supplementary Files

File name: Supplementary Data 1
Description: The QIIME2 tables underlying the analyses.

File name: Supplementary Data 2
Description: Source data related to plots and graph in the manuscript.
