# Supplementary material for: ASMT determines gut microbiota and increases neurobehavioral adaptability to exercise in female mice
Source: Commun Biol. 2023 Nov 7;6:1126. doi: 10.1038/s42003-023-05520-8 (PMC10630421; doi:10.1038/s42003-023-05520-8)
Supplement: Supplementary file 4 — Supplementary Data 1 [file 42003_2023_5520_MOESM4_ESM.zip › 2.OTUs/OTU_level_plot/annotationbarplot_class.pdf]

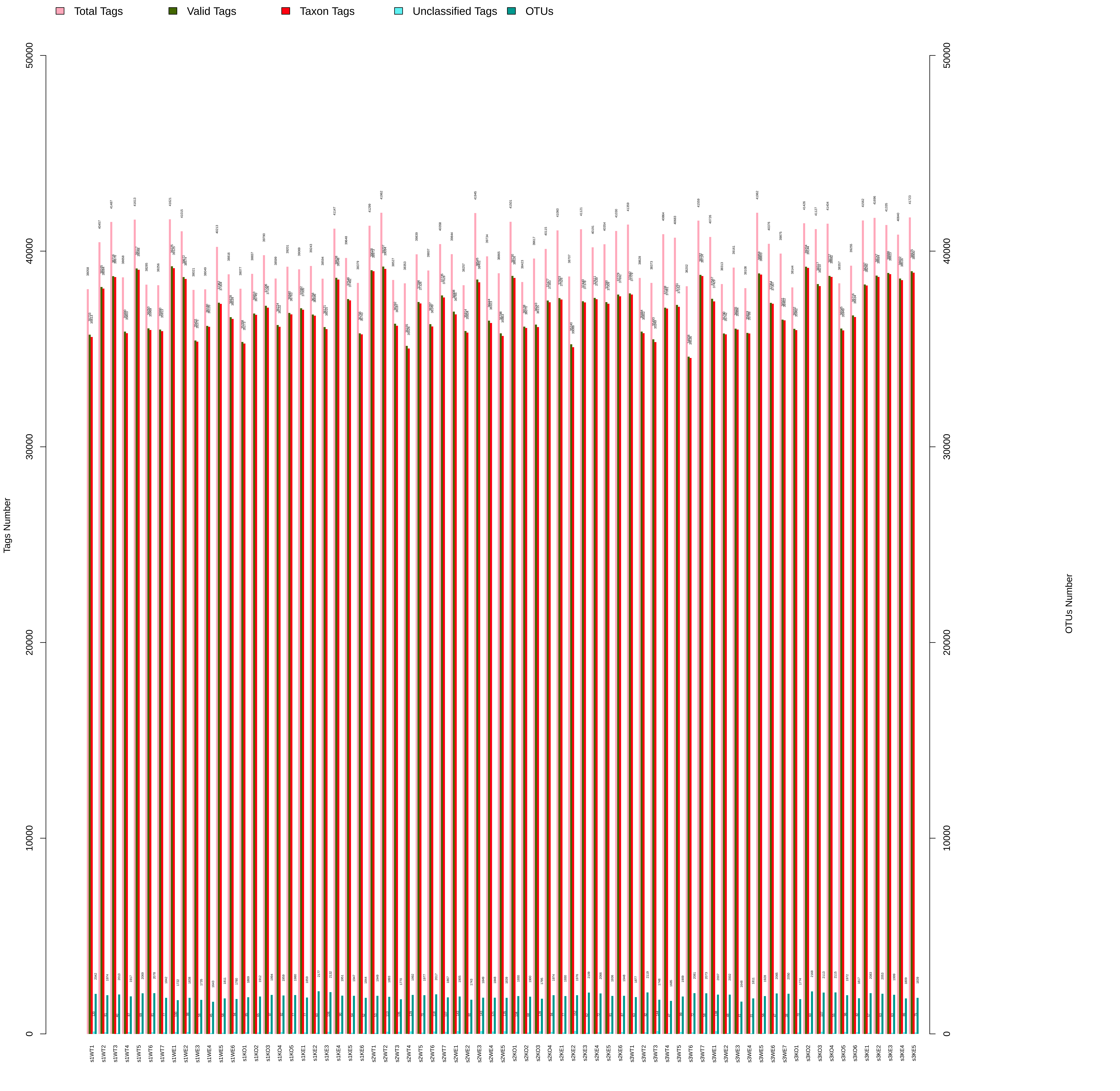

Total Tags
Valid Tags
Taxon Tags
Unclassified Tags
OTUs
Tags Number
OTUs Number
0
10000
20000
30000
40000
50000
