# Supplementary material for: ASMT determines gut microbiota and increases neurobehavioral adaptability to exercise in female mice
Source: Commun Biol. 2023 Nov 7;6:1126. doi: 10.1038/s42003-023-05520-8 (PMC10630421; doi:10.1038/s42003-023-05520-8)
Supplement: Supplementary file 4 — Supplementary Data 1 [file 42003_2023_5520_MOESM4_ESM.zip › 2.OTUs/OTU_level_plot/annotationbarplot_family.pdf]

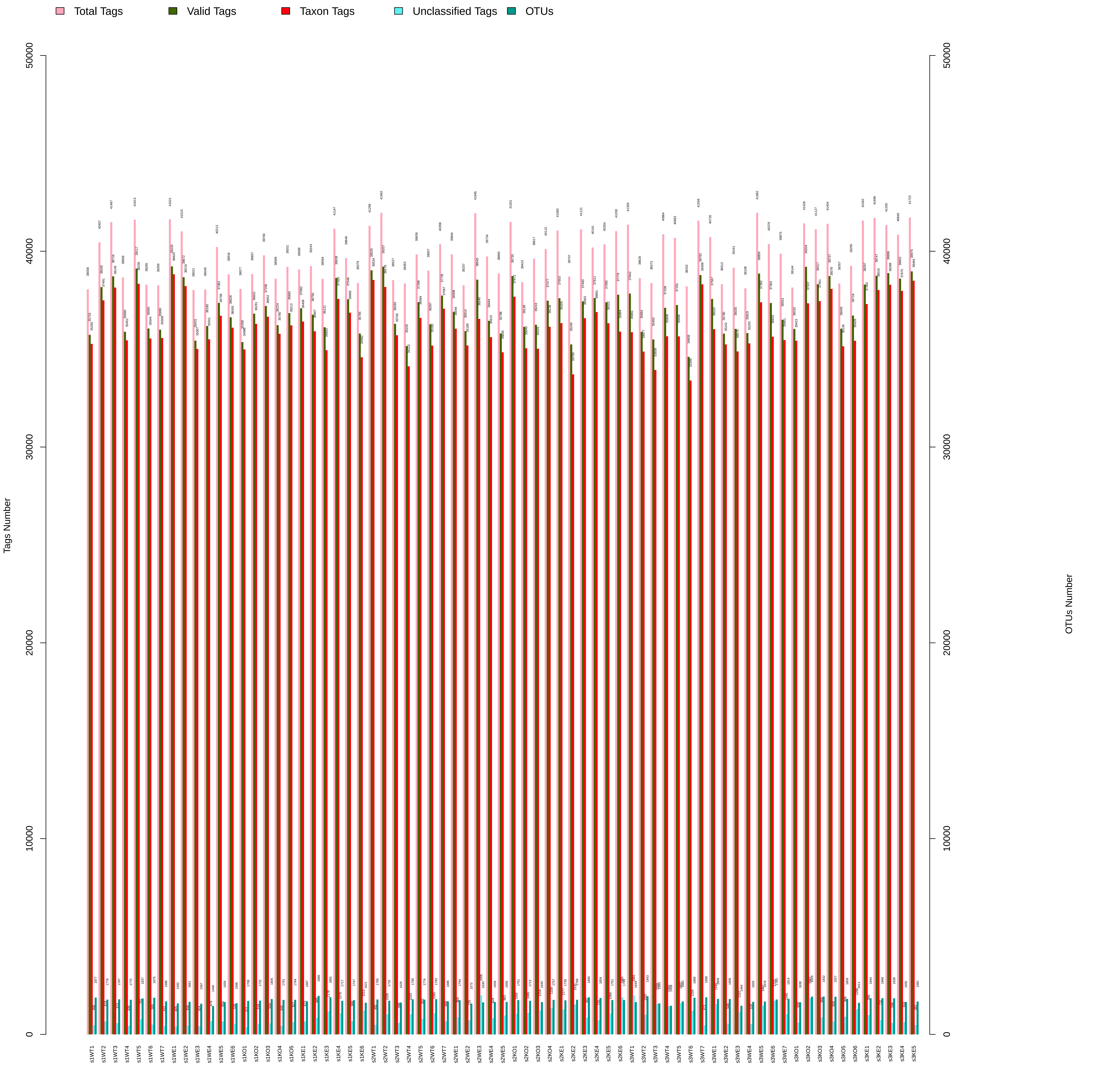
Total Tags
Valid Tags
Taxon Tags
Unclassified Tags
OTUs
Tags Number
OTUs Number
0
10000
20000
30000
40000
50000
s1WT1 s1WT2 s1WT3 s1WT4 s1WT5 s1WT6 s1WT7
s1WE1 s1WE2 s1WE3 s1WE4 s1WE5 s1WE6
s1KO1 s1KO2 s1KO3 s1KO4 s1KO5
s1KE1 s1KE2 s1KE3 s1KE4 s1KE5 s1KE6
s2WT1 s2WT2 s2WT3 s2WT4 s2WT5 s2WT6 s2WT7
s2WE1 s2WE2 s2WE3 s2WE4
s2KO1 s2KO2 s2KO3 s2KO4
s2KE1 s2KE2 s2KE3 s2KE4 s2KE5 s2KE6
s3WT1 s3WT2 s3WT3 s3WT4 s3WT5 s3WT6 s3WT7
s3WE1 s3WE2 s3WE3 s3WE4 s3WE5 s3WE6 s3WE7
s3KO1 s3KO2 s3KO3 s3KO4 s3KO5 s3KO6
s3KE1 s3KE2 s3KE3 s3KE4 s3KE5
