# Supplementary material for: ASMT determines gut microbiota and increases neurobehavioral adaptability to exercise in female mice
Source: Commun Biol. 2023 Nov 7;6:1126. doi: 10.1038/s42003-023-05520-8 (PMC10630421; doi:10.1038/s42003-023-05520-8)
Supplement: Supplementary file 4 — Supplementary Data 1 [file 42003_2023_5520_MOESM4_ESM.zip › 4.Alpha_Diversity/alpha_rarefaction_plot/rarefaction_plots_pdf_depth27686/rarefaction_plots.html]

 
 
 
   
   Rarefaction Curves 
 
td.data{font-size:10px;border-spacing:0px 10px;text-align:center;}
td.headers{font-size:12px;font-weight:bold;text-align:center;}
table{border-spacing:0px;}
.removed{display:none;}
.expands{cursor:pointer; cursor:hand;}
.child1 td:first-child{padding-left: 3px;}
 
 

function show_hide_category(checkobject){
    var imagetype=document.getElementById('imagetype').value;
    img=document.getElementById(checkobject.name.replace('_raw'+imagetype,'_ave'+imagetype))
    if (checkobject.checked==false){
        img.style.display='none';
    }else{
        img.style.display='';
    }
}

function reset_tree(){
    var category=document.getElementById('category').value;
    var metric=document.getElementById('metric').value;
    var old_all_categories=document.getElementById('all_categories');
    var imagetype=document.getElementById('imagetype').value;
    cat_list=old_all_categories.value.split('$#!')
    if (metric!='' && category != ''){
    for (var i=1, il=cat_list.length; i 

 
 
 
 
 
 
 
 
  
  Select a Metric:  
 
 
   
 PD_whole_tree 
 chao1 
 goods_coverage 
 observed_species 
 shannon 
 simpson 
 
 
    Select a Category:  
 
 
   
 BarcodeSequence 
 Description 
 Group 
 LinkerPrimerSequence 
 SampleID 
 
 
 
 
 
  

 
      Show Categories: 
     
           
         All 
         None 
         Invert 
     
      
 Legend   
  ▶     ■    ATCGA   
  ∟    ◆   s1KE1   
  ∟    ◆   s1KE2   
  ∟    ◆   s1KE3   
  ∟    ◆   s1KE4   
  ∟    ◆   s1KE5   
  ∟    ◆   s1KE6   
  ∟    ◆   s1KO1   
  ∟    ◆   s1KO2   
  ∟    ◆   s1KO3   
  ∟    ◆   s1KO4   
  ∟    ◆   s1KO5   
  ∟    ◆   s1WE1   
  ∟    ◆   s1WE2   
  ∟    ◆   s1WE3   
  ∟    ◆   s1WE4   
  ∟    ◆   s1WE5   
  ∟    ◆   s1WE6   
  ∟    ◆   s1WT1   
  ∟    ◆   s1WT2   
  ∟    ◆   s1WT3   
  ∟    ◆   s1WT4   
  ∟    ◆   s1WT5   
  ∟    ◆   s1WT6   
  ∟    ◆   s1WT7   
  ∟    ◆   s2KE1   
  ∟    ◆   s2KE2   
  ∟    ◆   s2KE3   
  ∟    ◆   s2KE4   
  ∟    ◆   s2KE5   
  ∟    ◆   s2KE6   
  ∟    ◆   s2KO1   
  ∟    ◆   s2KO2   
  ∟    ◆   s2KO3   
  ∟    ◆   s2KO4   
  ∟    ◆   s2WE1   
  ∟    ◆   s2WE2   
  ∟    ◆   s2WE3   
  ∟    ◆   s2WE4   
  ∟    ◆   s2WE5   
  ∟    ◆   s2WT1   
  ∟    ◆   s2WT2   
  ∟    ◆   s2WT3   
  ∟    ◆   s2WT4   
  ∟    ◆   s2WT5   
  ∟    ◆   s2WT6   
  ∟    ◆   s2WT7   
  ∟    ◆   s3KE1   
  ∟    ◆   s3KE2   
  ∟    ◆   s3KE3   
  ∟    ◆   s3KE4   
  ∟    ◆   s3KE5   
  ∟    ◆   s3KO1   
  ∟    ◆   s3KO2   
  ∟    ◆   s3KO3   
  ∟    ◆   s3KO4   
  ∟    ◆   s3KO5   
  ∟    ◆   s3KO6   
  ∟    ◆   s3WE1   
  ∟    ◆   s3WE2   
  ∟    ◆   s3WE3   
  ∟    ◆   s3WE4   
  ∟    ◆   s3WE5   
  ∟    ◆   s3WE6   
  ∟    ◆   s3WE7   
  ∟    ◆   s3WT1   
  ∟    ◆   s3WT2   
  ∟    ◆   s3WT3   
  ∟    ◆   s3WT4   
  ∟    ◆   s3WT5   
  ∟    ◆   s3WT6   
  ∟    ◆   s3WT7   
  ▶     ■    s1KE1   
  ∟    ◆   s1KE1   
  ▶     ■    s1KE2   
  ∟    ◆   s1KE2   
  ▶     ■    s1KE3   
  ∟    ◆   s1KE3   
  ▶     ■    s1KE4   
  ∟    ◆   s1KE4   
  ▶     ■    s1KE5   
  ∟    ◆   s1KE5   
  ▶     ■    s1KE6   
  ∟    ◆   s1KE6   
  ▶     ■    s1KO1   
  ∟    ◆   s1KO1   
  ▶     ■    s1KO2   
  ∟    ◆   s1KO2   
  ▶     ■    s1KO3   
  ∟    ◆   s1KO3   
  ▶     ■    s1KO4   
  ∟    ◆   s1KO4   
  ▶     ■    s1KO5   
  ∟    ◆   s1KO5   
  ▶     ■    s1WE1   
  ∟    ◆   s1WE1   
  ▶     ■    s1WE2   
  ∟    ◆   s1WE2   
  ▶     ■    s1WE3   
  ∟    ◆   s1WE3   
  ▶     ■    s1WE4   
  ∟    ◆   s1WE4   
  ▶     ■    s1WE5   
  ∟    ◆   s1WE5   
  ▶     ■    s1WE6   
  ∟    ◆   s1WE6   
  ▶     ■    s1WT1   
  ∟    ◆   s1WT1   
  ▶     ■    s1WT2   
  ∟    ◆   s1WT2   
  ▶     ■    s1WT3   
  ∟    ◆   s1WT3   
  ▶     ■    s1WT4   
  ∟    ◆   s1WT4   
  ▶     ■    s1WT5   
  ∟    ◆   s1WT5   
  ▶     ■    s1WT6   
  ∟    ◆   s1WT6   
  ▶     ■    s1WT7   
  ∟    ◆   s1WT7   
  ▶     ■    s2KE1   
  ∟    ◆   s2KE1   
  ▶     ■    s2KE2   
  ∟    ◆   s2KE2   
  ▶     ■    s2KE3   
  ∟    ◆   s2KE3   
  ▶     ■    s2KE4   
  ∟    ◆   s2KE4   
  ▶     ■    s2KE5   
  ∟    ◆   s2KE5   
  ▶     ■    s2KE6   
  ∟    ◆   s2KE6   
  ▶     ■    s2KO1   
  ∟    ◆   s2KO1   
  ▶     ■    s2KO2   
  ∟    ◆   s2KO2   
  ▶     ■    s2KO3   
  ∟    ◆   s2KO3   
  ▶     ■    s2KO4   
  ∟    ◆   s2KO4   
  ▶     ■    s2WE1   
  ∟    ◆   s2WE1   
  ▶     ■    s2WE2   
  ∟    ◆   s2WE2   
  ▶     ■    s2WE3   
  ∟    ◆   s2WE3   
  ▶     ■    s2WE4   
  ∟    ◆   s2WE4   
  ▶     ■    s2WE5   
  ∟    ◆   s2WE5   
  ▶     ■    s2WT1   
  ∟    ◆   s2WT1   
  ▶     ■    s2WT2   
  ∟    ◆   s2WT2   
  ▶     ■    s2WT3   
  ∟    ◆   s2WT3   
  ▶     ■    s2WT4   
  ∟    ◆   s2WT4   
  ▶     ■    s2WT5   
  ∟    ◆   s2WT5   
  ▶     ■    s2WT6   
  ∟    ◆   s2WT6   
  ▶     ■    s2WT7   
  ∟    ◆   s2WT7   
  ▶     ■    s3KE1   
  ∟    ◆   s3KE1   
  ▶     ■    s3KE2   
  ∟    ◆   s3KE2   
  ▶     ■    s3KE3   
  ∟    ◆   s3KE3   
  ▶     ■    s3KE4   
  ∟    ◆   s3KE4   
  ▶     ■    s3KE5   
  ∟    ◆   s3KE5   
  ▶     ■    s3KO1   
  ∟    ◆   s3KO1   
  ▶     ■    s3KO2   
  ∟    ◆   s3KO2   
  ▶     ■    s3KO3   
  ∟    ◆   s3KO3   
  ▶     ■    s3KO4   
  ∟    ◆   s3KO4   
  ▶     ■    s3KO5   
  ∟    ◆   s3KO5   
  ▶     ■    s3KO6   
  ∟    ◆   s3KO6   
  ▶     ■    s3WE1   
  ∟    ◆   s3WE1   
  ▶     ■    s3WE2   
  ∟    ◆   s3WE2   
  ▶     ■    s3WE3   
  ∟    ◆   s3WE3   
  ▶     ■    s3WE4   
  ∟    ◆   s3WE4   
  ▶     ■    s3WE5   
  ∟    ◆   s3WE5   
  ▶     ■    s3WE6   
  ∟    ◆   s3WE6   
  ▶     ■    s3WE7   
  ∟    ◆   s3WE7   
  ▶     ■    s3WT1   
  ∟    ◆   s3WT1   
  ▶     ■    s3WT2   
  ∟    ◆   s3WT2   
  ▶     ■    s3WT3   
  ∟    ◆   s3WT3   
  ▶     ■    s3WT4   
  ∟    ◆   s3WT4   
  ▶     ■    s3WT5   
  ∟    ◆   s3WT5   
  ▶     ■    s3WT6   
  ∟    ◆   s3WT6   
  ▶     ■    s3WT7   
  ∟    ◆   s3WT7   
  ▶     ■    1KE   
  ∟    ◆   s1KE1   
  ∟    ◆   s1KE2   
  ∟    ◆   s1KE3   
  ∟    ◆   s1KE4   
  ∟    ◆   s1KE5   
  ∟    ◆   s1KE6   
  ▶     ■    1KO   
  ∟    ◆   s1KO1   
  ∟    ◆   s1KO2   
  ∟    ◆   s1KO3   
  ∟    ◆   s1KO4   
  ∟    ◆   s1KO5   
  ▶     ■    1WE   
  ∟    ◆   s1WE1   
  ∟    ◆   s1WE2   
  ∟    ◆   s1WE3   
  ∟    ◆   s1WE4   
  ∟    ◆   s1WE5   
  ∟    ◆   s1WE6   
  ▶     ■    1WT   
  ∟    ◆   s1WT1   
  ∟    ◆   s1WT2   
  ∟    ◆   s1WT3   
  ∟    ◆   s1WT4   
  ∟    ◆   s1WT5   
  ∟    ◆   s1WT6   
  ∟    ◆   s1WT7   
  ▶     ■    2KE   
  ∟    ◆   s2KE1   
  ∟    ◆   s2KE2   
  ∟    ◆   s2KE3   
  ∟    ◆   s2KE4   
  ∟    ◆   s2KE5   
  ∟    ◆   s2KE6   
  ▶     ■    2KO   
  ∟    ◆   s2KO1   
  ∟    ◆   s2KO2   
  ∟    ◆   s2KO3   
  ∟    ◆   s2KO4   
  ▶     ■    2WE   
  ∟    ◆   s2WE1   
  ∟    ◆   s2WE2   
  ∟    ◆   s2WE3   
  ∟    ◆   s2WE4   
  ∟    ◆   s2WE5   
  ▶     ■    2WT   
  ∟    ◆   s2WT1   
  ∟    ◆   s2WT2   
  ∟    ◆   s2WT3   
  ∟    ◆   s2WT4   
  ∟    ◆   s2WT5   
  ∟    ◆   s2WT6   
  ∟    ◆   s2WT7   
  ▶     ■    3KE   
  ∟    ◆   s3KE1   
  ∟    ◆   s3KE2   
  ∟    ◆   s3KE3   
  ∟    ◆   s3KE4   
  ∟    ◆   s3KE5   
  ▶     ■    3KO   
  ∟    ◆   s3KO1   
  ∟    ◆   s3KO2   
  ∟    ◆   s3KO3   
  ∟    ◆   s3KO4   
  ∟    ◆   s3KO5   
  ∟    ◆   s3KO6   
  ▶     ■    3WE   
  ∟    ◆   s3WE1   
  ∟    ◆   s3WE2   
  ∟    ◆   s3WE3   
  ∟    ◆   s3WE4   
  ∟    ◆   s3WE5   
  ∟    ◆   s3WE6   
  ∟    ◆   s3WE7   
  ▶     ■    3WT   
  ∟    ◆   s3WT1   
  ∟    ◆   s3WT2   
  ∟    ◆   s3WT3   
  ∟    ◆   s3WT4   
  ∟    ◆   s3WT5   
  ∟    ◆   s3WT6   
  ∟    ◆   s3WT7   
  ▶     ■    AAAAAA   
  ∟    ◆   s1KE1   
  ∟    ◆   s1KE2   
  ∟    ◆   s1KE3   
  ∟    ◆   s1KE4   
  ∟    ◆   s1KE5   
  ∟    ◆   s1KE6   
  ∟    ◆   s1KO1   
  ∟    ◆   s1KO2   
  ∟    ◆   s1KO3   
  ∟    ◆   s1KO4   
  ∟    ◆   s1KO5   
  ∟    ◆   s1WE1   
  ∟    ◆   s1WE2   
  ∟    ◆   s1WE3   
  ∟    ◆   s1WE4   
  ∟    ◆   s1WE5   
  ∟    ◆   s1WE6   
  ∟    ◆   s1WT1   
  ∟    ◆   s1WT2   
  ∟    ◆   s1WT3   
  ∟    ◆   s1WT4   
  ∟    ◆   s1WT5   
  ∟    ◆   s1WT6   
  ∟    ◆   s1WT7   
  ∟    ◆   s2KE1   
  ∟    ◆   s2KE2   
  ∟    ◆   s2KE3   
  ∟    ◆   s2KE4   
  ∟    ◆   s2KE5   
  ∟    ◆   s2KE6   
  ∟    ◆   s2KO1   
  ∟    ◆   s2KO2   
  ∟    ◆   s2KO3   
  ∟    ◆   s2KO4   
  ∟    ◆   s2WE1   
  ∟    ◆   s2WE2   
  ∟    ◆   s2WE3   
  ∟    ◆   s2WE4   
  ∟    ◆   s2WE5   
  ∟    ◆   s2WT1   
  ∟    ◆   s2WT2   
  ∟    ◆   s2WT3   
  ∟    ◆   s2WT4   
  ∟    ◆   s2WT5   
  ∟    ◆   s2WT6   
  ∟    ◆   s2WT7   
  ∟    ◆   s3KE1   
  ∟    ◆   s3KE2   
  ∟    ◆   s3KE3   
  ∟    ◆   s3KE4   
  ∟    ◆   s3KE5   
  ∟    ◆   s3KO1   
  ∟    ◆   s3KO2   
  ∟    ◆   s3KO3   
  ∟    ◆   s3KO4   
  ∟    ◆   s3KO5   
  ∟    ◆   s3KO6   
  ∟    ◆   s3WE1   
  ∟    ◆   s3WE2   
  ∟    ◆   s3WE3   
  ∟    ◆   s3WE4   
  ∟    ◆   s3WE5   
  ∟    ◆   s3WE6   
  ∟    ◆   s3WE7   
  ∟    ◆   s3WT1   
  ∟    ◆   s3WT2   
  ∟    ◆   s3WT3   
  ∟    ◆   s3WT4   
  ∟    ◆   s3WT5   
  ∟    ◆   s3WT6   
  ∟    ◆   s3WT7   
  ▶     ■    s1KE1   
  ∟    ◆   s1KE1   
  ▶     ■    s1KE2   
  ∟    ◆   s1KE2   
  ▶     ■    s1KE3   
  ∟    ◆   s1KE3   
  ▶     ■    s1KE4   
  ∟    ◆   s1KE4   
  ▶     ■    s1KE5   
  ∟    ◆   s1KE5   
  ▶     ■    s1KE6   
  ∟    ◆   s1KE6   
  ▶     ■    s1KO1   
  ∟    ◆   s1KO1   
  ▶     ■    s1KO2   
  ∟    ◆   s1KO2   
  ▶     ■    s1KO3   
  ∟    ◆   s1KO3   
  ▶     ■    s1KO4   
  ∟    ◆   s1KO4   
  ▶     ■    s1KO5   
  ∟    ◆   s1KO5   
  ▶     ■    s1WE1   
  ∟    ◆   s1WE1   
  ▶     ■    s1WE2   
  ∟    ◆   s1WE2   
  ▶     ■    s1WE3   
  ∟    ◆   s1WE3   
  ▶     ■    s1WE4   
  ∟    ◆   s1WE4   
  ▶     ■    s1WE5   
  ∟    ◆   s1WE5   
  ▶     ■    s1WE6   
  ∟    ◆   s1WE6   
  ▶     ■    s1WT1   
  ∟    ◆   s1WT1   
  ▶     ■    s1WT2   
  ∟    ◆   s1WT2   
  ▶     ■    s1WT3   
  ∟    ◆   s1WT3   
  ▶     ■    s1WT4   
  ∟    ◆   s1WT4   
  ▶     ■    s1WT5   
  ∟    ◆   s1WT5   
  ▶     ■    s1WT6   
  ∟    ◆   s1WT6   
  ▶     ■    s1WT7   
  ∟    ◆   s1WT7   
  ▶     ■    s2KE1   
  ∟    ◆   s2KE1   
  ▶     ■    s2KE2   
  ∟    ◆   s2KE2   
  ▶     ■    s2KE3   
  ∟    ◆   s2KE3   
  ▶     ■    s2KE4   
  ∟    ◆   s2KE4   
  ▶     ■    s2KE5   
  ∟    ◆   s2KE5   
  ▶     ■    s2KE6   
  ∟    ◆   s2KE6   
  ▶     ■    s2KO1   
  ∟    ◆   s2KO1   
  ▶     ■    s2KO2   
  ∟    ◆   s2KO2   
  ▶     ■    s2KO3   
  ∟    ◆   s2KO3   
  ▶     ■    s2KO4   
  ∟    ◆   s2KO4   
  ▶     ■    s2WE1   
  ∟    ◆   s2WE1   
  ▶     ■    s2WE2   
  ∟    ◆   s2WE2   
  ▶     ■    s2WE3   
  ∟    ◆   s2WE3   
  ▶     ■    s2WE4   
  ∟    ◆   s2WE4   
  ▶     ■    s2WE5   
  ∟    ◆   s2WE5   
  ▶     ■    s2WT1   
  ∟    ◆   s2WT1   
  ▶     ■    s2WT2   
  ∟    ◆   s2WT2   
  ▶     ■    s2WT3   
  ∟    ◆   s2WT3   
  ▶     ■    s2WT4   
  ∟    ◆   s2WT4   
  ▶     ■    s2WT5   
  ∟    ◆   s2WT5   
  ▶     ■    s2WT6   
  ∟    ◆   s2WT6   
  ▶     ■    s2WT7   
  ∟    ◆   s2WT7   
  ▶     ■    s3KE1   
  ∟    ◆   s3KE1   
  ▶     ■    s3KE2   
  ∟    ◆   s3KE2   
  ▶     ■    s3KE3   
  ∟    ◆   s3KE3   
  ▶     ■    s3KE4   
  ∟    ◆   s3KE4   
  ▶     ■    s3KE5   
  ∟    ◆   s3KE5   
  ▶     ■    s3KO1   
  ∟    ◆   s3KO1   
  ▶     ■    s3KO2   
  ∟    ◆   s3KO2   
  ▶     ■    s3KO3   
  ∟    ◆   s3KO3   
  ▶     ■    s3KO4   
  ∟    ◆   s3KO4   
  ▶     ■    s3KO5   
  ∟    ◆   s3KO5   
  ▶     ■    s3KO6   
  ∟    ◆   s3KO6   
  ▶     ■    s3WE1   
  ∟    ◆   s3WE1   
  ▶     ■    s3WE2   
  ∟    ◆   s3WE2   
  ▶     ■    s3WE3   
  ∟    ◆   s3WE3   
  ▶     ■    s3WE4   
  ∟    ◆   s3WE4   
  ▶     ■    s3WE5   
  ∟    ◆   s3WE5   
  ▶     ■    s3WE6   
  ∟    ◆   s3WE6   
  ▶     ■    s3WE7   
  ∟    ◆   s3WE7   
  ▶     ■    s3WT1   
  ∟    ◆   s3WT1   
  ▶     ■    s3WT2   
  ∟    ◆   s3WT2   
  ▶     ■    s3WT3   
  ∟    ◆   s3WT3   
  ▶     ■    s3WT4   
  ∟    ◆   s3WT4   
  ▶     ■    s3WT5   
  ∟    ◆   s3WT5   
  ▶     ■    s3WT6   
  ∟    ◆   s3WT6   
  ▶     ■    s3WT7   
  ∟    ◆   s3WT7   
  ▶     ■    ATCGA   
  ∟    ◆   s1KE1   
  ∟    ◆   s1KE2   
  ∟    ◆   s1KE3   
  ∟    ◆   s1KE4   
  ∟    ◆   s1KE5   
  ∟    ◆   s1KE6   
  ∟    ◆   s1KO1   
  ∟    ◆   s1KO2   
  ∟    ◆   s1KO3   
  ∟    ◆   s1KO4   
  ∟    ◆   s1KO5   
  ∟    ◆   s1WE1   
  ∟    ◆   s1WE2   
  ∟    ◆   s1WE3   
  ∟    ◆   s1WE4   
  ∟    ◆   s1WE5   
  ∟    ◆   s1WE6   
  ∟    ◆   s1WT1   
  ∟    ◆   s1WT2   
  ∟    ◆   s1WT3   
  ∟    ◆   s1WT4   
  ∟    ◆   s1WT5   
  ∟    ◆   s1WT6   
  ∟    ◆   s1WT7   
  ∟    ◆   s2KE1   
  ∟    ◆   s2KE2   
  ∟    ◆   s2KE3   
  ∟    ◆   s2KE4   
  ∟    ◆   s2KE5   
  ∟    ◆   s2KE6   
  ∟    ◆   s2KO1   
  ∟    ◆   s2KO2   
  ∟    ◆   s2KO3   
  ∟    ◆   s2KO4   
  ∟    ◆   s2WE1   
  ∟    ◆   s2WE2   
  ∟    ◆   s2WE3   
  ∟    ◆   s2WE4   
  ∟    ◆   s2WE5   
  ∟    ◆   s2WT1   
  ∟    ◆   s2WT2   
  ∟    ◆   s2WT3   
  ∟    ◆   s2WT4   
  ∟    ◆   s2WT5   
  ∟    ◆   s2WT6   
  ∟    ◆   s2WT7   
  ∟    ◆   s3KE1   
  ∟    ◆   s3KE2   
  ∟    ◆   s3KE3   
  ∟    ◆   s3KE4   
  ∟    ◆   s3KE5   
  ∟    ◆   s3KO1   
  ∟    ◆   s3KO2   
  ∟    ◆   s3KO3   
  ∟    ◆   s3KO4   
  ∟    ◆   s3KO5   
  ∟    ◆   s3KO6   
  ∟    ◆   s3WE1   
  ∟    ◆   s3WE2   
  ∟    ◆   s3WE3   
  ∟    ◆   s3WE4   
  ∟    ◆   s3WE5   
  ∟    ◆   s3WE6   
  ∟    ◆   s3WE7   
  ∟    ◆   s3WT1   
  ∟    ◆   s3WT2   
  ∟    ◆   s3WT3   
  ∟    ◆   s3WT4   
  ∟    ◆   s3WT5   
  ∟    ◆   s3WT6   
  ∟    ◆   s3WT7   
  ▶     ■    s1KE1   
  ∟    ◆   s1KE1   
  ▶     ■    s1KE2   
  ∟    ◆   s1KE2   
  ▶     ■    s1KE3   
  ∟    ◆   s1KE3   
  ▶     ■    s1KE4   
  ∟    ◆   s1KE4   
  ▶     ■    s1KE5   
  ∟    ◆   s1KE5   
  ▶     ■    s1KE6   
  ∟    ◆   s1KE6   
  ▶     ■    s1KO1   
  ∟    ◆   s1KO1   
  ▶     ■    s1KO2   
  ∟    ◆   s1KO2   
  ▶     ■    s1KO3   
  ∟    ◆   s1KO3   
  ▶     ■    s1KO4   
  ∟    ◆   s1KO4   
  ▶     ■    s1KO5   
  ∟    ◆   s1KO5   
  ▶     ■    s1WE1   
  ∟    ◆   s1WE1   
  ▶     ■    s1WE2   
  ∟    ◆   s1WE2   
  ▶     ■    s1WE3   
  ∟    ◆   s1WE3   
  ▶     ■    s1WE4   
  ∟    ◆   s1WE4   
  ▶     ■    s1WE5   
  ∟    ◆   s1WE5   
  ▶     ■    s1WE6   
  ∟    ◆   s1WE6   
  ▶     ■    s1WT1   
  ∟    ◆   s1WT1   
  ▶     ■    s1WT2   
  ∟    ◆   s1WT2   
  ▶     ■    s1WT3   
  ∟    ◆   s1WT3   
  ▶     ■    s1WT4   
  ∟    ◆   s1WT4   
  ▶     ■    s1WT5   
  ∟    ◆   s1WT5   
  ▶     ■    s1WT6   
  ∟    ◆   s1WT6   
  ▶     ■    s1WT7   
  ∟    ◆   s1WT7   
  ▶     ■    s2KE1   
  ∟    ◆   s2KE1   
  ▶     ■    s2KE2   
  ∟    ◆   s2KE2   
  ▶     ■    s2KE3   
  ∟    ◆   s2KE3   
  ▶     ■    s2KE4   
  ∟    ◆   s2KE4   
  ▶     ■    s2KE5   
  ∟    ◆   s2KE5   
  ▶     ■    s2KE6   
  ∟    ◆   s2KE6   
  ▶     ■    s2KO1   
  ∟    ◆   s2KO1   
  ▶     ■    s2KO2   
  ∟    ◆   s2KO2   
  ▶     ■    s2KO3   
  ∟    ◆   s2KO3   
  ▶     ■    s2KO4   
  ∟    ◆   s2KO4   
  ▶     ■    s2WE1   
  ∟    ◆   s2WE1   
  ▶     ■    s2WE2   
  ∟    ◆   s2WE2   
  ▶     ■    s2WE3   
  ∟    ◆   s2WE3   
  ▶     ■    s2WE4   
  ∟    ◆   s2WE4   
  ▶     ■    s2WE5   
  ∟    ◆   s2WE5   
  ▶     ■    s2WT1   
  ∟    ◆   s2WT1   
  ▶     ■    s2WT2   
  ∟    ◆   s2WT2   
  ▶     ■    s2WT3   
  ∟    ◆   s2WT3   
  ▶     ■    s2WT4   
  ∟    ◆   s2WT4   
  ▶     ■    s2WT5   
  ∟    ◆   s2WT5   
  ▶     ■    s2WT6   
  ∟    ◆   s2WT6   
  ▶     ■    s2WT7   
  ∟    ◆   s2WT7   
  ▶     ■    s3KE1   
  ∟    ◆   s3KE1   
  ▶     ■    s3KE2   
  ∟    ◆   s3KE2   
  ▶     ■    s3KE3   
  ∟    ◆   s3KE3   
  ▶     ■    s3KE4   
  ∟    ◆   s3KE4   
  ▶     ■    s3KE5   
  ∟    ◆   s3KE5   
  ▶     ■    s3KO1   
  ∟    ◆   s3KO1   
  ▶     ■    s3KO2   
  ∟    ◆   s3KO2   
  ▶     ■    s3KO3   
  ∟    ◆   s3KO3   
  ▶     ■    s3KO4   
  ∟    ◆   s3KO4   
  ▶     ■    s3KO5   
  ∟    ◆   s3KO5   
  ▶     ■    s3KO6   
  ∟    ◆   s3KO6   
  ▶     ■    s3WE1   
  ∟    ◆   s3WE1   
  ▶     ■    s3WE2   
  ∟    ◆   s3WE2   
  ▶     ■    s3WE3   
  ∟    ◆   s3WE3   
  ▶     ■    s3WE4   
  ∟    ◆   s3WE4   
  ▶     ■    s3WE5   
  ∟    ◆   s3WE5   
  ▶     ■    s3WE6   
  ∟    ◆   s3WE6   
  ▶     ■    s3WE7   
  ∟    ◆   s3WE7   
  ▶     ■    s3WT1   
  ∟    ◆   s3WT1   
  ▶     ■    s3WT2   
  ∟    ◆   s3WT2   
  ▶     ■    s3WT3   
  ∟    ◆   s3WT3   
  ▶     ■    s3WT4   
  ∟    ◆   s3WT4   
  ▶     ■    s3WT5   
  ∟    ◆   s3WT5   
  ▶     ■    s3WT6   
  ∟    ◆   s3WT6   
  ▶     ■    s3WT7   
  ∟    ◆   s3WT7   
  ▶     ■    1KE   
  ∟    ◆   s1KE1   
  ∟    ◆   s1KE2   
  ∟    ◆   s1KE3   
  ∟    ◆   s1KE4   
  ∟    ◆   s1KE5   
  ∟    ◆   s1KE6   
  ▶     ■    1KO   
  ∟    ◆   s1KO1   
  ∟    ◆   s1KO2   
  ∟    ◆   s1KO3   
  ∟    ◆   s1KO4   
  ∟    ◆   s1KO5   
  ▶     ■    1WE   
  ∟    ◆   s1WE1   
  ∟    ◆   s1WE2   
  ∟    ◆   s1WE3   
  ∟    ◆   s1WE4   
  ∟    ◆   s1WE5   
  ∟    ◆   s1WE6   
  ▶     ■    1WT   
  ∟    ◆   s1WT1   
  ∟    ◆   s1WT2   
  ∟    ◆   s1WT3   
  ∟    ◆   s1WT4   
  ∟    ◆   s1WT5   
  ∟    ◆   s1WT6   
  ∟    ◆   s1WT7   
  ▶     ■    2KE   
  ∟    ◆   s2KE1   
  ∟    ◆   s2KE2   
  ∟    ◆   s2KE3   
  ∟    ◆   s2KE4   
  ∟    ◆   s2KE5   
  ∟    ◆   s2KE6   
  ▶     ■    2KO   
  ∟    ◆   s2KO1   
  ∟    ◆   s2KO2   
  ∟    ◆   s2KO3   
  ∟    ◆   s2KO4   
  ▶     ■    2WE   
  ∟    ◆   s2WE1   
  ∟    ◆   s2WE2   
  ∟    ◆   s2WE3   
  ∟    ◆   s2WE4   
  ∟    ◆   s2WE5   
  ▶     ■    2WT   
  ∟    ◆   s2WT1   
  ∟    ◆   s2WT2   
  ∟    ◆   s2WT3   
  ∟    ◆   s2WT4   
  ∟    ◆   s2WT5   
  ∟    ◆   s2WT6   
  ∟    ◆   s2WT7   
  ▶     ■    3KE   
  ∟    ◆   s3KE1   
  ∟    ◆   s3KE2   
  ∟    ◆   s3KE3   
  ∟    ◆   s3KE4   
  ∟    ◆   s3KE5   
  ▶     ■    3KO   
  ∟    ◆   s3KO1   
  ∟    ◆   s3KO2   
  ∟    ◆   s3KO3   
  ∟    ◆   s3KO4   
  ∟    ◆   s3KO5   
  ∟    ◆   s3KO6   
  ▶     ■    3WE   
  ∟    ◆   s3WE1   
  ∟    ◆   s3WE2   
  ∟    ◆   s3WE3   
  ∟    ◆   s3WE4   
  ∟    ◆   s3WE5   
  ∟    ◆   s3WE6   
  ∟    ◆   s3WE7   
  ▶     ■    3WT   
  ∟    ◆   s3WT1   
  ∟    ◆   s3WT2   
  ∟    ◆   s3WT3   
  ∟    ◆   s3WT4   
  ∟    ◆   s3WT5   
  ∟    ◆   s3WT6   
  ∟    ◆   s3WT7   
  ▶     ■    AAAAAA   
  ∟    ◆   s1KE1   
  ∟    ◆   s1KE2   
  ∟    ◆   s1KE3   
  ∟    ◆   s1KE4   
  ∟    ◆   s1KE5   
  ∟    ◆   s1KE6   
  ∟    ◆   s1KO1   
  ∟    ◆   s1KO2   
  ∟    ◆   s1KO3   
  ∟    ◆   s1KO4   
  ∟    ◆   s1KO5   
  ∟    ◆   s1WE1   
  ∟    ◆   s1WE2   
  ∟    ◆   s1WE3   
  ∟    ◆   s1WE4   
  ∟    ◆   s1WE5   
  ∟    ◆   s1WE6   
  ∟    ◆   s1WT1   
  ∟    ◆   s1WT2   
  ∟    ◆   s1WT3   
  ∟    ◆   s1WT4   
  ∟    ◆   s1WT5   
  ∟    ◆   s1WT6   
  ∟    ◆   s1WT7   
  ∟    ◆   s2KE1   
  ∟    ◆   s2KE2   
  ∟    ◆   s2KE3   
  ∟    ◆   s2KE4   
  ∟    ◆   s2KE5   
  ∟    ◆   s2KE6   
  ∟    ◆   s2KO1   
  ∟    ◆   s2KO2   
  ∟    ◆   s2KO3   
  ∟    ◆   s2KO4   
  ∟    ◆   s2WE1   
  ∟    ◆   s2WE2   
  ∟    ◆   s2WE3   
  ∟    ◆   s2WE4   
  ∟    ◆   s2WE5   
  ∟    ◆   s2WT1   
  ∟    ◆   s2WT2   
  ∟    ◆   s2WT3   
  ∟    ◆   s2WT4   
  ∟    ◆   s2WT5   
  ∟    ◆   s2WT6   
  ∟    ◆   s2WT7   
  ∟    ◆   s3KE1   
  ∟    ◆   s3KE2   
  ∟    ◆   s3KE3   
  ∟    ◆   s3KE4   
  ∟    ◆   s3KE5   
  ∟    ◆   s3KO1   
  ∟    ◆   s3KO2   
  ∟    ◆   s3KO3   
  ∟    ◆   s3KO4   
  ∟    ◆   s3KO5   
  ∟    ◆   s3KO6   
  ∟    ◆   s3WE1   
  ∟    ◆   s3WE2   
  ∟    ◆   s3WE3   
  ∟    ◆   s3WE4   
  ∟    ◆   s3WE5   
  ∟    ◆   s3WE6   
  ∟    ◆   s3WE7   
  ∟    ◆   s3WT1   
  ∟    ◆   s3WT2   
  ∟    ◆   s3WT3   
  ∟    ◆   s3WT4   
  ∟    ◆   s3WT5   
  ∟    ◆   s3WT6   
  ∟    ◆   s3WT7   
  ▶     ■    s1KE1   
  ∟    ◆   s1KE1   
  ▶     ■    s1KE2   
  ∟    ◆   s1KE2   
  ▶     ■    s1KE3   
  ∟    ◆   s1KE3   
  ▶     ■    s1KE4   
  ∟    ◆   s1KE4   
  ▶     ■    s1KE5   
  ∟    ◆   s1KE5   
  ▶     ■    s1KE6   
  ∟    ◆   s1KE6   
  ▶     ■    s1KO1   
  ∟    ◆   s1KO1   
  ▶     ■    s1KO2   
  ∟    ◆   s1KO2   
  ▶     ■    s1KO3   
  ∟    ◆   s1KO3   
  ▶     ■    s1KO4   
  ∟    ◆   s1KO4   
  ▶     ■    s1KO5   
  ∟    ◆   s1KO5   
  ▶     ■    s1WE1   
  ∟    ◆   s1WE1   
  ▶     ■    s1WE2   
  ∟    ◆   s1WE2   
  ▶     ■    s1WE3   
  ∟    ◆   s1WE3   
  ▶     ■    s1WE4   
  ∟    ◆   s1WE4   
  ▶     ■    s1WE5   
  ∟    ◆   s1WE5   
  ▶     ■    s1WE6   
  ∟    ◆   s1WE6   
  ▶     ■    s1WT1   
  ∟    ◆   s1WT1   
  ▶     ■    s1WT2   
  ∟    ◆   s1WT2   
  ▶     ■    s1WT3   
  ∟    ◆   s1WT3   
  ▶     ■    s1WT4   
  ∟    ◆   s1WT4   
  ▶     ■    s1WT5   
  ∟    ◆   s1WT5   
  ▶     ■    s1WT6   
  ∟    ◆   s1WT6   
  ▶     ■    s1WT7   
  ∟    ◆   s1WT7   
  ▶     ■    s2KE1   
  ∟    ◆   s2KE1   
  ▶     ■    s2KE2   
  ∟    ◆   s2KE2   
  ▶     ■    s2KE3   
  ∟    ◆   s2KE3   
  ▶     ■    s2KE4   
  ∟    ◆   s2KE4   
  ▶     ■    s2KE5   
  ∟    ◆   s2KE5   
  ▶     ■    s2KE6   
  ∟    ◆   s2KE6   
  ▶     ■    s2KO1   
  ∟    ◆   s2KO1   
  ▶     ■    s2KO2   
  ∟    ◆   s2KO2   
  ▶     ■    s2KO3   
  ∟    ◆   s2KO3   
  ▶     ■    s2KO4   
  ∟    ◆   s2KO4   
  ▶     ■    s2WE1   
  ∟    ◆   s2WE1   
  ▶     ■    s2WE2   
  ∟    ◆   s2WE2   
  ▶     ■    s2WE3   
  ∟    ◆   s2WE3   
  ▶     ■    s2WE4   
  ∟    ◆   s2WE4   
  ▶     ■    s2WE5   
  ∟    ◆   s2WE5   
  ▶     ■    s2WT1   
  ∟    ◆   s2WT1   
  ▶     ■    s2WT2   
  ∟    ◆   s2WT2   
  ▶     ■    s2WT3   
  ∟    ◆   s2WT3   
  ▶     ■    s2WT4   
  ∟    ◆   s2WT4   
  ▶     ■    s2WT5   
  ∟    ◆   s2WT5   
  ▶     ■    s2WT6   
  ∟    ◆   s2WT6   
  ▶     ■    s2WT7   
  ∟    ◆   s2WT7   
  ▶     ■    s3KE1   
  ∟    ◆   s3KE1   
  ▶     ■    s3KE2   
  ∟    ◆   s3KE2   
  ▶     ■    s3KE3   
  ∟    ◆   s3KE3   
  ▶     ■    s3KE4   
  ∟    ◆   s3KE4   
  ▶     ■    s3KE5   
  ∟    ◆   s3KE5   
  ▶     ■    s3KO1   
  ∟    ◆   s3KO1   
  ▶     ■    s3KO2   
  ∟    ◆   s3KO2   
  ▶     ■    s3KO3   
  ∟    ◆   s3KO3   
  ▶     ■    s3KO4   
  ∟    ◆   s3KO4   
  ▶     ■    s3KO5   
  ∟    ◆   s3KO5   
  ▶     ■    s3KO6   
  ∟    ◆   s3KO6   
  ▶     ■    s3WE1   
  ∟    ◆   s3WE1   
  ▶     ■    s3WE2   
  ∟    ◆   s3WE2   
  ▶     ■    s3WE3   
  ∟    ◆   s3WE3   
  ▶     ■    s3WE4   
  ∟    ◆   s3WE4   
  ▶     ■    s3WE5   
  ∟    ◆   s3WE5   
  ▶     ■    s3WE6   
  ∟    ◆   s3WE6   
  ▶     ■    s3WE7   
  ∟    ◆   s3WE7   
  ▶     ■    s3WT1   
  ∟    ◆   s3WT1   
  ▶     ■    s3WT2   
  ∟    ◆   s3WT2   
  ▶     ■    s3WT3   
  ∟    ◆   s3WT3   
  ▶     ■    s3WT4   
  ∟    ◆   s3WT4   
  ▶     ■    s3WT5   
  ∟    ◆   s3WT5   
  ▶     ■    s3WT6   
  ∟    ◆   s3WT6   
  ▶     ■    s3WT7   
  ∟    ◆   s3WT7   
  ▶     ■    ATCGA   
  ∟    ◆   s1KE1   
  ∟    ◆   s1KE2   
  ∟    ◆   s1KE3   
  ∟    ◆   s1KE4   
  ∟    ◆   s1KE5   
  ∟    ◆   s1KE6   
  ∟    ◆   s1KO1   
  ∟    ◆   s1KO2   
  ∟    ◆   s1KO3   
  ∟    ◆   s1KO4   
  ∟    ◆   s1KO5   
  ∟    ◆   s1WE1   
  ∟    ◆   s1WE2   
  ∟    ◆   s1WE3   
  ∟    ◆   s1WE4   
  ∟    ◆   s1WE5   
  ∟    ◆   s1WE6   
  ∟    ◆   s1WT1   
  ∟    ◆   s1WT2   
  ∟    ◆   s1WT3   
  ∟    ◆   s1WT4   
  ∟    ◆   s1WT5   
  ∟    ◆   s1WT6   
  ∟    ◆   s1WT7   
  ∟    ◆   s2KE1   
  ∟    ◆   s2KE2   
  ∟    ◆   s2KE3   
  ∟    ◆   s2KE4   
  ∟    ◆   s2KE5   
  ∟    ◆   s2KE6   
  ∟    ◆   s2KO1   
  ∟    ◆   s2KO2   
  ∟    ◆   s2KO3   
  ∟    ◆   s2KO4   
  ∟    ◆   s2WE1   
  ∟    ◆   s2WE2   
  ∟    ◆   s2WE3   
  ∟    ◆   s2WE4   
  ∟    ◆   s2WE5   
  ∟    ◆   s2WT1   
  ∟    ◆   s2WT2   
  ∟    ◆   s2WT3   
  ∟    ◆   s2WT4   
  ∟    ◆   s2WT5   
  ∟    ◆   s2WT6   
  ∟    ◆   s2WT7   
  ∟    ◆   s3KE1   
  ∟    ◆   s3KE2   
  ∟    ◆   s3KE3   
  ∟    ◆   s3KE4   
  ∟    ◆   s3KE5   
  ∟    ◆   s3KO1   
  ∟    ◆   s3KO2   
  ∟    ◆   s3KO3   
  ∟    ◆   s3KO4   
  ∟    ◆   s3KO5   
  ∟    ◆   s3KO6   
  ∟    ◆   s3WE1   
  ∟    ◆   s3WE2   
  ∟    ◆   s3WE3   
  ∟    ◆   s3WE4   
  ∟    ◆   s3WE5   
  ∟    ◆   s3WE6   
  ∟    ◆   s3WE7   
  ∟    ◆   s3WT1   
  ∟    ◆   s3WT2   
  ∟    ◆   s3WT3   
  ∟    ◆   s3WT4   
  ∟    ◆   s3WT5   
  ∟    ◆   s3WT6   
  ∟    ◆   s3WT7   
  ▶     ■    s1KE1   
  ∟    ◆   s1KE1   
  ▶     ■    s1KE2   
  ∟    ◆   s1KE2   
  ▶     ■    s1KE3   
  ∟    ◆   s1KE3   
  ▶     ■    s1KE4   
  ∟    ◆   s1KE4   
  ▶     ■    s1KE5   
  ∟    ◆   s1KE5   
  ▶     ■    s1KE6   
  ∟    ◆   s1KE6   
  ▶     ■    s1KO1   
  ∟    ◆   s1KO1   
  ▶     ■    s1KO2   
  ∟    ◆   s1KO2   
  ▶     ■    s1KO3   
  ∟    ◆   s1KO3   
  ▶     ■    s1KO4   
  ∟    ◆   s1KO4   
  ▶     ■    s1KO5   
  ∟    ◆   s1KO5   
  ▶     ■    s1WE1   
  ∟    ◆   s1WE1   
  ▶     ■    s1WE2   
  ∟    ◆   s1WE2   
  ▶     ■    s1WE3   
  ∟    ◆   s1WE3   
  ▶     ■    s1WE4   
  ∟    ◆   s1WE4   
  ▶     ■    s1WE5   
  ∟    ◆   s1WE5   
  ▶     ■    s1WE6   
  ∟    ◆   s1WE6   
  ▶     ■    s1WT1   
  ∟    ◆   s1WT1   
  ▶     ■    s1WT2   
  ∟    ◆   s1WT2   
  ▶     ■    s1WT3   
  ∟    ◆   s1WT3   
  ▶     ■    s1WT4   
  ∟    ◆   s1WT4   
  ▶     ■    s1WT5   
  ∟    ◆   s1WT5   
  ▶     ■    s1WT6   
  ∟    ◆   s1WT6   
  ▶     ■    s1WT7   
  ∟    ◆   s1WT7   
  ▶     ■    s2KE1   
  ∟    ◆   s2KE1   
  ▶     ■    s2KE2   
  ∟    ◆   s2KE2   
  ▶     ■    s2KE3   
  ∟    ◆   s2KE3   
  ▶     ■    s2KE4   
  ∟    ◆   s2KE4   
  ▶     ■    s2KE5   
  ∟    ◆   s2KE5   
  ▶     ■    s2KE6   
  ∟    ◆   s2KE6   
  ▶     ■    s2KO1   
  ∟    ◆   s2KO1   
  ▶     ■    s2KO2   
  ∟    ◆   s2KO2   
  ▶     ■    s2KO3   
  ∟    ◆   s2KO3   
  ▶     ■    s2KO4   
  ∟    ◆   s2KO4   
  ▶     ■    s2WE1   
  ∟    ◆   s2WE1   
  ▶     ■    s2WE2   
  ∟    ◆   s2WE2   
  ▶     ■    s2WE3   
  ∟    ◆   s2WE3   
  ▶     ■    s2WE4   
  ∟    ◆   s2WE4   
  ▶     ■    s2WE5   
  ∟    ◆   s2WE5   
  ▶     ■    s2WT1   
  ∟    ◆   s2WT1   
  ▶     ■    s2WT2   
  ∟    ◆   s2WT2   
  ▶     ■    s2WT3   
  ∟    ◆   s2WT3   
  ▶     ■    s2WT4   
  ∟    ◆   s2WT4   
  ▶     ■    s2WT5   
  ∟    ◆   s2WT5   
  ▶     ■    s2WT6   
  ∟    ◆   s2WT6   
  ▶     ■    s2WT7   
  ∟    ◆   s2WT7   
  ▶     ■    s3KE1   
  ∟    ◆   s3KE1   
  ▶     ■    s3KE2   
  ∟    ◆   s3KE2   
  ▶     ■    s3KE3   
  ∟    ◆   s3KE3   
  ▶     ■    s3KE4   
  ∟    ◆   s3KE4   
  ▶     ■    s3KE5   
  ∟    ◆   s3KE5   
  ▶     ■    s3KO1   
  ∟    ◆   s3KO1   
  ▶     ■    s3KO2   
  ∟    ◆   s3KO2   
  ▶     ■    s3KO3   
  ∟    ◆   s3KO3   
  ▶     ■    s3KO4   
  ∟    ◆   s3KO4   
  ▶     ■    s3KO5   
  ∟    ◆   s3KO5   
  ▶     ■    s3KO6   
  ∟    ◆   s3KO6   
  ▶     ■    s3WE1   
  ∟    ◆   s3WE1   
  ▶     ■    s3WE2   
  ∟    ◆   s3WE2   
  ▶     ■    s3WE3   
  ∟    ◆   s3WE3   
  ▶     ■    s3WE4   
  ∟    ◆   s3WE4   
  ▶     ■    s3WE5   
  ∟    ◆   s3WE5   
  ▶     ■    s3WE6   
  ∟    ◆   s3WE6   
  ▶     ■    s3WE7   
  ∟    ◆   s3WE7   
  ▶     ■    s3WT1   
  ∟    ◆   s3WT1   
  ▶     ■    s3WT2   
  ∟    ◆   s3WT2   
  ▶     ■    s3WT3   
  ∟    ◆   s3WT3   
  ▶     ■    s3WT4   
  ∟    ◆   s3WT4   
  ▶     ■    s3WT5   
  ∟    ◆   s3WT5   
  ▶     ■    s3WT6   
  ∟    ◆   s3WT6   
  ▶     ■    s3WT7   
  ∟    ◆   s3WT7   
  ▶     ■    1KE   
  ∟    ◆   s1KE1   
  ∟    ◆   s1KE2   
  ∟    ◆   s1KE3   
  ∟    ◆   s1KE4   
  ∟    ◆   s1KE5   
  ∟    ◆   s1KE6   
  ▶     ■    1KO   
  ∟    ◆   s1KO1   
  ∟    ◆   s1KO2   
  ∟    ◆   s1KO3   
  ∟    ◆   s1KO4   
  ∟    ◆   s1KO5   
  ▶     ■    1WE   
  ∟    ◆   s1WE1   
  ∟    ◆   s1WE2   
  ∟    ◆   s1WE3   
  ∟    ◆   s1WE4   
  ∟    ◆   s1WE5   
  ∟    ◆   s1WE6   
  ▶     ■    1WT   
  ∟    ◆   s1WT1   
  ∟    ◆   s1WT2   
  ∟    ◆   s1WT3   
  ∟    ◆   s1WT4   
  ∟    ◆   s1WT5   
  ∟    ◆   s1WT6   
  ∟    ◆   s1WT7   
  ▶     ■    2KE   
  ∟    ◆   s2KE1   
  ∟    ◆   s2KE2   
  ∟    ◆   s2KE3   
  ∟    ◆   s2KE4   
  ∟    ◆   s2KE5   
  ∟    ◆   s2KE6   
  ▶     ■    2KO   
  ∟    ◆   s2KO1   
  ∟    ◆   s2KO2   
  ∟    ◆   s2KO3   
  ∟    ◆   s2KO4   
  ▶     ■    2WE   
  ∟    ◆   s2WE1   
  ∟    ◆   s2WE2   
  ∟    ◆   s2WE3   
  ∟    ◆   s2WE4   
  ∟    ◆   s2WE5   
  ▶     ■    2WT   
  ∟    ◆   s2WT1   
  ∟    ◆   s2WT2   
  ∟    ◆   s2WT3   
  ∟    ◆   s2WT4   
  ∟    ◆   s2WT5   
  ∟    ◆   s2WT6   
  ∟    ◆   s2WT7   
  ▶     ■    3KE   
  ∟    ◆   s3KE1   
  ∟    ◆   s3KE2   
  ∟    ◆   s3KE3   
  ∟    ◆   s3KE4   
  ∟    ◆   s3KE5   
  ▶     ■    3KO   
  ∟    ◆   s3KO1   
  ∟    ◆   s3KO2   
  ∟    ◆   s3KO3   
  ∟    ◆   s3KO4   
  ∟    ◆   s3KO5   
  ∟    ◆   s3KO6   
  ▶     ■    3WE   
  ∟    ◆   s3WE1   
  ∟    ◆   s3WE2   
  ∟    ◆   s3WE3   
  ∟    ◆   s3WE4   
  ∟    ◆   s3WE5   
  ∟    ◆   s3WE6   
  ∟    ◆   s3WE7   
  ▶     ■    3WT   
  ∟    ◆   s3WT1   
  ∟    ◆   s3WT2   
  ∟    ◆   s3WT3   
  ∟    ◆   s3WT4   
  ∟    ◆   s3WT5   
  ∟    ◆   s3WT6   
  ∟    ◆   s3WT7   
  ▶     ■    AAAAAA   
  ∟    ◆   s1KE1   
  ∟    ◆   s1KE2   
  ∟    ◆   s1KE3   
  ∟    ◆   s1KE4   
  ∟    ◆   s1KE5   
  ∟    ◆   s1KE6   
  ∟    ◆   s1KO1   
  ∟    ◆   s1KO2   
  ∟    ◆   s1KO3   
  ∟    ◆   s1KO4   
  ∟    ◆   s1KO5   
  ∟    ◆   s1WE1   
  ∟    ◆   s1WE2   
  ∟    ◆   s1WE3   
  ∟    ◆   s1WE4   
  ∟    ◆   s1WE5   
  ∟    ◆   s1WE6   
  ∟    ◆   s1WT1   
  ∟    ◆   s1WT2   
  ∟    ◆   s1WT3   
  ∟    ◆   s1WT4   
  ∟    ◆   s1WT5   
  ∟    ◆   s1WT6   
  ∟    ◆   s1WT7   
  ∟    ◆   s2KE1   
  ∟    ◆   s2KE2   
  ∟    ◆   s2KE3   
  ∟    ◆   s2KE4   
  ∟    ◆   s2KE5   
  ∟    ◆   s2KE6   
  ∟    ◆   s2KO1   
  ∟    ◆   s2KO2   
  ∟    ◆   s2KO3   
  ∟    ◆   s2KO4   
  ∟    ◆   s2WE1   
  ∟    ◆   s2WE2   
  ∟    ◆   s2WE3   
  ∟    ◆   s2WE4   
  ∟    ◆   s2WE5   
  ∟    ◆   s2WT1   
  ∟    ◆   s2WT2   
  ∟    ◆   s2WT3   
  ∟    ◆   s2WT4   
  ∟    ◆   s2WT5   
  ∟    ◆   s2WT6   
  ∟    ◆   s2WT7   
  ∟    ◆   s3KE1   
  ∟    ◆   s3KE2   
  ∟    ◆   s3KE3   
  ∟    ◆   s3KE4   
  ∟    ◆   s3KE5   
  ∟    ◆   s3KO1   
  ∟    ◆   s3KO2   
  ∟    ◆   s3KO3   
  ∟    ◆   s3KO4   
  ∟    ◆   s3KO5   
  ∟    ◆   s3KO6   
  ∟    ◆   s3WE1   
  ∟    ◆   s3WE2   
  ∟    ◆   s3WE3   
  ∟    ◆   s3WE4   
  ∟    ◆   s3WE5   
  ∟    ◆   s3WE6   
  ∟    ◆   s3WE7   
  ∟    ◆   s3WT1   
  ∟    ◆   s3WT2   
  ∟    ◆   s3WT3   
  ∟    ◆   s3WT4   
  ∟    ◆   s3WT5   
  ∟    ◆   s3WT6   
  ∟    ◆   s3WT7   
  ▶     ■    s1KE1   
  ∟    ◆   s1KE1   
  ▶     ■    s1KE2   
  ∟    ◆   s1KE2   
  ▶     ■    s1KE3   
  ∟    ◆   s1KE3   
  ▶     ■    s1KE4   
  ∟    ◆   s1KE4   
  ▶     ■    s1KE5   
  ∟    ◆   s1KE5   
  ▶     ■    s1KE6   
  ∟    ◆   s1KE6   
  ▶     ■    s1KO1   
  ∟    ◆   s1KO1   
  ▶     ■    s1KO2   
  ∟    ◆   s1KO2   
  ▶     ■    s1KO3   
  ∟    ◆   s1KO3   
  ▶     ■    s1KO4   
  ∟    ◆   s1KO4   
  ▶     ■    s1KO5   
  ∟    ◆   s1KO5   
  ▶     ■    s1WE1   
  ∟    ◆   s1WE1   
  ▶     ■    s1WE2   
  ∟    ◆   s1WE2   
  ▶     ■    s1WE3   
  ∟    ◆   s1WE3   
  ▶     ■    s1WE4   
  ∟    ◆   s1WE4   
  ▶     ■    s1WE5   
  ∟    ◆   s1WE5   
  ▶     ■    s1WE6   
  ∟    ◆   s1WE6   
  ▶     ■    s1WT1   
  ∟    ◆   s1WT1   
  ▶     ■    s1WT2   
  ∟    ◆   s1WT2   
  ▶     ■    s1WT3   
  ∟    ◆   s1WT3   
  ▶     ■    s1WT4   
  ∟    ◆   s1WT4   
  ▶     ■    s1WT5   
  ∟    ◆   s1WT5   
  ▶     ■    s1WT6   
  ∟    ◆   s1WT6   
  ▶     ■    s1WT7   
  ∟    ◆   s1WT7   
  ▶     ■    s2KE1   
  ∟    ◆   s2KE1   
  ▶     ■    s2KE2   
  ∟    ◆   s2KE2   
  ▶     ■    s2KE3   
  ∟    ◆   s2KE3   
  ▶     ■    s2KE4   
  ∟    ◆   s2KE4   
  ▶     ■    s2KE5   
  ∟    ◆   s2KE5   
  ▶     ■    s2KE6   
  ∟    ◆   s2KE6   
  ▶     ■    s2KO1   
  ∟    ◆   s2KO1   
  ▶     ■    s2KO2   
  ∟    ◆   s2KO2   
  ▶     ■    s2KO3   
  ∟    ◆   s2KO3   
  ▶     ■    s2KO4   
  ∟    ◆   s2KO4   
  ▶     ■    s2WE1   
  ∟    ◆   s2WE1   
  ▶     ■    s2WE2   
  ∟    ◆   s2WE2   
  ▶     ■    s2WE3   
  ∟    ◆   s2WE3   
  ▶     ■    s2WE4   
  ∟    ◆   s2WE4   
  ▶     ■    s2WE5   
  ∟    ◆   s2WE5   
  ▶     ■    s2WT1   
  ∟    ◆   s2WT1   
  ▶     ■    s2WT2   
  ∟    ◆   s2WT2   
  ▶     ■    s2WT3   
  ∟    ◆   s2WT3   
  ▶     ■    s2WT4   
  ∟    ◆   s2WT4   
  ▶     ■    s2WT5   
  ∟    ◆   s2WT5   
  ▶     ■    s2WT6   
  ∟    ◆   s2WT6   
  ▶     ■    s2WT7   
  ∟    ◆   s2WT7   
  ▶     ■    s3KE1   
  ∟    ◆   s3KE1   
  ▶     ■    s3KE2   
  ∟    ◆   s3KE2   
  ▶     ■    s3KE3   
  ∟    ◆   s3KE3   
  ▶     ■    s3KE4   
  ∟    ◆   s3KE4   
  ▶     ■    s3KE5   
  ∟    ◆   s3KE5   
  ▶     ■    s3KO1   
  ∟    ◆   s3KO1   
  ▶     ■    s3KO2   
  ∟    ◆   s3KO2   
  ▶     ■    s3KO3   
  ∟    ◆   s3KO3   
  ▶     ■    s3KO4   
  ∟    ◆   s3KO4   
  ▶     ■    s3KO5   
  ∟    ◆   s3KO5   
  ▶     ■    s3KO6   
  ∟    ◆   s3KO6   
  ▶     ■    s3WE1   
  ∟    ◆   s3WE1   
  ▶     ■    s3WE2   
  ∟    ◆   s3WE2   
  ▶     ■    s3WE3   
  ∟    ◆   s3WE3   
  ▶     ■    s3WE4   
  ∟    ◆   s3WE4   
  ▶     ■    s3WE5   
  ∟    ◆   s3WE5   
  ▶     ■    s3WE6   
  ∟    ◆   s3WE6   
  ▶     ■    s3WE7   
  ∟    ◆   s3WE7   
  ▶     ■    s3WT1   
  ∟    ◆   s3WT1   
  ▶     ■    s3WT2   
  ∟    ◆   s3WT2   
  ▶     ■    s3WT3   
  ∟    ◆   s3WT3   
  ▶     ■    s3WT4   
  ∟    ◆   s3WT4   
  ▶     ■    s3WT5   
  ∟    ◆   s3WT5   
  ▶     ■    s3WT6   
  ∟    ◆   s3WT6   
  ▶     ■    s3WT7   
  ∟    ◆   s3WT7   
  ▶     ■    ATCGA   
  ∟    ◆   s1KE1   
  ∟    ◆   s1KE2   
  ∟    ◆   s1KE3   
  ∟    ◆   s1KE4   
  ∟    ◆   s1KE5   
  ∟    ◆   s1KE6   
  ∟    ◆   s1KO1   
  ∟    ◆   s1KO2   
  ∟    ◆   s1KO3   
  ∟    ◆   s1KO4   
  ∟    ◆   s1KO5   
  ∟    ◆   s1WE1   
  ∟    ◆   s1WE2   
  ∟    ◆   s1WE3   
  ∟    ◆   s1WE4   
  ∟    ◆   s1WE5   
  ∟    ◆   s1WE6   
  ∟    ◆   s1WT1   
  ∟    ◆   s1WT2   
  ∟    ◆   s1WT3   
  ∟    ◆   s1WT4   
  ∟    ◆   s1WT5   
  ∟    ◆   s1WT6   
  ∟    ◆   s1WT7   
  ∟    ◆   s2KE1   
  ∟    ◆   s2KE2   
  ∟    ◆   s2KE3   
  ∟    ◆   s2KE4   
  ∟    ◆   s2KE5   
  ∟    ◆   s2KE6   
  ∟    ◆   s2KO1   
  ∟    ◆   s2KO2   
  ∟    ◆   s2KO3   
  ∟    ◆   s2KO4   
  ∟    ◆   s2WE1   
  ∟    ◆   s2WE2   
  ∟    ◆   s2WE3   
  ∟    ◆   s2WE4   
  ∟    ◆   s2WE5   
  ∟    ◆   s2WT1   
  ∟    ◆   s2WT2   
  ∟    ◆   s2WT3   
  ∟    ◆   s2WT4   
  ∟    ◆   s2WT5   
  ∟    ◆   s2WT6   
  ∟    ◆   s2WT7   
  ∟    ◆   s3KE1   
  ∟    ◆   s3KE2   
  ∟    ◆   s3KE3   
  ∟    ◆   s3KE4   
  ∟    ◆   s3KE5   
  ∟    ◆   s3KO1   
  ∟    ◆   s3KO2   
  ∟    ◆   s3KO3   
  ∟    ◆   s3KO4   
  ∟    ◆   s3KO5   
  ∟    ◆   s3KO6   
  ∟    ◆   s3WE1   
  ∟    ◆   s3WE2   
  ∟    ◆   s3WE3   
  ∟    ◆   s3WE4   
  ∟    ◆   s3WE5   
  ∟    ◆   s3WE6   
  ∟    ◆   s3WE7   
  ∟    ◆   s3WT1   
  ∟    ◆   s3WT2   
  ∟    ◆   s3WT3   
  ∟    ◆   s3WT4   
  ∟    ◆   s3WT5   
  ∟    ◆   s3WT6   
  ∟    ◆   s3WT7   
  ▶     ■    s1KE1   
  ∟    ◆   s1KE1   
  ▶     ■    s1KE2   
  ∟    ◆   s1KE2   
  ▶     ■    s1KE3   
  ∟    ◆   s1KE3   
  ▶     ■    s1KE4   
  ∟    ◆   s1KE4   
  ▶     ■    s1KE5   
  ∟    ◆   s1KE5   
  ▶     ■    s1KE6   
  ∟    ◆   s1KE6   
  ▶     ■    s1KO1   
  ∟    ◆   s1KO1   
  ▶     ■    s1KO2   
  ∟    ◆   s1KO2   
  ▶     ■    s1KO3   
  ∟    ◆   s1KO3   
  ▶     ■    s1KO4   
  ∟    ◆   s1KO4   
  ▶     ■    s1KO5   
  ∟    ◆   s1KO5   
  ▶     ■    s1WE1   
  ∟    ◆   s1WE1   
  ▶     ■    s1WE2   
  ∟    ◆   s1WE2   
  ▶     ■    s1WE3   
  ∟    ◆   s1WE3   
  ▶     ■    s1WE4   
  ∟    ◆   s1WE4   
  ▶     ■    s1WE5   
  ∟    ◆   s1WE5   
  ▶     ■    s1WE6   
  ∟    ◆   s1WE6   
  ▶     ■    s1WT1   
  ∟    ◆   s1WT1   
  ▶     ■    s1WT2   
  ∟    ◆   s1WT2   
  ▶     ■    s1WT3   
  ∟    ◆   s1WT3   
  ▶     ■    s1WT4   
  ∟    ◆   s1WT4   
  ▶     ■    s1WT5   
  ∟    ◆   s1WT5   
  ▶     ■    s1WT6   
  ∟    ◆   s1WT6   
  ▶     ■    s1WT7   
  ∟    ◆   s1WT7   
  ▶     ■    s2KE1   
  ∟    ◆   s2KE1   
  ▶     ■    s2KE2   
  ∟    ◆   s2KE2   
  ▶     ■    s2KE3   
  ∟    ◆   s2KE3   
  ▶     ■    s2KE4   
  ∟    ◆   s2KE4   
  ▶     ■    s2KE5   
  ∟    ◆   s2KE5   
  ▶     ■    s2KE6   
  ∟    ◆   s2KE6   
  ▶     ■    s2KO1   
  ∟    ◆   s2KO1   
  ▶     ■    s2KO2   
  ∟    ◆   s2KO2   
  ▶     ■    s2KO3   
  ∟    ◆   s2KO3   
  ▶     ■    s2KO4   
  ∟    ◆   s2KO4   
  ▶     ■    s2WE1   
  ∟    ◆   s2WE1   
  ▶     ■    s2WE2   
  ∟    ◆   s2WE2   
  ▶     ■    s2WE3   
  ∟    ◆   s2WE3   
  ▶     ■    s2WE4   
  ∟    ◆   s2WE4   
  ▶     ■    s2WE5   
  ∟    ◆   s2WE5   
  ▶     ■    s2WT1   
  ∟    ◆   s2WT1   
  ▶     ■    s2WT2   
  ∟    ◆   s2WT2   
  ▶     ■    s2WT3   
  ∟    ◆   s2WT3   
  ▶     ■    s2WT4   
  ∟    ◆   s2WT4   
  ▶     ■    s2WT5   
  ∟    ◆   s2WT5   
  ▶     ■    s2WT6   
  ∟    ◆   s2WT6   
  ▶     ■    s2WT7   
  ∟    ◆   s2WT7   
  ▶     ■    s3KE1   
  ∟    ◆   s3KE1   
  ▶     ■    s3KE2   
  ∟    ◆   s3KE2   
  ▶     ■    s3KE3   
  ∟    ◆   s3KE3   
  ▶     ■    s3KE4   
  ∟    ◆   s3KE4   
  ▶     ■    s3KE5   
  ∟    ◆   s3KE5   
  ▶     ■    s3KO1   
  ∟    ◆   s3KO1   
  ▶     ■    s3KO2   
  ∟    ◆   s3KO2   
  ▶     ■    s3KO3   
  ∟    ◆   s3KO3   
  ▶     ■    s3KO4   
  ∟    ◆   s3KO4   
  ▶     ■    s3KO5   
  ∟    ◆   s3KO5   
  ▶     ■    s3KO6   
  ∟    ◆   s3KO6   
  ▶     ■    s3WE1   
  ∟    ◆   s3WE1   
  ▶     ■    s3WE2   
  ∟    ◆   s3WE2   
  ▶     ■    s3WE3   
  ∟    ◆   s3WE3   
  ▶     ■    s3WE4   
  ∟    ◆   s3WE4   
  ▶     ■    s3WE5   
  ∟    ◆   s3WE5   
  ▶     ■    s3WE6   
  ∟    ◆   s3WE6   
  ▶     ■    s3WE7   
  ∟    ◆   s3WE7   
  ▶     ■    s3WT1   
  ∟    ◆   s3WT1   
  ▶     ■    s3WT2   
  ∟    ◆   s3WT2   
  ▶     ■    s3WT3   
  ∟    ◆   s3WT3   
  ▶     ■    s3WT4   
  ∟    ◆   s3WT4   
  ▶     ■    s3WT5   
  ∟    ◆   s3WT5   
  ▶     ■    s3WT6   
  ∟    ◆   s3WT6   
  ▶     ■    s3WT7   
  ∟    ◆   s3WT7   
  ▶     ■    1KE   
  ∟    ◆   s1KE1   
  ∟    ◆   s1KE2   
  ∟    ◆   s1KE3   
  ∟    ◆   s1KE4   
  ∟    ◆   s1KE5   
  ∟    ◆   s1KE6   
  ▶     ■    1KO   
  ∟    ◆   s1KO1   
  ∟    ◆   s1KO2   
  ∟    ◆   s1KO3   
  ∟    ◆   s1KO4   
  ∟    ◆   s1KO5   
  ▶     ■    1WE   
  ∟    ◆   s1WE1   
  ∟    ◆   s1WE2   
  ∟    ◆   s1WE3   
  ∟    ◆   s1WE4   
  ∟    ◆   s1WE5   
  ∟    ◆   s1WE6   
  ▶     ■    1WT   
  ∟    ◆   s1WT1   
  ∟    ◆   s1WT2   
  ∟    ◆   s1WT3   
  ∟    ◆   s1WT4   
  ∟    ◆   s1WT5   
  ∟    ◆   s1WT6   
  ∟    ◆   s1WT7   
  ▶     ■    2KE   
  ∟    ◆   s2KE1   
  ∟    ◆   s2KE2   
  ∟    ◆   s2KE3   
  ∟    ◆   s2KE4   
  ∟    ◆   s2KE5   
  ∟    ◆   s2KE6   
  ▶     ■    2KO   
  ∟    ◆   s2KO1   
  ∟    ◆   s2KO2   
  ∟    ◆   s2KO3   
  ∟    ◆   s2KO4   
  ▶     ■    2WE   
  ∟    ◆   s2WE1   
  ∟    ◆   s2WE2   
  ∟    ◆   s2WE3   
  ∟    ◆   s2WE4   
  ∟    ◆   s2WE5   
  ▶     ■    2WT   
  ∟    ◆   s2WT1   
  ∟    ◆   s2WT2   
  ∟    ◆   s2WT3   
  ∟    ◆   s2WT4   
  ∟    ◆   s2WT5   
  ∟    ◆   s2WT6   
  ∟    ◆   s2WT7   
  ▶     ■    3KE   
  ∟    ◆   s3KE1   
  ∟    ◆   s3KE2   
  ∟    ◆   s3KE3   
  ∟    ◆   s3KE4   
  ∟    ◆   s3KE5   
  ▶     ■    3KO   
  ∟    ◆   s3KO1   
  ∟    ◆   s3KO2   
  ∟    ◆   s3KO3   
  ∟    ◆   s3KO4   
  ∟    ◆   s3KO5   
  ∟    ◆   s3KO6   
  ▶     ■    3WE   
  ∟    ◆   s3WE1   
  ∟    ◆   s3WE2   
  ∟    ◆   s3WE3   
  ∟    ◆   s3WE4   
  ∟    ◆   s3WE5   
  ∟    ◆   s3WE6   
  ∟    ◆   s3WE7   
  ▶     ■    3WT   
  ∟    ◆   s3WT1   
  ∟    ◆   s3WT2   
  ∟    ◆   s3WT3   
  ∟    ◆   s3WT4   
  ∟    ◆   s3WT5   
  ∟    ◆   s3WT6   
  ∟    ◆   s3WT7   
  ▶     ■    AAAAAA   
  ∟    ◆   s1KE1   
  ∟    ◆   s1KE2   
  ∟    ◆   s1KE3   
  ∟    ◆   s1KE4   
  ∟    ◆   s1KE5   
  ∟    ◆   s1KE6   
  ∟    ◆   s1KO1   
  ∟    ◆   s1KO2   
  ∟    ◆   s1KO3   
  ∟    ◆   s1KO4   
  ∟    ◆   s1KO5   
  ∟    ◆   s1WE1   
  ∟    ◆   s1WE2   
  ∟    ◆   s1WE3   
  ∟    ◆   s1WE4   
  ∟    ◆   s1WE5   
  ∟    ◆   s1WE6   
  ∟    ◆   s1WT1   
  ∟    ◆   s1WT2   
  ∟    ◆   s1WT3   
  ∟    ◆   s1WT4   
  ∟    ◆   s1WT5   
  ∟    ◆   s1WT6   
  ∟    ◆   s1WT7   
  ∟    ◆   s2KE1   
  ∟    ◆   s2KE2   
  ∟    ◆   s2KE3   
  ∟    ◆   s2KE4   
  ∟    ◆   s2KE5   
  ∟    ◆   s2KE6   
  ∟    ◆   s2KO1   
  ∟    ◆   s2KO2   
  ∟    ◆   s2KO3   
  ∟    ◆   s2KO4   
  ∟    ◆   s2WE1   
  ∟    ◆   s2WE2   
  ∟    ◆   s2WE3   
  ∟    ◆   s2WE4   
  ∟    ◆   s2WE5   
  ∟    ◆   s2WT1   
  ∟    ◆   s2WT2   
  ∟    ◆   s2WT3   
  ∟    ◆   s2WT4   
  ∟    ◆   s2WT5   
  ∟    ◆   s2WT6   
  ∟    ◆   s2WT7   
  ∟    ◆   s3KE1   
  ∟    ◆   s3KE2   
  ∟    ◆   s3KE3   
  ∟    ◆   s3KE4   
  ∟    ◆   s3KE5   
  ∟    ◆   s3KO1   
  ∟    ◆   s3KO2   
  ∟    ◆   s3KO3   
  ∟    ◆   s3KO4   
  ∟    ◆   s3KO5   
  ∟    ◆   s3KO6   
  ∟    ◆   s3WE1   
  ∟    ◆   s3WE2   
  ∟    ◆   s3WE3   
  ∟    ◆   s3WE4   
  ∟    ◆   s3WE5   
  ∟    ◆   s3WE6   
  ∟    ◆   s3WE7   
  ∟    ◆   s3WT1   
  ∟    ◆   s3WT2   
  ∟    ◆   s3WT3   
  ∟    ◆   s3WT4   
  ∟    ◆   s3WT5   
  ∟    ◆   s3WT6   
  ∟    ◆   s3WT7   
  ▶     ■    s1KE1   
  ∟    ◆   s1KE1   
  ▶     ■    s1KE2   
  ∟    ◆   s1KE2   
  ▶     ■    s1KE3   
  ∟    ◆   s1KE3   
  ▶     ■    s1KE4   
  ∟    ◆   s1KE4   
  ▶     ■    s1KE5   
  ∟    ◆   s1KE5   
  ▶     ■    s1KE6   
  ∟    ◆   s1KE6   
  ▶     ■    s1KO1   
  ∟    ◆   s1KO1   
  ▶     ■    s1KO2   
  ∟    ◆   s1KO2   
  ▶     ■    s1KO3   
  ∟    ◆   s1KO3   
  ▶     ■    s1KO4   
  ∟    ◆   s1KO4   
  ▶     ■    s1KO5   
  ∟    ◆   s1KO5   
  ▶     ■    s1WE1   
  ∟    ◆   s1WE1   
  ▶     ■    s1WE2   
  ∟    ◆   s1WE2   
  ▶     ■    s1WE3   
  ∟    ◆   s1WE3   
  ▶     ■    s1WE4   
  ∟    ◆   s1WE4   
  ▶     ■    s1WE5   
  ∟    ◆   s1WE5   
  ▶     ■    s1WE6   
  ∟    ◆   s1WE6   
  ▶     ■    s1WT1   
  ∟    ◆   s1WT1   
  ▶     ■    s1WT2   
  ∟    ◆   s1WT2   
  ▶     ■    s1WT3   
  ∟    ◆   s1WT3   
  ▶     ■    s1WT4   
  ∟    ◆   s1WT4   
  ▶     ■    s1WT5   
  ∟    ◆   s1WT5   
  ▶     ■    s1WT6   
  ∟    ◆   s1WT6   
  ▶     ■    s1WT7   
  ∟    ◆   s1WT7   
  ▶     ■    s2KE1   
  ∟    ◆   s2KE1   
  ▶     ■    s2KE2   
  ∟    ◆   s2KE2   
  ▶     ■    s2KE3   
  ∟    ◆   s2KE3   
  ▶     ■    s2KE4   
  ∟    ◆   s2KE4   
  ▶     ■    s2KE5   
  ∟    ◆   s2KE5   
  ▶     ■    s2KE6   
  ∟    ◆   s2KE6   
  ▶     ■    s2KO1   
  ∟    ◆   s2KO1   
  ▶     ■    s2KO2   
  ∟    ◆   s2KO2   
  ▶     ■    s2KO3   
  ∟    ◆   s2KO3   
  ▶     ■    s2KO4   
  ∟    ◆   s2KO4   
  ▶     ■    s2WE1   
  ∟    ◆   s2WE1   
  ▶     ■    s2WE2   
  ∟    ◆   s2WE2   
  ▶     ■    s2WE3   
  ∟    ◆   s2WE3   
  ▶     ■    s2WE4   
  ∟    ◆   s2WE4   
  ▶     ■    s2WE5   
  ∟    ◆   s2WE5   
  ▶     ■    s2WT1   
  ∟    ◆   s2WT1   
  ▶     ■    s2WT2   
  ∟    ◆   s2WT2   
  ▶     ■    s2WT3   
  ∟    ◆   s2WT3   
  ▶     ■    s2WT4   
  ∟    ◆   s2WT4   
  ▶     ■    s2WT5   
  ∟    ◆   s2WT5   
  ▶     ■    s2WT6   
  ∟    ◆   s2WT6   
  ▶     ■    s2WT7   
  ∟    ◆   s2WT7   
  ▶     ■    s3KE1   
  ∟    ◆   s3KE1   
  ▶     ■    s3KE2   
  ∟    ◆   s3KE2   
  ▶     ■    s3KE3   
  ∟    ◆   s3KE3   
  ▶     ■    s3KE4   
  ∟    ◆   s3KE4   
  ▶     ■    s3KE5   
  ∟    ◆   s3KE5   
  ▶     ■    s3KO1   
  ∟    ◆   s3KO1   
  ▶     ■    s3KO2   
  ∟    ◆   s3KO2   
  ▶     ■    s3KO3   
  ∟    ◆   s3KO3   
  ▶     ■    s3KO4   
  ∟    ◆   s3KO4   
  ▶     ■    s3KO5   
  ∟    ◆   s3KO5   
  ▶     ■    s3KO6   
  ∟    ◆   s3KO6   
  ▶     ■    s3WE1   
  ∟    ◆   s3WE1   
  ▶     ■    s3WE2   
  ∟    ◆   s3WE2   
  ▶     ■    s3WE3   
  ∟    ◆   s3WE3   
  ▶     ■    s3WE4   
  ∟    ◆   s3WE4   
  ▶     ■    s3WE5   
  ∟    ◆   s3WE5   
  ▶     ■    s3WE6   
  ∟    ◆   s3WE6   
  ▶     ■    s3WE7   
  ∟    ◆   s3WE7   
  ▶     ■    s3WT1   
  ∟    ◆   s3WT1   
  ▶     ■    s3WT2   
  ∟    ◆   s3WT2   
  ▶     ■    s3WT3   
  ∟    ◆   s3WT3   
  ▶     ■    s3WT4   
  ∟    ◆   s3WT4   
  ▶     ■    s3WT5   
  ∟    ◆   s3WT5   
  ▶     ■    s3WT6   
  ∟    ◆   s3WT6   
  ▶     ■    s3WT7   
  ∟    ◆   s3WT7   
  ▶     ■    ATCGA   
  ∟    ◆   s1KE1   
  ∟    ◆   s1KE2   
  ∟    ◆   s1KE3   
  ∟    ◆   s1KE4   
  ∟    ◆   s1KE5   
  ∟    ◆   s1KE6   
  ∟    ◆   s1KO1   
  ∟    ◆   s1KO2   
  ∟    ◆   s1KO3   
  ∟    ◆   s1KO4   
  ∟    ◆   s1KO5   
  ∟    ◆   s1WE1   
  ∟    ◆   s1WE2   
  ∟    ◆   s1WE3   
  ∟    ◆   s1WE4   
  ∟    ◆   s1WE5   
  ∟    ◆   s1WE6   
  ∟    ◆   s1WT1   
  ∟    ◆   s1WT2   
  ∟    ◆   s1WT3   
  ∟    ◆   s1WT4   
  ∟    ◆   s1WT5   
  ∟    ◆   s1WT6   
  ∟    ◆   s1WT7   
  ∟    ◆   s2KE1   
  ∟    ◆   s2KE2   
  ∟    ◆   s2KE3   
  ∟    ◆   s2KE4   
  ∟    ◆   s2KE5   
  ∟    ◆   s2KE6   
  ∟    ◆   s2KO1   
  ∟    ◆   s2KO2   
  ∟    ◆   s2KO3   
  ∟    ◆   s2KO4   
  ∟    ◆   s2WE1   
  ∟    ◆   s2WE2   
  ∟    ◆   s2WE3   
  ∟    ◆   s2WE4   
  ∟    ◆   s2WE5   
  ∟    ◆   s2WT1   
  ∟    ◆   s2WT2   
  ∟    ◆   s2WT3   
  ∟    ◆   s2WT4   
  ∟    ◆   s2WT5   
  ∟    ◆   s2WT6   
  ∟    ◆   s2WT7   
  ∟    ◆   s3KE1   
  ∟    ◆   s3KE2   
  ∟    ◆   s3KE3   
  ∟    ◆   s3KE4   
  ∟    ◆   s3KE5   
  ∟    ◆   s3KO1   
  ∟    ◆   s3KO2   
  ∟    ◆   s3KO3   
  ∟    ◆   s3KO4   
  ∟    ◆   s3KO5   
  ∟    ◆   s3KO6   
  ∟    ◆   s3WE1   
  ∟    ◆   s3WE2   
  ∟    ◆   s3WE3   
  ∟    ◆   s3WE4   
  ∟    ◆   s3WE5   
  ∟    ◆   s3WE6   
  ∟    ◆   s3WE7   
  ∟    ◆   s3WT1   
  ∟    ◆   s3WT2   
  ∟    ◆   s3WT3   
  ∟    ◆   s3WT4   
  ∟    ◆   s3WT5   
  ∟    ◆   s3WT6   
  ∟    ◆   s3WT7   
  ▶     ■    s1KE1   
  ∟    ◆   s1KE1   
  ▶     ■    s1KE2   
  ∟    ◆   s1KE2   
  ▶     ■    s1KE3   
  ∟    ◆   s1KE3   
  ▶     ■    s1KE4   
  ∟    ◆   s1KE4   
  ▶     ■    s1KE5   
  ∟    ◆   s1KE5   
  ▶     ■    s1KE6   
  ∟    ◆   s1KE6   
  ▶     ■    s1KO1   
  ∟    ◆   s1KO1   
  ▶     ■    s1KO2   
  ∟    ◆   s1KO2   
  ▶     ■    s1KO3   
  ∟    ◆   s1KO3   
  ▶     ■    s1KO4   
  ∟    ◆   s1KO4   
  ▶     ■    s1KO5   
  ∟    ◆   s1KO5   
  ▶     ■    s1WE1   
  ∟    ◆   s1WE1   
  ▶     ■    s1WE2   
  ∟    ◆   s1WE2   
  ▶     ■    s1WE3   
  ∟    ◆   s1WE3   
  ▶     ■    s1WE4   
  ∟    ◆   s1WE4   
  ▶     ■    s1WE5   
  ∟    ◆   s1WE5   
  ▶     ■    s1WE6   
  ∟    ◆   s1WE6   
  ▶     ■    s1WT1   
  ∟    ◆   s1WT1   
  ▶     ■    s1WT2   
  ∟    ◆   s1WT2   
  ▶     ■    s1WT3   
  ∟    ◆   s1WT3   
  ▶     ■    s1WT4   
  ∟    ◆   s1WT4   
  ▶     ■    s1WT5   
  ∟    ◆   s1WT5   
  ▶     ■    s1WT6   
  ∟    ◆   s1WT6   
  ▶     ■    s1WT7   
  ∟    ◆   s1WT7   
  ▶     ■    s2KE1   
  ∟    ◆   s2KE1   
  ▶     ■    s2KE2   
  ∟    ◆   s2KE2   
  ▶     ■    s2KE3   
  ∟    ◆   s2KE3   
  ▶     ■    s2KE4   
  ∟    ◆   s2KE4   
  ▶     ■    s2KE5   
  ∟    ◆   s2KE5   
  ▶     ■    s2KE6   
  ∟    ◆   s2KE6   
  ▶     ■    s2KO1   
  ∟    ◆   s2KO1   
  ▶     ■    s2KO2   
  ∟    ◆   s2KO2   
  ▶     ■    s2KO3   
  ∟    ◆   s2KO3   
  ▶     ■    s2KO4   
  ∟    ◆   s2KO4   
  ▶     ■    s2WE1   
  ∟    ◆   s2WE1   
  ▶     ■    s2WE2   
  ∟    ◆   s2WE2   
  ▶     ■    s2WE3   
  ∟    ◆   s2WE3   
  ▶     ■    s2WE4   
  ∟    ◆   s2WE4   
  ▶     ■    s2WE5   
  ∟    ◆   s2WE5   
  ▶     ■    s2WT1   
  ∟    ◆   s2WT1   
  ▶     ■    s2WT2   
  ∟    ◆   s2WT2   
  ▶     ■    s2WT3   
  ∟    ◆   s2WT3   
  ▶     ■    s2WT4   
  ∟    ◆   s2WT4   
  ▶     ■    s2WT5   
  ∟    ◆   s2WT5   
  ▶     ■    s2WT6   
  ∟    ◆   s2WT6   
  ▶     ■    s2WT7   
  ∟    ◆   s2WT7   
  ▶     ■    s3KE1   
  ∟    ◆   s3KE1   
  ▶     ■    s3KE2   
  ∟    ◆   s3KE2   
  ▶     ■    s3KE3   
  ∟    ◆   s3KE3   
  ▶     ■    s3KE4   
  ∟    ◆   s3KE4   
  ▶     ■    s3KE5   
  ∟    ◆   s3KE5   
  ▶     ■    s3KO1   
  ∟    ◆   s3KO1   
  ▶     ■    s3KO2   
  ∟    ◆   s3KO2   
  ▶     ■    s3KO3   
  ∟    ◆   s3KO3   
  ▶     ■    s3KO4   
  ∟    ◆   s3KO4   
  ▶     ■    s3KO5   
  ∟    ◆   s3KO5   
  ▶     ■    s3KO6   
  ∟    ◆   s3KO6   
  ▶     ■    s3WE1   
  ∟    ◆   s3WE1   
  ▶     ■    s3WE2   
  ∟    ◆   s3WE2   
  ▶     ■    s3WE3   
  ∟    ◆   s3WE3   
  ▶     ■    s3WE4   
  ∟    ◆   s3WE4   
  ▶     ■    s3WE5   
  ∟    ◆   s3WE5   
  ▶     ■    s3WE6   
  ∟    ◆   s3WE6   
  ▶     ■    s3WE7   
  ∟    ◆   s3WE7   
  ▶     ■    s3WT1   
  ∟    ◆   s3WT1   
  ▶     ■    s3WT2   
  ∟    ◆   s3WT2   
  ▶     ■    s3WT3   
  ∟    ◆   s3WT3   
  ▶     ■    s3WT4   
  ∟    ◆   s3WT4   
  ▶     ■    s3WT5   
  ∟    ◆   s3WT5   
  ▶     ■    s3WT6   
  ∟    ◆   s3WT6   
  ▶     ■    s3WT7   
  ∟    ◆   s3WT7   
  ▶     ■    1KE   
  ∟    ◆   s1KE1   
  ∟    ◆   s1KE2   
  ∟    ◆   s1KE3   
  ∟    ◆   s1KE4   
  ∟    ◆   s1KE5   
  ∟    ◆   s1KE6   
  ▶     ■    1KO   
  ∟    ◆   s1KO1   
  ∟    ◆   s1KO2   
  ∟    ◆   s1KO3   
  ∟    ◆   s1KO4   
  ∟    ◆   s1KO5   
  ▶     ■    1WE   
  ∟    ◆   s1WE1   
  ∟    ◆   s1WE2   
  ∟    ◆   s1WE3   
  ∟    ◆   s1WE4   
  ∟    ◆   s1WE5   
  ∟    ◆   s1WE6   
  ▶     ■    1WT   
  ∟    ◆   s1WT1   
  ∟    ◆   s1WT2   
  ∟    ◆   s1WT3   
  ∟    ◆   s1WT4   
  ∟    ◆   s1WT5   
  ∟    ◆   s1WT6   
  ∟    ◆   s1WT7   
  ▶     ■    2KE   
  ∟    ◆   s2KE1   
  ∟    ◆   s2KE2   
  ∟    ◆   s2KE3   
  ∟    ◆   s2KE4   
  ∟    ◆   s2KE5   
  ∟    ◆   s2KE6   
  ▶     ■    2KO   
  ∟    ◆   s2KO1   
  ∟    ◆   s2KO2   
  ∟    ◆   s2KO3   
  ∟    ◆   s2KO4   
  ▶     ■    2WE   
  ∟    ◆   s2WE1   
  ∟    ◆   s2WE2   
  ∟    ◆   s2WE3   
  ∟    ◆   s2WE4   
  ∟    ◆   s2WE5   
  ▶     ■    2WT   
  ∟    ◆   s2WT1   
  ∟    ◆   s2WT2   
  ∟    ◆   s2WT3   
  ∟    ◆   s2WT4   
  ∟    ◆   s2WT5   
  ∟    ◆   s2WT6   
  ∟    ◆   s2WT7   
  ▶     ■    3KE   
  ∟    ◆   s3KE1   
  ∟    ◆   s3KE2   
  ∟    ◆   s3KE3   
  ∟    ◆   s3KE4   
  ∟    ◆   s3KE5   
  ▶     ■    3KO   
  ∟    ◆   s3KO1   
  ∟    ◆   s3KO2   
  ∟    ◆   s3KO3   
  ∟    ◆   s3KO4   
  ∟    ◆   s3KO5   
  ∟    ◆   s3KO6   
  ▶     ■    3WE   
  ∟    ◆   s3WE1   
  ∟    ◆   s3WE2   
  ∟    ◆   s3WE3   
  ∟    ◆   s3WE4   
  ∟    ◆   s3WE5   
  ∟    ◆   s3WE6   
  ∟    ◆   s3WE7   
  ▶     ■    3WT   
  ∟    ◆   s3WT1   
  ∟    ◆   s3WT2   
  ∟    ◆   s3WT3   
  ∟    ◆   s3WT4   
  ∟    ◆   s3WT5   
  ∟    ◆   s3WT6   
  ∟    ◆   s3WT7   
  ▶     ■    AAAAAA   
  ∟    ◆   s1KE1   
  ∟    ◆   s1KE2   
  ∟    ◆   s1KE3   
  ∟    ◆   s1KE4   
  ∟    ◆   s1KE5   
  ∟    ◆   s1KE6   
  ∟    ◆   s1KO1   
  ∟    ◆   s1KO2   
  ∟    ◆   s1KO3   
  ∟    ◆   s1KO4   
  ∟    ◆   s1KO5   
  ∟    ◆   s1WE1   
  ∟    ◆   s1WE2   
  ∟    ◆   s1WE3   
  ∟    ◆   s1WE4   
  ∟    ◆   s1WE5   
  ∟    ◆   s1WE6   
  ∟    ◆   s1WT1   
  ∟    ◆   s1WT2   
  ∟    ◆   s1WT3   
  ∟    ◆   s1WT4   
  ∟    ◆   s1WT5   
  ∟    ◆   s1WT6   
  ∟    ◆   s1WT7   
  ∟    ◆   s2KE1   
  ∟    ◆   s2KE2   
  ∟    ◆   s2KE3   
  ∟    ◆   s2KE4   
  ∟    ◆   s2KE5   
  ∟    ◆   s2KE6   
  ∟    ◆   s2KO1   
  ∟    ◆   s2KO2   
  ∟    ◆   s2KO3   
  ∟    ◆   s2KO4   
  ∟    ◆   s2WE1   
  ∟    ◆   s2WE2   
  ∟    ◆   s2WE3   
  ∟    ◆   s2WE4   
  ∟    ◆   s2WE5   
  ∟    ◆   s2WT1   
  ∟    ◆   s2WT2   
  ∟    ◆   s2WT3   
  ∟    ◆   s2WT4   
  ∟    ◆   s2WT5   
  ∟    ◆   s2WT6   
  ∟    ◆   s2WT7   
  ∟    ◆   s3KE1   
  ∟    ◆   s3KE2   
  ∟    ◆   s3KE3   
  ∟    ◆   s3KE4   
  ∟    ◆   s3KE5   
  ∟    ◆   s3KO1   
  ∟    ◆   s3KO2   
  ∟    ◆   s3KO3   
  ∟    ◆   s3KO4   
  ∟    ◆   s3KO5   
  ∟    ◆   s3KO6   
  ∟    ◆   s3WE1   
  ∟    ◆   s3WE2   
  ∟    ◆   s3WE3   
  ∟    ◆   s3WE4   
  ∟    ◆   s3WE5   
  ∟    ◆   s3WE6   
  ∟    ◆   s3WE7   
  ∟    ◆   s3WT1   
  ∟    ◆   s3WT2   
  ∟    ◆   s3WT3   
  ∟    ◆   s3WT4   
  ∟    ◆   s3WT5   
  ∟    ◆   s3WT6   
  ∟    ◆   s3WT7   
  ▶     ■    s1KE1   
  ∟    ◆   s1KE1   
  ▶     ■    s1KE2   
  ∟    ◆   s1KE2   
  ▶     ■    s1KE3   
  ∟    ◆   s1KE3   
  ▶     ■    s1KE4   
  ∟    ◆   s1KE4   
  ▶     ■    s1KE5   
  ∟    ◆   s1KE5   
  ▶     ■    s1KE6   
  ∟    ◆   s1KE6   
  ▶     ■    s1KO1   
  ∟    ◆   s1KO1   
  ▶     ■    s1KO2   
  ∟    ◆   s1KO2   
  ▶     ■    s1KO3   
  ∟    ◆   s1KO3   
  ▶     ■    s1KO4   
  ∟    ◆   s1KO4   
  ▶     ■    s1KO5   
  ∟    ◆   s1KO5   
  ▶     ■    s1WE1   
  ∟    ◆   s1WE1   
  ▶     ■    s1WE2   
  ∟    ◆   s1WE2   
  ▶     ■    s1WE3   
  ∟    ◆   s1WE3   
  ▶     ■    s1WE4   
  ∟    ◆   s1WE4   
  ▶     ■    s1WE5   
  ∟    ◆   s1WE5   
  ▶     ■    s1WE6   
  ∟    ◆   s1WE6   
  ▶     ■    s1WT1   
  ∟    ◆   s1WT1   
  ▶     ■    s1WT2   
  ∟    ◆   s1WT2   
  ▶     ■    s1WT3   
  ∟    ◆   s1WT3   
  ▶     ■    s1WT4   
  ∟    ◆   s1WT4   
  ▶     ■    s1WT5   
  ∟    ◆   s1WT5   
  ▶     ■    s1WT6   
  ∟    ◆   s1WT6   
  ▶     ■    s1WT7   
  ∟    ◆   s1WT7   
  ▶     ■    s2KE1   
  ∟    ◆   s2KE1   
  ▶     ■    s2KE2   
  ∟    ◆   s2KE2   
  ▶     ■    s2KE3   
  ∟    ◆   s2KE3   
  ▶     ■    s2KE4   
  ∟    ◆   s2KE4   
  ▶     ■    s2KE5   
  ∟    ◆   s2KE5   
  ▶     ■    s2KE6   
  ∟    ◆   s2KE6   
  ▶     ■    s2KO1   
  ∟    ◆   s2KO1   
  ▶     ■    s2KO2   
  ∟    ◆   s2KO2   
  ▶     ■    s2KO3   
  ∟    ◆   s2KO3   
  ▶     ■    s2KO4   
  ∟    ◆   s2KO4   
  ▶     ■    s2WE1   
  ∟    ◆   s2WE1   
  ▶     ■    s2WE2   
  ∟    ◆   s2WE2   
  ▶     ■    s2WE3   
  ∟    ◆   s2WE3   
  ▶     ■    s2WE4   
  ∟    ◆   s2WE4   
  ▶     ■    s2WE5   
  ∟    ◆   s2WE5   
  ▶     ■    s2WT1   
  ∟    ◆   s2WT1   
  ▶     ■    s2WT2   
  ∟    ◆   s2WT2   
  ▶     ■    s2WT3   
  ∟    ◆   s2WT3   
  ▶     ■    s2WT4   
  ∟    ◆   s2WT4   
  ▶     ■    s2WT5   
  ∟    ◆   s2WT5   
  ▶     ■    s2WT6   
  ∟    ◆   s2WT6   
  ▶     ■    s2WT7   
  ∟    ◆   s2WT7   
  ▶     ■    s3KE1   
  ∟    ◆   s3KE1   
  ▶     ■    s3KE2   
  ∟    ◆   s3KE2   
  ▶     ■    s3KE3   
  ∟    ◆   s3KE3   
  ▶     ■    s3KE4   
  ∟    ◆   s3KE4   
  ▶     ■    s3KE5   
  ∟    ◆   s3KE5   
  ▶     ■    s3KO1   
  ∟    ◆   s3KO1   
  ▶     ■    s3KO2   
  ∟    ◆   s3KO2   
  ▶     ■    s3KO3   
  ∟    ◆   s3KO3   
  ▶     ■    s3KO4   
  ∟    ◆   s3KO4   
  ▶     ■    s3KO5   
  ∟    ◆   s3KO5   
  ▶     ■    s3KO6   
  ∟    ◆   s3KO6   
  ▶     ■    s3WE1   
  ∟    ◆   s3WE1   
  ▶     ■    s3WE2   
  ∟    ◆   s3WE2   
  ▶     ■    s3WE3   
  ∟    ◆   s3WE3   
  ▶     ■    s3WE4   
  ∟    ◆   s3WE4   
  ▶     ■    s3WE5   
  ∟    ◆   s3WE5   
  ▶     ■    s3WE6   
  ∟    ◆   s3WE6   
  ▶     ■    s3WE7   
  ∟    ◆   s3WE7   
  ▶     ■    s3WT1   
  ∟    ◆   s3WT1   
  ▶     ■    s3WT2   
  ∟    ◆   s3WT2   
  ▶     ■    s3WT3   
  ∟    ◆   s3WT3   
  ▶     ■    s3WT4   
  ∟    ◆   s3WT4   
  ▶     ■    s3WT5   
  ∟    ◆   s3WT5   
  ▶     ■    s3WT6   
  ∟    ◆   s3WT6   
  ▶     ■    s3WT7   
  ∟    ◆   s3WT7   
  ▶     ■    ATCGA   
  ∟    ◆   s1KE1   
  ∟    ◆   s1KE2   
  ∟    ◆   s1KE3   
  ∟    ◆   s1KE4   
  ∟    ◆   s1KE5   
  ∟    ◆   s1KE6   
  ∟    ◆   s1KO1   
  ∟    ◆   s1KO2   
  ∟    ◆   s1KO3   
  ∟    ◆   s1KO4   
  ∟    ◆   s1KO5   
  ∟    ◆   s1WE1   
  ∟    ◆   s1WE2   
  ∟    ◆   s1WE3   
  ∟    ◆   s1WE4   
  ∟    ◆   s1WE5   
  ∟    ◆   s1WE6   
  ∟    ◆   s1WT1   
  ∟    ◆   s1WT2   
  ∟    ◆   s1WT3   
  ∟    ◆   s1WT4   
  ∟    ◆   s1WT5   
  ∟    ◆   s1WT6   
  ∟    ◆   s1WT7   
  ∟    ◆   s2KE1   
  ∟    ◆   s2KE2   
  ∟    ◆   s2KE3   
  ∟    ◆   s2KE4   
  ∟    ◆   s2KE5   
  ∟    ◆   s2KE6   
  ∟    ◆   s2KO1   
  ∟    ◆   s2KO2   
  ∟    ◆   s2KO3   
  ∟    ◆   s2KO4   
  ∟    ◆   s2WE1   
  ∟    ◆   s2WE2   
  ∟    ◆   s2WE3   
  ∟    ◆   s2WE4   
  ∟    ◆   s2WE5   
  ∟    ◆   s2WT1   
  ∟    ◆   s2WT2   
  ∟    ◆   s2WT3   
  ∟    ◆   s2WT4   
  ∟    ◆   s2WT5   
  ∟    ◆   s2WT6   
  ∟    ◆   s2WT7   
  ∟    ◆   s3KE1   
  ∟    ◆   s3KE2   
  ∟    ◆   s3KE3   
  ∟    ◆   s3KE4   
  ∟    ◆   s3KE5   
  ∟    ◆   s3KO1   
  ∟    ◆   s3KO2   
  ∟    ◆   s3KO3   
  ∟    ◆   s3KO4   
  ∟    ◆   s3KO5   
  ∟    ◆   s3KO6   
  ∟    ◆   s3WE1   
  ∟    ◆   s3WE2   
  ∟    ◆   s3WE3   
  ∟    ◆   s3WE4   
  ∟    ◆   s3WE5   
  ∟    ◆   s3WE6   
  ∟    ◆   s3WE7   
  ∟    ◆   s3WT1   
  ∟    ◆   s3WT2   
  ∟    ◆   s3WT3   
  ∟    ◆   s3WT4   
  ∟    ◆   s3WT5   
  ∟    ◆   s3WT6   
  ∟    ◆   s3WT7   
  ▶     ■    s1KE1   
  ∟    ◆   s1KE1   
  ▶     ■    s1KE2   
  ∟    ◆   s1KE2   
  ▶     ■    s1KE3   
  ∟    ◆   s1KE3   
  ▶     ■    s1KE4   
  ∟    ◆   s1KE4   
  ▶     ■    s1KE5   
  ∟    ◆   s1KE5   
  ▶     ■    s1KE6   
  ∟    ◆   s1KE6   
  ▶     ■    s1KO1   
  ∟    ◆   s1KO1   
  ▶     ■    s1KO2   
  ∟    ◆   s1KO2   
  ▶     ■    s1KO3   
  ∟    ◆   s1KO3   
  ▶     ■    s1KO4   
  ∟    ◆   s1KO4   
  ▶     ■    s1KO5   
  ∟    ◆   s1KO5   
  ▶     ■    s1WE1   
  ∟    ◆   s1WE1   
  ▶     ■    s1WE2   
  ∟    ◆   s1WE2   
  ▶     ■    s1WE3   
  ∟    ◆   s1WE3   
  ▶     ■    s1WE4   
  ∟    ◆   s1WE4   
  ▶     ■    s1WE5   
  ∟    ◆   s1WE5   
  ▶     ■    s1WE6   
  ∟    ◆   s1WE6   
  ▶     ■    s1WT1   
  ∟    ◆   s1WT1   
  ▶     ■    s1WT2   
  ∟    ◆   s1WT2   
  ▶     ■    s1WT3   
  ∟    ◆   s1WT3   
  ▶     ■    s1WT4   
  ∟    ◆   s1WT4   
  ▶     ■    s1WT5   
  ∟    ◆   s1WT5   
  ▶     ■    s1WT6   
  ∟    ◆   s1WT6   
  ▶     ■    s1WT7   
  ∟    ◆   s1WT7   
  ▶     ■    s2KE1   
  ∟    ◆   s2KE1   
  ▶     ■    s2KE2   
  ∟    ◆   s2KE2   
  ▶     ■    s2KE3   
  ∟    ◆   s2KE3   
  ▶     ■    s2KE4   
  ∟    ◆   s2KE4   
  ▶     ■    s2KE5   
  ∟    ◆   s2KE5   
  ▶     ■    s2KE6   
  ∟    ◆   s2KE6   
  ▶     ■    s2KO1   
  ∟    ◆   s2KO1   
  ▶     ■    s2KO2   
  ∟    ◆   s2KO2   
  ▶     ■    s2KO3   
  ∟    ◆   s2KO3   
  ▶     ■    s2KO4   
  ∟    ◆   s2KO4   
  ▶     ■    s2WE1   
  ∟    ◆   s2WE1   
  ▶     ■    s2WE2   
  ∟    ◆   s2WE2   
  ▶     ■    s2WE3   
  ∟    ◆   s2WE3   
  ▶     ■    s2WE4   
  ∟    ◆   s2WE4   
  ▶     ■    s2WE5   
  ∟    ◆   s2WE5   
  ▶     ■    s2WT1   
  ∟    ◆   s2WT1   
  ▶     ■    s2WT2   
  ∟    ◆   s2WT2   
  ▶     ■    s2WT3   
  ∟    ◆   s2WT3   
  ▶     ■    s2WT4   
  ∟    ◆   s2WT4   
  ▶     ■    s2WT5   
  ∟    ◆   s2WT5   
  ▶     ■    s2WT6   
  ∟    ◆   s2WT6   
  ▶     ■    s2WT7   
  ∟    ◆   s2WT7   
  ▶     ■    s3KE1   
  ∟    ◆   s3KE1   
  ▶     ■    s3KE2   
  ∟    ◆   s3KE2   
  ▶     ■    s3KE3   
  ∟    ◆   s3KE3   
  ▶     ■    s3KE4   
  ∟    ◆   s3KE4   
  ▶     ■    s3KE5   
  ∟    ◆   s3KE5   
  ▶     ■    s3KO1   
  ∟    ◆   s3KO1   
  ▶     ■    s3KO2   
  ∟    ◆   s3KO2   
  ▶     ■    s3KO3   
  ∟    ◆   s3KO3   
  ▶     ■    s3KO4   
  ∟    ◆   s3KO4   
  ▶     ■    s3KO5   
  ∟    ◆   s3KO5   
  ▶     ■    s3KO6   
  ∟    ◆   s3KO6   
  ▶     ■    s3WE1   
  ∟    ◆   s3WE1   
  ▶     ■    s3WE2   
  ∟    ◆   s3WE2   
  ▶     ■    s3WE3   
  ∟    ◆   s3WE3   
  ▶     ■    s3WE4   
  ∟    ◆   s3WE4   
  ▶     ■    s3WE5   
  ∟    ◆   s3WE5   
  ▶     ■    s3WE6   
  ∟    ◆   s3WE6   
  ▶     ■    s3WE7   
  ∟    ◆   s3WE7   
  ▶     ■    s3WT1   
  ∟    ◆   s3WT1   
  ▶     ■    s3WT2   
  ∟    ◆   s3WT2   
  ▶     ■    s3WT3   
  ∟    ◆   s3WT3   
  ▶     ■    s3WT4   
  ∟    ◆   s3WT4   
  ▶     ■    s3WT5   
  ∟    ◆   s3WT5   
  ▶     ■    s3WT6   
  ∟    ◆   s3WT6   
  ▶     ■    s3WT7   
  ∟    ◆   s3WT7   
  ▶     ■    1KE   
  ∟    ◆   s1KE1   
  ∟    ◆   s1KE2   
  ∟    ◆   s1KE3   
  ∟    ◆   s1KE4   
  ∟    ◆   s1KE5   
  ∟    ◆   s1KE6   
  ▶     ■    1KO   
  ∟    ◆   s1KO1   
  ∟    ◆   s1KO2   
  ∟    ◆   s1KO3   
  ∟    ◆   s1KO4   
  ∟    ◆   s1KO5   
  ▶     ■    1WE   
  ∟    ◆   s1WE1   
  ∟    ◆   s1WE2   
  ∟    ◆   s1WE3   
  ∟    ◆   s1WE4   
  ∟    ◆   s1WE5   
  ∟    ◆   s1WE6   
  ▶     ■    1WT   
  ∟    ◆   s1WT1   
  ∟    ◆   s1WT2   
  ∟    ◆   s1WT3   
  ∟    ◆   s1WT4   
  ∟    ◆   s1WT5   
  ∟    ◆   s1WT6   
  ∟    ◆   s1WT7   
  ▶     ■    2KE   
  ∟    ◆   s2KE1   
  ∟    ◆   s2KE2   
  ∟    ◆   s2KE3   
  ∟    ◆   s2KE4   
  ∟    ◆   s2KE5   
  ∟    ◆   s2KE6   
  ▶     ■    2KO   
  ∟    ◆   s2KO1   
  ∟    ◆   s2KO2   
  ∟    ◆   s2KO3   
  ∟    ◆   s2KO4   
  ▶     ■    2WE   
  ∟    ◆   s2WE1   
  ∟    ◆   s2WE2   
  ∟    ◆   s2WE3   
  ∟    ◆   s2WE4   
  ∟    ◆   s2WE5   
  ▶     ■    2WT   
  ∟    ◆   s2WT1   
  ∟    ◆   s2WT2   
  ∟    ◆   s2WT3   
  ∟    ◆   s2WT4   
  ∟    ◆   s2WT5   
  ∟    ◆   s2WT6   
  ∟    ◆   s2WT7   
  ▶     ■    3KE   
  ∟    ◆   s3KE1   
  ∟    ◆   s3KE2   
  ∟    ◆   s3KE3   
  ∟    ◆   s3KE4   
  ∟    ◆   s3KE5   
  ▶     ■    3KO   
  ∟    ◆   s3KO1   
  ∟    ◆   s3KO2   
  ∟    ◆   s3KO3   
  ∟    ◆   s3KO4   
  ∟    ◆   s3KO5   
  ∟    ◆   s3KO6   
  ▶     ■    3WE   
  ∟    ◆   s3WE1   
  ∟    ◆   s3WE2   
  ∟    ◆   s3WE3   
  ∟    ◆   s3WE4   
  ∟    ◆   s3WE5   
  ∟    ◆   s3WE6   
  ∟    ◆   s3WE7   
  ▶     ■    3WT   
  ∟    ◆   s3WT1   
  ∟    ◆   s3WT2   
  ∟    ◆   s3WT3   
  ∟    ◆   s3WT4   
  ∟    ◆   s3WT5   
  ∟    ◆   s3WT6   
  ∟    ◆   s3WT7   
  ▶     ■    AAAAAA   
  ∟    ◆   s1KE1   
  ∟    ◆   s1KE2   
  ∟    ◆   s1KE3   
  ∟    ◆   s1KE4   
  ∟    ◆   s1KE5   
  ∟    ◆   s1KE6   
  ∟    ◆   s1KO1   
  ∟    ◆   s1KO2   
  ∟    ◆   s1KO3   
  ∟    ◆   s1KO4   
  ∟    ◆   s1KO5   
  ∟    ◆   s1WE1   
  ∟    ◆   s1WE2   
  ∟    ◆   s1WE3   
  ∟    ◆   s1WE4   
  ∟    ◆   s1WE5   
  ∟    ◆   s1WE6   
  ∟    ◆   s1WT1   
  ∟    ◆   s1WT2   
  ∟    ◆   s1WT3   
  ∟    ◆   s1WT4   
  ∟    ◆   s1WT5   
  ∟    ◆   s1WT6   
  ∟    ◆   s1WT7   
  ∟    ◆   s2KE1   
  ∟    ◆   s2KE2   
  ∟    ◆   s2KE3   
  ∟    ◆   s2KE4   
  ∟    ◆   s2KE5   
  ∟    ◆   s2KE6   
  ∟    ◆   s2KO1   
  ∟    ◆   s2KO2   
  ∟    ◆   s2KO3   
  ∟    ◆   s2KO4   
  ∟    ◆   s2WE1   
  ∟    ◆   s2WE2   
  ∟    ◆   s2WE3   
  ∟    ◆   s2WE4   
  ∟    ◆   s2WE5   
  ∟    ◆   s2WT1   
  ∟    ◆   s2WT2   
  ∟    ◆   s2WT3   
  ∟    ◆   s2WT4   
  ∟    ◆   s2WT5   
  ∟    ◆   s2WT6   
  ∟    ◆   s2WT7   
  ∟    ◆   s3KE1   
  ∟    ◆   s3KE2   
  ∟    ◆   s3KE3   
  ∟    ◆   s3KE4   
  ∟    ◆   s3KE5   
  ∟    ◆   s3KO1   
  ∟    ◆   s3KO2   
  ∟    ◆   s3KO3   
  ∟    ◆   s3KO4   
  ∟    ◆   s3KO5   
  ∟    ◆   s3KO6   
  ∟    ◆   s3WE1   
  ∟    ◆   s3WE2   
  ∟    ◆   s3WE3   
  ∟    ◆   s3WE4   
  ∟    ◆   s3WE5   
  ∟    ◆   s3WE6   
  ∟    ◆   s3WE7   
  ∟    ◆   s3WT1   
  ∟    ◆   s3WT2   
  ∟    ◆   s3WT3   
  ∟    ◆   s3WT4   
  ∟    ◆   s3WT5   
  ∟    ◆   s3WT6   
  ∟    ◆   s3WT7   
  ▶     ■    s1KE1   
  ∟    ◆   s1KE1   
  ▶     ■    s1KE2   
  ∟    ◆   s1KE2   
  ▶     ■    s1KE3   
  ∟    ◆   s1KE3   
  ▶     ■    s1KE4   
  ∟    ◆   s1KE4   
  ▶     ■    s1KE5   
  ∟    ◆   s1KE5   
  ▶     ■    s1KE6   
  ∟    ◆   s1KE6   
  ▶     ■    s1KO1   
  ∟    ◆   s1KO1   
  ▶     ■    s1KO2   
  ∟    ◆   s1KO2   
  ▶     ■    s1KO3   
  ∟    ◆   s1KO3   
  ▶     ■    s1KO4   
  ∟    ◆   s1KO4   
  ▶     ■    s1KO5   
  ∟    ◆   s1KO5   
  ▶     ■    s1WE1   
  ∟    ◆   s1WE1   
  ▶     ■    s1WE2   
  ∟    ◆   s1WE2   
  ▶     ■    s1WE3   
  ∟    ◆   s1WE3   
  ▶     ■    s1WE4   
  ∟    ◆   s1WE4   
  ▶     ■    s1WE5   
  ∟    ◆   s1WE5   
  ▶     ■    s1WE6   
  ∟    ◆   s1WE6   
  ▶     ■    s1WT1   
  ∟    ◆   s1WT1   
  ▶     ■    s1WT2   
  ∟    ◆   s1WT2   
  ▶     ■    s1WT3   
  ∟    ◆   s1WT3   
  ▶     ■    s1WT4   
  ∟    ◆   s1WT4   
  ▶     ■    s1WT5   
  ∟    ◆   s1WT5   
  ▶     ■    s1WT6   
  ∟    ◆   s1WT6   
  ▶     ■    s1WT7   
  ∟    ◆   s1WT7   
  ▶     ■    s2KE1   
  ∟    ◆   s2KE1   
  ▶     ■    s2KE2   
  ∟    ◆   s2KE2   
  ▶     ■    s2KE3   
  ∟    ◆   s2KE3   
  ▶     ■    s2KE4   
  ∟    ◆   s2KE4   
  ▶     ■    s2KE5   
  ∟    ◆   s2KE5   
  ▶     ■    s2KE6   
  ∟    ◆   s2KE6   
  ▶     ■    s2KO1   
  ∟    ◆   s2KO1   
  ▶     ■    s2KO2   
  ∟    ◆   s2KO2   
  ▶     ■    s2KO3   
  ∟    ◆   s2KO3   
  ▶     ■    s2KO4   
  ∟    ◆   s2KO4   
  ▶     ■    s2WE1   
  ∟    ◆   s2WE1   
  ▶     ■    s2WE2   
  ∟    ◆   s2WE2   
  ▶     ■    s2WE3   
  ∟    ◆   s2WE3   
  ▶     ■    s2WE4   
  ∟    ◆   s2WE4   
  ▶     ■    s2WE5   
  ∟    ◆   s2WE5   
  ▶     ■    s2WT1   
  ∟    ◆   s2WT1   
  ▶     ■    s2WT2   
  ∟    ◆   s2WT2   
  ▶     ■    s2WT3   
  ∟    ◆   s2WT3   
  ▶     ■    s2WT4   
  ∟    ◆   s2WT4   
  ▶     ■    s2WT5   
  ∟    ◆   s2WT5   
  ▶     ■    s2WT6   
  ∟    ◆   s2WT6   
  ▶     ■    s2WT7   
  ∟    ◆   s2WT7   
  ▶     ■    s3KE1   
  ∟    ◆   s3KE1   
  ▶     ■    s3KE2   
  ∟    ◆   s3KE2   
  ▶     ■    s3KE3   
  ∟    ◆   s3KE3   
  ▶     ■    s3KE4   
  ∟    ◆   s3KE4   
  ▶     ■    s3KE5   
  ∟    ◆   s3KE5   
  ▶     ■    s3KO1   
  ∟    ◆   s3KO1   
  ▶     ■    s3KO2   
  ∟    ◆   s3KO2   
  ▶     ■    s3KO3   
  ∟    ◆   s3KO3   
  ▶     ■    s3KO4   
  ∟    ◆   s3KO4   
  ▶     ■    s3KO5   
  ∟    ◆   s3KO5   
  ▶     ■    s3KO6   
  ∟    ◆   s3KO6   
  ▶     ■    s3WE1   
  ∟    ◆   s3WE1   
  ▶     ■    s3WE2   
  ∟    ◆   s3WE2   
  ▶     ■    s3WE3   
  ∟    ◆   s3WE3   
  ▶     ■    s3WE4   
  ∟    ◆   s3WE4   
  ▶     ■    s3WE5   
  ∟    ◆   s3WE5   
  ▶     ■    s3WE6   
  ∟    ◆   s3WE6   
  ▶     ■    s3WE7   
  ∟    ◆   s3WE7   
  ▶     ■    s3WT1   
  ∟    ◆   s3WT1   
  ▶     ■    s3WT2   
  ∟    ◆   s3WT2   
  ▶     ■    s3WT3   
  ∟    ◆   s3WT3   
  ▶     ■    s3WT4   
  ∟    ◆   s3WT4   
  ▶     ■    s3WT5   
  ∟    ◆   s3WT5   
  ▶     ■    s3WT6   
  ∟    ◆   s3WT6   
  ▶     ■    s3WT7   
  ∟    ◆   s3WT7   
   
 
 
 If the lines for some categories do not extend all the way to the right end of the x-axis, that means that at least one of the samples in that category does not have that many samples. 
 
  
 
  Group  Seqs/Sample 
 PD_whole_tree Ave.  PD_whole_tree Err. 
 chao1 Ave.  chao1 Err. 
 goods_coverage Ave.  goods_coverage Err. 
 observed_species Ave.  observed_species Err. 
 shannon Ave.  shannon Err. 
 simpson Ave.  simpson Err. 
 
 
 1KE  10.0 
      1.885       0.120 
     30.817       3.739 
      0.208       0.039 
      8.867       0.269 
      3.077       0.073 
      0.872       0.013 
 
 1KE  1010.0 
     17.660       0.735 
    704.748      77.166 
      0.827       0.015 
    272.550      17.149 
      6.457       0.205 
      0.967       0.008 
 
 1KE  2010.0 
     23.290       1.010 
   1009.885      63.977 
      0.872       0.009 
    415.550      26.807 
      6.639       0.214 
      0.967       0.009 
 
 1KE  3010.0 
     27.451       1.210 
   1227.405     117.002 
      0.893       0.010 
    532.533      43.196 
      6.731       0.238 
      0.967       0.009 
 
 1KE  4010.0 
     30.835       1.549 
   1444.268     109.170 
      0.905       0.008 
    637.267      49.886 
      6.790       0.235 
      0.967       0.009 
 
 1KE  5010.0 
     33.449       1.635 
   1530.094     104.680 
      0.917       0.006 
    719.233      55.067 
      6.825       0.238 
      0.967       0.009 
 
 1KE  6010.0 
     36.244       1.714 
   1683.439     114.794 
      0.923       0.006 
    802.850      62.039 
      6.859       0.243 
      0.967       0.008 
 
 1KE  7010.0 
     38.334       1.799 
   1794.018     110.507 
      0.930       0.005 
    874.117      64.665 
      6.885       0.238 
      0.968       0.008 
 
 1KE  8010.0 
     40.447       2.000 
   1903.077     142.324 
      0.934       0.005 
    943.267      72.138 
      6.906       0.241 
      0.968       0.008 
 
 1KE  9010.0 
     42.412       1.792 
   1992.354     106.063 
      0.938       0.004 
   1008.417      69.783 
      6.919       0.244 
      0.967       0.009 
 
 1KE  10010.0 
     44.078       2.020 
   2083.217      93.221 
      0.942       0.004 
   1069.033      77.058 
      6.932       0.245 
      0.967       0.009 
 
 1KE  11010.0 
     45.584       1.972 
   2115.911     131.927 
      0.946       0.004 
   1117.400      81.607 
      6.941       0.240 
      0.967       0.008 
 
 1KE  12010.0 
     47.010       2.270 
   2207.775     137.867 
      0.948       0.004 
   1172.633      87.935 
      6.947       0.252 
      0.967       0.009 
 
 1KE  13010.0 
     48.664       2.087 
   2279.919     137.705 
      0.951       0.003 
   1227.367      87.238 
      6.961       0.241 
      0.967       0.008 
 
 1KE  14010.0 
     49.869       2.315 
   2342.126     118.199 
      0.953       0.003 
   1272.083      90.569 
      6.965       0.248 
      0.967       0.009 
 
 1KE  15010.0 
     51.069       2.328 
   2413.681     148.324 
      0.955       0.003 
   1317.567      94.083 
      6.974       0.243 
      0.968       0.008 
 
 1KE  16010.0 
     52.438       2.397 
   2455.104     137.202 
      0.957       0.003 
   1362.583      96.888 
      6.976       0.242 
      0.967       0.008 
 
 1KE  17010.0 
     53.728       2.310 
   2527.860     131.842 
      0.958       0.002 
   1406.867      96.235 
      6.985       0.247 
      0.968       0.008 
 
 1KE  18010.0 
     54.593       2.362 
   2545.903     128.317 
      0.960       0.002 
   1444.483      98.751 
      6.987       0.243 
      0.967       0.008 
 
 1KE  19010.0 
     55.691       2.434 
   2591.532     134.771 
      0.961       0.002 
   1484.783     100.435 
      6.994       0.250 
      0.967       0.009 
 
 1KE  20010.0 
     56.861       2.441 
   2653.931     168.943 
      0.962       0.002 
   1526.183     105.912 
      6.999       0.249 
      0.967       0.009 
 
 1KE  21010.0 
     57.691       2.549 
   2696.197     148.004 
      0.964       0.002 
   1561.333     105.566 
      7.003       0.248 
      0.968       0.009 
 
 1KE  22010.0 
     58.571       2.619 
   2721.954     146.945 
      0.965       0.002 
   1596.700     111.953 
      7.007       0.250 
      0.967       0.009 
 
 1KE  23010.0 
     59.608       2.670 
   2755.153     174.431 
      0.966       0.002 
   1629.417     114.554 
      7.009       0.247 
      0.967       0.008 
 
 1KE  24010.0 
     60.467       2.613 
   2795.929     182.246 
      0.967       0.002 
   1665.667     117.100 
      7.014       0.252 
      0.967       0.009 
 
 1KE  25010.0 
     61.343       2.600 
   2814.437     152.202 
      0.968       0.002 
   1694.933     112.764 
      7.014       0.249 
      0.967       0.009 
 
 1KE  26010.0 
     62.224       2.687 
   2884.685     163.911 
      0.969       0.002 
   1731.600     116.675 
      7.023       0.246 
      0.968       0.008 
 
 1KE  27010.0 
     62.950       2.637 
   2916.174     180.286 
      0.969       0.002 
   1760.400     118.966 
      7.023       0.248 
      0.968       0.009 
 
 1KO  10.0 
      2.008       0.167 
     35.200       3.521 
      0.162       0.031 
      9.100       0.190 
      3.128       0.044 
      0.878       0.005 
 
 1KO  1010.0 
     17.858       0.353 
    647.701      22.161 
      0.835       0.004 
    272.260       5.522 
      6.541       0.169 
      0.970       0.006 
 
 1KO  2010.0 
     23.627       0.432 
    976.449      49.610 
      0.877       0.004 
    414.720       6.284 
      6.733       0.164 
      0.970       0.006 
 
 1KO  3010.0 
     27.459       0.239 
   1212.119      35.437 
      0.897       0.003 
    526.260       5.538 
      6.825       0.168 
      0.970       0.006 
 
 1KO  4010.0 
     30.557       0.473 
   1397.445      49.972 
      0.911       0.002 
    617.640       7.238 
      6.878       0.176 
      0.970       0.006 
 
 1KO  5010.0 
     33.509       0.669 
   1554.072      59.094 
      0.919       0.003 
    704.920      13.058 
      6.915       0.170 
      0.970       0.006 
 
 1KO  6010.0 
     35.757       0.660 
   1667.577      42.301 
      0.926       0.002 
    782.460      13.631 
      6.944       0.171 
      0.970       0.006 
 
 1KO  7010.0 
     38.073       0.626 
   1794.081      56.656 
      0.932       0.002 
    852.160      17.835 
      6.973       0.169 
      0.970       0.006 
 
 1KO  8010.0 
     39.963       0.766 
   1902.572      45.213 
      0.936       0.002 
    918.120      16.826 
      6.996       0.166 
      0.970       0.006 
 
 1KO  9010.0 
     41.816       0.799 
   2010.110      56.336 
      0.940       0.002 
    979.840      18.054 
      7.004       0.166 
      0.970       0.006 
 
 1KO  10010.0 
     43.576       0.770 
   2086.202      47.267 
      0.943       0.002 
   1039.900      20.260 
      7.022       0.173 
      0.970       0.006 
 
 1KO  11010.0 
     44.947       0.765 
   2149.288      26.872 
      0.947       0.001 
   1089.460      19.769 
      7.027       0.165 
      0.970       0.006 
 
 1KO  12010.0 
     46.563       0.909 
   2230.742      53.851 
      0.949       0.001 
   1144.500      25.696 
      7.039       0.167 
      0.970       0.006 
 
 1KO  13010.0 
     48.081       1.079 
   2311.533      64.154 
      0.951       0.001 
   1197.980      26.630 
      7.049       0.172 
      0.970       0.006 
 
 1KO  14010.0 
     49.256       0.888 
   2363.560      62.720 
      0.953       0.001 
   1241.480      25.047 
      7.051       0.166 
      0.970       0.006 
 
 1KO  15010.0 
     50.791       1.028 
   2410.902      75.529 
      0.955       0.001 
   1290.560      30.891 
      7.060       0.168 
      0.970       0.006 
 
 1KO  16010.0 
     51.699       1.204 
   2458.937      69.480 
      0.957       0.001 
   1330.660      31.614 
      7.066       0.173 
      0.970       0.006 
 
 1KO  17010.0 
     53.153       1.113 
   2541.480      48.851 
      0.958       0.001 
   1379.800      28.812 
      7.071       0.171 
      0.970       0.006 
 
 1KO  18010.0 
     53.932       1.072 
   2544.332      72.145 
      0.960       0.001 
   1413.180      31.173 
      7.078       0.168 
      0.970       0.006 
 
 1KO  19010.0 
     55.090       1.127 
   2610.097      68.589 
      0.961       0.001 
   1454.580      26.198 
      7.083       0.169 
      0.970       0.006 
 
 1KO  20010.0 
     56.361       1.085 
   2687.845      52.908 
      0.962       0.001 
   1499.940      29.854 
      7.088       0.172 
      0.970       0.006 
 
 1KO  21010.0 
     57.073       1.292 
   2675.987      75.147 
      0.964       0.001 
   1529.020      32.108 
      7.091       0.167 
      0.970       0.006 
 
 1KO  22010.0 
     58.123       1.174 
   2745.029      92.774 
      0.965       0.001 
   1569.000      35.025 
      7.093       0.169 
      0.970       0.006 
 
 1KO  23010.0 
     58.869       1.429 
   2769.153      86.429 
      0.966       0.001 
   1600.580      38.219 
      7.097       0.168 
      0.970       0.006 
 
 1KO  24010.0 
     59.805       1.194 
   2832.661      67.683 
      0.967       0.001 
   1637.980      34.972 
      7.103       0.168 
      0.970       0.006 
 
 1KO  25010.0 
     60.791       1.268 
   2862.759      61.858 
      0.968       0.001 
   1672.180      34.844 
      7.105       0.170 
      0.970       0.006 
 
 1KO  26010.0 
     61.490       1.270 
   2908.988      91.759 
      0.968       0.001 
   1701.940      34.823 
      7.110       0.170 
      0.970       0.006 
 
 1KO  27010.0 
     62.291       1.395 
   2945.242      95.109 
      0.969       0.001 
   1731.460      39.401 
      7.111       0.170 
      0.970       0.006 
 
 1WE  10.0 
      1.967       0.113 
     32.542       7.633 
      0.213       0.076 
      8.783       0.430 
      3.053       0.098 
      0.868       0.013 
 
 1WE  1010.0 
     16.827       0.206 
    592.234      25.679 
      0.852       0.006 
    249.400       9.620 
      6.358       0.214 
      0.963       0.013 
 
 1WE  2010.0 
     21.769       0.361 
    860.493      48.548 
      0.890       0.004 
    376.117      13.012 
      6.521       0.249 
      0.963       0.015 
 
 1WE  3010.0 
     25.606       0.564 
   1051.287      39.248 
      0.910       0.003 
    470.800      17.157 
      6.597       0.221 
      0.963       0.014 
 
 1WE  4010.0 
     28.544       0.519 
   1245.310      75.316 
      0.921       0.003 
    556.333      20.928 
      6.646       0.242 
      0.963       0.014 
 
 1WE  5010.0 
     31.289       0.754 
   1389.359      85.541 
      0.928       0.003 
    633.083      21.849 
      6.684       0.234 
      0.963       0.014 
 
 1WE  6010.0 
     33.465       0.787 
   1480.205      86.988 
      0.935       0.003 
    698.817      23.823 
      6.704       0.238 
      0.963       0.014 
 
 1WE  7010.0 
     35.443       0.607 
   1574.693      86.942 
      0.940       0.003 
    761.400      25.581 
      6.722       0.237 
      0.963       0.014 
 
 1WE  8010.0 
     37.283       0.718 
   1660.394      88.067 
      0.944       0.003 
    823.067      25.659 
      6.746       0.225 
      0.963       0.014 
 
 1WE  9010.0 
     39.025       1.001 
   1771.072      92.716 
      0.947       0.002 
    876.433      30.317 
      6.762       0.243 
      0.963       0.014 
 
 1WE  10010.0 
     40.554       0.914 
   1841.669      86.024 
      0.950       0.002 
    925.167      32.593 
      6.767       0.241 
      0.963       0.014 
 
 1WE  11010.0 
     42.223       0.946 
   1945.999      84.735 
      0.952       0.002 
    981.583      35.129 
      6.778       0.241 
      0.963       0.014 
 
 1WE  12010.0 
     43.455       0.906 
   2008.714      92.664 
      0.955       0.002 
   1024.833      34.671 
      6.794       0.240 
      0.963       0.014 
 
 1WE  13010.0 
     44.585       0.771 
   2045.394      78.903 
      0.957       0.002 
   1061.233      32.795 
      6.790       0.236 
      0.963       0.014 
 
 1WE  14010.0 
     45.933       1.058 
   2126.440     106.097 
      0.958       0.002 
   1110.483      38.410 
      6.804       0.238 
      0.963       0.014 
 
 1WE  15010.0 
     47.143       0.916 
   2176.199      97.986 
      0.960       0.002 
   1147.333      35.595 
      6.809       0.234 
      0.963       0.014 
 
 1WE  16010.0 
     48.150       0.954 
   2223.230      86.091 
      0.962       0.002 
   1186.800      36.566 
      6.812       0.233 
      0.963       0.014 
 
 1WE  17010.0 
     49.327       1.128 
   2301.638     103.379 
      0.963       0.002 
   1226.683      41.831 
      6.820       0.236 
      0.963       0.014 
 
 1WE  18010.0 
     50.399       1.271 
   2317.918      98.209 
      0.964       0.002 
   1263.367      45.885 
      6.827       0.238 
      0.963       0.014 
 
 1WE  19010.0 
     51.160       1.075 
   2367.523      84.446 
      0.966       0.002 
   1294.550      43.911 
      6.826       0.240 
      0.963       0.014 
 
 1WE  20010.0 
     52.142       1.194 
   2407.586      97.509 
      0.967       0.002 
   1331.000      45.049 
      6.835       0.237 
      0.964       0.014 
 
 1WE  21010.0 
     53.138       1.284 
   2474.750      84.556 
      0.968       0.002 
   1363.250      47.544 
      6.837       0.239 
      0.963       0.014 
 
 1WE  22010.0 
     53.995       1.450 
   2505.237      85.237 
      0.968       0.002 
   1396.150      54.304 
      6.839       0.242 
      0.963       0.014 
 
 1WE  23010.0 
     54.838       1.379 
   2539.013      93.489 
      0.969       0.001 
   1425.967      49.978 
      6.843       0.239 
      0.963       0.014 
 
 1WE  24010.0 
     55.639       1.335 
   2583.762      98.498 
      0.970       0.001 
   1454.167      49.733 
      6.843       0.238 
      0.963       0.014 
 
 1WE  25010.0 
     56.391       1.422 
   2630.268     109.006 
      0.971       0.001 
   1485.450      51.089 
      6.846       0.235 
      0.963       0.014 
 
 1WE  26010.0 
     57.256       1.570 
   2647.356      86.384 
      0.972       0.001 
   1515.283      57.133 
      6.849       0.239 
      0.963       0.014 
 
 1WE  27010.0 
     57.930       1.497 
   2688.411      86.154 
      0.972       0.001 
   1542.333      54.289 
      6.853       0.240 
      0.963       0.014 
 
 1WT  10.0 
      1.825       0.122 
     29.800       4.397 
      0.219       0.036 
      8.800       0.214 
      3.065       0.048 
      0.871       0.006 
 
 1WT  1010.0 
     16.832       0.353 
    696.063      45.700 
      0.828       0.006 
    271.871       9.428 
      6.461       0.141 
      0.967       0.005 
 
 1WT  2010.0 
     22.462       0.411 
   1038.180      39.003 
      0.870       0.005 
    420.914      13.764 
      6.683       0.133 
      0.968       0.004 
 
 1WT  3010.0 
     26.482       0.342 
   1242.340      64.852 
      0.891       0.005 
    540.586      18.465 
      6.774       0.133 
      0.968       0.004 
 
 1WT  4010.0 
     29.738       0.403 
   1410.828      60.268 
      0.906       0.004 
    639.000      20.904 
      6.827       0.135 
      0.968       0.004 
 
 1WT  5010.0 
     32.516       0.477 
   1557.833      97.628 
      0.915       0.004 
    730.914      26.219 
      6.869       0.132 
      0.968       0.004 
 
 1WT  6010.0 
     34.885       0.584 
   1667.220      69.745 
      0.923       0.003 
    811.643      29.454 
      6.905       0.136 
      0.969       0.004 
 
 1WT  7010.0 
     36.974       0.475 
   1785.202      50.948 
      0.929       0.002 
    881.371      28.559 
      6.918       0.139 
      0.968       0.004 
 
 1WT  8010.0 
     38.958       0.792 
   1900.089     111.924 
      0.934       0.003 
    948.571      38.306 
      6.933       0.149 
      0.968       0.004 
 
 1WT  9010.0 
     40.947       0.691 
   1990.195      77.361 
      0.938       0.003 
   1017.229      37.677 
      6.956       0.139 
      0.968       0.004 
 
 1WT  10010.0 
     42.523       0.801 
   2053.224     129.528 
      0.942       0.003 
   1075.257      44.268 
      6.968       0.138 
      0.968       0.004 
 
 1WT  11010.0 
     44.234       0.891 
   2148.722      86.118 
      0.945       0.003 
   1134.886      44.141 
      6.985       0.138 
      0.968       0.004 
 
 1WT  12010.0 
     45.532       0.805 
   2206.194      85.784 
      0.948       0.002 
   1183.143      42.409 
      6.990       0.138 
      0.969       0.004 
 
 1WT  13010.0 
     47.040       0.848 
   2292.815      98.482 
      0.950       0.002 
   1237.243      42.802 
      7.006       0.140 
      0.969       0.004 
 
 1WT  14010.0 
     48.169       0.831 
   2339.001      63.020 
      0.953       0.002 
   1285.271      43.596 
      7.009       0.136 
      0.968       0.004 
 
 1WT  15010.0 
     49.464       0.784 
   2405.749      80.888 
      0.955       0.002 
   1331.657      50.002 
      7.017       0.139 
      0.969       0.004 
 
 1WT  16010.0 
     50.563       0.831 
   2437.645      79.808 
      0.957       0.002 
   1370.286      47.799 
      7.021       0.140 
      0.969       0.004 
 
 1WT  17010.0 
     51.759       1.085 
   2509.812     119.007 
      0.958       0.002 
   1418.871      55.253 
      7.031       0.140 
      0.969       0.004 
 
 1WT  18010.0 
     52.824       0.803 
   2569.281     117.216 
      0.960       0.002 
   1458.743      54.773 
      7.033       0.141 
      0.968       0.004 
 
 1WT  19010.0 
     53.918       1.181 
   2593.051     107.184 
      0.961       0.002 
   1497.557      58.517 
      7.039       0.140 
      0.968       0.004 
 
 1WT  20010.0 
     54.696       1.107 
   2609.032     108.767 
      0.963       0.002 
   1531.086      58.405 
      7.040       0.142 
      0.969       0.004 
 
 1WT  21010.0 
     55.775       1.159 
   2683.129     110.497 
      0.964       0.002 
   1570.929      61.052 
      7.046       0.139 
      0.968       0.004 
 
 1WT  22010.0 
     56.616       1.346 
   2726.252     141.038 
      0.965       0.002 
   1605.829      64.680 
      7.051       0.142 
      0.969       0.004 
 
 1WT  23010.0 
     57.521       1.154 
   2760.281      96.233 
      0.966       0.001 
   1641.243      60.754 
      7.053       0.141 
      0.969       0.004 
 
 1WT  24010.0 
     58.503       1.076 
   2801.779      90.348 
      0.967       0.001 
   1677.129      61.115 
      7.057       0.140 
      0.968       0.004 
 
 1WT  25010.0 
     59.250       1.354 
   2845.525     114.852 
      0.968       0.001 
   1710.300      65.184 
      7.061       0.142 
      0.968       0.004 
 
 1WT  26010.0 
     59.942       1.153 
   2856.890     104.020 
      0.969       0.001 
   1739.986      64.312 
      7.062       0.141 
      0.968       0.004 
 
 1WT  27010.0 
     60.630       1.374 
   2909.366     126.424 
      0.970       0.001 
   1767.186      68.298 
      7.065       0.140 
      0.968       0.004 
 
 2KE  10.0 
      2.086       0.146 
     37.500       6.355 
      0.140       0.052 
      9.250       0.281 
      3.164       0.059 
      0.883       0.007 
 
 2KE  1010.0 
     17.861       0.558 
    673.020      50.928 
      0.832       0.006 
    277.000       8.783 
      6.741       0.179 
      0.977       0.005 
 
 2KE  2010.0 
     23.530       0.477 
    996.804      64.020 
      0.874       0.006 
    422.767      10.988 
      6.954       0.173 
      0.978       0.005 
 
 2KE  3010.0 
     27.631       0.644 
   1220.678      75.985 
      0.895       0.005 
    535.017      16.853 
      7.035       0.185 
      0.978       0.005 
 
 2KE  4010.0 
     30.989       0.516 
   1410.689      79.188 
      0.908       0.005 
    632.500      17.821 
      7.094       0.168 
      0.978       0.005 
 
 2KE  5010.0 
     33.707       0.442 
   1537.607      83.162 
      0.918       0.004 
    718.533      17.103 
      7.127       0.173 
      0.978       0.005 
 
 2KE  6010.0 
     36.156       0.593 
   1683.307      57.806 
      0.925       0.003 
    799.400      24.691 
      7.163       0.171 
      0.978       0.005 
 
 2KE  7010.0 
     38.622       0.569 
   1803.407      53.349 
      0.930       0.003 
    874.100      24.904 
      7.191       0.183 
      0.978       0.005 
 
 2KE  8010.0 
     40.548       0.737 
   1920.477      62.077 
      0.935       0.002 
    938.483      27.768 
      7.204       0.181 
      0.978       0.005 
 
 2KE  9010.0 
     42.475       0.712 
   2005.732      70.758 
      0.939       0.003 
   1003.067      33.606 
      7.218       0.179 
      0.978       0.005 
 
 2KE  10010.0 
     44.093       0.816 
   2097.176      99.740 
      0.943       0.003 
   1058.850      34.945 
      7.237       0.174 
      0.978       0.005 
 
 2KE  11010.0 
     45.729       0.675 
   2179.112      67.359 
      0.945       0.002 
   1116.483      38.078 
      7.245       0.174 
      0.978       0.005 
 
 2KE  12010.0 
     47.422       0.779 
   2265.356      91.968 
      0.948       0.002 
   1172.883      42.462 
      7.260       0.174 
      0.978       0.005 
 
 2KE  13010.0 
     48.835       0.784 
   2302.509      67.895 
      0.951       0.002 
   1218.017      44.173 
      7.260       0.171 
      0.978       0.005 
 
 2KE  14010.0 
     50.007       0.827 
   2381.181      74.838 
      0.953       0.002 
   1268.833      44.898 
      7.270       0.178 
      0.978       0.005 
 
 2KE  15010.0 
     51.318       1.012 
   2425.268      95.997 
      0.955       0.002 
   1314.967      49.673 
      7.280       0.177 
      0.978       0.005 
 
 2KE  16010.0 
     52.607       0.653 
   2492.236     112.691 
      0.956       0.002 
   1358.700      50.984 
      7.283       0.176 
      0.978       0.005 
 
 2KE  17010.0 
     53.891       0.847 
   2566.912      65.130 
      0.958       0.002 
   1405.450      50.096 
      7.294       0.174 
      0.978       0.005 
 
 2KE  18010.0 
     54.926       0.714 
   2570.849     107.484 
      0.960       0.002 
   1439.883      55.546 
      7.294       0.176 
      0.978       0.005 
 
 2KE  19010.0 
     55.965       0.796 
   2636.562     104.051 
      0.961       0.002 
   1482.133      54.739 
      7.299       0.176 
      0.978       0.005 
 
 2KE  20010.0 
     57.009       0.760 
   2673.295      74.480 
      0.962       0.001 
   1520.550      51.185 
      7.306       0.175 
      0.978       0.005 
 
 2KE  21010.0 
     58.008       0.797 
   2738.662      96.070 
      0.963       0.002 
   1560.667      59.808 
      7.309       0.176 
      0.978       0.005 
 
 2KE  22010.0 
     58.957       0.849 
   2792.787      86.265 
      0.964       0.001 
   1596.267      56.555 
      7.313       0.177 
      0.978       0.005 
 
 2KE  23010.0 
     59.851       0.802 
   2795.311      94.191 
      0.966       0.001 
   1628.200      59.844 
      7.317       0.172 
      0.978       0.005 
 
 2KE  24010.0 
     60.697       0.987 
   2847.746      57.986 
      0.966       0.001 
   1663.717      63.869 
      7.321       0.174 
      0.978       0.005 
 
 2KE  25010.0 
     61.608       0.895 
   2890.666      93.421 
      0.967       0.001 
   1695.950      61.822 
      7.322       0.175 
      0.978       0.005 
 
 2KE  26010.0 
     62.349       1.000 
   2893.720      91.721 
      0.968       0.001 
   1726.133      62.464 
      7.325       0.176 
      0.978       0.005 
 
 2KE  27010.0 
     63.266       0.979 
   2953.035     100.952 
      0.969       0.001 
   1762.383      63.767 
      7.332       0.173 
      0.978       0.005 
 
 2KO  10.0 
      2.080       0.092 
     38.550       6.203 
      0.140       0.053 
      9.200       0.308 
      3.144       0.071 
      0.879       0.009 
 
 2KO  1010.0 
     17.750       0.442 
    653.310      14.793 
      0.841       0.003 
    264.100       4.108 
      6.643       0.137 
      0.975       0.004 
 
 2KO  2010.0 
     22.869       0.305 
    948.045      62.266 
      0.883       0.004 
    396.400       4.379 
      6.821       0.136 
      0.976       0.004 
 
 2KO  3010.0 
     26.866       0.467 
   1152.444      76.074 
      0.903       0.003 
    502.025       5.468 
      6.911       0.132 
      0.976       0.004 
 
 2KO  4010.0 
     29.836       0.557 
   1337.231      83.318 
      0.915       0.003 
    589.900       6.872 
      6.951       0.132 
      0.976       0.004 
 
 2KO  5010.0 
     33.010       0.709 
   1535.668      94.323 
      0.921       0.002 
    680.125       6.593 
      7.004       0.133 
      0.976       0.004 
 
 2KO  6010.0 
     35.131       0.579 
   1655.959      68.552 
      0.929       0.002 
    745.875       9.539 
      7.014       0.124 
      0.976       0.004 
 
 2KO  7010.0 
     37.164       0.746 
   1747.151      75.001 
      0.934       0.002 
    812.375      13.124 
      7.029       0.132 
      0.976       0.004 
 
 2KO  8010.0 
     39.396       0.696 
   1874.145      83.693 
      0.938       0.002 
    883.675      12.224 
      7.067       0.127 
      0.976       0.004 
 
 2KO  9010.0 
     40.889       0.867 
   1964.267     125.966 
      0.942       0.002 
    936.425      16.572 
      7.071       0.133 
      0.976       0.004 
 
 2KO  10010.0 
     42.867       0.739 
   2066.526      90.159 
      0.944       0.002 
   1001.950      13.977 
      7.081       0.136 
      0.976       0.004 
 
 2KO  11010.0 
     44.138       1.164 
   2121.601     113.611 
      0.948       0.002 
   1048.625      23.770 
      7.091       0.134 
      0.976       0.004 
 
 2KO  12010.0 
     45.722       1.099 
   2198.200     134.012 
      0.950       0.002 
   1106.325      24.213 
      7.110       0.131 
      0.976       0.004 
 
 2KO  13010.0 
     46.997       0.924 
   2253.099     102.867 
      0.952       0.002 
   1152.375      20.739 
      7.111       0.133 
      0.976       0.004 
 
 2KO  14010.0 
     48.290       1.299 
   2290.866     116.483 
      0.955       0.002 
   1189.925      25.810 
      7.112       0.134 
      0.976       0.004 
 
 2KO  15010.0 
     49.787       1.394 
   2362.002     119.572 
      0.956       0.002 
   1244.975      30.004 
      7.125       0.132 
      0.976       0.004 
 
 2KO  16010.0 
     50.882       1.225 
   2416.495      71.498 
      0.958       0.002 
   1289.525      25.266 
      7.137       0.126 
      0.976       0.004 
 
 2KO  17010.0 
     52.018       1.433 
   2489.346      53.107 
      0.959       0.001 
   1330.100      29.876 
      7.133       0.137 
      0.976       0.004 
 
 2KO  18010.0 
     53.080       1.317 
   2535.781      58.669 
      0.960       0.002 
   1371.525      30.995 
      7.141       0.131 
      0.976       0.004 
 
 2KO  19010.0 
     54.344       1.513 
   2558.558     111.499 
      0.962       0.002 
   1410.850      38.546 
      7.147       0.135 
      0.976       0.004 
 
 2KO  20010.0 
     55.384       1.551 
   2592.371      95.650 
      0.963       0.002 
   1446.575      36.467 
      7.152       0.136 
      0.976       0.004 
 
 2KO  21010.0 
     56.245       1.651 
   2670.671     105.531 
      0.964       0.002 
   1484.475      39.924 
      7.152       0.137 
      0.976       0.004 
 
 2KO  22010.0 
     57.106       1.606 
   2698.356      64.078 
      0.965       0.001 
   1517.850      35.769 
      7.156       0.134 
      0.976       0.004 
 
 2KO  23010.0 
     57.984       1.732 
   2702.750     102.800 
      0.966       0.002 
   1552.300      44.494 
      7.161       0.134 
      0.976       0.004 
 
 2KO  24010.0 
     58.869       1.747 
   2778.096      79.275 
      0.967       0.001 
   1582.375      42.224 
      7.163       0.133 
      0.976       0.004 
 
 2KO  25010.0 
     59.726       1.614 
   2788.352      64.546 
      0.968       0.001 
   1614.875      41.664 
      7.165       0.134 
      0.976       0.004 
 
 2KO  26010.0 
     60.536       1.660 
   2838.519      61.596 
      0.969       0.001 
   1649.750      45.474 
      7.171       0.134 
      0.976       0.004 
 
 2KO  27010.0 
     61.265       1.700 
   2836.125      54.021 
      0.970       0.001 
   1673.550      43.414 
      7.170       0.134 
      0.976       0.004 
 
 2WE  10.0 
      2.124       0.189 
     31.680       6.063 
      0.188       0.053 
      9.020       0.279 
      3.120       0.059 
      0.879       0.007 
 
 2WE  1010.0 
     17.194       0.405 
    621.017      42.716 
      0.839       0.010 
    265.980      16.172 
      6.517       0.175 
      0.971       0.006 
 
 2WE  2010.0 
     22.613       0.335 
    914.453      62.225 
      0.883       0.007 
    399.760      19.934 
      6.699       0.191 
      0.971       0.006 
 
 2WE  3010.0 
     26.885       0.550 
   1113.397      52.909 
      0.902       0.005 
    512.880      23.643 
      6.802       0.196 
      0.972       0.006 
 
 2WE  4010.0 
     29.906       0.462 
   1271.435      80.536 
      0.915       0.005 
    602.060      32.362 
      6.856       0.206 
      0.972       0.006 
 
 2WE  5010.0 
     32.600       0.334 
   1443.052      43.125 
      0.923       0.003 
    682.660      32.392 
      6.885       0.198 
      0.972       0.006 
 
 2WE  6010.0 
     34.980       0.662 
   1564.468      48.748 
      0.931       0.003 
    754.360      28.415 
      6.921       0.185 
      0.972       0.006 
 
 2WE  7010.0 
     36.974       0.525 
   1650.254      40.525 
      0.936       0.002 
    817.840      37.368 
      6.936       0.201 
      0.972       0.006 
 
 2WE  8010.0 
     38.844       0.911 
   1786.345      67.767 
      0.940       0.003 
    879.780      40.092 
      6.948       0.196 
      0.972       0.006 
 
 2WE  9010.0 
     40.786       0.658 
   1891.196      41.773 
      0.944       0.002 
    941.460      39.240 
      6.973       0.198 
      0.972       0.006 
 
 2WE  10010.0 
     42.434       0.703 
   1964.417      22.607 
      0.947       0.001 
    997.760      36.408 
      6.984       0.201 
      0.972       0.006 
 
 2WE  11010.0 
     43.834       0.893 
   2030.692      30.338 
      0.950       0.001 
   1044.480      39.191 
      6.993       0.201 
      0.972       0.006 
 
 2WE  12010.0 
     45.401       0.901 
   2091.563      54.520 
      0.952       0.002 
   1097.460      41.388 
      7.004       0.198 
      0.972       0.006 
 
 2WE  13010.0 
     46.513       0.901 
   2144.168      45.709 
      0.955       0.001 
   1137.080      42.547 
      7.005       0.200 
      0.972       0.006 
 
 2WE  14010.0 
     47.914       0.976 
   2205.404      23.098 
      0.956       0.001 
   1186.280      41.400 
      7.014       0.202 
      0.972       0.006 
 
 2WE  15010.0 
     49.124       1.003 
   2254.508      24.263 
      0.958       0.001 
   1228.020      41.101 
      7.028       0.200 
      0.972       0.006 
 
 2WE  16010.0 
     50.311       1.068 
   2312.290      28.721 
      0.960       0.001 
   1269.740      43.581 
      7.030       0.193 
      0.972       0.006 
 
 2WE  17010.0 
     51.414       1.183 
   2360.898      56.744 
      0.961       0.001 
   1305.560      44.572 
      7.033       0.199 
      0.972       0.006 
 
 2WE  18010.0 
     52.424       0.991 
   2430.036      58.946 
      0.963       0.001 
   1343.040      48.042 
      7.037       0.198 
      0.972       0.006 
 
 2WE  19010.0 
     53.525       0.996 
   2455.589      51.811 
      0.964       0.001 
   1383.000      49.293 
      7.044       0.199 
      0.972       0.006 
 
 2WE  20010.0 
     54.589       1.221 
   2507.689      61.163 
      0.965       0.001 
   1416.640      50.913 
      7.044       0.200 
      0.972       0.006 
 
 2WE  21010.0 
     55.470       1.169 
   2538.419      45.038 
      0.966       0.001 
   1455.620      43.978 
      7.051       0.199 
      0.972       0.006 
 
 2WE  22010.0 
     56.385       1.221 
   2580.101      58.990 
      0.967       0.001 
   1484.180      44.460 
      7.054       0.196 
      0.972       0.006 
 
 2WE  23010.0 
     57.135       1.145 
   2623.119      49.666 
      0.968       0.001 
   1515.640      49.154 
      7.051       0.200 
      0.972       0.006 
 
 2WE  24010.0 
     58.082       1.117 
   2670.767      41.697 
      0.969       0.001 
   1550.500      47.812 
      7.061       0.200 
      0.972       0.006 
 
 2WE  25010.0 
     58.936       1.195 
   2695.112      59.083 
      0.970       0.001 
   1579.300      49.268 
      7.062       0.202 
      0.972       0.006 
 
 2WE  26010.0 
     59.774       1.178 
   2727.280      60.500 
      0.971       0.001 
   1609.440      49.020 
      7.066       0.199 
      0.972       0.006 
 
 2WE  27010.0 
     60.501       1.129 
   2760.365      31.732 
      0.971       0.000 
   1636.020      48.392 
      7.069       0.199 
      0.972       0.006 
 
 2WT  10.0 
      1.844       0.173 
     26.879       6.187 
      0.256       0.087 
      8.586       0.549 
      3.018       0.128 
      0.866       0.016 
 
 2WT  1010.0 
     17.132       0.831 
    677.601      36.143 
      0.830       0.009 
    274.871      11.579 
      6.557       0.113 
      0.970       0.004 
 
 2WT  2010.0 
     22.542       0.929 
    977.091      64.007 
      0.875       0.006 
    415.657      18.819 
      6.737       0.130 
      0.970       0.005 
 
 2WT  3010.0 
     26.490       1.337 
   1188.232      58.576 
      0.896       0.005 
    530.757      25.732 
      6.824       0.143 
      0.970       0.005 
 
 2WT  4010.0 
     29.627       1.287 
   1343.762      70.646 
      0.910       0.003 
    627.529      24.775 
      6.886       0.141 
      0.970       0.005 
 
 2WT  5010.0 
     32.465       1.692 
   1504.857      87.118 
      0.919       0.004 
    713.771      32.664 
      6.915       0.146 
      0.970       0.005 
 
 2WT  6010.0 
     34.938       1.639 
   1617.217      79.178 
      0.926       0.004 
    792.986      35.505 
      6.954       0.146 
      0.970       0.005 
 
 2WT  7010.0 
     36.930       1.818 
   1713.407      86.918 
      0.933       0.003 
    859.629      41.737 
      6.977       0.154 
      0.971       0.004 
 
 2WT  8010.0 
     38.972       1.702 
   1803.655     103.144 
      0.937       0.003 
    926.871      40.246 
      6.990       0.152 
      0.970       0.005 
 
 2WT  9010.0 
     40.640       2.013 
   1907.198     117.287 
      0.941       0.003 
    987.529      45.593 
      7.013       0.151 
      0.971       0.004 
 
 2WT  10010.0 
     42.191       1.951 
   1979.951     121.863 
      0.945       0.003 
   1041.786      45.216 
      7.017       0.150 
      0.970       0.005 
 
 2WT  11010.0 
     44.003       2.144 
   2070.869     106.402 
      0.947       0.003 
   1101.143      48.434 
      7.037       0.149 
      0.971       0.005 
 
 2WT  12010.0 
     45.352       2.193 
   2145.556     129.022 
      0.950       0.003 
   1151.143      51.274 
      7.044       0.147 
      0.971       0.004 
 
 2WT  13010.0 
     46.670       2.140 
   2203.853     124.669 
      0.953       0.003 
   1196.400      53.993 
      7.048       0.150 
      0.971       0.005 
 
 2WT  14010.0 
     48.064       2.334 
   2267.125     121.398 
      0.955       0.003 
   1247.600      57.974 
      7.060       0.156 
      0.971       0.005 
 
 2WT  15010.0 
     49.171       2.250 
   2329.259     115.194 
      0.957       0.002 
   1290.629      55.203 
      7.069       0.154 
      0.971       0.005 
 
 2WT  16010.0 
     50.414       2.264 
   2392.575     146.237 
      0.958       0.002 
   1335.329      57.105 
      7.078       0.149 
      0.971       0.004 
 
 2WT  17010.0 
     51.542       2.418 
   2455.602      97.818 
      0.960       0.002 
   1376.243      62.732 
      7.082       0.151 
      0.971       0.004 
 
 2WT  18010.0 
     52.415       2.522 
   2468.255     113.416 
      0.962       0.002 
   1410.800      63.050 
      7.084       0.152 
      0.971       0.005 
 
 2WT  19010.0 
     53.466       2.627 
   2515.239     116.666 
      0.963       0.002 
   1450.129      69.522 
      7.092       0.153 
      0.971       0.004 
 
 2WT  20010.0 
     54.445       2.496 
   2561.264     139.697 
      0.964       0.002 
   1486.800      69.669 
      7.095       0.150 
      0.971       0.004 
 
 2WT  21010.0 
     55.299       2.573 
   2594.702     126.669 
      0.965       0.002 
   1519.800      71.799 
      7.097       0.153 
      0.971       0.004 
 
 2WT  22010.0 
     56.270       2.591 
   2644.501     108.493 
      0.966       0.002 
   1556.843      72.667 
      7.101       0.151 
      0.971       0.004 
 
 2WT  23010.0 
     57.192       2.634 
   2675.818     123.303 
      0.967       0.002 
   1589.343      75.104 
      7.104       0.152 
      0.971       0.004 
 
 2WT  24010.0 
     57.972       2.691 
   2695.059     125.151 
      0.968       0.002 
   1620.014      73.362 
      7.106       0.154 
      0.971       0.004 
 
 2WT  25010.0 
     58.741       2.518 
   2721.283     112.497 
      0.969       0.002 
   1650.343      73.165 
      7.108       0.151 
      0.971       0.004 
 
 2WT  26010.0 
     59.604       2.650 
   2768.703     116.712 
      0.970       0.002 
   1682.129      77.454 
      7.112       0.154 
      0.971       0.005 
 
 2WT  27010.0 
     60.464       2.629 
   2802.793     111.153 
      0.971       0.001 
   1713.600      77.359 
      7.115       0.153 
      0.971       0.004 
 
 3KE  10.0 
      1.843       0.117 
     39.240       5.062 
      0.126       0.045 
      9.320       0.248 
      3.178       0.054 
      0.884       0.006 
 
 3KE  1010.0 
     16.805       0.818 
    687.319      60.420 
      0.832       0.008 
    268.600      13.147 
      6.547       0.251 
      0.970       0.009 
 
 3KE  2010.0 
     22.255       0.817 
   1009.438      74.995 
      0.873       0.006 
    414.100      13.776 
      6.749       0.230 
      0.970       0.009 
 
 3KE  3010.0 
     25.888       0.779 
   1231.221      75.391 
      0.894       0.007 
    527.980      23.643 
      6.829       0.246 
      0.970       0.009 
 
 3KE  4010.0 
     28.800       1.099 
   1360.710      92.586 
      0.909       0.006 
    623.580      30.878 
      6.886       0.250 
      0.970       0.009 
 
 3KE  5010.0 
     31.431       1.221 
   1510.219     107.854 
      0.918       0.005 
    713.140      33.464 
      6.926       0.242 
      0.970       0.009 
 
 3KE  6010.0 
     33.510       1.368 
   1588.573     119.094 
      0.927       0.005 
    785.780      39.560 
      6.954       0.244 
      0.970       0.009 
 
 3KE  7010.0 
     35.765       1.293 
   1728.812     121.196 
      0.931       0.005 
    861.940      45.259 
      6.978       0.254 
      0.970       0.009 
 
 3KE  8010.0 
     37.471       1.431 
   1795.251     112.268 
      0.937       0.004 
    924.460      49.561 
      6.993       0.252 
      0.970       0.009 
 
 3KE  9010.0 
     39.467       1.286 
   1899.087      78.999 
      0.941       0.003 
    989.460      50.584 
      7.020       0.247 
      0.971       0.008 
 
 3KE  10010.0 
     40.801       1.476 
   1978.085     120.535 
      0.944       0.003 
   1043.860      54.997 
      7.025       0.254 
      0.970       0.009 
 
 3KE  11010.0 
     42.169       1.730 
   2001.516     115.729 
      0.948       0.003 
   1096.960      60.828 
      7.040       0.257 
      0.971       0.009 
 
 3KE  12010.0 
     43.736       1.627 
   2118.204     121.412 
      0.950       0.003 
   1150.280      62.710 
      7.049       0.256 
      0.971       0.009 
 
 3KE  13010.0 
     44.896       1.504 
   2188.086      95.986 
      0.953       0.003 
   1200.000      66.355 
      7.056       0.257 
      0.970       0.009 
 
 3KE  14010.0 
     46.096       1.786 
   2220.700     134.323 
      0.955       0.003 
   1244.900      71.852 
      7.068       0.260 
      0.971       0.009 
 
 3KE  15010.0 
     47.280       1.856 
   2256.020     118.803 
      0.957       0.003 
   1288.140      72.997 
      7.071       0.259 
      0.971       0.009 
 
 3KE  16010.0 
     48.353       2.078 
   2329.460     125.292 
      0.959       0.003 
   1332.200      78.198 
      7.083       0.258 
      0.971       0.009 
 
 3KE  17010.0 
     49.486       1.935 
   2385.607     131.791 
      0.960       0.002 
   1372.240      75.892 
      7.084       0.256 
      0.971       0.009 
 
 3KE  18010.0 
     50.519       1.936 
   2423.029     141.624 
      0.962       0.002 
   1408.000      78.798 
      7.088       0.259 
      0.971       0.009 
 
 3KE  19010.0 
     51.562       1.986 
   2475.975     123.153 
      0.963       0.002 
   1448.340      79.435 
      7.094       0.258 
      0.971       0.009 
 
 3KE  20010.0 
     52.575       2.089 
   2507.702     137.470 
      0.965       0.002 
   1484.300      86.241 
      7.101       0.259 
      0.971       0.009 
 
 3KE  21010.0 
     53.402       2.076 
   2557.891     145.961 
      0.966       0.002 
   1520.600      86.219 
      7.105       0.254 
      0.971       0.009 
 
 3KE  22010.0 
     53.916       2.025 
   2579.654     140.097 
      0.967       0.002 
   1545.800      86.803 
      7.104       0.254 
      0.971       0.009 
 
 3KE  23010.0 
     55.064       2.045 
   2618.929     116.280 
      0.968       0.002 
   1585.640      88.407 
      7.110       0.259 
      0.971       0.009 
 
 3KE  24010.0 
     55.718       2.065 
   2681.829     145.726 
      0.969       0.002 
   1618.140      89.489 
      7.113       0.258 
      0.971       0.009 
 
 3KE  25010.0 
     56.739       1.985 
   2717.517     146.929 
      0.970       0.002 
   1649.660      90.874 
      7.114       0.258 
      0.971       0.009 
 
 3KE  26010.0 
     57.398       2.165 
   2748.405     158.812 
      0.970       0.002 
   1678.920      96.986 
      7.116       0.261 
      0.971       0.009 
 
 3KE  27010.0 
     58.207       2.104 
   2777.766     150.186 
      0.971       0.002 
   1706.660      95.723 
      7.120       0.259 
      0.971       0.009 
 
 3KO  10.0 
      1.851       0.106 
     34.483       4.693 
      0.157       0.029 
      9.167       0.180 
      3.147       0.043 
      0.881       0.006 
 
 3KO  1010.0 
     17.175       0.619 
    703.450      58.050 
      0.829       0.011 
    273.750      14.377 
      6.642       0.207 
      0.975       0.007 
 
 3KO  2010.0 
     22.971       0.986 
   1025.966      74.107 
      0.870       0.009 
    424.217      21.611 
      6.844       0.199 
      0.976       0.007 
 
 3KO  3010.0 
     26.827       1.042 
   1271.651      91.420 
      0.891       0.007 
    541.133      24.675 
      6.937       0.194 
      0.976       0.007 
 
 3KO  4010.0 
     29.946       1.506 
   1406.265     123.950 
      0.906       0.007 
    640.250      38.305 
      6.988       0.216 
      0.976       0.007 
 
 3KO  5010.0 
     32.811       1.729 
   1538.762     117.575 
      0.916       0.007 
    734.533      44.129 
      7.043       0.217 
      0.976       0.007 
 
 3KO  6010.0 
     35.104       1.863 
   1665.374     103.363 
      0.924       0.005 
    809.467      46.924 
      7.066       0.215 
      0.976       0.007 
 
 3KO  7010.0 
     37.329       1.910 
   1793.598     106.648 
      0.929       0.005 
    882.650      52.325 
      7.081       0.215 
      0.976       0.007 
 
 3KO  8010.0 
     39.192       2.106 
   1893.169     141.458 
      0.935       0.005 
    950.133      59.679 
      7.107       0.220 
      0.976       0.007 
 
 3KO  9010.0 
     40.987       2.304 
   1979.562     141.144 
      0.939       0.005 
   1014.567      64.515 
      7.120       0.223 
      0.976       0.007 
 
 3KO  10010.0 
     42.558       2.219 
   2034.685     112.786 
      0.943       0.004 
   1068.300      59.534 
      7.126       0.215 
      0.976       0.007 
 
 3KO  11010.0 
     44.085       2.253 
   2128.928     119.261 
      0.946       0.003 
   1124.350      66.942 
      7.139       0.224 
      0.976       0.007 
 
 3KO  12010.0 
     45.659       2.572 
   2200.941     139.062 
      0.949       0.004 
   1180.017      73.750 
      7.155       0.220 
      0.976       0.007 
 
 3KO  13010.0 
     46.974       2.651 
   2272.907     165.624 
      0.951       0.004 
   1228.200      79.160 
      7.162       0.220 
      0.976       0.007 
 
 3KO  14010.0 
     48.491       2.776 
   2347.337     141.118 
      0.953       0.003 
   1286.917      79.934 
      7.179       0.221 
      0.976       0.007 
 
 3KO  15010.0 
     49.490       2.816 
   2366.030     157.585 
      0.955       0.003 
   1323.650      84.707 
      7.177       0.223 
      0.976       0.007 
 
 3KO  16010.0 
     50.511       2.888 
   2435.261     128.998 
      0.957       0.003 
   1362.283      84.349 
      7.178       0.222 
      0.976       0.007 
 
 3KO  17010.0 
     51.555       2.700 
   2474.203     163.391 
      0.959       0.003 
   1404.283      88.098 
      7.186       0.224 
      0.976       0.007 
 
 3KO  18010.0 
     52.805       2.920 
   2558.517     168.240 
      0.960       0.003 
   1447.667      87.028 
      7.193       0.222 
      0.976       0.007 
 
 3KO  19010.0 
     53.866       3.007 
   2574.383     153.224 
      0.962       0.003 
   1488.800      95.032 
      7.202       0.224 
      0.976       0.007 
 
 3KO  20010.0 
     54.865       3.150 
   2623.638     161.340 
      0.963       0.003 
   1525.683      95.457 
      7.201       0.221 
      0.976       0.007 
 
 3KO  21010.0 
     55.898       3.117 
   2701.722     170.696 
      0.964       0.003 
   1565.717      99.617 
      7.212       0.223 
      0.976       0.007 
 
 3KO  22010.0 
     56.612       3.216 
   2715.892     175.503 
      0.965       0.002 
   1597.283      98.811 
      7.209       0.225 
      0.976       0.007 
 
 3KO  23010.0 
     57.602       3.245 
   2766.470     178.632 
      0.966       0.002 
   1633.533     103.767 
      7.214       0.223 
      0.976       0.007 
 
 3KO  24010.0 
     58.349       3.189 
   2805.325     166.524 
      0.967       0.002 
   1662.517     103.182 
      7.214       0.225 
      0.976       0.007 
 
 3KO  25010.0 
     59.112       3.316 
   2823.736     187.073 
      0.968       0.002 
   1695.200     105.911 
      7.216       0.224 
      0.976       0.007 
 
 3KO  26010.0 
     60.101       3.386 
   2883.403     203.394 
      0.969       0.002 
   1731.217     110.899 
      7.223       0.227 
      0.976       0.007 
 
 3KO  27010.0 
     60.819       3.336 
   2915.362     177.041 
      0.970       0.002 
   1760.467     106.979 
      7.224       0.224 
      0.976       0.007 
 
 3WE  10.0 
      1.977       0.206 
     34.871       5.631 
      0.164       0.050 
      9.100       0.278 
      3.128       0.062 
      0.878       0.008 
 
 3WE  1010.0 
     17.074       0.821 
    710.512      85.758 
      0.836       0.012 
    264.100      13.609 
      6.638       0.152 
      0.977       0.003 
 
 3WE  2010.0 
     22.426       0.882 
   1001.887      88.690 
      0.876       0.008 
    404.386      20.567 
      6.826       0.161 
      0.977       0.003 
 
 3WE  3010.0 
     26.381       1.123 
   1233.404     102.433 
      0.895       0.008 
    521.043      28.988 
      6.926       0.175 
      0.977       0.004 
 
 3WE  4010.0 
     29.544       1.284 
   1371.055     115.238 
      0.909       0.007 
    617.171      34.838 
      6.969       0.179 
      0.977       0.004 
 
 3WE  5010.0 
     32.084       1.381 
   1512.344     136.662 
      0.918       0.007 
    702.943      40.714 
      7.007       0.172 
      0.977       0.004 
 
 3WE  6010.0 
     34.620       1.367 
   1618.616     112.990 
      0.925       0.006 
    783.957      44.888 
      7.041       0.171 
      0.977       0.004 
 
 3WE  7010.0 
     36.719       1.506 
   1728.800     144.341 
      0.932       0.006 
    852.443      50.410 
      7.064       0.166 
      0.977       0.004 
 
 3WE  8010.0 
     38.485       1.571 
   1822.120     140.292 
      0.936       0.005 
    919.329      54.538 
      7.083       0.168 
      0.977       0.003 
 
 3WE  9010.0 
     40.407       1.765 
   1889.681     171.609 
      0.941       0.005 
    979.057      62.650 
      7.094       0.171 
      0.977       0.003 
 
 3WE  10010.0 
     42.145       1.683 
   1987.547     152.393 
      0.944       0.005 
   1040.800      61.480 
      7.109       0.169 
      0.977       0.004 
 
 3WE  11010.0 
     43.653       1.934 
   2069.335     162.409 
      0.947       0.005 
   1096.314      70.119 
      7.121       0.171 
      0.977       0.003 
 
 3WE  12010.0 
     45.091       2.060 
   2142.656     179.021 
      0.950       0.004 
   1148.100      72.557 
      7.135       0.172 
      0.977       0.003 
 
 3WE  13010.0 
     46.354       2.048 
   2186.252     156.881 
      0.952       0.004 
   1196.414      77.657 
      7.137       0.180 
      0.977       0.004 
 
 3WE  14010.0 
     47.655       2.115 
   2238.656     156.416 
      0.954       0.004 
   1243.000      81.079 
      7.151       0.175 
      0.977       0.003 
 
 3WE  15010.0 
     48.803       2.129 
   2280.647     143.675 
      0.956       0.003 
   1286.071      80.556 
      7.155       0.174 
      0.977       0.003 
 
 3WE  16010.0 
     49.733       2.358 
   2323.020     181.150 
      0.958       0.004 
   1327.229      88.003 
      7.160       0.176 
      0.977       0.003 
 
 3WE  17010.0 
     51.134       2.415 
   2385.298     159.379 
      0.960       0.003 
   1371.086      92.110 
      7.166       0.175 
      0.977       0.003 
 
 3WE  18010.0 
     52.103       2.530 
   2434.722     170.376 
      0.961       0.003 
   1412.514      95.041 
      7.175       0.178 
      0.977       0.003 
 
 3WE  19010.0 
     53.121       2.415 
   2477.352     163.925 
      0.963       0.003 
   1448.714      97.450 
      7.177       0.177 
      0.977       0.003 
 
 3WE  20010.0 
     53.879       2.605 
   2527.323     182.362 
      0.964       0.003 
   1483.486     102.104 
      7.180       0.178 
      0.977       0.004 
 
 3WE  21010.0 
     54.905       2.621 
   2550.180     186.856 
      0.965       0.003 
   1516.929     105.401 
      7.184       0.180 
      0.977       0.004 
 
 3WE  22010.0 
     55.757       2.730 
   2596.696     207.104 
      0.966       0.003 
   1553.729     107.372 
      7.188       0.176 
      0.977       0.003 
 
 3WE  23010.0 
     56.600       2.769 
   2644.912     166.790 
      0.967       0.003 
   1588.557     111.350 
      7.193       0.180 
      0.977       0.004 
 
 3WE  24010.0 
     57.440       2.715 
   2662.144     182.578 
      0.968       0.003 
   1619.814     111.581 
      7.198       0.176 
      0.977       0.003 
 
 3WE  25010.0 
     58.321       2.788 
   2695.776     187.931 
      0.969       0.003 
   1652.729     114.169 
      7.200       0.177 
      0.977       0.003 
 
 3WE  26010.0 
     58.961       2.862 
   2716.174     183.530 
      0.970       0.002 
   1679.014     117.207 
      7.200       0.180 
      0.977       0.004 
 
 3WE  27010.0 
     59.792       2.860 
   2774.418     191.029 
      0.971       0.002 
   1710.543     116.596 
      7.204       0.177 
      0.977       0.003 
 
 3WT  10.0 
      2.121       0.125 
     34.814       6.478 
      0.160       0.058 
      9.143       0.342 
      3.140       0.078 
      0.880       0.010 
 
 3WT  1010.0 
     17.775       0.349 
    658.402      78.521 
      0.839       0.012 
    269.471      11.427 
      6.767       0.189 
      0.979       0.005 
 
 3WT  2010.0 
     23.064       0.423 
    988.800     128.424 
      0.880       0.011 
    405.429      21.991 
      6.947       0.197 
      0.980       0.005 
 
 3WT  3010.0 
     26.971       0.705 
   1185.532     122.776 
      0.900       0.009 
    514.457      30.305 
      7.041       0.198 
      0.980       0.005 
 
 3WT  4010.0 
     30.184       0.842 
   1387.806     187.578 
      0.911       0.010 
    611.100      44.856 
      7.097       0.210 
      0.980       0.005 
 
 3WT  5010.0 
     32.723       1.020 
   1526.205     161.269 
      0.920       0.008 
    695.443      50.629 
      7.130       0.206 
      0.980       0.005 
 
 3WT  6010.0 
     35.147       1.130 
   1666.693     166.547 
      0.927       0.008 
    771.443      57.687 
      7.168       0.211 
      0.980       0.005 
 
 3WT  7010.0 
     37.314       1.247 
   1788.293     183.323 
      0.932       0.008 
    842.386      68.008 
      7.182       0.208 
      0.980       0.005 
 
 3WT  8010.0 
     39.448       1.342 
   1893.954     191.836 
      0.936       0.007 
    909.271      72.045 
      7.199       0.208 
      0.980       0.005 
 
 3WT  9010.0 
     41.073       1.466 
   1952.970     166.931 
      0.941       0.006 
    967.971      79.685 
      7.213       0.212 
      0.980       0.005 
 
 3WT  10010.0 
     42.881       1.665 
   2030.182     187.174 
      0.944       0.006 
   1027.214      87.447 
      7.232       0.214 
      0.980       0.005 
 
 3WT  11010.0 
     44.270       1.650 
   2137.181     194.111 
      0.947       0.006 
   1079.114      93.144 
      7.240       0.209 
      0.980       0.005 
 
 3WT  12010.0 
     45.788       1.887 
   2204.296     189.648 
      0.949       0.006 
   1135.271      97.039 
      7.255       0.212 
      0.980       0.005 
 
 3WT  13010.0 
     47.105       1.995 
   2248.203     179.587 
      0.952       0.005 
   1182.429     104.316 
      7.259       0.216 
      0.980       0.005 
 
 3WT  14010.0 
     48.399       2.130 
   2301.217     171.879 
      0.954       0.005 
   1230.343     109.509 
      7.268       0.215 
      0.980       0.005 
 
 3WT  15010.0 
     49.620       2.277 
   2372.961     175.174 
      0.956       0.005 
   1274.871     118.576 
      7.274       0.214 
      0.980       0.005 
 
 3WT  16010.0 
     50.857       2.058 
   2392.049     181.793 
      0.958       0.004 
   1313.586     114.389 
      7.276       0.212 
      0.980       0.005 
 
 3WT  17010.0 
     52.245       2.303 
   2487.059     175.641 
      0.959       0.004 
   1363.486     121.714 
      7.286       0.219 
      0.980       0.005 
 
 3WT  18010.0 
     53.179       2.364 
   2544.365     174.210 
      0.960       0.004 
   1400.271     125.803 
      7.291       0.213 
      0.980       0.005 
 
 3WT  19010.0 
     54.238       2.359 
   2581.170     162.112 
      0.962       0.004 
   1441.129     127.671 
      7.298       0.215 
      0.980       0.005 
 
 3WT  20010.0 
     55.334       2.466 
   2642.149     164.585 
      0.963       0.004 
   1480.043     134.796 
      7.300       0.219 
      0.980       0.005 
 
 3WT  21010.0 
     56.203       2.445 
   2667.752     139.146 
      0.964       0.003 
   1514.814     131.788 
      7.303       0.219 
      0.980       0.005 
 
 3WT  22010.0 
     57.085       2.587 
   2705.876     172.135 
      0.965       0.003 
   1548.543     140.813 
      7.306       0.217 
      0.980       0.005 
 
 3WT  23010.0 
     58.066       2.581 
   2766.205     144.248 
      0.966       0.003 
   1586.443     141.959 
      7.312       0.217 
      0.980       0.005 
 
 3WT  24010.0 
     59.083       2.774 
   2784.735     166.827 
      0.967       0.003 
   1620.257     145.924 
      7.314       0.217 
      0.980       0.005 
 
 3WT  25010.0 
     59.824       2.641 
   2811.104     152.546 
      0.968       0.003 
   1651.229     145.236 
      7.319       0.218 
      0.980       0.005 
 
 3WT  26010.0 
     60.522       2.634 
   2851.938     142.165 
      0.969       0.002 
   1680.043     148.843 
      7.320       0.217 
      0.980       0.005 
 
 3WT  27010.0 
     61.269       2.682 
   2875.585     141.381 
      0.970       0.002 
   1709.386     149.569 
      7.322       0.216 
      0.980       0.005 
 
  LinkerPrimerSequence  Seqs/Sample 
 PD_whole_tree Ave.  PD_whole_tree Err. 
 chao1 Ave.  chao1 Err. 
 goods_coverage Ave.  goods_coverage Err. 
 observed_species Ave.  observed_species Err. 
 shannon Ave.  shannon Err. 
 simpson Ave.  simpson Err. 
 
 
 AAAAAA  10.0 
      1.962       0.183 
     33.541       6.641 
      0.181       0.066 
      9.011       0.386 
      3.110       0.088 
      0.876       0.011 
 
 AAAAAA  1010.0 
     17.311       0.711 
    670.876      66.024 
      0.835       0.012 
    268.859      13.916 
      6.574       0.215 
      0.972       0.008 
 
 AAAAAA  2010.0 
     22.771       0.857 
    981.612      87.551 
      0.877       0.009 
    409.649      21.997 
      6.765       0.221 
      0.972       0.009 
 
 AAAAAA  3010.0 
     26.732       1.029 
   1196.706     101.064 
      0.897       0.008 
    522.092      31.120 
      6.855       0.226 
      0.972       0.009 
 
 AAAAAA  4010.0 
     29.876       1.208 
   1368.031     116.898 
      0.910       0.007 
    617.432      38.740 
      6.908       0.231 
      0.972       0.009 
 
 AAAAAA  5010.0 
     32.617       1.348 
   1512.155     115.003 
      0.919       0.006 
    703.632      44.064 
      6.946       0.229 
      0.972       0.009 
 
 AAAAAA  6010.0 
     34.996       1.451 
   1630.832     113.701 
      0.927       0.006 
    779.999      49.777 
      6.977       0.230 
      0.973       0.008 
 
 AAAAAA  7010.0 
     37.136       1.529 
   1743.078     123.939 
      0.932       0.005 
    849.487      55.251 
      6.997       0.232 
      0.973       0.008 
 
 AAAAAA  8010.0 
     39.082       1.659 
   1846.744     138.849 
      0.937       0.005 
    915.556      59.140 
      7.016       0.231 
      0.973       0.008 
 
 AAAAAA  9010.0 
     40.912       1.728 
   1937.066     132.205 
      0.941       0.004 
    977.139      64.095 
      7.033       0.232 
      0.973       0.008 
 
 AAAAAA  10010.0 
     42.555       1.792 
   2015.583     135.985 
      0.944       0.004 
   1034.351      66.788 
      7.044       0.234 
      0.973       0.009 
 
 AAAAAA  11010.0 
     44.087       1.864 
   2092.906     133.985 
      0.947       0.004 
   1088.283      71.028 
      7.056       0.233 
      0.973       0.009 
 
 AAAAAA  12010.0 
     45.564       1.994 
   2168.774     143.833 
      0.950       0.004 
   1141.018      74.627 
      7.068       0.234 
      0.973       0.009 
 
 AAAAAA  13010.0 
     46.901       2.051 
   2227.515     141.830 
      0.952       0.004 
   1188.466      79.401 
      7.073       0.234 
      0.973       0.009 
 
 AAAAAA  14010.0 
     48.187       2.139 
   2286.145     138.010 
      0.954       0.003 
   1236.569      82.198 
      7.082       0.236 
      0.973       0.009 
 
 AAAAAA  15010.0 
     49.418       2.188 
   2338.909     143.128 
      0.956       0.003 
   1280.524      85.312 
      7.089       0.235 
      0.973       0.008 
 
 AAAAAA  16010.0 
     50.543       2.254 
   2389.034     145.626 
      0.958       0.003 
   1322.089      86.340 
      7.094       0.233 
      0.973       0.008 
 
 AAAAAA  17010.0 
     51.776       2.321 
   2457.541     143.530 
      0.959       0.003 
   1365.858      90.059 
      7.101       0.235 
      0.973       0.009 
 
 AAAAAA  18010.0 
     52.770       2.333 
   2495.853     148.676 
      0.961       0.003 
   1403.604      92.087 
      7.105       0.235 
      0.973       0.008 
 
 AAAAAA  19010.0 
     53.825       2.404 
   2537.045     143.894 
      0.962       0.003 
   1442.779      95.281 
      7.111       0.237 
      0.973       0.008 
 
 AAAAAA  20010.0 
     54.830       2.486 
   2583.306     153.057 
      0.964       0.003 
   1480.015      97.973 
      7.115       0.236 
      0.973       0.008 
 
 AAAAAA  21010.0 
     55.752       2.501 
   2629.331     150.701 
      0.965       0.002 
   1515.876      99.636 
      7.119       0.236 
      0.973       0.008 
 
 AAAAAA  22010.0 
     56.612       2.594 
   2668.251     158.103 
      0.966       0.002 
   1549.849     102.540 
      7.121       0.236 
      0.973       0.009 
 
 AAAAAA  23010.0 
     57.526       2.602 
   2703.284     148.831 
      0.967       0.002 
   1583.948     104.823 
      7.125       0.237 
      0.973       0.008 
 
 AAAAAA  24010.0 
     58.386       2.630 
   2744.243     152.354 
      0.968       0.002 
   1616.894     106.391 
      7.128       0.237 
      0.973       0.009 
 
 AAAAAA  25010.0 
     59.224       2.635 
   2774.659     152.451 
      0.969       0.002 
   1648.544     106.942 
      7.131       0.237 
      0.973       0.008 
 
 AAAAAA  26010.0 
     60.003       2.684 
   2809.695     156.004 
      0.970       0.002 
   1679.494     109.897 
      7.134       0.237 
      0.973       0.009 
 
 AAAAAA  27010.0 
     60.774       2.696 
   2846.963     155.919 
      0.970       0.002 
   1708.780     110.592 
      7.137       0.236 
      0.973       0.008 
 
  BarcodeSequence  Seqs/Sample 
 PD_whole_tree Ave.  PD_whole_tree Err. 
 chao1 Ave.  chao1 Err. 
 goods_coverage Ave.  goods_coverage Err. 
 observed_species Ave.  observed_species Err. 
 shannon Ave.  shannon Err. 
 simpson Ave.  simpson Err. 
 
 
 ATCGA  10.0 
      1.962       0.183 
     33.541       6.641 
      0.181       0.066 
      9.011       0.386 
      3.110       0.088 
      0.876       0.011 
 
 ATCGA  1010.0 
     17.311       0.711 
    670.876      66.024 
      0.835       0.012 
    268.859      13.916 
      6.574       0.215 
      0.972       0.008 
 
 ATCGA  2010.0 
     22.771       0.857 
    981.612      87.551 
      0.877       0.009 
    409.649      21.997 
      6.765       0.221 
      0.972       0.009 
 
 ATCGA  3010.0 
     26.732       1.029 
   1196.706     101.064 
      0.897       0.008 
    522.092      31.120 
      6.855       0.226 
      0.972       0.009 
 
 ATCGA  4010.0 
     29.876       1.208 
   1368.031     116.898 
      0.910       0.007 
    617.432      38.740 
      6.908       0.231 
      0.972       0.009 
 
 ATCGA  5010.0 
     32.617       1.348 
   1512.155     115.003 
      0.919       0.006 
    703.632      44.064 
      6.946       0.229 
      0.972       0.009 
 
 ATCGA  6010.0 
     34.996       1.451 
   1630.832     113.701 
      0.927       0.006 
    779.999      49.777 
      6.977       0.230 
      0.973       0.008 
 
 ATCGA  7010.0 
     37.136       1.529 
   1743.078     123.939 
      0.932       0.005 
    849.487      55.251 
      6.997       0.232 
      0.973       0.008 
 
 ATCGA  8010.0 
     39.082       1.659 
   1846.744     138.849 
      0.937       0.005 
    915.556      59.140 
      7.016       0.231 
      0.973       0.008 
 
 ATCGA  9010.0 
     40.912       1.728 
   1937.066     132.205 
      0.941       0.004 
    977.139      64.095 
      7.033       0.232 
      0.973       0.008 
 
 ATCGA  10010.0 
     42.555       1.792 
   2015.583     135.985 
      0.944       0.004 
   1034.351      66.788 
      7.044       0.234 
      0.973       0.009 
 
 ATCGA  11010.0 
     44.087       1.864 
   2092.906     133.985 
      0.947       0.004 
   1088.283      71.028 
      7.056       0.233 
      0.973       0.009 
 
 ATCGA  12010.0 
     45.564       1.994 
   2168.774     143.833 
      0.950       0.004 
   1141.018      74.627 
      7.068       0.234 
      0.973       0.009 
 
 ATCGA  13010.0 
     46.901       2.051 
   2227.515     141.830 
      0.952       0.004 
   1188.466      79.401 
      7.073       0.234 
      0.973       0.009 
 
 ATCGA  14010.0 
     48.187       2.139 
   2286.145     138.010 
      0.954       0.003 
   1236.569      82.198 
      7.082       0.236 
      0.973       0.009 
 
 ATCGA  15010.0 
     49.418       2.188 
   2338.909     143.128 
      0.956       0.003 
   1280.524      85.312 
      7.089       0.235 
      0.973       0.008 
 
 ATCGA  16010.0 
     50.543       2.254 
   2389.034     145.626 
      0.958       0.003 
   1322.089      86.340 
      7.094       0.233 
      0.973       0.008 
 
 ATCGA  17010.0 
     51.776       2.321 
   2457.541     143.530 
      0.959       0.003 
   1365.858      90.059 
      7.101       0.235 
      0.973       0.009 
 
 ATCGA  18010.0 
     52.770       2.333 
   2495.853     148.676 
      0.961       0.003 
   1403.604      92.087 
      7.105       0.235 
      0.973       0.008 
 
 ATCGA  19010.0 
     53.825       2.404 
   2537.045     143.894 
      0.962       0.003 
   1442.779      95.281 
      7.111       0.237 
      0.973       0.008 
 
 ATCGA  20010.0 
     54.830       2.486 
   2583.306     153.057 
      0.964       0.003 
   1480.015      97.973 
      7.115       0.236 
      0.973       0.008 
 
 ATCGA  21010.0 
     55.752       2.501 
   2629.331     150.701 
      0.965       0.002 
   1515.876      99.636 
      7.119       0.236 
      0.973       0.008 
 
 ATCGA  22010.0 
     56.612       2.594 
   2668.251     158.103 
      0.966       0.002 
   1549.849     102.540 
      7.121       0.236 
      0.973       0.009 
 
 ATCGA  23010.0 
     57.526       2.602 
   2703.284     148.831 
      0.967       0.002 
   1583.948     104.823 
      7.125       0.237 
      0.973       0.008 
 
 ATCGA  24010.0 
     58.386       2.630 
   2744.243     152.354 
      0.968       0.002 
   1616.894     106.391 
      7.128       0.237 
      0.973       0.009 
 
 ATCGA  25010.0 
     59.224       2.635 
   2774.659     152.451 
      0.969       0.002 
   1648.544     106.942 
      7.131       0.237 
      0.973       0.008 
 
 ATCGA  26010.0 
     60.003       2.684 
   2809.695     156.004 
      0.970       0.002 
   1679.494     109.897 
      7.134       0.237 
      0.973       0.009 
 
 ATCGA  27010.0 
     60.774       2.696 
   2846.963     155.919 
      0.970       0.002 
   1708.780     110.592 
      7.137       0.236 
      0.973       0.008 
 
  Description  Seqs/Sample 
 PD_whole_tree Ave.  PD_whole_tree Err. 
 chao1 Ave.  chao1 Err. 
 goods_coverage Ave.  goods_coverage Err. 
 observed_species Ave.  observed_species Err. 
 shannon Ave.  shannon Err. 
 simpson Ave.  simpson Err. 
 
 
 s1KE1  10.0 
      1.965         nan 
     27.600         nan 
      0.240         nan 
      8.700         nan 
      3.047         nan 
      0.870         nan 
 
 s1KE1  1010.0 
     16.882         nan 
    649.637         nan 
      0.838         nan 
    256.200         nan 
      6.162         nan 
      0.956         nan 
 
 s1KE1  2010.0 
     21.857         nan 
    954.259         nan 
      0.881         nan 
    389.400         nan 
      6.348         nan 
      0.956         nan 
 
 s1KE1  3010.0 
     26.076         nan 
   1117.921         nan 
      0.902         nan 
    494.200         nan 
      6.428         nan 
      0.957         nan 
 
 s1KE1  4010.0 
     29.232         nan 
   1326.428         nan 
      0.912         nan 
    591.000         nan 
      6.469         nan 
      0.956         nan 
 
 s1KE1  5010.0 
     31.812         nan 
   1374.237         nan 
      0.922         nan 
    673.500         nan 
      6.505         nan 
      0.956         nan 
 
 s1KE1  6010.0 
     34.546         nan 
   1580.933         nan 
      0.928         nan 
    749.900         nan 
      6.531         nan 
      0.956         nan 
 
 s1KE1  7010.0 
     36.194         nan 
   1661.565         nan 
      0.934         nan 
    815.200         nan 
      6.565         nan 
      0.957         nan 
 
 s1KE1  8010.0 
     38.927         nan 
   1731.085         nan 
      0.939         nan 
    889.400         nan 
      6.593         nan 
      0.957         nan 
 
 s1KE1  9010.0 
     40.560         nan 
   1879.658         nan 
      0.942         nan 
    943.200         nan 
      6.589         nan 
      0.956         nan 
 
 s1KE1  10010.0 
     42.123         nan 
   1954.242         nan 
      0.945         nan 
   1003.000         nan 
      6.610         nan 
      0.957         nan 
 
 s1KE1  11010.0 
     43.931         nan 
   1958.828         nan 
      0.949         nan 
   1051.800         nan 
      6.628         nan 
      0.957         nan 
 
 s1KE1  12010.0 
     44.686         nan 
   2036.673         nan 
      0.952         nan 
   1095.600         nan 
      6.615         nan 
      0.956         nan 
 
 s1KE1  13010.0 
     46.473         nan 
   2114.152         nan 
      0.954         nan 
   1148.400         nan 
      6.641         nan 
      0.957         nan 
 
 s1KE1  14010.0 
     47.209         nan 
   2219.424         nan 
      0.956         nan 
   1190.900         nan 
      6.633         nan 
      0.956         nan 
 
 s1KE1  15010.0 
     48.916         nan 
   2281.013         nan 
      0.958         nan 
   1239.300         nan 
      6.649         nan 
      0.957         nan 
 
 s1KE1  16010.0 
     49.842         nan 
   2326.964         nan 
      0.959         nan 
   1277.600         nan 
      6.662         nan 
      0.957         nan 
 
 s1KE1  17010.0 
     51.178         nan 
   2425.192         nan 
      0.960         nan 
   1320.100         nan 
      6.665         nan 
      0.957         nan 
 
 s1KE1  18010.0 
     51.859         nan 
   2426.872         nan 
      0.962         nan 
   1357.600         nan 
      6.670         nan 
      0.957         nan 
 
 s1KE1  19010.0 
     52.873         nan 
   2532.451         nan 
      0.963         nan 
   1394.300         nan 
      6.658         nan 
      0.956         nan 
 
 s1KE1  20010.0 
     54.158         nan 
   2522.400         nan 
      0.965         nan 
   1420.800         nan 
      6.664         nan 
      0.957         nan 
 
 s1KE1  21010.0 
     54.750         nan 
   2624.935         nan 
      0.966         nan 
   1462.300         nan 
      6.678         nan 
      0.957         nan 
 
 s1KE1  22010.0 
     55.840         nan 
   2663.347         nan 
      0.967         nan 
   1492.700         nan 
      6.675         nan 
      0.956         nan 
 
 s1KE1  23010.0 
     56.800         nan 
   2621.788         nan 
      0.968         nan 
   1524.700         nan 
      6.685         nan 
      0.957         nan 
 
 s1KE1  24010.0 
     57.556         nan 
   2678.945         nan 
      0.969         nan 
   1558.300         nan 
      6.686         nan 
      0.957         nan 
 
 s1KE1  25010.0 
     58.256         nan 
   2721.047         nan 
      0.970         nan 
   1586.500         nan 
      6.684         nan 
      0.957         nan 
 
 s1KE1  26010.0 
     59.191         nan 
   2753.627         nan 
      0.970         nan 
   1620.500         nan 
      6.698         nan 
      0.957         nan 
 
 s1KE1  27010.0 
     59.847         nan 
   2857.651         nan 
      0.971         nan 
   1651.300         nan 
      6.696         nan 
      0.957         nan 
 
 s1KE2  10.0 
      1.985         nan 
     35.400         nan 
      0.150         nan 
      9.200         nan 
      3.154         nan 
      0.882         nan 
 
 s1KE2  1010.0 
     18.831         nan 
    813.425         nan 
      0.801         nan 
    304.200         nan 
      6.753         nan 
      0.974         nan 
 
 s1KE2  2010.0 
     24.808         nan 
   1085.877         nan 
      0.857         nan 
    465.300         nan 
      6.959         nan 
      0.975         nan 
 
 s1KE2  3010.0 
     29.282         nan 
   1371.172         nan 
      0.877         nan 
    610.200         nan 
      7.089         nan 
      0.975         nan 
 
 s1KE2  4010.0 
     33.179         nan 
   1610.334         nan 
      0.893         nan 
    724.800         nan 
      7.138         nan 
      0.974         nan 
 
 s1KE2  5010.0 
     36.044         nan 
   1676.857         nan 
      0.906         nan 
    816.700         nan 
      7.177         nan 
      0.975         nan 
 
 s1KE2  6010.0 
     38.913         nan 
   1861.937         nan 
      0.914         nan 
    908.200         nan 
      7.218         nan 
      0.975         nan 
 
 s1KE2  7010.0 
     41.087         nan 
   1895.475         nan 
      0.923         nan 
    982.300         nan 
      7.242         nan 
      0.975         nan 
 
 s1KE2  8010.0 
     43.225         nan 
   2129.675         nan 
      0.927         nan 
   1064.300         nan 
      7.264         nan 
      0.975         nan 
 
 s1KE2  9010.0 
     44.725         nan 
   2068.605         nan 
      0.934         nan 
   1117.400         nan 
      7.271         nan 
      0.975         nan 
 
 s1KE2  10010.0 
     46.858         nan 
   2155.122         nan 
      0.938         nan 
   1194.000         nan 
      7.299         nan 
      0.975         nan 
 
 s1KE2  11010.0 
     48.539         nan 
   2285.545         nan 
      0.941         nan 
   1249.200         nan 
      7.297         nan 
      0.975         nan 
 
 s1KE2  12010.0 
     50.460         nan 
   2370.817         nan 
      0.944         nan 
   1313.500         nan 
      7.321         nan 
      0.975         nan 
 
 s1KE2  13010.0 
     51.673         nan 
   2481.023         nan 
      0.946         nan 
   1365.800         nan 
      7.312         nan 
      0.974         nan 
 
 s1KE2  14010.0 
     53.588         nan 
   2545.178         nan 
      0.949         nan 
   1424.200         nan 
      7.334         nan 
      0.975         nan 
 
 s1KE2  15010.0 
     54.531         nan 
   2620.585         nan 
      0.951         nan 
   1467.600         nan 
      7.336         nan 
      0.975         nan 
 
 s1KE2  16010.0 
     56.109         nan 
   2624.855         nan 
      0.953         nan 
   1517.500         nan 
      7.344         nan 
      0.975         nan 
 
 s1KE2  17010.0 
     57.046         nan 
   2711.870         nan 
      0.956         nan 
   1552.300         nan 
      7.356         nan 
      0.975         nan 
 
 s1KE2  18010.0 
     57.950         nan 
   2721.603         nan 
      0.958         nan 
   1594.800         nan 
      7.345         nan 
      0.974         nan 
 
 s1KE2  19010.0 
     59.491         nan 
   2760.603         nan 
      0.959         nan 
   1640.000         nan 
      7.367         nan 
      0.975         nan 
 
 s1KE2  20010.0 
     60.372         nan 
   2862.534         nan 
      0.960         nan 
   1682.000         nan 
      7.367         nan 
      0.975         nan 
 
 s1KE2  21010.0 
     61.570         nan 
   2919.968         nan 
      0.961         nan 
   1722.500         nan 
      7.374         nan 
      0.975         nan 
 
 s1KE2  22010.0 
     62.325         nan 
   2894.012         nan 
      0.963         nan 
   1757.600         nan 
      7.370         nan 
      0.975         nan 
 
 s1KE2  23010.0 
     63.292         nan 
   2983.440         nan 
      0.964         nan 
   1792.900         nan 
      7.373         nan 
      0.975         nan 
 
 s1KE2  24010.0 
     64.291         nan 
   3058.823         nan 
      0.965         nan 
   1841.800         nan 
      7.388         nan 
      0.975         nan 
 
 s1KE2  25010.0 
     65.152         nan 
   3026.562         nan 
      0.966         nan 
   1859.200         nan 
      7.384         nan 
      0.975         nan 
 
 s1KE2  26010.0 
     66.124         nan 
   3097.823         nan 
      0.967         nan 
   1899.300         nan 
      7.392         nan 
      0.975         nan 
 
 s1KE2  27010.0 
     66.849         nan 
   3148.352         nan 
      0.968         nan 
   1936.800         nan 
      7.395         nan 
      0.975         nan 
 
 s1KE3  10.0 
      1.963         nan 
     30.600         nan 
      0.180         nan 
      9.100         nan 
      3.142         nan 
      0.882         nan 
 
 s1KE3  1010.0 
     18.400         nan 
    804.554         nan 
      0.814         nan 
    285.700         nan 
      6.683         nan 
      0.976         nan 
 
 s1KE3  2010.0 
     24.400         nan 
   1089.535         nan 
      0.863         nan 
    434.500         nan 
      6.866         nan 
      0.977         nan 
 
 s1KE3  3010.0 
     28.934         nan 
   1397.337         nan 
      0.881         nan 
    569.500         nan 
      6.995         nan 
      0.978         nan 
 
 s1KE3  4010.0 
     32.798         nan 
   1579.305         nan 
      0.895         nan 
    683.900         nan 
      7.044         nan 
      0.977         nan 
 
 s1KE3  5010.0 
     35.392         nan 
   1654.409         nan 
      0.909         nan 
    769.800         nan 
      7.088         nan 
      0.977         nan 
 
 s1KE3  6010.0 
     38.345         nan 
   1824.859         nan 
      0.916         nan 
    867.600         nan 
      7.129         nan 
      0.978         nan 
 
 s1KE3  7010.0 
     40.475         nan 
   1970.567         nan 
      0.923         nan 
    939.500         nan 
      7.148         nan 
      0.978         nan 
 
 s1KE3  8010.0 
     43.317         nan 
   2054.539         nan 
      0.928         nan 
   1021.800         nan 
      7.181         nan 
      0.978         nan 
 
 s1KE3  9010.0 
     45.093         nan 
   2185.388         nan 
      0.932         nan 
   1091.700         nan 
      7.206         nan 
      0.978         nan 
 
 s1KE3  10010.0 
     46.955         nan 
   2242.889         nan 
      0.936         nan 
   1157.900         nan 
      7.205         nan 
      0.978         nan 
 
 s1KE3  11010.0 
     48.153         nan 
   2305.080         nan 
      0.940         nan 
   1210.200         nan 
      7.212         nan 
      0.978         nan 
 
 s1KE3  12010.0 
     49.881         nan 
   2409.703         nan 
      0.943         nan 
   1276.300         nan 
      7.232         nan 
      0.978         nan 
 
 s1KE3  13010.0 
     51.454         nan 
   2444.117         nan 
      0.946         nan 
   1330.200         nan 
      7.238         nan 
      0.978         nan 
 
 s1KE3  14010.0 
     52.450         nan 
   2446.445         nan 
      0.949         nan 
   1366.900         nan 
      7.242         nan 
      0.978         nan 
 
 s1KE3  15010.0 
     54.113         nan 
   2612.023         nan 
      0.951         nan 
   1429.600         nan 
      7.247         nan 
      0.978         nan 
 
 s1KE3  16010.0 
     55.371         nan 
   2651.041         nan 
      0.953         nan 
   1472.600         nan 
      7.245         nan 
      0.978         nan 
 
 s1KE3  17010.0 
     56.771         nan 
   2696.724         nan 
      0.954         nan 
   1528.300         nan 
      7.265         nan 
      0.978         nan 
 
 s1KE3  18010.0 
     57.710         nan 
   2712.938         nan 
      0.956         nan 
   1568.700         nan 
      7.270         nan 
      0.978         nan 
 
 s1KE3  19010.0 
     58.557         nan 
   2765.380         nan 
      0.958         nan 
   1606.300         nan 
      7.271         nan 
      0.978         nan 
 
 s1KE3  20010.0 
     60.105         nan 
   2856.291         nan 
      0.959         nan 
   1660.700         nan 
      7.278         nan 
      0.978         nan 
 
 s1KE3  21010.0 
     60.798         nan 
   2867.342         nan 
      0.961         nan 
   1692.300         nan 
      7.282         nan 
      0.978         nan 
 
 s1KE3  22010.0 
     62.119         nan 
   2929.725         nan 
      0.961         nan 
   1747.300         nan 
      7.298         nan 
      0.978         nan 
 
 s1KE3  23010.0 
     63.352         nan 
   2967.566         nan 
      0.962         nan 
   1783.100         nan 
      7.294         nan 
      0.978         nan 
 
 s1KE3  24010.0 
     63.852         nan 
   3030.330         nan 
      0.964         nan 
   1815.900         nan 
      7.301         nan 
      0.978         nan 
 
 s1KE3  25010.0 
     64.651         nan 
   2995.921         nan 
      0.965         nan 
   1843.500         nan 
      7.295         nan 
      0.978         nan 
 
 s1KE3  26010.0 
     65.747         nan 
   3083.744         nan 
      0.966         nan 
   1886.700         nan 
      7.301         nan 
      0.978         nan 
 
 s1KE3  27010.0 
     66.282         nan 
   3127.122         nan 
      0.967         nan 
   1914.200         nan 
      7.302         nan 
      0.978         nan 
 
 s1KE4  10.0 
      1.707         nan 
     35.900         nan 
      0.210         nan 
      8.800         nan 
      3.059         nan 
      0.870         nan 
 
 s1KE4  1010.0 
     16.857         nan 
    698.867         nan 
      0.833         nan 
    261.500         nan 
      6.381         nan 
      0.970         nan 
 
 s1KE4  2010.0 
     23.050         nan 
   1028.133         nan 
      0.873         nan 
    410.200         nan 
      6.613         nan 
      0.972         nan 
 
 s1KE4  3010.0 
     27.030         nan 
   1225.797         nan 
      0.895         nan 
    519.900         nan 
      6.688         nan 
      0.972         nan 
 
 s1KE4  4010.0 
     30.030         nan 
   1395.968         nan 
      0.908         nan 
    620.900         nan 
      6.741         nan 
      0.972         nan 
 
 s1KE4  5010.0 
     32.465         nan 
   1493.542         nan 
      0.920         nan 
    700.400         nan 
      6.791         nan 
      0.972         nan 
 
 s1KE4  6010.0 
     35.243         nan 
   1587.415         nan 
      0.927         nan 
    775.500         nan 
      6.808         nan 
      0.972         nan 
 
 s1KE4  7010.0 
     37.686         nan 
   1777.069         nan 
      0.931         nan 
    855.700         nan 
      6.837         nan 
      0.972         nan 
 
 s1KE4  8010.0 
     39.038         nan 
   1821.682         nan 
      0.938         nan 
    900.200         nan 
      6.846         nan 
      0.972         nan 
 
 s1KE4  9010.0 
     41.431         nan 
   1933.663         nan 
      0.941         nan 
    973.500         nan 
      6.868         nan 
      0.972         nan 
 
 s1KE4  10010.0 
     42.924         nan 
   2076.453         nan 
      0.944         nan 
   1032.700         nan 
      6.871         nan 
      0.972         nan 
 
 s1KE4  11010.0 
     44.717         nan 
   2029.723         nan 
      0.948         nan 
   1082.300         nan 
      6.907         nan 
      0.972         nan 
 
 s1KE4  12010.0 
     45.841         nan 
   2089.619         nan 
      0.951         nan 
   1126.400         nan 
      6.887         nan 
      0.972         nan 
 
 s1KE4  13010.0 
     47.775         nan 
   2211.179         nan 
      0.952         nan 
   1188.400         nan 
      6.913         nan 
      0.972         nan 
 
 s1KE4  14010.0 
     48.975         nan 
   2279.719         nan 
      0.954         nan 
   1237.100         nan 
      6.913         nan 
      0.972         nan 
 
 s1KE4  15010.0 
     49.649         nan 
   2386.029         nan 
      0.956         nan 
   1267.300         nan 
      6.924         nan 
      0.972         nan 
 
 s1KE4  16010.0 
     51.414         nan 
   2396.242         nan 
      0.958         nan 
   1323.200         nan 
      6.929         nan 
      0.972         nan 
 
 s1KE4  17010.0 
     52.931         nan 
   2490.106         nan 
      0.959         nan 
   1364.100         nan 
      6.924         nan 
      0.972         nan 
 
 s1KE4  18010.0 
     53.794         nan 
   2492.701         nan 
      0.961         nan 
   1399.700         nan 
      6.929         nan 
      0.972         nan 
 
 s1KE4  19010.0 
     54.476         nan 
   2495.129         nan 
      0.963         nan 
   1439.400         nan 
      6.944         nan 
      0.972         nan 
 
 s1KE4  20010.0 
     55.787         nan 
   2606.120         nan 
      0.963         nan 
   1487.600         nan 
      6.956         nan 
      0.972         nan 
 
 s1KE4  21010.0 
     56.600         nan 
   2623.357         nan 
      0.965         nan 
   1517.700         nan 
      6.950         nan 
      0.972         nan 
 
 s1KE4  22010.0 
     57.001         nan 
   2587.208         nan 
      0.967         nan 
   1533.200         nan 
      6.956         nan 
      0.972         nan 
 
 s1KE4  23010.0 
     58.323         nan 
   2625.923         nan 
      0.967         nan 
   1573.900         nan 
      6.956         nan 
      0.972         nan 
 
 s1KE4  24010.0 
     59.146         nan 
   2674.785         nan 
      0.968         nan 
   1605.500         nan 
      6.959         nan 
      0.972         nan 
 
 s1KE4  25010.0 
     60.302         nan 
   2735.651         nan 
      0.969         nan 
   1644.900         nan 
      6.964         nan 
      0.972         nan 
 
 s1KE4  26010.0 
     60.971         nan 
   2848.447         nan 
      0.969         nan 
   1675.200         nan 
      6.968         nan 
      0.972         nan 
 
 s1KE4  27010.0 
     61.814         nan 
   2840.630         nan 
      0.970         nan 
   1707.400         nan 
      6.973         nan 
      0.972         nan 
 
 s1KE5  10.0 
      1.726         nan 
     25.800         nan 
      0.270         nan 
      8.400         nan 
      2.939         nan 
      0.846         nan 
 
 s1KE5  1010.0 
     17.598         nan 
    632.888         nan 
      0.836         nan 
    268.800         nan 
      6.309         nan 
      0.956         nan 
 
 s1KE5  2010.0 
     22.892         nan 
    982.528         nan 
      0.879         nan 
    403.000         nan 
      6.452         nan 
      0.955         nan 
 
 s1KE5  3010.0 
     26.767         nan 
   1132.282         nan 
      0.901         nan 
    509.100         nan 
      6.522         nan 
      0.954         nan 
 
 s1KE5  4010.0 
     29.999         nan 
   1390.379         nan 
      0.911         nan 
    609.500         nan 
      6.590         nan 
      0.955         nan 
 
 s1KE5  5010.0 
     32.631         nan 
   1494.729         nan 
      0.921         nan 
    687.700         nan 
      6.627         nan 
      0.955         nan 
 
 s1KE5  6010.0 
     35.038         nan 
   1630.125         nan 
      0.927         nan 
    768.400         nan 
      6.666         nan 
      0.956         nan 
 
 s1KE5  7010.0 
     37.416         nan 
   1783.132         nan 
      0.932         nan 
    844.300         nan 
      6.703         nan 
      0.956         nan 
 
 s1KE5  8010.0 
     39.236         nan 
   1881.189         nan 
      0.937         nan 
    905.800         nan 
      6.711         nan 
      0.956         nan 
 
 s1KE5  9010.0 
     41.370         nan 
   1985.130         nan 
      0.939         nan 
    980.900         nan 
      6.739         nan 
      0.956         nan 
 
 s1KE5  10010.0 
     42.601         nan 
   2025.225         nan 
      0.944         nan 
   1023.800         nan 
      6.733         nan 
      0.956         nan 
 
 s1KE5  11010.0 
     44.054         nan 
   2081.500         nan 
      0.947         nan 
   1075.000         nan 
      6.735         nan 
      0.956         nan 
 
 s1KE5  12010.0 
     45.585         nan 
   2187.003         nan 
      0.949         nan 
   1125.800         nan 
      6.744         nan 
      0.956         nan 
 
 s1KE5  13010.0 
     47.222         nan 
   2267.507         nan 
      0.952         nan 
   1182.500         nan 
      6.762         nan 
      0.956         nan 
 
 s1KE5  14010.0 
     48.402         nan 
   2332.422         nan 
      0.954         nan 
   1225.700         nan 
      6.766         nan 
      0.956         nan 
 
 s1KE5  15010.0 
     49.218         nan 
   2318.536         nan 
      0.957         nan 
   1266.300         nan 
      6.780         nan 
      0.956         nan 
 
 s1KE5  16010.0 
     51.219         nan 
   2436.085         nan 
      0.957         nan 
   1320.300         nan 
      6.780         nan 
      0.956         nan 
 
 s1KE5  17010.0 
     52.014         nan 
   2482.403         nan 
      0.959         nan 
   1359.200         nan 
      6.788         nan 
      0.956         nan 
 
 s1KE5  18010.0 
     52.994         nan 
   2525.788         nan 
      0.961         nan 
   1392.100         nan 
      6.793         nan 
      0.956         nan 
 
 s1KE5  19010.0 
     54.444         nan 
   2597.365         nan 
      0.962         nan 
   1441.700         nan 
      6.799         nan 
      0.956         nan 
 
 s1KE5  20010.0 
     55.456         nan 
   2680.207         nan 
      0.963         nan 
   1481.500         nan 
      6.804         nan 
      0.956         nan 
 
 s1KE5  21010.0 
     56.031         nan 
   2642.305         nan 
      0.964         nan 
   1507.300         nan 
      6.805         nan 
      0.956         nan 
 
 s1KE5  22010.0 
     56.944         nan 
   2723.072         nan 
      0.965         nan 
   1551.300         nan 
      6.807         nan 
      0.956         nan 
 
 s1KE5  23010.0 
     58.134         nan 
   2798.344         nan 
      0.966         nan 
   1582.800         nan 
      6.814         nan 
      0.956         nan 
 
 s1KE5  24010.0 
     58.632         nan 
   2747.828         nan 
      0.968         nan 
   1607.700         nan 
      6.812         nan 
      0.956         nan 
 
 s1KE5  25010.0 
     59.716         nan 
   2807.678         nan 
      0.968         nan 
   1640.800         nan 
      6.817         nan 
      0.956         nan 
 
 s1KE5  26010.0 
     60.557         nan 
   2879.859         nan 
      0.969         nan 
   1684.900         nan 
      6.832         nan 
      0.956         nan 
 
 s1KE5  27010.0 
     61.320         nan 
   2902.574         nan 
      0.970         nan 
   1701.900         nan 
      6.826         nan 
      0.956         nan 
 
 s1KE6  10.0 
      1.967         nan 
     29.600         nan 
      0.200         nan 
      9.000         nan 
      3.122         nan 
      0.880         nan 
 
 s1KE6  1010.0 
     17.390         nan 
    629.119         nan 
      0.842         nan 
    258.900         nan 
      6.453         nan 
      0.968         nan 
 
 s1KE6  2010.0 
     22.731         nan 
    918.980         nan 
      0.881         nan 
    390.900         nan 
      6.598         nan 
      0.968         nan 
 
 s1KE6  3010.0 
     26.615         nan 
   1119.921         nan 
      0.902         nan 
    492.300         nan 
      6.663         nan 
      0.967         nan 
 
 s1KE6  4010.0 
     29.774         nan 
   1363.194         nan 
      0.912         nan 
    593.500         nan 
      6.755         nan 
      0.969         nan 
 
 s1KE6  5010.0 
     32.349         nan 
   1486.790         nan 
      0.922         nan 
    667.300         nan 
      6.761         nan 
      0.968         nan 
 
 s1KE6  6010.0 
     35.379         nan 
   1615.367         nan 
      0.928         nan 
    747.500         nan 
      6.801         nan 
      0.968         nan 
 
 s1KE6  7010.0 
     37.147         nan 
   1676.297         nan 
      0.935         nan 
    807.700         nan 
      6.813         nan 
      0.968         nan 
 
 s1KE6  8010.0 
     38.941         nan 
   1800.293         nan 
      0.938         nan 
    878.100         nan 
      6.842         nan 
      0.968         nan 
 
 s1KE6  9010.0 
     41.294         nan 
   1901.678         nan 
      0.941         nan 
    943.800         nan 
      6.841         nan 
      0.967         nan 
 
 s1KE6  10010.0 
     43.006         nan 
   2045.371         nan 
      0.944         nan 
   1002.800         nan 
      6.877         nan 
      0.968         nan 
 
 s1KE6  11010.0 
     44.111         nan 
   2034.791         nan 
      0.949         nan 
   1035.900         nan 
      6.867         nan 
      0.968         nan 
 
 s1KE6  12010.0 
     45.610         nan 
   2152.836         nan 
      0.950         nan 
   1098.200         nan 
      6.882         nan 
      0.968         nan 
 
 s1KE6  13010.0 
     47.385         nan 
   2161.534         nan 
      0.953         nan 
   1148.900         nan 
      6.902         nan 
      0.968         nan 
 
 s1KE6  14010.0 
     48.594         nan 
   2229.570         nan 
      0.955         nan 
   1187.700         nan 
      6.901         nan 
      0.968         nan 
 
 s1KE6  15010.0 
     49.984         nan 
   2263.903         nan 
      0.957         nan 
   1235.300         nan 
      6.907         nan 
      0.968         nan 
 
 s1KE6  16010.0 
     50.675         nan 
   2295.435         nan 
      0.959         nan 
   1264.300         nan 
      6.898         nan 
      0.968         nan 
 
 s1KE6  17010.0 
     52.430         nan 
   2360.867         nan 
      0.960         nan 
   1317.200         nan 
      6.909         nan 
      0.968         nan 
 
 s1KE6  18010.0 
     53.248         nan 
   2395.515         nan 
      0.962         nan 
   1354.000         nan 
      6.912         nan 
      0.968         nan 
 
 s1KE6  19010.0 
     54.306         nan 
   2398.264         nan 
      0.963         nan 
   1387.000         nan 
      6.926         nan 
      0.968         nan 
 
 s1KE6  20010.0 
     55.291         nan 
   2396.037         nan 
      0.965         nan 
   1424.500         nan 
      6.926         nan 
      0.968         nan 
 
 s1KE6  21010.0 
     56.396         nan 
   2499.275         nan 
      0.966         nan 
   1465.900         nan 
      6.931         nan 
      0.968         nan 
 
 s1KE6  22010.0 
     57.195         nan 
   2534.359         nan 
      0.967         nan 
   1498.100         nan 
      6.940         nan 
      0.968         nan 
 
 s1KE6  23010.0 
     57.746         nan 
   2533.860         nan 
      0.968         nan 
   1519.100         nan 
      6.930         nan 
      0.968         nan 
 
 s1KE6  24010.0 
     59.325         nan 
   2584.861         nan 
      0.969         nan 
   1564.800         nan 
      6.937         nan 
      0.968         nan 
 
 s1KE6  25010.0 
     59.982         nan 
   2599.764         nan 
      0.970         nan 
   1594.700         nan 
      6.940         nan 
      0.968         nan 
 
 s1KE6  26010.0 
     60.755         nan 
   2644.609         nan 
      0.971         nan 
   1623.000         nan 
      6.947         nan 
      0.968         nan 
 
 s1KE6  27010.0 
     61.587         nan 
   2620.715         nan 
      0.972         nan 
   1650.800         nan 
      6.949         nan 
      0.968         nan 
 
 s1KO1  10.0 
      1.791         nan 
     30.200         nan 
      0.210         nan 
      8.800         nan 
      3.059         nan 
      0.870         nan 
 
 s1KO1  1010.0 
     17.868         nan 
    644.387         nan 
      0.830         nan 
    275.800         nan 
      6.457         nan 
      0.967         nan 
 
 s1KO1  2010.0 
     23.100         nan 
    947.227         nan 
      0.878         nan 
    414.800         nan 
      6.604         nan 
      0.966         nan 
 
 s1KO1  3010.0 
     27.171         nan 
   1213.124         nan 
      0.898         nan 
    526.800         nan 
      6.699         nan 
      0.966         nan 
 
 s1KO1  4010.0 
     30.091         nan 
   1331.808         nan 
      0.912         nan 
    619.400         nan 
      6.759         nan 
      0.967         nan 
 
 s1KO1  5010.0 
     32.585         nan 
   1454.523         nan 
      0.923         nan 
    695.300         nan 
      6.797         nan 
      0.967         nan 
 
 s1KO1  6010.0 
     35.313         nan 
   1626.781         nan 
      0.927         nan 
    783.300         nan 
      6.825         nan 
      0.967         nan 
 
 s1KO1  7010.0 
     37.282         nan 
   1753.340         nan 
      0.934         nan 
    840.200         nan 
      6.839         nan 
      0.967         nan 
 
 s1KO1  8010.0 
     39.283         nan 
   1829.251         nan 
      0.938         nan 
    909.500         nan 
      6.869         nan 
      0.967         nan 
 
 s1KO1  9010.0 
     40.943         nan 
   1915.756         nan 
      0.942         nan 
    968.300         nan 
      6.874         nan 
      0.967         nan 
 
 s1KO1  10010.0 
     43.028         nan 
   2013.331         nan 
      0.945         nan 
   1026.000         nan 
      6.879         nan 
      0.967         nan 
 
 s1KO1  11010.0 
     44.316         nan 
   2126.318         nan 
      0.948         nan 
   1077.100         nan 
      6.907         nan 
      0.967         nan 
 
 s1KO1  12010.0 
     45.477         nan 
   2145.496         nan 
      0.951         nan 
   1122.900         nan 
      6.906         nan 
      0.967         nan 
 
 s1KO1  13010.0 
     47.105         nan 
   2236.616         nan 
      0.953         nan 
   1175.800         nan 
      6.922         nan 
      0.967         nan 
 
 s1KO1  14010.0 
     48.488         nan 
   2314.797         nan 
      0.955         nan 
   1219.700         nan 
      6.923         nan 
      0.967         nan 
 
 s1KO1  15010.0 
     49.794         nan 
   2345.859         nan 
      0.957         nan 
   1267.800         nan 
      6.930         nan 
      0.967         nan 
 
 s1KO1  16010.0 
     50.237         nan 
   2401.802         nan 
      0.959         nan 
   1294.000         nan 
      6.930         nan 
      0.967         nan 
 
 s1KO1  17010.0 
     51.858         nan 
   2472.904         nan 
      0.960         nan 
   1349.100         nan 
      6.937         nan 
      0.967         nan 
 
 s1KO1  18010.0 
     52.478         nan 
   2486.993         nan 
      0.962         nan 
   1381.500         nan 
      6.948         nan 
      0.967         nan 
 
 s1KO1  19010.0 
     53.949         nan 
   2543.108         nan 
      0.963         nan 
   1425.400         nan 
      6.953         nan 
      0.967         nan 
 
 s1KO1  20010.0 
     54.947         nan 
   2653.476         nan 
      0.963         nan 
   1462.300         nan 
      6.954         nan 
      0.967         nan 
 
 s1KO1  21010.0 
     55.975         nan 
   2638.458         nan 
      0.965         nan 
   1501.100         nan 
      6.963         nan 
      0.967         nan 
 
 s1KO1  22010.0 
     56.775         nan 
   2669.554         nan 
      0.966         nan 
   1534.500         nan 
      6.963         nan 
      0.967         nan 
 
 s1KO1  23010.0 
     57.066         nan 
   2695.220         nan 
      0.967         nan 
   1556.100         nan 
      6.959         nan 
      0.967         nan 
 
 s1KO1  24010.0 
     58.514         nan 
   2830.243         nan 
      0.968         nan 
   1600.000         nan 
      6.972         nan 
      0.967         nan 
 
 s1KO1  25010.0 
     59.387         nan 
   2800.706         nan 
      0.969         nan 
   1633.100         nan 
      6.974         nan 
      0.967         nan 
 
 s1KO1  26010.0 
     59.958         nan 
   2911.162         nan 
      0.969         nan 
   1662.600         nan 
      6.976         nan 
      0.967         nan 
 
 s1KO1  27010.0 
     60.668         nan 
   2888.838         nan 
      0.970         nan 
   1682.800         nan 
      6.979         nan 
      0.967         nan 
 
 s1KO2  10.0 
      1.939         nan 
     36.400         nan 
      0.130         nan 
      9.300         nan 
      3.174         nan 
      0.884         nan 
 
 s1KO2  1010.0 
     17.327         nan 
    615.062         nan 
      0.841         nan 
    266.400         nan 
      6.543         nan 
      0.971         nan 
 
 s1KO2  2010.0 
     23.244         nan 
    893.559         nan 
      0.882         nan 
    403.700         nan 
      6.700         nan 
      0.970         nan 
 
 s1KO2  3010.0 
     27.302         nan 
   1200.682         nan 
      0.899         nan 
    515.700         nan 
      6.807         nan 
      0.971         nan 
 
 s1KO2  4010.0 
     30.395         nan 
   1382.600         nan 
      0.913         nan 
    605.800         nan 
      6.850         nan 
      0.970         nan 
 
 s1KO2  5010.0 
     32.821         nan 
   1548.018         nan 
      0.922         nan 
    684.500         nan 
      6.891         nan 
      0.971         nan 
 
 s1KO2  6010.0 
     34.710         nan 
   1648.879         nan 
      0.928         nan 
    758.700         nan 
      6.907         nan 
      0.971         nan 
 
 s1KO2  7010.0 
     37.405         nan 
   1718.178         nan 
      0.934         nan 
    825.500         nan 
      6.942         nan 
      0.971         nan 
 
 s1KO2  8010.0 
     38.896         nan 
   1880.246         nan 
      0.938         nan 
    892.400         nan 
      6.966         nan 
      0.971         nan 
 
 s1KO2  9010.0 
     40.899         nan 
   1985.728         nan 
      0.941         nan 
    953.300         nan 
      6.981         nan 
      0.971         nan 
 
 s1KO2  10010.0 
     42.411         nan 
   2111.910         nan 
      0.944         nan 
   1012.800         nan 
      6.992         nan 
      0.971         nan 
 
 s1KO2  11010.0 
     43.921         nan 
   2124.298         nan 
      0.948         nan 
   1060.200         nan 
      7.000         nan 
      0.971         nan 
 
 s1KO2  12010.0 
     45.475         nan 
   2218.033         nan 
      0.950         nan 
   1111.000         nan 
      7.005         nan 
      0.971         nan 
 
 s1KO2  13010.0 
     46.669         nan 
   2282.270         nan 
      0.952         nan 
   1157.800         nan 
      7.014         nan 
      0.971         nan 
 
 s1KO2  14010.0 
     48.017         nan 
   2370.573         nan 
      0.954         nan 
   1207.400         nan 
      7.015         nan 
      0.971         nan 
 
 s1KO2  15010.0 
     49.362         nan 
   2307.831         nan 
      0.956         nan 
   1249.300         nan 
      7.028         nan 
      0.971         nan 
 
 s1KO2  16010.0 
     50.298         nan 
   2430.137         nan 
      0.958         nan 
   1293.500         nan 
      7.034         nan 
      0.971         nan 
 
 s1KO2  17010.0 
     51.771         nan 
   2564.866         nan 
      0.958         nan 
   1346.800         nan 
      7.039         nan 
      0.971         nan 
 
 s1KO2  18010.0 
     52.860         nan 
   2496.341         nan 
      0.961         nan 
   1372.800         nan 
      7.051         nan 
      0.971         nan 
 
 s1KO2  19010.0 
     53.566         nan 
   2604.127         nan 
      0.961         nan 
   1424.200         nan 
      7.055         nan 
      0.971         nan 
 
 s1KO2  20010.0 
     55.195         nan 
   2688.342         nan 
      0.962         nan 
   1472.500         nan 
      7.060         nan 
      0.971         nan 
 
 s1KO2  21010.0 
     55.179         nan 
   2581.067         nan 
      0.965         nan 
   1485.300         nan 
      7.060         nan 
      0.971         nan 
 
 s1KO2  22010.0 
     56.646         nan 
   2651.408         nan 
      0.966         nan 
   1522.300         nan 
      7.057         nan 
      0.971         nan 
 
 s1KO2  23010.0 
     57.277         nan 
   2683.021         nan 
      0.967         nan 
   1555.800         nan 
      7.059         nan 
      0.971         nan 
 
 s1KO2  24010.0 
     58.303         nan 
   2759.556         nan 
      0.967         nan 
   1595.900         nan 
      7.074         nan 
      0.971         nan 
 
 s1KO2  25010.0 
     59.205         nan 
   2837.572         nan 
      0.968         nan 
   1629.500         nan 
      7.070         nan 
      0.971         nan 
 
 s1KO2  26010.0 
     60.027         nan 
   2840.372         nan 
      0.969         nan 
   1661.100         nan 
      7.077         nan 
      0.971         nan 
 
 s1KO2  27010.0 
     60.582         nan 
   2873.593         nan 
      0.970         nan 
   1686.900         nan 
      7.079         nan 
      0.971         nan 
 
 s1KO3  10.0 
      2.199         nan 
     36.000         nan 
      0.160         nan 
      9.100         nan 
      3.127         nan 
      0.878         nan 
 
 s1KO3  1010.0 
     17.744         nan 
    667.663         nan 
      0.833         nan 
    269.700         nan 
      6.439         nan 
      0.967         nan 
 
 s1KO3  2010.0 
     23.543         nan 
   1027.860         nan 
      0.874         nan 
    414.100         nan 
      6.647         nan 
      0.968         nan 
 
 s1KO3  3010.0 
     27.384         nan 
   1260.180         nan 
      0.893         nan 
    531.900         nan 
      6.771         nan 
      0.969         nan 
 
 s1KO3  4010.0 
     30.207         nan 
   1462.202         nan 
      0.908         nan 
    616.800         nan 
      6.804         nan 
      0.969         nan 
 
 s1KO3  5010.0 
     33.883         nan 
   1624.586         nan 
      0.915         nan 
    718.300         nan 
      6.854         nan 
      0.968         nan 
 
 s1KO3  6010.0 
     36.299         nan 
   1739.380         nan 
      0.922         nan 
    800.100         nan 
      6.882         nan 
      0.969         nan 
 
 s1KO3  7010.0 
     38.225         nan 
   1822.455         nan 
      0.928         nan 
    874.600         nan 
      6.909         nan 
      0.969         nan 
 
 s1KO3  8010.0 
     40.116         nan 
   1929.454         nan 
      0.933         nan 
    941.100         nan 
      6.927         nan 
      0.969         nan 
 
 s1KO3  9010.0 
     41.919         nan 
   2058.750         nan 
      0.937         nan 
   1003.000         nan 
      6.935         nan 
      0.969         nan 
 
 s1KO3  10010.0 
     43.775         nan 
   2121.400         nan 
      0.940         nan 
   1071.500         nan 
      6.975         nan 
      0.969         nan 
 
 s1KO3  11010.0 
     44.968         nan 
   2141.227         nan 
      0.944         nan 
   1117.200         nan 
      6.958         nan 
      0.969         nan 
 
 s1KO3  12010.0 
     46.931         nan 
   2213.806         nan 
      0.947         nan 
   1182.000         nan 
      6.987         nan 
      0.969         nan 
 
 s1KO3  13010.0 
     48.075         nan 
   2262.636         nan 
      0.950         nan 
   1223.900         nan 
      6.980         nan 
      0.969         nan 
 
 s1KO3  14010.0 
     49.375         nan 
   2272.569         nan 
      0.953         nan 
   1272.300         nan 
      6.996         nan 
      0.969         nan 
 
 s1KO3  15010.0 
     51.362         nan 
   2438.919         nan 
      0.953         nan 
   1337.600         nan 
      7.000         nan 
      0.969         nan 
 
 s1KO3  16010.0 
     52.160         nan 
   2388.390         nan 
      0.956         nan 
   1370.400         nan 
      7.008         nan 
      0.969         nan 
 
 s1KO3  17010.0 
     53.680         nan 
   2497.463         nan 
      0.957         nan 
   1421.200         nan 
      7.012         nan 
      0.969         nan 
 
 s1KO3  18010.0 
     54.287         nan 
   2491.237         nan 
      0.959         nan 
   1454.400         nan 
      7.019         nan 
      0.969         nan 
 
 s1KO3  19010.0 
     55.515         nan 
   2555.783         nan 
      0.961         nan 
   1492.000         nan 
      7.024         nan 
      0.969         nan 
 
 s1KO3  20010.0 
     56.783         nan 
   2615.482         nan 
      0.962         nan 
   1544.200         nan 
      7.023         nan 
      0.969         nan 
 
 s1KO3  21010.0 
     57.468         nan 
   2635.879         nan 
      0.963         nan 
   1572.500         nan 
      7.034         nan 
      0.969         nan 
 
 s1KO3  22010.0 
     58.675         nan 
   2689.117         nan 
      0.964         nan 
   1613.600         nan 
      7.031         nan 
      0.969         nan 
 
 s1KO3  23010.0 
     59.380         nan 
   2733.075         nan 
      0.966         nan 
   1650.200         nan 
      7.051         nan 
      0.969         nan 
 
 s1KO3  24010.0 
     60.175         nan 
   2765.128         nan 
      0.967         nan 
   1682.900         nan 
      7.044         nan 
      0.969         nan 
 
 s1KO3  25010.0 
     61.173         nan 
   2805.053         nan 
      0.968         nan 
   1716.100         nan 
      7.043         nan 
      0.969         nan 
 
 s1KO3  26010.0 
     61.869         nan 
   2793.216         nan 
      0.969         nan 
   1748.300         nan 
      7.059         nan 
      0.969         nan 
 
 s1KO3  27010.0 
     62.849         nan 
   2848.342         nan 
      0.969         nan 
   1778.700         nan 
      7.052         nan 
      0.969         nan 
 
 s1KO4  10.0 
      2.207         nan 
     40.600         nan 
      0.130         nan 
      9.300         nan 
      3.174         nan 
      0.884         nan 
 
 s1KO4  1010.0 
     18.428         nan 
    635.044         nan 
      0.838         nan 
    281.300         nan 
      6.865         nan 
      0.980         nan 
 
 s1KO4  2010.0 
     24.187         nan 
   1013.717         nan 
      0.879         nan 
    418.400         nan 
      7.056         nan 
      0.981         nan 
 
 s1KO4  3010.0 
     27.856         nan 
   1153.748         nan 
      0.901         nan 
    528.700         nan 
      7.151         nan 
      0.981         nan 
 
 s1KO4  4010.0 
     31.417         nan 
   1447.973         nan 
      0.910         nan 
    628.500         nan 
      7.223         nan 
      0.981         nan 
 
 s1KO4  5010.0 
     34.183         nan 
   1602.651         nan 
      0.919         nan 
    709.700         nan 
      7.246         nan 
      0.981         nan 
 
 s1KO4  6010.0 
     36.495         nan 
   1690.592         nan 
      0.927         nan 
    780.700         nan 
      7.281         nan 
      0.981         nan 
 
 s1KO4  7010.0 
     38.783         nan 
   1882.531         nan 
      0.931         nan 
    853.100         nan 
      7.302         nan 
      0.981         nan 
 
 s1KO4  8010.0 
     40.630         nan 
   1962.054         nan 
      0.936         nan 
    917.200         nan 
      7.322         nan 
      0.981         nan 
 
 s1KO4  9010.0 
     42.376         nan 
   2072.990         nan 
      0.939         nan 
    978.800         nan 
      7.327         nan 
      0.981         nan 
 
 s1KO4  10010.0 
     44.579         nan 
   2136.283         nan 
      0.944         nan 
   1038.200         nan 
      7.357         nan 
      0.981         nan 
 
 s1KO4  11010.0 
     46.025         nan 
   2197.599         nan 
      0.946         nan 
   1090.100         nan 
      7.352         nan 
      0.981         nan 
 
 s1KO4  12010.0 
     47.462         nan 
   2278.586         nan 
      0.949         nan 
   1144.600         nan 
      7.364         nan 
      0.981         nan 
 
 s1KO4  13010.0 
     49.359         nan 
   2389.261         nan 
      0.950         nan 
   1209.000         nan 
      7.386         nan 
      0.981         nan 
 
 s1KO4  14010.0 
     50.321         nan 
   2421.195         nan 
      0.953         nan 
   1243.100         nan 
      7.377         nan 
      0.981         nan 
 
 s1KO4  15010.0 
     51.399         nan 
   2520.129         nan 
      0.954         nan 
   1289.200         nan 
      7.388         nan 
      0.981         nan 
 
 s1KO4  16010.0 
     53.060         nan 
   2574.749         nan 
      0.956         nan 
   1340.200         nan 
      7.404         nan 
      0.981         nan 
 
 s1KO4  17010.0 
     54.136         nan 
   2605.658         nan 
      0.957         nan 
   1382.100         nan 
      7.405         nan 
      0.981         nan 
 
 s1KO4  18010.0 
     54.986         nan 
   2673.872         nan 
      0.959         nan 
   1423.600         nan 
      7.406         nan 
      0.981         nan 
 
 s1KO4  19010.0 
     56.345         nan 
   2736.782         nan 
      0.960         nan 
   1463.200         nan 
      7.412         nan 
      0.981         nan 
 
 s1KO4  20010.0 
     57.550         nan 
   2772.072         nan 
      0.962         nan 
   1502.900         nan 
      7.424         nan 
      0.981         nan 
 
 s1KO4  21010.0 
     58.242         nan 
   2787.961         nan 
      0.963         nan 
   1533.700         nan 
      7.417         nan 
      0.981         nan 
 
 s1KO4  22010.0 
     59.213         nan 
   2865.713         nan 
      0.964         nan 
   1580.100         nan 
      7.426         nan 
      0.981         nan 
 
 s1KO4  23010.0 
     60.271         nan 
   2911.540         nan 
      0.965         nan 
   1614.400         nan 
      7.424         nan 
      0.981         nan 
 
 s1KO4  24010.0 
     60.764         nan 
   2941.348         nan 
      0.966         nan 
   1644.000         nan 
      7.432         nan 
      0.981         nan 
 
 s1KO4  25010.0 
     62.112         nan 
   2955.386         nan 
      0.967         nan 
   1684.900         nan 
      7.439         nan 
      0.981         nan 
 
 s1KO4  26010.0 
     62.721         nan 
   3060.848         nan 
      0.967         nan 
   1710.900         nan 
      7.442         nan 
      0.981         nan 
 
 s1KO4  27010.0 
     63.600         nan 
   3091.047         nan 
      0.968         nan 
   1746.700         nan 
      7.444         nan 
      0.981         nan 
 
 s1KO5  10.0 
      1.904         nan 
     32.800         nan 
      0.180         nan 
      9.000         nan 
      3.107         nan 
      0.876         nan 
 
 s1KO5  1010.0 
     17.920         nan 
    676.347         nan 
      0.834         nan 
    268.100         nan 
      6.399         nan 
      0.962         nan 
 
 s1KO5  2010.0 
     24.060         nan 
    999.881         nan 
      0.872         nan 
    422.600         nan 
      6.657         nan 
      0.964         nan 
 
 s1KO5  3010.0 
     27.580         nan 
   1232.860         nan 
      0.895         nan 
    528.200         nan 
      6.699         nan 
      0.963         nan 
 
 s1KO5  4010.0 
     30.672         nan 
   1362.644         nan 
      0.910         nan 
    617.700         nan 
      6.752         nan 
      0.963         nan 
 
 s1KO5  5010.0 
     34.074         nan 
   1540.584         nan 
      0.917         nan 
    716.800         nan 
      6.787         nan 
      0.962         nan 
 
 s1KO5  6010.0 
     35.969         nan 
   1632.253         nan 
      0.926         nan 
    789.500         nan 
      6.827         nan 
      0.963         nan 
 
 s1KO5  7010.0 
     38.672         nan 
   1793.904         nan 
      0.930         nan 
    867.400         nan 
      6.870         nan 
      0.964         nan 
 
 s1KO5  8010.0 
     40.891         nan 
   1911.857         nan 
      0.936         nan 
    930.400         nan 
      6.898         nan 
      0.964         nan 
 
 s1KO5  9010.0 
     42.941         nan 
   2017.325         nan 
      0.939         nan 
    995.800         nan 
      6.900         nan 
      0.963         nan 
 
 s1KO5  10010.0 
     44.087         nan 
   2048.086         nan 
      0.943         nan 
   1051.000         nan 
      6.908         nan 
      0.963         nan 
 
 s1KO5  11010.0 
     45.506         nan 
   2156.997         nan 
      0.946         nan 
   1102.700         nan 
      6.921         nan 
      0.963         nan 
 
 s1KO5  12010.0 
     47.471         nan 
   2297.789         nan 
      0.948         nan 
   1162.000         nan 
      6.930         nan 
      0.963         nan 
 
 s1KO5  13010.0 
     49.197         nan 
   2386.880         nan 
      0.950         nan 
   1223.400         nan 
      6.941         nan 
      0.963         nan 
 
 s1KO5  14010.0 
     50.077         nan 
   2438.666         nan 
      0.952         nan 
   1264.900         nan 
      6.945         nan 
      0.963         nan 
 
 s1KO5  15010.0 
     52.037         nan 
   2441.771         nan 
      0.955         nan 
   1308.900         nan 
      6.954         nan 
      0.963         nan 
 
 s1KO5  16010.0 
     52.738         nan 
   2499.610         nan 
      0.956         nan 
   1355.200         nan 
      6.956         nan 
      0.963         nan 
 
 s1KO5  17010.0 
     54.322         nan 
   2566.509         nan 
      0.958         nan 
   1399.800         nan 
      6.963         nan 
      0.963         nan 
 
 s1KO5  18010.0 
     55.050         nan 
   2573.214         nan 
      0.960         nan 
   1433.600         nan 
      6.968         nan 
      0.963         nan 
 
 s1KO5  19010.0 
     56.076         nan 
   2610.686         nan 
      0.961         nan 
   1468.100         nan 
      6.969         nan 
      0.963         nan 
 
 s1KO5  20010.0 
     57.329         nan 
   2709.852         nan 
      0.962         nan 
   1517.800         nan 
      6.978         nan 
      0.963         nan 
 
 s1KO5  21010.0 
     58.500         nan 
   2736.569         nan 
      0.963         nan 
   1552.500         nan 
      6.980         nan 
      0.963         nan 
 
 s1KO5  22010.0 
     59.306         nan 
   2849.352         nan 
      0.964         nan 
   1594.500         nan 
      6.990         nan 
      0.963         nan 
 
 s1KO5  23010.0 
     60.352         nan 
   2822.907         nan 
      0.965         nan 
   1626.400         nan 
      6.990         nan 
      0.963         nan 
 
 s1KO5  24010.0 
     61.272         nan 
   2867.027         nan 
      0.966         nan 
   1667.100         nan 
      6.996         nan 
      0.963         nan 
 
 s1KO5  25010.0 
     62.079         nan 
   2915.080         nan 
      0.967         nan 
   1697.300         nan 
      6.998         nan 
      0.963         nan 
 
 s1KO5  26010.0 
     62.874         nan 
   2939.343         nan 
      0.968         nan 
   1726.800         nan 
      6.997         nan 
      0.963         nan 
 
 s1KO5  27010.0 
     63.757         nan 
   3024.392         nan 
      0.969         nan 
   1762.200         nan 
      7.003         nan 
      0.963         nan 
 
 s1WE1  10.0 
      2.014         nan 
     20.650         nan 
      0.310         nan 
      8.400         nan 
      2.994         nan 
      0.866         nan 
 
 s1WE1  1010.0 
     16.750         nan 
    601.844         nan 
      0.854         nan 
    246.700         nan 
      6.433         nan 
      0.970         nan 
 
 s1WE1  2010.0 
     21.453         nan 
    796.027         nan 
      0.895         nan 
    367.900         nan 
      6.592         nan 
      0.970         nan 
 
 s1WE1  3010.0 
     24.949         nan 
   1060.092         nan 
      0.911         nan 
    462.300         nan 
      6.653         nan 
      0.970         nan 
 
 s1WE1  4010.0 
     28.256         nan 
   1134.094         nan 
      0.924         nan 
    541.600         nan 
      6.692         nan 
      0.970         nan 
 
 s1WE1  5010.0 
     30.447         nan 
   1293.014         nan 
      0.932         nan 
    613.800         nan 
      6.738         nan 
      0.970         nan 
 
 s1WE1  6010.0 
     32.440         nan 
   1422.768         nan 
      0.937         nan 
    677.500         nan 
      6.752         nan 
      0.970         nan 
 
 s1WE1  7010.0 
     34.843         nan 
   1482.821         nan 
      0.943         nan 
    738.200         nan 
      6.784         nan 
      0.970         nan 
 
 s1WE1  8010.0 
     36.177         nan 
   1561.978         nan 
      0.947         nan 
    792.200         nan 
      6.794         nan 
      0.970         nan 
 
 s1WE1  9010.0 
     37.663         nan 
   1694.780         nan 
      0.950         nan 
    839.400         nan 
      6.805         nan 
      0.970         nan 
 
 s1WE1  10010.0 
     39.276         nan 
   1782.219         nan 
      0.953         nan 
    886.200         nan 
      6.810         nan 
      0.970         nan 
 
 s1WE1  11010.0 
     41.106         nan 
   1840.974         nan 
      0.954         nan 
    945.200         nan 
      6.823         nan 
      0.970         nan 
 
 s1WE1  12010.0 
     42.322         nan 
   1892.260         nan 
      0.957         nan 
    986.100         nan 
      6.843         nan 
      0.970         nan 
 
 s1WE1  13010.0 
     43.505         nan 
   2002.095         nan 
      0.959         nan 
   1023.600         nan 
      6.838         nan 
      0.970         nan 
 
 s1WE1  14010.0 
     44.701         nan 
   2001.813         nan 
      0.961         nan 
   1062.200         nan 
      6.848         nan 
      0.970         nan 
 
 s1WE1  15010.0 
     45.979         nan 
   2044.107         nan 
      0.962         nan 
   1105.500         nan 
      6.861         nan 
      0.970         nan 
 
 s1WE1  16010.0 
     46.897         nan 
   2121.719         nan 
      0.964         nan 
   1142.800         nan 
      6.866         nan 
      0.970         nan 
 
 s1WE1  17010.0 
     47.685         nan 
   2146.617         nan 
      0.965         nan 
   1177.800         nan 
      6.868         nan 
      0.970         nan 
 
 s1WE1  18010.0 
     48.932         nan 
   2181.322         nan 
      0.966         nan 
   1212.500         nan 
      6.878         nan 
      0.971         nan 
 
 s1WE1  19010.0 
     49.705         nan 
   2228.031         nan 
      0.967         nan 
   1242.000         nan 
      6.878         nan 
      0.971         nan 
 
 s1WE1  20010.0 
     50.631         nan 
   2267.704         nan 
      0.968         nan 
   1282.900         nan 
      6.885         nan 
      0.971         nan 
 
 s1WE1  21010.0 
     51.667         nan 
   2378.446         nan 
      0.969         nan 
   1310.300         nan 
      6.882         nan 
      0.970         nan 
 
 s1WE1  22010.0 
     52.544         nan 
   2355.419         nan 
      0.970         nan 
   1336.300         nan 
      6.891         nan 
      0.971         nan 
 
 s1WE1  23010.0 
     53.315         nan 
   2453.127         nan 
      0.971         nan 
   1371.600         nan 
      6.885         nan 
      0.970         nan 
 
 s1WE1  24010.0 
     54.098         nan 
   2488.389         nan 
      0.971         nan 
   1405.500         nan 
      6.892         nan 
      0.970         nan 
 
 s1WE1  25010.0 
     54.655         nan 
   2455.782         nan 
      0.973         nan 
   1424.200         nan 
      6.886         nan 
      0.970         nan 
 
 s1WE1  26010.0 
     55.203         nan 
   2529.023         nan 
      0.973         nan 
   1448.100         nan 
      6.892         nan 
      0.970         nan 
 
 s1WE1  27010.0 
     56.106         nan 
   2553.897         nan 
      0.974         nan 
   1484.700         nan 
      6.897         nan 
      0.970         nan 
 
 s1WE2  10.0 
      2.134         nan 
     44.300         nan 
      0.090         nan 
      9.500         nan 
      3.214         nan 
      0.888         nan 
 
 s1WE2  1010.0 
     16.509         nan 
    586.866         nan 
      0.859         nan 
    238.500         nan 
      6.396         nan 
      0.972         nan 
 
 s1WE2  2010.0 
     21.390         nan 
    932.551         nan 
      0.889         nan 
    369.400         nan 
      6.562         nan 
      0.971         nan 
 
 s1WE2  3010.0 
     25.027         nan 
   1012.988         nan 
      0.911         nan 
    458.100         nan 
      6.620         nan 
      0.971         nan 
 
 s1WE2  4010.0 
     27.999         nan 
   1304.307         nan 
      0.919         nan 
    548.600         nan 
      6.669         nan 
      0.971         nan 
 
 s1WE2  5010.0 
     31.149         nan 
   1448.401         nan 
      0.926         nan 
    631.300         nan 
      6.731         nan 
      0.971         nan 
 
 s1WE2  6010.0 
     33.637         nan 
   1583.905         nan 
      0.931         nan 
    705.400         nan 
      6.748         nan 
      0.971         nan 
 
 s1WE2  7010.0 
     35.556         nan 
   1695.005         nan 
      0.937         nan 
    767.500         nan 
      6.753         nan 
      0.971         nan 
 
 s1WE2  8010.0 
     37.556         nan 
   1731.182         nan 
      0.941         nan 
    829.200         nan 
      6.783         nan 
      0.971         nan 
 
 s1WE2  9010.0 
     39.035         nan 
   1879.649         nan 
      0.944         nan 
    882.400         nan 
      6.784         nan 
      0.971         nan 
 
 s1WE2  10010.0 
     41.063         nan 
   1926.599         nan 
      0.947         nan 
    941.000         nan 
      6.817         nan 
      0.971         nan 
 
 s1WE2  11010.0 
     42.485         nan 
   1962.163         nan 
      0.950         nan 
    993.200         nan 
      6.820         nan 
      0.971         nan 
 
 s1WE2  12010.0 
     44.067         nan 
   2104.829         nan 
      0.952         nan 
   1043.800         nan 
      6.828         nan 
      0.971         nan 
 
 s1WE2  13010.0 
     45.035         nan 
   2136.177         nan 
      0.955         nan 
   1080.800         nan 
      6.823         nan 
      0.971         nan 
 
 s1WE2  14010.0 
     47.007         nan 
   2213.154         nan 
      0.956         nan 
   1138.500         nan 
      6.842         nan 
      0.971         nan 
 
 s1WE2  15010.0 
     47.813         nan 
   2300.874         nan 
      0.958         nan 
   1166.400         nan 
      6.843         nan 
      0.971         nan 
 
 s1WE2  16010.0 
     48.832         nan 
   2348.557         nan 
      0.959         nan 
   1219.100         nan 
      6.851         nan 
      0.971         nan 
 
 s1WE2  17010.0 
     50.480         nan 
   2437.197         nan 
      0.960         nan 
   1261.300         nan 
      6.852         nan 
      0.971         nan 
 
 s1WE2  18010.0 
     51.594         nan 
   2404.414         nan 
      0.962         nan 
   1304.300         nan 
      6.862         nan 
      0.971         nan 
 
 s1WE2  19010.0 
     52.055         nan 
   2438.445         nan 
      0.964         nan 
   1325.100         nan 
      6.856         nan 
      0.971         nan 
 
 s1WE2  20010.0 
     53.376         nan 
   2517.366         nan 
      0.964         nan 
   1373.200         nan 
      6.871         nan 
      0.971         nan 
 
 s1WE2  21010.0 
     54.489         nan 
   2537.755         nan 
      0.966         nan 
   1402.600         nan 
      6.875         nan 
      0.971         nan 
 
 s1WE2  22010.0 
     55.461         nan 
   2570.442         nan 
      0.967         nan 
   1442.800         nan 
      6.886         nan 
      0.971         nan 
 
 s1WE2  23010.0 
     56.084         nan 
   2567.758         nan 
      0.968         nan 
   1466.900         nan 
      6.879         nan 
      0.971         nan 
 
 s1WE2  24010.0 
     56.777         nan 
   2613.635         nan 
      0.969         nan 
   1487.200         nan 
      6.877         nan 
      0.971         nan 
 
 s1WE2  25010.0 
     58.287         nan 
   2764.318         nan 
      0.969         nan 
   1540.100         nan 
      6.891         nan 
      0.971         nan 
 
 s1WE2  26010.0 
     59.247         nan 
   2755.476         nan 
      0.970         nan 
   1577.400         nan 
      6.893         nan 
      0.971         nan 
 
 s1WE2  27010.0 
     59.460         nan 
   2732.602         nan 
      0.971         nan 
   1593.300         nan 
      6.898         nan 
      0.971         nan 
 
 s1WE3  10.0 
      1.877         nan 
     36.800         nan 
      0.210         nan 
      8.700         nan 
      3.019         nan 
      0.862         nan 
 
 s1WE3  1010.0 
     17.116         nan 
    627.449         nan 
      0.840         nan 
    266.600         nan 
      6.455         nan 
      0.967         nan 
 
 s1WE3  2010.0 
     22.343         nan 
    889.293         nan 
      0.883         nan 
    400.900         nan 
      6.652         nan 
      0.968         nan 
 
 s1WE3  3010.0 
     26.645         nan 
   1076.991         nan 
      0.905         nan 
    506.500         nan 
      6.736         nan 
      0.967         nan 
 
 s1WE3  4010.0 
     29.464         nan 
   1284.846         nan 
      0.917         nan 
    593.500         nan 
      6.772         nan 
      0.967         nan 
 
 s1WE3  5010.0 
     32.609         nan 
   1373.813         nan 
      0.927         nan 
    670.000         nan 
      6.813         nan 
      0.967         nan 
 
 s1WE3  6010.0 
     34.561         nan 
   1401.832         nan 
      0.936         nan 
    737.700         nan 
      6.847         nan 
      0.967         nan 
 
 s1WE3  7010.0 
     36.523         nan 
   1569.398         nan 
      0.939         nan 
    801.600         nan 
      6.849         nan 
      0.967         nan 
 
 s1WE3  8010.0 
     38.016         nan 
   1644.463         nan 
      0.945         nan 
    853.100         nan 
      6.864         nan 
      0.967         nan 
 
 s1WE3  9010.0 
     40.534         nan 
   1719.763         nan 
      0.948         nan 
    918.400         nan 
      6.906         nan 
      0.967         nan 
 
 s1WE3  10010.0 
     41.647         nan 
   1792.146         nan 
      0.951         nan 
    963.000         nan 
      6.903         nan 
      0.967         nan 
 
 s1WE3  11010.0 
     43.513         nan 
   1952.311         nan 
      0.952         nan 
   1020.800         nan 
      6.911         nan 
      0.967         nan 
 
 s1WE3  12010.0 
     44.661         nan 
   2025.641         nan 
      0.955         nan 
   1061.600         nan 
      6.936         nan 
      0.968         nan 
 
 s1WE3  13010.0 
     45.330         nan 
   2000.786         nan 
      0.959         nan 
   1086.600         nan 
      6.914         nan 
      0.967         nan 
 
 s1WE3  14010.0 
     46.742         nan 
   2084.500         nan 
      0.960         nan 
   1141.200         nan 
      6.939         nan 
      0.967         nan 
 
 s1WE3  15010.0 
     47.920         nan 
   2181.763         nan 
      0.961         nan 
   1176.700         nan 
      6.934         nan 
      0.967         nan 
 
 s1WE3  16010.0 
     49.086         nan 
   2171.690         nan 
      0.963         nan 
   1205.900         nan 
      6.939         nan 
      0.967         nan 
 
 s1WE3  17010.0 
     49.961         nan 
   2228.119         nan 
      0.965         nan 
   1246.200         nan 
      6.946         nan 
      0.967         nan 
 
 s1WE3  18010.0 
     51.207         nan 
   2308.151         nan 
      0.965         nan 
   1290.500         nan 
      6.959         nan 
      0.967         nan 
 
 s1WE3  19010.0 
     51.852         nan 
   2382.719         nan 
      0.967         nan 
   1316.500         nan 
      6.960         nan 
      0.967         nan 
 
 s1WE3  20010.0 
     52.888         nan 
   2443.139         nan 
      0.967         nan 
   1353.500         nan 
      6.963         nan 
      0.967         nan 
 
 s1WE3  21010.0 
     54.026         nan 
   2499.331         nan 
      0.968         nan 
   1385.500         nan 
      6.968         nan 
      0.967         nan 
 
 s1WE3  22010.0 
     54.573         nan 
   2548.469         nan 
      0.969         nan 
   1412.200         nan 
      6.962         nan 
      0.967         nan 
 
 s1WE3  23010.0 
     55.850         nan 
   2557.920         nan 
      0.970         nan 
   1449.900         nan 
      6.978         nan 
      0.967         nan 
 
 s1WE3  24010.0 
     56.220         nan 
   2559.506         nan 
      0.971         nan 
   1467.900         nan 
      6.973         nan 
      0.967         nan 
 
 s1WE3  25010.0 
     56.987         nan 
   2637.178         nan 
      0.972         nan 
   1499.200         nan 
      6.972         nan 
      0.967         nan 
 
 s1WE3  26010.0 
     57.910         nan 
   2662.624         nan 
      0.973         nan 
   1527.600         nan 
      6.976         nan 
      0.967         nan 
 
 s1WE3  27010.0 
     58.692         nan 
   2718.862         nan 
      0.973         nan 
   1553.200         nan 
      6.981         nan 
      0.967         nan 
 
 s1WE4  10.0 
      1.778         nan 
     25.900         nan 
      0.300         nan 
      8.200         nan 
      2.907         nan 
      0.848         nan 
 
 s1WE4  1010.0 
     16.742         nan 
    547.206         nan 
      0.857         nan 
    240.600         nan 
      5.898         nan 
      0.933         nan 
 
 s1WE4  2010.0 
     21.484         nan 
    800.830         nan 
      0.895         nan 
    359.900         nan 
      5.980         nan 
      0.929         nan 
 
 s1WE4  3010.0 
     25.499         nan 
    990.076         nan 
      0.914         nan 
    458.100         nan 
      6.127         nan 
      0.933         nan 
 
 s1WE4  4010.0 
     28.032         nan 
   1145.746         nan 
      0.926         nan 
    529.000         nan 
      6.133         nan 
      0.931         nan 
 
 s1WE4  5010.0 
     30.402         nan 
   1269.560         nan 
      0.933         nan 
    604.300         nan 
      6.183         nan 
      0.933         nan 
 
 s1WE4  6010.0 
     32.424         nan 
   1370.836         nan 
      0.939         nan 
    664.100         nan 
      6.195         nan 
      0.932         nan 
 
 s1WE4  7010.0 
     34.653         nan 
   1454.919         nan 
      0.944         nan 
    722.200         nan 
      6.215         nan 
      0.932         nan 
 
 s1WE4  8010.0 
     36.484         nan 
   1533.704         nan 
      0.948         nan 
    789.000         nan 
      6.269         nan 
      0.933         nan 
 
 s1WE4  9010.0 
     37.903         nan 
   1633.294         nan 
      0.951         nan 
    834.700         nan 
      6.245         nan 
      0.932         nan 
 
 s1WE4  10010.0 
     39.396         nan 
   1714.088         nan 
      0.954         nan 
    878.900         nan 
      6.251         nan 
      0.931         nan 
 
 s1WE4  11010.0 
     40.838         nan 
   1837.024         nan 
      0.956         nan 
    923.400         nan 
      6.264         nan 
      0.932         nan 
 
 s1WE4  12010.0 
     42.189         nan 
   1881.524         nan 
      0.958         nan 
    970.300         nan 
      6.282         nan 
      0.932         nan 
 
 s1WE4  13010.0 
     43.514         nan 
   1916.728         nan 
      0.960         nan 
   1008.900         nan 
      6.287         nan 
      0.932         nan 
 
 s1WE4  14010.0 
     44.244         nan 
   1994.953         nan 
      0.961         nan 
   1051.300         nan 
      6.296         nan 
      0.932         nan 
 
 s1WE4  15010.0 
     45.737         nan 
   2063.403         nan 
      0.963         nan 
   1090.000         nan 
      6.309         nan 
      0.933         nan 
 
 s1WE4  16010.0 
     46.738         nan 
   2133.369         nan 
      0.964         nan 
   1130.300         nan 
      6.314         nan 
      0.933         nan 
 
 s1WE4  17010.0 
     47.826         nan 
   2240.487         nan 
      0.965         nan 
   1161.300         nan 
      6.318         nan 
      0.932         nan 
 
 s1WE4  18010.0 
     48.329         nan 
   2200.720         nan 
      0.967         nan 
   1187.000         nan 
      6.319         nan 
      0.933         nan 
 
 s1WE4  19010.0 
     49.586         nan 
   2278.910         nan 
      0.967         nan 
   1224.800         nan 
      6.314         nan 
      0.932         nan 
 
 s1WE4  20010.0 
     50.335         nan 
   2291.609         nan 
      0.969         nan 
   1255.800         nan 
      6.330         nan 
      0.933         nan 
 
 s1WE4  21010.0 
     51.088         nan 
   2341.047         nan 
      0.970         nan 
   1285.500         nan 
      6.328         nan 
      0.932         nan 
 
 s1WE4  22010.0 
     51.511         nan 
   2429.347         nan 
      0.971         nan 
   1308.100         nan 
      6.321         nan 
      0.932         nan 
 
 s1WE4  23010.0 
     52.538         nan 
   2386.699         nan 
      0.972         nan 
   1342.700         nan 
      6.334         nan 
      0.932         nan 
 
 s1WE4  24010.0 
     53.478         nan 
   2470.189         nan 
      0.972         nan 
   1369.400         nan 
      6.334         nan 
      0.932         nan 
 
 s1WE4  25010.0 
     54.324         nan 
   2556.704         nan 
      0.973         nan 
   1407.300         nan 
      6.345         nan 
      0.933         nan 
 
 s1WE4  26010.0 
     55.047         nan 
   2563.586         nan 
      0.973         nan 
   1427.300         nan 
      6.339         nan 
      0.932         nan 
 
 s1WE4  27010.0 
     55.597         nan 
   2622.155         nan 
      0.974         nan 
   1453.000         nan 
      6.340         nan 
      0.932         nan 
 
 s1WE5  10.0 
      2.000         nan 
     32.300         nan 
      0.210         nan 
      8.800         nan 
      3.054         nan 
      0.868         nan 
 
 s1WE5  1010.0 
     17.062         nan 
    611.632         nan 
      0.849         nan 
    256.600         nan 
      6.566         nan 
      0.971         nan 
 
 s1WE5  2010.0 
     21.825         nan 
    880.303         nan 
      0.890         nan 
    381.000         nan 
      6.739         nan 
      0.972         nan 
 
 s1WE5  3010.0 
     25.816         nan 
   1108.058         nan 
      0.908         nan 
    476.800         nan 
      6.816         nan 
      0.972         nan 
 
 s1WE5  4010.0 
     28.889         nan 
   1315.740         nan 
      0.918         nan 
    571.100         nan 
      6.904         nan 
      0.973         nan 
 
 s1WE5  5010.0 
     31.556         nan 
   1447.063         nan 
      0.925         nan 
    649.900         nan 
      6.919         nan 
      0.972         nan 
 
 s1WE5  6010.0 
     33.731         nan 
   1510.718         nan 
      0.933         nan 
    712.300         nan 
      6.937         nan 
      0.973         nan 
 
 s1WE5  7010.0 
     35.656         nan 
   1581.800         nan 
      0.939         nan 
    775.300         nan 
      6.962         nan 
      0.972         nan 
 
 s1WE5  8010.0 
     38.059         nan 
   1722.703         nan 
      0.941         nan 
    853.000         nan 
      6.987         nan 
      0.972         nan 
 
 s1WE5  9010.0 
     39.234         nan 
   1844.040         nan 
      0.945         nan 
    899.900         nan 
      7.004         nan 
      0.973         nan 
 
 s1WE5  10010.0 
     41.314         nan 
   1958.887         nan 
      0.947         nan 
    957.300         nan 
      7.009         nan 
      0.972         nan 
 
 s1WE5  11010.0 
     42.603         nan 
   2014.077         nan 
      0.950         nan 
   1009.200         nan 
      7.021         nan 
      0.973         nan 
 
 s1WE5  12010.0 
     43.896         nan 
   2030.841         nan 
      0.953         nan 
   1056.300         nan 
      7.036         nan 
      0.973         nan 
 
 s1WE5  13010.0 
     44.921         nan 
   2088.514         nan 
      0.955         nan 
   1095.100         nan 
      7.035         nan 
      0.973         nan 
 
 s1WE5  14010.0 
     46.471         nan 
   2192.684         nan 
      0.957         nan 
   1142.000         nan 
      7.040         nan 
      0.973         nan 
 
 s1WE5  15010.0 
     47.611         nan 
   2182.098         nan 
      0.959         nan 
   1177.800         nan 
      7.043         nan 
      0.973         nan 
 
 s1WE5  16010.0 
     48.603         nan 
   2261.847         nan 
      0.960         nan 
   1223.200         nan 
      7.048         nan 
      0.973         nan 
 
 s1WE5  17010.0 
     50.127         nan 
   2360.078         nan 
      0.961         nan 
   1271.700         nan 
      7.066         nan 
      0.973         nan 
 
 s1WE5  18010.0 
     51.203         nan 
   2373.110         nan 
      0.963         nan 
   1298.000         nan 
      7.066         nan 
      0.973         nan 
 
 s1WE5  19010.0 
     51.965         nan 
   2430.306         nan 
      0.964         nan 
   1336.200         nan 
      7.067         nan 
      0.973         nan 
 
 s1WE5  20010.0 
     52.942         nan 
   2414.850         nan 
      0.966         nan 
   1370.400         nan 
      7.083         nan 
      0.973         nan 
 
 s1WE5  21010.0 
     53.993         nan 
   2523.251         nan 
      0.966         nan 
   1409.700         nan 
      7.082         nan 
      0.973         nan 
 
 s1WE5  22010.0 
     55.137         nan 
   2529.294         nan 
      0.967         nan 
   1450.900         nan 
      7.085         nan 
      0.973         nan 
 
 s1WE5  23010.0 
     55.684         nan 
   2598.743         nan 
      0.968         nan 
   1472.400         nan 
      7.090         nan 
      0.973         nan 
 
 s1WE5  24010.0 
     56.780         nan 
   2601.568         nan 
      0.969         nan 
   1508.700         nan 
      7.084         nan 
      0.973         nan 
 
 s1WE5  25010.0 
     57.192         nan 
   2607.375         nan 
      0.970         nan 
   1528.200         nan 
      7.086         nan 
      0.973         nan 
 
 s1WE5  26010.0 
     58.077         nan 
   2620.638         nan 
      0.971         nan 
   1561.200         nan 
      7.093         nan 
      0.973         nan 
 
 s1WE5  27010.0 
     58.949         nan 
   2676.587         nan 
      0.972         nan 
   1589.500         nan 
      7.095         nan 
      0.973         nan 
 
 s1WE6  10.0 
      1.999         nan 
     35.300         nan 
      0.160         nan 
      9.100         nan 
      3.127         nan 
      0.878         nan 
 
 s1WE6  1010.0 
     16.784         nan 
    578.405         nan 
      0.855         nan 
    247.400         nan 
      6.400         nan 
      0.966         nan 
 
 s1WE6  2010.0 
     22.121         nan 
    863.952         nan 
      0.890         nan 
    377.600         nan 
      6.602         nan 
      0.967         nan 
 
 s1WE6  3010.0 
     25.699         nan 
   1059.516         nan 
      0.911         nan 
    463.000         nan 
      6.631         nan 
      0.966         nan 
 
 s1WE6  4010.0 
     28.627         nan 
   1287.126         nan 
      0.920         nan 
    554.200         nan 
      6.704         nan 
      0.967         nan 
 
 s1WE6  5010.0 
     31.572         nan 
   1504.302         nan 
      0.927         nan 
    629.200         nan 
      6.721         nan 
      0.966         nan 
 
 s1WE6  6010.0 
     33.994         nan 
   1591.172         nan 
      0.934         nan 
    695.900         nan 
      6.747         nan 
      0.967         nan 
 
 s1WE6  7010.0 
     35.428         nan 
   1664.214         nan 
      0.938         nan 
    763.600         nan 
      6.768         nan 
      0.966         nan 
 
 s1WE6  8010.0 
     37.408         nan 
   1768.335         nan 
      0.942         nan 
    821.900         nan 
      6.781         nan 
      0.966         nan 
 
 s1WE6  9010.0 
     39.784         nan 
   1854.908         nan 
      0.946         nan 
    883.800         nan 
      6.826         nan 
      0.967         nan 
 
 s1WE6  10010.0 
     40.629         nan 
   1876.075         nan 
      0.949         nan 
    924.600         nan 
      6.811         nan 
      0.967         nan 
 
 s1WE6  11010.0 
     42.793         nan 
   2069.445         nan 
      0.950         nan 
    997.700         nan 
      6.830         nan 
      0.966         nan 
 
 s1WE6  12010.0 
     43.597         nan 
   2117.188         nan 
      0.953         nan 
   1030.900         nan 
      6.842         nan 
      0.967         nan 
 
 s1WE6  13010.0 
     45.203         nan 
   2128.062         nan 
      0.955         nan 
   1072.400         nan 
      6.843         nan 
      0.966         nan 
 
 s1WE6  14010.0 
     46.433         nan 
   2271.534         nan 
      0.956         nan 
   1127.700         nan 
      6.859         nan 
      0.967         nan 
 
 s1WE6  15010.0 
     47.800         nan 
   2284.949         nan 
      0.958         nan 
   1167.600         nan 
      6.862         nan 
      0.967         nan 
 
 s1WE6  16010.0 
     48.745         nan 
   2302.197         nan 
      0.960         nan 
   1199.500         nan 
      6.855         nan 
      0.967         nan 
 
 s1WE6  17010.0 
     49.884         nan 
   2397.327         nan 
      0.961         nan 
   1241.800         nan 
      6.873         nan 
      0.967         nan 
 
 s1WE6  18010.0 
     51.128         nan 
   2439.791         nan 
      0.962         nan 
   1287.900         nan 
      6.878         nan 
      0.967         nan 
 
 s1WE6  19010.0 
     51.800         nan 
   2446.728         nan 
      0.964         nan 
   1322.700         nan 
      6.883         nan 
      0.967         nan 
 
 s1WE6  20010.0 
     52.680         nan 
   2510.844         nan 
      0.965         nan 
   1350.200         nan 
      6.879         nan 
      0.967         nan 
 
 s1WE6  21010.0 
     53.567         nan 
   2568.671         nan 
      0.966         nan 
   1385.900         nan 
      6.884         nan 
      0.967         nan 
 
 s1WE6  22010.0 
     54.742         nan 
   2598.449         nan 
      0.967         nan 
   1426.600         nan 
      6.887         nan 
      0.967         nan 
 
 s1WE6  23010.0 
     55.556         nan 
   2669.831         nan 
      0.968         nan 
   1452.300         nan 
      6.889         nan 
      0.967         nan 
 
 s1WE6  24010.0 
     56.485         nan 
   2769.284         nan 
      0.968         nan 
   1486.300         nan 
      6.895         nan 
      0.967         nan 
 
 s1WE6  25010.0 
     56.899         nan 
   2760.251         nan 
      0.969         nan 
   1513.700         nan 
      6.897         nan 
      0.967         nan 
 
 s1WE6  26010.0 
     58.049         nan 
   2752.789         nan 
      0.970         nan 
   1550.100         nan 
      6.901         nan 
      0.967         nan 
 
 s1WE6  27010.0 
     58.773         nan 
   2826.364         nan 
      0.971         nan 
   1580.300         nan 
      6.906         nan 
      0.967         nan 
 
 s1WT1  10.0 
      1.916         nan 
     38.800         nan 
      0.160         nan 
      9.100         nan 
      3.122         nan 
      0.876         nan 
 
 s1WT1  1010.0 
     17.457         nan 
    734.423         nan 
      0.818         nan 
    286.300         nan 
      6.696         nan 
      0.975         nan 
 
 s1WT1  2010.0 
     23.352         nan 
   1055.647         nan 
      0.863         nan 
    442.500         nan 
      6.895         nan 
      0.975         nan 
 
 s1WT1  3010.0 
     27.003         nan 
   1237.734         nan 
      0.888         nan 
    560.400         nan 
      6.978         nan 
      0.975         nan 
 
 s1WT1  4010.0 
     30.225         nan 
   1480.451         nan 
      0.902         nan 
    663.700         nan 
      7.047         nan 
      0.976         nan 
 
 s1WT1  5010.0 
     33.165         nan 
   1701.636         nan 
      0.910         nan 
    763.300         nan 
      7.084         nan 
      0.976         nan 
 
 s1WT1  6010.0 
     35.742         nan 
   1722.070         nan 
      0.920         nan 
    844.500         nan 
      7.128         nan 
      0.976         nan 
 
 s1WT1  7010.0 
     37.445         nan 
   1821.590         nan 
      0.927         nan 
    918.900         nan 
      7.148         nan 
      0.976         nan 
 
 s1WT1  8010.0 
     39.694         nan 
   2049.044         nan 
      0.931         nan 
    990.600         nan 
      7.164         nan 
      0.976         nan 
 
 s1WT1  9010.0 
     41.937         nan 
   2088.250         nan 
      0.936         nan 
   1058.000         nan 
      7.181         nan 
      0.976         nan 
 
 s1WT1  10010.0 
     43.431         nan 
   2226.046         nan 
      0.939         nan 
   1122.600         nan 
      7.189         nan 
      0.976         nan 
 
 s1WT1  11010.0 
     44.824         nan 
   2234.569         nan 
      0.943         nan 
   1173.200         nan 
      7.195         nan 
      0.976         nan 
 
 s1WT1  12010.0 
     46.075         nan 
   2323.568         nan 
      0.946         nan 
   1221.700         nan 
      7.201         nan 
      0.976         nan 
 
 s1WT1  13010.0 
     47.869         nan 
   2415.768         nan 
      0.948         nan 
   1282.500         nan 
      7.221         nan 
      0.976         nan 
 
 s1WT1  14010.0 
     49.193         nan 
   2466.340         nan 
      0.950         nan 
   1339.200         nan 
      7.229         nan 
      0.976         nan 
 
 s1WT1  15010.0 
     50.506         nan 
   2563.540         nan 
      0.952         nan 
   1393.100         nan 
      7.238         nan 
      0.976         nan 
 
 s1WT1  16010.0 
     51.517         nan 
   2565.360         nan 
      0.955         nan 
   1416.900         nan 
      7.248         nan 
      0.976         nan 
 
 s1WT1  17010.0 
     53.040         nan 
   2746.952         nan 
      0.955         nan 
   1486.400         nan 
      7.258         nan 
      0.976         nan 
 
 s1WT1  18010.0 
     53.613         nan 
   2761.823         nan 
      0.957         nan 
   1518.300         nan 
      7.251         nan 
      0.976         nan 
 
 s1WT1  19010.0 
     55.168         nan 
   2777.196         nan 
      0.959         nan 
   1554.200         nan 
      7.257         nan 
      0.976         nan 
 
 s1WT1  20010.0 
     56.142         nan 
   2815.393         nan 
      0.960         nan 
   1594.000         nan 
      7.260         nan 
      0.976         nan 
 
 s1WT1  21010.0 
     57.107         nan 
   2837.245         nan 
      0.962         nan 
   1630.500         nan 
      7.266         nan 
      0.976         nan 
 
 s1WT1  22010.0 
     58.127         nan 
   2959.891         nan 
      0.962         nan 
   1676.000         nan 
      7.271         nan 
      0.976         nan 
 
 s1WT1  23010.0 
     59.123         nan 
   2903.254         nan 
      0.964         nan 
   1710.500         nan 
      7.273         nan 
      0.976         nan 
 
 s1WT1  24010.0 
     60.094         nan 
   2986.814         nan 
      0.965         nan 
   1752.500         nan 
      7.280         nan 
      0.976         nan 
 
 s1WT1  25010.0 
     60.859         nan 
   3043.302         nan 
      0.966         nan 
   1783.500         nan 
      7.284         nan 
      0.976         nan 
 
 s1WT1  26010.0 
     61.560         nan 
   3029.132         nan 
      0.967         nan 
   1821.800         nan 
      7.288         nan 
      0.976         nan 
 
 s1WT1  27010.0 
     62.344         nan 
   3160.755         nan 
      0.967         nan 
   1847.000         nan 
      7.283         nan 
      0.976         nan 
 
 s1WT2  10.0 
      1.746         nan 
     29.000         nan 
      0.190         nan 
      9.000         nan 
      3.114         nan 
      0.878         nan 
 
 s1WT2  1010.0 
     16.262         nan 
    752.251         nan 
      0.831         nan 
    262.200         nan 
      6.322         nan 
      0.963         nan 
 
 s1WT2  2010.0 
     22.052         nan 
   1012.429         nan 
      0.873         nan 
    409.800         nan 
      6.559         nan 
      0.964         nan 
 
 s1WT2  3010.0 
     26.114         nan 
   1247.774         nan 
      0.892         nan 
    527.000         nan 
      6.631         nan 
      0.964         nan 
 
 s1WT2  4010.0 
     29.859         nan 
   1359.594         nan 
      0.906         nan 
    632.100         nan 
      6.708         nan 
      0.965         nan 
 
 s1WT2  5010.0 
     32.482         nan 
   1511.027         nan 
      0.916         nan 
    725.700         nan 
      6.761         nan 
      0.965         nan 
 
 s1WT2  6010.0 
     34.574         nan 
   1630.296         nan 
      0.924         nan 
    797.900         nan 
      6.774         nan 
      0.965         nan 
 
 s1WT2  7010.0 
     36.977         nan 
   1723.616         nan 
      0.930         nan 
    871.700         nan 
      6.787         nan 
      0.964         nan 
 
 s1WT2  8010.0 
     38.472         nan 
   1839.377         nan 
      0.936         nan 
    932.700         nan 
      6.801         nan 
      0.964         nan 
 
 s1WT2  9010.0 
     40.929         nan 
   1977.774         nan 
      0.938         nan 
   1008.000         nan 
      6.835         nan 
      0.965         nan 
 
 s1WT2  10010.0 
     41.992         nan 
   2012.311         nan 
      0.943         nan 
   1057.400         nan 
      6.837         nan 
      0.965         nan 
 
 s1WT2  11010.0 
     43.711         nan 
   2076.452         nan 
      0.947         nan 
   1113.500         nan 
      6.864         nan 
      0.965         nan 
 
 s1WT2  12010.0 
     45.106         nan 
   2126.562         nan 
      0.950         nan 
   1161.200         nan 
      6.857         nan 
      0.965         nan 
 
 s1WT2  13010.0 
     46.088         nan 
   2153.686         nan 
      0.953         nan 
   1211.000         nan 
      6.873         nan 
      0.965         nan 
 
 s1WT2  14010.0 
     47.604         nan 
   2319.371         nan 
      0.954         nan 
   1264.600         nan 
      6.882         nan 
      0.965         nan 
 
 s1WT2  15010.0 
     48.877         nan 
   2356.943         nan 
      0.956         nan 
   1305.200         nan 
      6.888         nan 
      0.965         nan 
 
 s1WT2  16010.0 
     50.103         nan 
   2400.608         nan 
      0.958         nan 
   1354.300         nan 
      6.905         nan 
      0.965         nan 
 
 s1WT2  17010.0 
     51.061         nan 
   2405.247         nan 
      0.960         nan 
   1394.100         nan 
      6.902         nan 
      0.965         nan 
 
 s1WT2  18010.0 
     52.348         nan 
   2545.400         nan 
      0.961         nan 
   1432.500         nan 
      6.907         nan 
      0.965         nan 
 
 s1WT2  19010.0 
     52.963         nan 
   2511.295         nan 
      0.963         nan 
   1468.900         nan 
      6.912         nan 
      0.965         nan 
 
 s1WT2  20010.0 
     53.871         nan 
   2530.772         nan 
      0.964         nan 
   1500.700         nan 
      6.908         nan 
      0.965         nan 
 
 s1WT2  21010.0 
     54.766         nan 
   2616.667         nan 
      0.965         nan 
   1544.500         nan 
      6.914         nan 
      0.965         nan 
 
 s1WT2  22010.0 
     55.472         nan 
   2624.058         nan 
      0.966         nan 
   1570.300         nan 
      6.928         nan 
      0.965         nan 
 
 s1WT2  23010.0 
     56.502         nan 
   2733.084         nan 
      0.967         nan 
   1611.100         nan 
      6.925         nan 
      0.965         nan 
 
 s1WT2  24010.0 
     57.427         nan 
   2770.786         nan 
      0.968         nan 
   1644.800         nan 
      6.930         nan 
      0.965         nan 
 
 s1WT2  25010.0 
     57.913         nan 
   2776.523         nan 
      0.969         nan 
   1671.900         nan 
      6.929         nan 
      0.965         nan 
 
 s1WT2  26010.0 
     58.916         nan 
   2813.369         nan 
      0.970         nan 
   1706.600         nan 
      6.934         nan 
      0.965         nan 
 
 s1WT2  27010.0 
     59.206         nan 
   2811.326         nan 
      0.971         nan 
   1729.800         nan 
      6.938         nan 
      0.965         nan 
 
 s1WT3  10.0 
      1.632         nan 
     32.300         nan 
      0.220         nan 
      8.700         nan 
      3.032         nan 
      0.866         nan 
 
 s1WT3  1010.0 
     16.908         nan 
    648.774         nan 
      0.831         nan 
    274.000         nan 
      6.492         nan 
      0.965         nan 
 
 s1WT3  2010.0 
     22.375         nan 
   1099.934         nan 
      0.868         nan 
    424.700         nan 
      6.717         nan 
      0.967         nan 
 
 s1WT3  3010.0 
     26.122         nan 
   1221.066         nan 
      0.893         nan 
    544.200         nan 
      6.841         nan 
      0.968         nan 
 
 s1WT3  4010.0 
     29.389         nan 
   1441.723         nan 
      0.906         nan 
    641.800         nan 
      6.878         nan 
      0.968         nan 
 
 s1WT3  5010.0 
     32.304         nan 
   1476.006         nan 
      0.918         nan 
    720.400         nan 
      6.910         nan 
      0.968         nan 
 
 s1WT3  6010.0 
     34.570         nan 
   1635.937         nan 
      0.924         nan 
    811.600         nan 
      6.972         nan 
      0.969         nan 
 
 s1WT3  7010.0 
     36.298         nan 
   1721.546         nan 
      0.931         nan 
    877.200         nan 
      6.976         nan 
      0.968         nan 
 
 s1WT3  8010.0 
     38.673         nan 
   1820.700         nan 
      0.936         nan 
    946.000         nan 
      6.991         nan 
      0.968         nan 
 
 s1WT3  9010.0 
     40.386         nan 
   1911.720         nan 
      0.940         nan 
   1014.100         nan 
      7.006         nan 
      0.968         nan 
 
 s1WT3  10010.0 
     42.110         nan 
   2004.027         nan 
      0.943         nan 
   1076.100         nan 
      7.027         nan 
      0.968         nan 
 
 s1WT3  11010.0 
     43.818         nan 
   2115.433         nan 
      0.946         nan 
   1132.300         nan 
      7.041         nan 
      0.968         nan 
 
 s1WT3  12010.0 
     45.054         nan 
   2221.503         nan 
      0.948         nan 
   1184.400         nan 
      7.050         nan 
      0.968         nan 
 
 s1WT3  13010.0 
     46.684         nan 
   2196.564         nan 
      0.952         nan 
   1233.400         nan 
      7.067         nan 
      0.968         nan 
 
 s1WT3  14010.0 
     47.306         nan 
   2291.765         nan 
      0.954         nan 
   1274.700         nan 
      7.058         nan 
      0.968         nan 
 
 s1WT3  15010.0 
     48.694         nan 
   2371.848         nan 
      0.956         nan 
   1325.300         nan 
      7.072         nan 
      0.968         nan 
 
 s1WT3  16010.0 
     49.828         nan 
   2346.903         nan 
      0.958         nan 
   1359.100         nan 
      7.076         nan 
      0.968         nan 
 
 s1WT3  17010.0 
     50.604         nan 
   2406.786         nan 
      0.960         nan 
   1401.400         nan 
      7.083         nan 
      0.968         nan 
 
 s1WT3  18010.0 
     52.345         nan 
   2479.009         nan 
      0.961         nan 
   1454.100         nan 
      7.093         nan 
      0.968         nan 
 
 s1WT3  19010.0 
     52.908         nan 
   2502.383         nan 
      0.963         nan 
   1474.400         nan 
      7.086         nan 
      0.968         nan 
 
 s1WT3  20010.0 
     53.744         nan 
   2529.497         nan 
      0.964         nan 
   1517.100         nan 
      7.104         nan 
      0.968         nan 
 
 s1WT3  21010.0 
     55.226         nan 
   2608.284         nan 
      0.965         nan 
   1558.700         nan 
      7.102         nan 
      0.968         nan 
 
 s1WT3  22010.0 
     55.732         nan 
   2623.703         nan 
      0.966         nan 
   1592.200         nan 
      7.114         nan 
      0.968         nan 
 
 s1WT3  23010.0 
     56.733         nan 
   2674.422         nan 
      0.967         nan 
   1629.800         nan 
      7.115         nan 
      0.968         nan 
 
 s1WT3  24010.0 
     57.731         nan 
   2735.692         nan 
      0.968         nan 
   1660.100         nan 
      7.108         nan 
      0.968         nan 
 
 s1WT3  25010.0 
     58.114         nan 
   2730.653         nan 
      0.969         nan 
   1689.100         nan 
      7.114         nan 
      0.968         nan 
 
 s1WT3  26010.0 
     58.905         nan 
   2758.926         nan 
      0.970         nan 
   1721.100         nan 
      7.114         nan 
      0.968         nan 
 
 s1WT3  27010.0 
     59.628         nan 
   2852.386         nan 
      0.971         nan 
   1747.600         nan 
      7.124         nan 
      0.968         nan 
 
 s1WT4  10.0 
      2.047         nan 
     28.050         nan 
      0.250         nan 
      8.700         nan 
      3.054         nan 
      0.872         nan 
 
 s1WT4  1010.0 
     16.956         nan 
    702.394         nan 
      0.829         nan 
    274.000         nan 
      6.533         nan 
      0.971         nan 
 
 s1WT4  2010.0 
     22.604         nan 
   1027.298         nan 
      0.874         nan 
    416.300         nan 
      6.734         nan 
      0.972         nan 
 
 s1WT4  3010.0 
     26.463         nan 
   1167.872         nan 
      0.897         nan 
    527.800         nan 
      6.807         nan 
      0.971         nan 
 
 s1WT4  4010.0 
     29.974         nan 
   1349.765         nan 
      0.908         nan 
    632.200         nan 
      6.886         nan 
      0.972         nan 
 
 s1WT4  5010.0 
     32.385         nan 
   1495.653         nan 
      0.918         nan 
    713.900         nan 
      6.908         nan 
      0.972         nan 
 
 s1WT4  6010.0 
     35.196         nan 
   1667.170         nan 
      0.924         nan 
    799.700         nan 
      6.940         nan 
      0.972         nan 
 
 s1WT4  7010.0 
     37.520         nan 
   1772.706         nan 
      0.931         nan 
    868.500         nan 
      6.959         nan 
      0.972         nan 
 
 s1WT4  8010.0 
     39.135         nan 
   1832.788         nan 
      0.936         nan 
    932.400         nan 
      6.978         nan 
      0.972         nan 
 
 s1WT4  9010.0 
     40.979         nan 
   1970.884         nan 
      0.940         nan 
    999.600         nan 
      6.999         nan 
      0.972         nan 
 
 s1WT4  10010.0 
     42.654         nan 
   1949.734         nan 
      0.945         nan 
   1046.700         nan 
      7.009         nan 
      0.972         nan 
 
 s1WT4  11010.0 
     44.450         nan 
   2109.620         nan 
      0.946         nan 
   1118.500         nan 
      7.041         nan 
      0.972         nan 
 
 s1WT4  12010.0 
     45.797         nan 
   2158.911         nan 
      0.949         nan 
   1162.100         nan 
      7.037         nan 
      0.972         nan 
 
 s1WT4  13010.0 
     47.490         nan 
   2280.896         nan 
      0.951         nan 
   1218.400         nan 
      7.054         nan 
      0.972         nan 
 
 s1WT4  14010.0 
     48.432         nan 
   2282.330         nan 
      0.954         nan 
   1257.700         nan 
      7.049         nan 
      0.972         nan 
 
 s1WT4  15010.0 
     49.436         nan 
   2354.932         nan 
      0.956         nan 
   1293.400         nan 
      7.054         nan 
      0.972         nan 
 
 s1WT4  16010.0 
     50.436         nan 
   2400.703         nan 
      0.958         nan 
   1341.500         nan 
      7.062         nan 
      0.972         nan 
 
 s1WT4  17010.0 
     51.944         nan 
   2478.721         nan 
      0.959         nan 
   1389.200         nan 
      7.069         nan 
      0.972         nan 
 
 s1WT4  18010.0 
     53.186         nan 
   2512.704         nan 
      0.961         nan 
   1429.700         nan 
      7.079         nan 
      0.972         nan 
 
 s1WT4  19010.0 
     54.353         nan 
   2531.442         nan 
      0.962         nan 
   1472.500         nan 
      7.088         nan 
      0.972         nan 
 
 s1WT4  20010.0 
     55.024         nan 
   2583.200         nan 
      0.964         nan 
   1499.800         nan 
      7.079         nan 
      0.972         nan 
 
 s1WT4  21010.0 
     55.861         nan 
   2626.861         nan 
      0.965         nan 
   1533.800         nan 
      7.079         nan 
      0.972         nan 
 
 s1WT4  22010.0 
     57.103         nan 
   2750.256         nan 
      0.965         nan 
   1569.600         nan 
      7.087         nan 
      0.972         nan 
 
 s1WT4  23010.0 
     57.752         nan 
   2717.412         nan 
      0.967         nan 
   1608.600         nan 
      7.094         nan 
      0.972         nan 
 
 s1WT4  24010.0 
     58.760         nan 
   2749.907         nan 
      0.968         nan 
   1639.900         nan 
      7.098         nan 
      0.972         nan 
 
 s1WT4  25010.0 
     59.838         nan 
   2809.941         nan 
      0.968         nan 
   1675.000         nan 
      7.100         nan 
      0.972         nan 
 
 s1WT4  26010.0 
     60.249         nan 
   2812.063         nan 
      0.970         nan 
   1698.400         nan 
      7.104         nan 
      0.972         nan 
 
 s1WT4  27010.0 
     60.995         nan 
   2841.954         nan 
      0.970         nan 
   1731.700         nan 
      7.108         nan 
      0.972         nan 
 
 s1WT5  10.0 
      1.795         nan 
     26.200         nan 
      0.280         nan 
      8.400         nan 
      2.972         nan 
      0.860         nan 
 
 s1WT5  1010.0 
     16.954         nan 
    728.520         nan 
      0.823         nan 
    275.600         nan 
      6.394         nan 
      0.963         nan 
 
 s1WT5  2010.0 
     22.334         nan 
   1032.376         nan 
      0.870         nan 
    417.300         nan 
      6.601         nan 
      0.964         nan 
 
 s1WT5  3010.0 
     26.538         nan 
   1285.812         nan 
      0.889         nan 
    543.500         nan 
      6.698         nan 
      0.964         nan 
 
 s1WT5  4010.0 
     29.575         nan 
   1409.611         nan 
      0.906         nan 
    636.300         nan 
      6.747         nan 
      0.964         nan 
 
 s1WT5  5010.0 
     32.718         nan 
   1629.509         nan 
      0.912         nan 
    742.800         nan 
      6.816         nan 
      0.965         nan 
 
 s1WT5  6010.0 
     35.205         nan 
   1738.716         nan 
      0.920         nan 
    821.300         nan 
      6.829         nan 
      0.964         nan 
 
 s1WT5  7010.0 
     37.095         nan 
   1855.073         nan 
      0.928         nan 
    883.100         nan 
      6.837         nan 
      0.964         nan 
 
 s1WT5  8010.0 
     39.194         nan 
   1939.001         nan 
      0.933         nan 
    952.100         nan 
      6.856         nan 
      0.964         nan 
 
 s1WT5  9010.0 
     41.110         nan 
   2051.121         nan 
      0.937         nan 
   1022.000         nan 
      6.877         nan 
      0.964         nan 
 
 s1WT5  10010.0 
     42.951         nan 
   2164.080         nan 
      0.940         nan 
   1090.400         nan 
      6.900         nan 
      0.965         nan 
 
 s1WT5  11010.0 
     44.517         nan 
   2191.132         nan 
      0.944         nan 
   1148.000         nan 
      6.913         nan 
      0.965         nan 
 
 s1WT5  12010.0 
     46.054         nan 
   2288.249         nan 
      0.946         nan 
   1204.600         nan 
      6.921         nan 
      0.964         nan 
 
 s1WT5  13010.0 
     47.506         nan 
   2373.541         nan 
      0.949         nan 
   1252.300         nan 
      6.934         nan 
      0.965         nan 
 
 s1WT5  14010.0 
     48.547         nan 
   2373.006         nan 
      0.952         nan 
   1303.300         nan 
      6.940         nan 
      0.965         nan 
 
 s1WT5  15010.0 
     49.847         nan 
   2414.464         nan 
      0.954         nan 
   1351.000         nan 
      6.943         nan 
      0.964         nan 
 
 s1WT5  16010.0 
     51.147         nan 
   2471.052         nan 
      0.956         nan 
   1386.900         nan 
      6.939         nan 
      0.964         nan 
 
 s1WT5  17010.0 
     52.134         nan 
   2559.312         nan 
      0.958         nan 
   1429.500         nan 
      6.950         nan 
      0.964         nan 
 
 s1WT5  18010.0 
     53.040         nan 
   2547.937         nan 
      0.959         nan 
   1470.900         nan 
      6.959         nan 
      0.964         nan 
 
 s1WT5  19010.0 
     54.358         nan 
   2626.475         nan 
      0.960         nan 
   1519.400         nan 
      6.962         nan 
      0.964         nan 
 
 s1WT5  20010.0 
     55.238         nan 
   2661.909         nan 
      0.962         nan 
   1557.100         nan 
      6.972         nan 
      0.965         nan 
 
 s1WT5  21010.0 
     56.474         nan 
   2754.397         nan 
      0.963         nan 
   1597.400         nan 
      6.976         nan 
      0.965         nan 
 
 s1WT5  22010.0 
     57.229         nan 
   2766.537         nan 
      0.964         nan 
   1632.100         nan 
      6.973         nan 
      0.964         nan 
 
 s1WT5  23010.0 
     58.024         nan 
   2819.443         nan 
      0.965         nan 
   1664.000         nan 
      6.977         nan 
      0.964         nan 
 
 s1WT5  24010.0 
     58.729         nan 
   2792.546         nan 
      0.967         nan 
   1698.000         nan 
      6.984         nan 
      0.964         nan 
 
 s1WT5  25010.0 
     59.601         nan 
   2886.763         nan 
      0.967         nan 
   1736.400         nan 
      6.985         nan 
      0.964         nan 
 
 s1WT5  26010.0 
     60.271         nan 
   2872.240         nan 
      0.968         nan 
   1760.800         nan 
      6.987         nan 
      0.964         nan 
 
 s1WT5  27010.0 
     61.027         nan 
   2917.085         nan 
      0.969         nan 
   1792.600         nan 
      6.988         nan 
      0.964         nan 
 
 s1WT6  10.0 
      1.781         nan 
     24.200         nan 
      0.220         nan 
      8.800         nan 
      3.067         nan 
      0.872         nan 
 
 s1WT6  1010.0 
     16.803         nan 
    691.501         nan 
      0.826         nan 
    275.900         nan 
      6.542         nan 
      0.969         nan 
 
 s1WT6  2010.0 
     22.480         nan 
   1069.976         nan 
      0.864         nan 
    436.200         nan 
      6.794         nan 
      0.970         nan 
 
 s1WT6  3010.0 
     26.932         nan 
   1368.508         nan 
      0.882         nan 
    569.100         nan 
      6.885         nan 
      0.970         nan 
 
 s1WT6  4010.0 
     30.131         nan 
   1498.518         nan 
      0.899         nan 
    667.200         nan 
      6.911         nan 
      0.970         nan 
 
 s1WT6  5010.0 
     32.970         nan 
   1663.492         nan 
      0.909         nan 
    764.700         nan 
      6.959         nan 
      0.969         nan 
 
 s1WT6  6010.0 
     35.124         nan 
   1742.820         nan 
      0.919         nan 
    850.300         nan 
      6.998         nan 
      0.970         nan 
 
 s1WT6  7010.0 
     37.230         nan 
   1842.580         nan 
      0.926         nan 
    919.500         nan 
      7.013         nan 
      0.970         nan 
 
 s1WT6  8010.0 
     40.068         nan 
   2066.826         nan 
      0.929         nan 
   1006.000         nan 
      7.049         nan 
      0.970         nan 
 
 s1WT6  9010.0 
     41.602         nan 
   2067.522         nan 
      0.934         nan 
   1071.600         nan 
      7.056         nan 
      0.970         nan 
 
 s1WT6  10010.0 
     43.459         nan 
   2176.151         nan 
      0.938         nan 
   1137.400         nan 
      7.068         nan 
      0.970         nan 
 
 s1WT6  11010.0 
     45.679         nan 
   2288.742         nan 
      0.941         nan 
   1204.200         nan 
      7.085         nan 
      0.970         nan 
 
 s1WT6  12010.0 
     46.622         nan 
   2258.025         nan 
      0.946         nan 
   1243.100         nan 
      7.095         nan 
      0.970         nan 
 
 s1WT6  13010.0 
     48.004         nan 
   2405.572         nan 
      0.948         nan 
   1300.200         nan 
      7.110         nan 
      0.970         nan 
 
 s1WT6  14010.0 
     49.166         nan 
   2364.178         nan 
      0.951         nan 
   1344.600         nan 
      7.111         nan 
      0.970         nan 
 
 s1WT6  15010.0 
     50.473         nan 
   2473.424         nan 
      0.953         nan 
   1401.300         nan 
      7.127         nan 
      0.970         nan 
 
 s1WT6  16010.0 
     51.636         nan 
   2529.393         nan 
      0.955         nan 
   1445.100         nan 
      7.123         nan 
      0.970         nan 
 
 s1WT6  17010.0 
     53.281         nan 
   2577.450         nan 
      0.956         nan 
   1502.000         nan 
      7.144         nan 
      0.970         nan 
 
 s1WT6  18010.0 
     53.877         nan 
   2721.902         nan 
      0.957         nan 
   1541.400         nan 
      7.140         nan 
      0.970         nan 
 
 s1WT6  19010.0 
     55.549         nan 
   2716.513         nan 
      0.959         nan 
   1592.400         nan 
      7.156         nan 
      0.970         nan 
 
 s1WT6  20010.0 
     55.900         nan 
   2674.798         nan 
      0.961         nan 
   1616.800         nan 
      7.151         nan 
      0.970         nan 
 
 s1WT6  21010.0 
     57.177         nan 
   2815.963         nan 
      0.962         nan 
   1664.700         nan 
      7.159         nan 
      0.970         nan 
 
 s1WT6  22010.0 
     58.265         nan 
   2848.709         nan 
      0.963         nan 
   1702.400         nan 
      7.165         nan 
      0.970         nan 
 
 s1WT6  23010.0 
     58.781         nan 
   2859.377         nan 
      0.965         nan 
   1727.900         nan 
      7.159         nan 
      0.970         nan 
 
 s1WT6  24010.0 
     59.746         nan 
   2873.315         nan 
      0.966         nan 
   1765.200         nan 
      7.164         nan 
      0.970         nan 
 
 s1WT6  25010.0 
     61.041         nan 
   2962.448         nan 
      0.966         nan 
   1809.400         nan 
      7.177         nan 
      0.970         nan 
 
 s1WT6  26010.0 
     61.319         nan 
   2983.403         nan 
      0.967         nan 
   1832.000         nan 
      7.169         nan 
      0.970         nan 
 
 s1WT6  27010.0 
     62.470         nan 
   3014.622         nan 
      0.968         nan 
   1866.900         nan 
      7.176         nan 
      0.970         nan 
 
 s1WT7  10.0 
      1.860         nan 
     30.050         nan 
      0.210         nan 
      8.900         nan 
      3.094         nan 
      0.876         nan 
 
 s1WT7  1010.0 
     16.485         nan 
    614.576         nan 
      0.840         nan 
    255.100         nan 
      6.245         nan 
      0.962         nan 
 
 s1WT7  2010.0 
     22.038         nan 
    969.597         nan 
      0.879         nan 
    399.600         nan 
      6.481         nan 
      0.964         nan 
 
 s1WT7  3010.0 
     26.205         nan 
   1167.616         nan 
      0.898         nan 
    512.100         nan 
      6.577         nan 
      0.964         nan 
 
 s1WT7  4010.0 
     29.014         nan 
   1336.133         nan 
      0.912         nan 
    599.700         nan 
      6.611         nan 
      0.964         nan 
 
 s1WT7  5010.0 
     31.588         nan 
   1427.506         nan 
      0.921         nan 
    685.600         nan 
      6.647         nan 
      0.963         nan 
 
 s1WT7  6010.0 
     33.788         nan 
   1533.530         nan 
      0.930         nan 
    756.200         nan 
      6.699         nan 
      0.965         nan 
 
 s1WT7  7010.0 
     36.251         nan 
   1759.301         nan 
      0.933         nan 
    830.700         nan 
      6.706         nan 
      0.964         nan 
 
 s1WT7  8010.0 
     37.470         nan 
   1752.889         nan 
      0.940         nan 
    880.200         nan 
      6.689         nan 
      0.963         nan 
 
 s1WT7  9010.0 
     39.686         nan 
   1864.094         nan 
      0.943         nan 
    947.300         nan 
      6.736         nan 
      0.964         nan 
 
 s1WT7  10010.0 
     41.062         nan 
   1840.221         nan 
      0.948         nan 
    996.200         nan 
      6.749         nan 
      0.964         nan 
 
 s1WT7  11010.0 
     42.637         nan 
   2025.110         nan 
      0.949         nan 
   1054.500         nan 
      6.755         nan 
      0.964         nan 
 
 s1WT7  12010.0 
     44.015         nan 
   2066.535         nan 
      0.952         nan 
   1104.900         nan 
      6.768         nan 
      0.964         nan 
 
 s1WT7  13010.0 
     45.639         nan 
   2223.681         nan 
      0.953         nan 
   1162.900         nan 
      6.781         nan 
      0.964         nan 
 
 s1WT7  14010.0 
     46.939         nan 
   2276.018         nan 
      0.955         nan 
   1212.800         nan 
      6.794         nan 
      0.964         nan 
 
 s1WT7  15010.0 
     48.410         nan 
   2305.088         nan 
      0.957         nan 
   1252.300         nan 
      6.798         nan 
      0.964         nan 
 
 s1WT7  16010.0 
     49.271         nan 
   2349.498         nan 
      0.959         nan 
   1288.200         nan 
      6.797         nan 
      0.964         nan 
 
 s1WT7  17010.0 
     50.248         nan 
   2394.215         nan 
      0.961         nan 
   1329.500         nan 
      6.815         nan 
      0.964         nan 
 
 s1WT7  18010.0 
     51.357         nan 
   2416.195         nan 
      0.962         nan 
   1364.300         nan 
      6.801         nan 
      0.964         nan 
 
 s1WT7  19010.0 
     52.125         nan 
   2486.050         nan 
      0.963         nan 
   1401.100         nan 
      6.815         nan 
      0.964         nan 
 
 s1WT7  20010.0 
     52.952         nan 
   2467.654         nan 
      0.965         nan 
   1432.100         nan 
      6.810         nan 
      0.964         nan 
 
 s1WT7  21010.0 
     53.815         nan 
   2522.487         nan 
      0.966         nan 
   1466.900         nan 
      6.826         nan 
      0.964         nan 
 
 s1WT7  22010.0 
     54.384         nan 
   2510.613         nan 
      0.968         nan 
   1498.200         nan 
      6.821         nan 
      0.964         nan 
 
 s1WT7  23010.0 
     55.731         nan 
   2614.973         nan 
      0.968         nan 
   1536.800         nan 
      6.825         nan 
      0.964         nan 
 
 s1WT7  24010.0 
     57.034         nan 
   2703.391         nan 
      0.968         nan 
   1579.400         nan 
      6.834         nan 
      0.964         nan 
 
 s1WT7  25010.0 
     57.382         nan 
   2709.045         nan 
      0.969         nan 
   1606.800         nan 
      6.837         nan 
      0.964         nan 
 
 s1WT7  26010.0 
     58.376         nan 
   2729.095         nan 
      0.970         nan 
   1639.200         nan 
      6.839         nan 
      0.964         nan 
 
 s1WT7  27010.0 
     58.742         nan 
   2767.438         nan 
      0.971         nan 
   1654.700         nan 
      6.839         nan 
      0.964         nan 
 
 s2KE1  10.0 
      2.216         nan 
     39.600         nan 
      0.110         nan 
      9.400         nan 
      3.194         nan 
      0.886         nan 
 
 s2KE1  1010.0 
     17.576         nan 
    587.739         nan 
      0.842         nan 
    268.200         nan 
      6.617         nan 
      0.974         nan 
 
 s2KE1  2010.0 
     23.608         nan 
   1005.278         nan 
      0.875         nan 
    422.500         nan 
      6.882         nan 
      0.975         nan 
 
 s2KE1  3010.0 
     27.107         nan 
   1160.560         nan 
      0.900         nan 
    520.600         nan 
      6.951         nan 
      0.976         nan 
 
 s2KE1  4010.0 
     30.516         nan 
   1282.642         nan 
      0.913         nan 
    617.700         nan 
      7.022         nan 
      0.976         nan 
 
 s2KE1  5010.0 
     32.892         nan 
   1401.663         nan 
      0.924         nan 
    694.600         nan 
      7.043         nan 
      0.976         nan 
 
 s2KE1  6010.0 
     35.713         nan 
   1614.045         nan 
      0.929         nan 
    775.400         nan 
      7.072         nan 
      0.976         nan 
 
 s2KE1  7010.0 
     38.288         nan 
   1746.825         nan 
      0.933         nan 
    849.600         nan 
      7.108         nan 
      0.976         nan 
 
 s2KE1  8010.0 
     39.697         nan 
   1806.800         nan 
      0.939         nan 
    906.300         nan 
      7.117         nan 
      0.976         nan 
 
 s2KE1  9010.0 
     41.545         nan 
   1897.225         nan 
      0.943         nan 
    962.100         nan 
      7.119         nan 
      0.976         nan 
 
 s2KE1  10010.0 
     42.900         nan 
   1935.432         nan 
      0.947         nan 
   1015.100         nan 
      7.152         nan 
      0.976         nan 
 
 s2KE1  11010.0 
     44.837         nan 
   2069.098         nan 
      0.949         nan 
   1075.400         nan 
      7.155         nan 
      0.976         nan 
 
 s2KE1  12010.0 
     45.923         nan 
   2093.782         nan 
      0.952         nan 
   1118.600         nan 
      7.163         nan 
      0.976         nan 
 
 s2KE1  13010.0 
     47.875         nan 
   2206.595         nan 
      0.954         nan 
   1167.300         nan 
      7.154         nan 
      0.975         nan 
 
 s2KE1  14010.0 
     48.765         nan 
   2276.302         nan 
      0.956         nan 
   1212.300         nan 
      7.171         nan 
      0.976         nan 
 
 s2KE1  15010.0 
     49.933         nan 
   2255.733         nan 
      0.958         nan 
   1257.400         nan 
      7.181         nan 
      0.976         nan 
 
 s2KE1  16010.0 
     51.505         nan 
   2347.387         nan 
      0.960         nan 
   1291.900         nan 
      7.178         nan 
      0.976         nan 
 
 s2KE1  17010.0 
     52.673         nan 
   2526.995         nan 
      0.960         nan 
   1346.300         nan 
      7.197         nan 
      0.976         nan 
 
 s2KE1  18010.0 
     53.742         nan 
   2477.457         nan 
      0.962         nan 
   1375.200         nan 
      7.198         nan 
      0.976         nan 
 
 s2KE1  19010.0 
     54.974         nan 
   2534.798         nan 
      0.963         nan 
   1422.000         nan 
      7.209         nan 
      0.976         nan 
 
 s2KE1  20010.0 
     56.129         nan 
   2611.314         nan 
      0.964         nan 
   1458.300         nan 
      7.217         nan 
      0.976         nan 
 
 s2KE1  21010.0 
     56.629         nan 
   2642.932         nan 
      0.965         nan 
   1487.800         nan 
      7.211         nan 
      0.976         nan 
 
 s2KE1  22010.0 
     57.733         nan 
   2700.581         nan 
      0.966         nan 
   1527.100         nan 
      7.213         nan 
      0.976         nan 
 
 s2KE1  23010.0 
     58.597         nan 
   2746.973         nan 
      0.967         nan 
   1553.700         nan 
      7.217         nan 
      0.976         nan 
 
 s2KE1  24010.0 
     59.251         nan 
   2788.348         nan 
      0.968         nan 
   1584.000         nan 
      7.224         nan 
      0.976         nan 
 
 s2KE1  25010.0 
     60.472         nan 
   2845.786         nan 
      0.969         nan 
   1628.800         nan 
      7.231         nan 
      0.976         nan 
 
 s2KE1  26010.0 
     61.034         nan 
   2880.238         nan 
      0.970         nan 
   1655.800         nan 
      7.230         nan 
      0.976         nan 
 
 s2KE1  27010.0 
     61.747         nan 
   2906.633         nan 
      0.970         nan 
   1686.200         nan 
      7.242         nan 
      0.976         nan 
 
 s2KE2  10.0 
      2.238         nan 
     31.200         nan 
      0.190         nan 
      9.000         nan 
      3.114         nan 
      0.878         nan 
 
 s2KE2  1010.0 
     18.869         nan 
    697.469         nan 
      0.824         nan 
    293.000         nan 
      6.947         nan 
      0.982         nan 
 
 s2KE2  2010.0 
     24.472         nan 
   1027.218         nan 
      0.869         nan 
    444.400         nan 
      7.161         nan 
      0.982         nan 
 
 s2KE2  3010.0 
     28.944         nan 
   1304.467         nan 
      0.890         nan 
    565.300         nan 
      7.265         nan 
      0.983         nan 
 
 s2KE2  4010.0 
     32.050         nan 
   1473.424         nan 
      0.905         nan 
    660.300         nan 
      7.293         nan 
      0.982         nan 
 
 s2KE2  5010.0 
     34.278         nan 
   1514.818         nan 
      0.918         nan 
    735.200         nan 
      7.310         nan 
      0.982         nan 
 
 s2KE2  6010.0 
     37.226         nan 
   1653.581         nan 
      0.925         nan 
    823.000         nan 
      7.358         nan 
      0.982         nan 
 
 s2KE2  7010.0 
     39.636         nan 
   1822.245         nan 
      0.929         nan 
    905.800         nan 
      7.392         nan 
      0.982         nan 
 
 s2KE2  8010.0 
     41.933         nan 
   1881.530         nan 
      0.935         nan 
    972.200         nan 
      7.411         nan 
      0.983         nan 
 
 s2KE2  9010.0 
     43.733         nan 
   2018.817         nan 
      0.938         nan 
   1041.200         nan 
      7.427         nan 
      0.982         nan 
 
 s2KE2  10010.0 
     44.893         nan 
   2133.645         nan 
      0.943         nan 
   1084.300         nan 
      7.424         nan 
      0.982         nan 
 
 s2KE2  11010.0 
     46.652         nan 
   2223.506         nan 
      0.944         nan 
   1150.300         nan 
      7.444         nan 
      0.983         nan 
 
 s2KE2  12010.0 
     48.319         nan 
   2288.163         nan 
      0.947         nan 
   1211.900         nan 
      7.461         nan 
      0.983         nan 
 
 s2KE2  13010.0 
     49.727         nan 
   2313.520         nan 
      0.951         nan 
   1249.700         nan 
      7.456         nan 
      0.982         nan 
 
 s2KE2  14010.0 
     51.065         nan 
   2456.204         nan 
      0.952         nan 
   1306.400         nan 
      7.474         nan 
      0.982         nan 
 
 s2KE2  15010.0 
     52.498         nan 
   2487.216         nan 
      0.954         nan 
   1355.400         nan 
      7.489         nan 
      0.983         nan 
 
 s2KE2  16010.0 
     53.461         nan 
   2595.180         nan 
      0.956         nan 
   1399.900         nan 
      7.490         nan 
      0.982         nan 
 
 s2KE2  17010.0 
     54.888         nan 
   2560.846         nan 
      0.958         nan 
   1440.700         nan 
      7.500         nan 
      0.983         nan 
 
 s2KE2  18010.0 
     55.740         nan 
   2600.412         nan 
      0.959         nan 
   1476.000         nan 
      7.493         nan 
      0.982         nan 
 
 s2KE2  19010.0 
     57.147         nan 
   2684.229         nan 
      0.960         nan 
   1525.800         nan 
      7.508         nan 
      0.983         nan 
 
 s2KE2  20010.0 
     58.142         nan 
   2759.570         nan 
      0.962         nan 
   1561.800         nan 
      7.508         nan 
      0.982         nan 
 
 s2KE2  21010.0 
     58.899         nan 
   2777.682         nan 
      0.963         nan 
   1599.100         nan 
      7.508         nan 
      0.982         nan 
 
 s2KE2  22010.0 
     59.934         nan 
   2848.627         nan 
      0.964         nan 
   1636.700         nan 
      7.522         nan 
      0.983         nan 
 
 s2KE2  23010.0 
     60.867         nan 
   2896.279         nan 
      0.965         nan 
   1665.100         nan 
      7.515         nan 
      0.983         nan 
 
 s2KE2  24010.0 
     62.090         nan 
   2888.594         nan 
      0.966         nan 
   1709.900         nan 
      7.527         nan 
      0.983         nan 
 
 s2KE2  25010.0 
     62.478         nan 
   2958.923         nan 
      0.967         nan 
   1732.900         nan 
      7.522         nan 
      0.982         nan 
 
 s2KE2  26010.0 
     63.963         nan 
   2982.866         nan 
      0.968         nan 
   1773.600         nan 
      7.535         nan 
      0.983         nan 
 
 s2KE2  27010.0 
     64.555         nan 
   3048.593         nan 
      0.969         nan 
   1807.800         nan 
      7.539         nan 
      0.983         nan 
 
 s2KE3  10.0 
      2.026         nan 
     48.200         nan 
      0.070         nan 
      9.600         nan 
      3.234         nan 
      0.890         nan 
 
 s2KE3  1010.0 
     17.621         nan 
    758.367         nan 
      0.825         nan 
    276.700         nan 
      6.652         nan 
      0.974         nan 
 
 s2KE3  2010.0 
     22.984         nan 
   1038.806         nan 
      0.872         nan 
    417.500         nan 
      6.819         nan 
      0.974         nan 
 
 s2KE3  3010.0 
     27.309         nan 
   1313.873         nan 
      0.889         nan 
    542.600         nan 
      6.913         nan 
      0.974         nan 
 
 s2KE3  4010.0 
     30.649         nan 
   1501.810         nan 
      0.902         nan 
    651.800         nan 
      6.981         nan 
      0.974         nan 
 
 s2KE3  5010.0 
     33.567         nan 
   1660.716         nan 
      0.912         nan 
    741.100         nan 
      7.016         nan 
      0.974         nan 
 
 s2KE3  6010.0 
     35.966         nan 
   1745.593         nan 
      0.920         nan 
    830.100         nan 
      7.073         nan 
      0.974         nan 
 
 s2KE3  7010.0 
     38.225         nan 
   1873.507         nan 
      0.926         nan 
    902.700         nan 
      7.079         nan 
      0.974         nan 
 
 s2KE3  8010.0 
     40.198         nan 
   2004.232         nan 
      0.932         nan 
    969.300         nan 
      7.095         nan 
      0.974         nan 
 
 s2KE3  9010.0 
     42.559         nan 
   2127.890         nan 
      0.935         nan 
   1050.100         nan 
      7.129         nan 
      0.974         nan 
 
 s2KE3  10010.0 
     44.016         nan 
   2187.041         nan 
      0.939         nan 
   1101.800         nan 
      7.133         nan 
      0.974         nan 
 
 s2KE3  11010.0 
     45.703         nan 
   2219.209         nan 
      0.942         nan 
   1163.900         nan 
      7.139         nan 
      0.974         nan 
 
 s2KE3  12010.0 
     47.351         nan 
   2369.608         nan 
      0.944         nan 
   1230.600         nan 
      7.167         nan 
      0.974         nan 
 
 s2KE3  13010.0 
     48.762         nan 
   2413.286         nan 
      0.947         nan 
   1284.000         nan 
      7.178         nan 
      0.974         nan 
 
 s2KE3  14010.0 
     49.717         nan 
   2454.974         nan 
      0.950         nan 
   1332.700         nan 
      7.165         nan 
      0.974         nan 
 
 s2KE3  15010.0 
     51.004         nan 
   2508.602         nan 
      0.952         nan 
   1378.200         nan 
      7.184         nan 
      0.974         nan 
 
 s2KE3  16010.0 
     52.612         nan 
   2612.624         nan 
      0.953         nan 
   1432.700         nan 
      7.190         nan 
      0.974         nan 
 
 s2KE3  17010.0 
     53.626         nan 
   2676.768         nan 
      0.955         nan 
   1473.800         nan 
      7.200         nan 
      0.975         nan 
 
 s2KE3  18010.0 
     55.179         nan 
   2757.956         nan 
      0.956         nan 
   1526.600         nan 
      7.208         nan 
      0.974         nan 
 
 s2KE3  19010.0 
     56.146         nan 
   2803.925         nan 
      0.958         nan 
   1563.700         nan 
      7.207         nan 
      0.974         nan 
 
 s2KE3  20010.0 
     56.845         nan 
   2731.223         nan 
      0.960         nan 
   1591.400         nan 
      7.208         nan 
      0.974         nan 
 
 s2KE3  21010.0 
     58.154         nan 
   2897.079         nan 
      0.961         nan 
   1651.400         nan 
      7.218         nan 
      0.974         nan 
 
 s2KE3  22010.0 
     58.526         nan 
   2895.874         nan 
      0.962         nan 
   1675.800         nan 
      7.219         nan 
      0.974         nan 
 
 s2KE3  23010.0 
     59.866         nan 
   2912.263         nan 
      0.963         nan 
   1714.200         nan 
      7.222         nan 
      0.974         nan 
 
 s2KE3  24010.0 
     60.452         nan 
   2893.741         nan 
      0.965         nan 
   1751.900         nan 
      7.230         nan 
      0.974         nan 
 
 s2KE3  25010.0 
     61.343         nan 
   3021.946         nan 
      0.965         nan 
   1784.200         nan 
      7.228         nan 
      0.974         nan 
 
 s2KE3  26010.0 
     62.074         nan 
   2998.813         nan 
      0.967         nan 
   1816.000         nan 
      7.230         nan 
      0.974         nan 
 
 s2KE3  27010.0 
     63.046         nan 
   3027.285         nan 
      0.968         nan 
   1852.300         nan 
      7.235         nan 
      0.974         nan 
 
 s2KE4  10.0 
      1.858         nan 
     35.400         nan 
      0.150         nan 
      9.200         nan 
      3.154         nan 
      0.882         nan 
 
 s2KE4  1010.0 
     17.075         nan 
    673.048         nan 
      0.834         nan 
    268.300         nan 
      6.549         nan 
      0.972         nan 
 
 s2KE4  2010.0 
     23.296         nan 
   1057.668         nan 
      0.868         nan 
    423.600         nan 
      6.784         nan 
      0.973         nan 
 
 s2KE4  3010.0 
     27.484         nan 
   1261.178         nan 
      0.892         nan 
    534.000         nan 
      6.868         nan 
      0.973         nan 
 
 s2KE4  4010.0 
     31.170         nan 
   1475.143         nan 
      0.905         nan 
    632.800         nan 
      6.924         nan 
      0.973         nan 
 
 s2KE4  5010.0 
     34.094         nan 
   1607.907         nan 
      0.914         nan 
    727.800         nan 
      6.952         nan 
      0.972         nan 
 
 s2KE4  6010.0 
     36.413         nan 
   1771.641         nan 
      0.921         nan 
    812.200         nan 
      7.003         nan 
      0.973         nan 
 
 s2KE4  7010.0 
     38.731         nan 
   1864.304         nan 
      0.928         nan 
    878.300         nan 
      7.019         nan 
      0.973         nan 
 
 s2KE4  8010.0 
     40.829         nan 
   1938.234         nan 
      0.933         nan 
    952.800         nan 
      7.037         nan 
      0.973         nan 
 
 s2KE4  9010.0 
     42.373         nan 
   2029.385         nan 
      0.938         nan 
   1005.200         nan 
      7.039         nan 
      0.973         nan 
 
 s2KE4  10010.0 
     45.110         nan 
   2234.205         nan 
      0.939         nan 
   1090.400         nan 
      7.082         nan 
      0.974         nan 
 
 s2KE4  11010.0 
     46.461         nan 
   2272.814         nan 
      0.942         nan 
   1146.600         nan 
      7.088         nan 
      0.973         nan 
 
 s2KE4  12010.0 
     48.184         nan 
   2345.759         nan 
      0.945         nan 
   1197.700         nan 
      7.093         nan 
      0.973         nan 
 
 s2KE4  13010.0 
     49.875         nan 
   2345.324         nan 
      0.949         nan 
   1246.300         nan 
      7.100         nan 
      0.973         nan 
 
 s2KE4  14010.0 
     50.951         nan 
   2444.969         nan 
      0.951         nan 
   1294.700         nan 
      7.105         nan 
      0.973         nan 
 
 s2KE4  15010.0 
     52.809         nan 
   2526.971         nan 
      0.952         nan 
   1357.400         nan 
      7.125         nan 
      0.974         nan 
 
 s2KE4  16010.0 
     53.317         nan 
   2594.746         nan 
      0.955         nan 
   1388.100         nan 
      7.121         nan 
      0.973         nan 
 
 s2KE4  17010.0 
     55.100         nan 
   2623.026         nan 
      0.956         nan 
   1447.300         nan 
      7.136         nan 
      0.973         nan 
 
 s2KE4  18010.0 
     55.618         nan 
   2629.274         nan 
      0.958         nan 
   1474.100         nan 
      7.124         nan 
      0.973         nan 
 
 s2KE4  19010.0 
     56.769         nan 
   2704.325         nan 
      0.959         nan 
   1514.000         nan 
      7.131         nan 
      0.973         nan 
 
 s2KE4  20010.0 
     57.915         nan 
   2709.080         nan 
      0.961         nan 
   1556.400         nan 
      7.142         nan 
      0.973         nan 
 
 s2KE4  21010.0 
     58.932         nan 
   2802.003         nan 
      0.962         nan 
   1600.800         nan 
      7.143         nan 
      0.973         nan 
 
 s2KE4  22010.0 
     60.151         nan 
   2884.699         nan 
      0.963         nan 
   1639.600         nan 
      7.148         nan 
      0.973         nan 
 
 s2KE4  23010.0 
     60.830         nan 
   2845.775         nan 
      0.964         nan 
   1677.000         nan 
      7.159         nan 
      0.973         nan 
 
 s2KE4  24010.0 
     61.866         nan 
   2919.626         nan 
      0.965         nan 
   1714.300         nan 
      7.163         nan 
      0.973         nan 
 
 s2KE4  25010.0 
     63.095         nan 
   2938.140         nan 
      0.966         nan 
   1750.200         nan 
      7.163         nan 
      0.973         nan 
 
 s2KE4  26010.0 
     63.361         nan 
   2940.920         nan 
      0.967         nan 
   1770.300         nan 
      7.163         nan 
      0.973         nan 
 
 s2KE4  27010.0 
     64.448         nan 
   3044.409         nan 
      0.968         nan 
   1812.200         nan 
      7.167         nan 
      0.973         nan 
 
 s2KE5  10.0 
      2.217         nan 
     41.200         nan 
      0.100         nan 
      9.500         nan 
      3.222         nan 
      0.890         nan 
 
 s2KE5  1010.0 
     17.852         nan 
    668.025         nan 
      0.832         nan 
    272.800         nan 
      6.654         nan 
      0.977         nan 
 
 s2KE5  2010.0 
     23.198         nan 
    862.372         nan 
      0.884         nan 
    407.800         nan 
      6.851         nan 
      0.977         nan 
 
 s2KE5  3010.0 
     27.075         nan 
   1116.503         nan 
      0.903         nan 
    512.200         nan 
      6.891         nan 
      0.977         nan 
 
 s2KE5  4010.0 
     30.716         nan 
   1385.072         nan 
      0.912         nan 
    614.000         nan 
      6.982         nan 
      0.977         nan 
 
 s2KE5  5010.0 
     33.628         nan 
   1550.232         nan 
      0.919         nan 
    708.200         nan 
      7.021         nan 
      0.977         nan 
 
 s2KE5  6010.0 
     35.349         nan 
   1631.480         nan 
      0.929         nan 
    762.200         nan 
      7.028         nan 
      0.977         nan 
 
 s2KE5  7010.0 
     37.898         nan 
   1773.358         nan 
      0.934         nan 
    838.600         nan 
      7.053         nan 
      0.977         nan 
 
 s2KE5  8010.0 
     39.921         nan 
   1941.251         nan 
      0.937         nan 
    904.900         nan 
      7.064         nan 
      0.977         nan 
 
 s2KE5  9010.0 
     41.806         nan 
   2006.458         nan 
      0.941         nan 
    966.600         nan 
      7.089         nan 
      0.977         nan 
 
 s2KE5  10010.0 
     43.209         nan 
   2055.873         nan 
      0.945         nan 
   1017.600         nan 
      7.101         nan 
      0.977         nan 
 
 s2KE5  11010.0 
     44.990         nan 
   2130.233         nan 
      0.948         nan 
   1070.300         nan 
      7.114         nan 
      0.977         nan 
 
 s2KE5  12010.0 
     47.368         nan 
   2284.915         nan 
      0.949         nan 
   1130.900         nan 
      7.133         nan 
      0.977         nan 
 
 s2KE5  13010.0 
     47.897         nan 
   2298.774         nan 
      0.952         nan 
   1171.800         nan 
      7.131         nan 
      0.977         nan 
 
 s2KE5  14010.0 
     49.353         nan 
   2356.124         nan 
      0.954         nan 
   1222.600         nan 
      7.143         nan 
      0.977         nan 
 
 s2KE5  15010.0 
     50.674         nan 
   2421.370         nan 
      0.956         nan 
   1262.400         nan 
      7.137         nan 
      0.977         nan 
 
 s2KE5  16010.0 
     52.331         nan 
   2441.967         nan 
      0.957         nan 
   1313.900         nan 
      7.150         nan 
      0.977         nan 
 
 s2KE5  17010.0 
     53.397         nan 
   2533.430         nan 
      0.959         nan 
   1353.100         nan 
      7.158         nan 
      0.977         nan 
 
 s2KE5  18010.0 
     54.261         nan 
   2528.931         nan 
      0.961         nan 
   1387.900         nan 
      7.159         nan 
      0.977         nan 
 
 s2KE5  19010.0 
     55.210         nan 
   2586.935         nan 
      0.962         nan 
   1427.100         nan 
      7.160         nan 
      0.977         nan 
 
 s2KE5  20010.0 
     56.331         nan 
   2686.399         nan 
      0.963         nan 
   1468.600         nan 
      7.170         nan 
      0.977         nan 
 
 s2KE5  21010.0 
     57.526         nan 
   2687.446         nan 
      0.964         nan 
   1502.900         nan 
      7.176         nan 
      0.977         nan 
 
 s2KE5  22010.0 
     58.452         nan 
   2739.673         nan 
      0.965         nan 
   1540.900         nan 
      7.179         nan 
      0.977         nan 
 
 s2KE5  23010.0 
     59.532         nan 
   2696.807         nan 
      0.966         nan 
   1573.500         nan 
      7.191         nan 
      0.977         nan 
 
 s2KE5  24010.0 
     60.228         nan 
   2835.250         nan 
      0.967         nan 
   1603.500         nan 
      7.183         nan 
      0.977         nan 
 
 s2KE5  25010.0 
     61.232         nan 
   2843.334         nan 
      0.968         nan 
   1636.600         nan 
      7.182         nan 
      0.977         nan 
 
 s2KE5  26010.0 
     61.712         nan 
   2819.067         nan 
      0.969         nan 
   1669.000         nan 
      7.188         nan 
      0.977         nan 
 
 s2KE5  27010.0 
     63.086         nan 
   2926.064         nan 
      0.969         nan 
   1711.600         nan 
      7.202         nan 
      0.977         nan 
 
 s2KE6  10.0 
      1.962         nan 
     29.400         nan 
      0.220         nan 
      8.800         nan 
      3.067         nan 
      0.872         nan 
 
 s2KE6  1010.0 
     18.174         nan 
    653.472         nan 
      0.836         nan 
    283.000         nan 
      7.027         nan 
      0.985         nan 
 
 s2KE6  2010.0 
     23.622         nan 
    989.484         nan 
      0.879         nan 
    420.800         nan 
      7.225         nan 
      0.986         nan 
 
 s2KE6  3010.0 
     27.865         nan 
   1167.483         nan 
      0.899         nan 
    535.400         nan 
      7.322         nan 
      0.986         nan 
 
 s2KE6  4010.0 
     30.836         nan 
   1346.041         nan 
      0.913         nan 
    618.400         nan 
      7.361         nan 
      0.986         nan 
 
 s2KE6  5010.0 
     33.782         nan 
   1490.304         nan 
      0.922         nan 
    704.300         nan 
      7.420         nan 
      0.986         nan 
 
 s2KE6  6010.0 
     36.270         nan 
   1683.502         nan 
      0.926         nan 
    793.500         nan 
      7.442         nan 
      0.986         nan 
 
 s2KE6  7010.0 
     38.957         nan 
   1740.203         nan 
      0.932         nan 
    869.600         nan 
      7.495         nan 
      0.986         nan 
 
 s2KE6  8010.0 
     40.707         nan 
   1950.813         nan 
      0.936         nan 
    925.400         nan 
      7.498         nan 
      0.986         nan 
 
 s2KE6  9010.0 
     42.832         nan 
   1954.618         nan 
      0.940         nan 
    993.200         nan 
      7.505         nan 
      0.986         nan 
 
 s2KE6  10010.0 
     44.429         nan 
   2036.859         nan 
      0.944         nan 
   1043.900         nan 
      7.530         nan 
      0.986         nan 
 
 s2KE6  11010.0 
     45.733         nan 
   2159.814         nan 
      0.947         nan 
   1092.400         nan 
      7.530         nan 
      0.986         nan 
 
 s2KE6  12010.0 
     47.384         nan 
   2209.907         nan 
      0.949         nan 
   1147.600         nan 
      7.541         nan 
      0.986         nan 
 
 s2KE6  13010.0 
     48.871         nan 
   2237.554         nan 
      0.952         nan 
   1189.000         nan 
      7.539         nan 
      0.986         nan 
 
 s2KE6  14010.0 
     50.188         nan 
   2298.513         nan 
      0.954         nan 
   1244.300         nan 
      7.561         nan 
      0.986         nan 
 
 s2KE6  15010.0 
     50.991         nan 
   2351.714         nan 
      0.957         nan 
   1279.000         nan 
      7.565         nan 
      0.986         nan 
 
 s2KE6  16010.0 
     52.414         nan 
   2361.512         nan 
      0.958         nan 
   1325.700         nan 
      7.566         nan 
      0.986         nan 
 
 s2KE6  17010.0 
     53.659         nan 
   2480.409         nan 
      0.959         nan 
   1371.500         nan 
      7.573         nan 
      0.986         nan 
 
 s2KE6  18010.0 
     55.018         nan 
   2431.060         nan 
      0.962         nan 
   1399.500         nan 
      7.580         nan 
      0.986         nan 
 
 s2KE6  19010.0 
     55.547         nan 
   2505.160         nan 
      0.963         nan 
   1440.200         nan 
      7.579         nan 
      0.986         nan 
 
 s2KE6  20010.0 
     56.693         nan 
   2542.183         nan 
      0.964         nan 
   1486.800         nan 
      7.590         nan 
      0.986         nan 
 
 s2KE6  21010.0 
     57.911         nan 
   2624.828         nan 
      0.965         nan 
   1522.000         nan 
      7.598         nan 
      0.986         nan 
 
 s2KE6  22010.0 
     58.943         nan 
   2687.268         nan 
      0.966         nan 
   1557.500         nan 
      7.597         nan 
      0.986         nan 
 
 s2KE6  23010.0 
     59.411         nan 
   2673.767         nan 
      0.967         nan 
   1585.700         nan 
      7.598         nan 
      0.986         nan 
 
 s2KE6  24010.0 
     60.294         nan 
   2760.916         nan 
      0.968         nan 
   1618.700         nan 
      7.599         nan 
      0.986         nan 
 
 s2KE6  25010.0 
     61.030         nan 
   2735.867         nan 
      0.969         nan 
   1643.000         nan 
      7.607         nan 
      0.986         nan 
 
 s2KE6  26010.0 
     61.950         nan 
   2740.415         nan 
      0.970         nan 
   1672.100         nan 
      7.606         nan 
      0.986         nan 
 
 s2KE6  27010.0 
     62.711         nan 
   2765.223         nan 
      0.971         nan 
   1704.200         nan 
      7.607         nan 
      0.986         nan 
 
 s2KO1  10.0 
      2.000         nan 
     35.900         nan 
      0.180         nan 
      8.900         nan 
      3.067         nan 
      0.868         nan 
 
 s2KO1  1010.0 
     17.398         nan 
    635.322         nan 
      0.836         nan 
    265.300         nan 
      6.499         nan 
      0.970         nan 
 
 s2KO1  2010.0 
     22.639         nan 
    943.691         nan 
      0.879         nan 
    401.100         nan 
      6.680         nan 
      0.971         nan 
 
 s2KO1  3010.0 
     26.510         nan 
   1137.809         nan 
      0.901         nan 
    509.400         nan 
      6.776         nan 
      0.971         nan 
 
 s2KO1  4010.0 
     29.569         nan 
   1365.900         nan 
      0.912         nan 
    600.100         nan 
      6.804         nan 
      0.970         nan 
 
 s2KO1  5010.0 
     32.411         nan 
   1521.920         nan 
      0.920         nan 
    689.300         nan 
      6.856         nan 
      0.970         nan 
 
 s2KO1  6010.0 
     34.507         nan 
   1606.353         nan 
      0.928         nan 
    760.400         nan 
      6.875         nan 
      0.971         nan 
 
 s2KO1  7010.0 
     36.963         nan 
   1672.174         nan 
      0.934         nan 
    825.800         nan 
      6.898         nan 
      0.971         nan 
 
 s2KO1  8010.0 
     39.115         nan 
   1824.845         nan 
      0.938         nan 
    895.600         nan 
      6.930         nan 
      0.971         nan 
 
 s2KO1  9010.0 
     40.704         nan 
   1885.388         nan 
      0.942         nan 
    948.800         nan 
      6.922         nan 
      0.970         nan 
 
 s2KO1  10010.0 
     42.512         nan 
   2012.739         nan 
      0.945         nan 
   1010.800         nan 
      6.934         nan 
      0.970         nan 
 
 s2KO1  11010.0 
     43.881         nan 
   2060.455         nan 
      0.948         nan 
   1061.900         nan 
      6.946         nan 
      0.970         nan 
 
 s2KO1  12010.0 
     45.301         nan 
   2132.559         nan 
      0.950         nan 
   1121.300         nan 
      6.966         nan 
      0.970         nan 
 
 s2KO1  13010.0 
     46.511         nan 
   2190.206         nan 
      0.953         nan 
   1162.100         nan 
      6.970         nan 
      0.971         nan 
 
 s2KO1  14010.0 
     47.883         nan 
   2212.508         nan 
      0.956         nan 
   1198.200         nan 
      6.968         nan 
      0.970         nan 
 
 s2KO1  15010.0 
     49.315         nan 
   2273.690         nan 
      0.957         nan 
   1250.100         nan 
      6.979         nan 
      0.970         nan 
 
 s2KO1  16010.0 
     50.732         nan 
   2402.975         nan 
      0.958         nan 
   1305.000         nan 
      7.005         nan 
      0.971         nan 
 
 s2KO1  17010.0 
     51.363         nan 
   2504.789         nan 
      0.959         nan 
   1338.500         nan 
      6.992         nan 
      0.970         nan 
 
 s2KO1  18010.0 
     52.695         nan 
   2502.814         nan 
      0.961         nan 
   1381.700         nan 
      7.000         nan 
      0.970         nan 
 
 s2KO1  19010.0 
     54.041         nan 
   2534.494         nan 
      0.963         nan 
   1420.400         nan 
      7.006         nan 
      0.970         nan 
 
 s2KO1  20010.0 
     54.798         nan 
   2593.929         nan 
      0.964         nan 
   1446.300         nan 
      7.008         nan 
      0.970         nan 
 
 s2KO1  21010.0 
     55.819         nan 
   2678.740         nan 
      0.965         nan 
   1487.000         nan 
      7.004         nan 
      0.970         nan 
 
 s2KO1  22010.0 
     56.459         nan 
   2727.388         nan 
      0.966         nan 
   1515.100         nan 
      7.007         nan 
      0.970         nan 
 
 s2KO1  23010.0 
     57.652         nan 
   2715.970         nan 
      0.967         nan 
   1557.900         nan 
      7.023         nan 
      0.971         nan 
 
 s2KO1  24010.0 
     58.317         nan 
   2814.268         nan 
      0.968         nan 
   1584.800         nan 
      7.022         nan 
      0.970         nan 
 
 s2KO1  25010.0 
     59.497         nan 
   2812.678         nan 
      0.969         nan 
   1618.200         nan 
      7.021         nan 
      0.970         nan 
 
 s2KO1  26010.0 
     60.316         nan 
   2851.550         nan 
      0.969         nan 
   1652.500         nan 
      7.030         nan 
      0.970         nan 
 
 s2KO1  27010.0 
     60.939         nan 
   2869.070         nan 
      0.971         nan 
   1667.500         nan 
      7.024         nan 
      0.970         nan 
 
 s2KO2  10.0 
      1.977         nan 
     33.700         nan 
      0.180         nan 
      9.000         nan 
      3.102         nan 
      0.874         nan 
 
 s2KO2  1010.0 
     17.448         nan 
    659.630         nan 
      0.844         nan 
    259.400         nan 
      6.647         nan 
      0.976         nan 
 
 s2KO2  2010.0 
     22.727         nan 
   1031.655         nan 
      0.880         nan 
    399.500         nan 
      6.857         nan 
      0.977         nan 
 
 s2KO2  3010.0 
     26.872         nan 
   1151.651         nan 
      0.903         nan 
    499.400         nan 
      6.927         nan 
      0.977         nan 
 
 s2KO2  4010.0 
     29.572         nan 
   1336.978         nan 
      0.914         nan 
    588.500         nan 
      6.946         nan 
      0.976         nan 
 
 s2KO2  5010.0 
     32.828         nan 
   1539.364         nan 
      0.921         nan 
    677.100         nan 
      7.015         nan 
      0.977         nan 
 
 s2KO2  6010.0 
     35.555         nan 
   1673.424         nan 
      0.928         nan 
    745.700         nan 
      7.020         nan 
      0.977         nan 
 
 s2KO2  7010.0 
     37.375         nan 
   1801.340         nan 
      0.933         nan 
    812.000         nan 
      7.021         nan 
      0.976         nan 
 
 s2KO2  8010.0 
     39.319         nan 
   1891.988         nan 
      0.937         nan 
    891.700         nan 
      7.079         nan 
      0.977         nan 
 
 s2KO2  9010.0 
     40.993         nan 
   1962.800         nan 
      0.941         nan 
    943.700         nan 
      7.089         nan 
      0.977         nan 
 
 s2KO2  10010.0 
     43.010         nan 
   2083.044         nan 
      0.943         nan 
   1010.800         nan 
      7.096         nan 
      0.976         nan 
 
 s2KO2  11010.0 
     44.877         nan 
   2176.525         nan 
      0.946         nan 
   1065.300         nan 
      7.107         nan 
      0.977         nan 
 
 s2KO2  12010.0 
     46.071         nan 
   2210.449         nan 
      0.949         nan 
   1117.400         nan 
      7.133         nan 
      0.977         nan 
 
 s2KO2  13010.0 
     47.212         nan 
   2271.372         nan 
      0.951         nan 
   1159.900         nan 
      7.116         nan 
      0.977         nan 
 
 s2KO2  14010.0 
     48.644         nan 
   2384.920         nan 
      0.953         nan 
   1203.800         nan 
      7.125         nan 
      0.977         nan 
 
 s2KO2  15010.0 
     50.175         nan 
   2353.937         nan 
      0.955         nan 
   1260.800         nan 
      7.141         nan 
      0.977         nan 
 
 s2KO2  16010.0 
     51.118         nan 
   2431.289         nan 
      0.957         nan 
   1302.400         nan 
      7.151         nan 
      0.977         nan 
 
 s2KO2  17010.0 
     52.351         nan 
   2488.424         nan 
      0.958         nan 
   1342.700         nan 
      7.143         nan 
      0.977         nan 
 
 s2KO2  18010.0 
     53.587         nan 
   2545.535         nan 
      0.960         nan 
   1387.600         nan 
      7.157         nan 
      0.977         nan 
 
 s2KO2  19010.0 
     54.961         nan 
   2588.107         nan 
      0.961         nan 
   1426.600         nan 
      7.160         nan 
      0.977         nan 
 
 s2KO2  20010.0 
     55.472         nan 
   2578.084         nan 
      0.963         nan 
   1453.300         nan 
      7.162         nan 
      0.977         nan 
 
 s2KO2  21010.0 
     56.720         nan 
   2683.439         nan 
      0.963         nan 
   1502.900         nan 
      7.171         nan 
      0.977         nan 
 
 s2KO2  22010.0 
     57.708         nan 
   2682.397         nan 
      0.965         nan 
   1540.500         nan 
      7.177         nan 
      0.977         nan 
 
 s2KO2  23010.0 
     58.476         nan 
   2723.364         nan 
      0.966         nan 
   1577.400         nan 
      7.179         nan 
      0.977         nan 
 
 s2KO2  24010.0 
     59.360         nan 
   2807.811         nan 
      0.967         nan 
   1604.100         nan 
      7.182         nan 
      0.977         nan 
 
 s2KO2  25010.0 
     60.423         nan 
   2807.452         nan 
      0.968         nan 
   1637.200         nan 
      7.182         nan 
      0.977         nan 
 
 s2KO2  26010.0 
     60.755         nan 
   2851.947         nan 
      0.968         nan 
   1671.500         nan 
      7.187         nan 
      0.977         nan 
 
 s2KO2  27010.0 
     61.627         nan 
   2811.411         nan 
      0.970         nan 
   1692.800         nan 
      7.189         nan 
      0.977         nan 
 
 s2KO3  10.0 
      2.173         nan 
     35.400         nan 
      0.150         nan 
      9.200         nan 
      3.154         nan 
      0.882         nan 
 
 s2KO3  1010.0 
     17.658         nan 
    644.249         nan 
      0.842         nan 
    261.500         nan 
      6.564         nan 
      0.975         nan 
 
 s2KO3  2010.0 
     22.718         nan 
    856.659         nan 
      0.888         nan 
    389.800         nan 
      6.721         nan 
      0.976         nan 
 
 s2KO3  3010.0 
     26.455         nan 
   1053.352         nan 
      0.907         nan 
    494.800         nan 
      6.822         nan 
      0.976         nan 
 
 s2KO3  4010.0 
     29.409         nan 
   1207.823         nan 
      0.919         nan 
    580.800         nan 
      6.890         nan 
      0.976         nan 
 
 s2KO3  5010.0 
     32.589         nan 
   1407.777         nan 
      0.925         nan 
    671.500         nan 
      6.933         nan 
      0.976         nan 
 
 s2KO3  6010.0 
     34.618         nan 
   1583.954         nan 
      0.932         nan 
    733.700         nan 
      6.952         nan 
      0.976         nan 
 
 s2KO3  7010.0 
     36.124         nan 
   1674.699         nan 
      0.938         nan 
    791.300         nan 
      6.951         nan 
      0.976         nan 
 
 s2KO3  8010.0 
     38.629         nan 
   1778.574         nan 
      0.941         nan 
    863.900         nan 
      6.991         nan 
      0.976         nan 
 
 s2KO3  9010.0 
     39.713         nan 
   1840.096         nan 
      0.945         nan 
    907.900         nan 
      6.996         nan 
      0.976         nan 
 
 s2KO3  10010.0 
     41.969         nan 
   1965.465         nan 
      0.947         nan 
    977.800         nan 
      7.000         nan 
      0.976         nan 
 
 s2KO3  11010.0 
     42.366         nan 
   1974.954         nan 
      0.952         nan 
   1007.600         nan 
      7.011         nan 
      0.976         nan 
 
 s2KO3  12010.0 
     44.253         nan 
   2043.300         nan 
      0.953         nan 
   1064.500         nan 
      7.029         nan 
      0.976         nan 
 
 s2KO3  13010.0 
     45.888         nan 
   2139.317         nan 
      0.955         nan 
   1117.100         nan 
      7.036         nan 
      0.976         nan 
 
 s2KO3  14010.0 
     46.520         nan 
   2143.083         nan 
      0.958         nan 
   1146.000         nan 
      7.033         nan 
      0.976         nan 
 
 s2KO3  15010.0 
     47.906         nan 
   2260.723         nan 
      0.959         nan 
   1195.100         nan 
      7.046         nan 
      0.976         nan 
 
 s2KO3  16010.0 
     49.119         nan 
   2315.744         nan 
      0.960         nan 
   1245.800         nan 
      7.058         nan 
      0.976         nan 
 
 s2KO3  17010.0 
     50.227         nan 
   2408.138         nan 
      0.961         nan 
   1280.100         nan 
      7.045         nan 
      0.976         nan 
 
 s2KO3  18010.0 
     51.212         nan 
   2468.912         nan 
      0.962         nan 
   1318.800         nan 
      7.061         nan 
      0.976         nan 
 
 s2KO3  19010.0 
     52.109         nan 
   2400.476         nan 
      0.965         nan 
   1346.800         nan 
      7.061         nan 
      0.976         nan 
 
 s2KO3  20010.0 
     53.495         nan 
   2463.998         nan 
      0.965         nan 
   1392.100         nan 
      7.071         nan 
      0.976         nan 
 
 s2KO3  21010.0 
     53.930         nan 
   2511.758         nan 
      0.966         nan 
   1420.100         nan 
      7.067         nan 
      0.976         nan 
 
 s2KO3  22010.0 
     54.947         nan 
   2604.524         nan 
      0.967         nan 
   1461.000         nan 
      7.077         nan 
      0.976         nan 
 
 s2KO3  23010.0 
     55.493         nan 
   2542.482         nan 
      0.969         nan 
   1478.700         nan 
      7.072         nan 
      0.976         nan 
 
 s2KO3  24010.0 
     56.485         nan 
   2643.231         nan 
      0.969         nan 
   1513.900         nan 
      7.078         nan 
      0.976         nan 
 
 s2KO3  25010.0 
     57.281         nan 
   2680.717         nan 
      0.970         nan 
   1546.800         nan 
      7.085         nan 
      0.976         nan 
 
 s2KO3  26010.0 
     58.199         nan 
   2740.213         nan 
      0.971         nan 
   1576.300         nan 
      7.086         nan 
      0.976         nan 
 
 s2KO3  27010.0 
     58.869         nan 
   2761.502         nan 
      0.971         nan 
   1607.600         nan 
      7.089         nan 
      0.976         nan 
 
 s2KO4  10.0 
      2.171         nan 
     49.200         nan 
      0.050         nan 
      9.700         nan 
      3.254         nan 
      0.892         nan 
 
 s2KO4  1010.0 
     18.497         nan 
    674.040         nan 
      0.843         nan 
    270.200         nan 
      6.862         nan 
      0.981         nan 
 
 s2KO4  2010.0 
     23.393         nan 
    960.174         nan 
      0.887         nan 
    395.200         nan 
      7.027         nan 
      0.981         nan 
 
 s2KO4  3010.0 
     27.626         nan 
   1266.966         nan 
      0.902         nan 
    504.500         nan 
      7.118         nan 
      0.981         nan 
 
 s2KO4  4010.0 
     30.793         nan 
   1438.221         nan 
      0.914         nan 
    590.200         nan 
      7.163         nan 
      0.981         nan 
 
 s2KO4  5010.0 
     34.211         nan 
   1673.613         nan 
      0.919         nan 
    682.600         nan 
      7.214         nan 
      0.981         nan 
 
 s2KO4  6010.0 
     35.843         nan 
   1760.107         nan 
      0.928         nan 
    743.700         nan 
      7.208         nan 
      0.981         nan 
 
 s2KO4  7010.0 
     38.192         nan 
   1840.390         nan 
      0.932         nan 
    820.400         nan 
      7.244         nan 
      0.981         nan 
 
 s2KO4  8010.0 
     40.519         nan 
   2001.171         nan 
      0.936         nan 
    883.500         nan 
      7.267         nan 
      0.981         nan 
 
 s2KO4  9010.0 
     42.146         nan 
   2168.784         nan 
      0.939         nan 
    945.300         nan 
      7.277         nan 
      0.981         nan 
 
 s2KO4  10010.0 
     43.977         nan 
   2204.857         nan 
      0.942         nan 
   1008.400         nan 
      7.295         nan 
      0.981         nan 
 
 s2KO4  11010.0 
     45.427         nan 
   2274.471         nan 
      0.945         nan 
   1059.700         nan 
      7.301         nan 
      0.981         nan 
 
 s2KO4  12010.0 
     47.264         nan 
   2406.489         nan 
      0.947         nan 
   1122.100         nan 
      7.312         nan 
      0.981         nan 
 
 s2KO4  13010.0 
     48.378         nan 
   2411.500         nan 
      0.949         nan 
   1170.400         nan 
      7.324         nan 
      0.981         nan 
 
 s2KO4  14010.0 
     50.114         nan 
   2422.954         nan 
      0.952         nan 
   1211.700         nan 
      7.324         nan 
      0.981         nan 
 
 s2KO4  15010.0 
     51.753         nan 
   2559.660         nan 
      0.952         nan 
   1273.900         nan 
      7.331         nan 
      0.981         nan 
 
 s2KO4  16010.0 
     52.561         nan 
   2515.972         nan 
      0.955         nan 
   1304.900         nan 
      7.336         nan 
      0.981         nan 
 
 s2KO4  17010.0 
     54.132         nan 
   2556.033         nan 
      0.957         nan 
   1359.100         nan 
      7.351         nan 
      0.981         nan 
 
 s2KO4  18010.0 
     54.825         nan 
   2625.864         nan 
      0.958         nan 
   1398.000         nan 
      7.346         nan 
      0.981         nan 
 
 s2KO4  19010.0 
     56.265         nan 
   2711.155         nan 
      0.959         nan 
   1449.600         nan 
      7.362         nan 
      0.981         nan 
 
 s2KO4  20010.0 
     57.771         nan 
   2733.473         nan 
      0.960         nan 
   1494.600         nan 
      7.367         nan 
      0.981         nan 
 
 s2KO4  21010.0 
     58.512         nan 
   2808.748         nan 
      0.961         nan 
   1527.900         nan 
      7.366         nan 
      0.981         nan 
 
 s2KO4  22010.0 
     59.313         nan 
   2779.116         nan 
      0.963         nan 
   1554.800         nan 
      7.365         nan 
      0.981         nan 
 
 s2KO4  23010.0 
     60.316         nan 
   2829.184         nan 
      0.964         nan 
   1595.200         nan 
      7.371         nan 
      0.981         nan 
 
 s2KO4  24010.0 
     61.315         nan 
   2847.074         nan 
      0.965         nan 
   1626.700         nan 
      7.370         nan 
      0.981         nan 
 
 s2KO4  25010.0 
     61.702         nan 
   2852.562         nan 
      0.966         nan 
   1657.300         nan 
      7.374         nan 
      0.981         nan 
 
 s2KO4  26010.0 
     62.873         nan 
   2910.367         nan 
      0.967         nan 
   1698.700         nan 
      7.383         nan 
      0.981         nan 
 
 s2KO4  27010.0 
     63.626         nan 
   2902.518         nan 
      0.968         nan 
   1726.300         nan 
      7.380         nan 
      0.981         nan 
 
 s2WE1  10.0 
      2.074         nan 
     32.600         nan 
      0.140         nan 
      9.300         nan 
      3.182         nan 
      0.886         nan 
 
 s2WE1  1010.0 
     17.496         nan 
    687.657         nan 
      0.822         nan 
    291.600         nan 
      6.823         nan 
      0.978         nan 
 
 s2WE1  2010.0 
     22.743         nan 
    992.613         nan 
      0.872         nan 
    434.400         nan 
      7.026         nan 
      0.979         nan 
 
 s2WE1  3010.0 
     26.636         nan 
   1159.483         nan 
      0.896         nan 
    553.100         nan 
      7.124         nan 
      0.979         nan 
 
 s2WE1  4010.0 
     30.209         nan 
   1407.764         nan 
      0.906         nan 
    661.800         nan 
      7.205         nan 
      0.980         nan 
 
 s2WE1  5010.0 
     33.050         nan 
   1482.706         nan 
      0.918         nan 
    743.600         nan 
      7.232         nan 
      0.980         nan 
 
 s2WE1  6010.0 
     34.624         nan 
   1574.395         nan 
      0.928         nan 
    802.300         nan 
      7.233         nan 
      0.979         nan 
 
 s2WE1  7010.0 
     37.172         nan 
   1721.627         nan 
      0.933         nan 
    883.800         nan 
      7.268         nan 
      0.979         nan 
 
 s2WE1  8010.0 
     38.870         nan 
   1846.309         nan 
      0.937         nan 
    949.100         nan 
      7.281         nan 
      0.979         nan 
 
 s2WE1  9010.0 
     40.693         nan 
   1894.299         nan 
      0.942         nan 
   1007.900         nan 
      7.310         nan 
      0.980         nan 
 
 s2WE1  10010.0 
     42.114         nan 
   1966.617         nan 
      0.946         nan 
   1058.100         nan 
      7.319         nan 
      0.980         nan 
 
 s2WE1  11010.0 
     43.687         nan 
   2042.217         nan 
      0.949         nan 
   1111.900         nan 
      7.334         nan 
      0.980         nan 
 
 s2WE1  12010.0 
     45.196         nan 
   2105.051         nan 
      0.952         nan 
   1165.200         nan 
      7.341         nan 
      0.979         nan 
 
 s2WE1  13010.0 
     46.421         nan 
   2166.340         nan 
      0.954         nan 
   1209.300         nan 
      7.345         nan 
      0.979         nan 
 
 s2WE1  14010.0 
     47.994         nan 
   2220.578         nan 
      0.956         nan 
   1256.300         nan 
      7.355         nan 
      0.980         nan 
 
 s2WE1  15010.0 
     48.487         nan 
   2266.834         nan 
      0.958         nan 
   1291.300         nan 
      7.359         nan 
      0.980         nan 
 
 s2WE1  16010.0 
     49.609         nan 
   2338.578         nan 
      0.959         nan 
   1342.000         nan 
      7.368         nan 
      0.980         nan 
 
 s2WE1  17010.0 
     50.666         nan 
   2360.543         nan 
      0.961         nan 
   1373.900         nan 
      7.367         nan 
      0.980         nan 
 
 s2WE1  18010.0 
     52.130         nan 
   2453.018         nan 
      0.962         nan 
   1418.700         nan 
      7.375         nan 
      0.979         nan 
 
 s2WE1  19010.0 
     53.338         nan 
   2538.614         nan 
      0.963         nan 
   1465.500         nan 
      7.384         nan 
      0.980         nan 
 
 s2WE1  20010.0 
     54.234         nan 
   2595.318         nan 
      0.964         nan 
   1500.500         nan 
      7.384         nan 
      0.979         nan 
 
 s2WE1  21010.0 
     54.593         nan 
   2601.855         nan 
      0.966         nan 
   1525.600         nan 
      7.385         nan 
      0.979         nan 
 
 s2WE1  22010.0 
     55.526         nan 
   2630.794         nan 
      0.967         nan 
   1550.300         nan 
      7.385         nan 
      0.979         nan 
 
 s2WE1  23010.0 
     56.734         nan 
   2648.567         nan 
      0.968         nan 
   1595.600         nan 
      7.393         nan 
      0.979         nan 
 
 s2WE1  24010.0 
     57.451         nan 
   2721.663         nan 
      0.969         nan 
   1630.100         nan 
      7.398         nan 
      0.980         nan 
 
 s2WE1  25010.0 
     58.140         nan 
   2747.964         nan 
      0.969         nan 
   1657.700         nan 
      7.404         nan 
      0.980         nan 
 
 s2WE1  26010.0 
     59.188         nan 
   2750.362         nan 
      0.971         nan 
   1687.300         nan 
      7.408         nan 
      0.980         nan 
 
 s2WE1  27010.0 
     59.651         nan 
   2788.280         nan 
      0.971         nan 
   1712.400         nan 
      7.405         nan 
      0.980         nan 
 
 s2WE2  10.0 
      1.841         nan 
     28.500         nan 
      0.230         nan 
      8.800         nan 
      3.074         nan 
      0.874         nan 
 
 s2WE2  1010.0 
     17.631         nan 
    577.642         nan 
      0.840         nan 
    272.100         nan 
      6.499         nan 
      0.968         nan 
 
 s2WE2  2010.0 
     22.776         nan 
    815.810         nan 
      0.890         nan 
    398.000         nan 
      6.666         nan 
      0.968         nan 
 
 s2WE2  3010.0 
     26.418         nan 
   1012.718         nan 
      0.910         nan 
    496.900         nan 
      6.731         nan 
      0.968         nan 
 
 s2WE2  4010.0 
     29.611         nan 
   1199.546         nan 
      0.921         nan 
    586.300         nan 
      6.822         nan 
      0.969         nan 
 
 s2WE2  5010.0 
     32.301         nan 
   1378.763         nan 
      0.928         nan 
    665.000         nan 
      6.847         nan 
      0.969         nan 
 
 s2WE2  6010.0 
     34.441         nan 
   1470.940         nan 
      0.935         nan 
    731.100         nan 
      6.876         nan 
      0.969         nan 
 
 s2WE2  7010.0 
     36.648         nan 
   1624.180         nan 
      0.939         nan 
    801.000         nan 
      6.921         nan 
      0.969         nan 
 
 s2WE2  8010.0 
     37.459         nan 
   1654.652         nan 
      0.945         nan 
    847.300         nan 
      6.920         nan 
      0.969         nan 
 
 s2WE2  9010.0 
     39.943         nan 
   1809.788         nan 
      0.947         nan 
    914.100         nan 
      6.932         nan 
      0.969         nan 
 
 s2WE2  10010.0 
     41.725         nan 
   1932.682         nan 
      0.949         nan 
    968.600         nan 
      6.959         nan 
      0.969         nan 
 
 s2WE2  11010.0 
     42.656         nan 
   1972.550         nan 
      0.953         nan 
   1005.600         nan 
      6.958         nan 
      0.969         nan 
 
 s2WE2  12010.0 
     44.047         nan 
   1991.515         nan 
      0.955         nan 
   1054.000         nan 
      6.974         nan 
      0.969         nan 
 
 s2WE2  13010.0 
     45.315         nan 
   2064.724         nan 
      0.957         nan 
   1097.600         nan 
      6.969         nan 
      0.969         nan 
 
 s2WE2  14010.0 
     46.598         nan 
   2206.495         nan 
      0.958         nan 
   1143.600         nan 
      6.970         nan 
      0.969         nan 
 
 s2WE2  15010.0 
     47.892         nan 
   2243.303         nan 
      0.960         nan 
   1189.400         nan 
      6.998         nan 
      0.969         nan 
 
 s2WE2  16010.0 
     48.717         nan 
   2260.159         nan 
      0.962         nan 
   1217.700         nan 
      6.979         nan 
      0.969         nan 
 
 s2WE2  17010.0 
     49.783         nan 
   2256.957         nan 
      0.964         nan 
   1247.600         nan 
      6.995         nan 
      0.969         nan 
 
 s2WE2  18010.0 
     50.900         nan 
   2400.333         nan 
      0.965         nan 
   1285.900         nan 
      7.000         nan 
      0.969         nan 
 
 s2WE2  19010.0 
     51.857         nan 
   2386.798         nan 
      0.966         nan 
   1325.800         nan 
      7.005         nan 
      0.969         nan 
 
 s2WE2  20010.0 
     52.744         nan 
   2406.239         nan 
      0.967         nan 
   1350.400         nan 
      6.998         nan 
      0.969         nan 
 
 s2WE2  21010.0 
     53.965         nan 
   2461.002         nan 
      0.968         nan 
   1400.000         nan 
      7.012         nan 
      0.969         nan 
 
 s2WE2  22010.0 
     54.776         nan 
   2485.968         nan 
      0.969         nan 
   1423.800         nan 
      7.013         nan 
      0.969         nan 
 
 s2WE2  23010.0 
     55.488         nan 
   2538.688         nan 
      0.969         nan 
   1454.200         nan 
      7.015         nan 
      0.969         nan 
 
 s2WE2  24010.0 
     56.719         nan 
   2614.321         nan 
      0.970         nan 
   1492.400         nan 
      7.032         nan 
      0.969         nan 
 
 s2WE2  25010.0 
     57.260         nan 
   2588.711         nan 
      0.971         nan 
   1513.600         nan 
      7.024         nan 
      0.969         nan 
 
 s2WE2  26010.0 
     57.978         nan 
   2625.488         nan 
      0.972         nan 
   1544.900         nan 
      7.028         nan 
      0.969         nan 
 
 s2WE2  27010.0 
     59.063         nan 
   2712.307         nan 
      0.972         nan 
   1573.300         nan 
      7.029         nan 
      0.969         nan 
 
 s2WE3  10.0 
      2.392         nan 
     40.200         nan 
      0.120         nan 
      9.400         nan 
      3.202         nan 
      0.888         nan 
 
 s2WE3  1010.0 
     16.527         nan 
    574.822         nan 
      0.852         nan 
    245.400         nan 
      6.425         nan 
      0.973         nan 
 
 s2WE3  2010.0 
     22.416         nan 
    873.940         nan 
      0.889         nan 
    380.500         nan 
      6.646         nan 
      0.974         nan 
 
 s2WE3  3010.0 
     27.640         nan 
   1152.466         nan 
      0.902         nan 
    500.300         nan 
      6.790         nan 
      0.975         nan 
 
 s2WE3  4010.0 
     30.071         nan 
   1308.890         nan 
      0.916         nan 
    581.700         nan 
      6.811         nan 
      0.975         nan 
 
 s2WE3  5010.0 
     32.833         nan 
   1404.037         nan 
      0.926         nan 
    653.900         nan 
      6.828         nan 
      0.975         nan 
 
 s2WE3  6010.0 
     35.542         nan 
   1590.375         nan 
      0.931         nan 
    731.500         nan 
      6.874         nan 
      0.975         nan 
 
 s2WE3  7010.0 
     37.177         nan 
   1603.923         nan 
      0.938         nan 
    788.100         nan 
      6.873         nan 
      0.975         nan 
 
 s2WE3  8010.0 
     39.385         nan 
   1796.967         nan 
      0.941         nan 
    848.300         nan 
      6.892         nan 
      0.975         nan 
 
 s2WE3  9010.0 
     40.835         nan 
   1919.148         nan 
      0.945         nan 
    905.100         nan 
      6.927         nan 
      0.975         nan 
 
 s2WE3  10010.0 
     43.079         nan 
   1947.779         nan 
      0.947         nan 
    971.200         nan 
      6.928         nan 
      0.975         nan 
 
 s2WE3  11010.0 
     44.668         nan 
   2060.942         nan 
      0.950         nan 
   1021.900         nan 
      6.938         nan 
      0.975         nan 
 
 s2WE3  12010.0 
     46.191         nan 
   2084.025         nan 
      0.952         nan 
   1069.700         nan 
      6.943         nan 
      0.975         nan 
 
 s2WE3  13010.0 
     47.083         nan 
   2182.747         nan 
      0.955         nan 
   1104.200         nan 
      6.950         nan 
      0.975         nan 
 
 s2WE3  14010.0 
     48.934         nan 
   2160.904         nan 
      0.957         nan 
   1163.000         nan 
      6.970         nan 
      0.975         nan 
 
 s2WE3  15010.0 
     49.956         nan 
   2212.063         nan 
      0.959         nan 
   1195.900         nan 
      6.974         nan 
      0.975         nan 
 
 s2WE3  16010.0 
     51.039         nan 
   2329.111         nan 
      0.960         nan 
   1239.300         nan 
      6.966         nan 
      0.975         nan 
 
 s2WE3  17010.0 
     52.332         nan 
   2371.369         nan 
      0.961         nan 
   1278.800         nan 
      6.984         nan 
      0.975         nan 
 
 s2WE3  18010.0 
     52.945         nan 
   2351.270         nan 
      0.963         nan 
   1303.600         nan 
      6.976         nan 
      0.975         nan 
 
 s2WE3  19010.0 
     54.350         nan 
   2419.304         nan 
      0.964         nan 
   1352.300         nan 
      6.985         nan 
      0.975         nan 
 
 s2WE3  20010.0 
     55.542         nan 
   2503.748         nan 
      0.965         nan 
   1394.400         nan 
      6.990         nan 
      0.975         nan 
 
 s2WE3  21010.0 
     56.430         nan 
   2548.819         nan 
      0.966         nan 
   1431.400         nan 
      7.002         nan 
      0.975         nan 
 
 s2WE3  22010.0 
     57.633         nan 
   2625.499         nan 
      0.967         nan 
   1463.400         nan 
      6.999         nan 
      0.975         nan 
 
 s2WE3  23010.0 
     58.182         nan 
   2614.362         nan 
      0.968         nan 
   1490.100         nan 
      6.994         nan 
      0.975         nan 
 
 s2WE3  24010.0 
     59.077         nan 
   2687.535         nan 
      0.968         nan 
   1528.300         nan 
      7.007         nan 
      0.975         nan 
 
 s2WE3  25010.0 
     60.018         nan 
   2699.275         nan 
      0.970         nan 
   1554.800         nan 
      7.007         nan 
      0.975         nan 
 
 s2WE3  26010.0 
     60.728         nan 
   2719.396         nan 
      0.971         nan 
   1579.200         nan 
      7.015         nan 
      0.975         nan 
 
 s2WE3  27010.0 
     61.612         nan 
   2784.841         nan 
      0.971         nan 
   1609.600         nan 
      7.015         nan 
      0.975         nan 
 
 s2WE4  10.0 
      2.262         nan 
     34.900         nan 
      0.190         nan 
      8.900         nan 
      3.079         nan 
      0.872         nan 
 
 s2WE4  1010.0 
     16.948         nan 
    618.264         nan 
      0.844         nan 
    252.400         nan 
      6.295         nan 
      0.961         nan 
 
 s2WE4  2010.0 
     22.077         nan 
    948.029         nan 
      0.883         nan 
    380.300         nan 
      6.432         nan 
      0.962         nan 
 
 s2WE4  3010.0 
     26.291         nan 
   1114.467         nan 
      0.903         nan 
    488.400         nan 
      6.517         nan 
      0.961         nan 
 
 s2WE4  4010.0 
     29.169         nan 
   1188.219         nan 
      0.918         nan 
    571.200         nan 
      6.562         nan 
      0.962         nan 
 
 s2WE4  5010.0 
     32.145         nan 
   1470.117         nan 
      0.923         nan 
    663.400         nan 
      6.620         nan 
      0.962         nan 
 
 s2WE4  6010.0 
     34.310         nan 
   1574.602         nan 
      0.930         nan 
    735.100         nan 
      6.660         nan 
      0.962         nan 
 
 s2WE4  7010.0 
     36.166         nan 
   1638.720         nan 
      0.937         nan 
    782.900         nan 
      6.643         nan 
      0.962         nan 
 
 s2WE4  8010.0 
     38.358         nan 
   1812.273         nan 
      0.940         nan 
    852.900         nan 
      6.672         nan 
      0.962         nan 
 
 s2WE4  9010.0 
     40.504         nan 
   1911.956         nan 
      0.943         nan 
    915.500         nan 
      6.695         nan 
      0.962         nan 
 
 s2WE4  10010.0 
     41.797         nan 
   1997.517         nan 
      0.946         nan 
    968.800         nan 
      6.693         nan 
      0.962         nan 
 
 s2WE4  11010.0 
     43.140         nan 
   2043.326         nan 
      0.949         nan 
   1018.100         nan 
      6.710         nan 
      0.962         nan 
 
 s2WE4  12010.0 
     44.991         nan 
   2148.271         nan 
      0.951         nan 
   1073.600         nan 
      6.728         nan 
      0.962         nan 
 
 s2WE4  13010.0 
     45.867         nan 
   2184.983         nan 
      0.954         nan 
   1112.700         nan 
      6.725         nan 
      0.962         nan 
 
 s2WE4  14010.0 
     47.031         nan 
   2214.166         nan 
      0.956         nan 
   1158.800         nan 
      6.728         nan 
      0.962         nan 
 
 s2WE4  15010.0 
     48.671         nan 
   2272.859         nan 
      0.958         nan 
   1201.000         nan 
      6.736         nan 
      0.962         nan 
 
 s2WE4  16010.0 
     50.421         nan 
   2330.802         nan 
      0.959         nan 
   1257.700         nan 
      6.775         nan 
      0.963         nan 
 
 s2WE4  17010.0 
     51.177         nan 
   2427.610         nan 
      0.960         nan 
   1290.800         nan 
      6.748         nan 
      0.962         nan 
 
 s2WE4  18010.0 
     52.238         nan 
   2528.028         nan 
      0.961         nan 
   1333.800         nan 
      6.763         nan 
      0.962         nan 
 
 s2WE4  19010.0 
     53.358         nan 
   2477.490         nan 
      0.963         nan 
   1362.100         nan 
      6.768         nan 
      0.962         nan 
 
 s2WE4  20010.0 
     54.155         nan 
   2533.308         nan 
      0.964         nan 
   1396.200         nan 
      6.765         nan 
      0.962         nan 
 
 s2WE4  21010.0 
     55.215         nan 
   2542.455         nan 
      0.965         nan 
   1437.700         nan 
      6.766         nan 
      0.962         nan 
 
 s2WE4  22010.0 
     56.039         nan 
   2623.312         nan 
      0.966         nan 
   1466.000         nan 
      6.777         nan 
      0.962         nan 
 
 s2WE4  23010.0 
     56.614         nan 
   2690.052         nan 
      0.967         nan 
   1494.300         nan 
      6.772         nan 
      0.962         nan 
 
 s2WE4  24010.0 
     57.462         nan 
   2701.025         nan 
      0.968         nan 
   1526.100         nan 
      6.776         nan 
      0.962         nan 
 
 s2WE4  25010.0 
     58.746         nan 
   2752.230         nan 
      0.969         nan 
   1562.800         nan 
      6.778         nan 
      0.962         nan 
 
 s2WE4  26010.0 
     59.639         nan 
   2812.976         nan 
      0.970         nan 
   1598.800         nan 
      6.788         nan 
      0.962         nan 
 
 s2WE4  27010.0 
     60.180         nan 
   2784.057         nan 
      0.971         nan 
   1618.300         nan 
      6.788         nan 
      0.962         nan 
 
 s2WE5  10.0 
      2.052         nan 
     22.200         nan 
      0.260         nan 
      8.700         nan 
      3.062         nan 
      0.874         nan 
 
 s2WE5  1010.0 
     17.369         nan 
    646.701         nan 
      0.835         nan 
    268.400         nan 
      6.543         nan 
      0.973         nan 
 
 s2WE5  2010.0 
     23.051         nan 
    941.872         nan 
      0.879         nan 
    405.600         nan 
      6.726         nan 
      0.974         nan 
 
 s2WE5  3010.0 
     27.443         nan 
   1127.849         nan 
      0.898         nan 
    525.700         nan 
      6.848         nan 
      0.974         nan 
 
 s2WE5  4010.0 
     30.472         nan 
   1252.755         nan 
      0.914         nan 
    609.300         nan 
      6.883         nan 
      0.974         nan 
 
 s2WE5  5010.0 
     32.670         nan 
   1479.638         nan 
      0.922         nan 
    687.400         nan 
      6.896         nan 
      0.974         nan 
 
 s2WE5  6010.0 
     35.985         nan 
   1612.028         nan 
      0.928         nan 
    771.800         nan 
      6.960         nan 
      0.975         nan 
 
 s2WE5  7010.0 
     37.708         nan 
   1662.818         nan 
      0.935         nan 
    833.400         nan 
      6.973         nan 
      0.974         nan 
 
 s2WE5  8010.0 
     40.150         nan 
   1821.527         nan 
      0.938         nan 
    901.300         nan 
      6.976         nan 
      0.974         nan 
 
 s2WE5  9010.0 
     41.954         nan 
   1920.791         nan 
      0.942         nan 
    964.700         nan 
      7.003         nan 
      0.974         nan 
 
 s2WE5  10010.0 
     43.457         nan 
   1977.490         nan 
      0.946         nan 
   1022.100         nan 
      7.023         nan 
      0.974         nan 
 
 s2WE5  11010.0 
     45.019         nan 
   2034.423         nan 
      0.949         nan 
   1064.900         nan 
      7.024         nan 
      0.974         nan 
 
 s2WE5  12010.0 
     46.581         nan 
   2128.952         nan 
      0.951         nan 
   1124.800         nan 
      7.035         nan 
      0.974         nan 
 
 s2WE5  13010.0 
     47.882         nan 
   2122.045         nan 
      0.954         nan 
   1161.600         nan 
      7.038         nan 
      0.974         nan 
 
 s2WE5  14010.0 
     49.013         nan 
   2224.875         nan 
      0.956         nan 
   1209.700         nan 
      7.044         nan 
      0.974         nan 
 
 s2WE5  15010.0 
     50.612         nan 
   2277.481         nan 
      0.957         nan 
   1262.500         nan 
      7.071         nan 
      0.975         nan 
 
 s2WE5  16010.0 
     51.771         nan 
   2302.801         nan 
      0.959         nan 
   1292.000         nan 
      7.060         nan 
      0.974         nan 
 
 s2WE5  17010.0 
     53.111         nan 
   2388.009         nan 
      0.961         nan 
   1336.700         nan 
      7.072         nan 
      0.975         nan 
 
 s2WE5  18010.0 
     53.905         nan 
   2417.531         nan 
      0.962         nan 
   1373.200         nan 
      7.071         nan 
      0.974         nan 
 
 s2WE5  19010.0 
     54.723         nan 
   2455.741         nan 
      0.964         nan 
   1409.300         nan 
      7.078         nan 
      0.974         nan 
 
 s2WE5  20010.0 
     56.270         nan 
   2499.834         nan 
      0.965         nan 
   1441.700         nan 
      7.081         nan 
      0.974         nan 
 
 s2WE5  21010.0 
     57.146         nan 
   2537.964         nan 
      0.966         nan 
   1483.400         nan 
      7.091         nan 
      0.974         nan 
 
 s2WE5  22010.0 
     57.951         nan 
   2534.933         nan 
      0.967         nan 
   1517.400         nan 
      7.093         nan 
      0.974         nan 
 
 s2WE5  23010.0 
     58.658         nan 
   2623.927         nan 
      0.968         nan 
   1544.000         nan 
      7.082         nan 
      0.974         nan 
 
 s2WE5  24010.0 
     59.699         nan 
   2629.292         nan 
      0.969         nan 
   1575.600         nan 
      7.093         nan 
      0.974         nan 
 
 s2WE5  25010.0 
     60.515         nan 
   2687.380         nan 
      0.970         nan 
   1607.600         nan 
      7.099         nan 
      0.974         nan 
 
 s2WE5  26010.0 
     61.335         nan 
   2728.179         nan 
      0.970         nan 
   1637.000         nan 
      7.094         nan 
      0.974         nan 
 
 s2WE5  27010.0 
     61.998         nan 
   2732.338         nan 
      0.972         nan 
   1666.500         nan 
      7.109         nan 
      0.974         nan 
 
 s2WT1  10.0 
      1.730         nan 
     25.200         nan 
      0.200         nan 
      9.000         nan 
      3.122         nan 
      0.880         nan 
 
 s2WT1  1010.0 
     16.357         nan 
    678.271         nan 
      0.837         nan 
    265.900         nan 
      6.523         nan 
      0.970         nan 
 
 s2WT1  2010.0 
     21.775         nan 
   1010.521         nan 
      0.876         nan 
    407.300         nan 
      6.698         nan 
      0.969         nan 
 
 s2WT1  3010.0 
     25.194         nan 
   1162.993         nan 
      0.896         nan 
    520.600         nan 
      6.768         nan 
      0.969         nan 
 
 s2WT1  4010.0 
     28.285         nan 
   1395.635         nan 
      0.909         nan 
    619.500         nan 
      6.839         nan 
      0.969         nan 
 
 s2WT1  5010.0 
     30.816         nan 
   1498.825         nan 
      0.918         nan 
    707.800         nan 
      6.878         nan 
      0.969         nan 
 
 s2WT1  6010.0 
     33.445         nan 
   1614.671         nan 
      0.925         nan 
    786.400         nan 
      6.914         nan 
      0.969         nan 
 
 s2WT1  7010.0 
     35.647         nan 
   1695.120         nan 
      0.932         nan 
    854.100         nan 
      6.950         nan 
      0.970         nan 
 
 s2WT1  8010.0 
     37.478         nan 
   1772.881         nan 
      0.938         nan 
    913.800         nan 
      6.939         nan 
      0.969         nan 
 
 s2WT1  9010.0 
     38.892         nan 
   1828.585         nan 
      0.942         nan 
    969.800         nan 
      6.955         nan 
      0.970         nan 
 
 s2WT1  10010.0 
     40.396         nan 
   1944.697         nan 
      0.945         nan 
   1025.100         nan 
      6.957         nan 
      0.969         nan 
 
 s2WT1  11010.0 
     41.871         nan 
   1966.395         nan 
      0.949         nan 
   1087.100         nan 
      6.989         nan 
      0.970         nan 
 
 s2WT1  12010.0 
     43.746         nan 
   2114.539         nan 
      0.950         nan 
   1142.600         nan 
      7.000         nan 
      0.970         nan 
 
 s2WT1  13010.0 
     44.592         nan 
   2137.785         nan 
      0.954         nan 
   1176.300         nan 
      6.995         nan 
      0.969         nan 
 
 s2WT1  14010.0 
     46.028         nan 
   2158.000         nan 
      0.956         nan 
   1227.600         nan 
      7.007         nan 
      0.969         nan 
 
 s2WT1  15010.0 
     46.800         nan 
   2312.351         nan 
      0.957         nan 
   1270.700         nan 
      7.007         nan 
      0.969         nan 
 
 s2WT1  16010.0 
     48.373         nan 
   2341.776         nan 
      0.959         nan 
   1320.000         nan 
      7.025         nan 
      0.970         nan 
 
 s2WT1  17010.0 
     50.032         nan 
   2423.533         nan 
      0.960         nan 
   1361.800         nan 
      7.033         nan 
      0.970         nan 
 
 s2WT1  18010.0 
     49.935         nan 
   2415.553         nan 
      0.962         nan 
   1389.300         nan 
      7.027         nan 
      0.969         nan 
 
 s2WT1  19010.0 
     51.219         nan 
   2451.363         nan 
      0.964         nan 
   1431.500         nan 
      7.040         nan 
      0.970         nan 
 
 s2WT1  20010.0 
     52.367         nan 
   2531.061         nan 
      0.965         nan 
   1469.400         nan 
      7.044         nan 
      0.970         nan 
 
 s2WT1  21010.0 
     53.160         nan 
   2477.752         nan 
      0.966         nan 
   1502.200         nan 
      7.040         nan 
      0.969         nan 
 
 s2WT1  22010.0 
     54.186         nan 
   2614.851         nan 
      0.967         nan 
   1538.100         nan 
      7.048         nan 
      0.970         nan 
 
 s2WT1  23010.0 
     55.002         nan 
   2634.472         nan 
      0.968         nan 
   1566.400         nan 
      7.048         nan 
      0.969         nan 
 
 s2WT1  24010.0 
     55.664         nan 
   2600.744         nan 
      0.969         nan 
   1595.400         nan 
      7.047         nan 
      0.969         nan 
 
 s2WT1  25010.0 
     56.751         nan 
   2700.982         nan 
      0.970         nan 
   1630.600         nan 
      7.057         nan 
      0.970         nan 
 
 s2WT1  26010.0 
     57.479         nan 
   2747.907         nan 
      0.970         nan 
   1662.600         nan 
      7.055         nan 
      0.969         nan 
 
 s2WT1  27010.0 
     58.423         nan 
   2761.121         nan 
      0.971         nan 
   1687.400         nan 
      7.060         nan 
      0.969         nan 
 
 s2WT2  10.0 
      1.875         nan 
     24.200         nan 
      0.280         nan 
      8.400         nan 
      2.972         nan 
      0.860         nan 
 
 s2WT2  1010.0 
     18.042         nan 
    690.194         nan 
      0.822         nan 
    286.400         nan 
      6.598         nan 
      0.970         nan 
 
 s2WT2  2010.0 
     23.534         nan 
    995.709         nan 
      0.872         nan 
    432.500         nan 
      6.799         nan 
      0.970         nan 
 
 s2WT2  3010.0 
     27.569         nan 
   1204.361         nan 
      0.895         nan 
    545.600         nan 
      6.861         nan 
      0.969         nan 
 
 s2WT2  4010.0 
     31.030         nan 
   1343.951         nan 
      0.910         nan 
    642.100         nan 
      6.944         nan 
      0.970         nan 
 
 s2WT2  5010.0 
     33.478         nan 
   1418.017         nan 
      0.922         nan 
    716.300         nan 
      6.947         nan 
      0.970         nan 
 
 s2WT2  6010.0 
     36.068         nan 
   1594.165         nan 
      0.928         nan 
    794.100         nan 
      6.984         nan 
      0.970         nan 
 
 s2WT2  7010.0 
     38.001         nan 
   1693.851         nan 
      0.934         nan 
    864.800         nan 
      7.022         nan 
      0.970         nan 
 
 s2WT2  8010.0 
     39.991         nan 
   1730.995         nan 
      0.939         nan 
    929.400         nan 
      7.047         nan 
      0.970         nan 
 
 s2WT2  9010.0 
     41.905         nan 
   1880.613         nan 
      0.943         nan 
    994.900         nan 
      7.058         nan 
      0.970         nan 
 
 s2WT2  10010.0 
     43.398         nan 
   1868.893         nan 
      0.947         nan 
   1043.100         nan 
      7.071         nan 
      0.970         nan 
 
 s2WT2  11010.0 
     45.325         nan 
   2004.459         nan 
      0.950         nan 
   1096.700         nan 
      7.078         nan 
      0.970         nan 
 
 s2WT2  12010.0 
     46.395         nan 
   2069.611         nan 
      0.952         nan 
   1144.000         nan 
      7.080         nan 
      0.970         nan 
 
 s2WT2  13010.0 
     47.599         nan 
   2077.396         nan 
      0.955         nan 
   1184.900         nan 
      7.099         nan 
      0.970         nan 
 
 s2WT2  14010.0 
     49.165         nan 
   2133.980         nan 
      0.957         nan 
   1231.100         nan 
      7.095         nan 
      0.970         nan 
 
 s2WT2  15010.0 
     50.362         nan 
   2191.430         nan 
      0.959         nan 
   1281.000         nan 
      7.126         nan 
      0.970         nan 
 
 s2WT2  16010.0 
     51.488         nan 
   2191.781         nan 
      0.961         nan 
   1318.300         nan 
      7.127         nan 
      0.970         nan 
 
 s2WT2  17010.0 
     52.461         nan 
   2342.446         nan 
      0.962         nan 
   1361.000         nan 
      7.129         nan 
      0.970         nan 
 
 s2WT2  18010.0 
     53.156         nan 
   2336.358         nan 
      0.964         nan 
   1387.000         nan 
      7.129         nan 
      0.970         nan 
 
 s2WT2  19010.0 
     54.467         nan 
   2381.827         nan 
      0.965         nan 
   1428.000         nan 
      7.134         nan 
      0.970         nan 
 
 s2WT2  20010.0 
     55.162         nan 
   2416.162         nan 
      0.966         nan 
   1457.800         nan 
      7.128         nan 
      0.970         nan 
 
 s2WT2  21010.0 
     56.435         nan 
   2462.508         nan 
      0.967         nan 
   1498.600         nan 
      7.147         nan 
      0.970         nan 
 
 s2WT2  22010.0 
     57.100         nan 
   2482.536         nan 
      0.969         nan 
   1516.700         nan 
      7.144         nan 
      0.970         nan 
 
 s2WT2  23010.0 
     57.976         nan 
   2533.226         nan 
      0.969         nan 
   1558.700         nan 
      7.150         nan 
      0.970         nan 
 
 s2WT2  24010.0 
     58.714         nan 
   2542.449         nan 
      0.970         nan 
   1587.900         nan 
      7.152         nan 
      0.970         nan 
 
 s2WT2  25010.0 
     59.432         nan 
   2556.006         nan 
      0.972         nan 
   1608.200         nan 
      7.150         nan 
      0.970         nan 
 
 s2WT2  26010.0 
     60.172         nan 
   2606.652         nan 
      0.972         nan 
   1638.100         nan 
      7.156         nan 
      0.970         nan 
 
 s2WT2  27010.0 
     61.222         nan 
   2611.196         nan 
      0.973         nan 
   1674.100         nan 
      7.163         nan 
      0.970         nan 
 
 s2WT3  10.0 
      1.953         nan 
     31.200         nan 
      0.190         nan 
      9.000         nan 
      3.114         nan 
      0.878         nan 
 
 s2WT3  1010.0 
     15.713         nan 
    628.206         nan 
      0.840         nan 
    262.600         nan 
      6.418         nan 
      0.967         nan 
 
 s2WT3  2010.0 
     21.037         nan 
    852.606         nan 
      0.886         nan 
    389.800         nan 
      6.584         nan 
      0.966         nan 
 
 s2WT3  3010.0 
     24.703         nan 
   1112.096         nan 
      0.903         nan 
    500.500         nan 
      6.661         nan 
      0.966         nan 
 
 s2WT3  4010.0 
     27.741         nan 
   1246.737         nan 
      0.915         nan 
    595.500         nan 
      6.720         nan 
      0.966         nan 
 
 s2WT3  5010.0 
     29.867         nan 
   1366.148         nan 
      0.926         nan 
    664.400         nan 
      6.729         nan 
      0.965         nan 
 
 s2WT3  6010.0 
     32.275         nan 
   1484.184         nan 
      0.932         nan 
    736.300         nan 
      6.761         nan 
      0.966         nan 
 
 s2WT3  7010.0 
     34.438         nan 
   1584.407         nan 
      0.938         nan 
    800.800         nan 
      6.782         nan 
      0.966         nan 
 
 s2WT3  8010.0 
     36.331         nan 
   1648.043         nan 
      0.942         nan 
    866.000         nan 
      6.791         nan 
      0.966         nan 
 
 s2WT3  9010.0 
     37.555         nan 
   1801.875         nan 
      0.946         nan 
    916.300         nan 
      6.815         nan 
      0.966         nan 
 
 s2WT3  10010.0 
     39.296         nan 
   1862.684         nan 
      0.949         nan 
    973.800         nan 
      6.833         nan 
      0.966         nan 
 
 s2WT3  11010.0 
     41.335         nan 
   1961.405         nan 
      0.951         nan 
   1031.500         nan 
      6.844         nan 
      0.966         nan 
 
 s2WT3  12010.0 
     41.929         nan 
   1963.270         nan 
      0.954         nan 
   1069.000         nan 
      6.859         nan 
      0.966         nan 
 
 s2WT3  13010.0 
     43.374         nan 
   2086.243         nan 
      0.956         nan 
   1116.500         nan 
      6.851         nan 
      0.966         nan 
 
 s2WT3  14010.0 
     44.591         nan 
   2202.625         nan 
      0.957         nan 
   1169.200         nan 
      6.867         nan 
      0.966         nan 
 
 s2WT3  15010.0 
     45.965         nan 
   2243.269         nan 
      0.959         nan 
   1211.600         nan 
      6.863         nan 
      0.966         nan 
 
 s2WT3  16010.0 
     47.036         nan 
   2242.007         nan 
      0.961         nan 
   1250.700         nan 
      6.886         nan 
      0.966         nan 
 
 s2WT3  17010.0 
     47.862         nan 
   2371.628         nan 
      0.962         nan 
   1279.200         nan 
      6.887         nan 
      0.966         nan 
 
 s2WT3  18010.0 
     48.817         nan 
   2318.423         nan 
      0.964         nan 
   1316.100         nan 
      6.893         nan 
      0.966         nan 
 
 s2WT3  19010.0 
     49.340         nan 
   2359.940         nan 
      0.966         nan 
   1338.600         nan 
      6.891         nan 
      0.966         nan 
 
 s2WT3  20010.0 
     50.678         nan 
   2395.200         nan 
      0.967         nan 
   1382.300         nan 
      6.905         nan 
      0.966         nan 
 
 s2WT3  21010.0 
     51.491         nan 
   2554.924         nan 
      0.967         nan 
   1413.700         nan 
      6.899         nan 
      0.966         nan 
 
 s2WT3  22010.0 
     52.236         nan 
   2550.864         nan 
      0.968         nan 
   1449.400         nan 
      6.902         nan 
      0.966         nan 
 
 s2WT3  23010.0 
     53.235         nan 
   2525.768         nan 
      0.970         nan 
   1479.300         nan 
      6.912         nan 
      0.966         nan 
 
 s2WT3  24010.0 
     54.074         nan 
   2602.095         nan 
      0.970         nan 
   1515.200         nan 
      6.908         nan 
      0.966         nan 
 
 s2WT3  25010.0 
     55.104         nan 
   2659.494         nan 
      0.971         nan 
   1551.800         nan 
      6.913         nan 
      0.966         nan 
 
 s2WT3  26010.0 
     55.779         nan 
   2699.312         nan 
      0.971         nan 
   1576.000         nan 
      6.915         nan 
      0.966         nan 
 
 s2WT3  27010.0 
     56.547         nan 
   2738.357         nan 
      0.972         nan 
   1603.100         nan 
      6.915         nan 
      0.966         nan 
 
 s2WT4  10.0 
      2.089         nan 
     37.600         nan 
      0.150         nan 
      9.200         nan 
      3.154         nan 
      0.882         nan 
 
 s2WT4  1010.0 
     18.057         nan 
    711.385         nan 
      0.814         nan 
    296.800         nan 
      6.737         nan 
      0.975         nan 
 
 s2WT4  2010.0 
     23.697         nan 
   1058.561         nan 
      0.864         nan 
    450.800         nan 
      6.939         nan 
      0.976         nan 
 
 s2WT4  3010.0 
     28.033         nan 
   1278.004         nan 
      0.886         nan 
    580.000         nan 
      7.044         nan 
      0.976         nan 
 
 s2WT4  4010.0 
     31.234         nan 
   1406.186         nan 
      0.904         nan 
    676.200         nan 
      7.100         nan 
      0.976         nan 
 
 s2WT4  5010.0 
     34.399         nan 
   1559.844         nan 
      0.913         nan 
    775.500         nan 
      7.145         nan 
      0.976         nan 
 
 s2WT4  6010.0 
     36.544         nan 
   1688.432         nan 
      0.922         nan 
    854.500         nan 
      7.174         nan 
      0.976         nan 
 
 s2WT4  7010.0 
     39.198         nan 
   1785.977         nan 
      0.929         nan 
    932.300         nan 
      7.204         nan 
      0.976         nan 
 
 s2WT4  8010.0 
     40.903         nan 
   1949.321         nan 
      0.933         nan 
    995.800         nan 
      7.212         nan 
      0.976         nan 
 
 s2WT4  9010.0 
     43.147         nan 
   2025.828         nan 
      0.938         nan 
   1063.700         nan 
      7.241         nan 
      0.976         nan 
 
 s2WT4  10010.0 
     44.301         nan 
   2081.704         nan 
      0.942         nan 
   1118.900         nan 
      7.240         nan 
      0.975         nan 
 
 s2WT4  11010.0 
     46.441         nan 
   2208.615         nan 
      0.944         nan 
   1179.300         nan 
      7.249         nan 
      0.975         nan 
 
 s2WT4  12010.0 
     47.792         nan 
   2246.123         nan 
      0.948         nan 
   1222.600         nan 
      7.263         nan 
      0.976         nan 
 
 s2WT4  13010.0 
     49.219         nan 
   2363.831         nan 
      0.950         nan 
   1282.500         nan 
      7.266         nan 
      0.975         nan 
 
 s2WT4  14010.0 
     50.962         nan 
   2455.601         nan 
      0.951         nan 
   1346.300         nan 
      7.302         nan 
      0.976         nan 
 
 s2WT4  15010.0 
     51.772         nan 
   2511.675         nan 
      0.954         nan 
   1379.500         nan 
      7.296         nan 
      0.976         nan 
 
 s2WT4  16010.0 
     52.460         nan 
   2564.858         nan 
      0.956         nan 
   1416.000         nan 
      7.288         nan 
      0.976         nan 
 
 s2WT4  17010.0 
     54.183         nan 
   2569.024         nan 
      0.958         nan 
   1469.800         nan 
      7.310         nan 
      0.976         nan 
 
 s2WT4  18010.0 
     55.179         nan 
   2618.252         nan 
      0.960         nan 
   1501.000         nan 
      7.308         nan 
      0.976         nan 
 
 s2WT4  19010.0 
     56.226         nan 
   2617.412         nan 
      0.961         nan 
   1547.400         nan 
      7.319         nan 
      0.976         nan 
 
 s2WT4  20010.0 
     57.057         nan 
   2729.303         nan 
      0.962         nan 
   1582.300         nan 
      7.318         nan 
      0.976         nan 
 
 s2WT4  21010.0 
     57.820         nan 
   2714.221         nan 
      0.964         nan 
   1618.200         nan 
      7.321         nan 
      0.976         nan 
 
 s2WT4  22010.0 
     59.214         nan 
   2755.693         nan 
      0.964         nan 
   1663.000         nan 
      7.322         nan 
      0.976         nan 
 
 s2WT4  23010.0 
     60.104         nan 
   2809.555         nan 
      0.965         nan 
   1696.800         nan 
      7.330         nan 
      0.976         nan 
 
 s2WT4  24010.0 
     60.968         nan 
   2838.426         nan 
      0.967         nan 
   1725.500         nan 
      7.336         nan 
      0.976         nan 
 
 s2WT4  25010.0 
     61.681         nan 
   2838.884         nan 
      0.968         nan 
   1755.600         nan 
      7.333         nan 
      0.976         nan 
 
 s2WT4  26010.0 
     62.612         nan 
   2879.536         nan 
      0.969         nan 
   1793.600         nan 
      7.337         nan 
      0.976         nan 
 
 s2WT4  27010.0 
     63.259         nan 
   2879.017         nan 
      0.970         nan 
   1819.100         nan 
      7.338         nan 
      0.976         nan 
 
 s2WT5  10.0 
      1.828         nan 
     18.300         nan 
      0.400         nan 
      7.700         nan 
      2.817         nan 
      0.842         nan 
 
 s2WT5  1010.0 
     16.882         nan 
    655.502         nan 
      0.838         nan 
    268.400         nan 
      6.516         nan 
      0.967         nan 
 
 s2WT5  2010.0 
     22.529         nan 
    940.848         nan 
      0.877         nan 
    412.500         nan 
      6.667         nan 
      0.965         nan 
 
 s2WT5  3010.0 
     26.660         nan 
   1216.729         nan 
      0.896         nan 
    522.400         nan 
      6.756         nan 
      0.966         nan 
 
 s2WT5  4010.0 
     29.619         nan 
   1319.644         nan 
      0.911         nan 
    617.100         nan 
      6.805         nan 
      0.966         nan 
 
 s2WT5  5010.0 
     32.847         nan 
   1561.881         nan 
      0.918         nan 
    710.400         nan 
      6.855         nan 
      0.966         nan 
 
 s2WT5  6010.0 
     35.197         nan 
   1628.208         nan 
      0.925         nan 
    793.100         nan 
      6.898         nan 
      0.966         nan 
 
 s2WT5  7010.0 
     36.681         nan 
   1764.665         nan 
      0.932         nan 
    851.400         nan 
      6.911         nan 
      0.967         nan 
 
 s2WT5  8010.0 
     39.263         nan 
   1879.831         nan 
      0.935         nan 
    936.000         nan 
      6.945         nan 
      0.966         nan 
 
 s2WT5  9010.0 
     40.838         nan 
   2002.232         nan 
      0.940         nan 
    989.200         nan 
      6.963         nan 
      0.966         nan 
 
 s2WT5  10010.0 
     42.423         nan 
   2045.119         nan 
      0.944         nan 
   1040.900         nan 
      6.959         nan 
      0.966         nan 
 
 s2WT5  11010.0 
     44.140         nan 
   2128.463         nan 
      0.946         nan 
   1104.700         nan 
      6.988         nan 
      0.967         nan 
 
 s2WT5  12010.0 
     45.788         nan 
   2192.643         nan 
      0.949         nan 
   1159.700         nan 
      6.996         nan 
      0.967         nan 
 
 s2WT5  13010.0 
     46.954         nan 
   2303.729         nan 
      0.951         nan 
   1206.600         nan 
      6.994         nan 
      0.966         nan 
 
 s2WT5  14010.0 
     48.575         nan 
   2326.017         nan 
      0.953         nan 
   1256.900         nan 
      7.013         nan 
      0.967         nan 
 
 s2WT5  15010.0 
     49.617         nan 
   2351.145         nan 
      0.955         nan 
   1298.400         nan 
      7.012         nan 
      0.966         nan 
 
 s2WT5  16010.0 
     51.062         nan 
   2511.211         nan 
      0.956         nan 
   1354.000         nan 
      7.031         nan 
      0.967         nan 
 
 s2WT5  17010.0 
     52.031         nan 
   2494.358         nan 
      0.958         nan 
   1392.500         nan 
      7.032         nan 
      0.966         nan 
 
 s2WT5  18010.0 
     53.019         nan 
   2521.810         nan 
      0.960         nan 
   1424.900         nan 
      7.027         nan 
      0.966         nan 
 
 s2WT5  19010.0 
     54.200         nan 
   2608.856         nan 
      0.961         nan 
   1470.400         nan 
      7.048         nan 
      0.967         nan 
 
 s2WT5  20010.0 
     54.857         nan 
   2596.553         nan 
      0.963         nan 
   1503.800         nan 
      7.048         nan 
      0.967         nan 
 
 s2WT5  21010.0 
     55.856         nan 
   2672.786         nan 
      0.964         nan 
   1537.000         nan 
      7.048         nan 
      0.967         nan 
 
 s2WT5  22010.0 
     56.750         nan 
   2738.646         nan 
      0.965         nan 
   1583.100         nan 
      7.056         nan 
      0.967         nan 
 
 s2WT5  23010.0 
     58.001         nan 
   2772.131         nan 
      0.966         nan 
   1620.200         nan 
      7.056         nan 
      0.967         nan 
 
 s2WT5  24010.0 
     58.453         nan 
   2786.387         nan 
      0.967         nan 
   1645.000         nan 
      7.060         nan 
      0.967         nan 
 
 s2WT5  25010.0 
     59.263         nan 
   2773.554         nan 
      0.968         nan 
   1678.800         nan 
      7.062         nan 
      0.967         nan 
 
 s2WT5  26010.0 
     60.084         nan 
   2807.475         nan 
      0.969         nan 
   1707.100         nan 
      7.063         nan 
      0.966         nan 
 
 s2WT5  27010.0 
     61.126         nan 
   2881.910         nan 
      0.970         nan 
   1744.700         nan 
      7.068         nan 
      0.967         nan 
 
 s2WT6  10.0 
      1.927         nan 
     30.750         nan 
      0.210         nan 
      8.900         nan 
      3.094         nan 
      0.876         nan 
 
 s2WT6  1010.0 
     17.816         nan 
    737.869         nan 
      0.826         nan 
    276.200         nan 
      6.685         nan 
      0.977         nan 
 
 s2WT6  2010.0 
     23.267         nan 
   1031.906         nan 
      0.873         nan 
    415.600         nan 
      6.885         nan 
      0.978         nan 
 
 s2WT6  3010.0 
     28.051         nan 
   1234.599         nan 
      0.892         nan 
    542.900         nan 
      7.011         nan 
      0.979         nan 
 
 s2WT6  4010.0 
     30.650         nan 
   1442.825         nan 
      0.907         nan 
    635.900         nan 
      7.060         nan 
      0.979         nan 
 
 s2WT6  5010.0 
     34.542         nan 
   1644.907         nan 
      0.913         nan 
    735.000         nan 
      7.093         nan 
      0.978         nan 
 
 s2WT6  6010.0 
     37.083         nan 
   1749.065         nan 
      0.921         nan 
    823.100         nan 
      7.143         nan 
      0.979         nan 
 
 s2WT6  7010.0 
     39.358         nan 
   1850.472         nan 
      0.927         nan 
    897.700         nan 
      7.171         nan 
      0.979         nan 
 
 s2WT6  8010.0 
     41.125         nan 
   1912.580         nan 
      0.933         nan 
    959.600         nan 
      7.178         nan 
      0.979         nan 
 
 s2WT6  9010.0 
     43.007         nan 
   2071.765         nan 
      0.937         nan 
   1030.000         nan 
      7.204         nan 
      0.979         nan 
 
 s2WT6  10010.0 
     44.815         nan 
   2197.622         nan 
      0.940         nan 
   1086.800         nan 
      7.208         nan 
      0.979         nan 
 
 s2WT6  11010.0 
     46.943         nan 
   2227.472         nan 
      0.943         nan 
   1155.400         nan 
      7.237         nan 
      0.979         nan 
 
 s2WT6  12010.0 
     48.306         nan 
   2380.993         nan 
      0.945         nan 
   1215.700         nan 
      7.232         nan 
      0.979         nan 
 
 s2WT6  13010.0 
     49.493         nan 
   2366.427         nan 
      0.949         nan 
   1258.100         nan 
      7.242         nan 
      0.979         nan 
 
 s2WT6  14010.0 
     50.968         nan 
   2416.432         nan 
      0.951         nan 
   1309.600         nan 
      7.253         nan 
      0.979         nan 
 
 s2WT6  15010.0 
     52.105         nan 
   2473.552         nan 
      0.953         nan 
   1353.900         nan 
      7.262         nan 
      0.979         nan 
 
 s2WT6  16010.0 
     53.807         nan 
   2578.932         nan 
      0.954         nan 
   1407.900         nan 
      7.274         nan 
      0.979         nan 
 
 s2WT6  17010.0 
     54.975         nan 
   2614.033         nan 
      0.956         nan 
   1450.000         nan 
      7.268         nan 
      0.979         nan 
 
 s2WT6  18010.0 
     56.131         nan 
   2615.812         nan 
      0.958         nan 
   1495.200         nan 
      7.286         nan 
      0.979         nan 
 
 s2WT6  19010.0 
     57.172         nan 
   2687.934         nan 
      0.959         nan 
   1538.500         nan 
      7.286         nan 
      0.979         nan 
 
 s2WT6  20010.0 
     58.271         nan 
   2785.290         nan 
      0.960         nan 
   1582.700         nan 
      7.291         nan 
      0.979         nan 
 
 s2WT6  21010.0 
     59.148         nan 
   2806.238         nan 
      0.962         nan 
   1617.100         nan 
      7.293         nan 
      0.979         nan 
 
 s2WT6  22010.0 
     59.945         nan 
   2789.567         nan 
      0.963         nan 
   1647.700         nan 
      7.295         nan 
      0.979         nan 
 
 s2WT6  23010.0 
     60.930         nan 
   2848.834         nan 
      0.965         nan 
   1682.900         nan 
      7.299         nan 
      0.979         nan 
 
 s2WT6  24010.0 
     62.000         nan 
   2878.772         nan 
      0.966         nan 
   1715.600         nan 
      7.301         nan 
      0.979         nan 
 
 s2WT6  25010.0 
     62.369         nan 
   2896.923         nan 
      0.967         nan 
   1745.400         nan 
      7.302         nan 
      0.979         nan 
 
 s2WT6  26010.0 
     63.603         nan 
   2969.668         nan 
      0.967         nan 
   1784.800         nan 
      7.311         nan 
      0.979         nan 
 
 s2WT6  27010.0 
     64.378         nan 
   2978.099         nan 
      0.968         nan 
   1818.700         nan 
      7.314         nan 
      0.979         nan 
 
 s2WT7  10.0 
      1.503         nan 
     20.900         nan 
      0.360         nan 
      7.900         nan 
      2.852         nan 
      0.844         nan 
 
 s2WT7  1010.0 
     17.058         nan 
    641.777         nan 
      0.834         nan 
    267.800         nan 
      6.426         nan 
      0.967         nan 
 
 s2WT7  2010.0 
     21.955         nan 
    949.482         nan 
      0.880         nan 
    401.100         nan 
      6.586         nan 
      0.967         nan 
 
 s2WT7  3010.0 
     25.217         nan 
   1108.839         nan 
      0.902         nan 
    503.300         nan 
      6.670         nan 
      0.967         nan 
 
 s2WT7  4010.0 
     28.833         nan 
   1251.354         nan 
      0.914         nan 
    606.400         nan 
      6.733         nan 
      0.967         nan 
 
 s2WT7  5010.0 
     31.303         nan 
   1484.377         nan 
      0.921         nan 
    687.000         nan 
      6.757         nan 
      0.967         nan 
 
 s2WT7  6010.0 
     33.957         nan 
   1561.792         nan 
      0.928         nan 
    763.400         nan 
      6.805         nan 
      0.968         nan 
 
 s2WT7  7010.0 
     35.189         nan 
   1619.361         nan 
      0.936         nan 
    816.300         nan 
      6.800         nan 
      0.967         nan 
 
 s2WT7  8010.0 
     37.713         nan 
   1731.935         nan 
      0.939         nan 
    887.500         nan 
      6.817         nan 
      0.967         nan 
 
 s2WT7  9010.0 
     39.134         nan 
   1739.487         nan 
      0.944         nan 
    948.800         nan 
      6.855         nan 
      0.968         nan 
 
 s2WT7  10010.0 
     40.708         nan 
   1858.937         nan 
      0.947         nan 
   1003.900         nan 
      6.853         nan 
      0.967         nan 
 
 s2WT7  11010.0 
     41.964         nan 
   1999.274         nan 
      0.949         nan 
   1053.300         nan 
      6.873         nan 
      0.967         nan 
 
 s2WT7  12010.0 
     43.509         nan 
   2051.712         nan 
      0.952         nan 
   1104.400         nan 
      6.878         nan 
      0.967         nan 
 
 s2WT7  13010.0 
     45.459         nan 
   2091.559         nan 
      0.955         nan 
   1149.900         nan 
      6.889         nan 
      0.968         nan 
 
 s2WT7  14010.0 
     46.158         nan 
   2177.220         nan 
      0.957         nan 
   1192.500         nan 
      6.886         nan 
      0.967         nan 
 
 s2WT7  15010.0 
     47.575         nan 
   2221.388         nan 
      0.958         nan 
   1239.300         nan 
      6.915         nan 
      0.968         nan 
 
 s2WT7  16010.0 
     48.670         nan 
   2317.460         nan 
      0.960         nan 
   1280.400         nan 
      6.912         nan 
      0.968         nan 
 
 s2WT7  17010.0 
     49.251         nan 
   2374.192         nan 
      0.961         nan 
   1319.400         nan 
      6.918         nan 
      0.968         nan 
 
 s2WT7  18010.0 
     50.667         nan 
   2451.576         nan 
      0.962         nan 
   1362.100         nan 
      6.920         nan 
      0.968         nan 
 
 s2WT7  19010.0 
     51.637         nan 
   2499.341         nan 
      0.964         nan 
   1396.500         nan 
      6.924         nan 
      0.968         nan 
 
 s2WT7  20010.0 
     52.722         nan 
   2475.280         nan 
      0.965         nan 
   1429.300         nan 
      6.932         nan 
      0.968         nan 
 
 s2WT7  21010.0 
     53.181         nan 
   2474.488         nan 
      0.967         nan 
   1451.800         nan 
      6.933         nan 
      0.968         nan 
 
 s2WT7  22010.0 
     54.456         nan 
   2579.351         nan 
      0.967         nan 
   1499.900         nan 
      6.937         nan 
      0.968         nan 
 
 s2WT7  23010.0 
     55.097         nan 
   2606.738         nan 
      0.969         nan 
   1521.100         nan 
      6.935         nan 
      0.968         nan 
 
 s2WT7  24010.0 
     55.928         nan 
   2616.536         nan 
      0.969         nan 
   1555.500         nan 
      6.936         nan 
      0.967         nan
[truncated: 453,881 more chars]
