# Supplementary material for: ASMT determines gut microbiota and increases neurobehavioral adaptability to exercise in female mice
Source: Commun Biol. 2023 Nov 7;6:1126. doi: 10.1038/s42003-023-05520-8 (PMC10630421; doi:10.1038/s42003-023-05520-8)
Supplement: Supplementary file 4 — Supplementary Data 1 [file 42003_2023_5520_MOESM4_ESM.zip › 4.Alpha_Diversity/rank_abundance/RankAbundance.pdf]

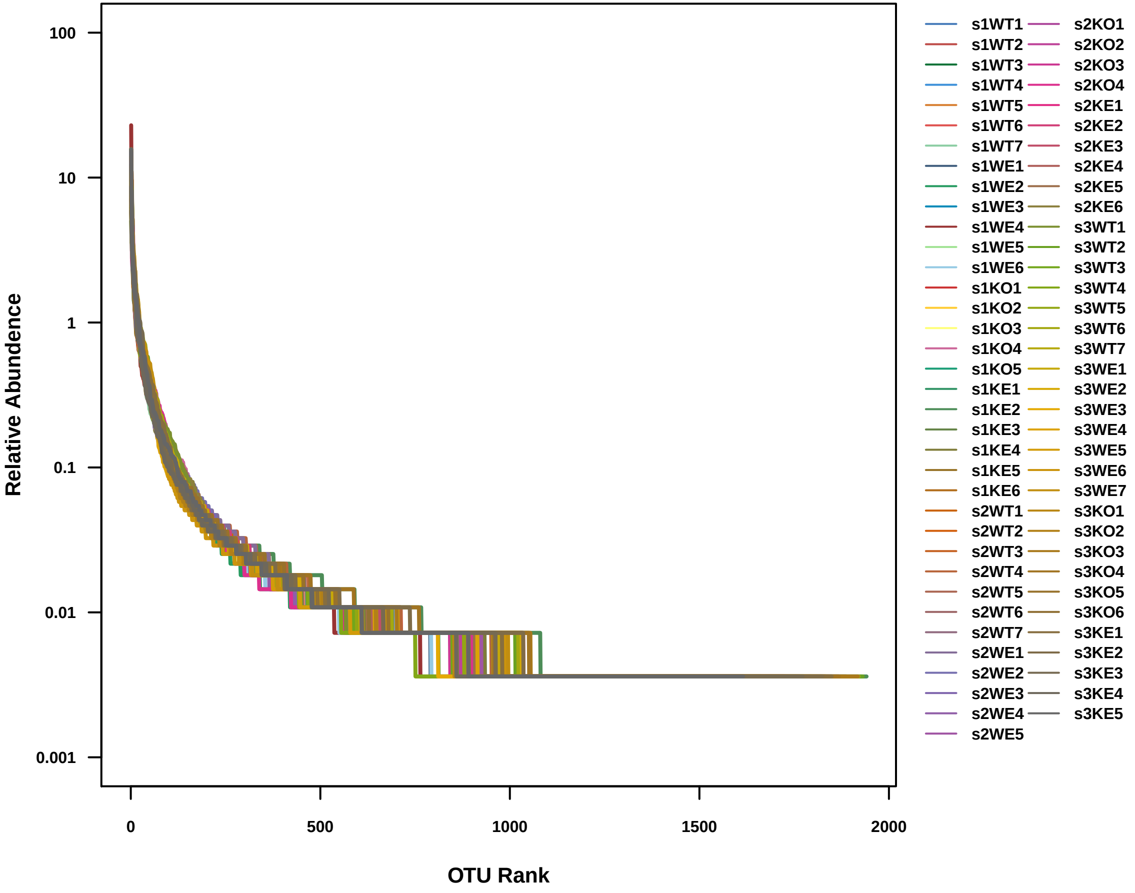
Relative Abundence
100
10
1
0.1
0.01
0.001
0
500
1000
1500
2000
OTU Rank
s1WT1
s1WT2
s1WT3
s1WT4
s1WT5
s1WT6
s1WT7
s1WE1
s1WE2
s1WE3
s1WE4
s1WE5
s1WE6
s1KO1
s1KO2
s1KO3
s1KO4
s1KO5
s1KE1
s1KE2
s1KE3
s1KE4
s1KE5
s1KE6
s2WT1
s2WT2
s2WT3
s2WT4
s2WT5
s2WT6
s2WT7
s2WE1
s2WE2
s2WE3
s2WE4
s2WE5
s2KO1
s2KO2
s2KO3
s2KO4
s2KE1
s2KE2
s2KE3
s2KE4
s2KE5
s2KE6
s3WT1
s3WT2
s3WT3
s3WT4
s3WT5
s3WT6
s3WT7
s3WE1
s3WE2
s3WE3
s3WE4
s3WE5
s3WE6
s3WE7
s3KO1
s3KO2
s3KO3
s3KO4
s3KO5
s3KO6
s3KE1
s3KE2
s3KE3
s3KE4
s3KE5
