# Supplementary figures and images for: ASMT determines gut microbiota and increases neurobehavioral adaptability to exercise in female mice (part 2 of 2)
Source: Commun Biol. 2023 Nov 7;6:1126. doi: 10.1038/s42003-023-05520-8 (PMC10630421; doi:10.1038/s42003-023-05520-8)

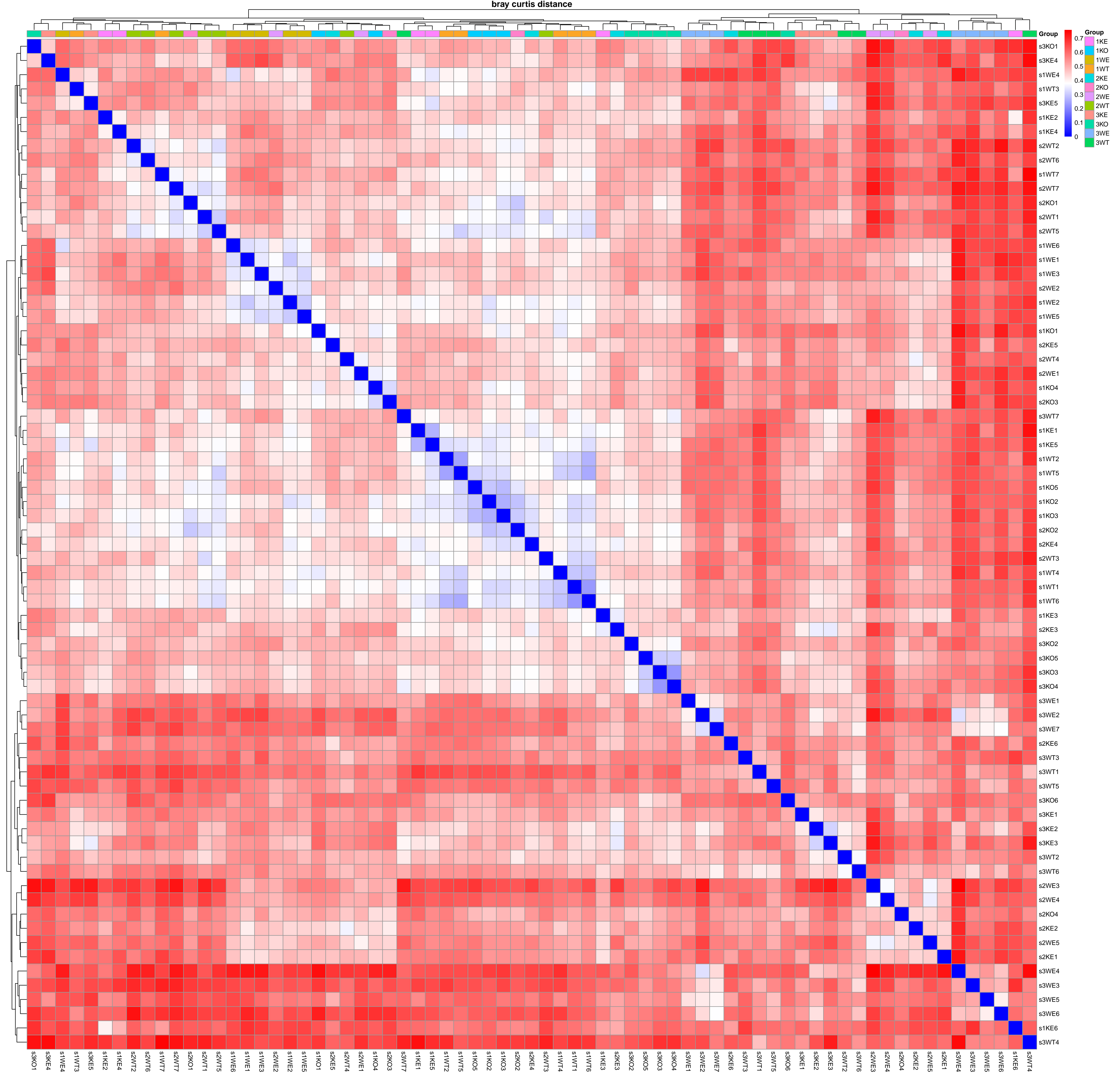

Supplement: Supplementary file 4 — Supplementary Data 1 [file 42003_2023_5520_MOESM4_ESM.zip › 5.Beta_Diversity/Distance/bray_curtis_distance.pdf]

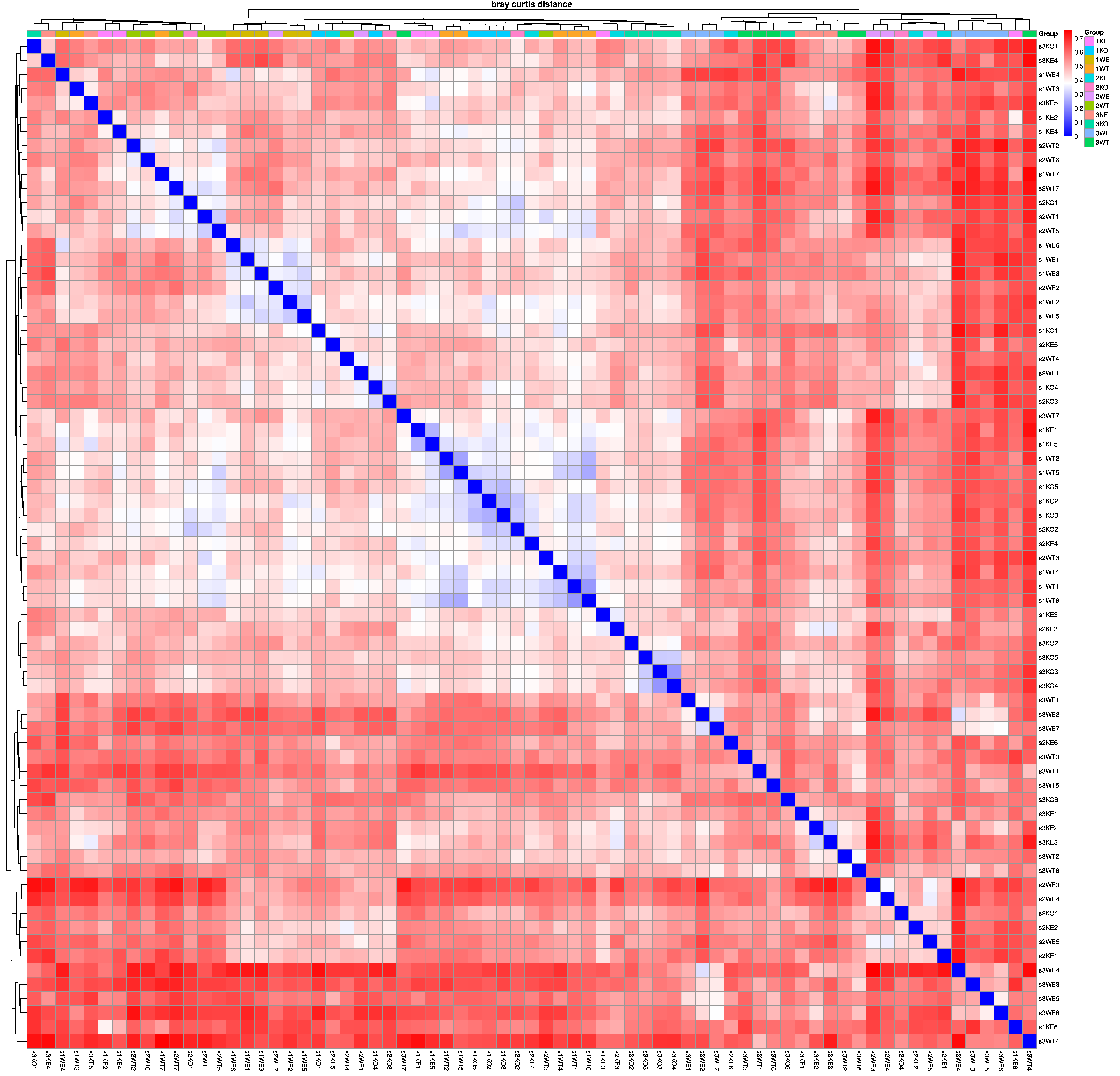

Supplement: Supplementary file 4 — Supplementary Data 1 [file 42003_2023_5520_MOESM4_ESM.zip › 5.Beta_Diversity/Distance/bray_curtis_distance.png]

euclidean distance

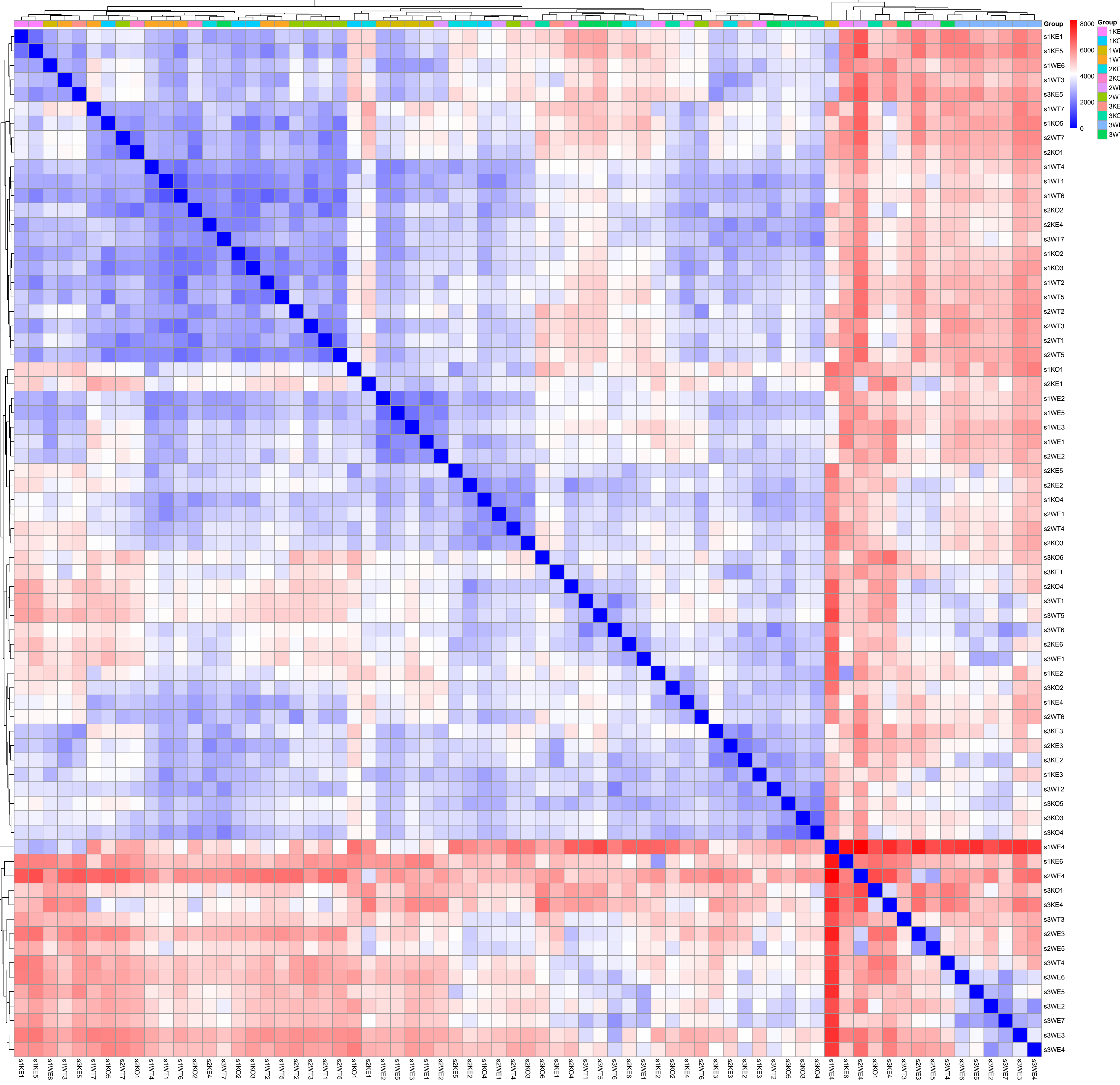

Supplement: Supplementary file 4 — Supplementary Data 1 [file 42003_2023_5520_MOESM4_ESM.zip › 5.Beta_Diversity/Distance/euclidean_distance.pdf]

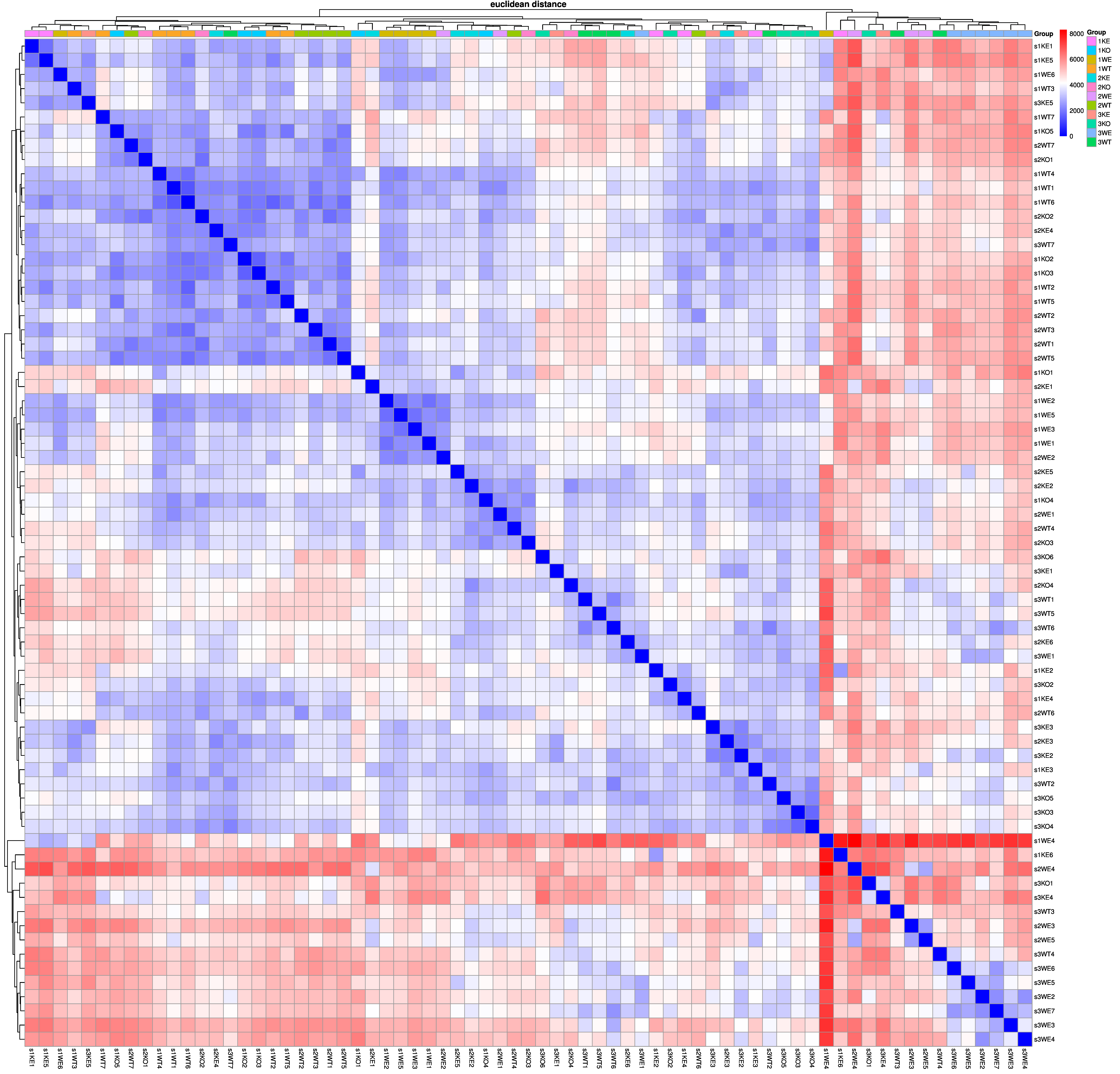

Supplement: Supplementary file 4 — Supplementary Data 1 [file 42003_2023_5520_MOESM4_ESM.zip › 5.Beta_Diversity/Distance/euclidean_distance.png]

unweighted unifracs distance

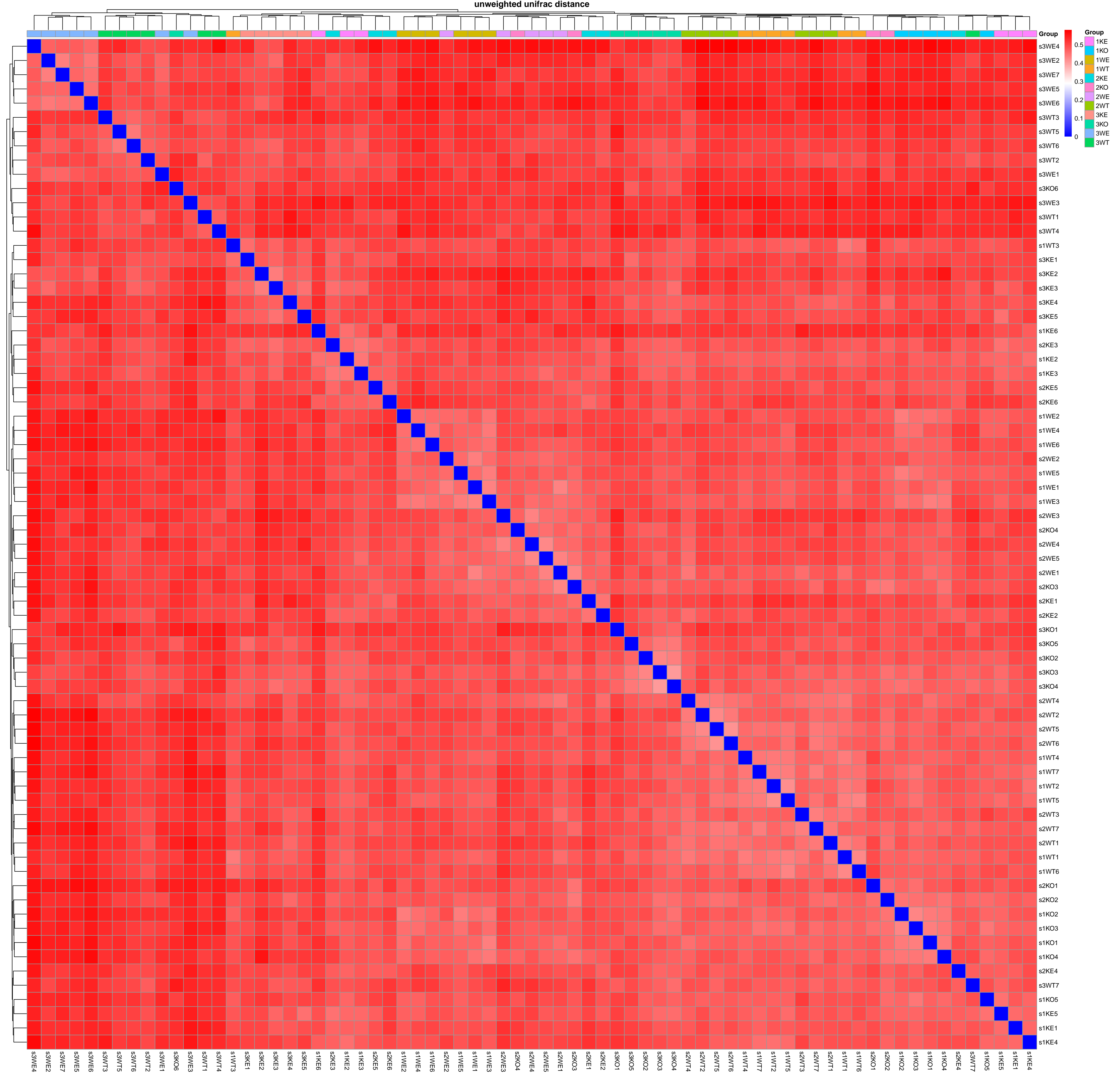

Supplement: Supplementary file 4 — Supplementary Data 1 [file 42003_2023_5520_MOESM4_ESM.zip › 5.Beta_Diversity/Distance/unweighted_unifrac_distance.pdf]

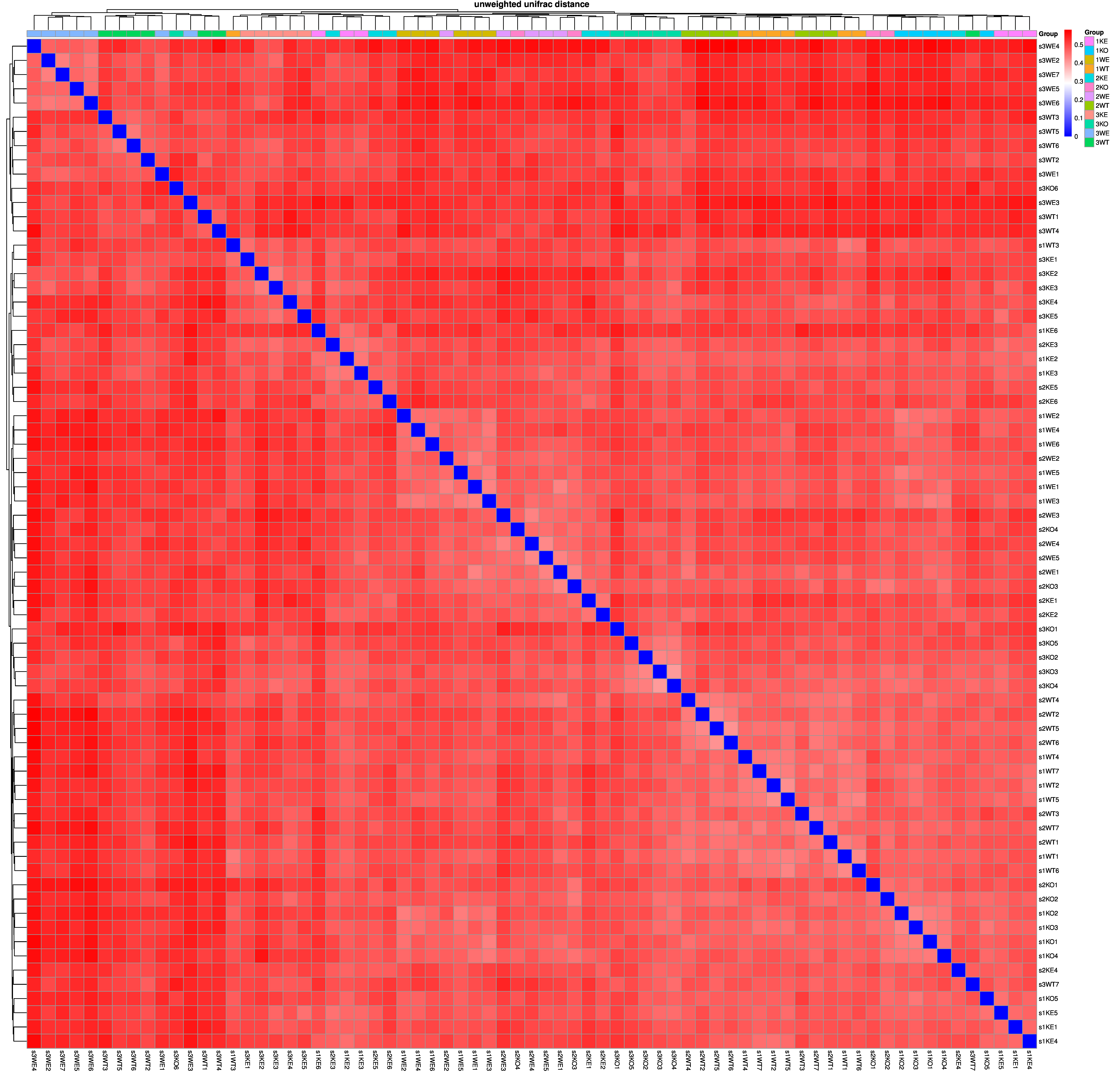

Supplement: Supplementary file 4 — Supplementary Data 1 [file 42003_2023_5520_MOESM4_ESM.zip › 5.Beta_Diversity/Distance/unweighted_unifrac_distance.png]

weighted unifracs distance

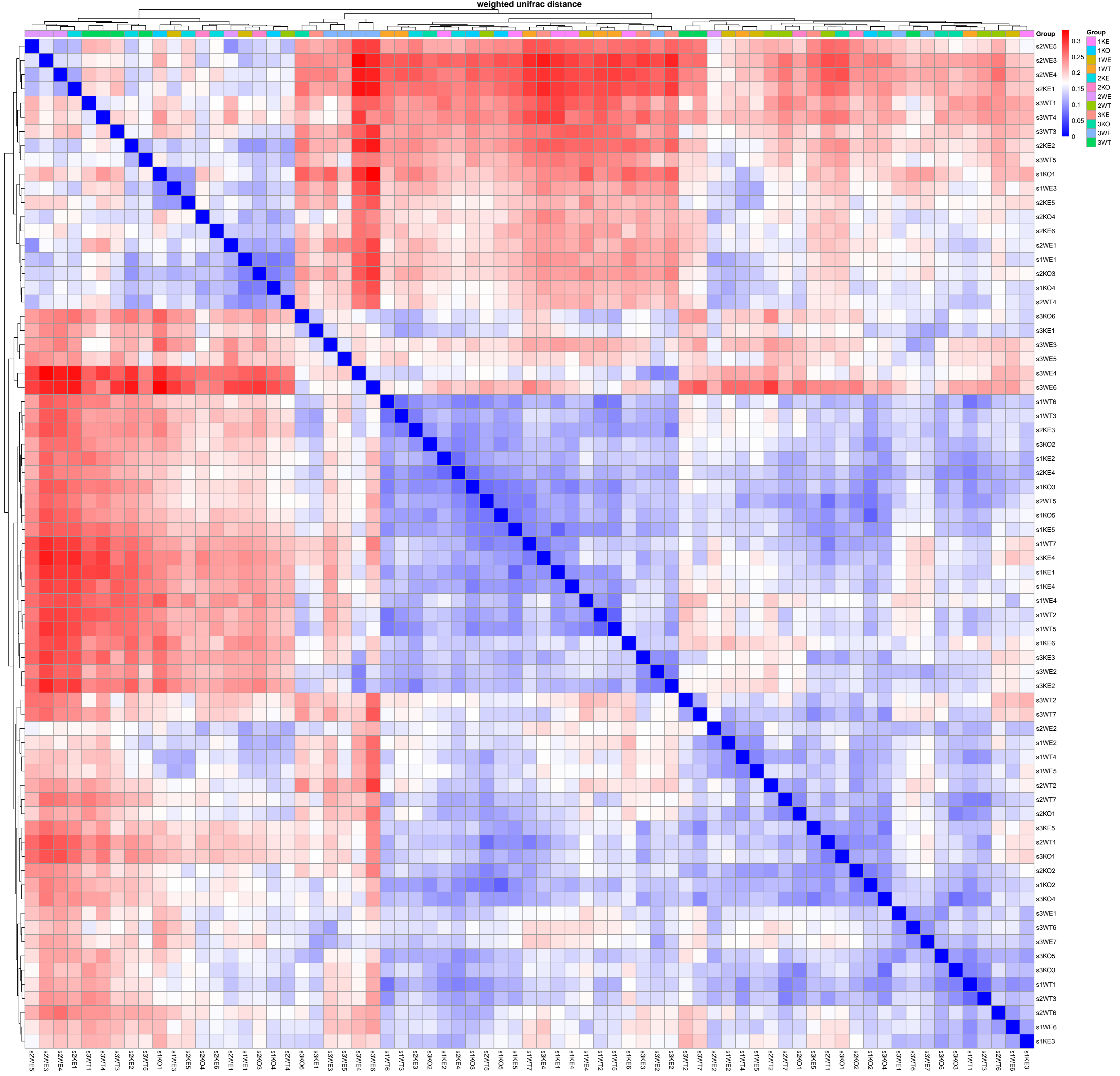

Supplement: Supplementary file 4 — Supplementary Data 1 [file 42003_2023_5520_MOESM4_ESM.zip › 5.Beta_Diversity/Distance/weighted_unifrac_distance.pdf]

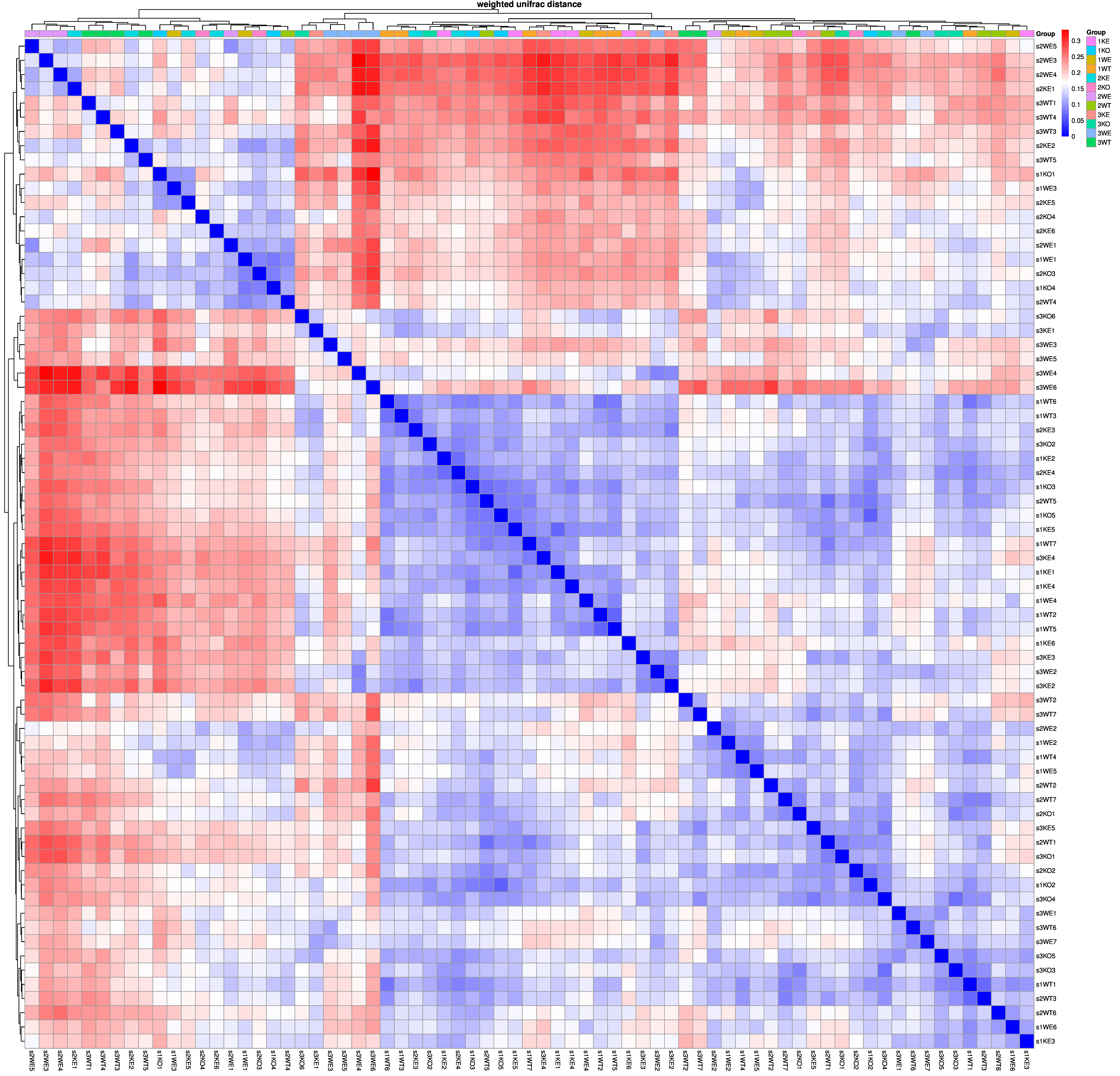

Supplement: Supplementary file 4 — Supplementary Data 1 [file 42003_2023_5520_MOESM4_ESM.zip › 5.Beta_Diversity/Distance/weighted_unifrac_distance.png]

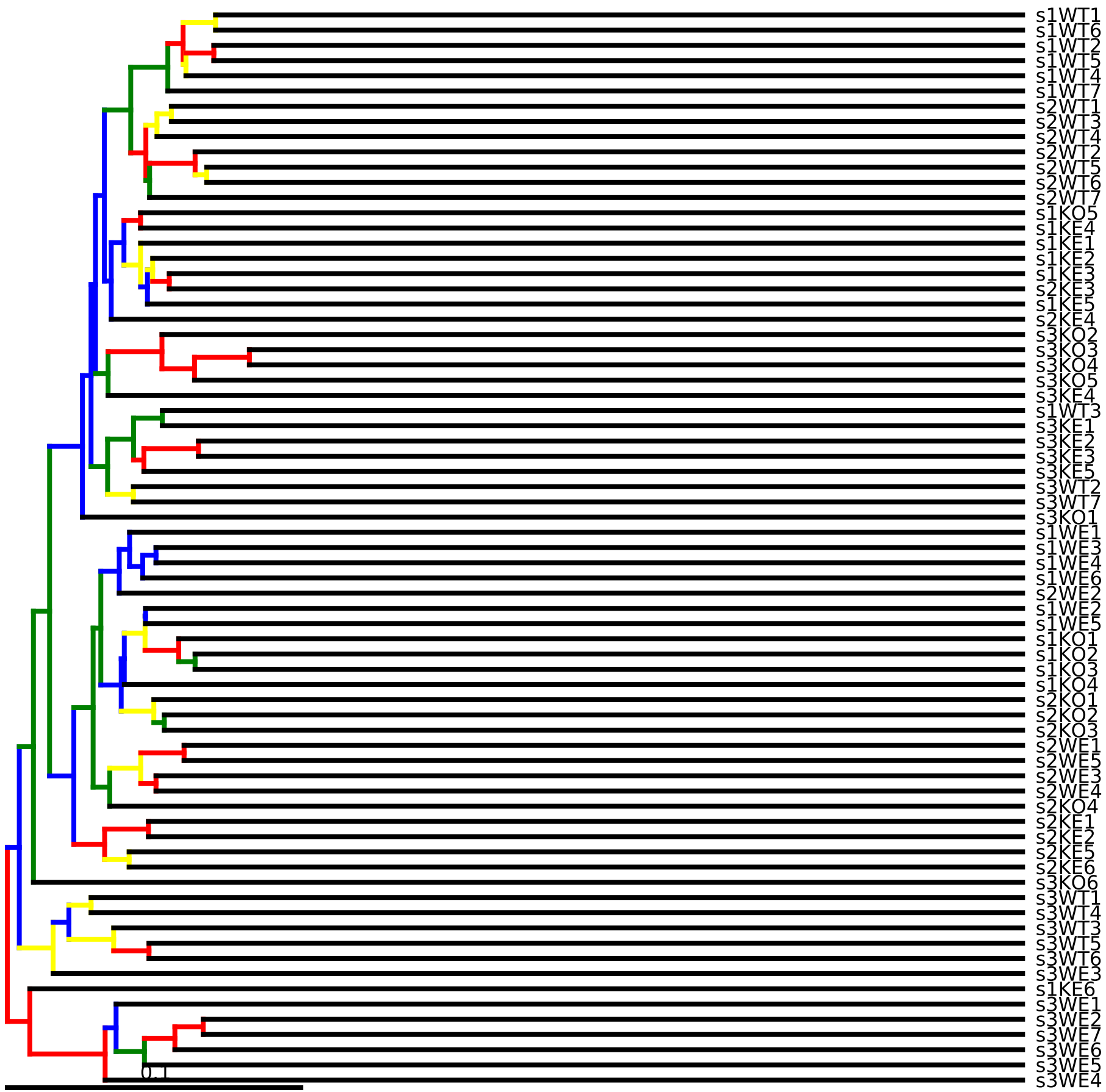

Supplement: Supplementary file 4 — Supplementary Data 1 [file 42003_2023_5520_MOESM4_ESM.zip › 5.Beta_Diversity/Jackknifed/binary_jaccard_tree_compared/colour_binary_jaccard_jackknife_named_nodes.pdf]

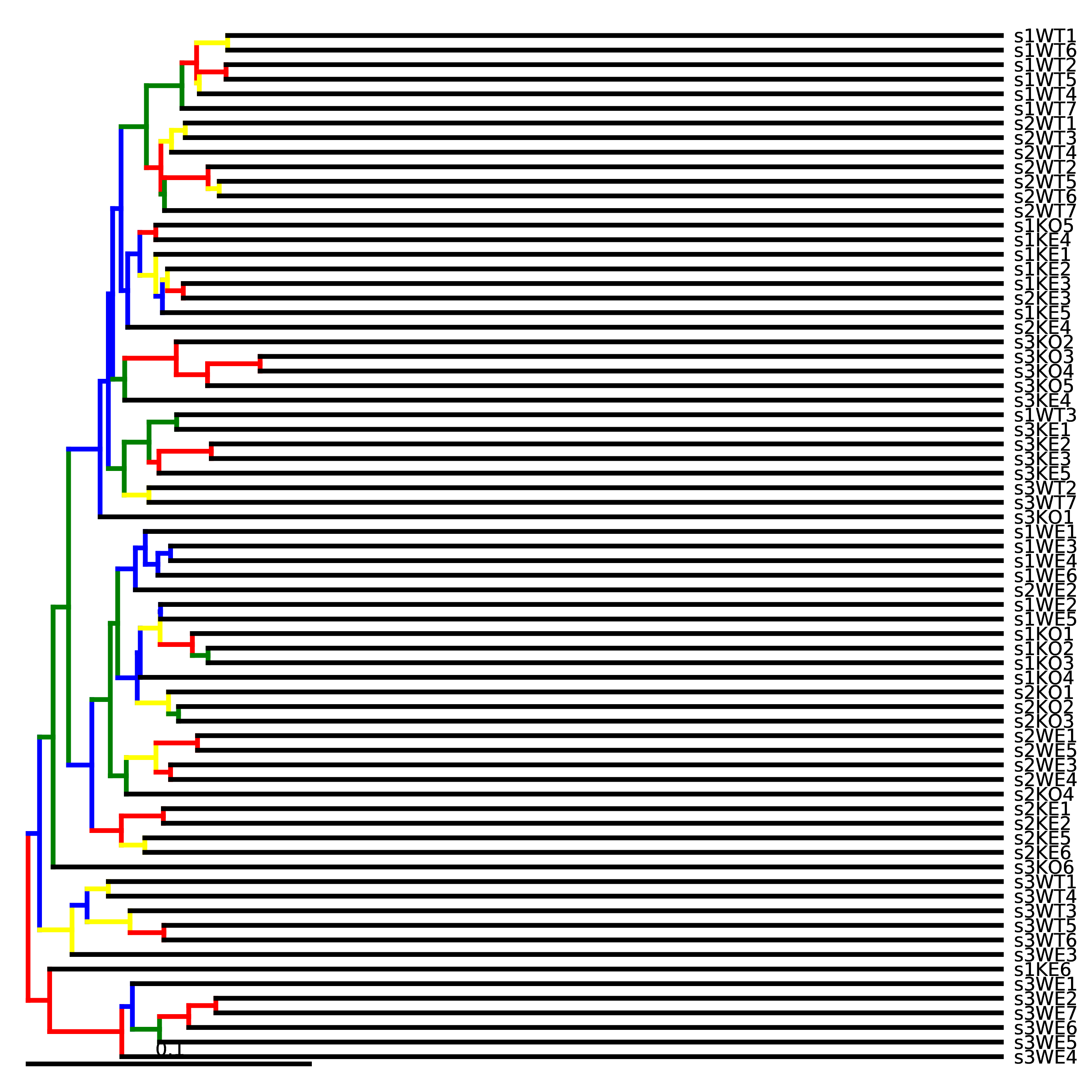

Supplement: Supplementary file 4 — Supplementary Data 1 [file 42003_2023_5520_MOESM4_ESM.zip › 5.Beta_Diversity/Jackknifed/binary_jaccard_tree_compared/colour_binary_jaccard_jackknife_named_nodes.png]
