# Supplementary figures and images for: ASMT determines gut microbiota and increases neurobehavioral adaptability to exercise in female mice (part 1 of 2)
Source: Commun Biol. 2023 Nov 7;6:1126. doi: 10.1038/s42003-023-05520-8 (PMC10630421; doi:10.1038/s42003-023-05520-8)

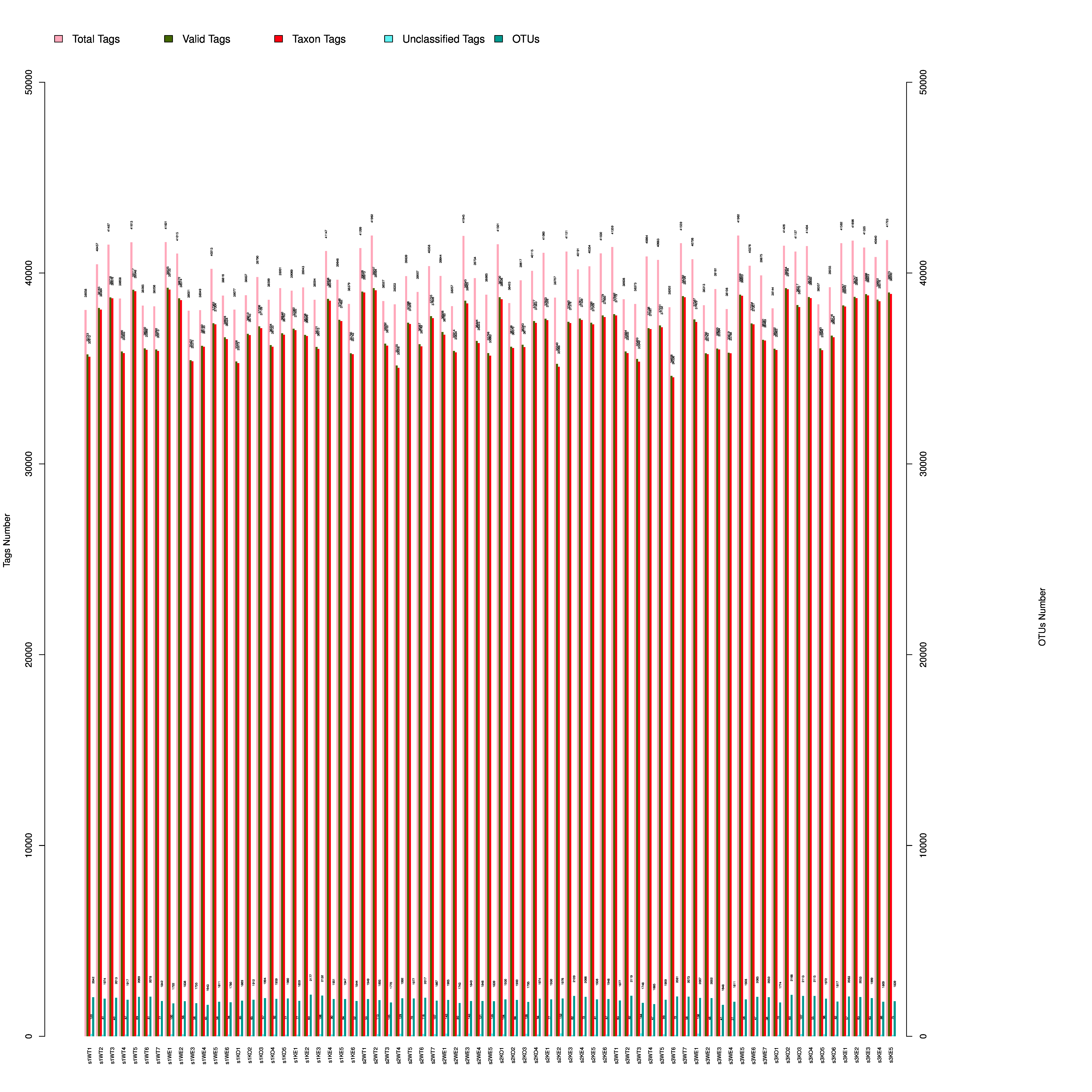

Supplement: Supplementary file 4 — Supplementary Data 1 [file 42003_2023_5520_MOESM4_ESM.zip › 2.OTUs/OTU_level_plot/annotationbarplot_class.png]

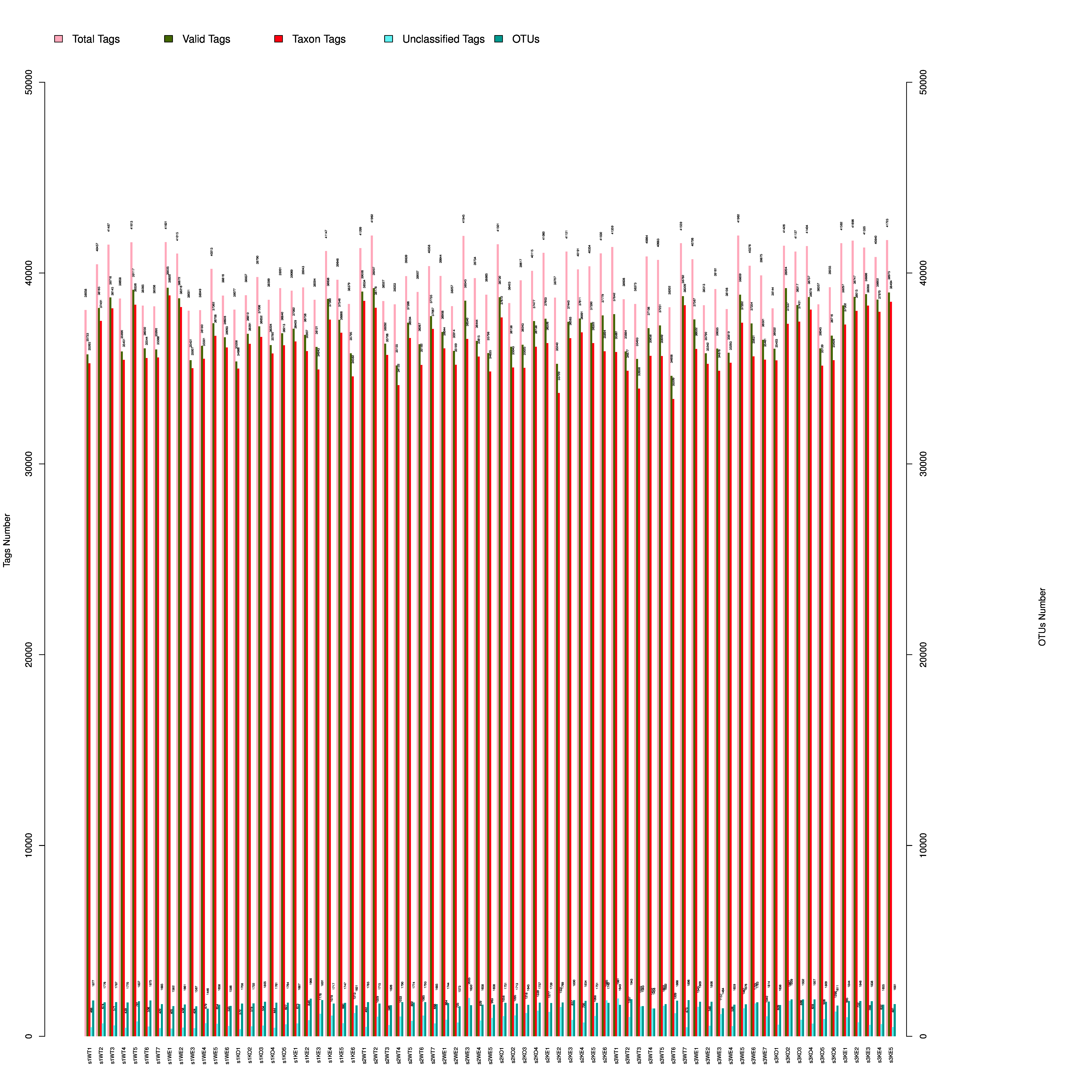

Supplement: Supplementary file 4 — Supplementary Data 1 [file 42003_2023_5520_MOESM4_ESM.zip › 2.OTUs/OTU_level_plot/annotationbarplot_family.png]

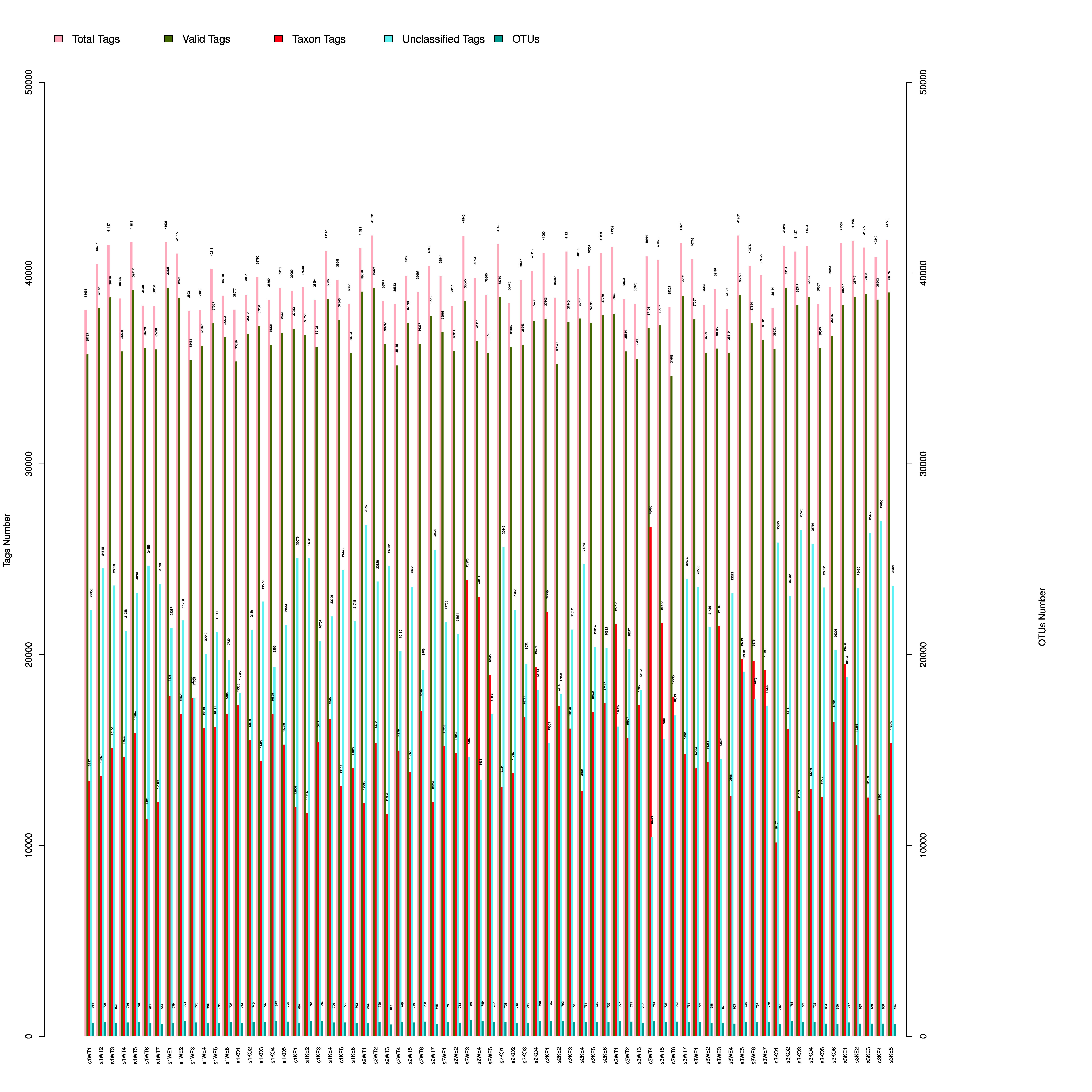

Supplement: Supplementary file 4 — Supplementary Data 1 [file 42003_2023_5520_MOESM4_ESM.zip › 2.OTUs/OTU_level_plot/annotationbarplot_genus.png]

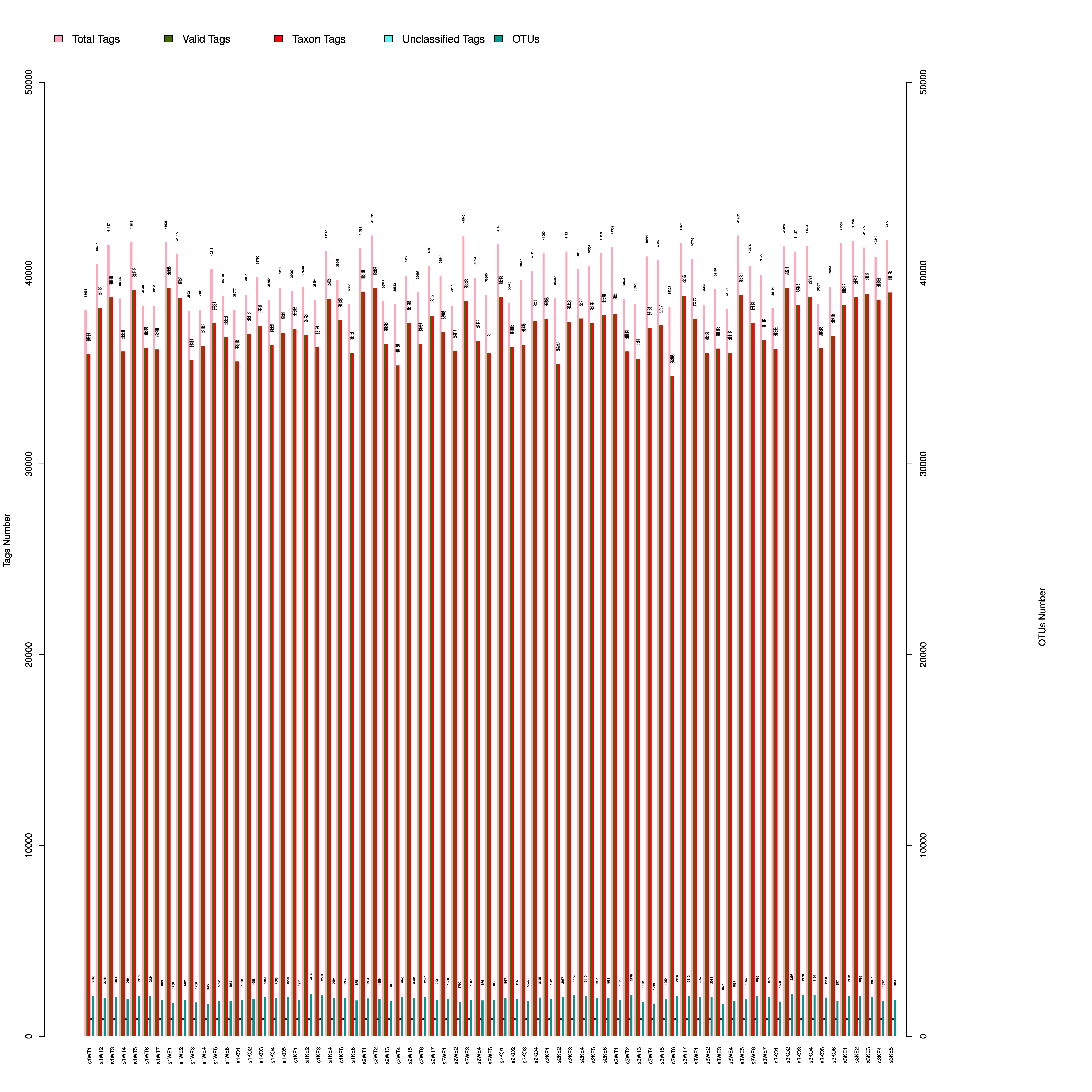

Supplement: Supplementary file 4 — Supplementary Data 1 [file 42003_2023_5520_MOESM4_ESM.zip › 2.OTUs/OTU_level_plot/annotationbarplot_kindom.png]

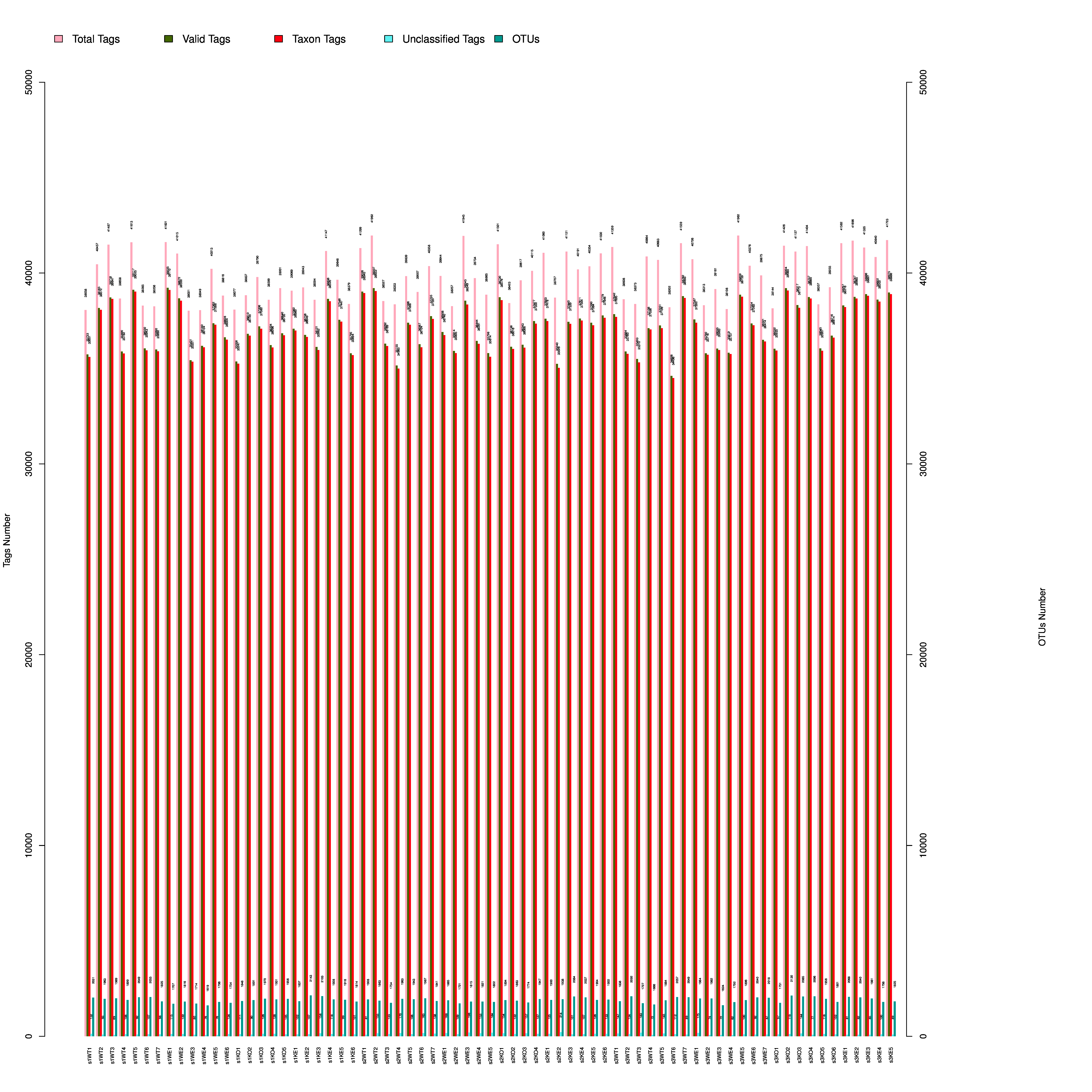

Supplement: Supplementary file 4 — Supplementary Data 1 [file 42003_2023_5520_MOESM4_ESM.zip › 2.OTUs/OTU_level_plot/annotationbarplot_order.png]

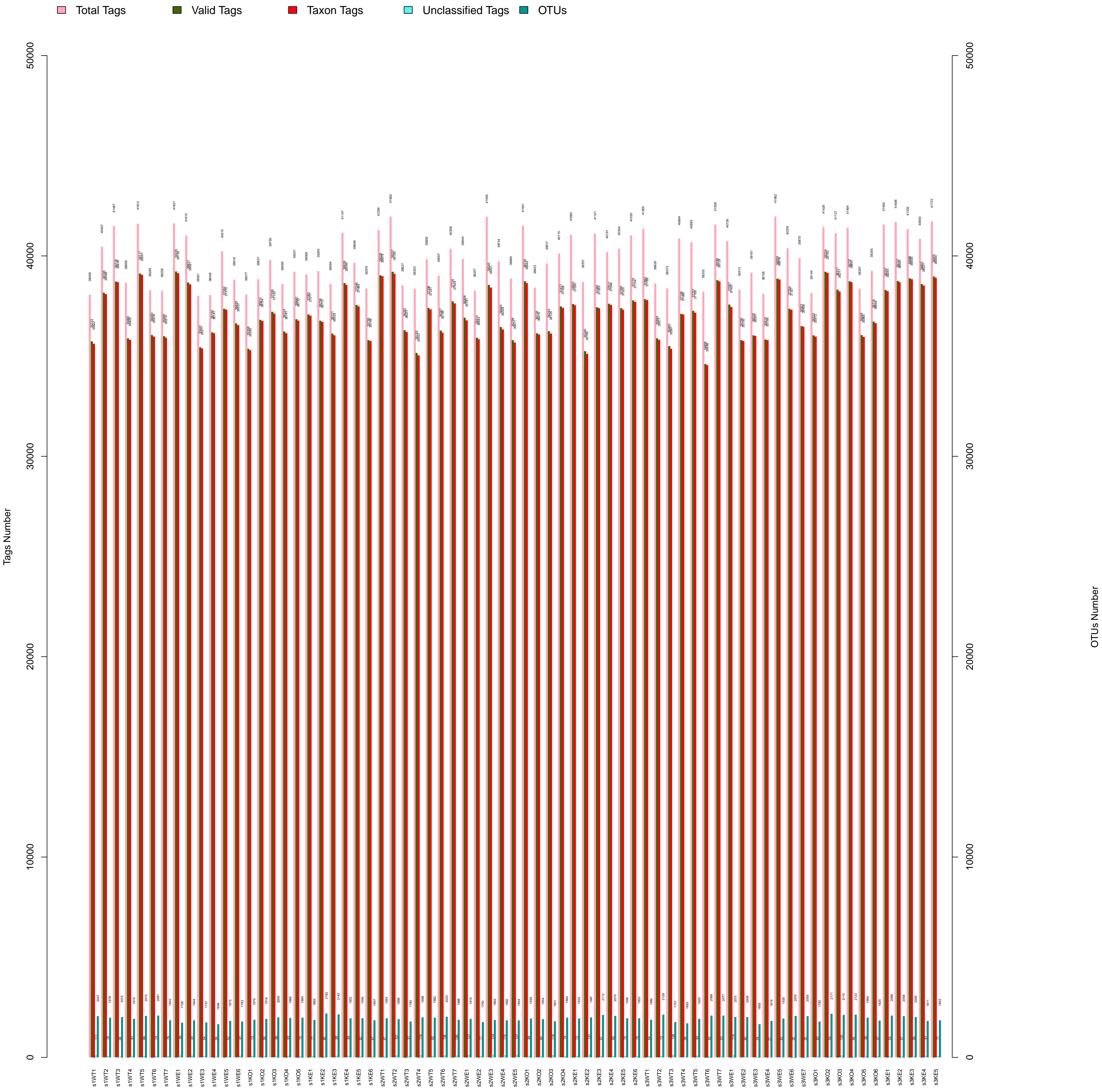

OTUs Number

Supplement: Supplementary file 4 — Supplementary Data 1 [file 42003_2023_5520_MOESM4_ESM.zip › 2.OTUs/OTU_level_plot/annotationbarplot_phylum.pdf]

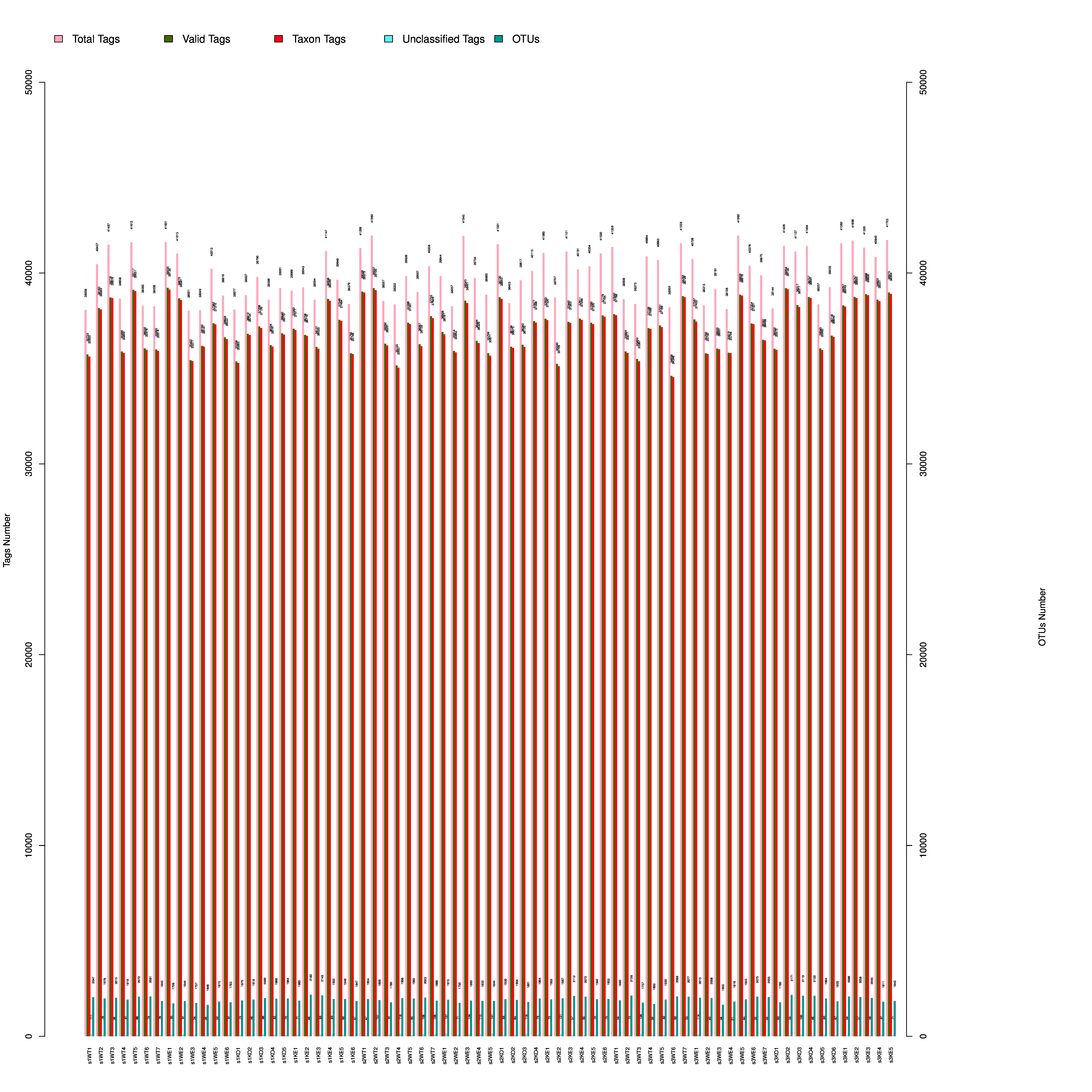

Supplement: Supplementary file 4 — Supplementary Data 1 [file 42003_2023_5520_MOESM4_ESM.zip › 2.OTUs/OTU_level_plot/annotationbarplot_phylum.png]

Tags Number

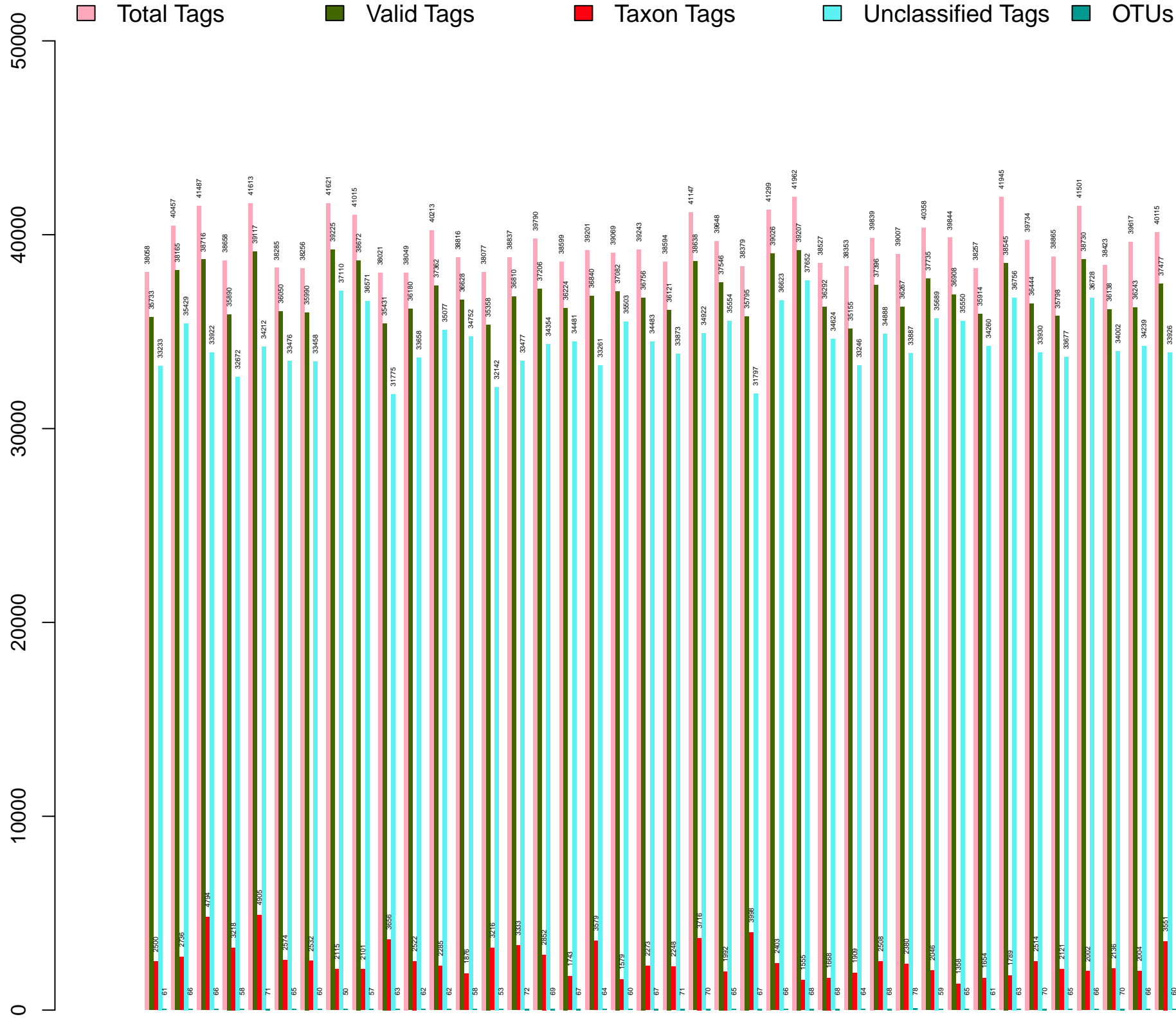

OTUs Number

Supplement: Supplementary file 4 — Supplementary Data 1 [file 42003_2023_5520_MOESM4_ESM.zip › 2.OTUs/OTU_level_plot/annotationbarplot_species.pdf]

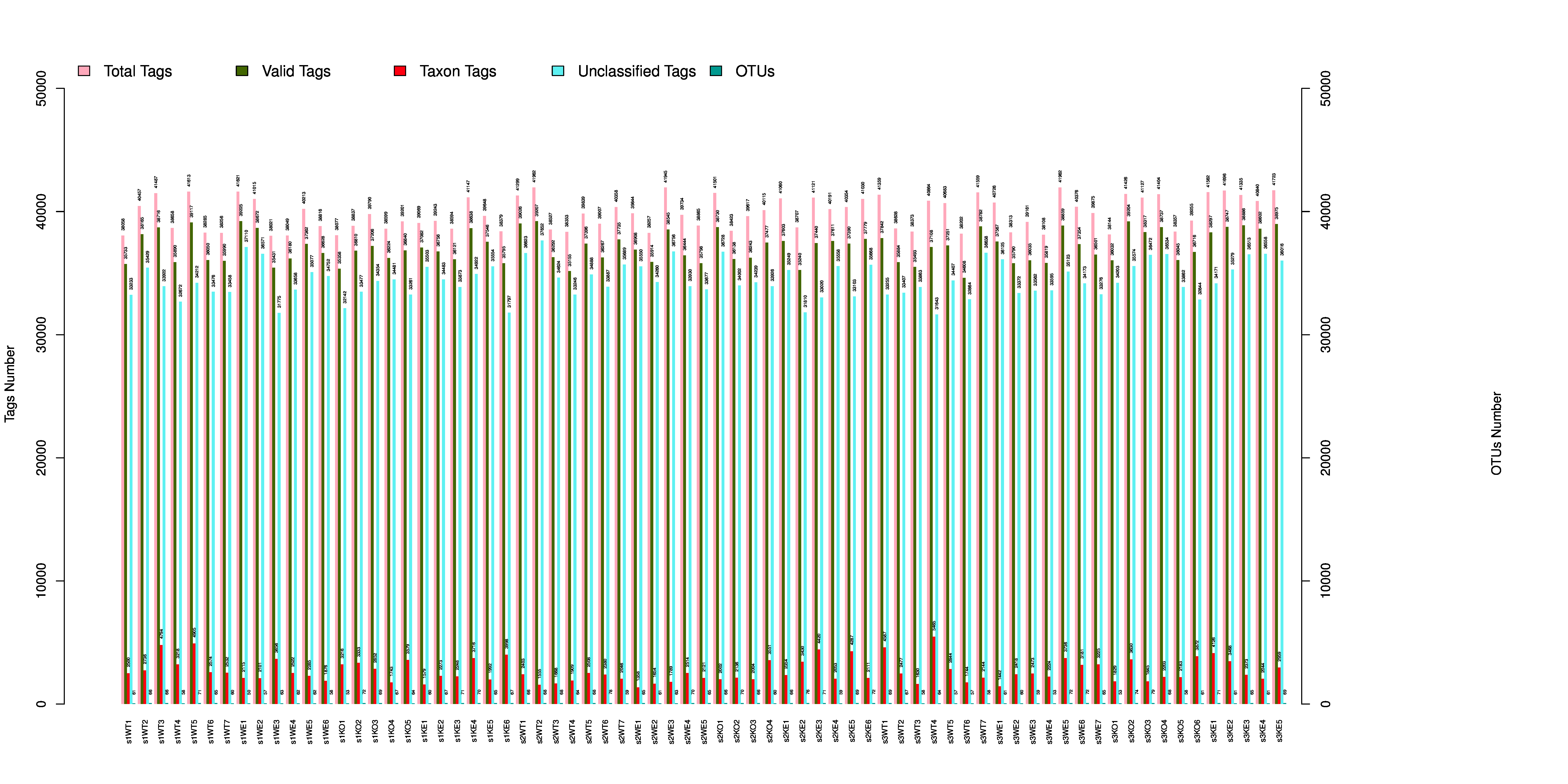

Supplement: Supplementary file 4 — Supplementary Data 1 [file 42003_2023_5520_MOESM4_ESM.zip › 2.OTUs/OTU_level_plot/annotationbarplot_species.png]

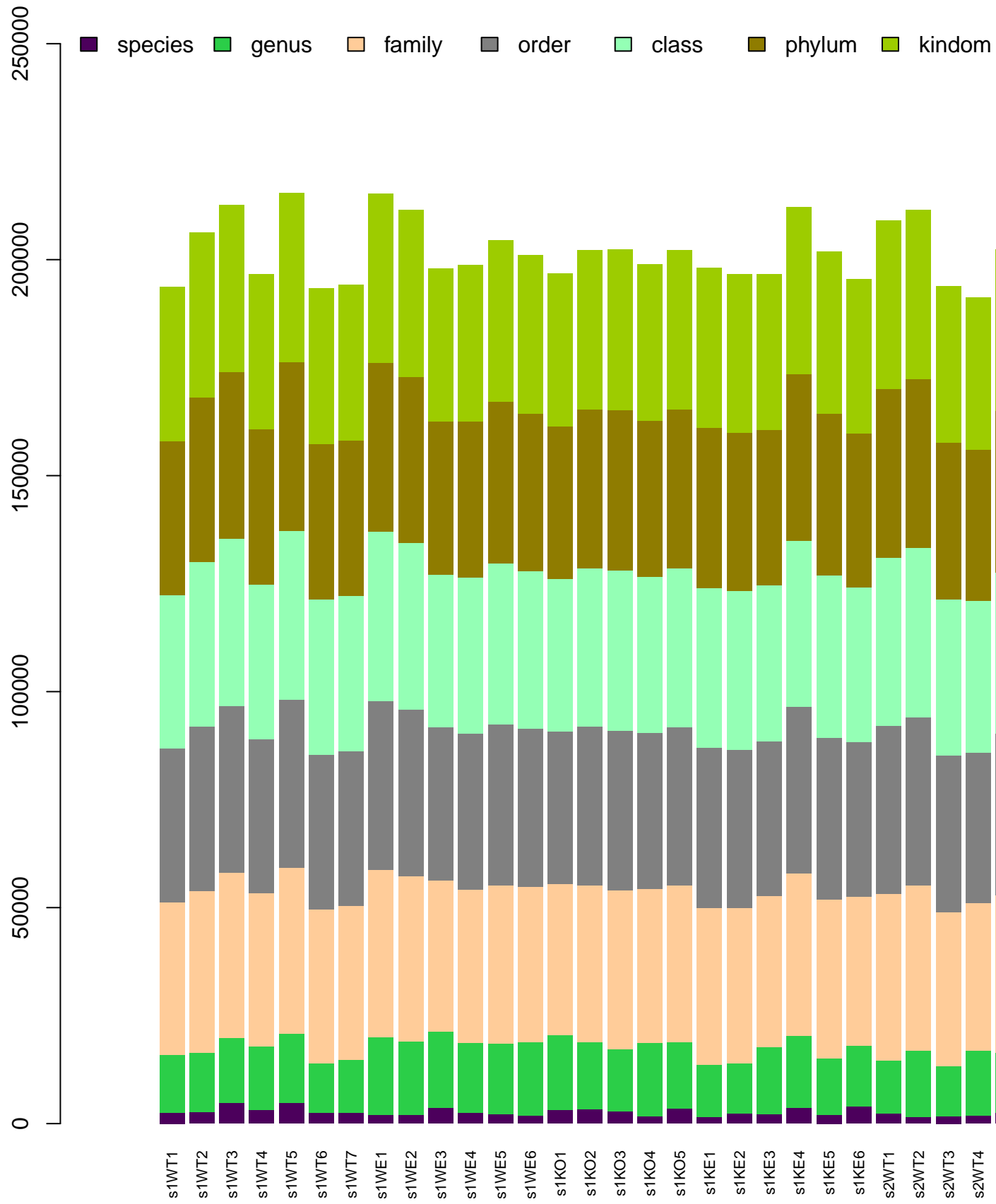

Supplement: Supplementary file 4 — Supplementary Data 1 [file 42003_2023_5520_MOESM4_ESM.zip › 2.OTUs/OTU_level_plot/levelbar.pdf]

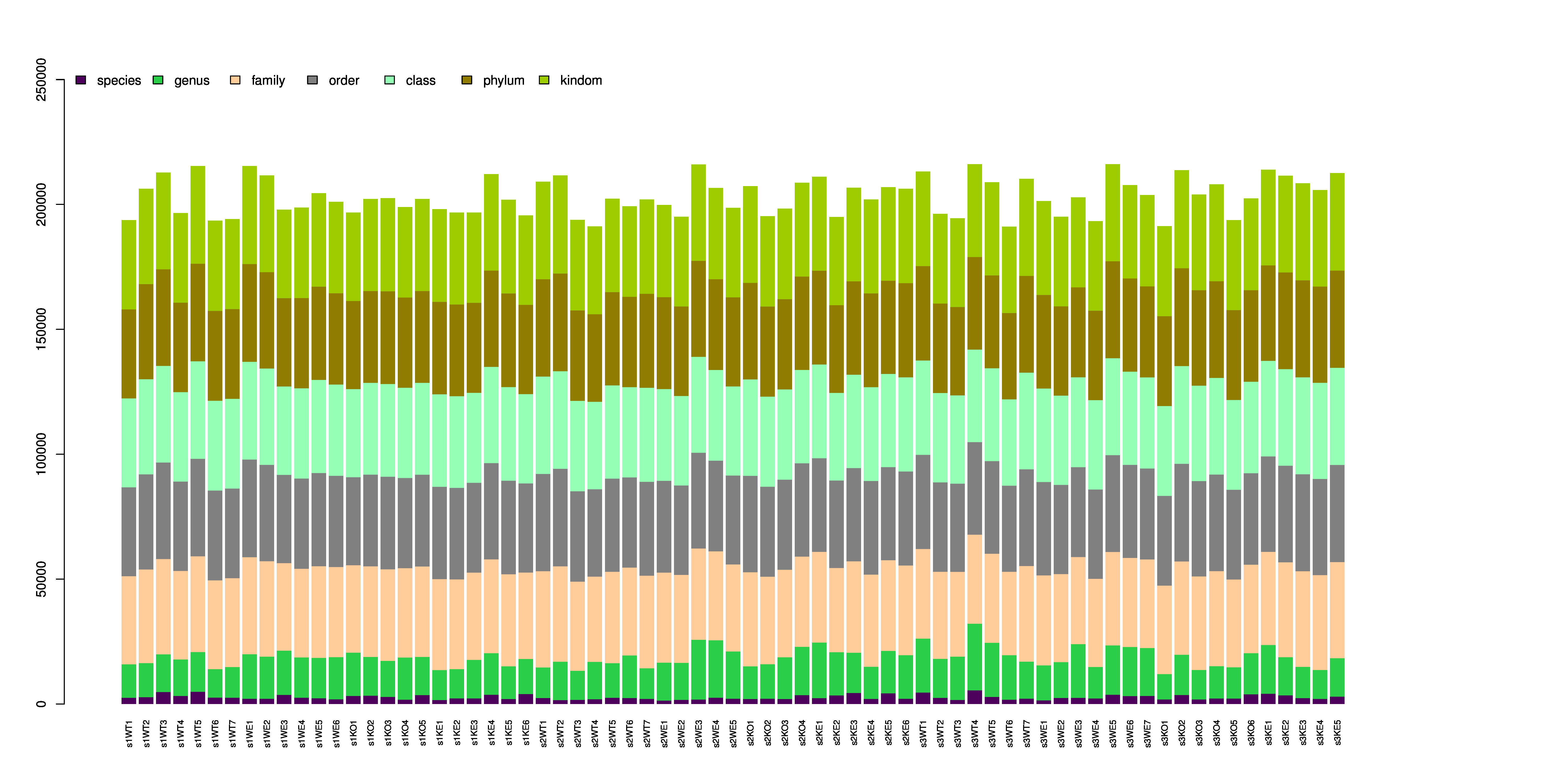

Supplement: Supplementary file 4 — Supplementary Data 1 [file 42003_2023_5520_MOESM4_ESM.zip › 2.OTUs/OTU_level_plot/levelbar.png]

kruskal-wallis  $p=1.42\text{e-}02$

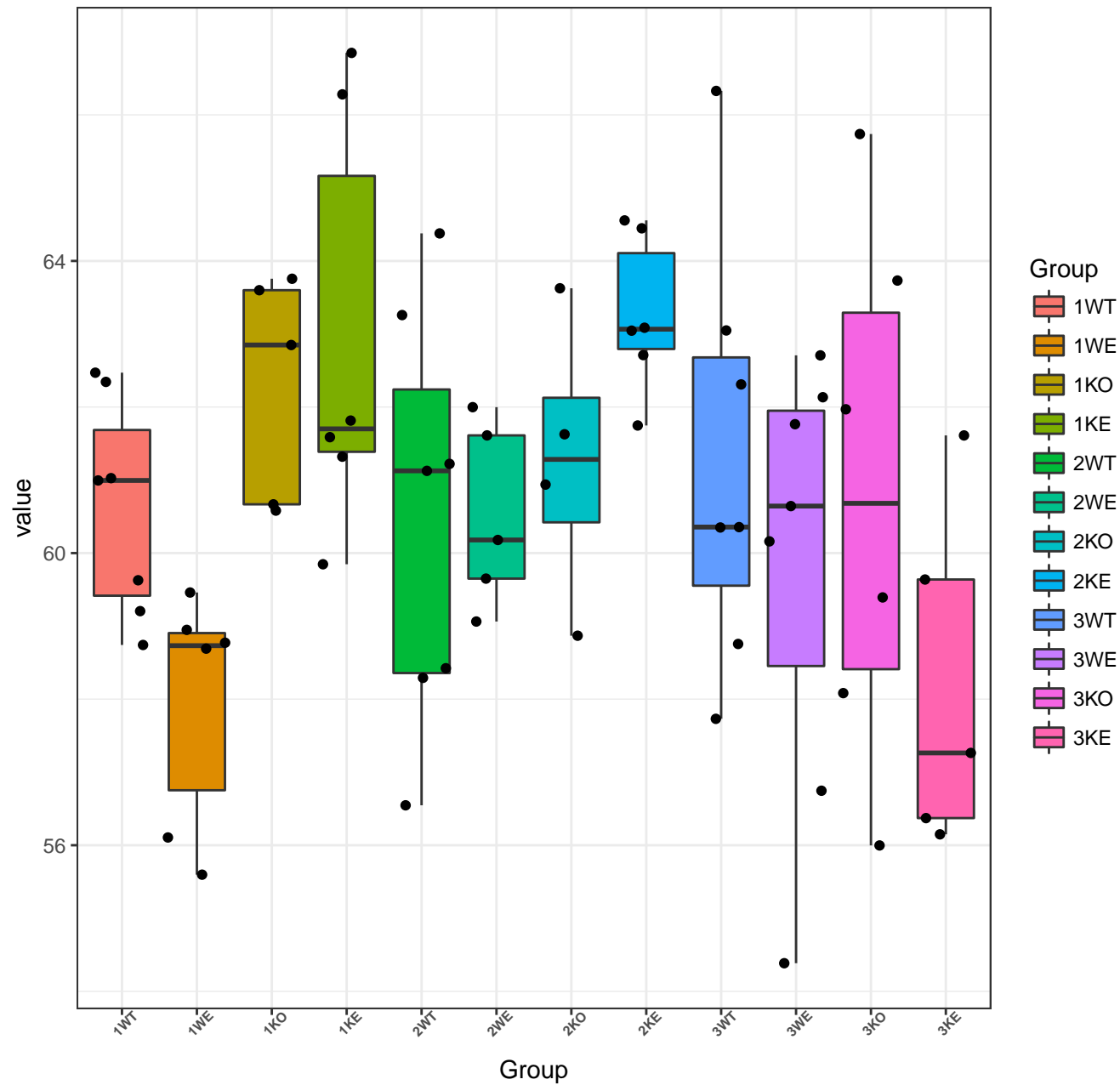

Supplement: Supplementary file 4 — Supplementary Data 1 [file 42003_2023_5520_MOESM4_ESM.zip › 4.Alpha_Diversity/alpha_boxplot/PD_whole_tree_boxplot.pdf]

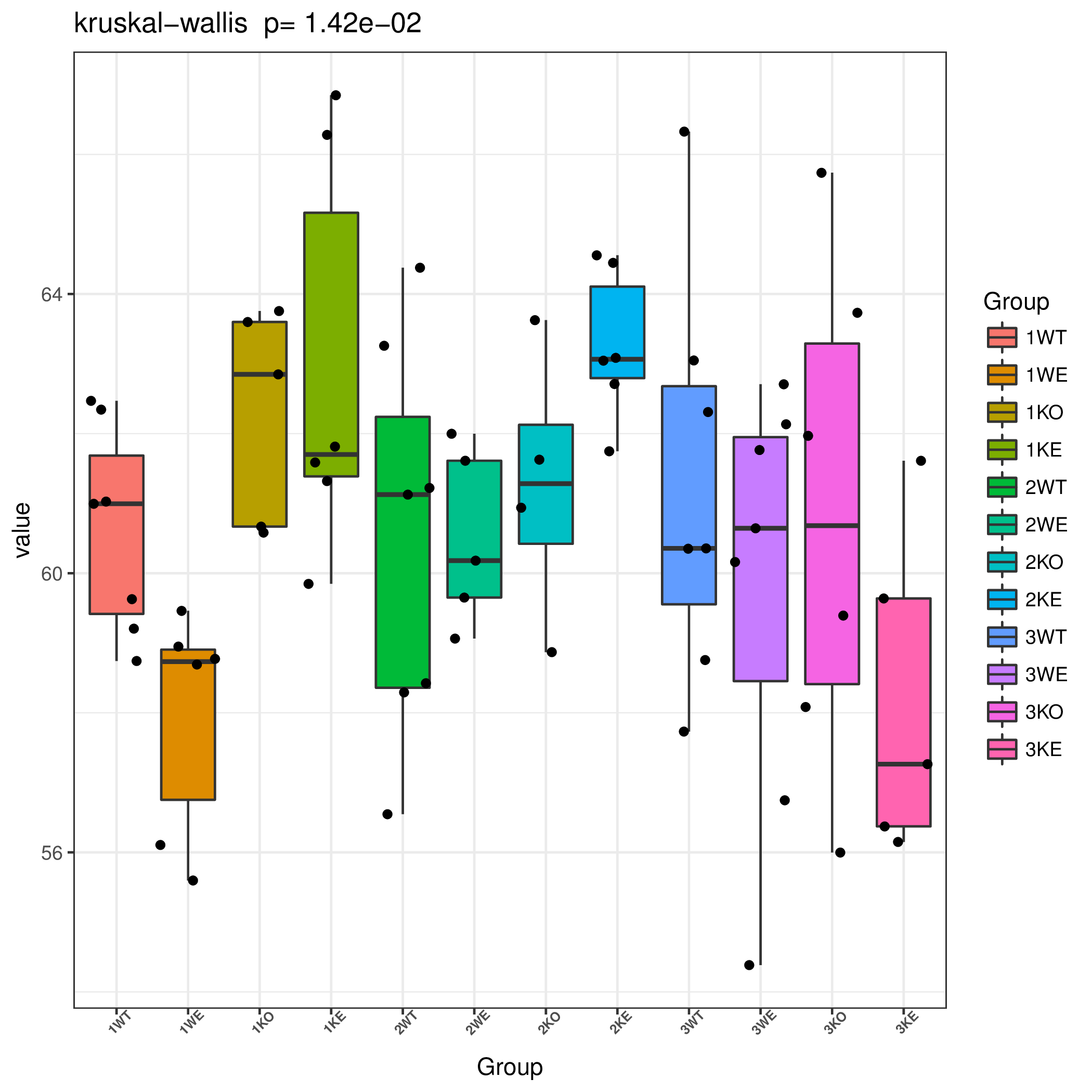

Supplement: Supplementary file 4 — Supplementary Data 1 [file 42003_2023_5520_MOESM4_ESM.zip › 4.Alpha_Diversity/alpha_boxplot/PD_whole_tree_boxplot.png]

kruskal-wallis p= 3.27e-02

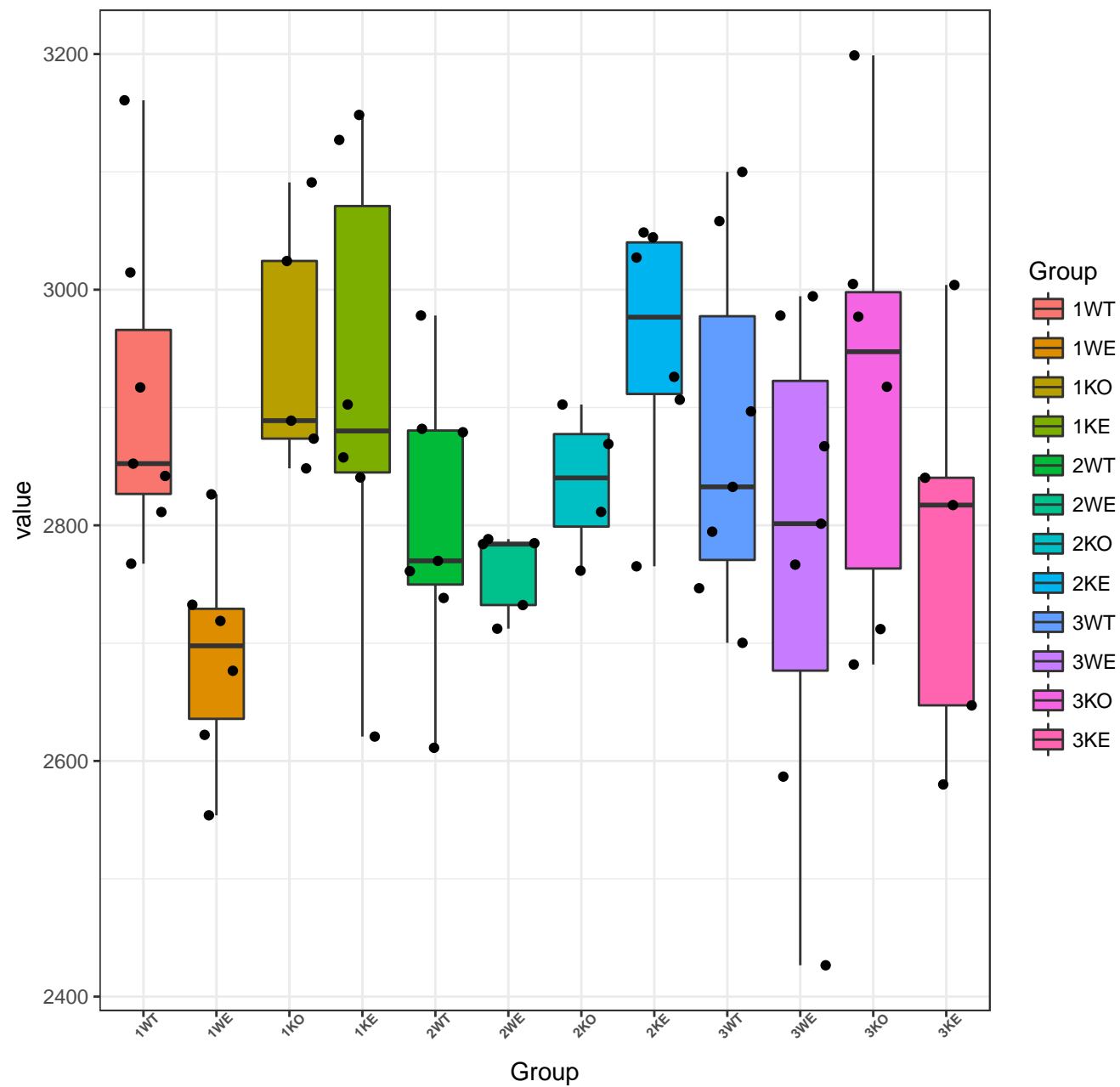

Supplement: Supplementary file 4 — Supplementary Data 1 [file 42003_2023_5520_MOESM4_ESM.zip › 4.Alpha_Diversity/alpha_boxplot/chao1_boxplot.pdf]

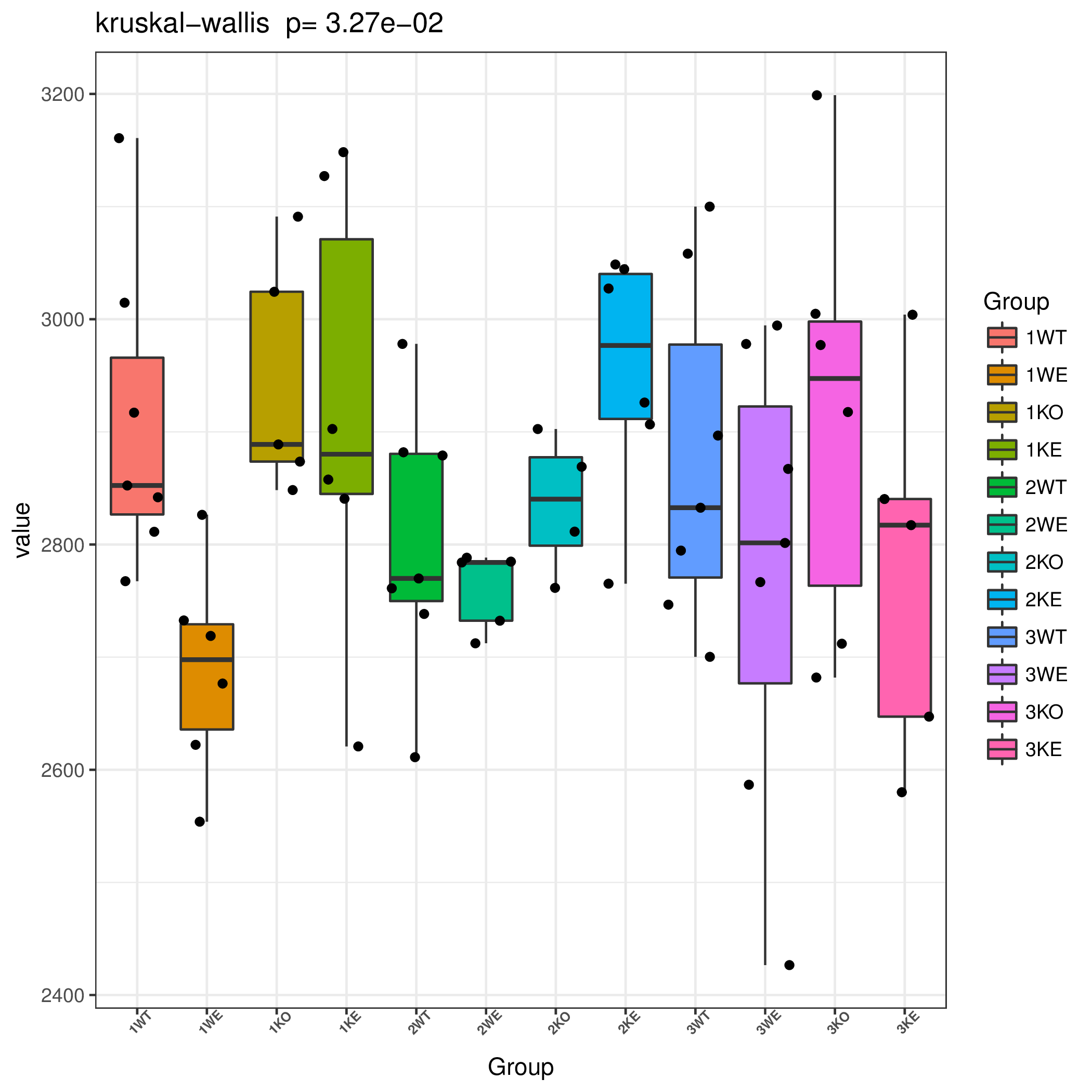

Supplement: Supplementary file 4 — Supplementary Data 1 [file 42003_2023_5520_MOESM4_ESM.zip › 4.Alpha_Diversity/alpha_boxplot/chao1_boxplot.png]

kruskal-wallis p= 5.17e-02

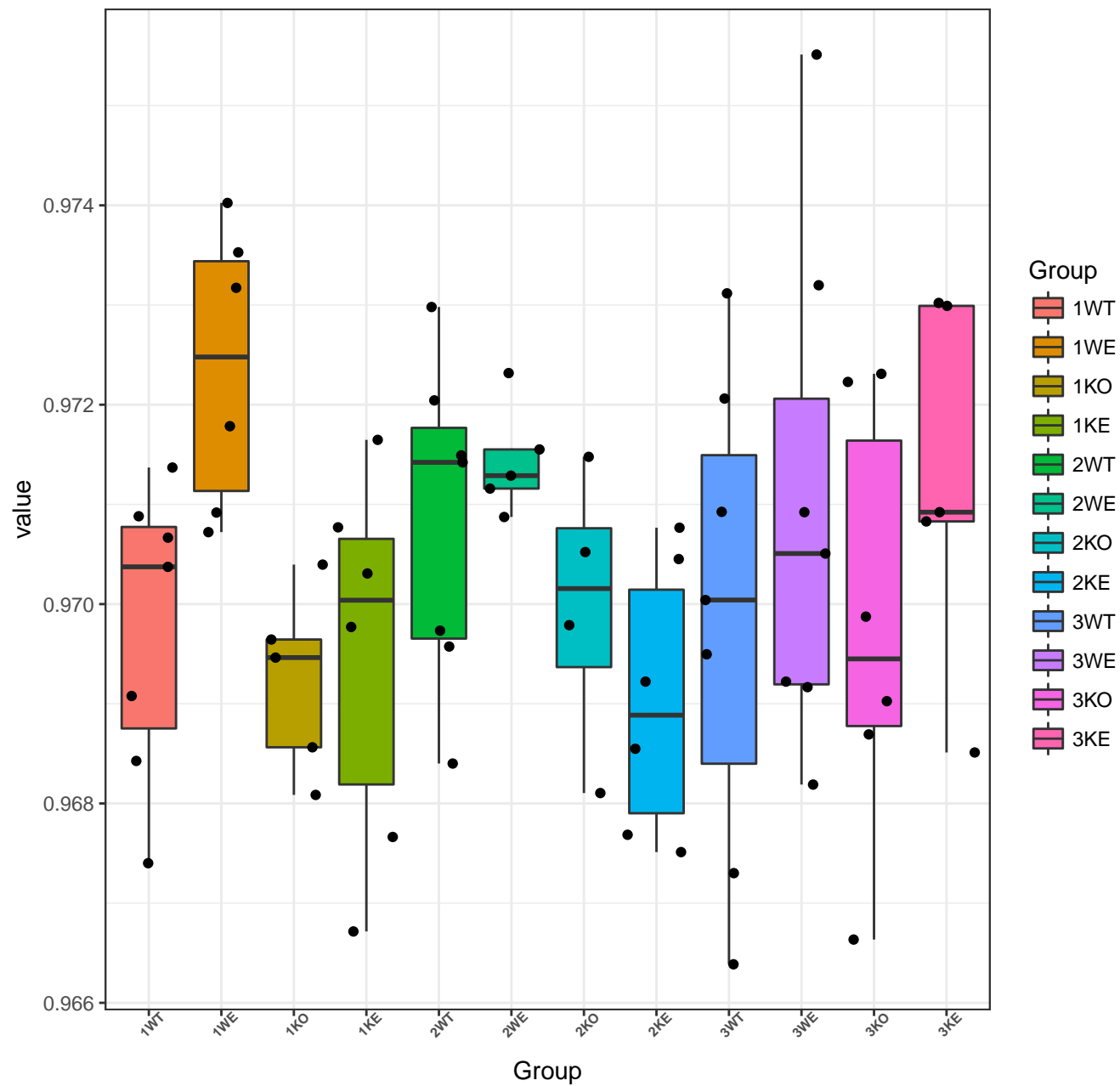

Supplement: Supplementary file 4 — Supplementary Data 1 [file 42003_2023_5520_MOESM4_ESM.zip › 4.Alpha_Diversity/alpha_boxplot/goods_coverage_boxplot.pdf]

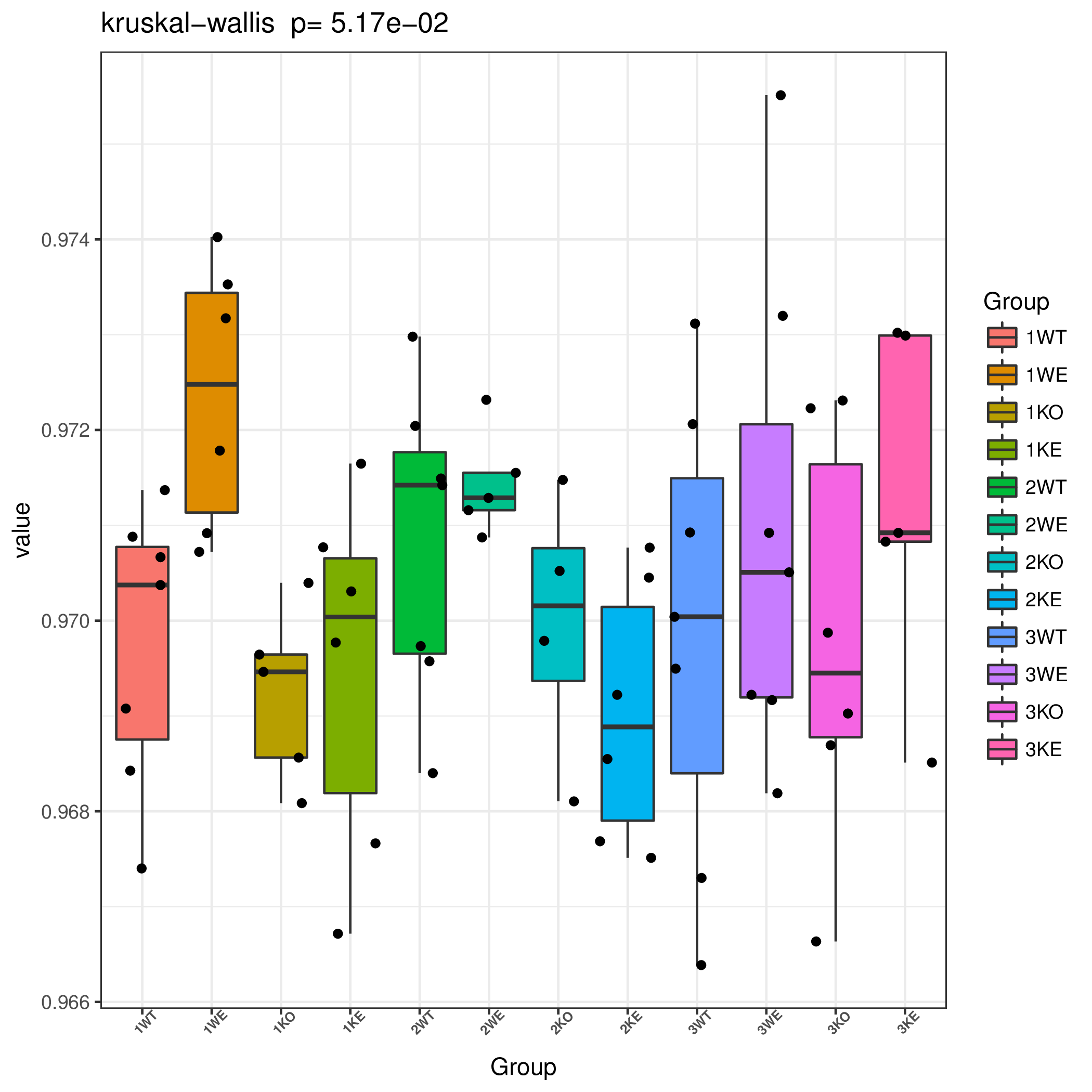

Supplement: Supplementary file 4 — Supplementary Data 1 [file 42003_2023_5520_MOESM4_ESM.zip › 4.Alpha_Diversity/alpha_boxplot/goods_coverage_boxplot.png]

kruskal-wallis p= 2.31e-02

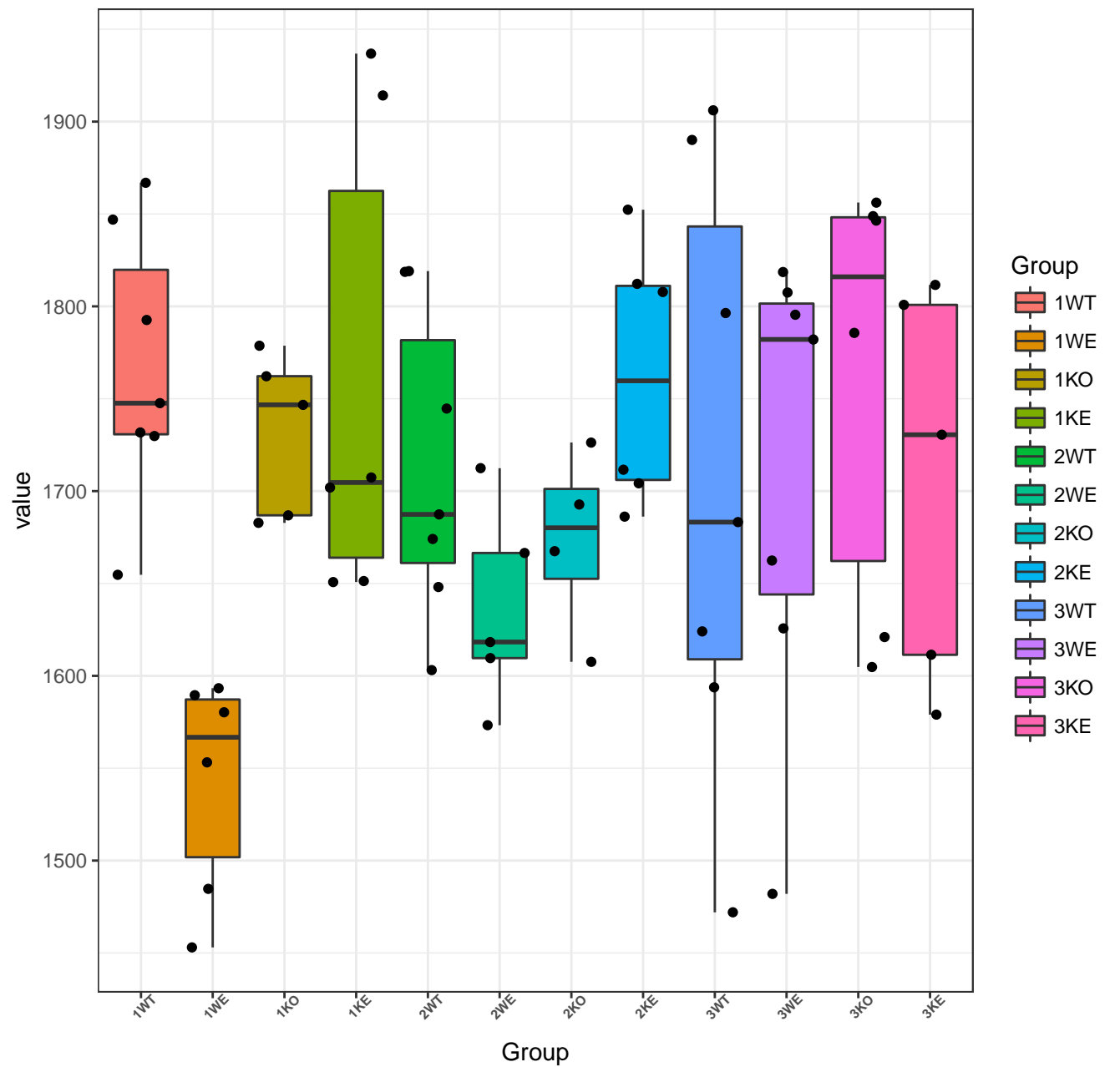

Supplement: Supplementary file 4 — Supplementary Data 1 [file 42003_2023_5520_MOESM4_ESM.zip › 4.Alpha_Diversity/alpha_boxplot/observed_species_boxplot.pdf]

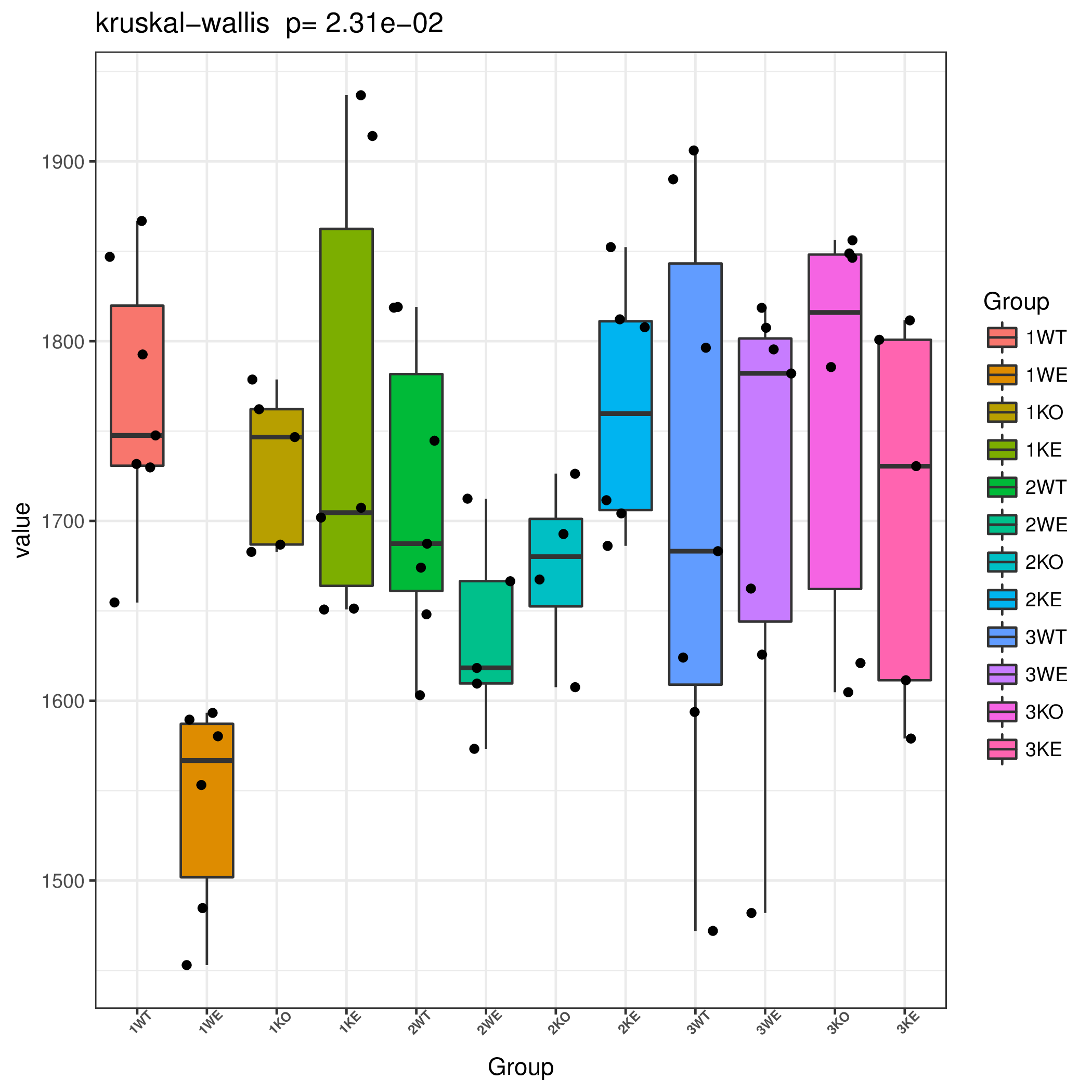

Supplement: Supplementary file 4 — Supplementary Data 1 [file 42003_2023_5520_MOESM4_ESM.zip › 4.Alpha_Diversity/alpha_boxplot/observed_species_boxplot.png]

kruskal-wallis p= 4.18e-02

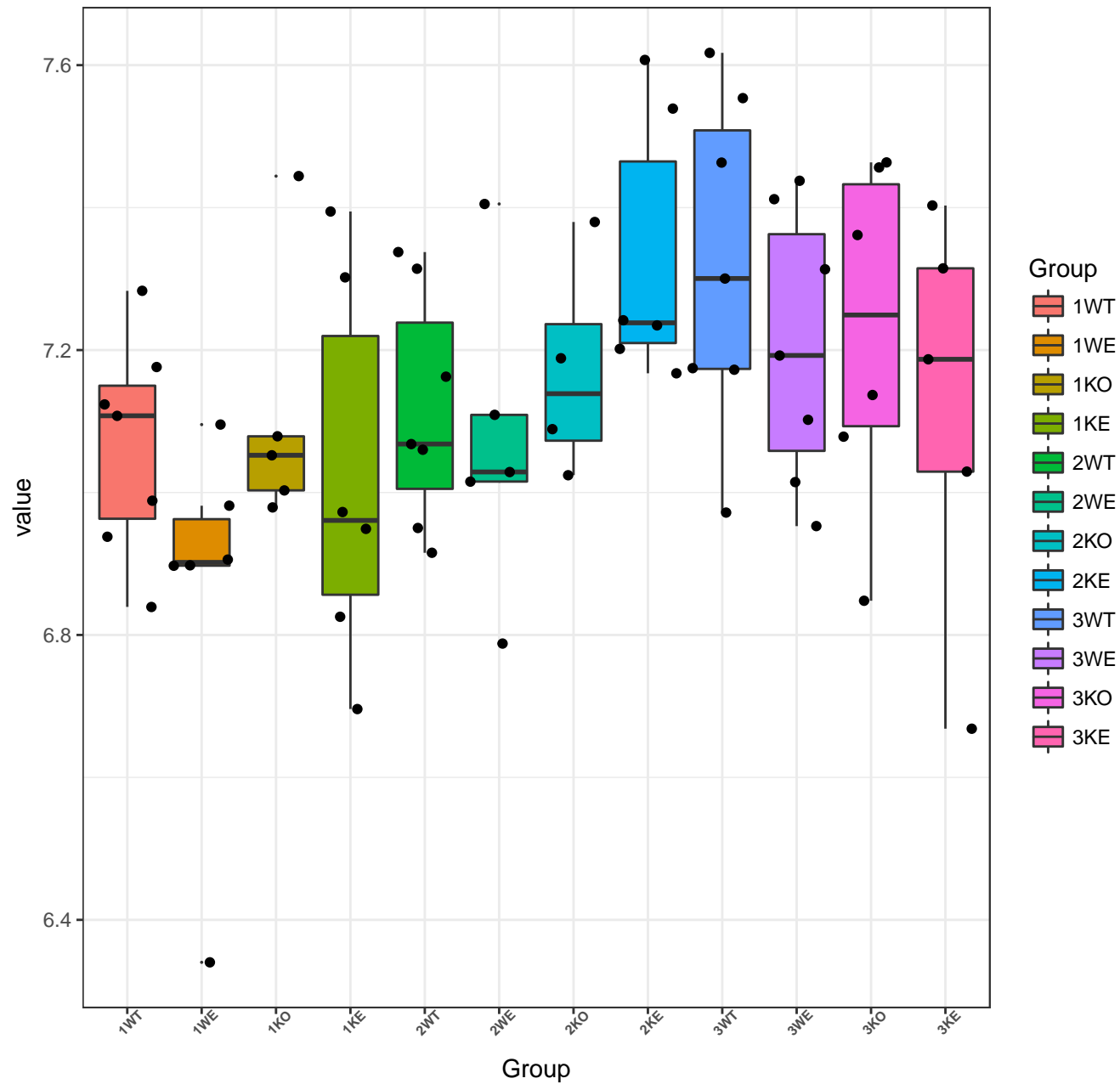

Supplement: Supplementary file 4 — Supplementary Data 1 [file 42003_2023_5520_MOESM4_ESM.zip › 4.Alpha_Diversity/alpha_boxplot/shannon_boxplot.pdf]

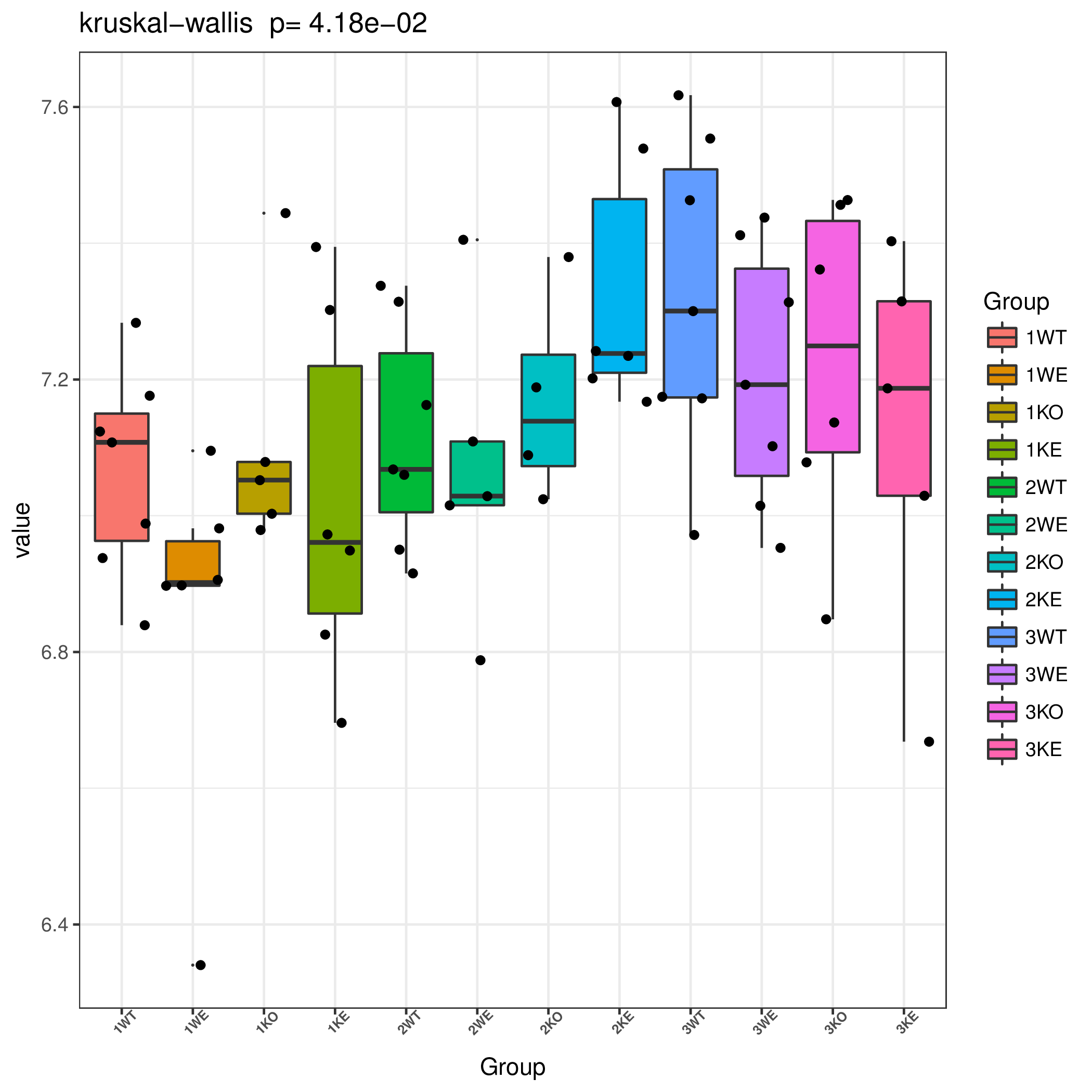

Supplement: Supplementary file 4 — Supplementary Data 1 [file 42003_2023_5520_MOESM4_ESM.zip › 4.Alpha_Diversity/alpha_boxplot/shannon_boxplot.png]

kruskal-wallis p= 4.47e-03

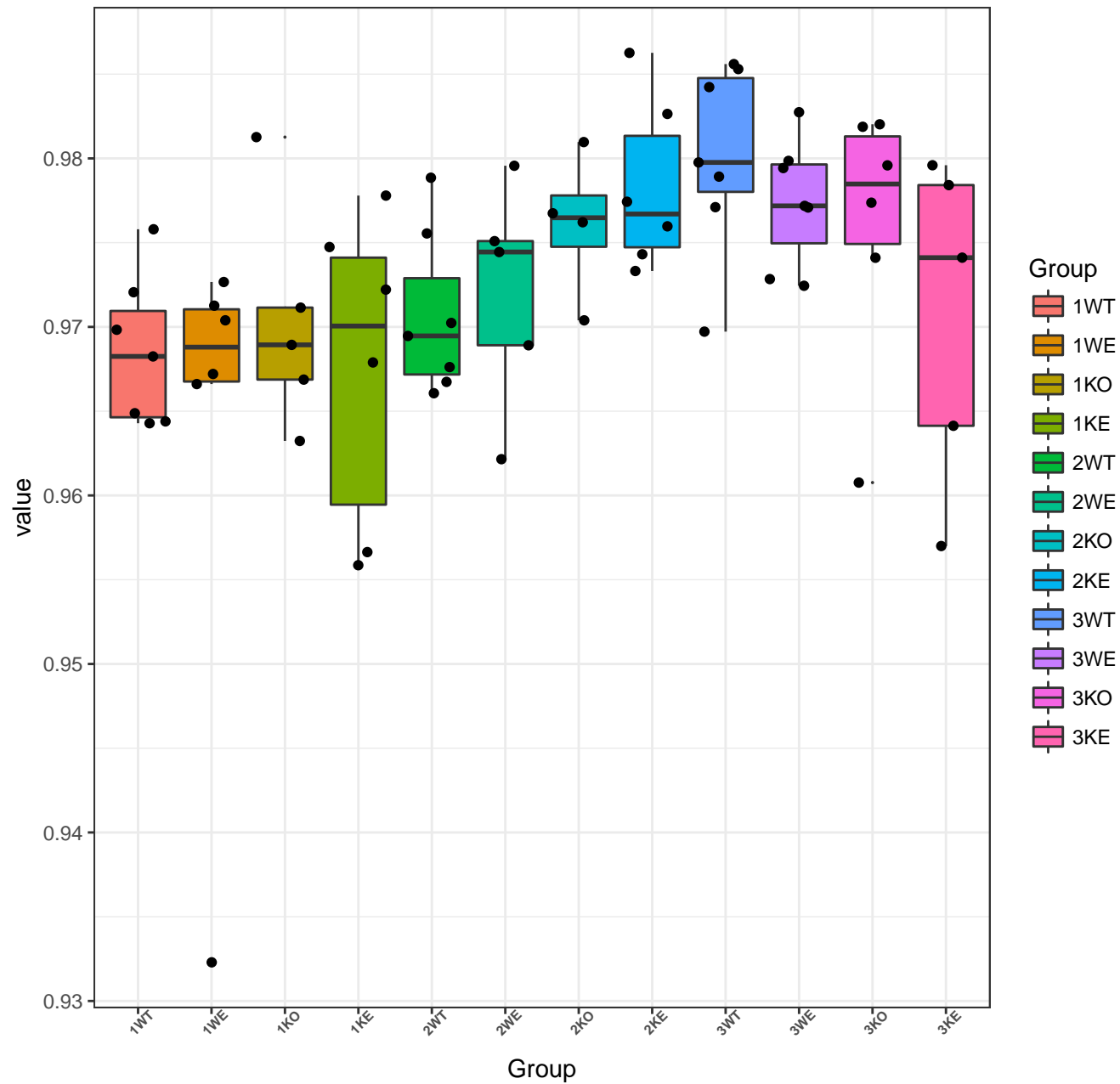

Supplement: Supplementary file 4 — Supplementary Data 1 [file 42003_2023_5520_MOESM4_ESM.zip › 4.Alpha_Diversity/alpha_boxplot/simpson_boxplot.pdf]

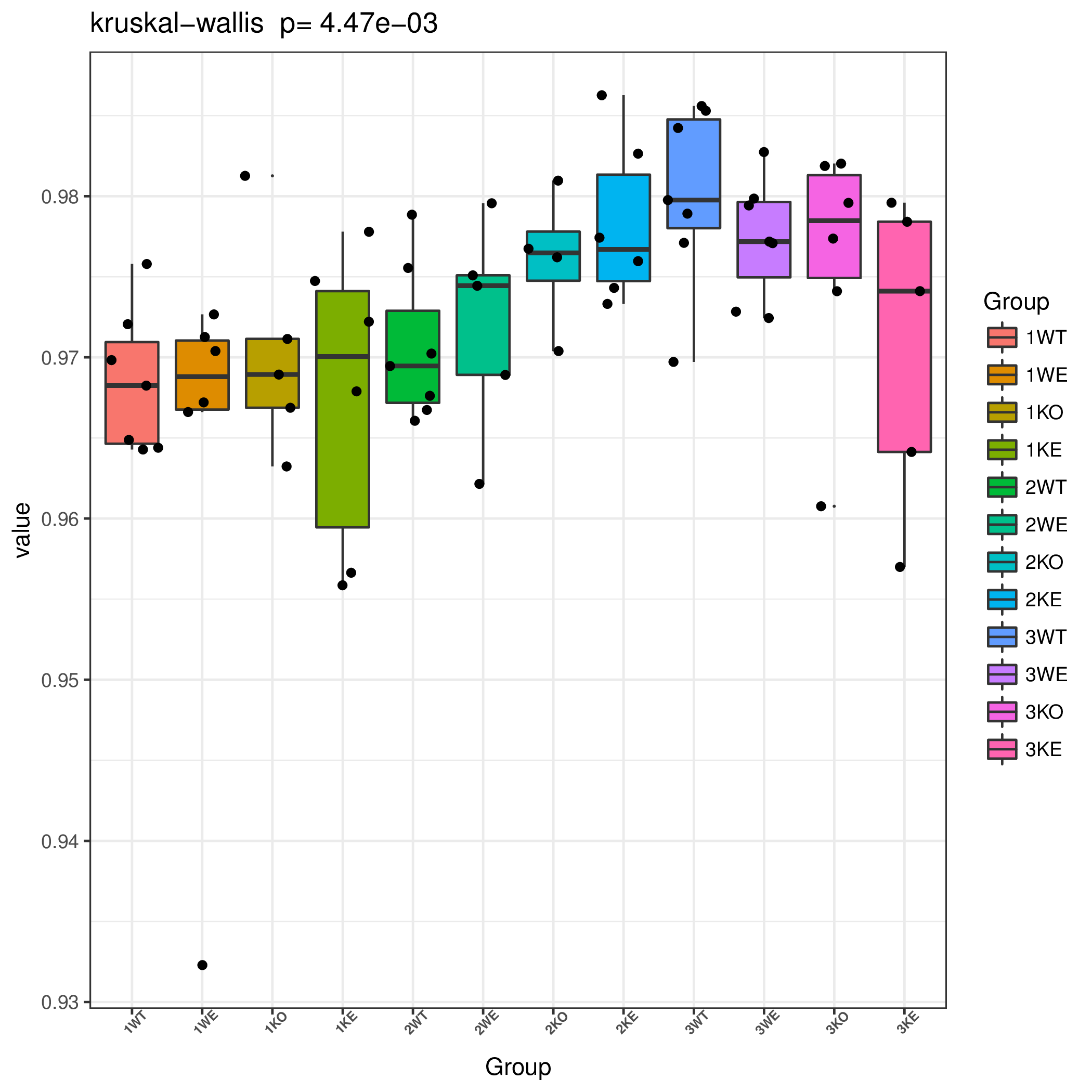

Supplement: Supplementary file 4 — Supplementary Data 1 [file 42003_2023_5520_MOESM4_ESM.zip › 4.Alpha_Diversity/alpha_boxplot/simpson_boxplot.png]

PD\_whole\_tree

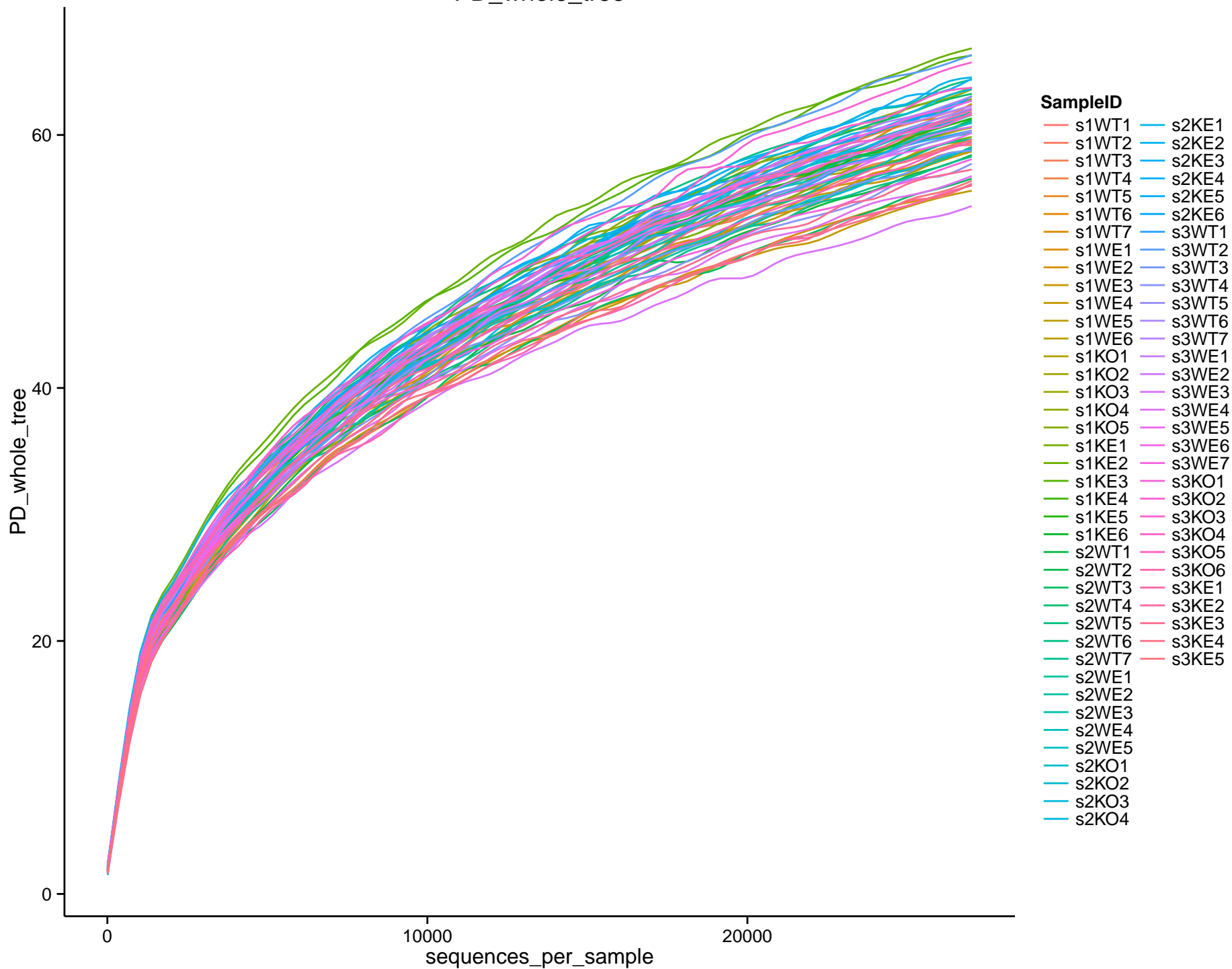

Supplement: Supplementary file 4 — Supplementary Data 1 [file 42003_2023_5520_MOESM4_ESM.zip › 4.Alpha_Diversity/alpha_rarefaction_plot/PD_whole_tree.pdf]

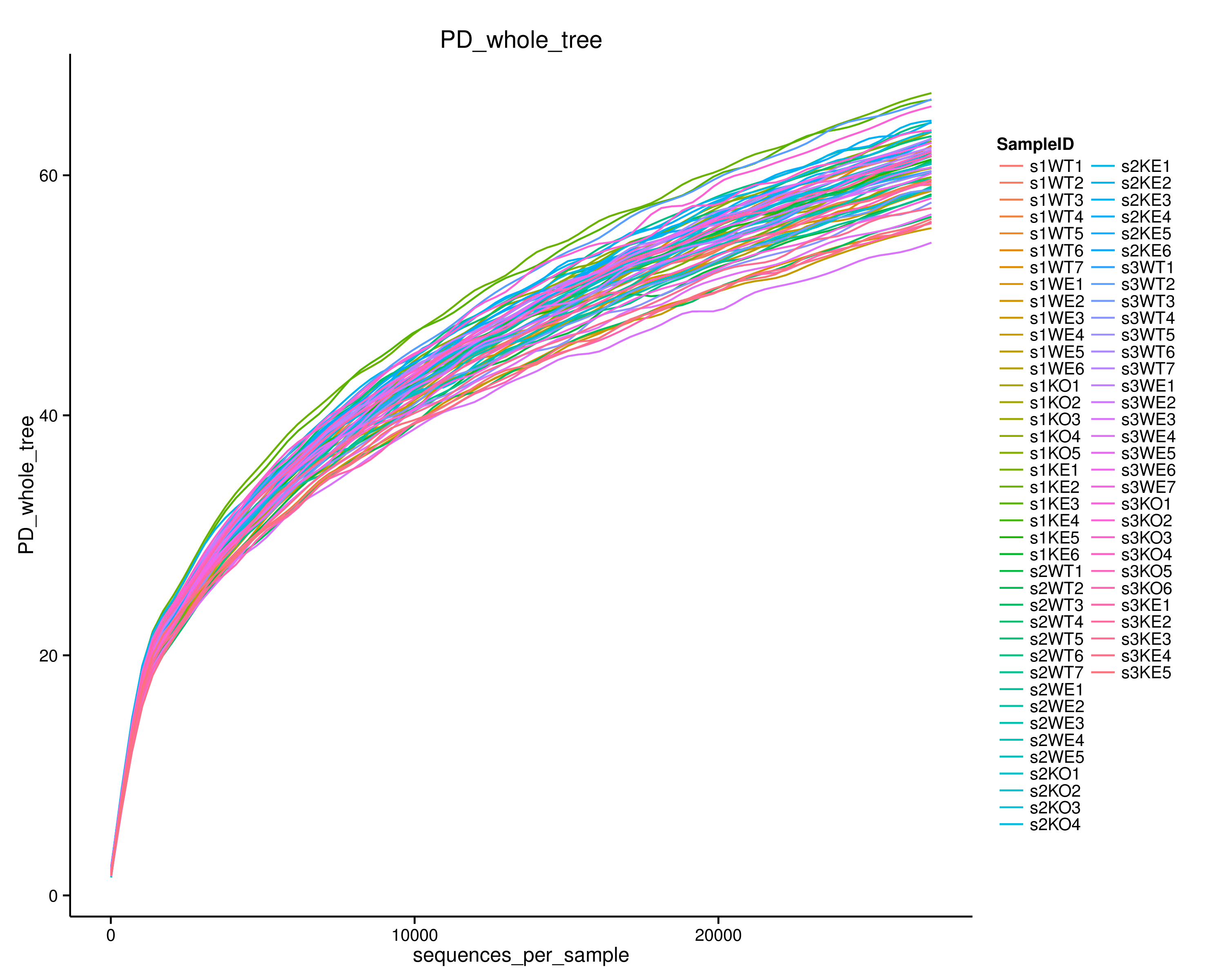

Supplement: Supplementary file 4 — Supplementary Data 1 [file 42003_2023_5520_MOESM4_ESM.zip › 4.Alpha_Diversity/alpha_rarefaction_plot/PD_whole_tree.png]

chao1

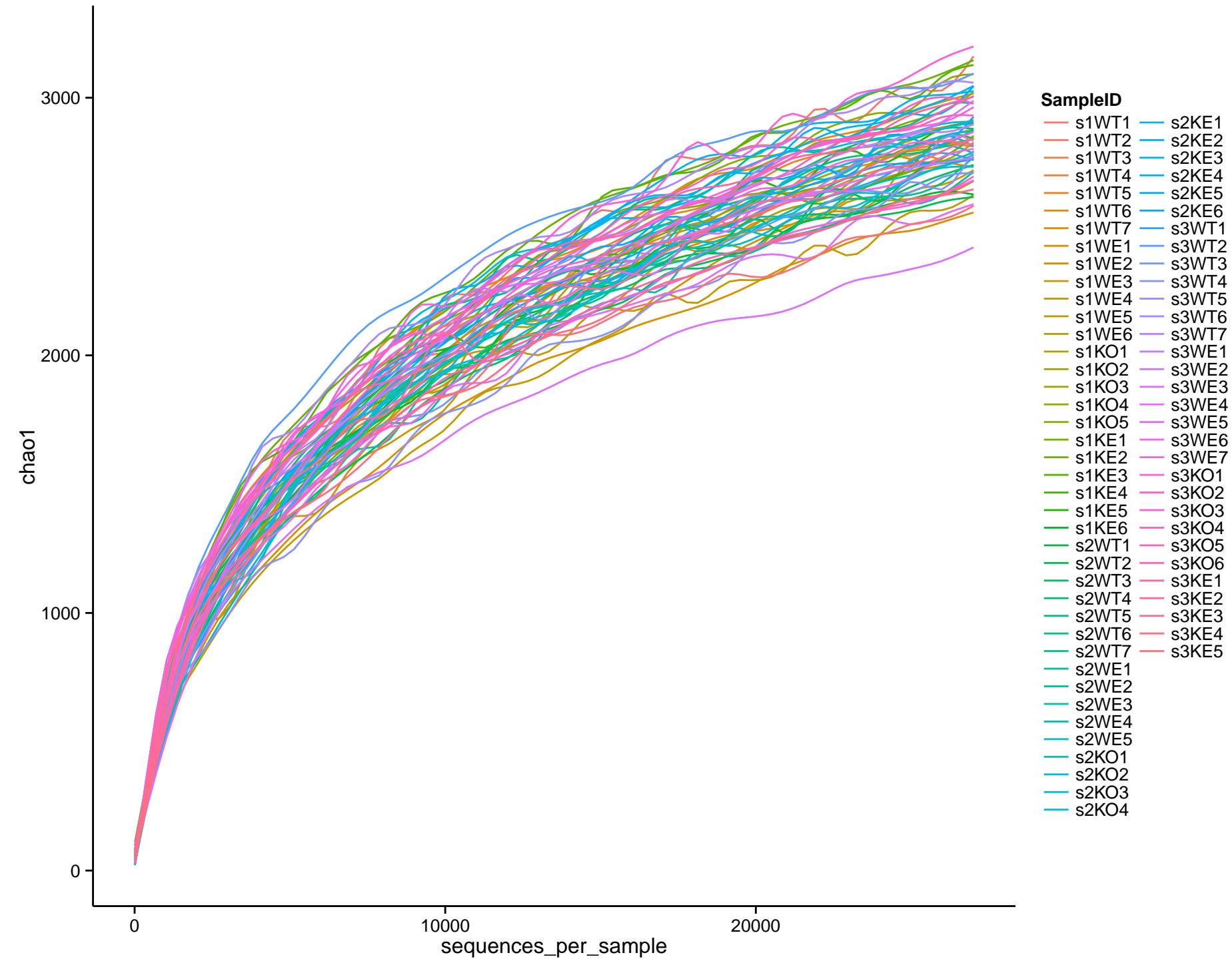

Supplement: Supplementary file 4 — Supplementary Data 1 [file 42003_2023_5520_MOESM4_ESM.zip › 4.Alpha_Diversity/alpha_rarefaction_plot/chao1.pdf]

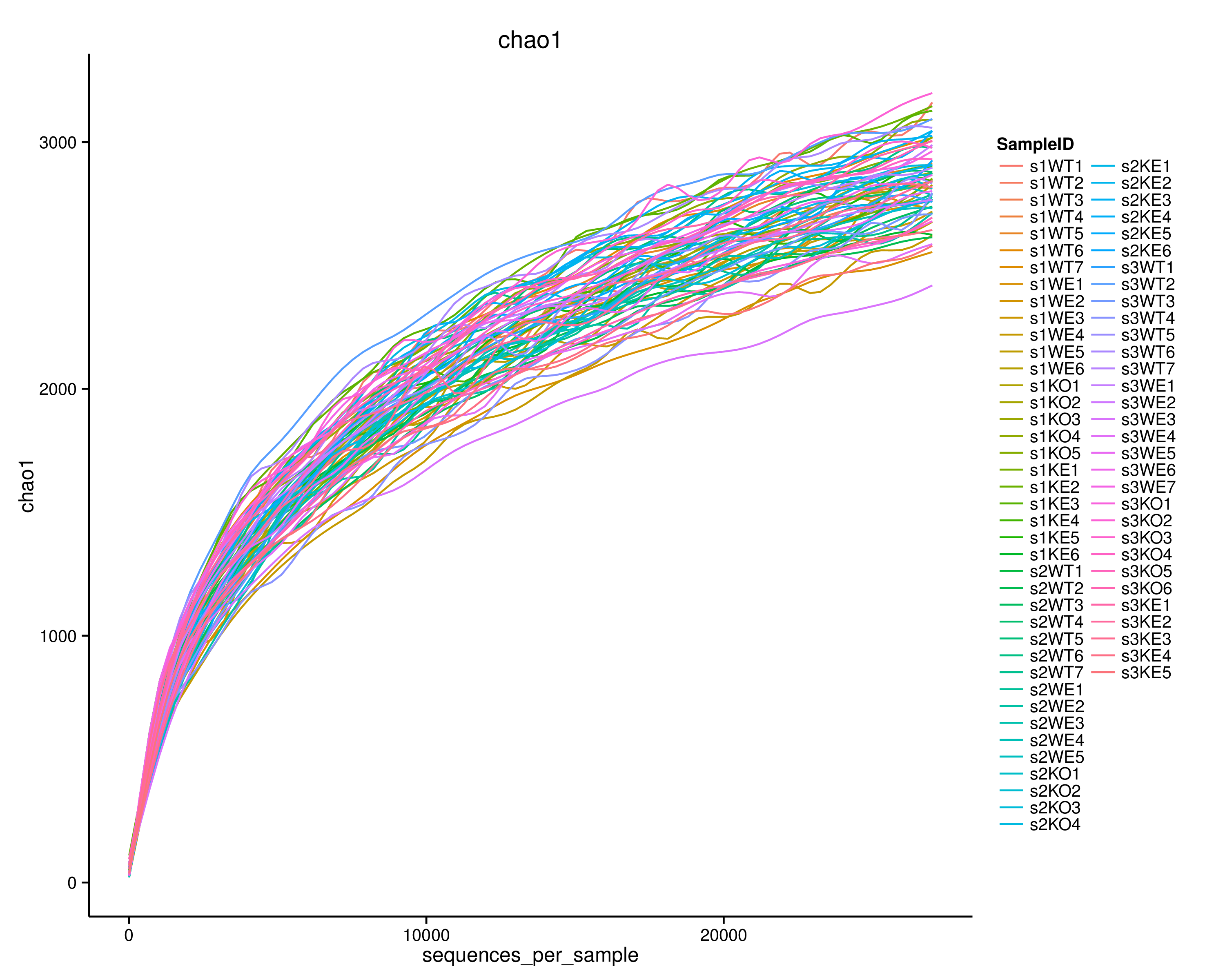

Supplement: Supplementary file 4 — Supplementary Data 1 [file 42003_2023_5520_MOESM4_ESM.zip › 4.Alpha_Diversity/alpha_rarefaction_plot/chao1.png]

goods\_coverage

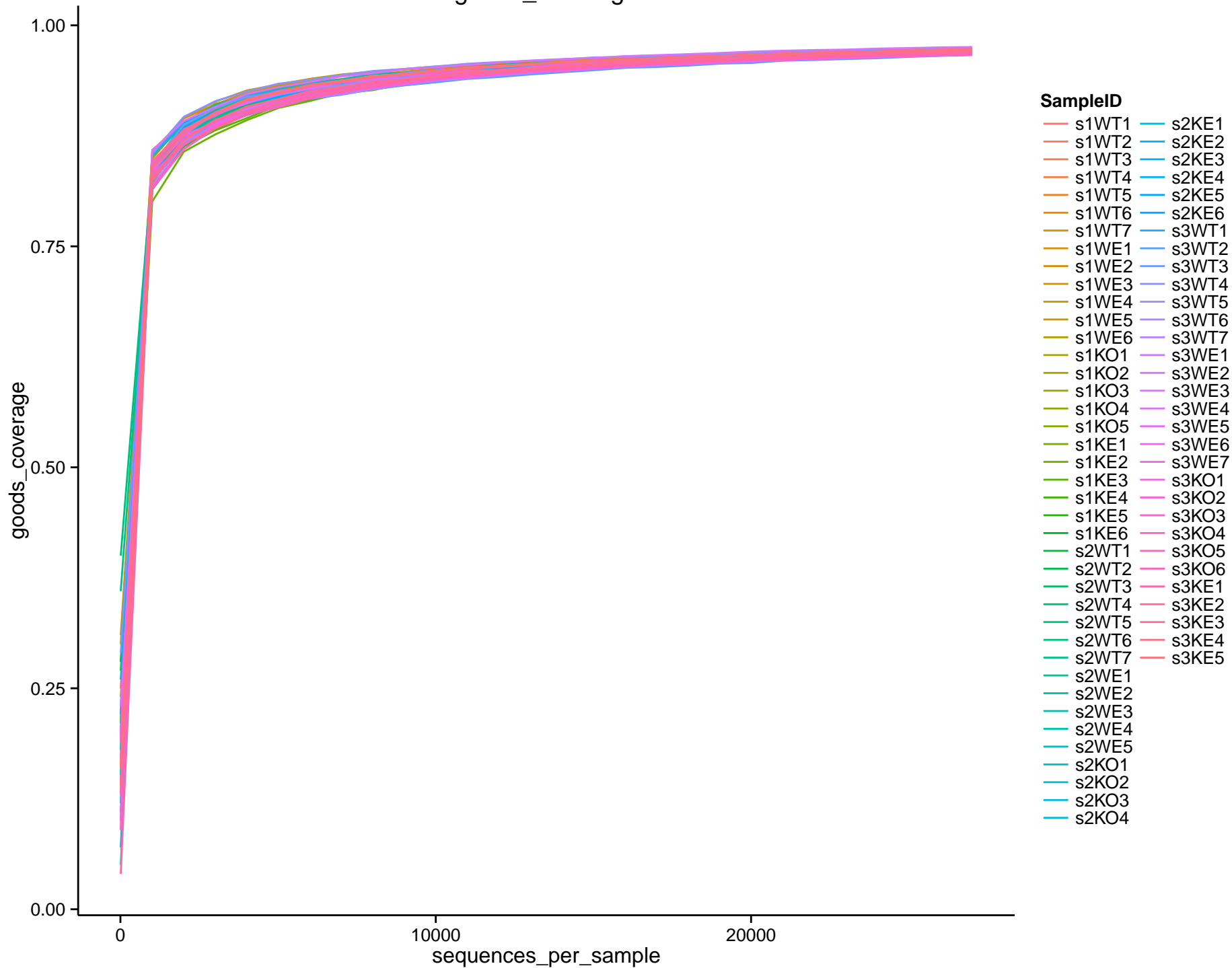

Supplement: Supplementary file 4 — Supplementary Data 1 [file 42003_2023_5520_MOESM4_ESM.zip › 4.Alpha_Diversity/alpha_rarefaction_plot/goods_coverage.pdf]

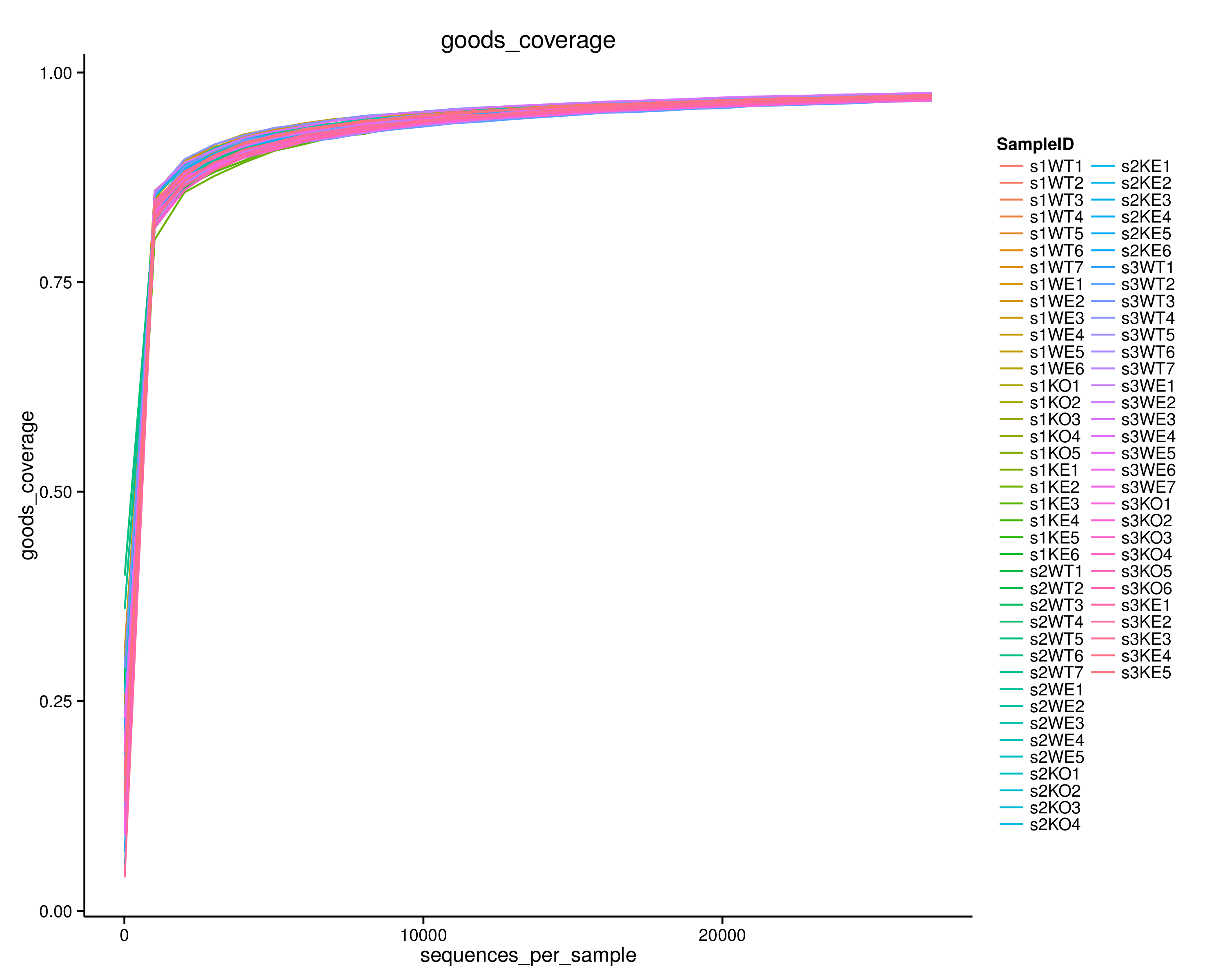

Supplement: Supplementary file 4 — Supplementary Data 1 [file 42003_2023_5520_MOESM4_ESM.zip › 4.Alpha_Diversity/alpha_rarefaction_plot/goods_coverage.png]

observed\_species

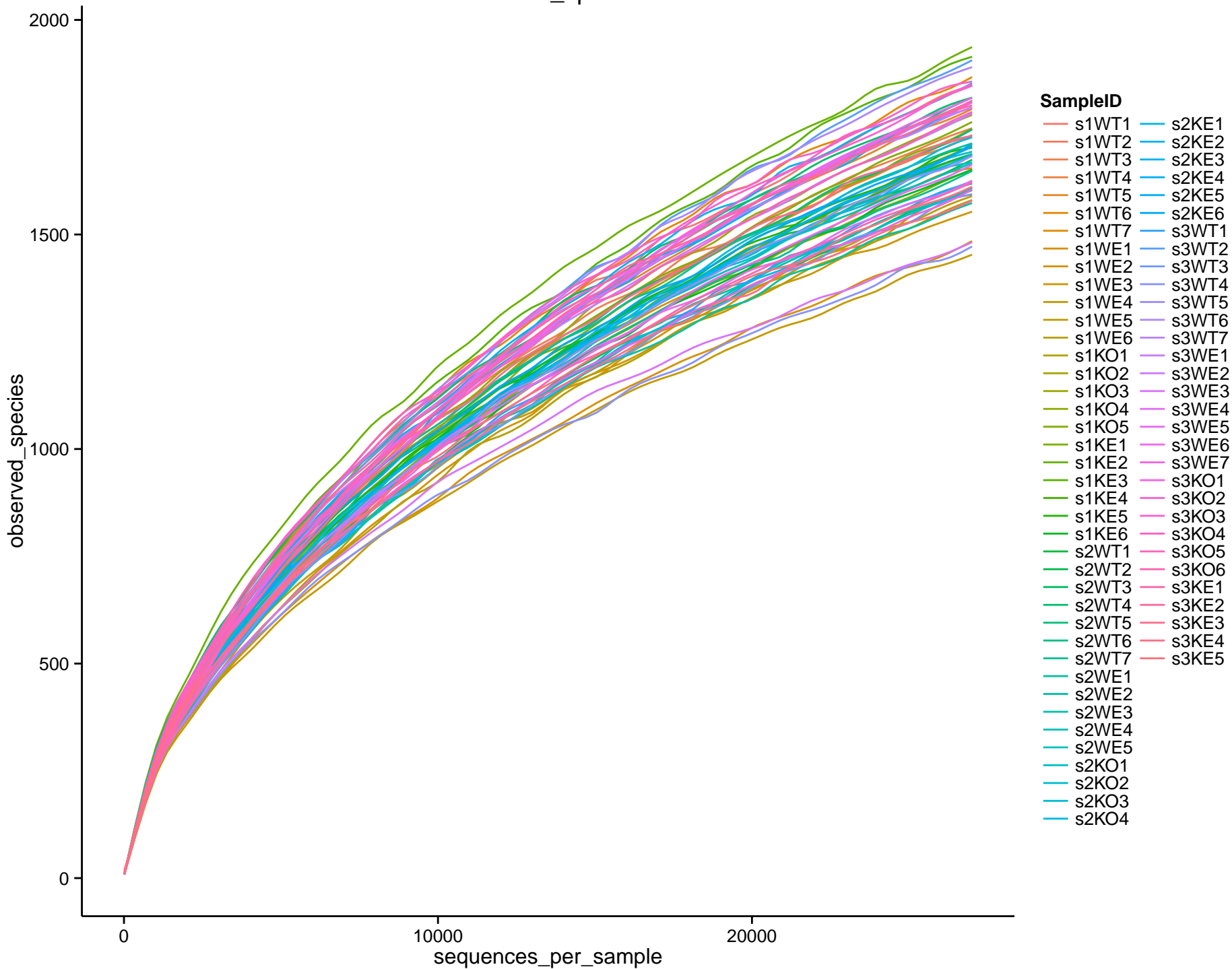

Supplement: Supplementary file 4 — Supplementary Data 1 [file 42003_2023_5520_MOESM4_ESM.zip › 4.Alpha_Diversity/alpha_rarefaction_plot/observed_species.pdf]

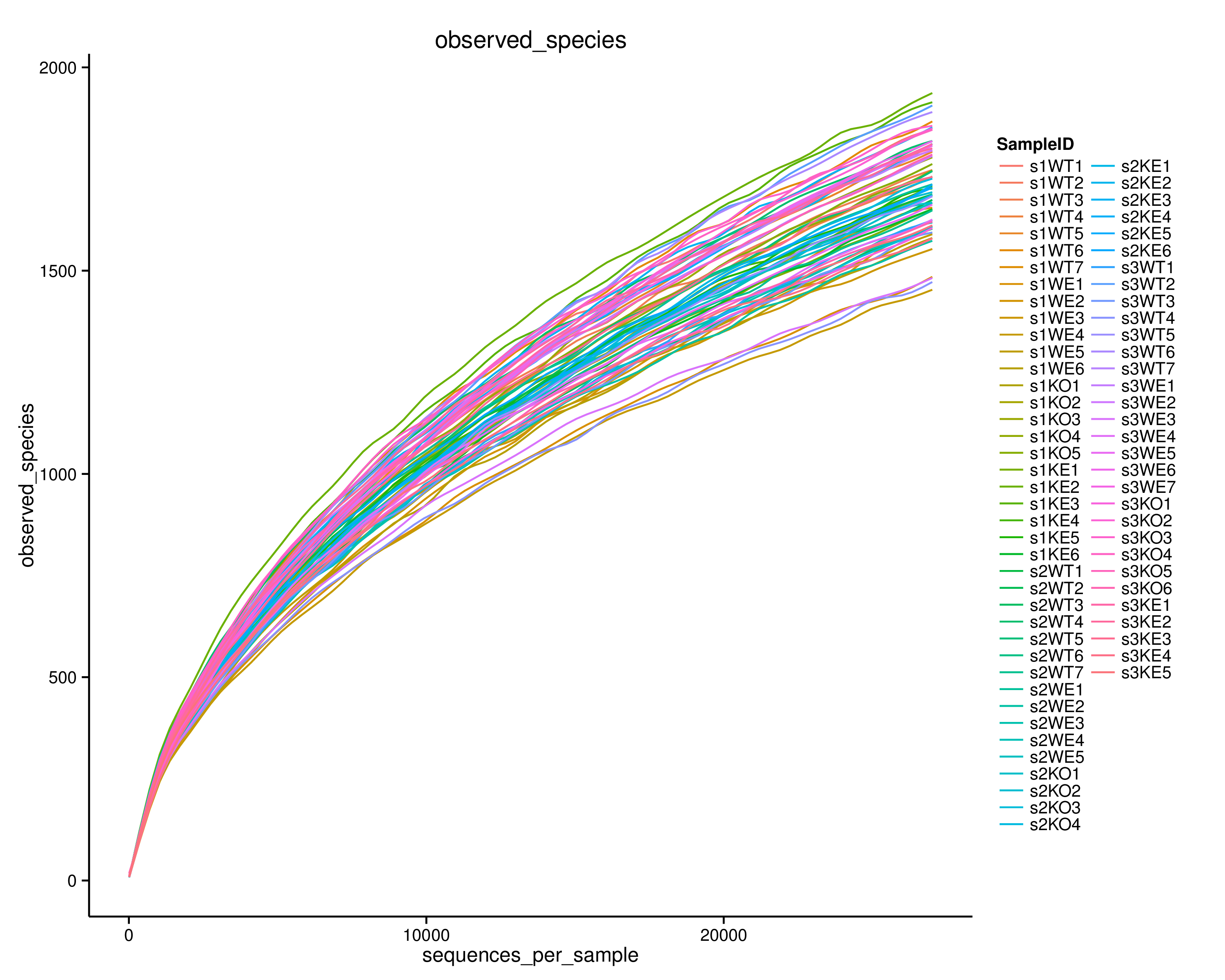

Supplement: Supplementary file 4 — Supplementary Data 1 [file 42003_2023_5520_MOESM4_ESM.zip › 4.Alpha_Diversity/alpha_rarefaction_plot/observed_species.png]

PD\_whole\_tree: BarcodeSequence

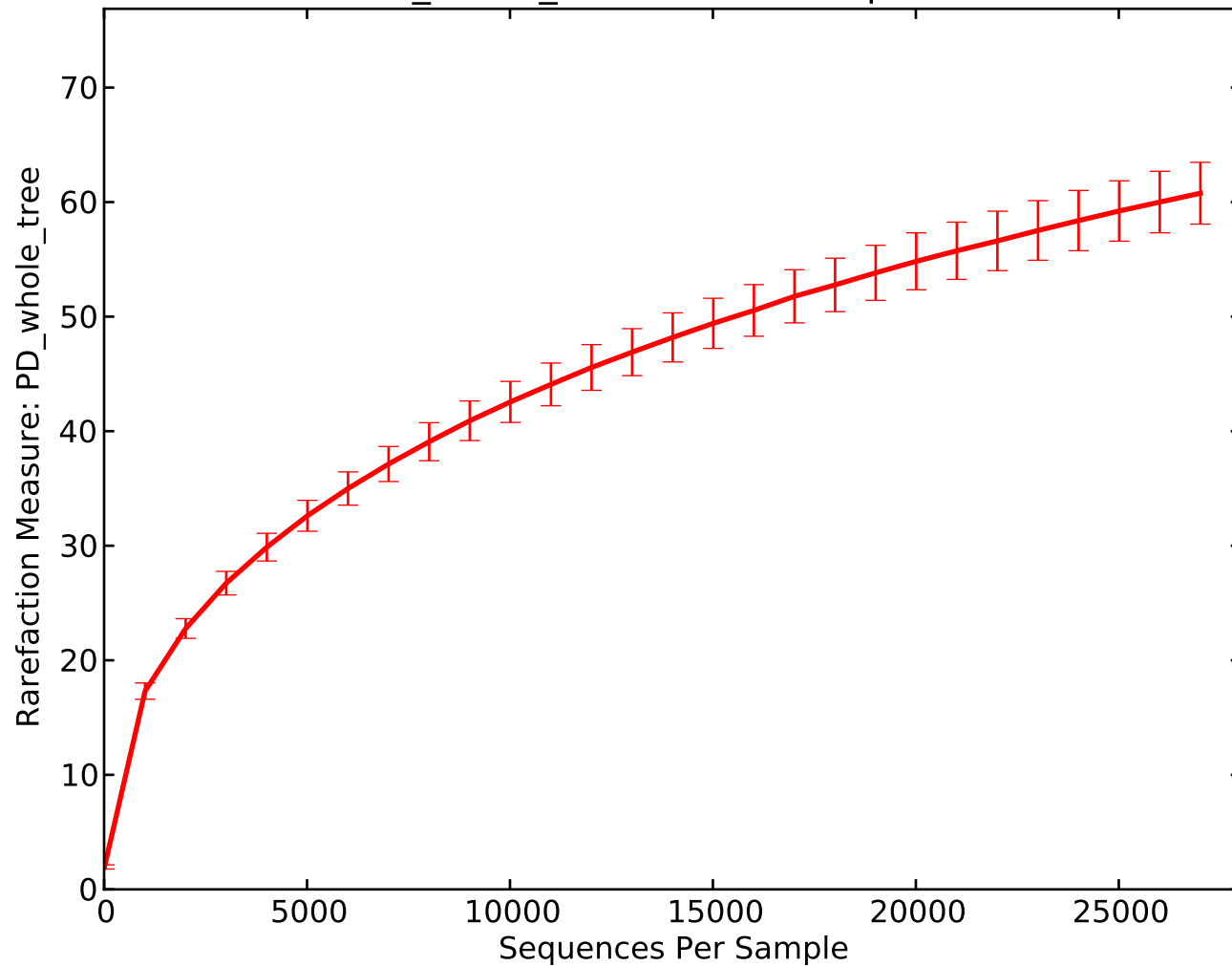

Supplement: Supplementary file 4 — Supplementary Data 1 [file 42003_2023_5520_MOESM4_ESM.zip › 4.Alpha_Diversity/alpha_rarefaction_plot/rarefaction_plots_pdf_depth27686/average_plots/PD_whole_treeBarcodeSequence.pdf]

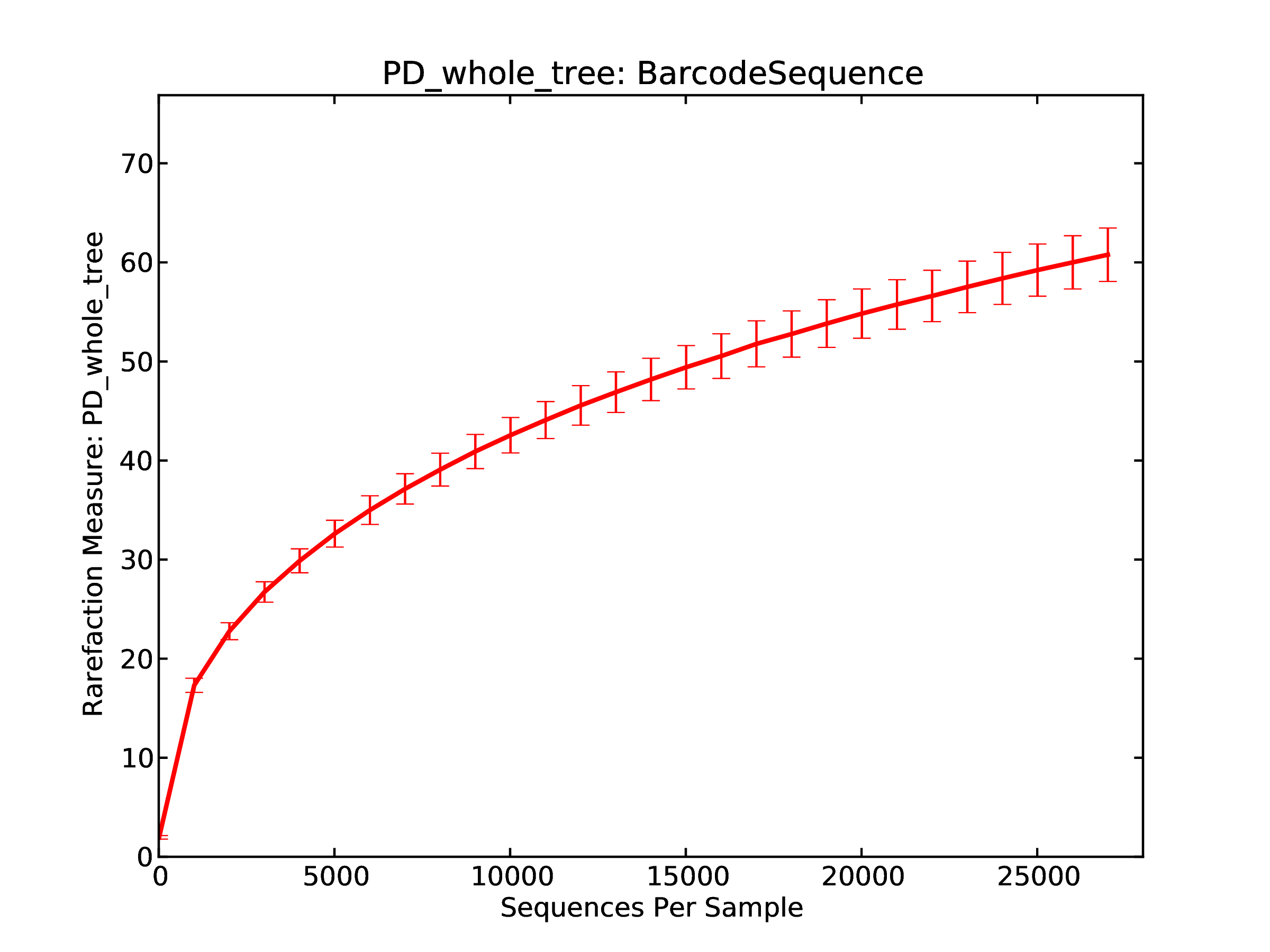

Supplement: Supplementary file 4 — Supplementary Data 1 [file 42003_2023_5520_MOESM4_ESM.zip › 4.Alpha_Diversity/alpha_rarefaction_plot/rarefaction_plots_pdf_depth27686/average_plots/PD_whole_treeBarcodeSequence.png]

PD\_whole\_tree: Description

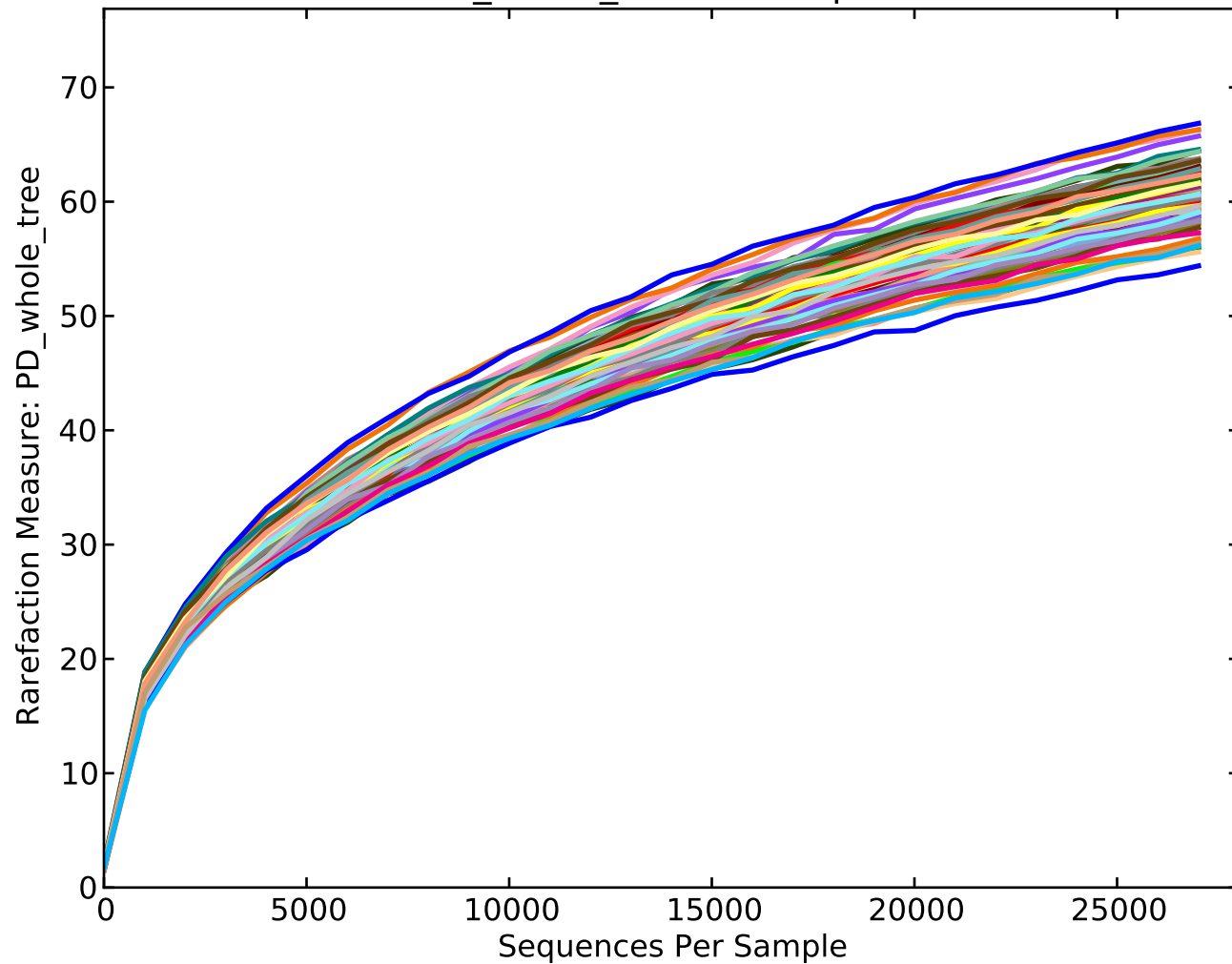

Supplement: Supplementary file 4 — Supplementary Data 1 [file 42003_2023_5520_MOESM4_ESM.zip › 4.Alpha_Diversity/alpha_rarefaction_plot/rarefaction_plots_pdf_depth27686/average_plots/PD_whole_treeDescription.pdf]

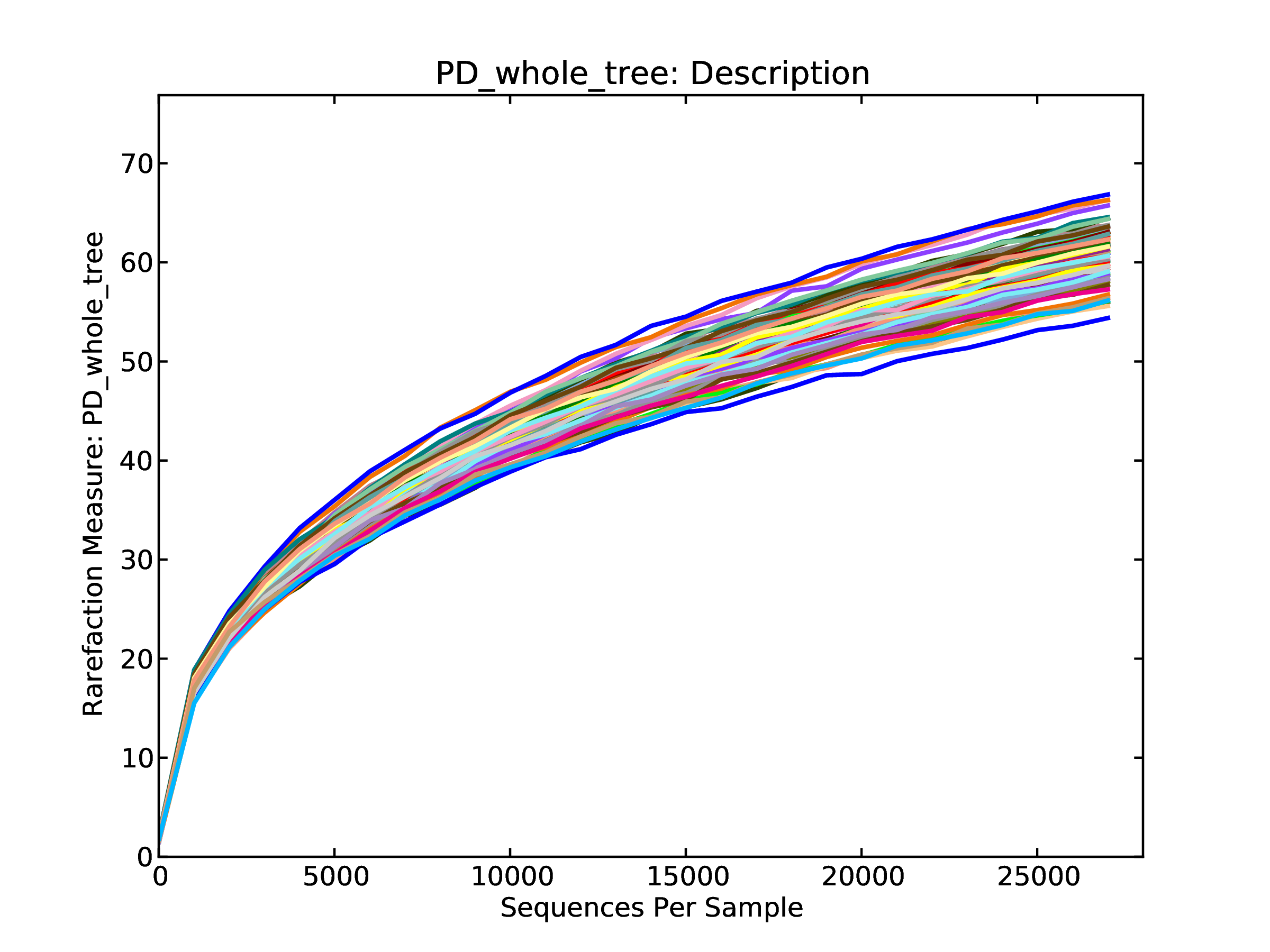

Supplement: Supplementary file 4 — Supplementary Data 1 [file 42003_2023_5520_MOESM4_ESM.zip › 4.Alpha_Diversity/alpha_rarefaction_plot/rarefaction_plots_pdf_depth27686/average_plots/PD_whole_treeDescription.png]

PD\_whole\_tree: Group

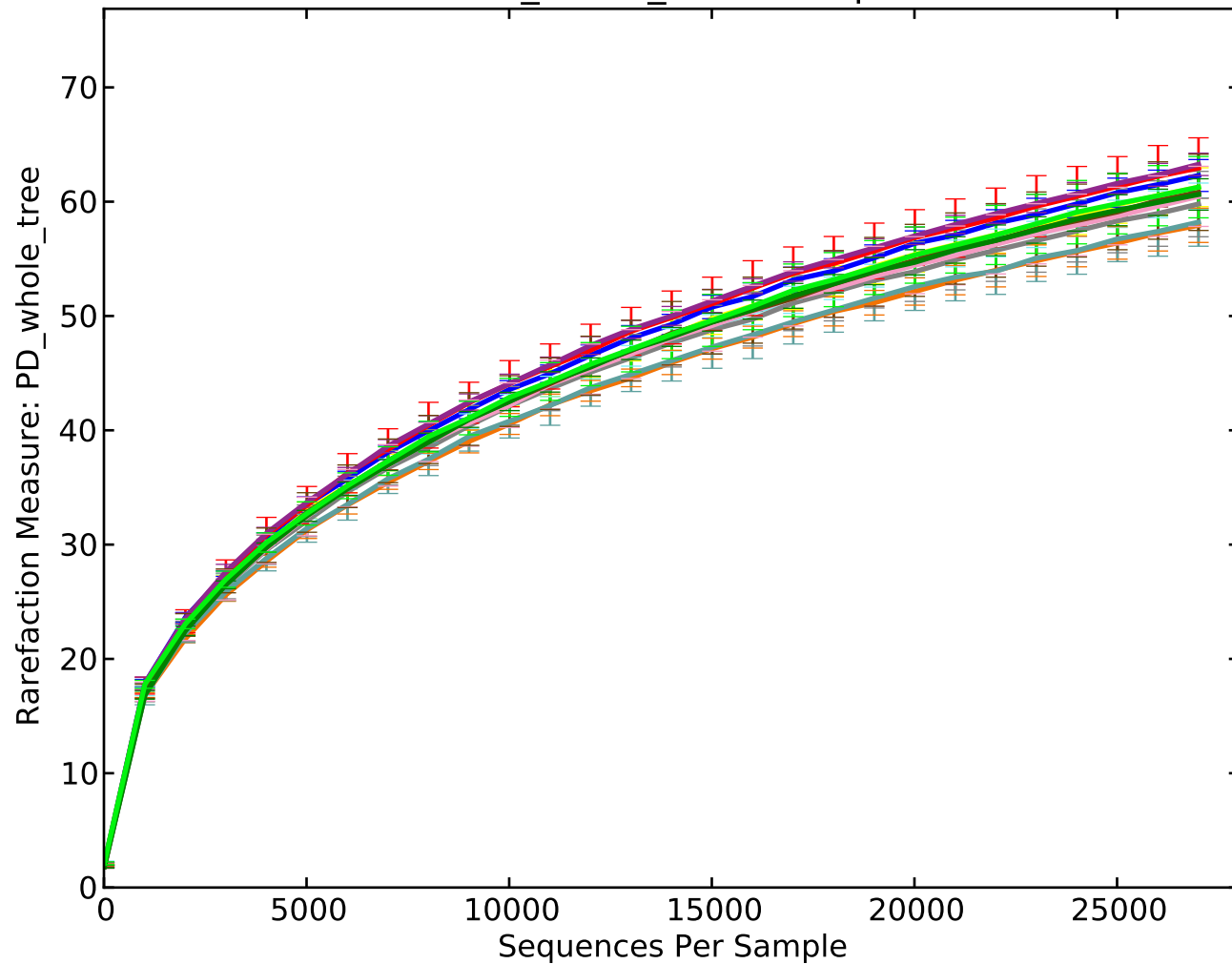

Supplement: Supplementary file 4 — Supplementary Data 1 [file 42003_2023_5520_MOESM4_ESM.zip › 4.Alpha_Diversity/alpha_rarefaction_plot/rarefaction_plots_pdf_depth27686/average_plots/PD_whole_treeGroup.pdf]

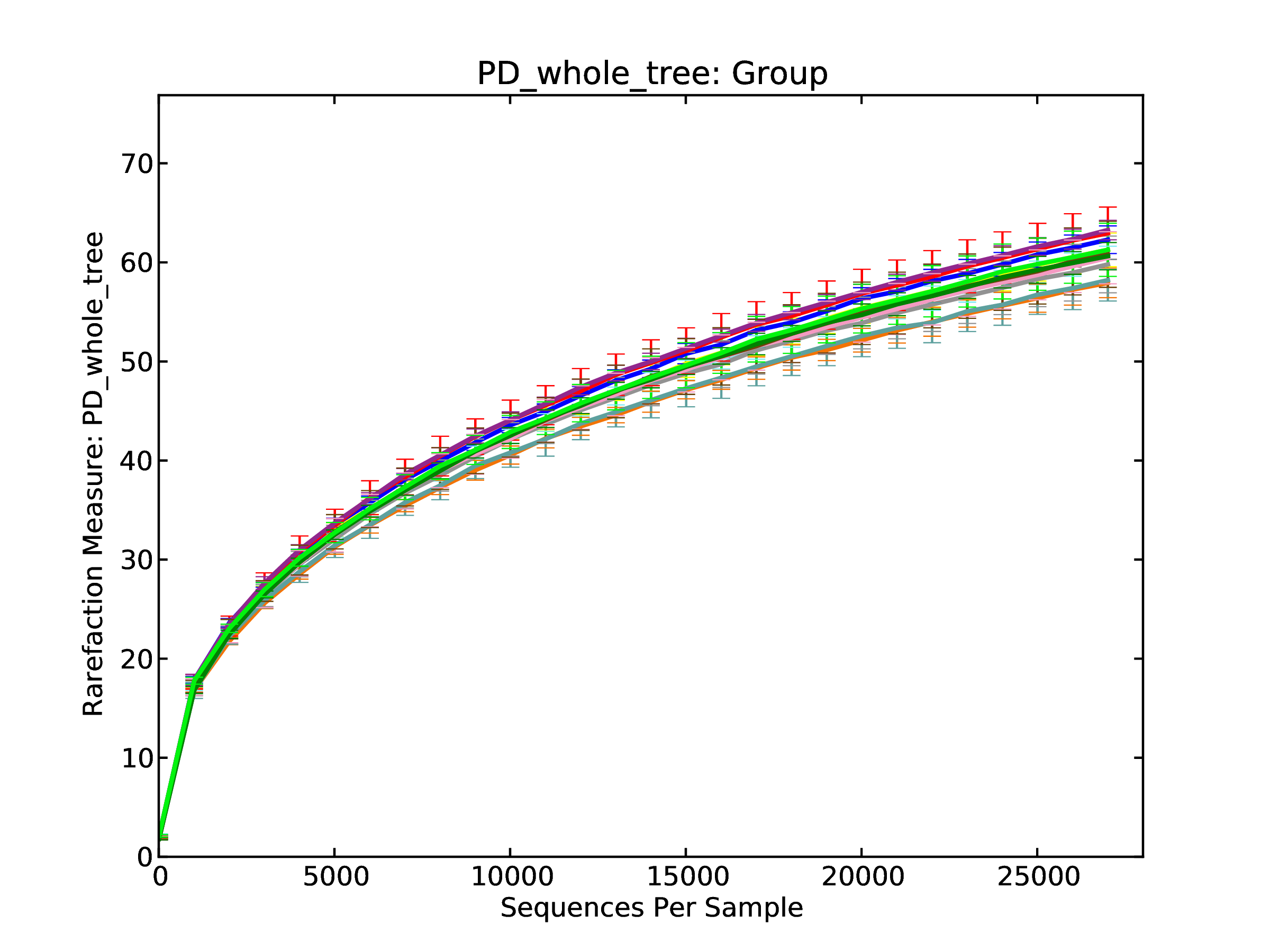

Supplement: Supplementary file 4 — Supplementary Data 1 [file 42003_2023_5520_MOESM4_ESM.zip › 4.Alpha_Diversity/alpha_rarefaction_plot/rarefaction_plots_pdf_depth27686/average_plots/PD_whole_treeGroup.png]

PD\_whole\_tree: LinkerPrimerSequence

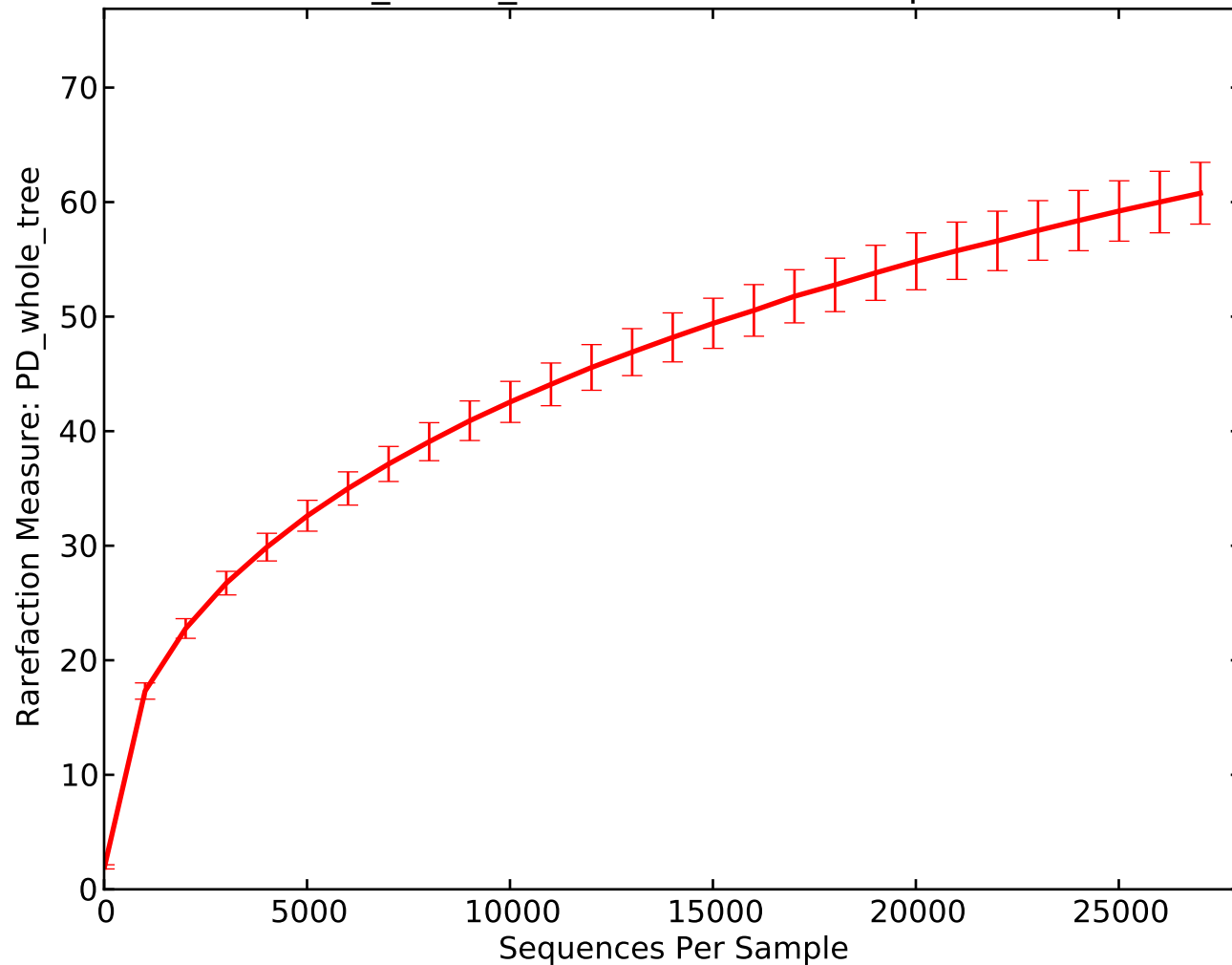

Supplement: Supplementary file 4 — Supplementary Data 1 [file 42003_2023_5520_MOESM4_ESM.zip › 4.Alpha_Diversity/alpha_rarefaction_plot/rarefaction_plots_pdf_depth27686/average_plots/PD_whole_treeLinkerPrimerSequence.pdf]

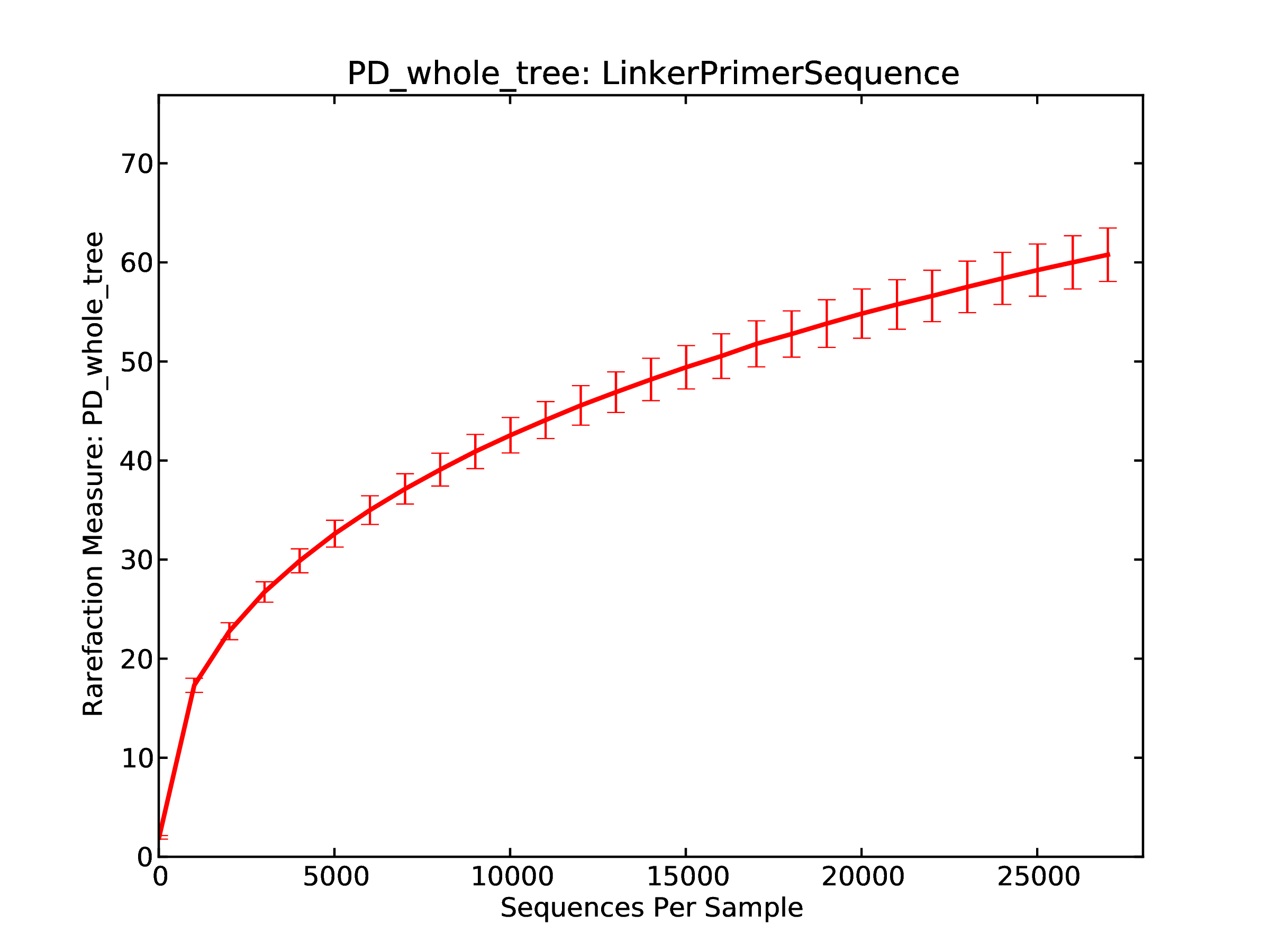

Supplement: Supplementary file 4 — Supplementary Data 1 [file 42003_2023_5520_MOESM4_ESM.zip › 4.Alpha_Diversity/alpha_rarefaction_plot/rarefaction_plots_pdf_depth27686/average_plots/PD_whole_treeLinkerPrimerSequence.png]

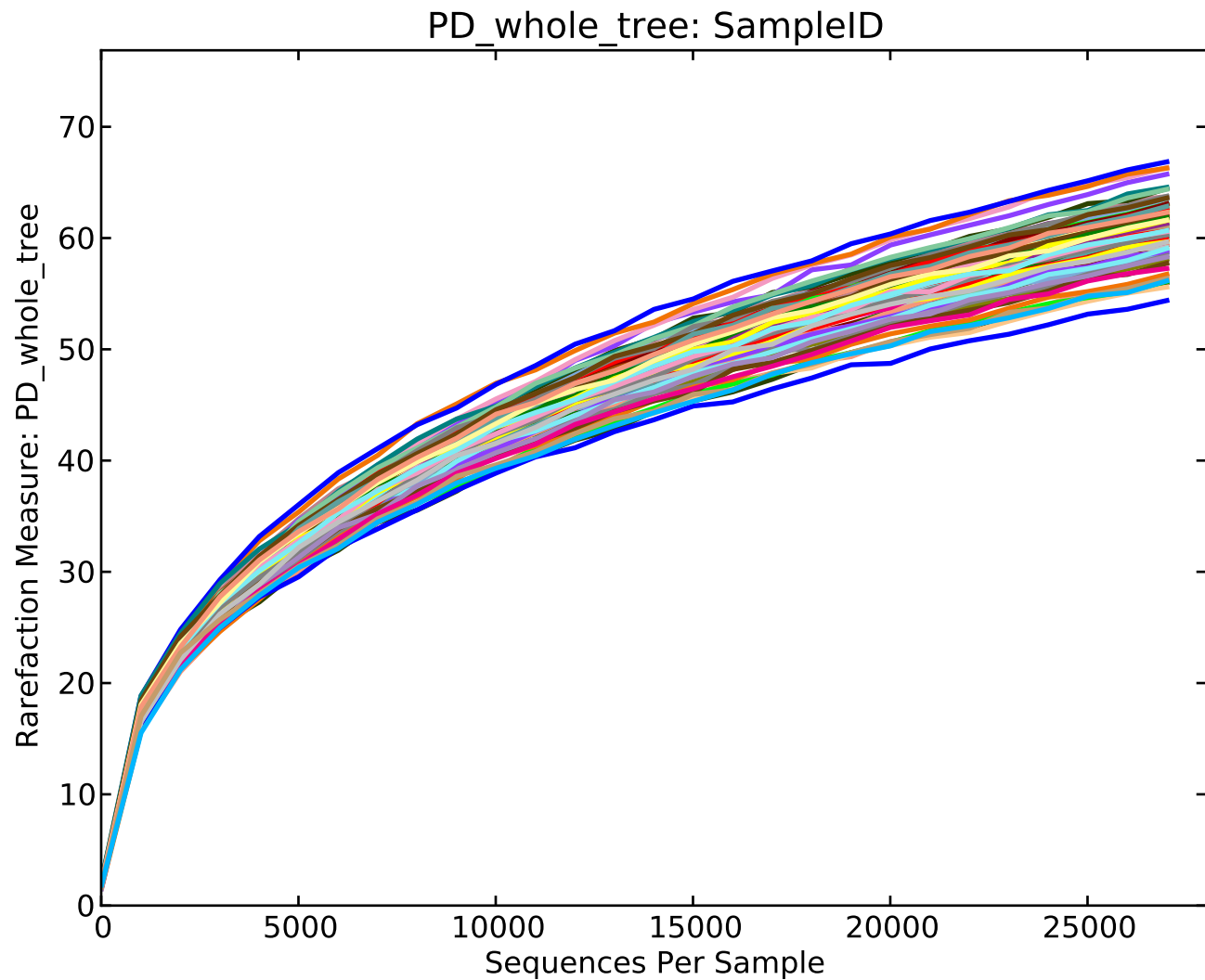

Supplement: Supplementary file 4 — Supplementary Data 1 [file 42003_2023_5520_MOESM4_ESM.zip › 4.Alpha_Diversity/alpha_rarefaction_plot/rarefaction_plots_pdf_depth27686/average_plots/PD_whole_treeSampleID.pdf]

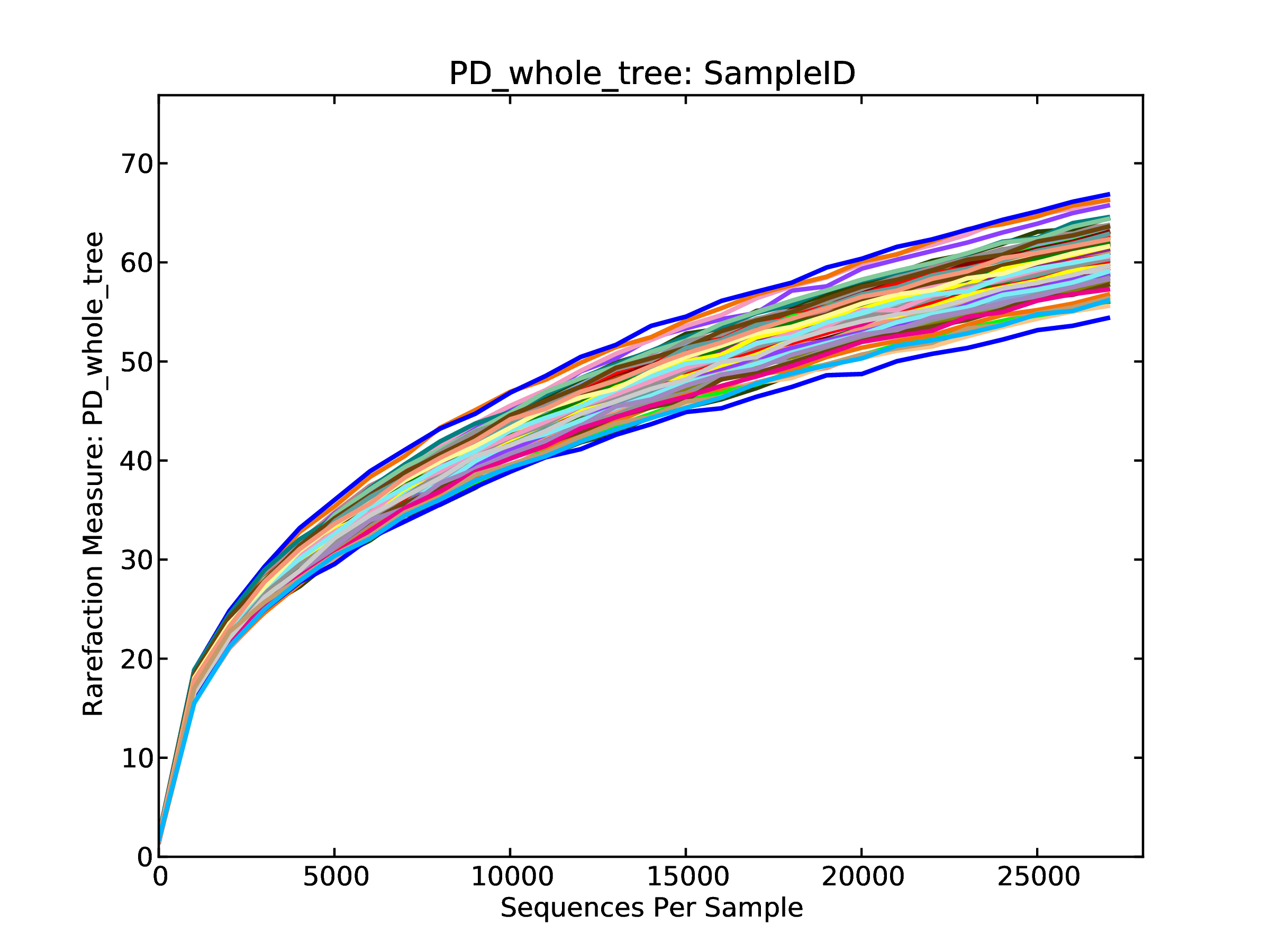

Supplement: Supplementary file 4 — Supplementary Data 1 [file 42003_2023_5520_MOESM4_ESM.zip › 4.Alpha_Diversity/alpha_rarefaction_plot/rarefaction_plots_pdf_depth27686/average_plots/PD_whole_treeSampleID.png]

chao1: BarcodeSequence

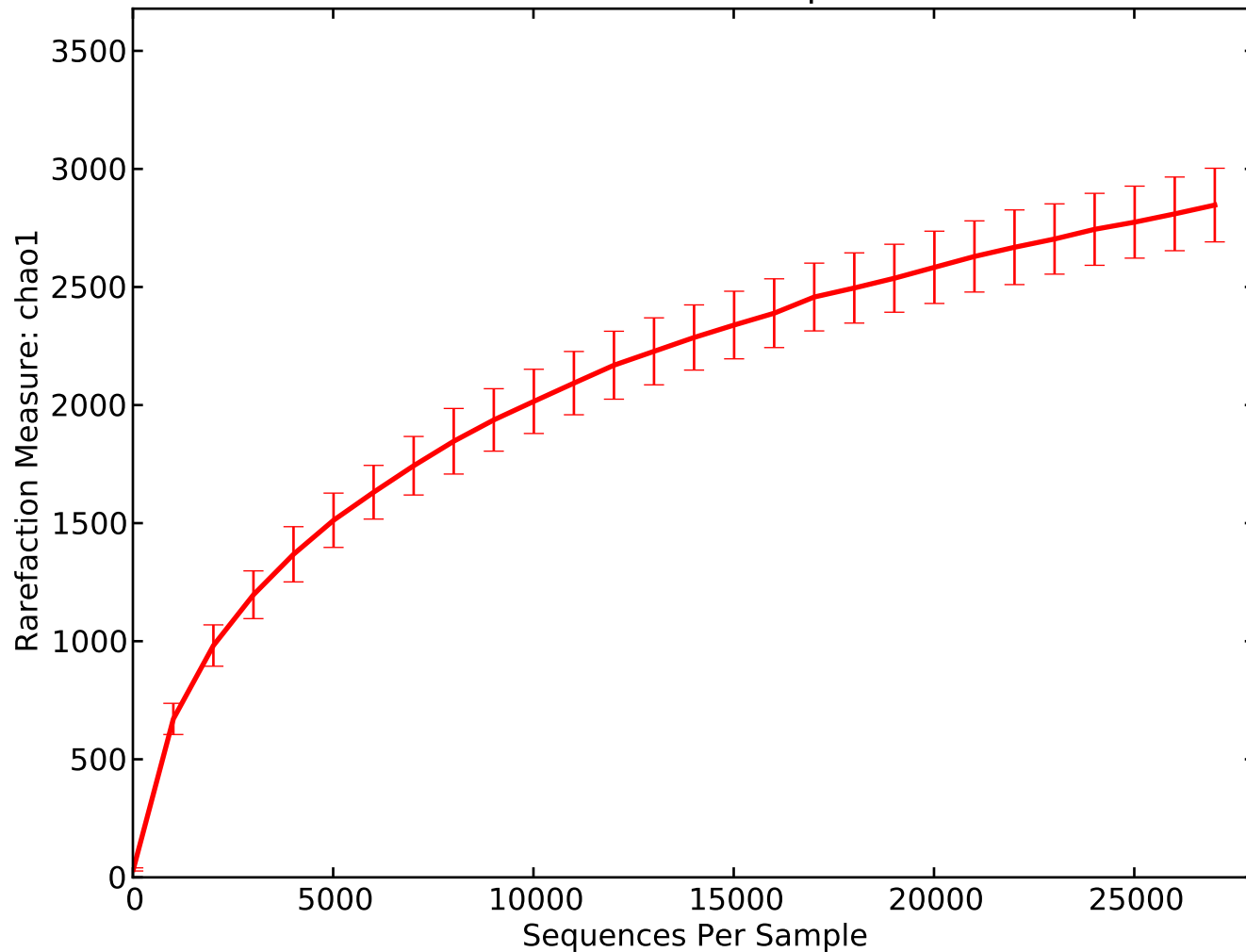

Supplement: Supplementary file 4 — Supplementary Data 1 [file 42003_2023_5520_MOESM4_ESM.zip › 4.Alpha_Diversity/alpha_rarefaction_plot/rarefaction_plots_pdf_depth27686/average_plots/chao1BarcodeSequence.pdf]

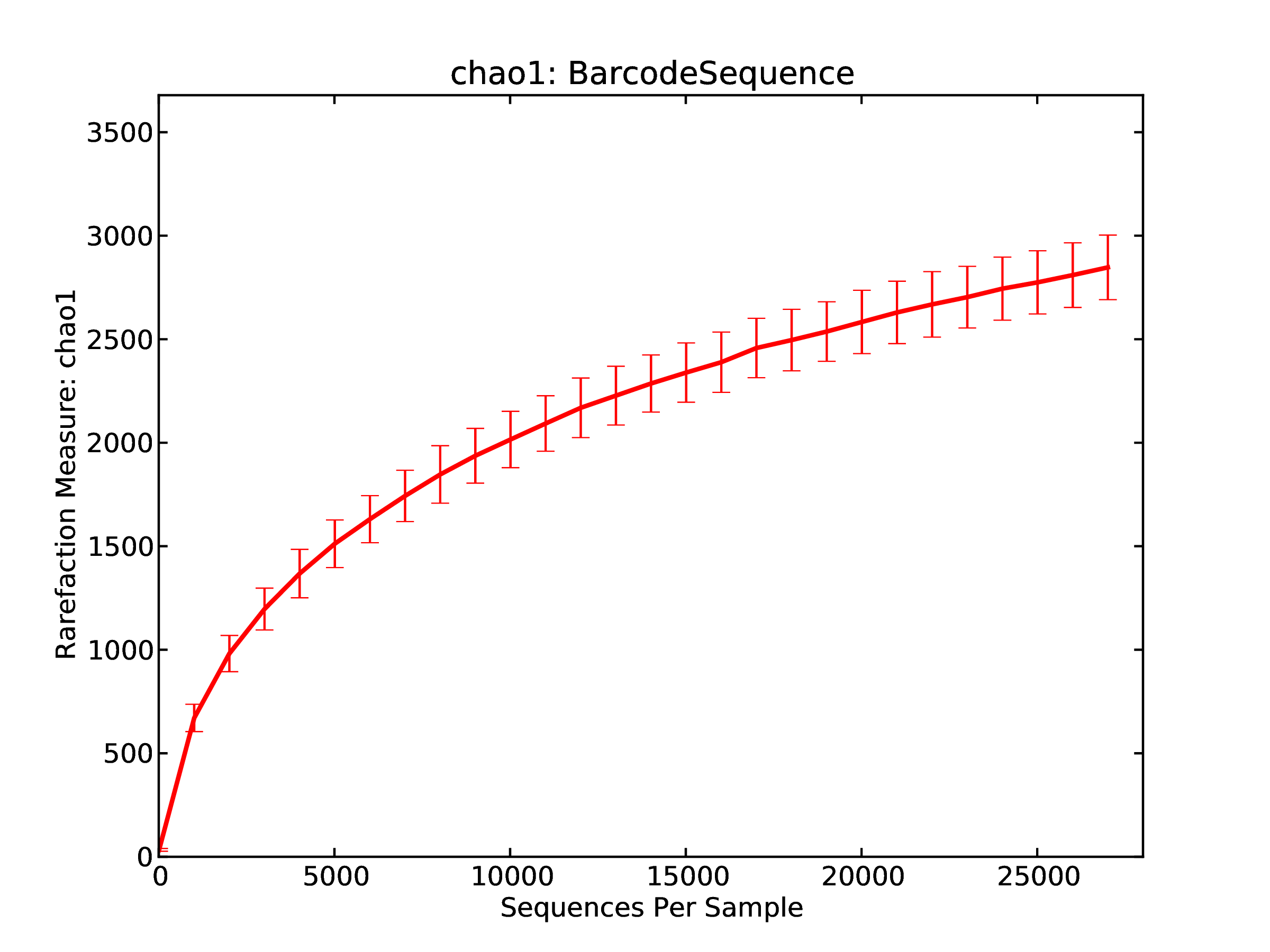

Supplement: Supplementary file 4 — Supplementary Data 1 [file 42003_2023_5520_MOESM4_ESM.zip › 4.Alpha_Diversity/alpha_rarefaction_plot/rarefaction_plots_pdf_depth27686/average_plots/chao1BarcodeSequence.png]

chao1: Description

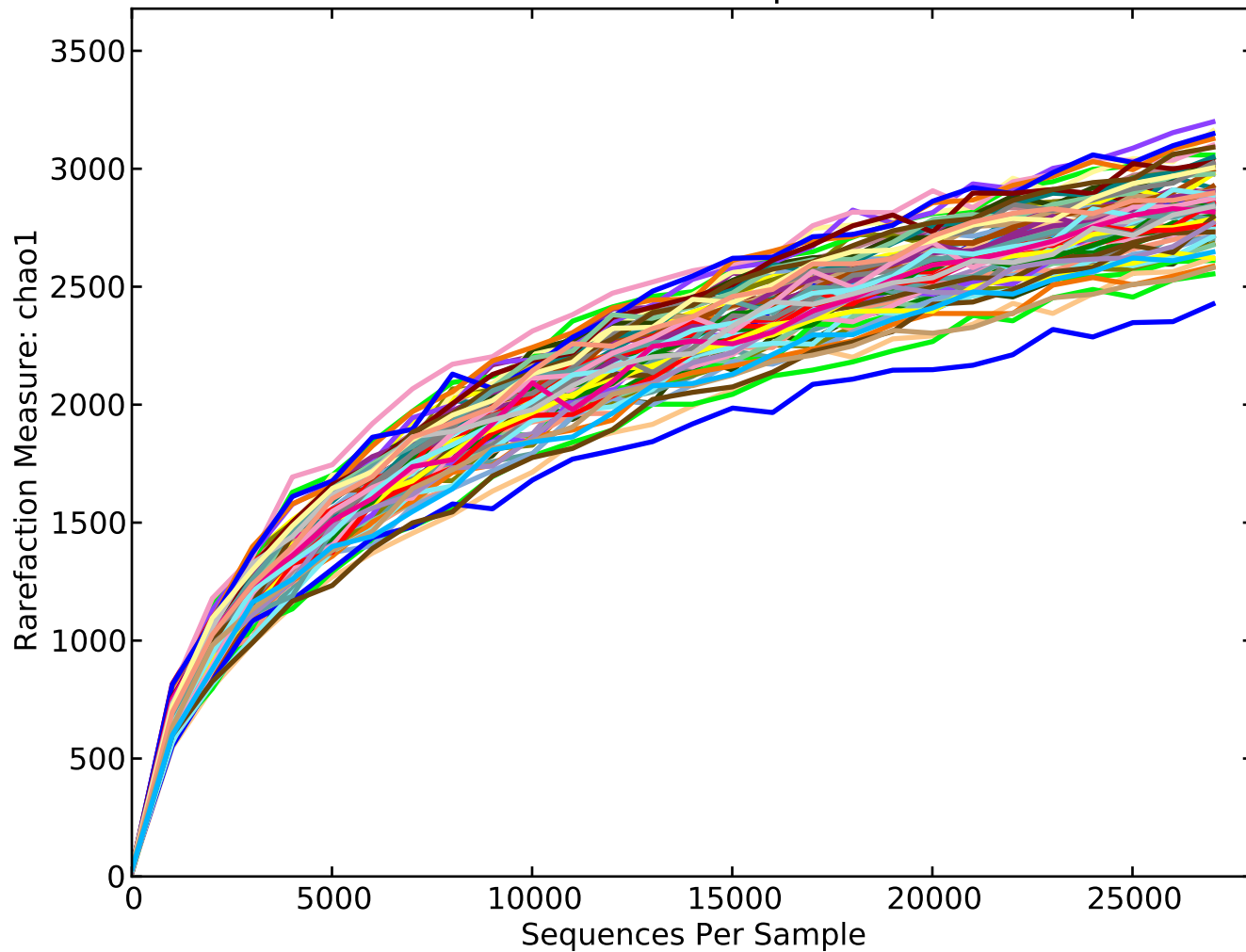

Supplement: Supplementary file 4 — Supplementary Data 1 [file 42003_2023_5520_MOESM4_ESM.zip › 4.Alpha_Diversity/alpha_rarefaction_plot/rarefaction_plots_pdf_depth27686/average_plots/chao1Description.pdf]

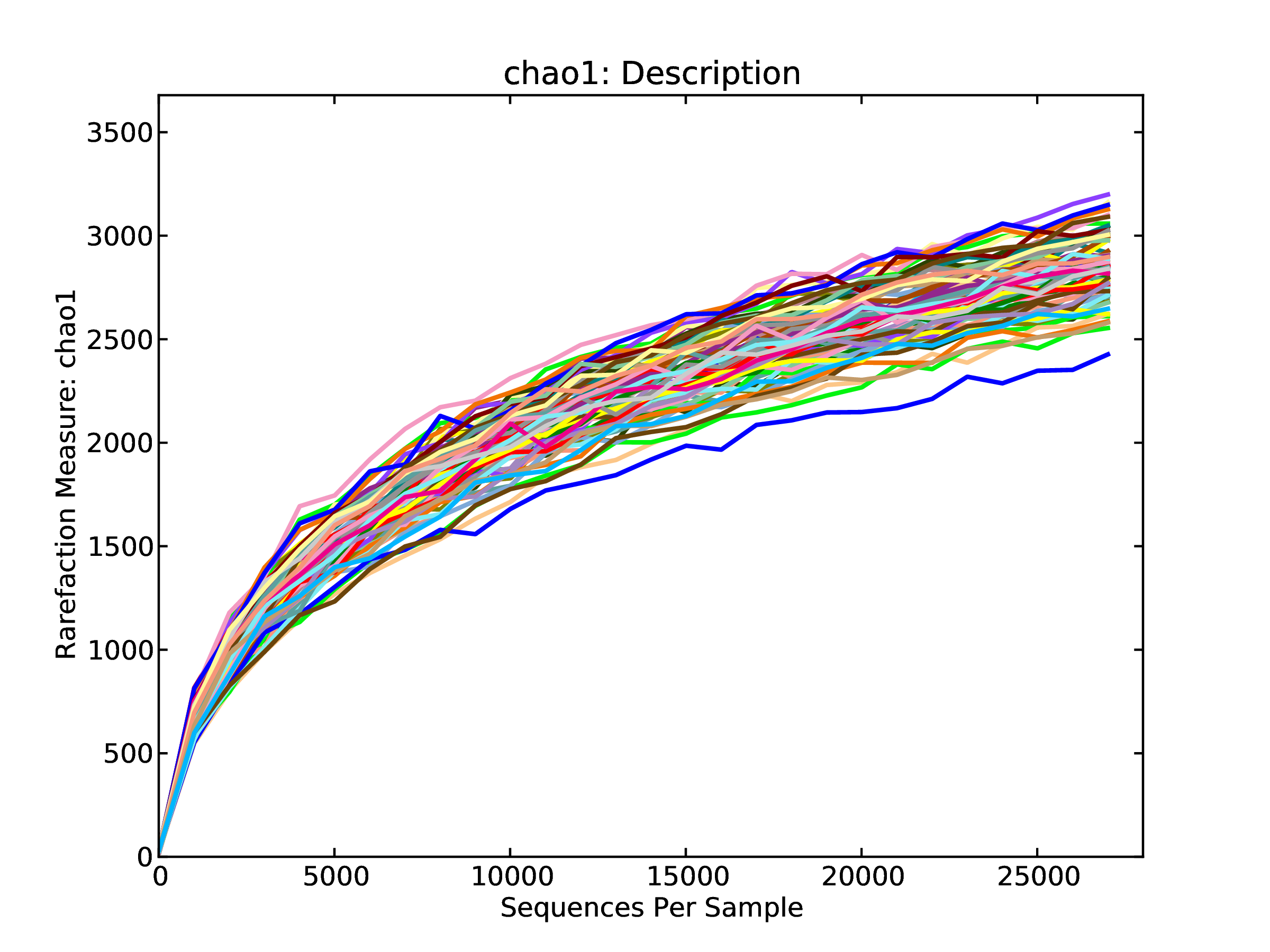

Supplement: Supplementary file 4 — Supplementary Data 1 [file 42003_2023_5520_MOESM4_ESM.zip › 4.Alpha_Diversity/alpha_rarefaction_plot/rarefaction_plots_pdf_depth27686/average_plots/chao1Description.png]

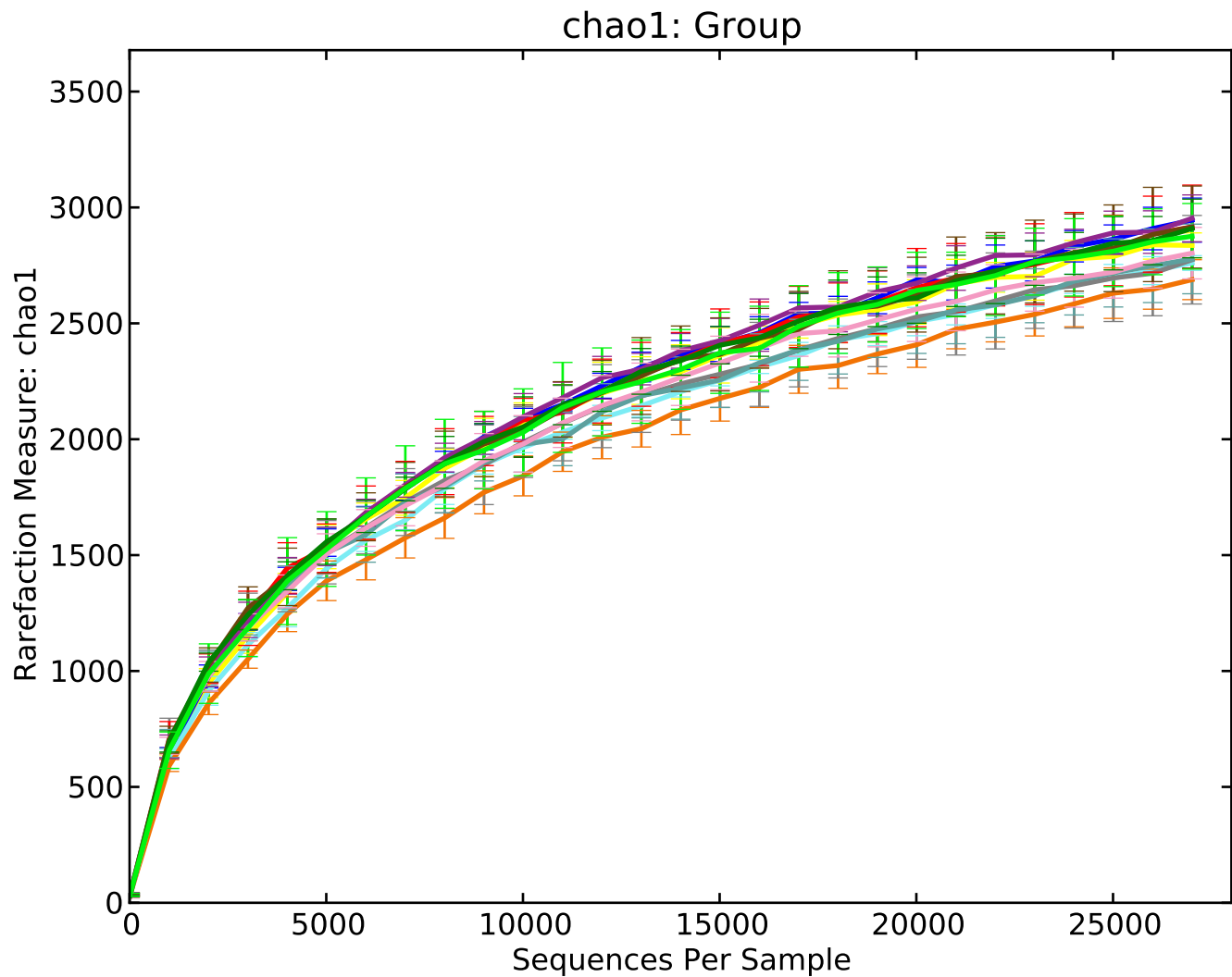

Supplement: Supplementary file 4 — Supplementary Data 1 [file 42003_2023_5520_MOESM4_ESM.zip › 4.Alpha_Diversity/alpha_rarefaction_plot/rarefaction_plots_pdf_depth27686/average_plots/chao1Group.pdf]

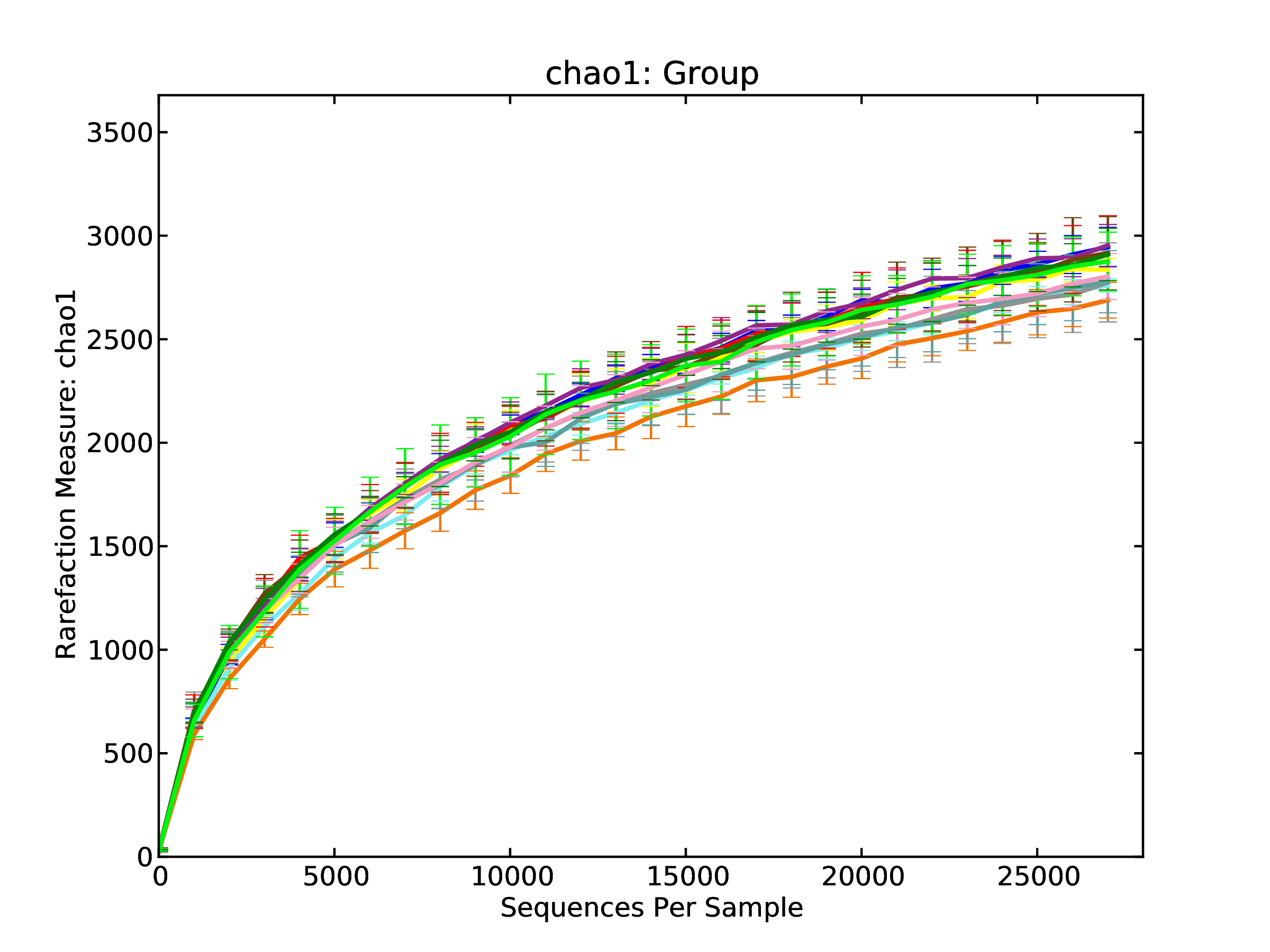

Supplement: Supplementary file 4 — Supplementary Data 1 [file 42003_2023_5520_MOESM4_ESM.zip › 4.Alpha_Diversity/alpha_rarefaction_plot/rarefaction_plots_pdf_depth27686/average_plots/chao1Group.png]

chao1: LinkerPrimerSequence

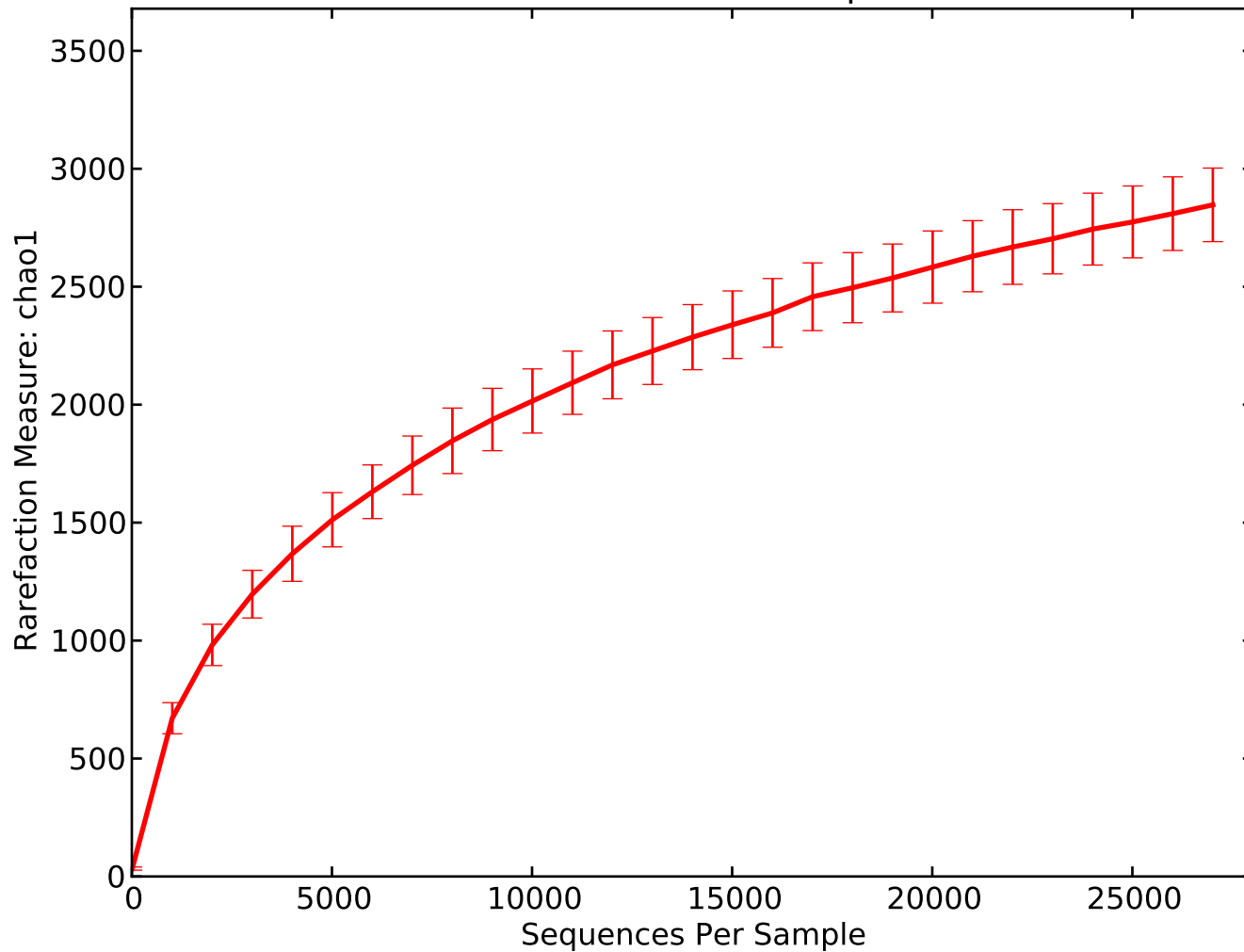

Supplement: Supplementary file 4 — Supplementary Data 1 [file 42003_2023_5520_MOESM4_ESM.zip › 4.Alpha_Diversity/alpha_rarefaction_plot/rarefaction_plots_pdf_depth27686/average_plots/chao1LinkerPrimerSequence.pdf]

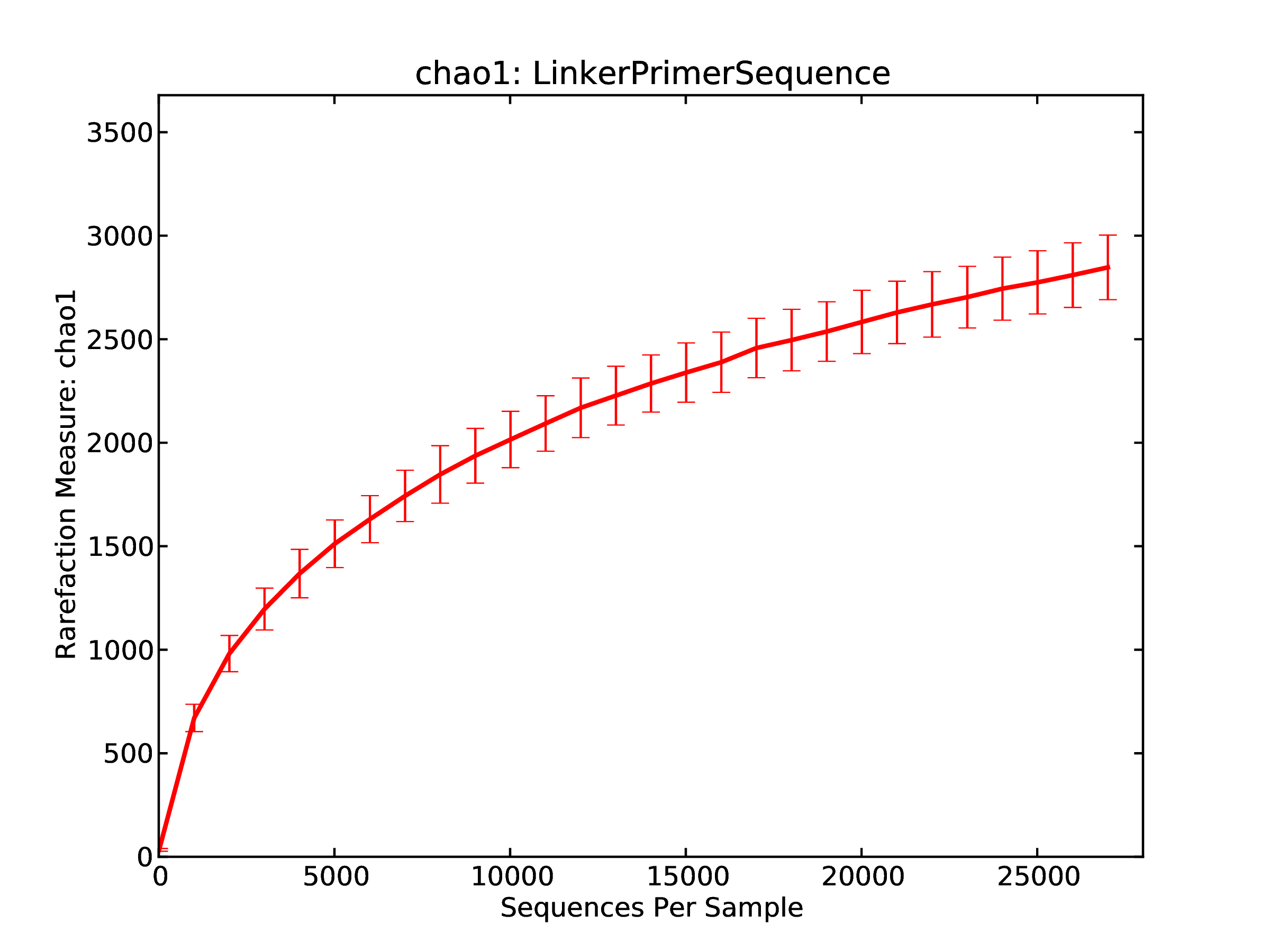

Supplement: Supplementary file 4 — Supplementary Data 1 [file 42003_2023_5520_MOESM4_ESM.zip › 4.Alpha_Diversity/alpha_rarefaction_plot/rarefaction_plots_pdf_depth27686/average_plots/chao1LinkerPrimerSequence.png]

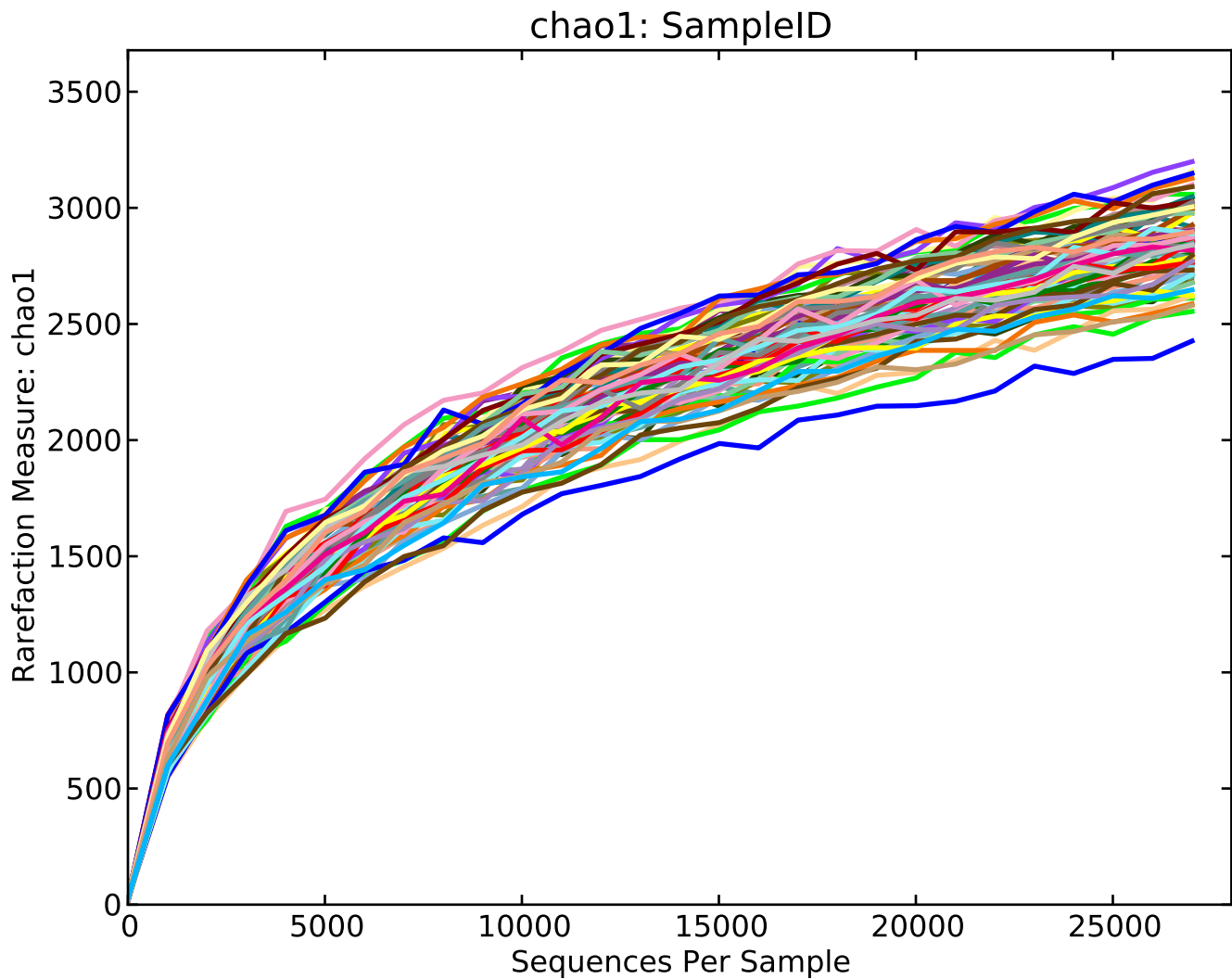

Supplement: Supplementary file 4 — Supplementary Data 1 [file 42003_2023_5520_MOESM4_ESM.zip › 4.Alpha_Diversity/alpha_rarefaction_plot/rarefaction_plots_pdf_depth27686/average_plots/chao1SampleID.pdf]

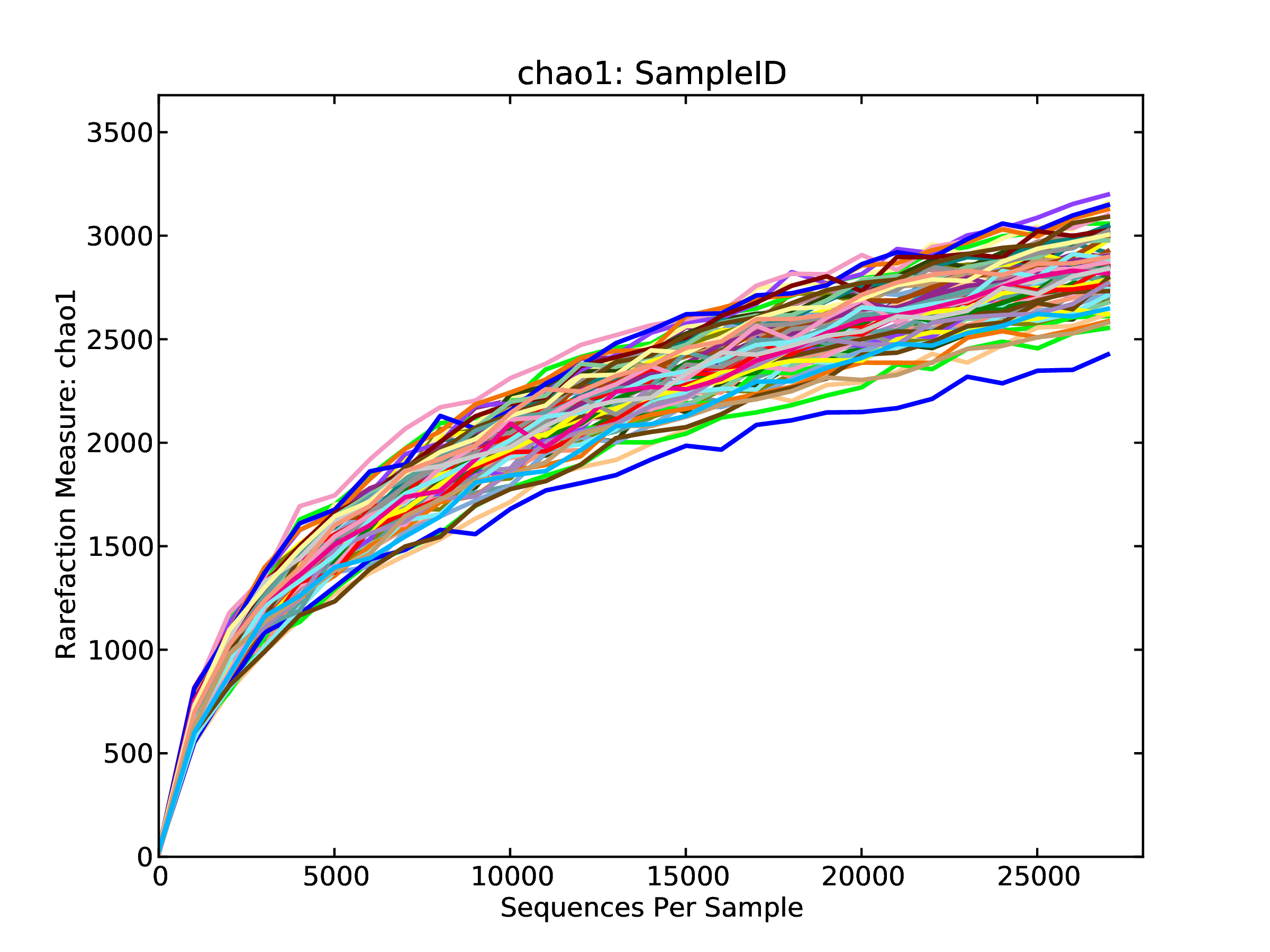

Supplement: Supplementary file 4 — Supplementary Data 1 [file 42003_2023_5520_MOESM4_ESM.zip › 4.Alpha_Diversity/alpha_rarefaction_plot/rarefaction_plots_pdf_depth27686/average_plots/chao1SampleID.png]

goods\_coverage: BarcodeSequence

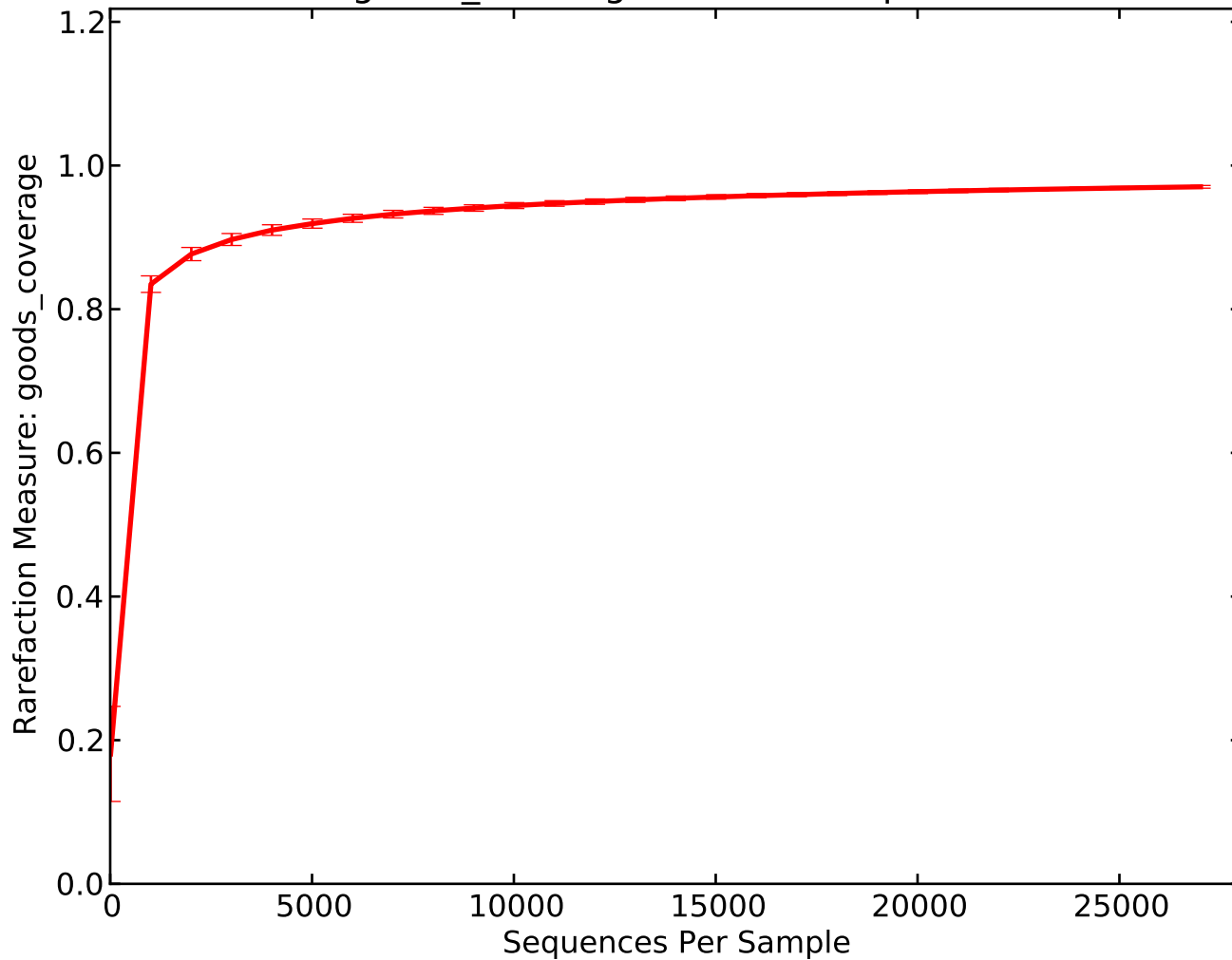

Supplement: Supplementary file 4 — Supplementary Data 1 [file 42003_2023_5520_MOESM4_ESM.zip › 4.Alpha_Diversity/alpha_rarefaction_plot/rarefaction_plots_pdf_depth27686/average_plots/goods_coverageBarcodeSequence.pdf]

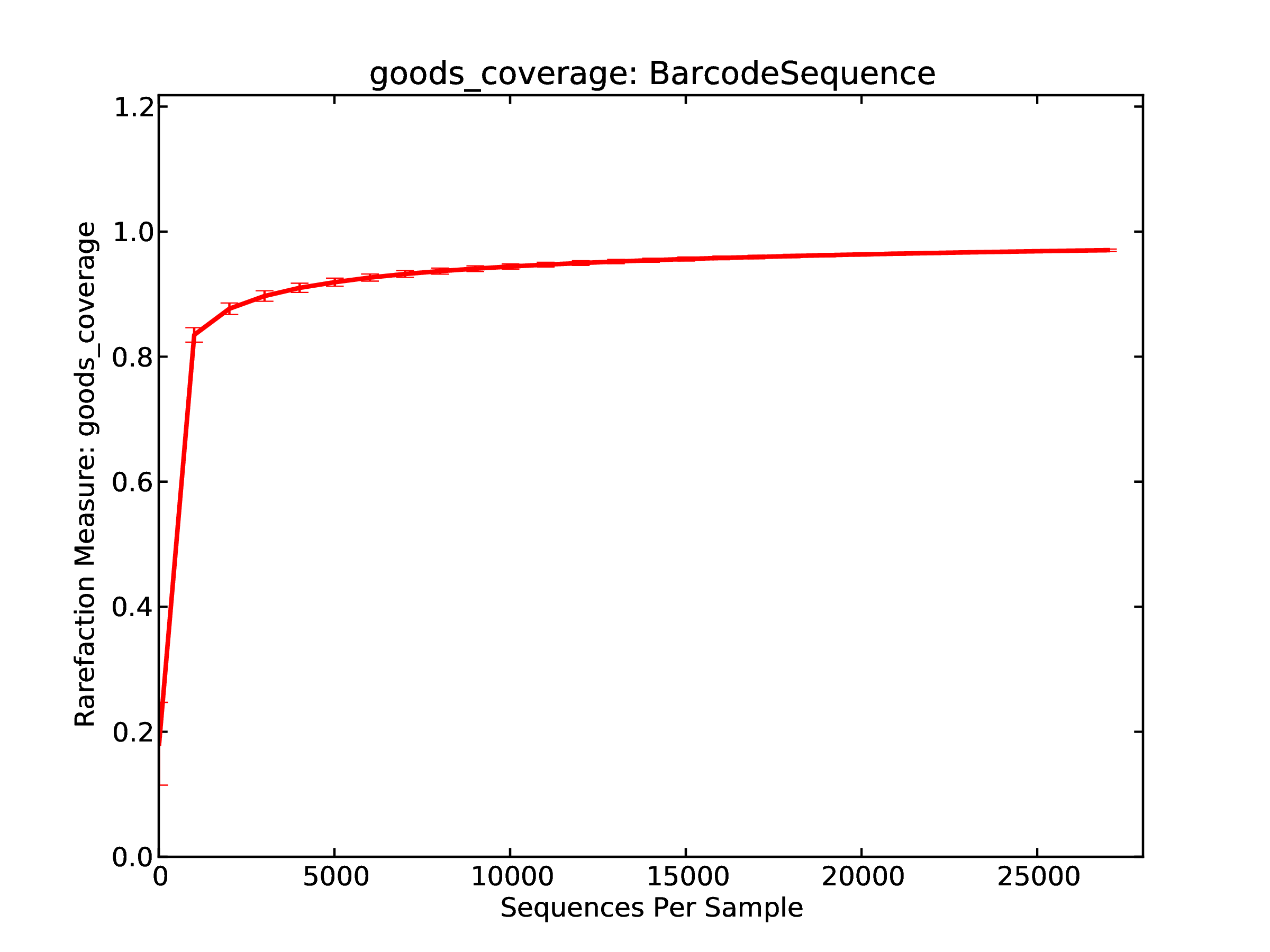

Supplement: Supplementary file 4 — Supplementary Data 1 [file 42003_2023_5520_MOESM4_ESM.zip › 4.Alpha_Diversity/alpha_rarefaction_plot/rarefaction_plots_pdf_depth27686/average_plots/goods_coverageBarcodeSequence.png]

goods\_coverage: Description

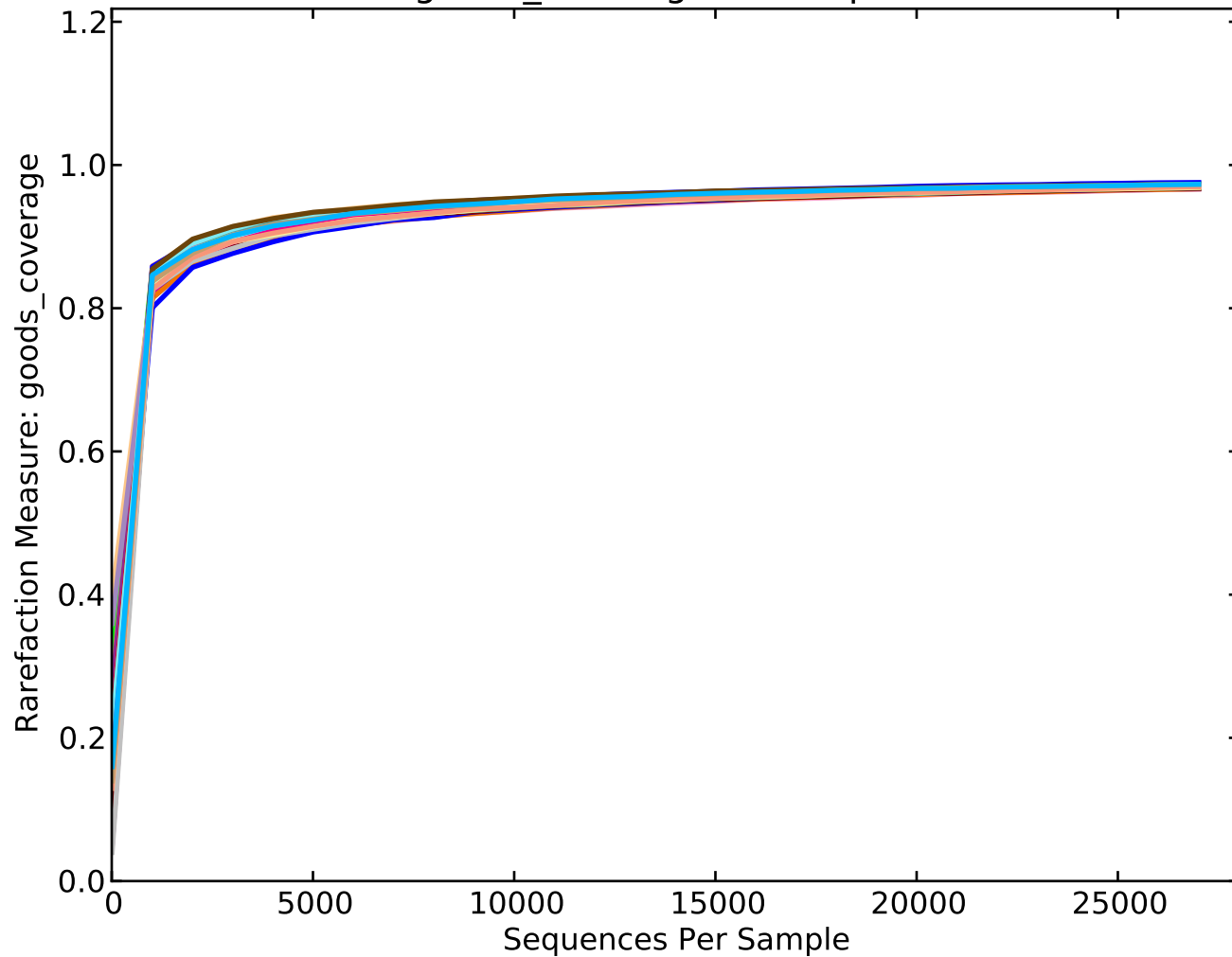

Supplement: Supplementary file 4 — Supplementary Data 1 [file 42003_2023_5520_MOESM4_ESM.zip › 4.Alpha_Diversity/alpha_rarefaction_plot/rarefaction_plots_pdf_depth27686/average_plots/goods_coverageDescription.pdf]

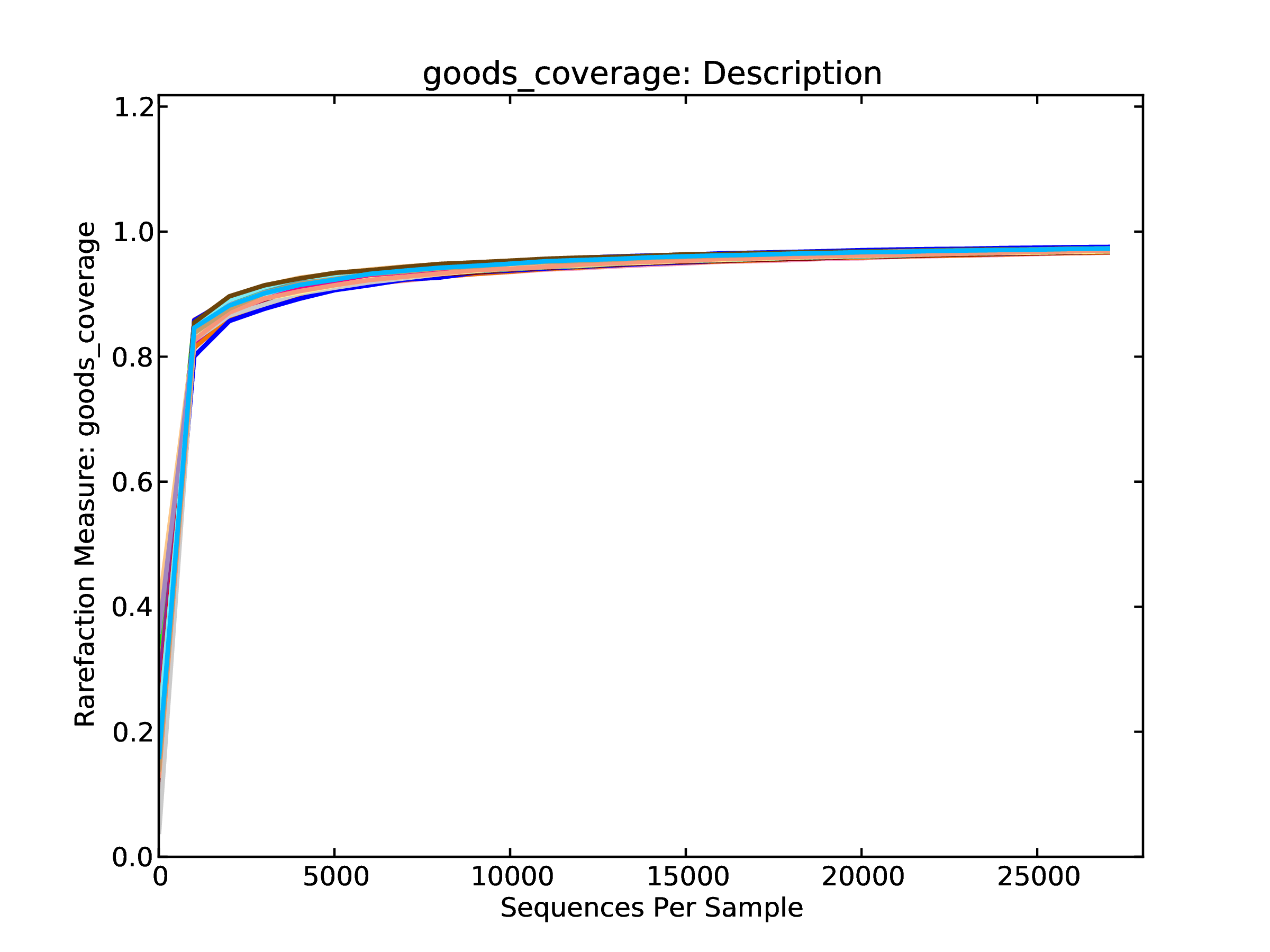

Supplement: Supplementary file 4 — Supplementary Data 1 [file 42003_2023_5520_MOESM4_ESM.zip › 4.Alpha_Diversity/alpha_rarefaction_plot/rarefaction_plots_pdf_depth27686/average_plots/goods_coverageDescription.png]

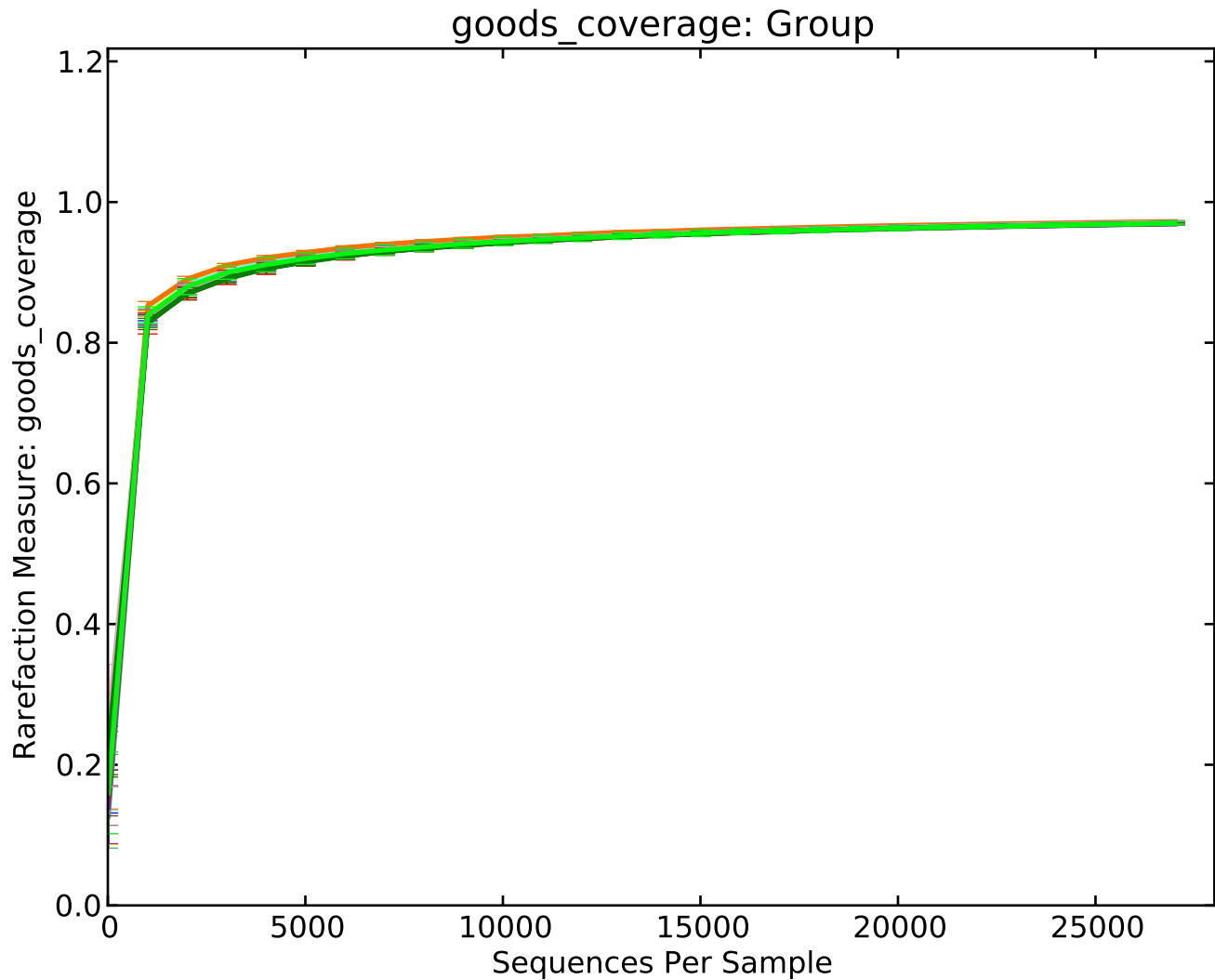

Supplement: Supplementary file 4 — Supplementary Data 1 [file 42003_2023_5520_MOESM4_ESM.zip › 4.Alpha_Diversity/alpha_rarefaction_plot/rarefaction_plots_pdf_depth27686/average_plots/goods_coverageGroup.pdf]

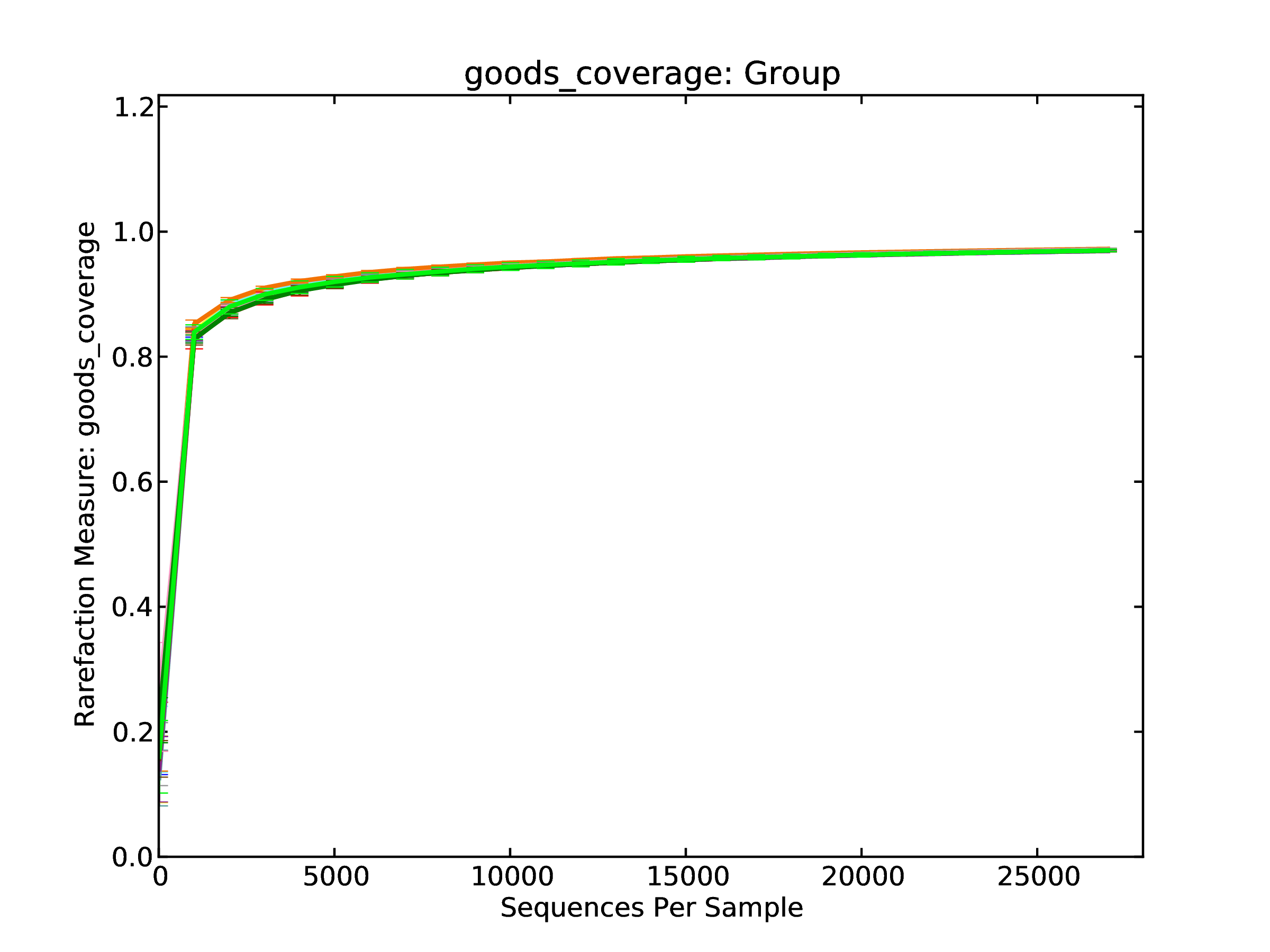

Supplement: Supplementary file 4 — Supplementary Data 1 [file 42003_2023_5520_MOESM4_ESM.zip › 4.Alpha_Diversity/alpha_rarefaction_plot/rarefaction_plots_pdf_depth27686/average_plots/goods_coverageGroup.png]

goods\_coverage: LinkerPrimerSequence

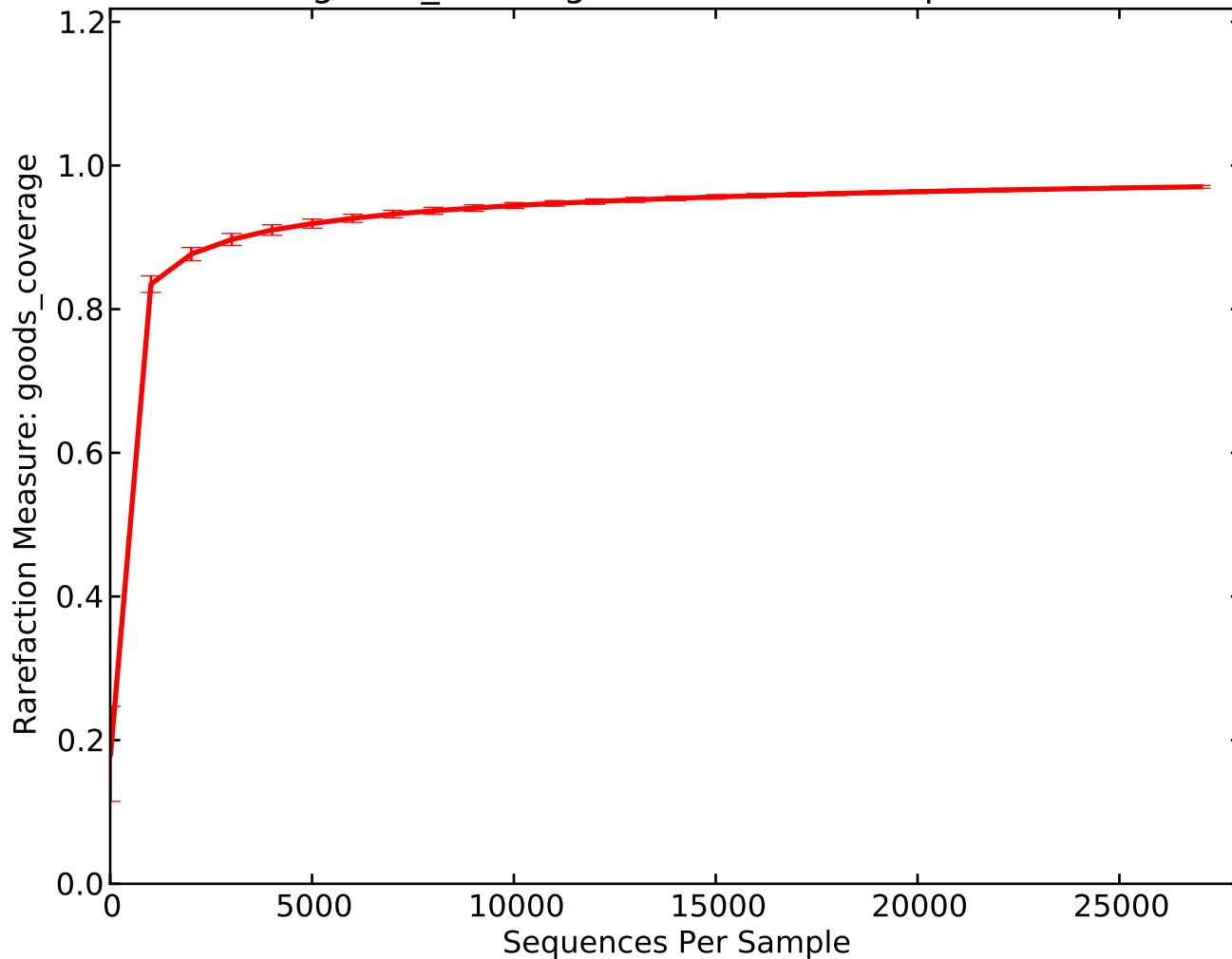

Supplement: Supplementary file 4 — Supplementary Data 1 [file 42003_2023_5520_MOESM4_ESM.zip › 4.Alpha_Diversity/alpha_rarefaction_plot/rarefaction_plots_pdf_depth27686/average_plots/goods_coverageLinkerPrimerSequence.pdf]

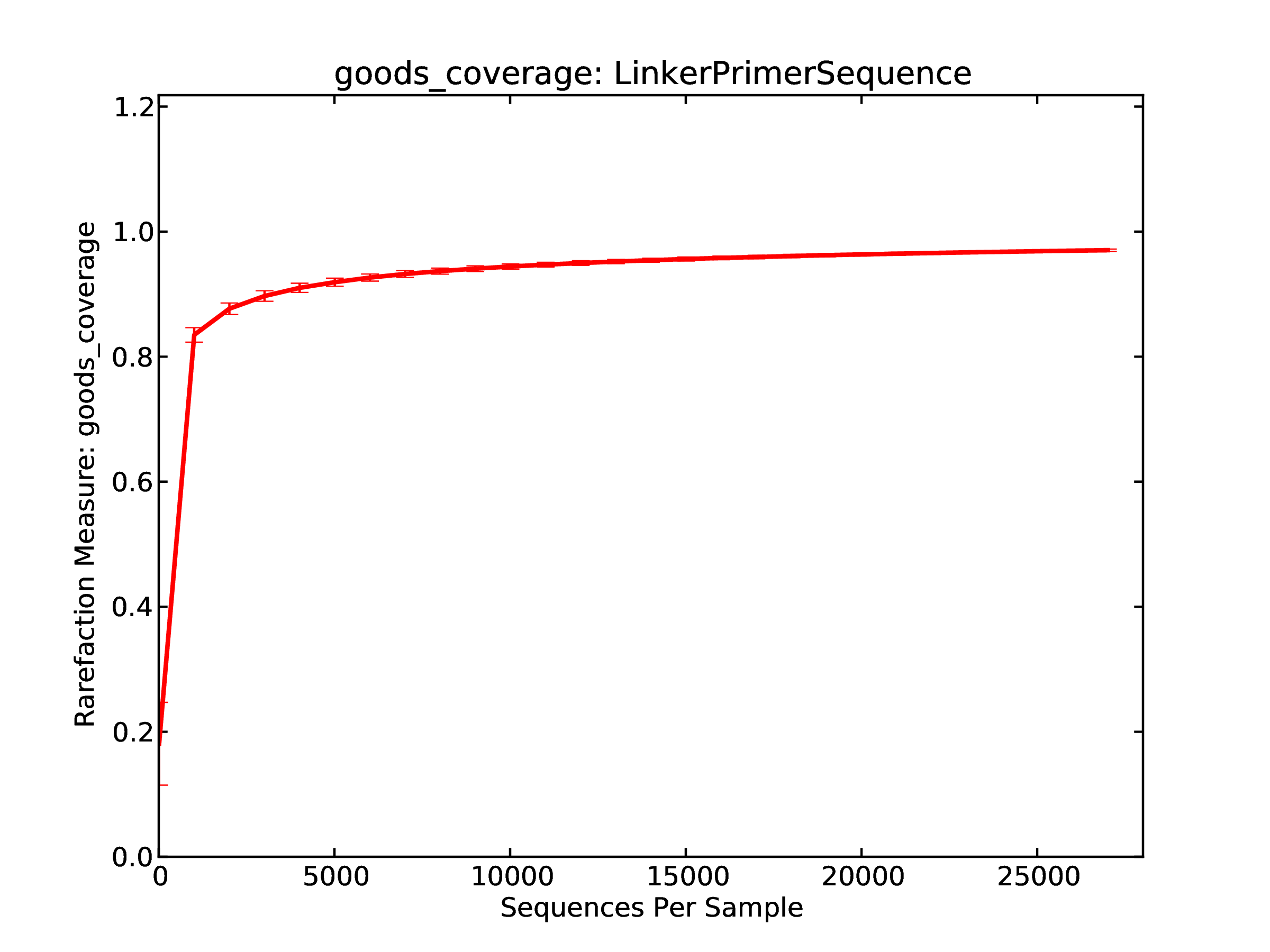

Supplement: Supplementary file 4 — Supplementary Data 1 [file 42003_2023_5520_MOESM4_ESM.zip › 4.Alpha_Diversity/alpha_rarefaction_plot/rarefaction_plots_pdf_depth27686/average_plots/goods_coverageLinkerPrimerSequence.png]

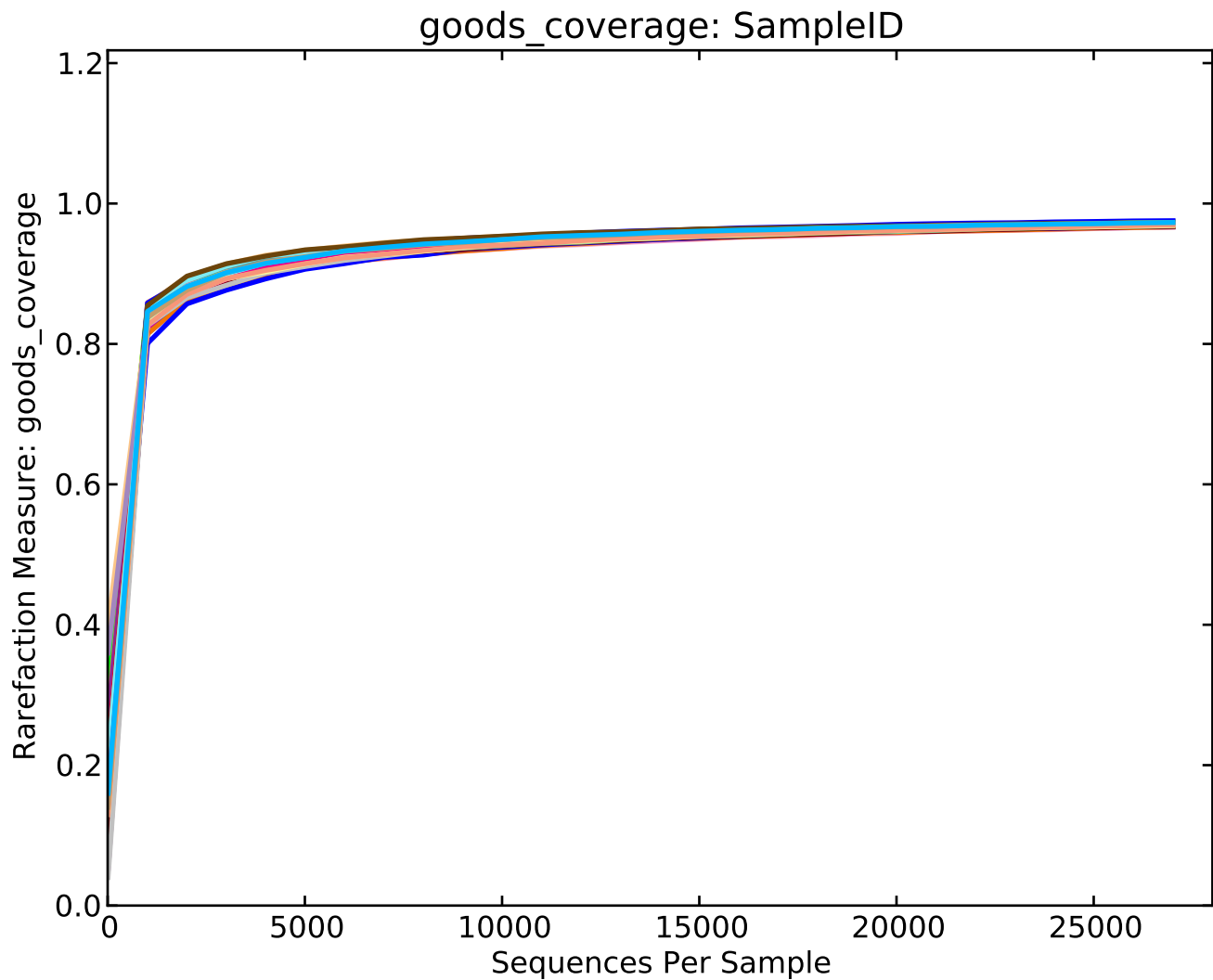

Supplement: Supplementary file 4 — Supplementary Data 1 [file 42003_2023_5520_MOESM4_ESM.zip › 4.Alpha_Diversity/alpha_rarefaction_plot/rarefaction_plots_pdf_depth27686/average_plots/goods_coverageSampleID.pdf]

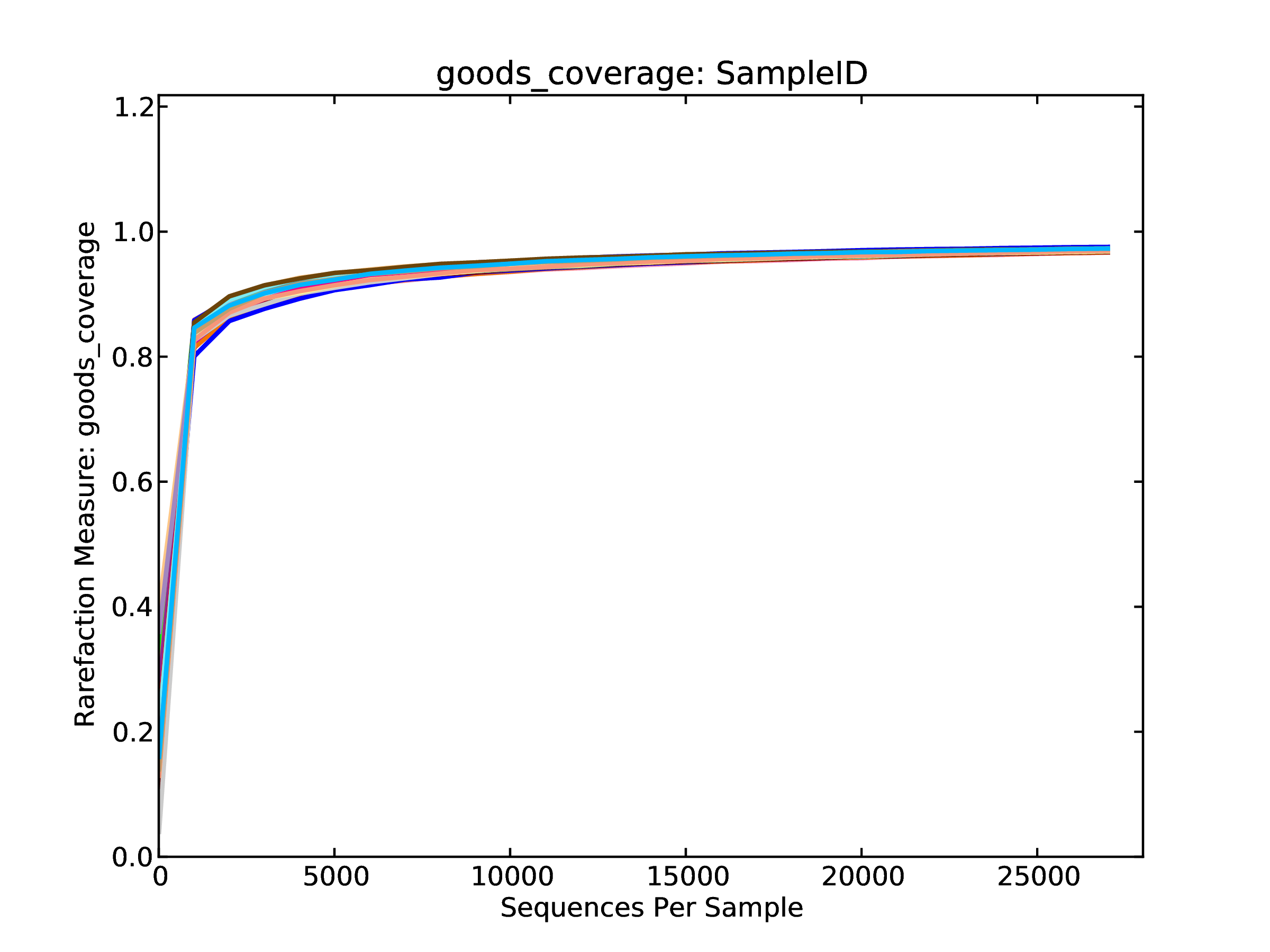

Supplement: Supplementary file 4 — Supplementary Data 1 [file 42003_2023_5520_MOESM4_ESM.zip › 4.Alpha_Diversity/alpha_rarefaction_plot/rarefaction_plots_pdf_depth27686/average_plots/goods_coverageSampleID.png]

observed\_species: BarcodeSequence

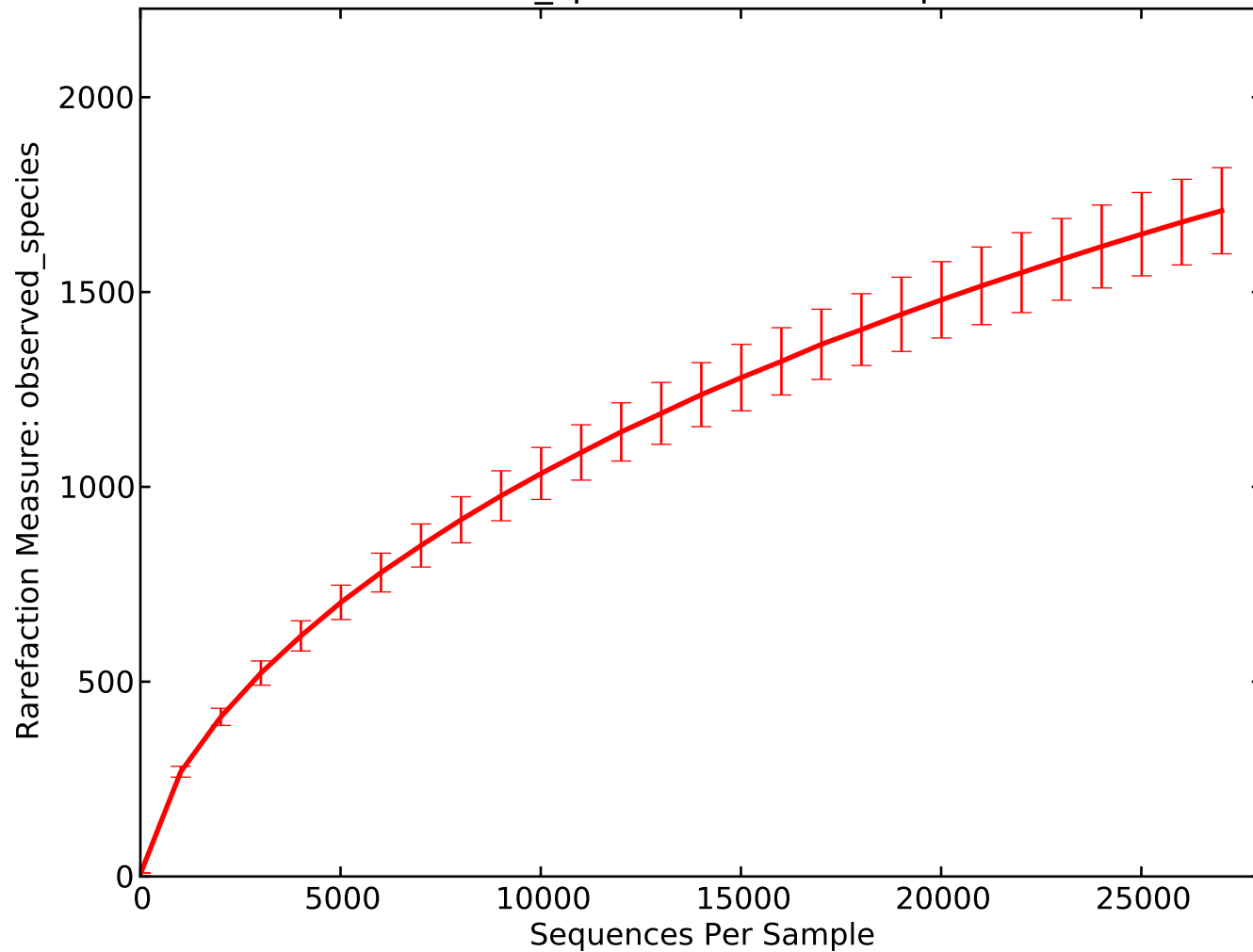

Supplement: Supplementary file 4 — Supplementary Data 1 [file 42003_2023_5520_MOESM4_ESM.zip › 4.Alpha_Diversity/alpha_rarefaction_plot/rarefaction_plots_pdf_depth27686/average_plots/observed_speciesBarcodeSequence.pdf]

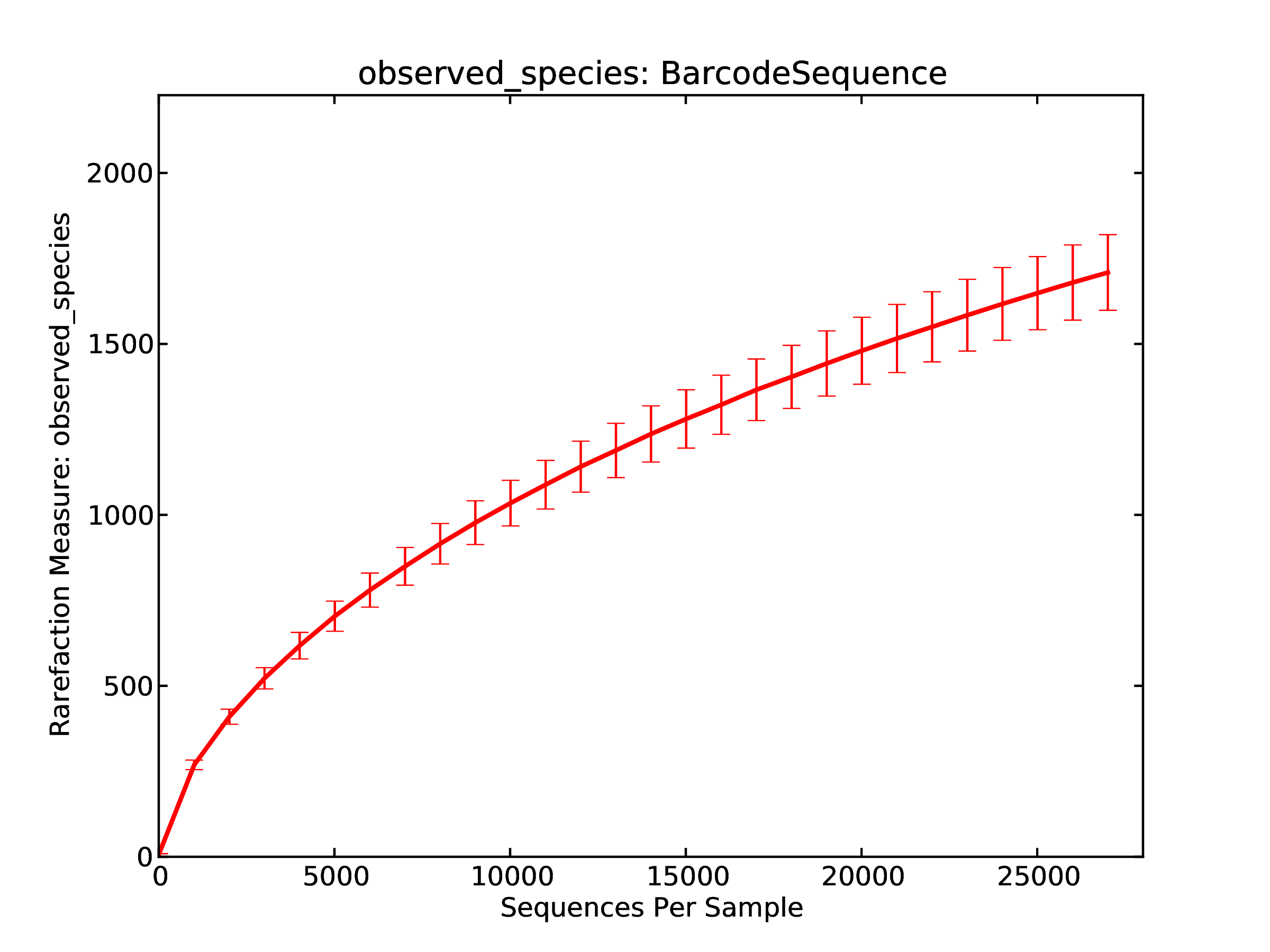

Supplement: Supplementary file 4 — Supplementary Data 1 [file 42003_2023_5520_MOESM4_ESM.zip › 4.Alpha_Diversity/alpha_rarefaction_plot/rarefaction_plots_pdf_depth27686/average_plots/observed_speciesBarcodeSequence.png]

observed\_species: Description

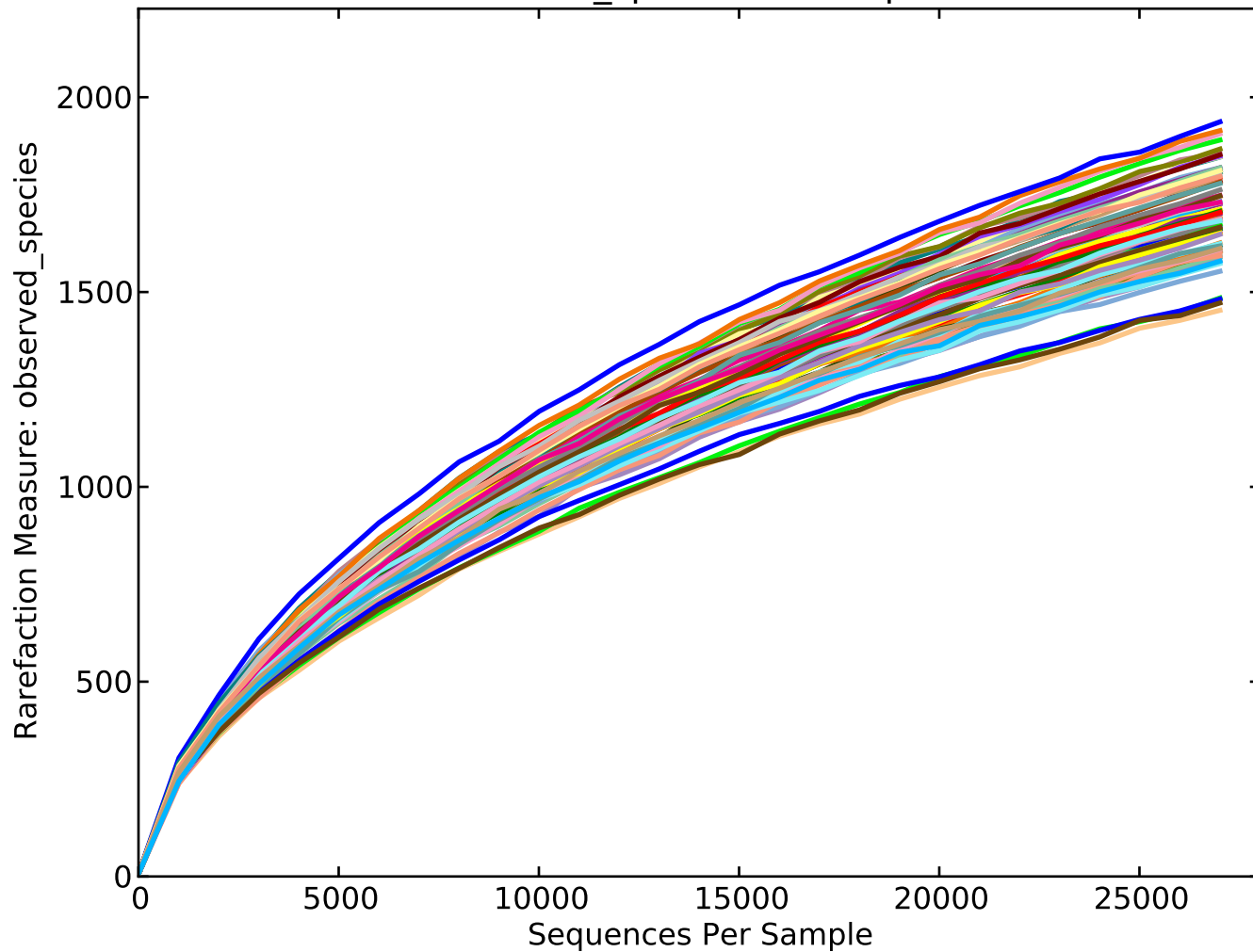

Supplement: Supplementary file 4 — Supplementary Data 1 [file 42003_2023_5520_MOESM4_ESM.zip › 4.Alpha_Diversity/alpha_rarefaction_plot/rarefaction_plots_pdf_depth27686/average_plots/observed_speciesDescription.pdf]

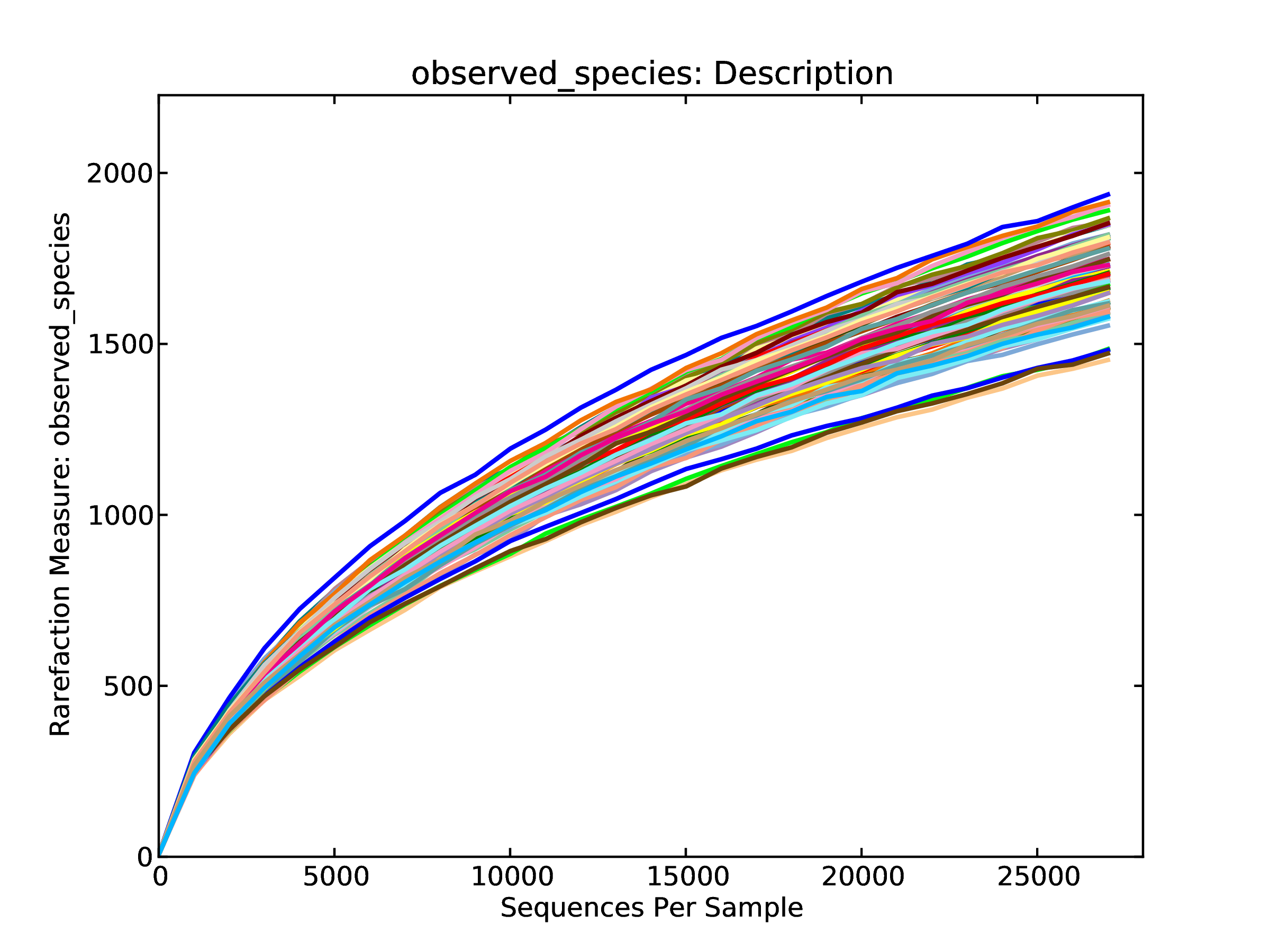

Supplement: Supplementary file 4 — Supplementary Data 1 [file 42003_2023_5520_MOESM4_ESM.zip › 4.Alpha_Diversity/alpha_rarefaction_plot/rarefaction_plots_pdf_depth27686/average_plots/observed_speciesDescription.png]

observed\_species: Group

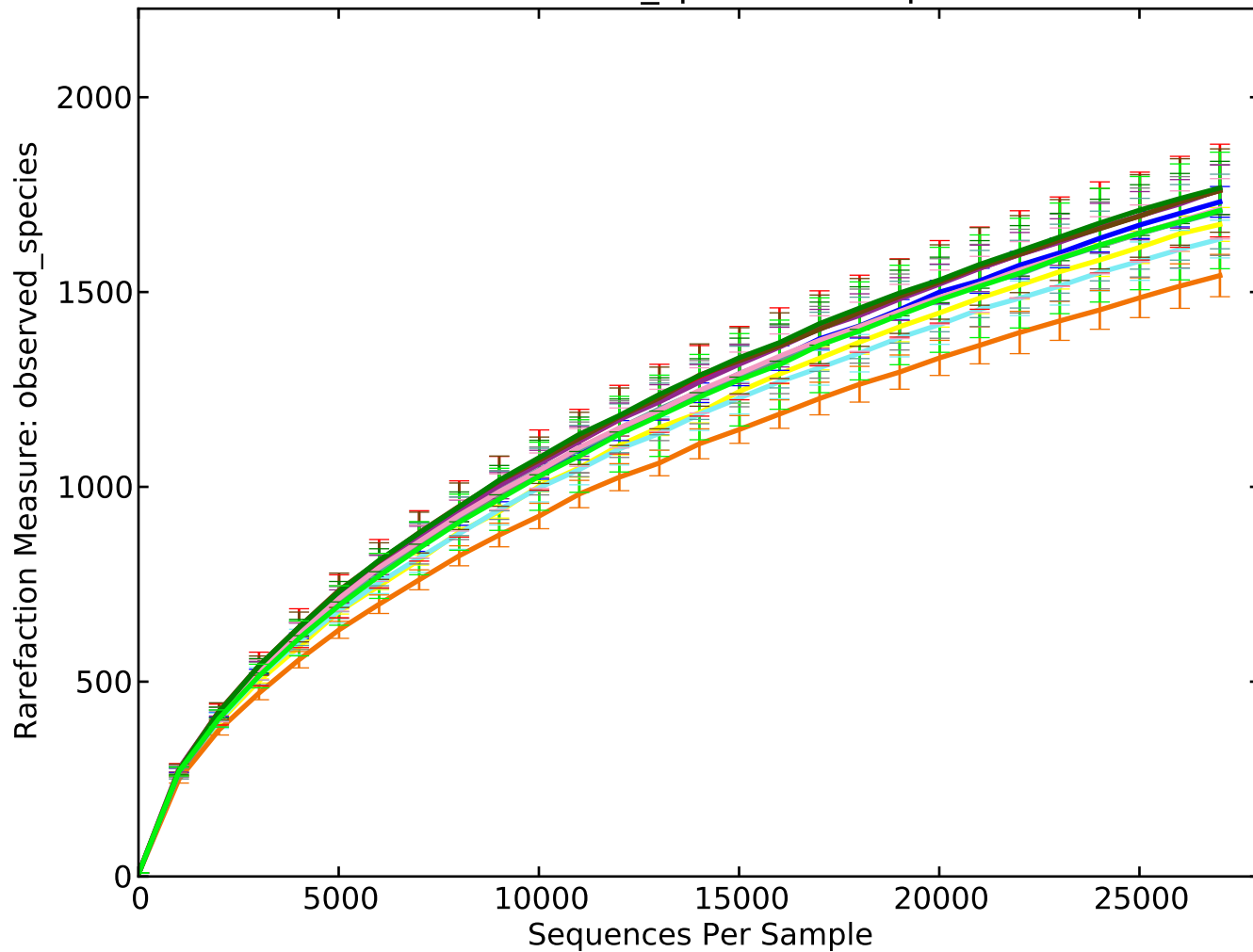

Supplement: Supplementary file 4 — Supplementary Data 1 [file 42003_2023_5520_MOESM4_ESM.zip › 4.Alpha_Diversity/alpha_rarefaction_plot/rarefaction_plots_pdf_depth27686/average_plots/observed_speciesGroup.pdf]

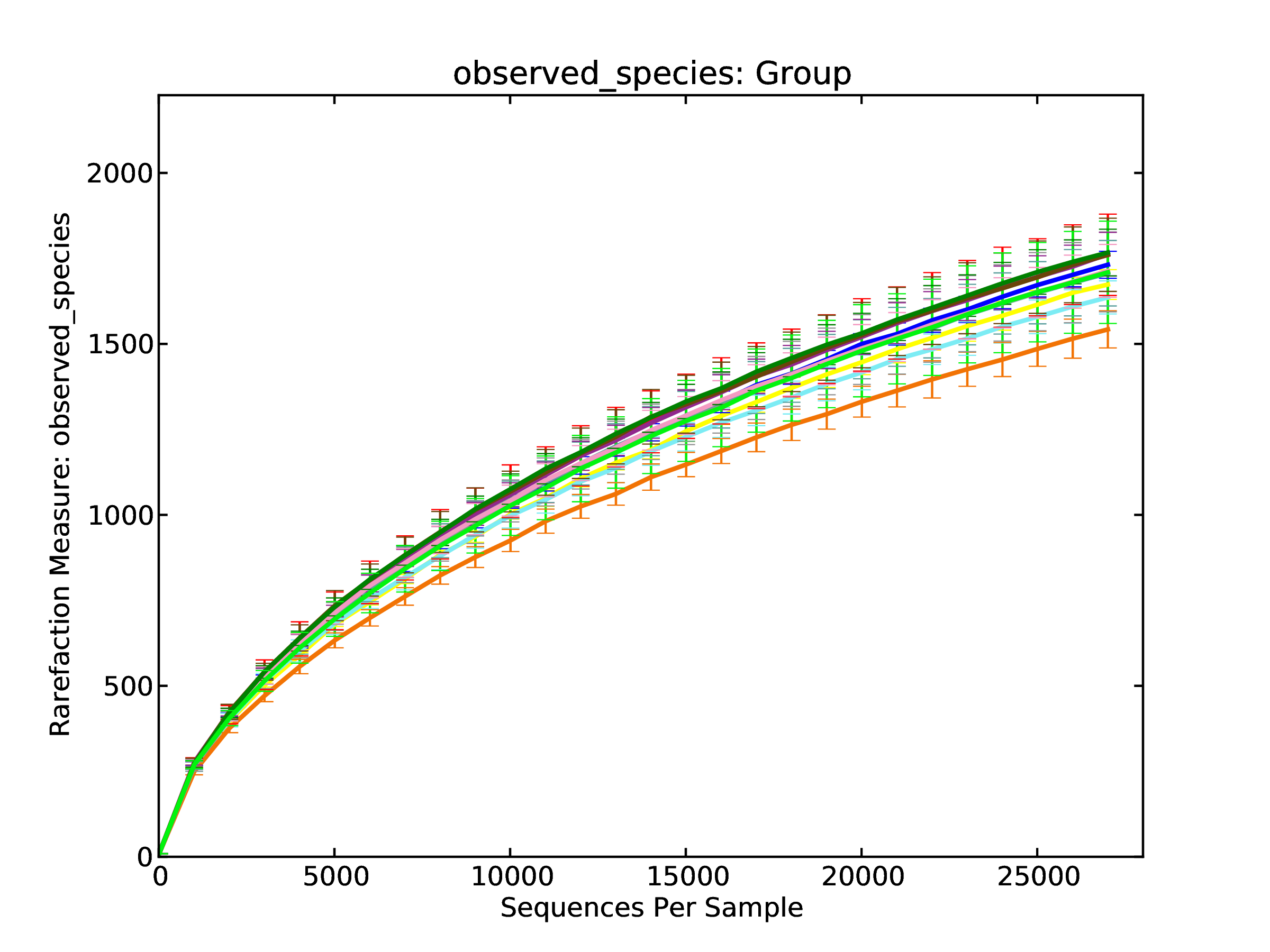

Supplement: Supplementary file 4 — Supplementary Data 1 [file 42003_2023_5520_MOESM4_ESM.zip › 4.Alpha_Diversity/alpha_rarefaction_plot/rarefaction_plots_pdf_depth27686/average_plots/observed_speciesGroup.png]

observed\_species: LinkerPrimerSequence

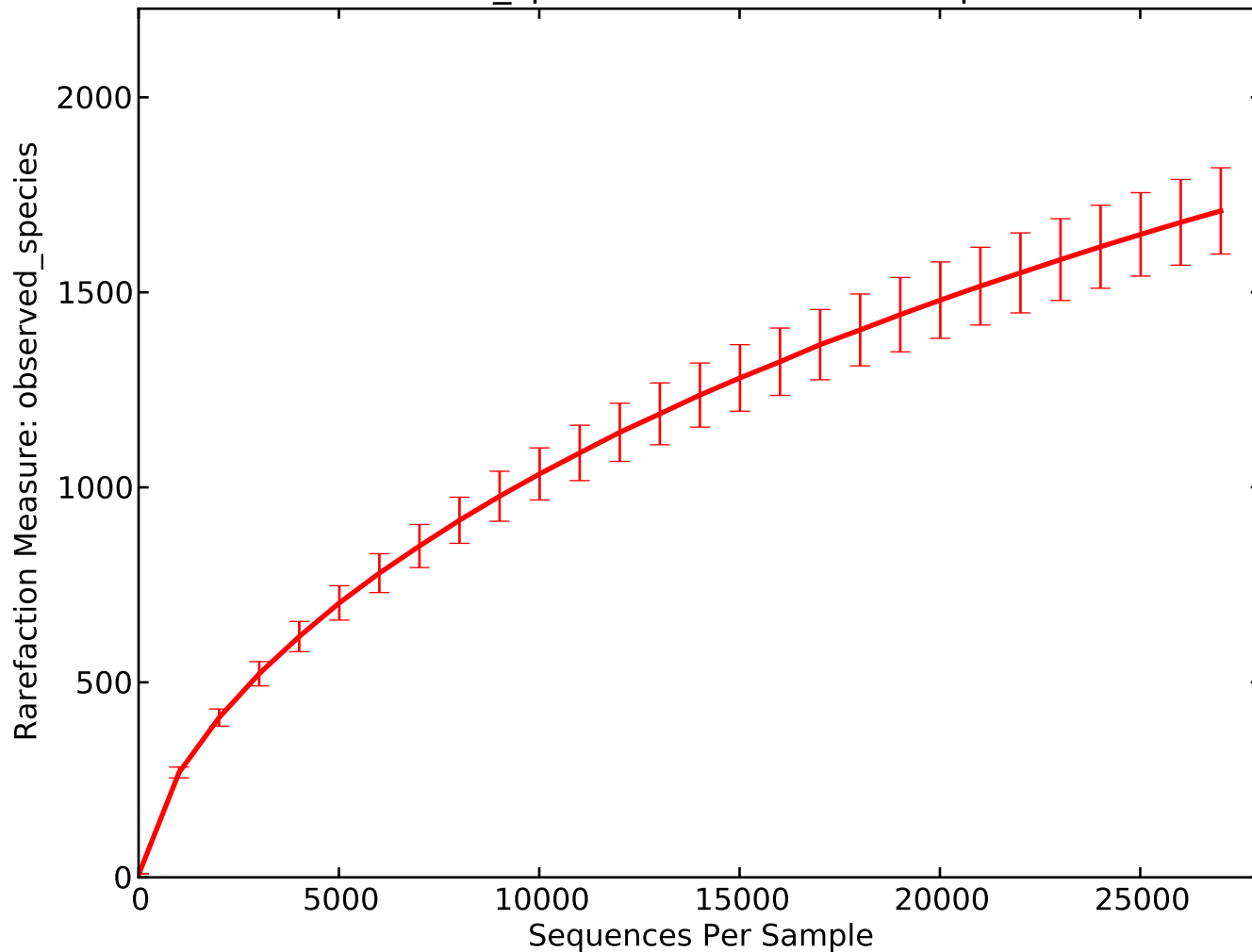

Supplement: Supplementary file 4 — Supplementary Data 1 [file 42003_2023_5520_MOESM4_ESM.zip › 4.Alpha_Diversity/alpha_rarefaction_plot/rarefaction_plots_pdf_depth27686/average_plots/observed_speciesLinkerPrimerSequence.pdf]

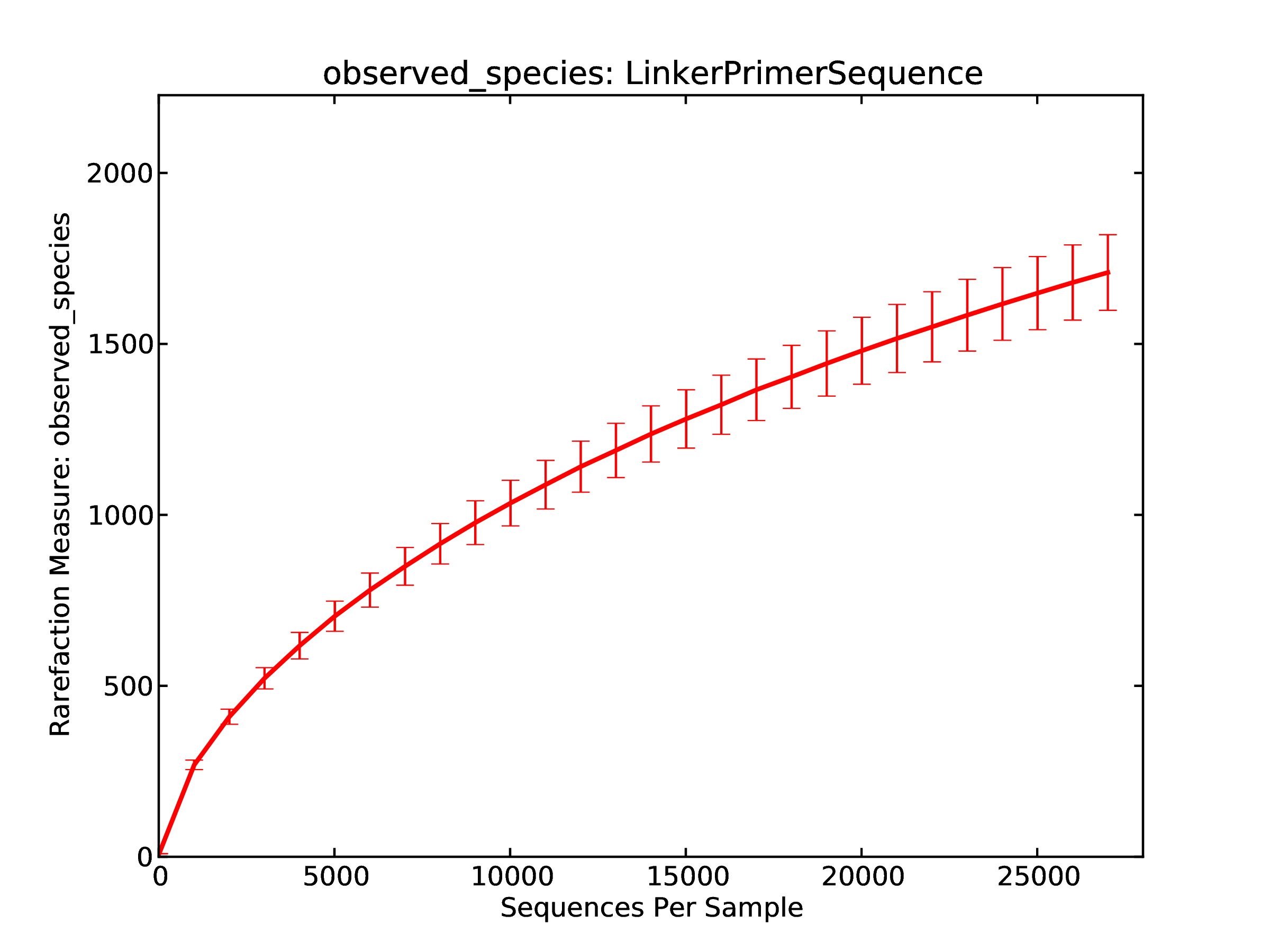

Supplement: Supplementary file 4 — Supplementary Data 1 [file 42003_2023_5520_MOESM4_ESM.zip › 4.Alpha_Diversity/alpha_rarefaction_plot/rarefaction_plots_pdf_depth27686/average_plots/observed_speciesLinkerPrimerSequence.png]

observed\_species: SampleID

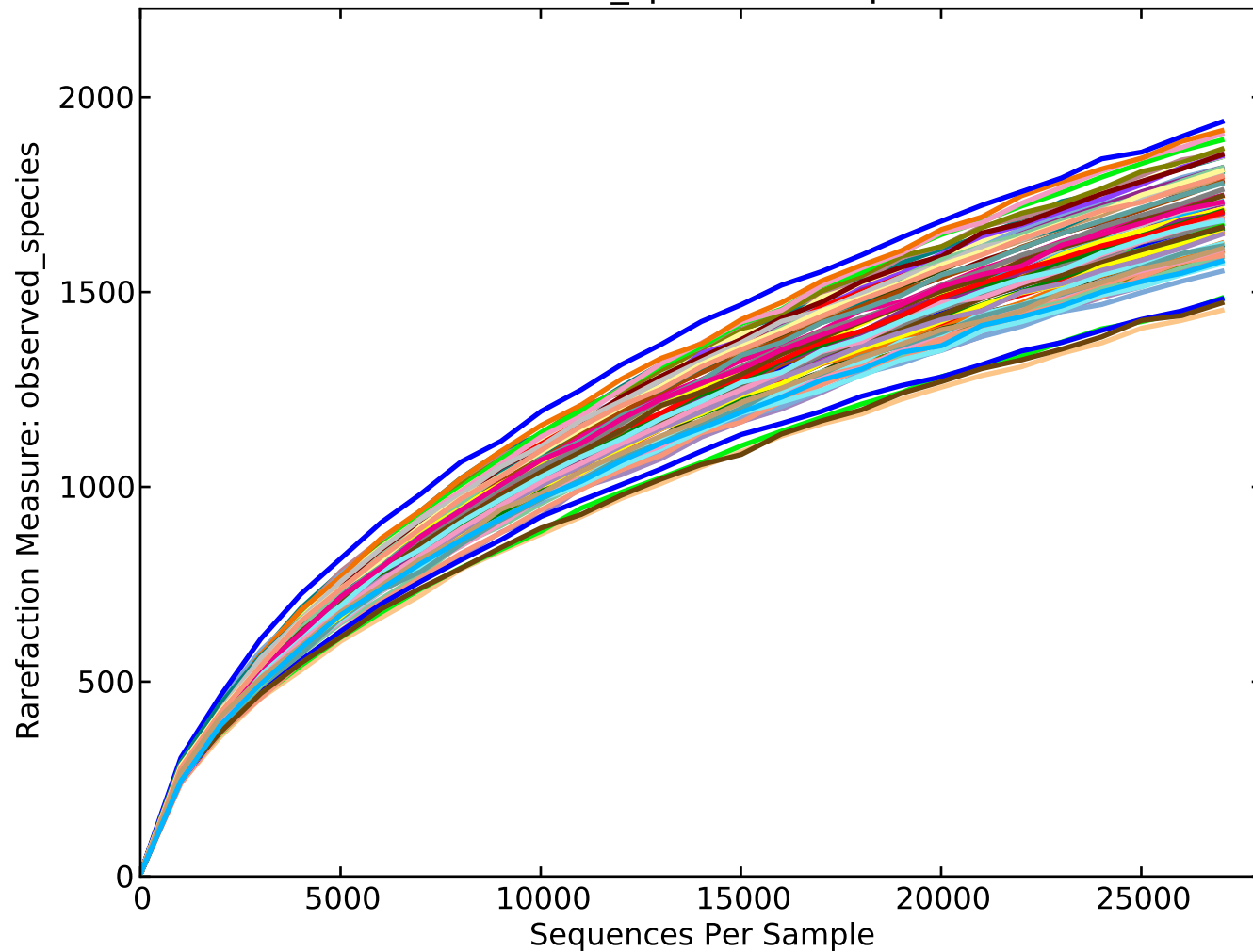

Supplement: Supplementary file 4 — Supplementary Data 1 [file 42003_2023_5520_MOESM4_ESM.zip › 4.Alpha_Diversity/alpha_rarefaction_plot/rarefaction_plots_pdf_depth27686/average_plots/observed_speciesSampleID.pdf]

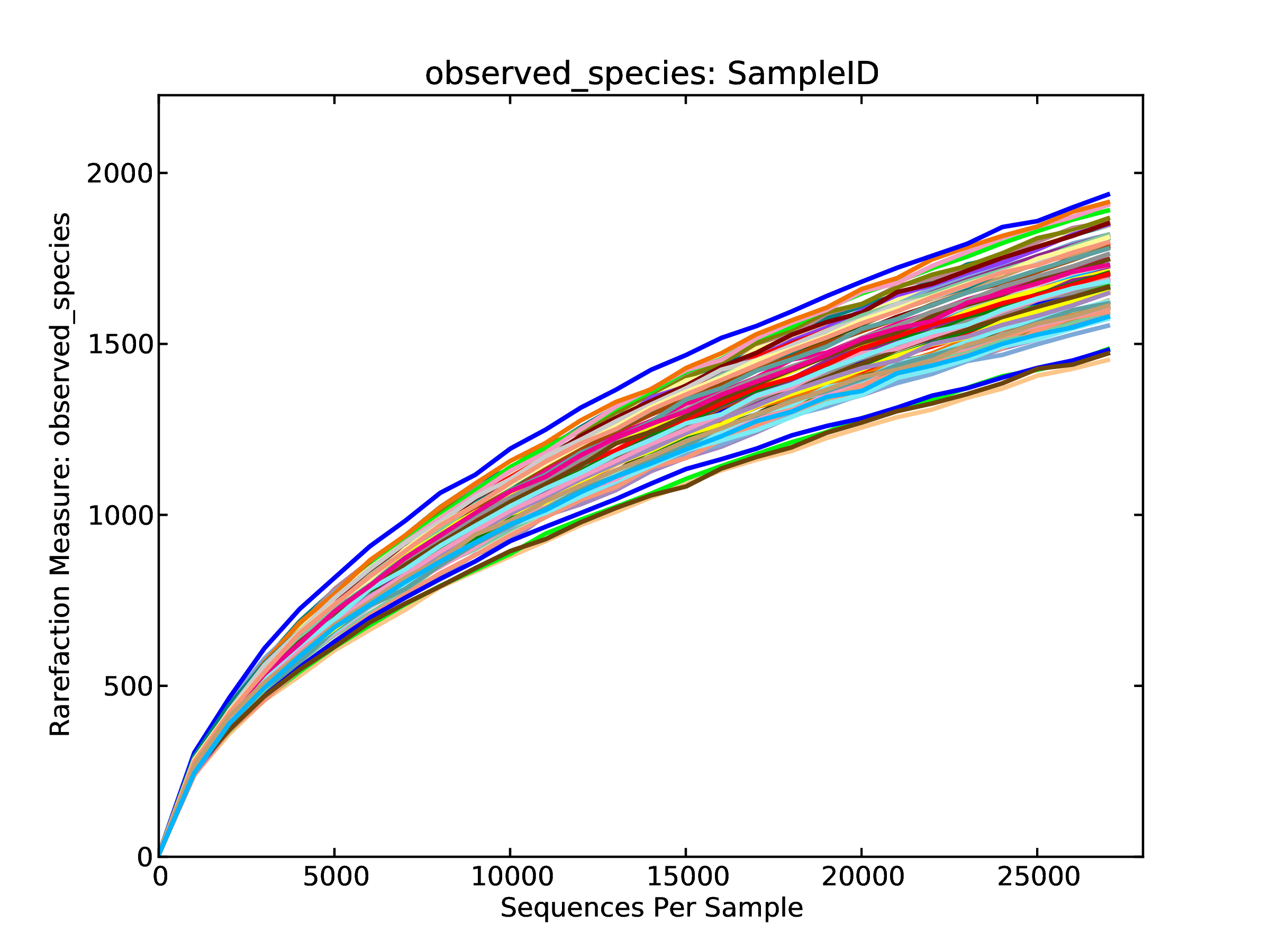

Supplement: Supplementary file 4 — Supplementary Data 1 [file 42003_2023_5520_MOESM4_ESM.zip › 4.Alpha_Diversity/alpha_rarefaction_plot/rarefaction_plots_pdf_depth27686/average_plots/observed_speciesSampleID.png]

shannon: BarcodeSequence

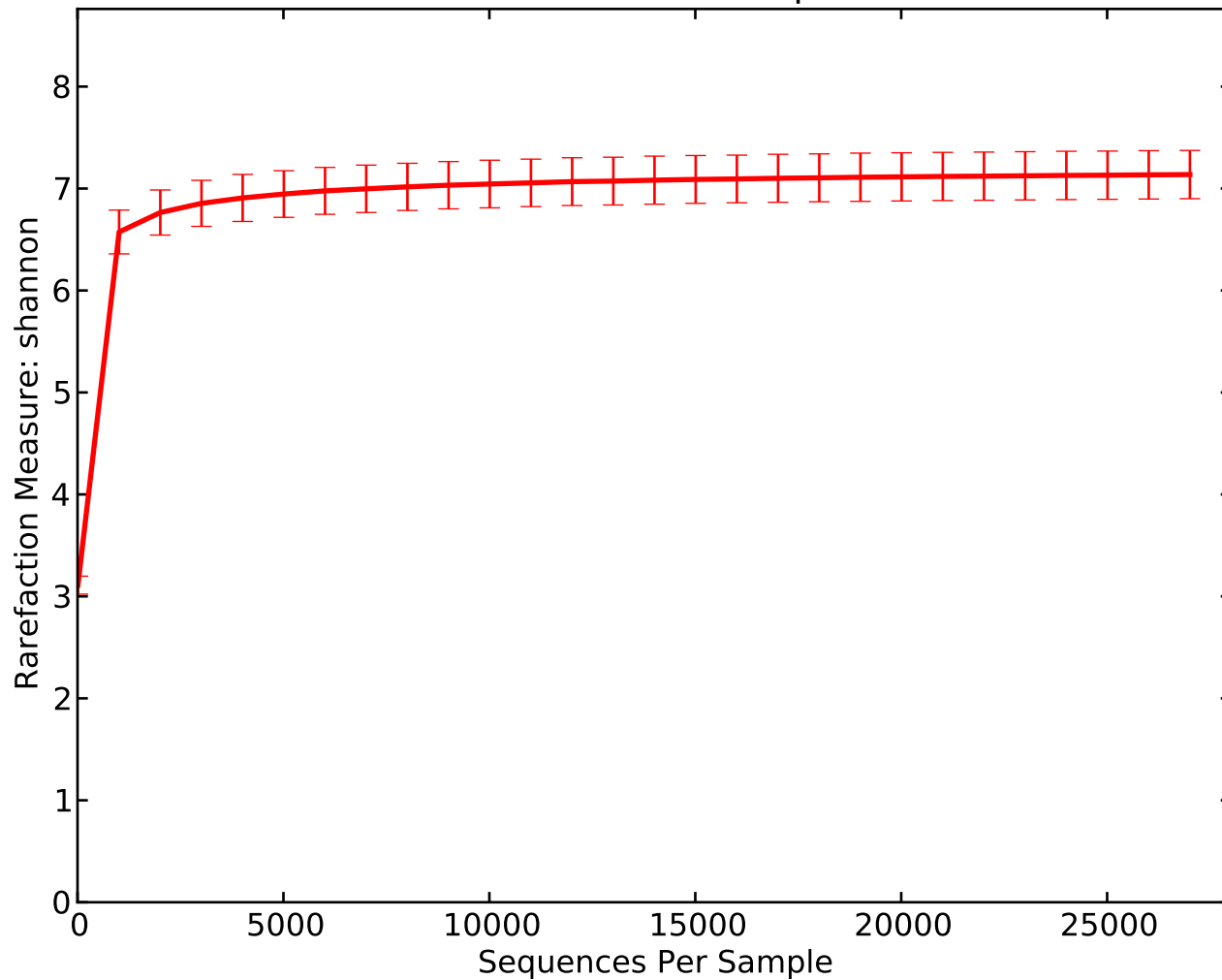

Supplement: Supplementary file 4 — Supplementary Data 1 [file 42003_2023_5520_MOESM4_ESM.zip › 4.Alpha_Diversity/alpha_rarefaction_plot/rarefaction_plots_pdf_depth27686/average_plots/shannonBarcodeSequence.pdf]

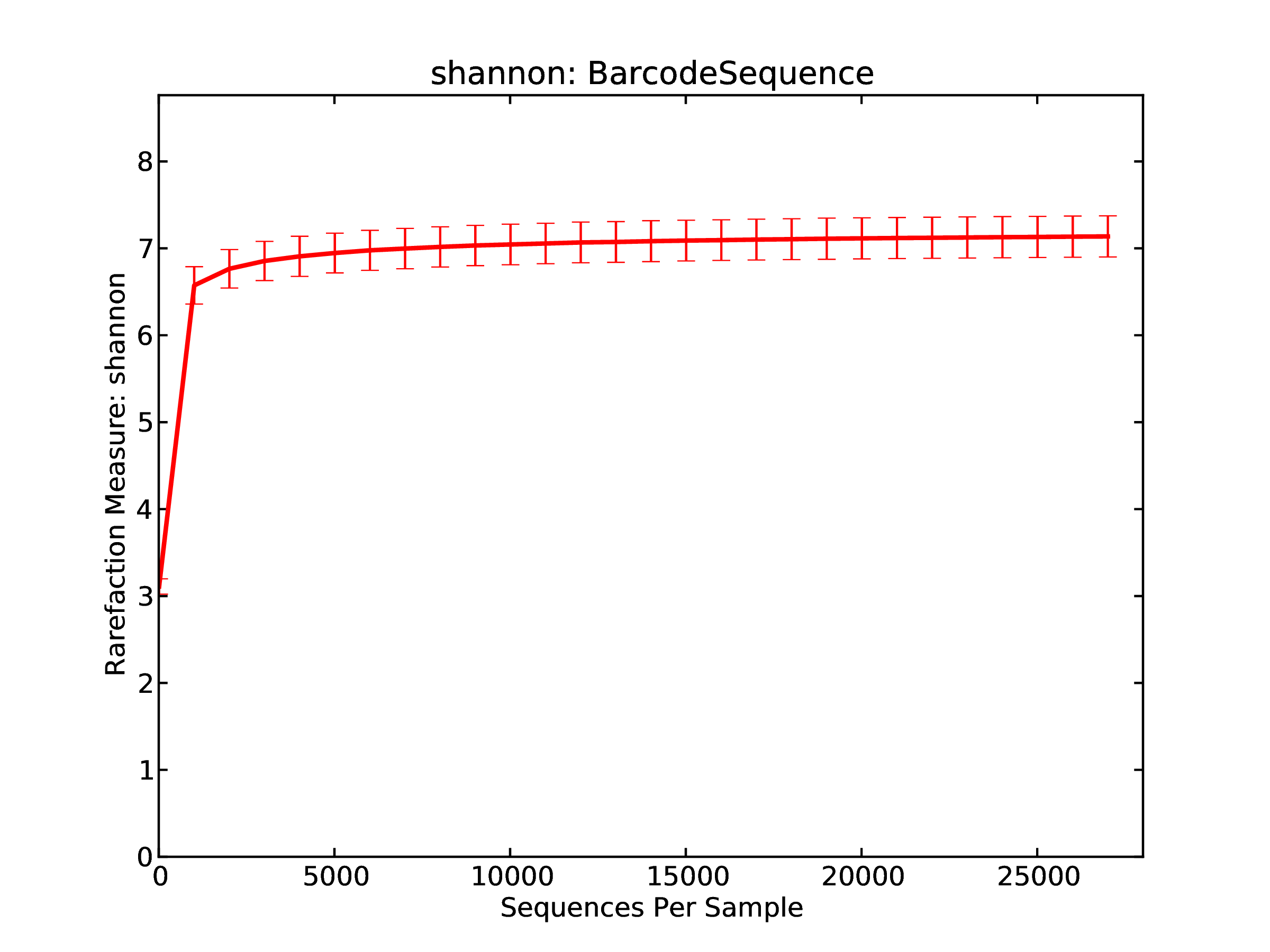

Supplement: Supplementary file 4 — Supplementary Data 1 [file 42003_2023_5520_MOESM4_ESM.zip › 4.Alpha_Diversity/alpha_rarefaction_plot/rarefaction_plots_pdf_depth27686/average_plots/shannonBarcodeSequence.png]

shannon: Description

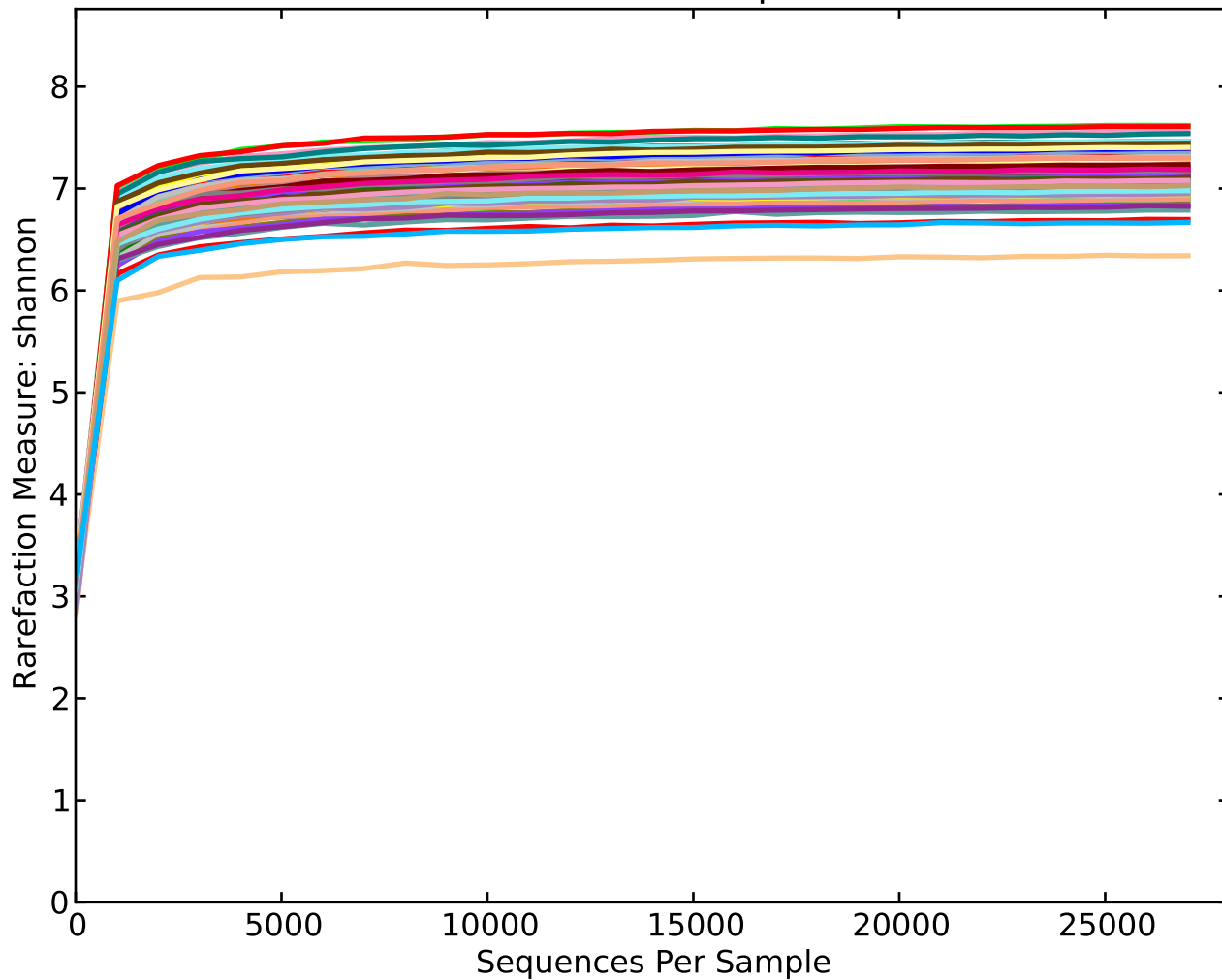

Supplement: Supplementary file 4 — Supplementary Data 1 [file 42003_2023_5520_MOESM4_ESM.zip › 4.Alpha_Diversity/alpha_rarefaction_plot/rarefaction_plots_pdf_depth27686/average_plots/shannonDescription.pdf]

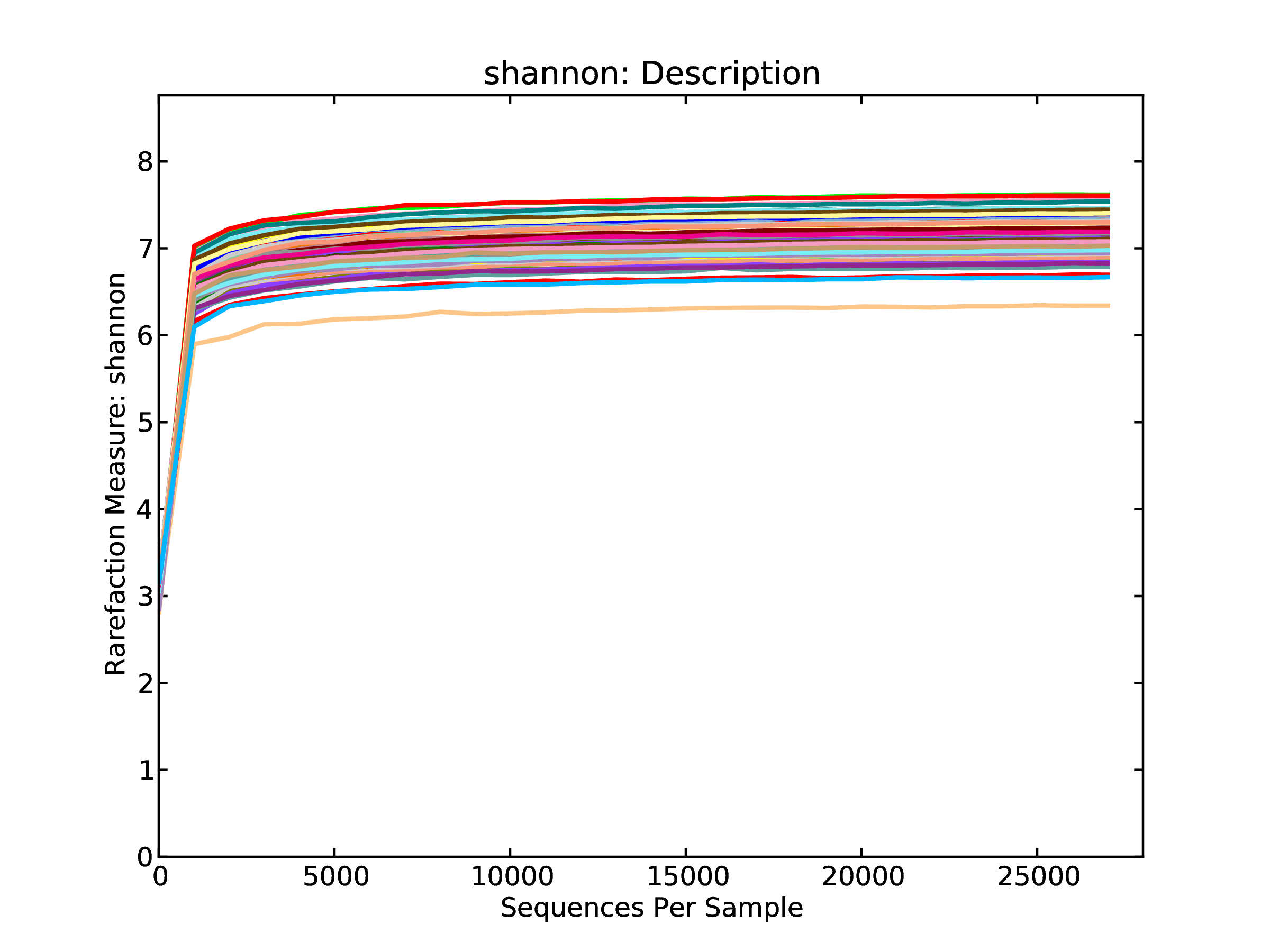

Supplement: Supplementary file 4 — Supplementary Data 1 [file 42003_2023_5520_MOESM4_ESM.zip › 4.Alpha_Diversity/alpha_rarefaction_plot/rarefaction_plots_pdf_depth27686/average_plots/shannonDescription.png]

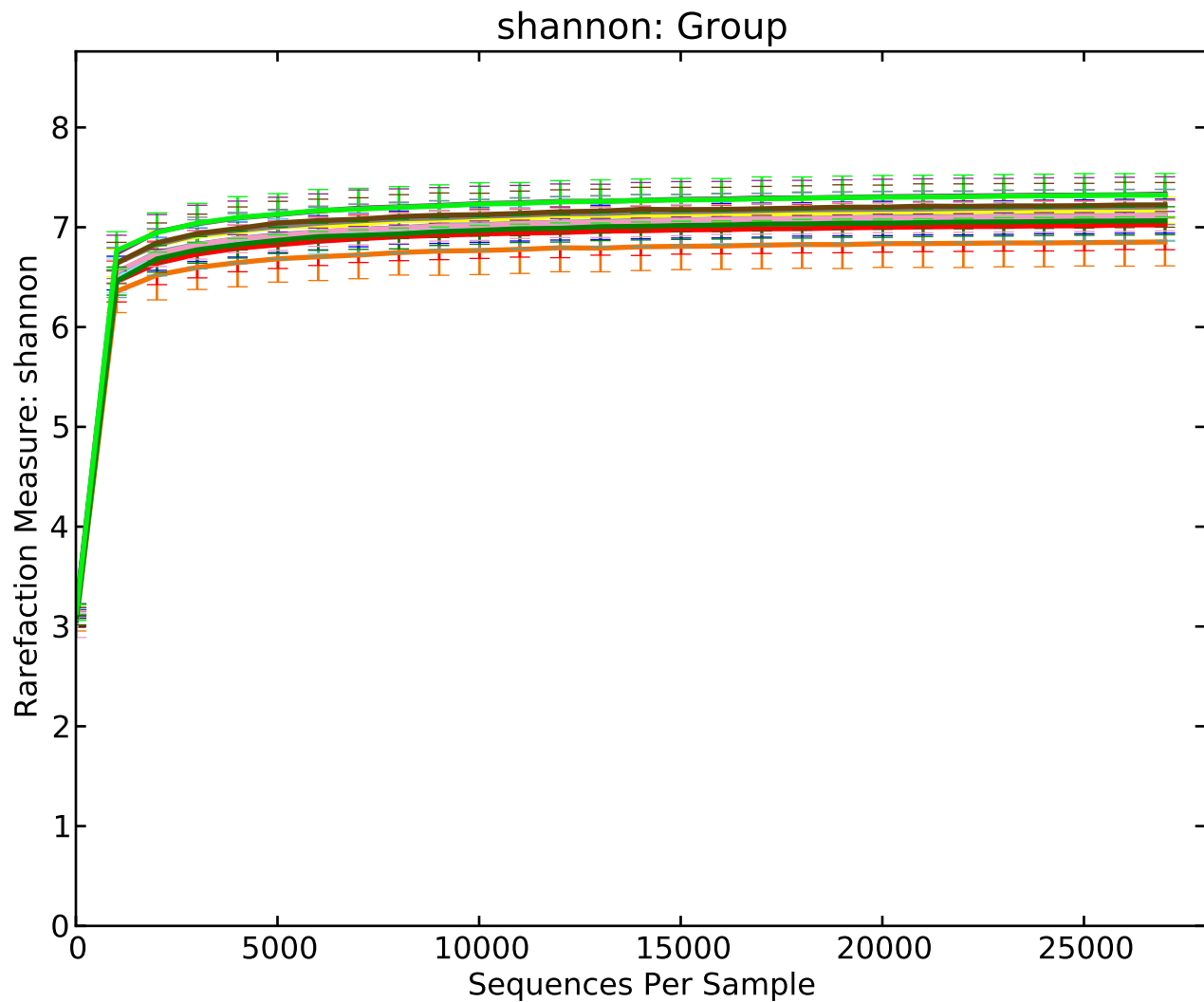

Supplement: Supplementary file 4 — Supplementary Data 1 [file 42003_2023_5520_MOESM4_ESM.zip › 4.Alpha_Diversity/alpha_rarefaction_plot/rarefaction_plots_pdf_depth27686/average_plots/shannonGroup.pdf]

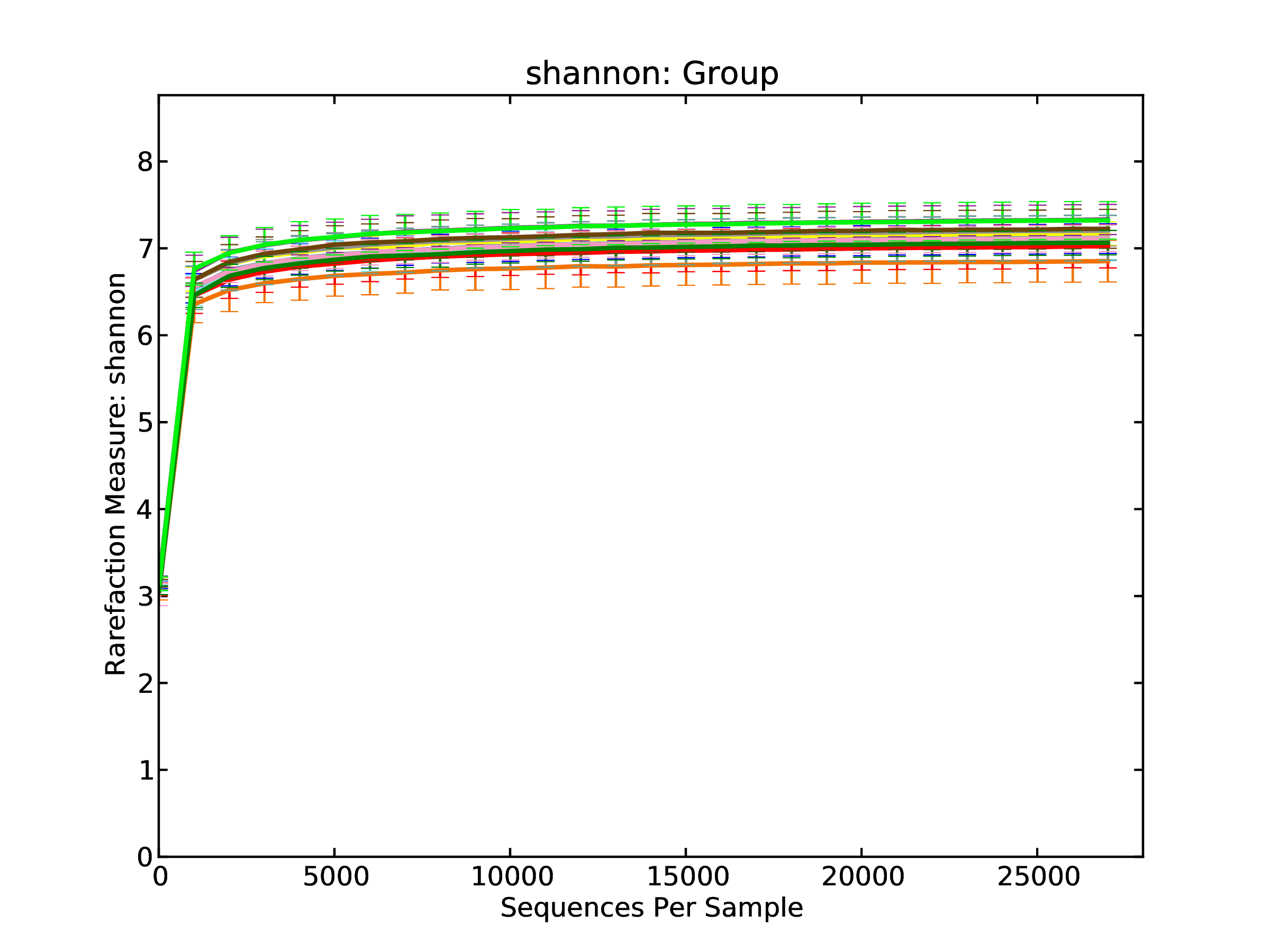

Supplement: Supplementary file 4 — Supplementary Data 1 [file 42003_2023_5520_MOESM4_ESM.zip › 4.Alpha_Diversity/alpha_rarefaction_plot/rarefaction_plots_pdf_depth27686/average_plots/shannonGroup.png]

shannon: LinkerPrimerSequence

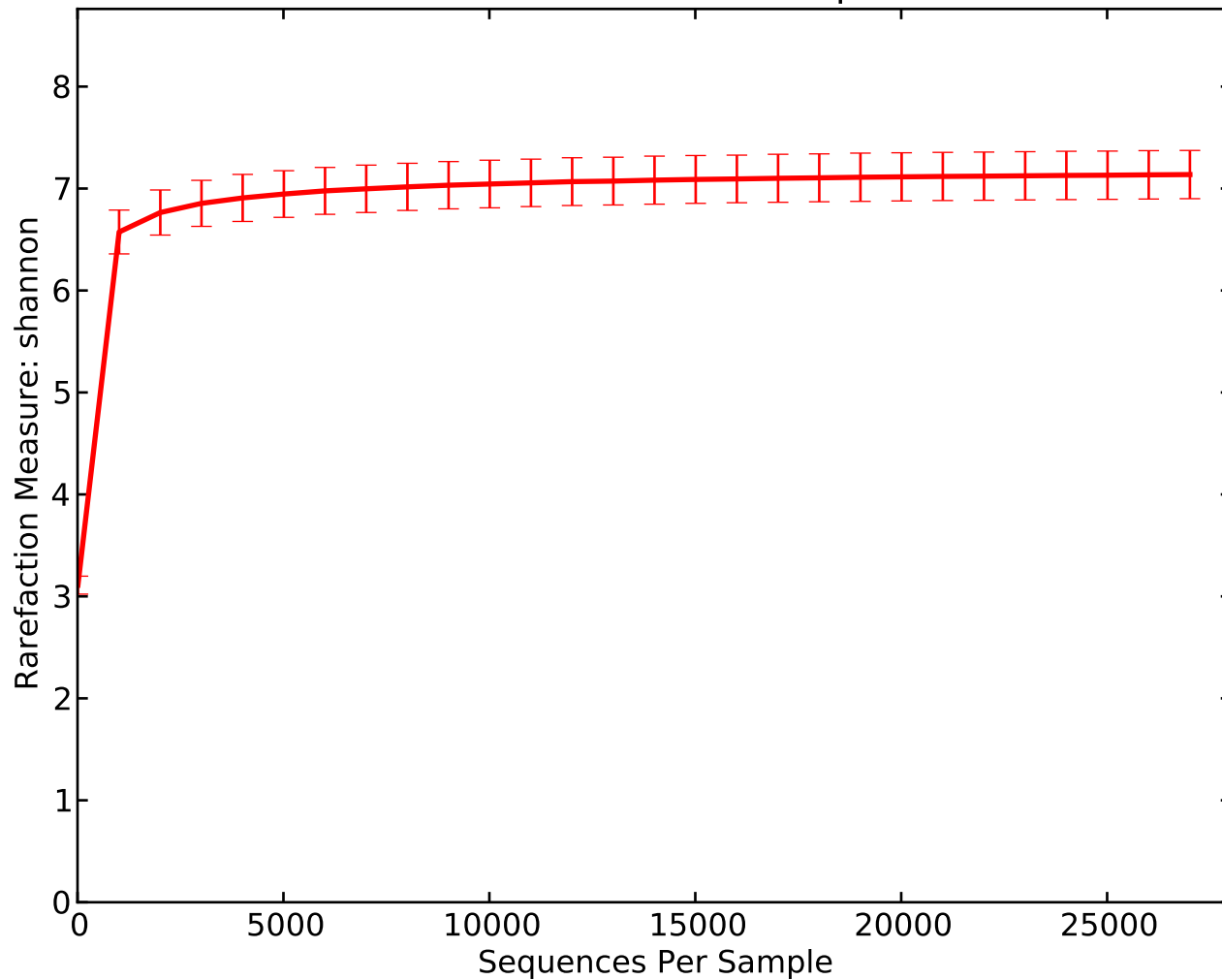

Supplement: Supplementary file 4 — Supplementary Data 1 [file 42003_2023_5520_MOESM4_ESM.zip › 4.Alpha_Diversity/alpha_rarefaction_plot/rarefaction_plots_pdf_depth27686/average_plots/shannonLinkerPrimerSequence.pdf]

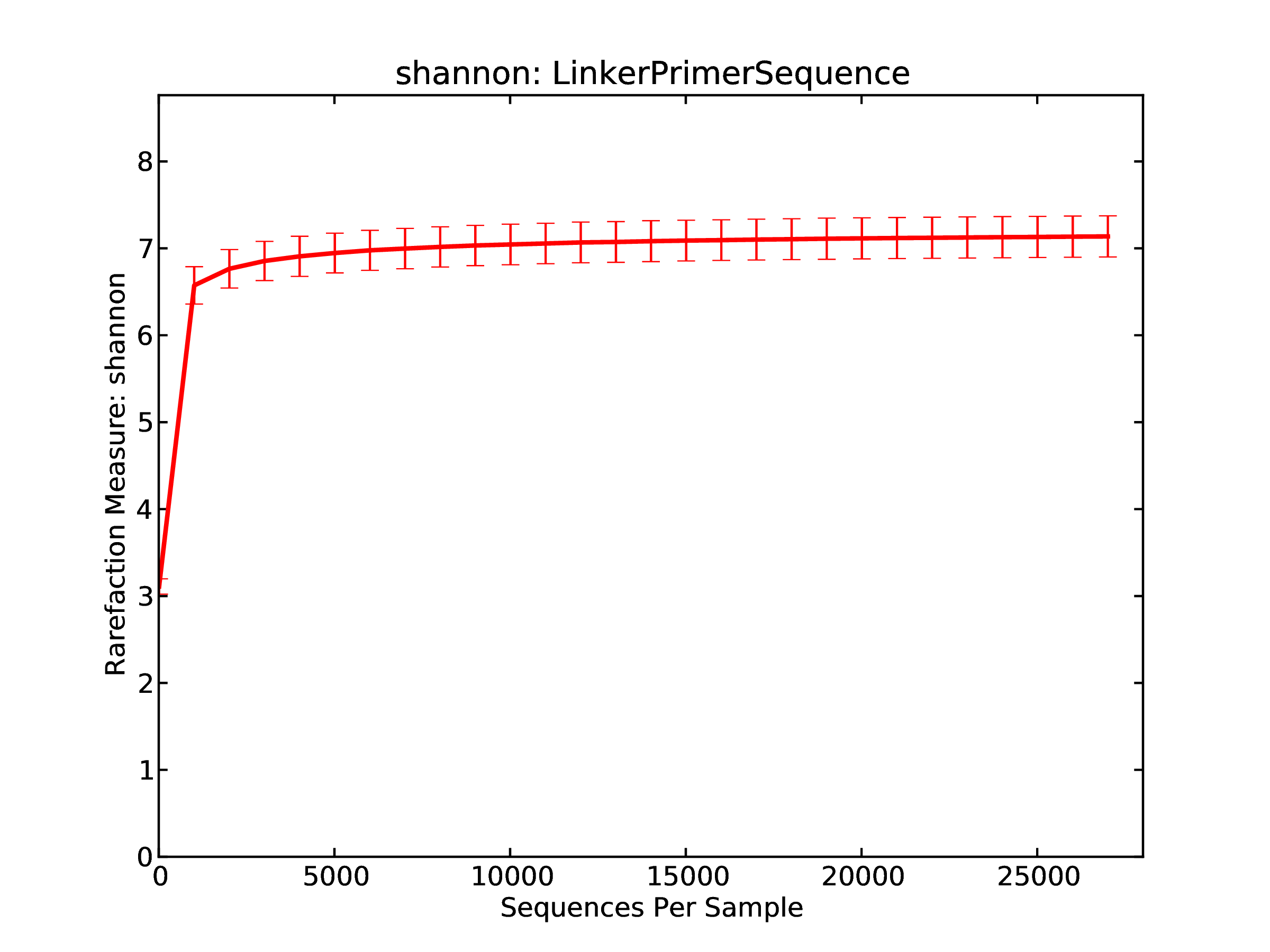

Supplement: Supplementary file 4 — Supplementary Data 1 [file 42003_2023_5520_MOESM4_ESM.zip › 4.Alpha_Diversity/alpha_rarefaction_plot/rarefaction_plots_pdf_depth27686/average_plots/shannonLinkerPrimerSequence.png]

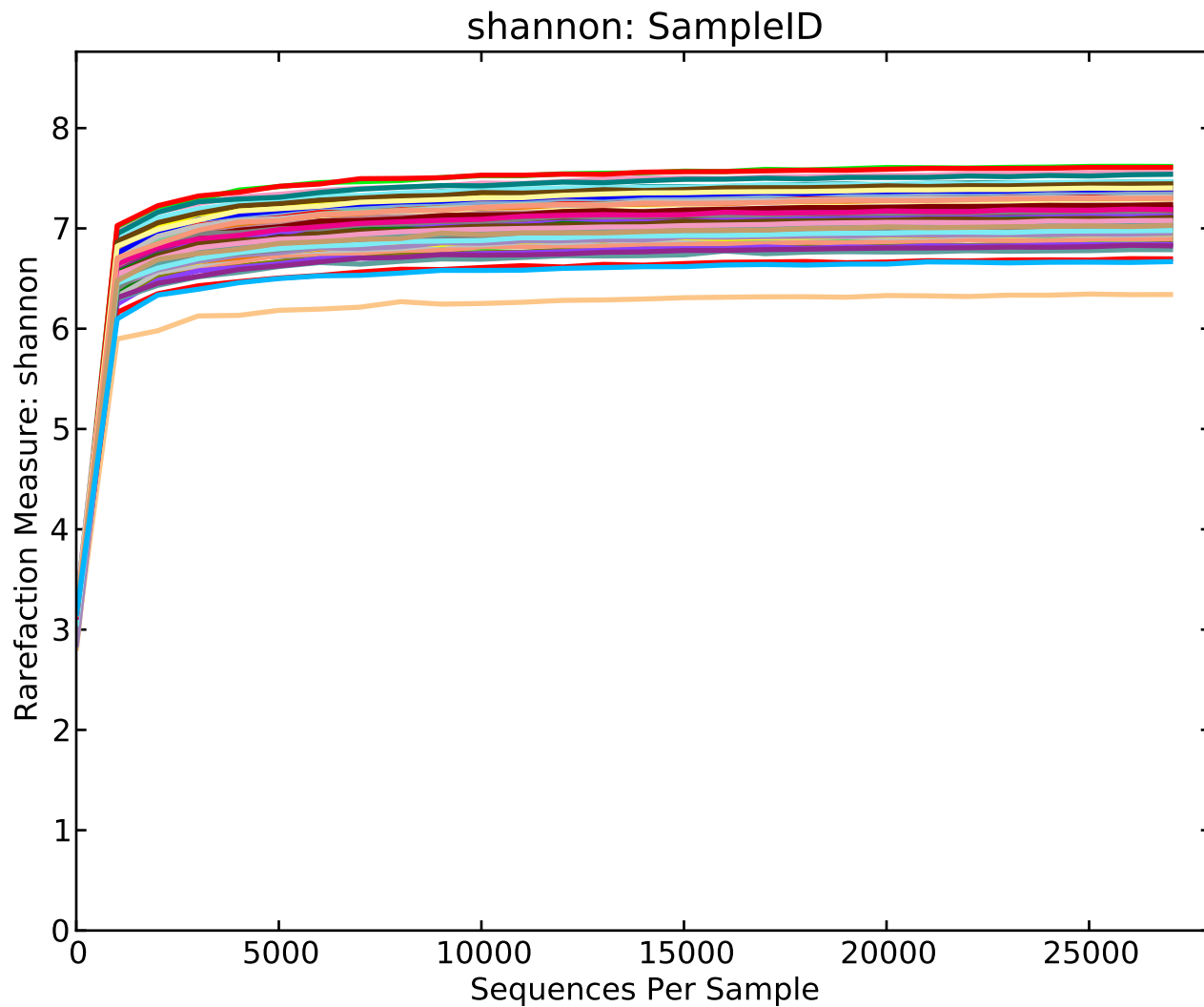

Supplement: Supplementary file 4 — Supplementary Data 1 [file 42003_2023_5520_MOESM4_ESM.zip › 4.Alpha_Diversity/alpha_rarefaction_plot/rarefaction_plots_pdf_depth27686/average_plots/shannonSampleID.pdf]

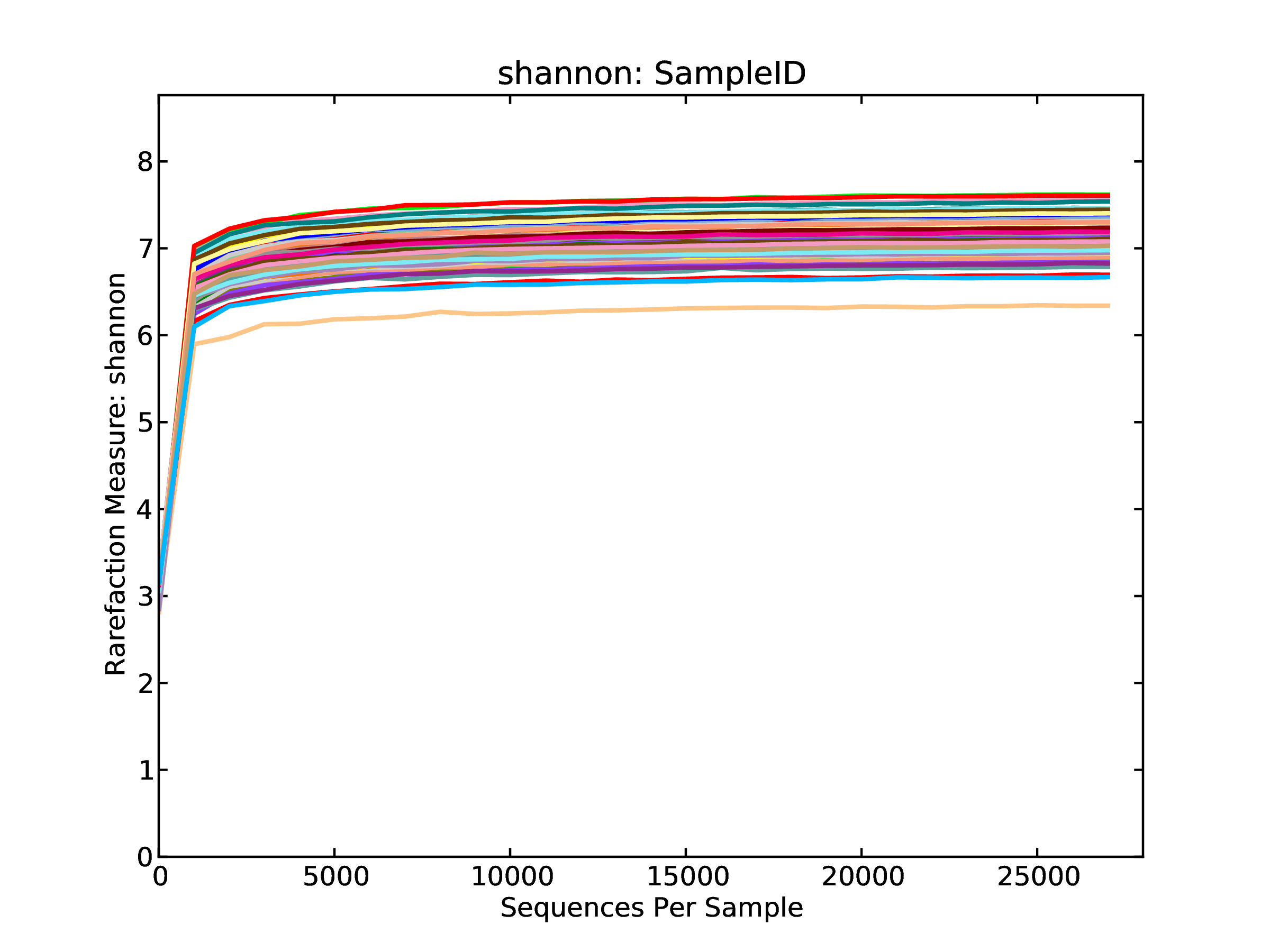

Supplement: Supplementary file 4 — Supplementary Data 1 [file 42003_2023_5520_MOESM4_ESM.zip › 4.Alpha_Diversity/alpha_rarefaction_plot/rarefaction_plots_pdf_depth27686/average_plots/shannonSampleID.png]

simpson: BarcodeSequence

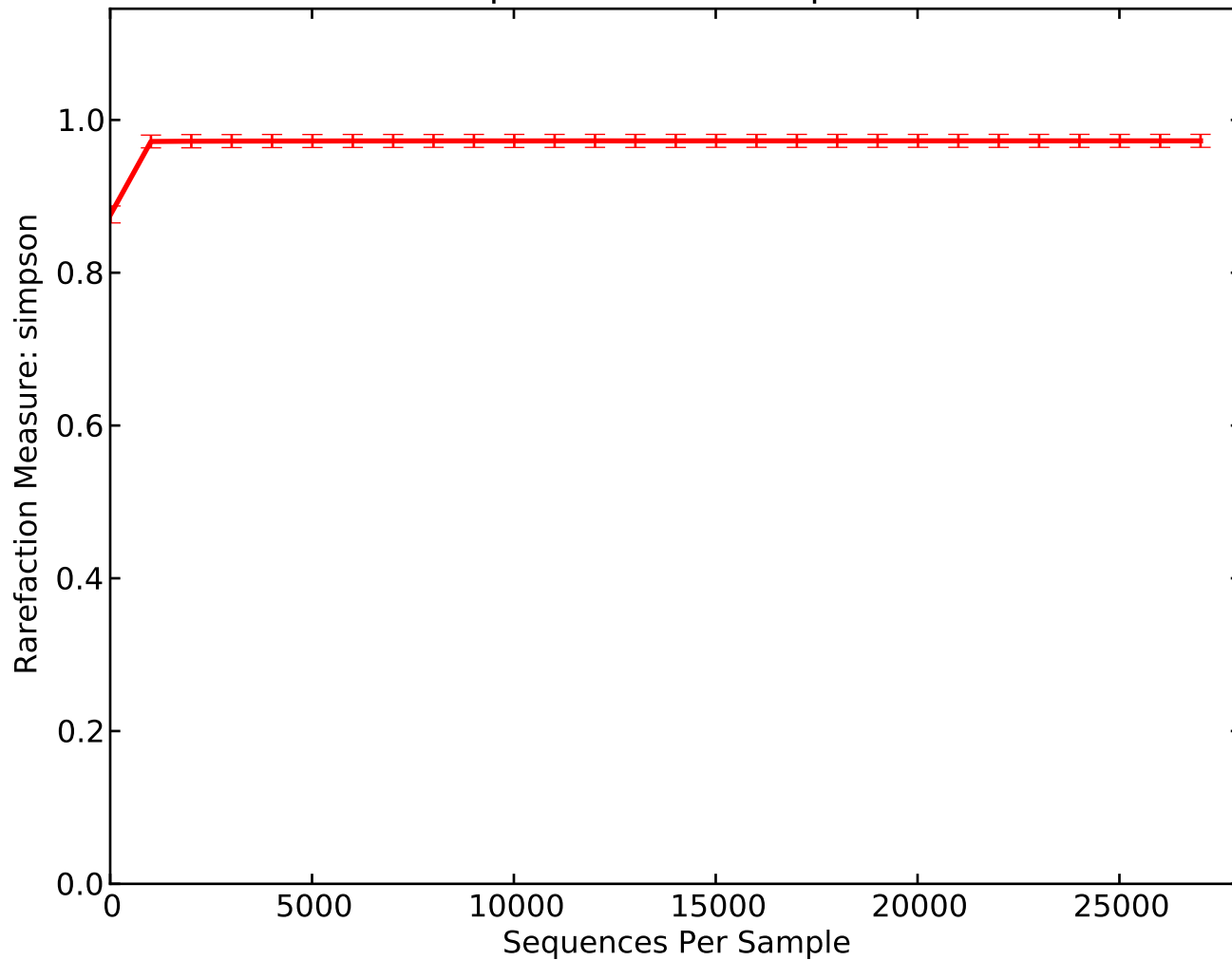

Supplement: Supplementary file 4 — Supplementary Data 1 [file 42003_2023_5520_MOESM4_ESM.zip › 4.Alpha_Diversity/alpha_rarefaction_plot/rarefaction_plots_pdf_depth27686/average_plots/simpsonBarcodeSequence.pdf]

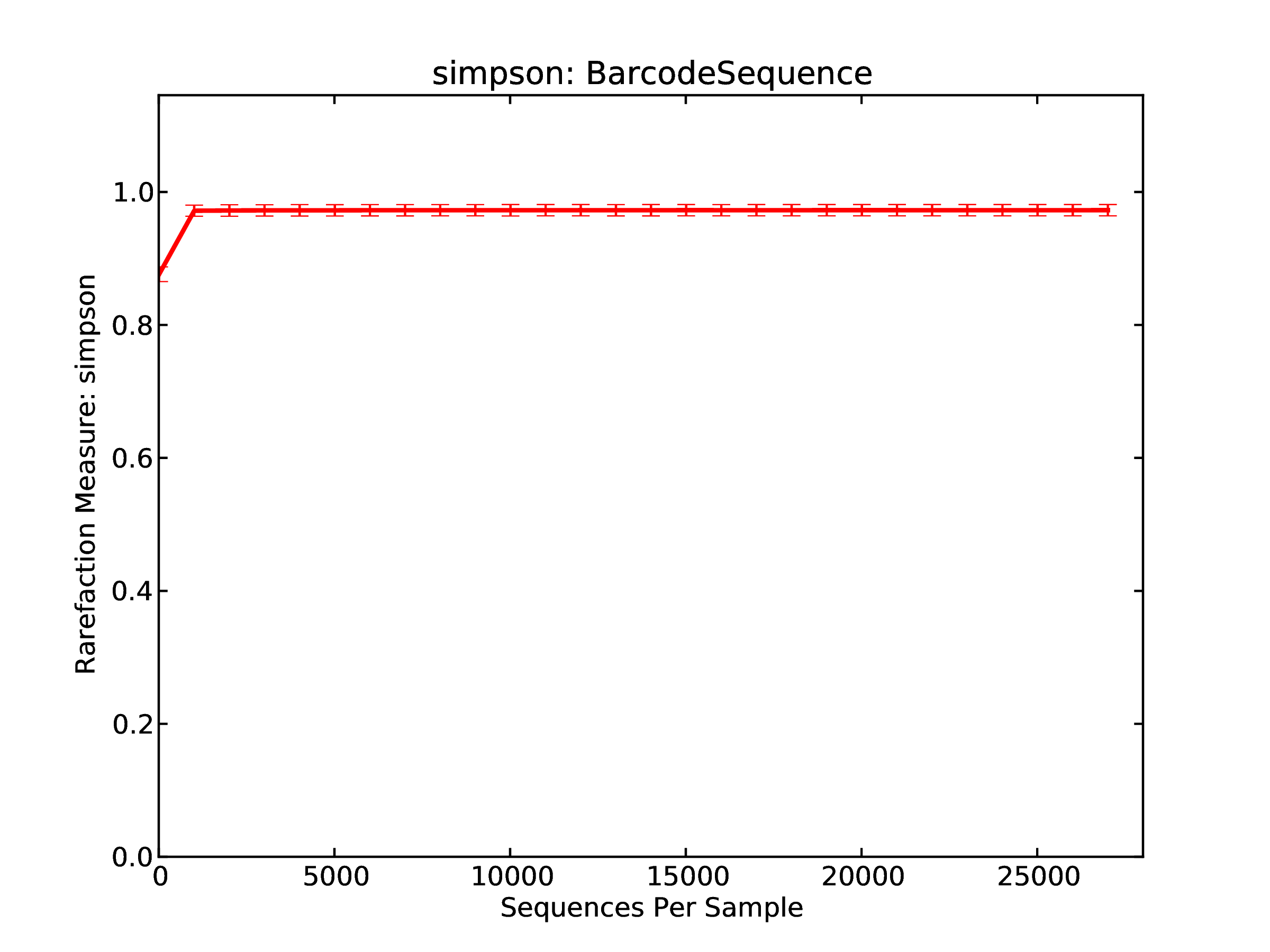

Supplement: Supplementary file 4 — Supplementary Data 1 [file 42003_2023_5520_MOESM4_ESM.zip › 4.Alpha_Diversity/alpha_rarefaction_plot/rarefaction_plots_pdf_depth27686/average_plots/simpsonBarcodeSequence.png]

simpson: Description

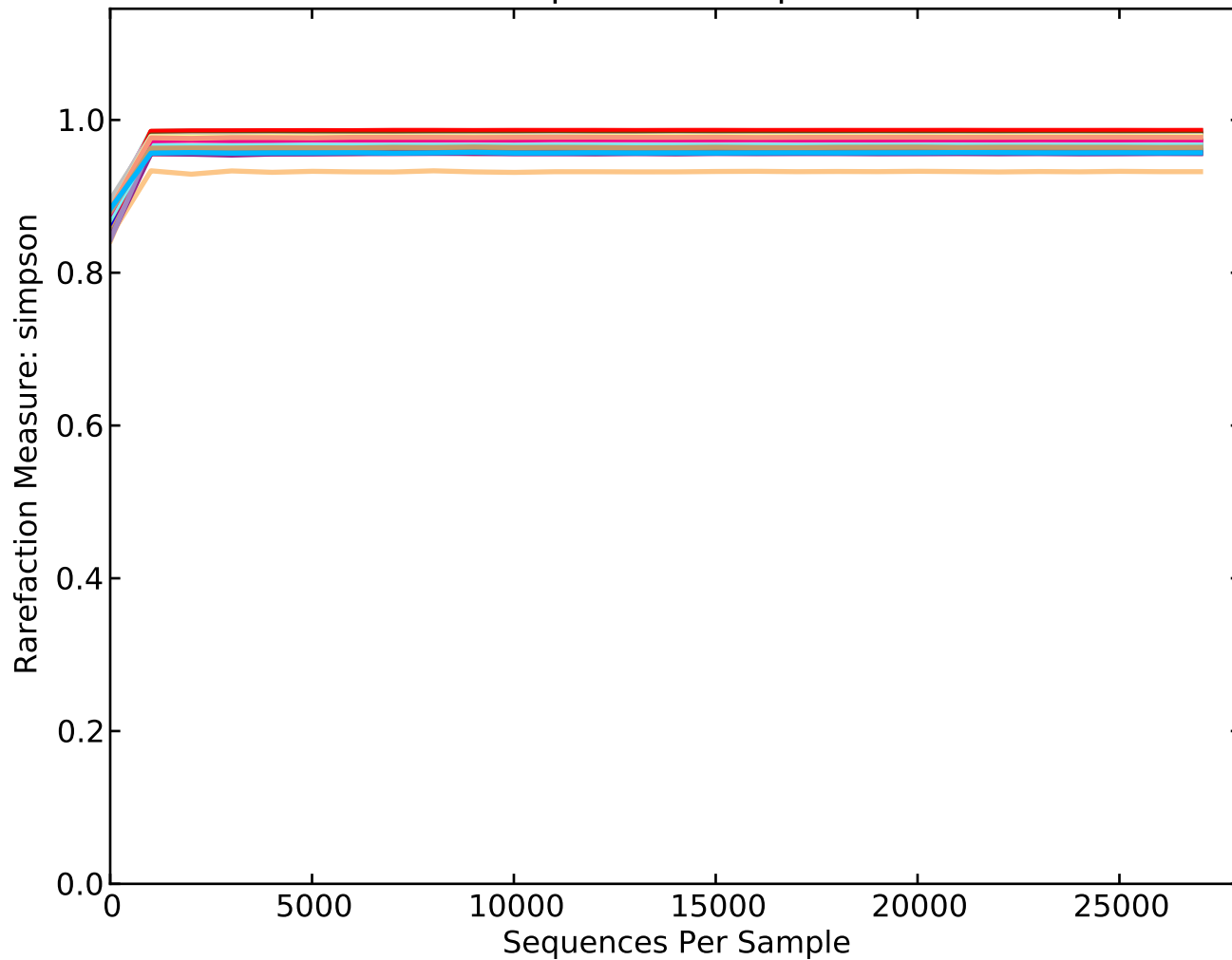

Supplement: Supplementary file 4 — Supplementary Data 1 [file 42003_2023_5520_MOESM4_ESM.zip › 4.Alpha_Diversity/alpha_rarefaction_plot/rarefaction_plots_pdf_depth27686/average_plots/simpsonDescription.pdf]

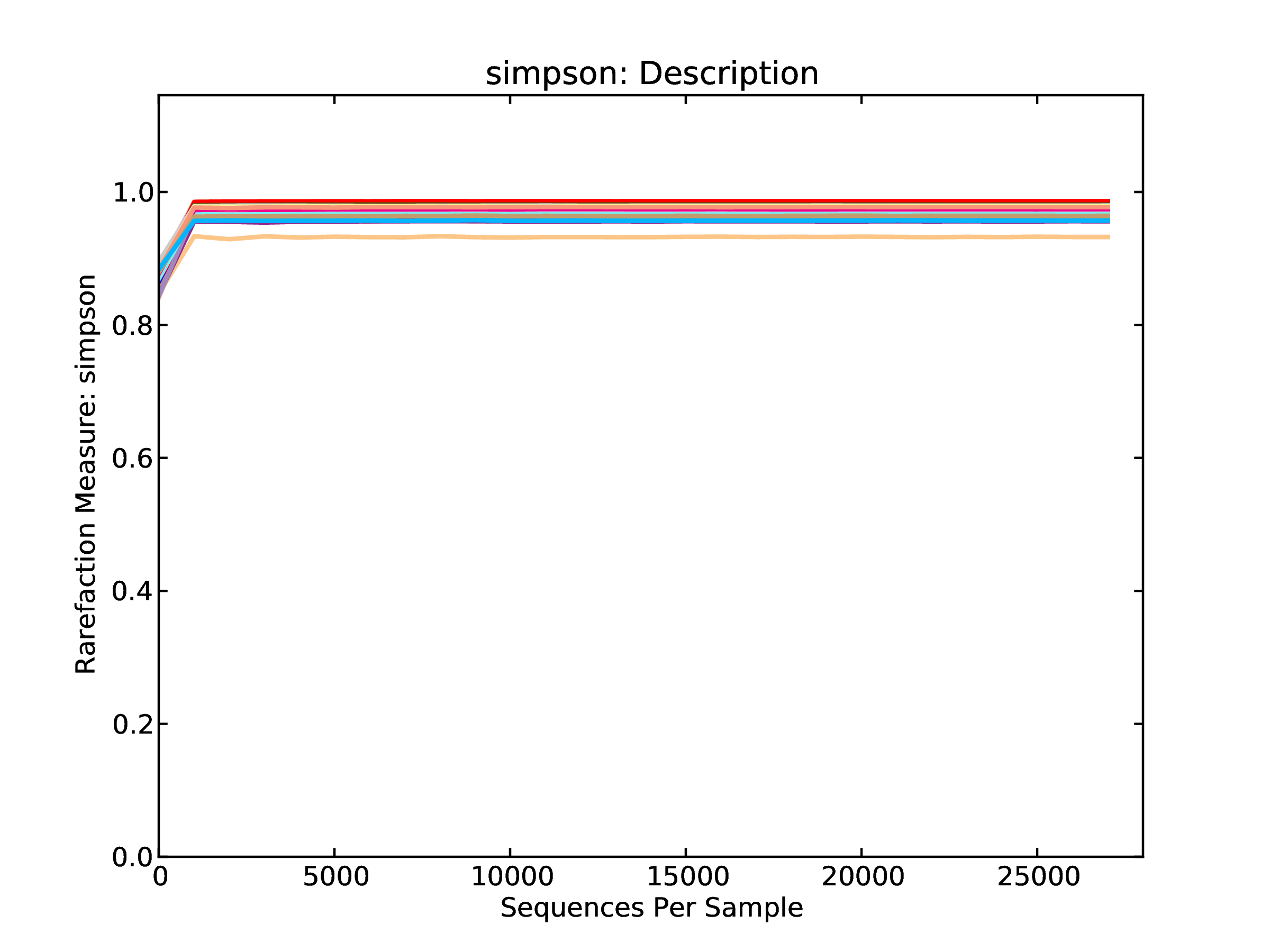

Supplement: Supplementary file 4 — Supplementary Data 1 [file 42003_2023_5520_MOESM4_ESM.zip › 4.Alpha_Diversity/alpha_rarefaction_plot/rarefaction_plots_pdf_depth27686/average_plots/simpsonDescription.png]

simpson: Group

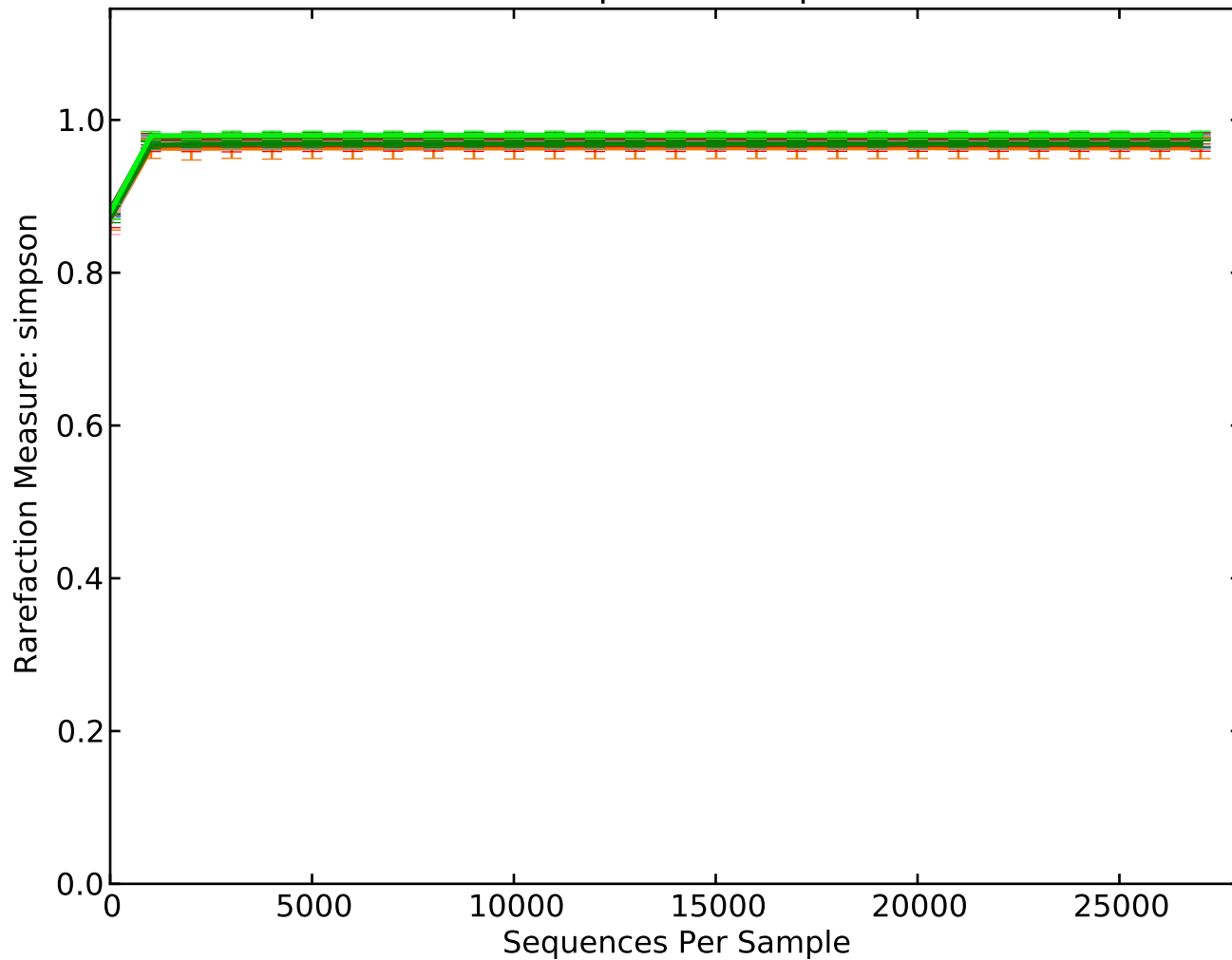

Supplement: Supplementary file 4 — Supplementary Data 1 [file 42003_2023_5520_MOESM4_ESM.zip › 4.Alpha_Diversity/alpha_rarefaction_plot/rarefaction_plots_pdf_depth27686/average_plots/simpsonGroup.pdf]

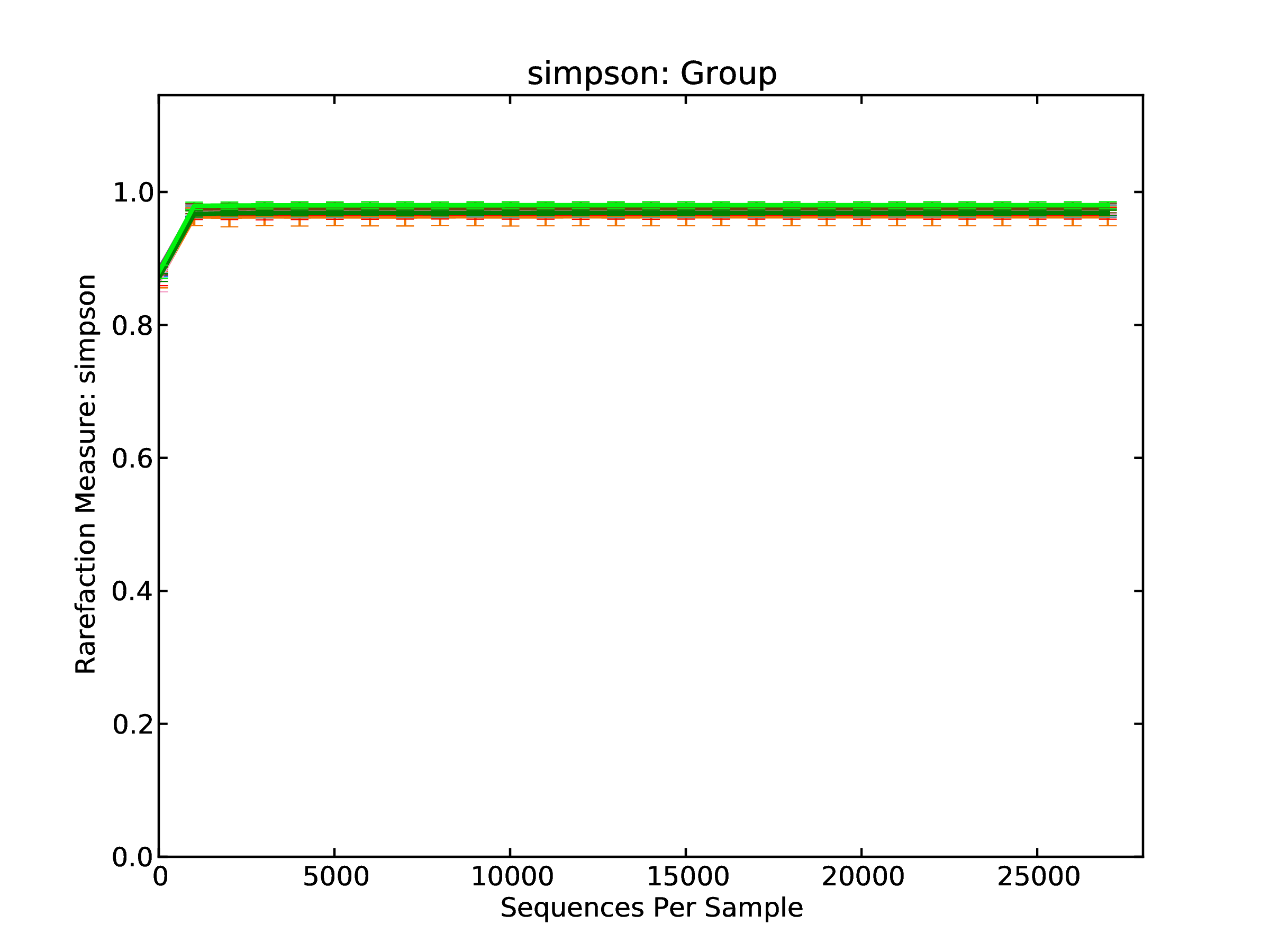

Supplement: Supplementary file 4 — Supplementary Data 1 [file 42003_2023_5520_MOESM4_ESM.zip › 4.Alpha_Diversity/alpha_rarefaction_plot/rarefaction_plots_pdf_depth27686/average_plots/simpsonGroup.png]

simpson: LinkerPrimerSequence

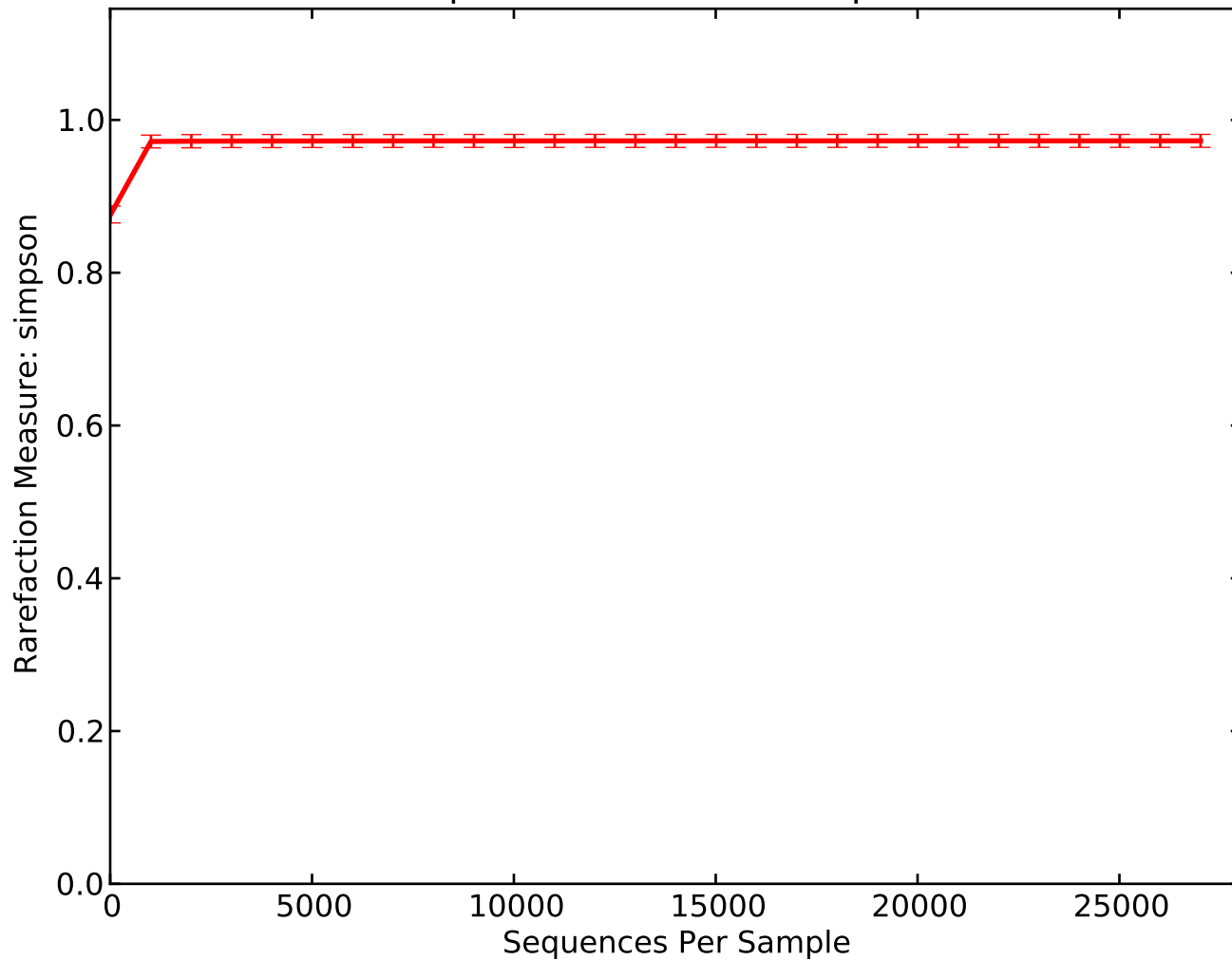

Supplement: Supplementary file 4 — Supplementary Data 1 [file 42003_2023_5520_MOESM4_ESM.zip › 4.Alpha_Diversity/alpha_rarefaction_plot/rarefaction_plots_pdf_depth27686/average_plots/simpsonLinkerPrimerSequence.pdf]

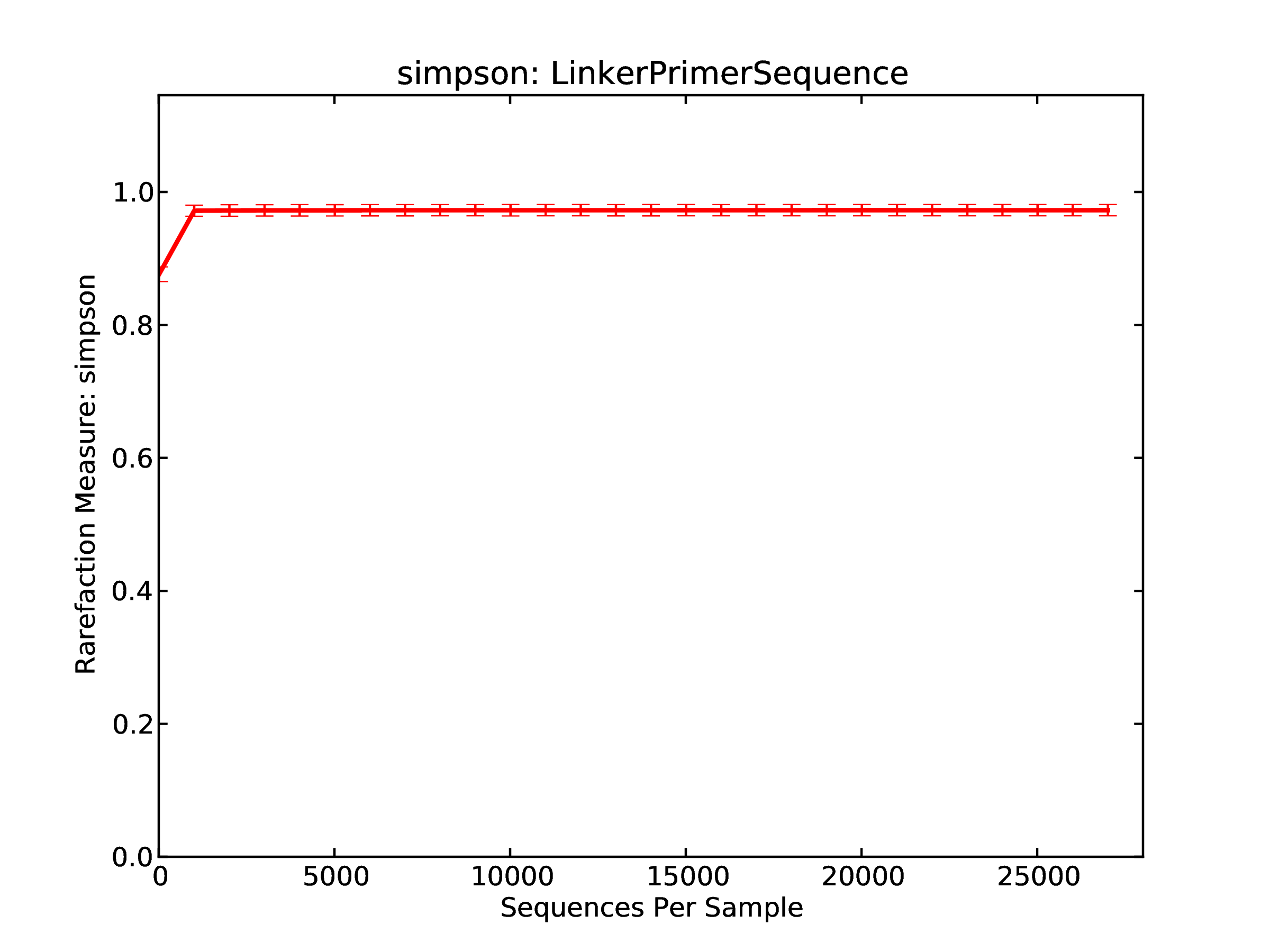

Supplement: Supplementary file 4 — Supplementary Data 1 [file 42003_2023_5520_MOESM4_ESM.zip › 4.Alpha_Diversity/alpha_rarefaction_plot/rarefaction_plots_pdf_depth27686/average_plots/simpsonLinkerPrimerSequence.png]

simpson: SampleID

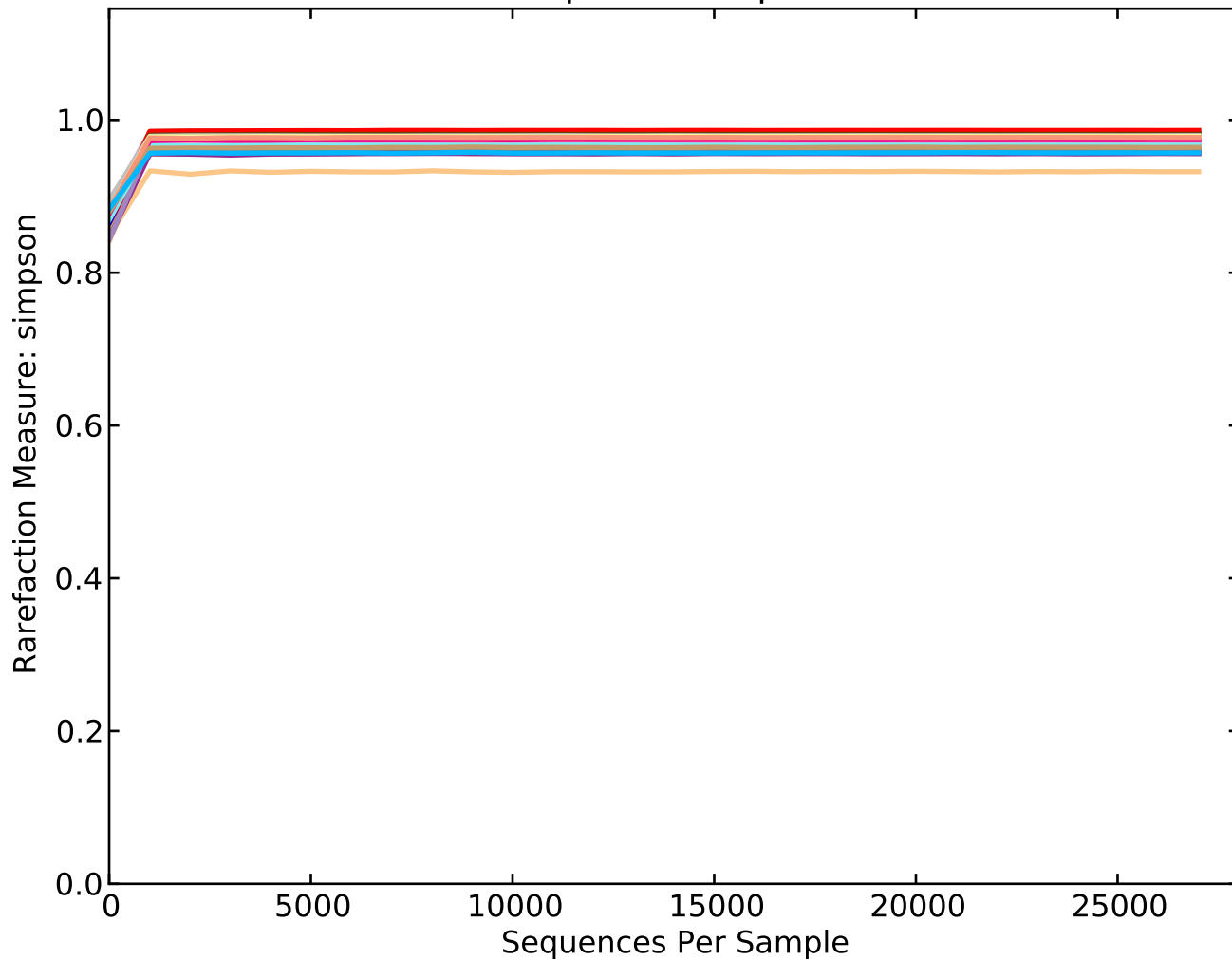

Supplement: Supplementary file 4 — Supplementary Data 1 [file 42003_2023_5520_MOESM4_ESM.zip › 4.Alpha_Diversity/alpha_rarefaction_plot/rarefaction_plots_pdf_depth27686/average_plots/simpsonSampleID.pdf]

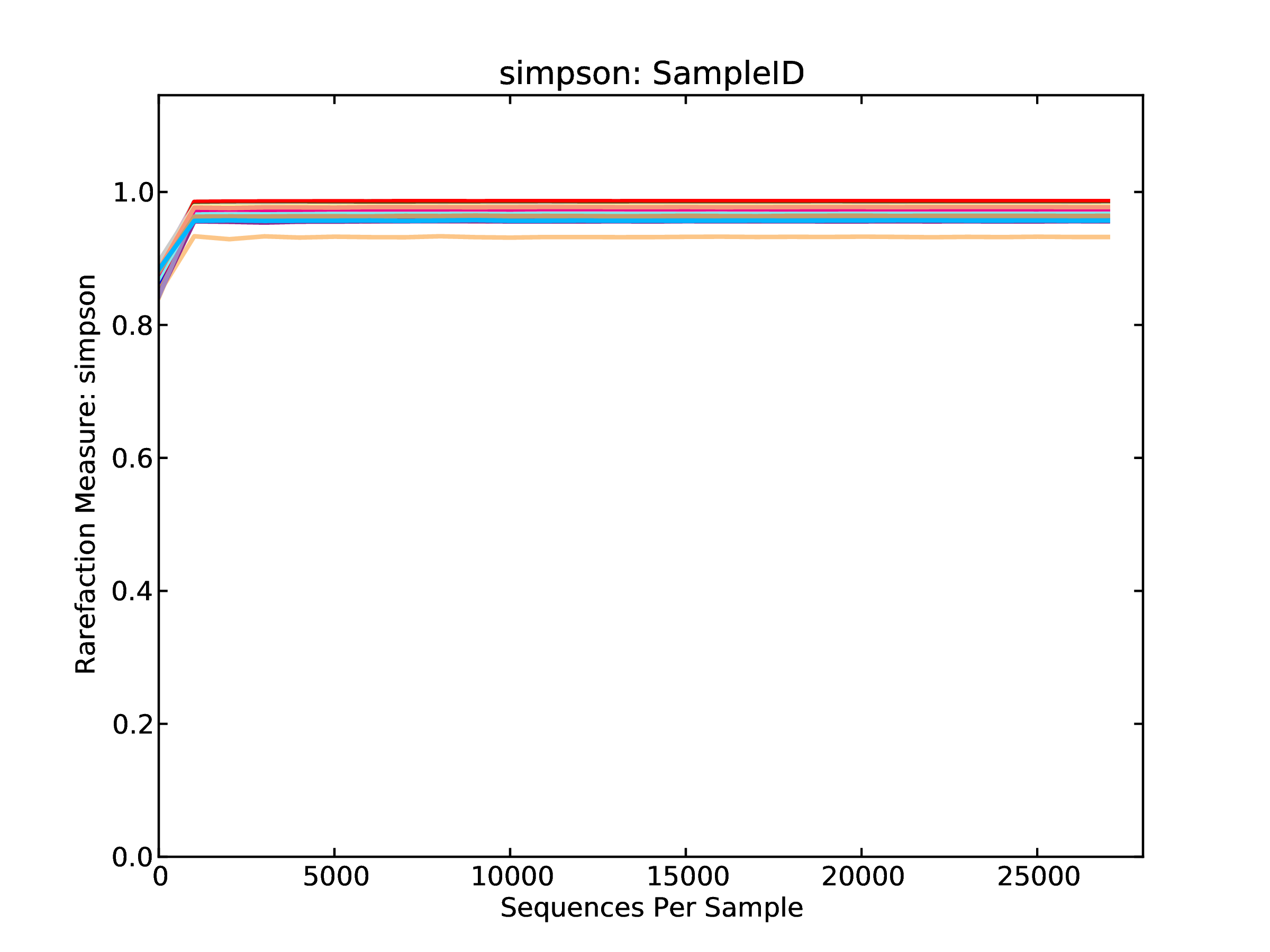

Supplement: Supplementary file 4 — Supplementary Data 1 [file 42003_2023_5520_MOESM4_ESM.zip › 4.Alpha_Diversity/alpha_rarefaction_plot/rarefaction_plots_pdf_depth27686/average_plots/simpsonSampleID.png]

# shannon

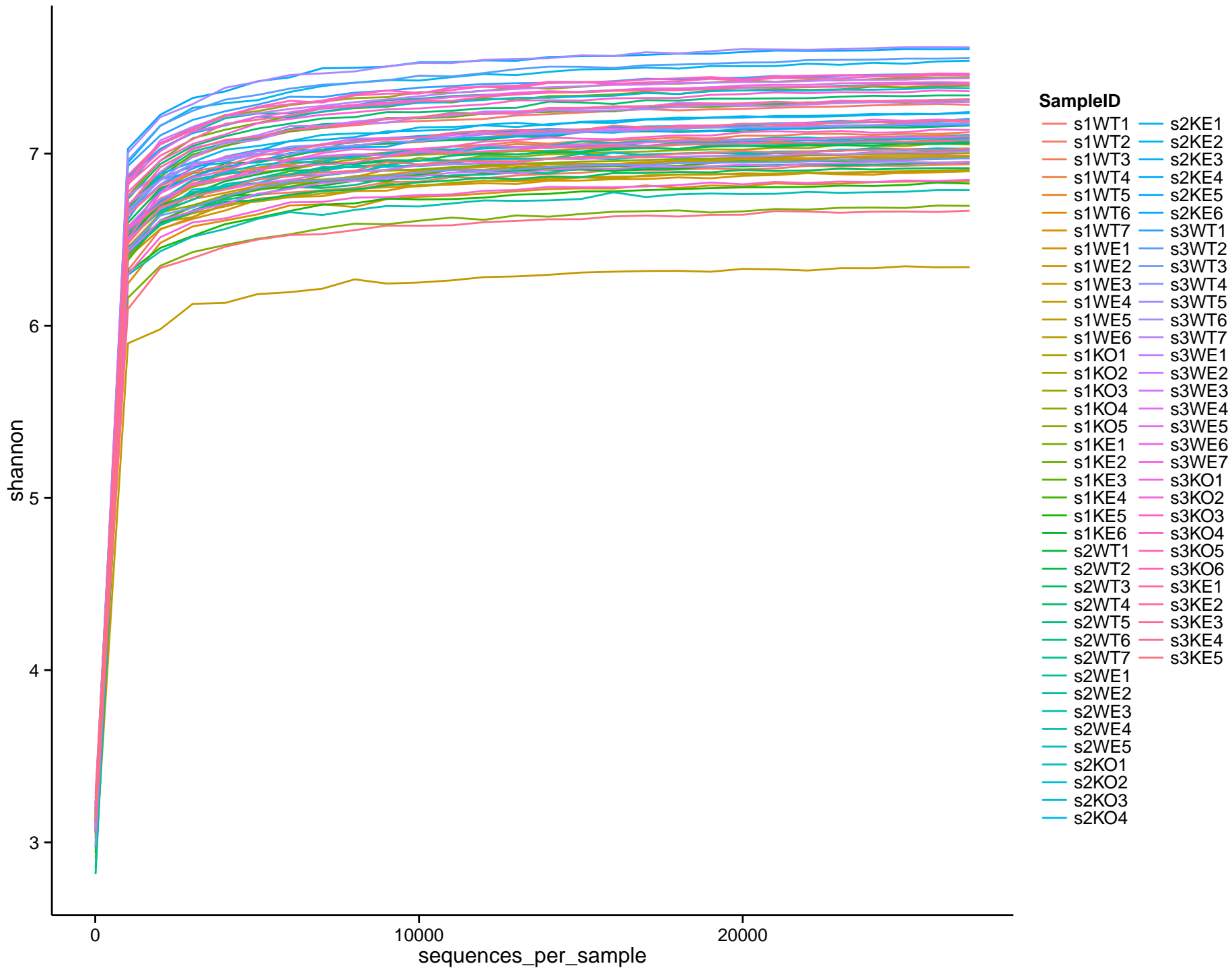

Supplement: Supplementary file 4 — Supplementary Data 1 [file 42003_2023_5520_MOESM4_ESM.zip › 4.Alpha_Diversity/alpha_rarefaction_plot/shannon.pdf]

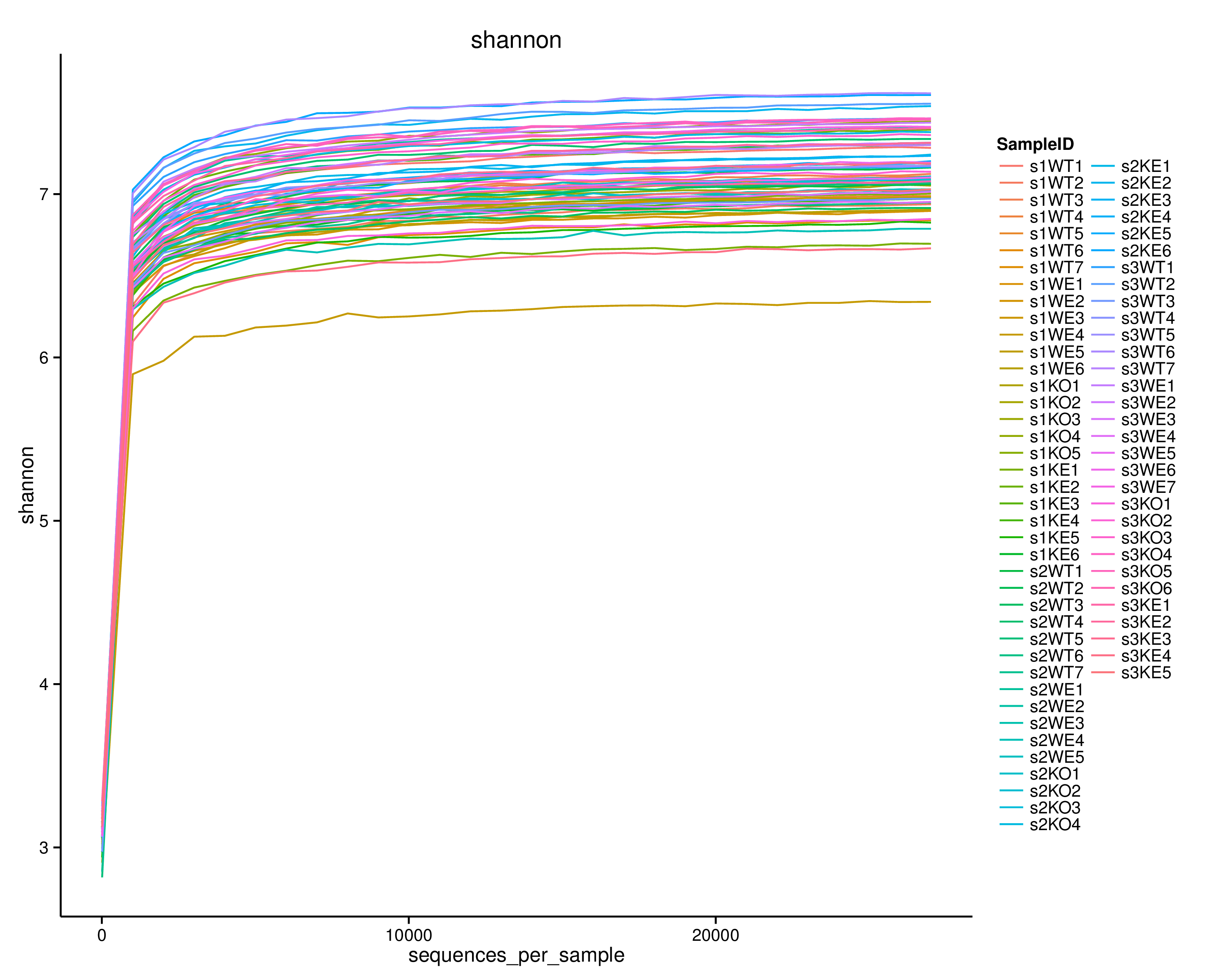

Supplement: Supplementary file 4 — Supplementary Data 1 [file 42003_2023_5520_MOESM4_ESM.zip › 4.Alpha_Diversity/alpha_rarefaction_plot/shannon.png]

simpson

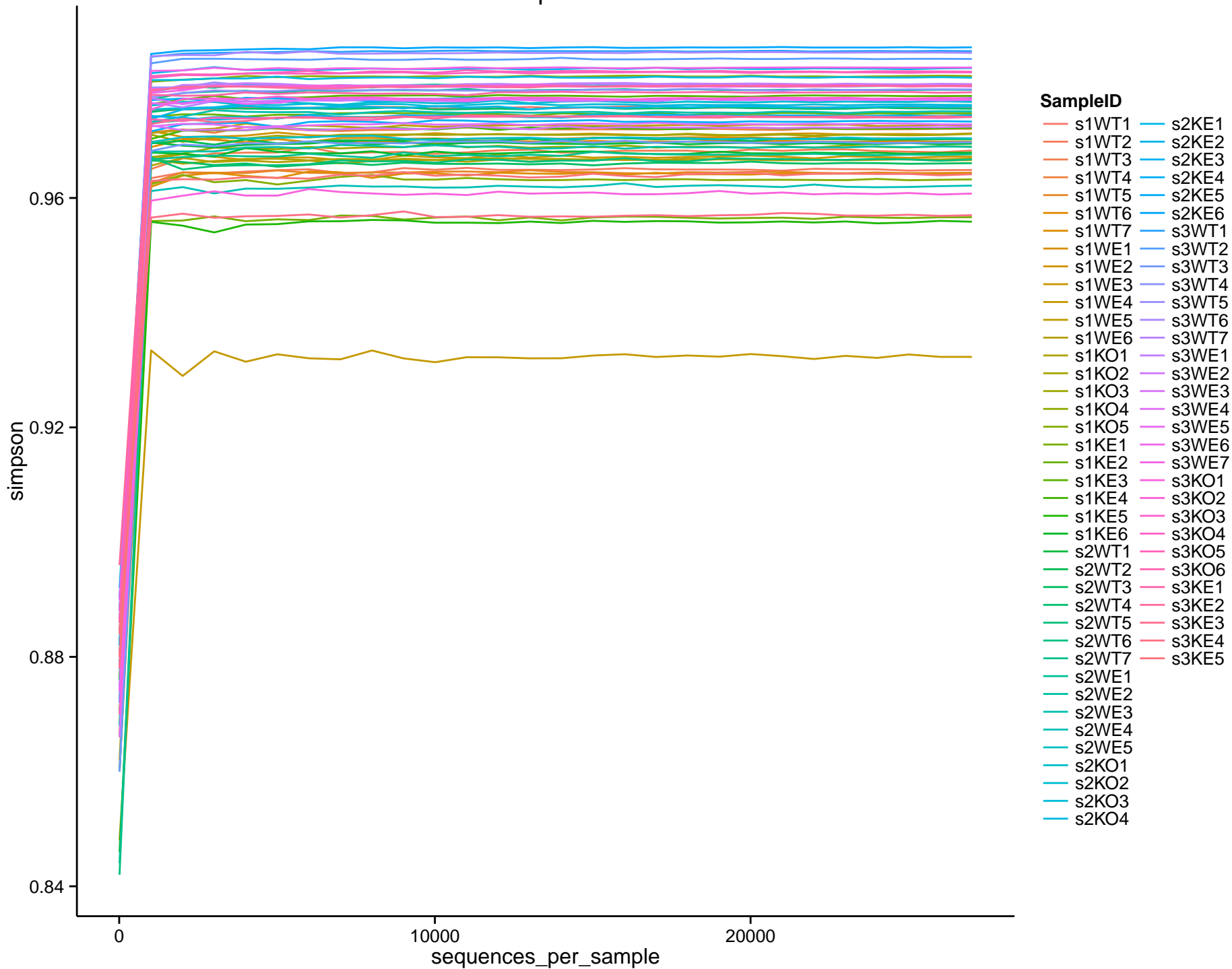

Supplement: Supplementary file 4 — Supplementary Data 1 [file 42003_2023_5520_MOESM4_ESM.zip › 4.Alpha_Diversity/alpha_rarefaction_plot/simpson.pdf]

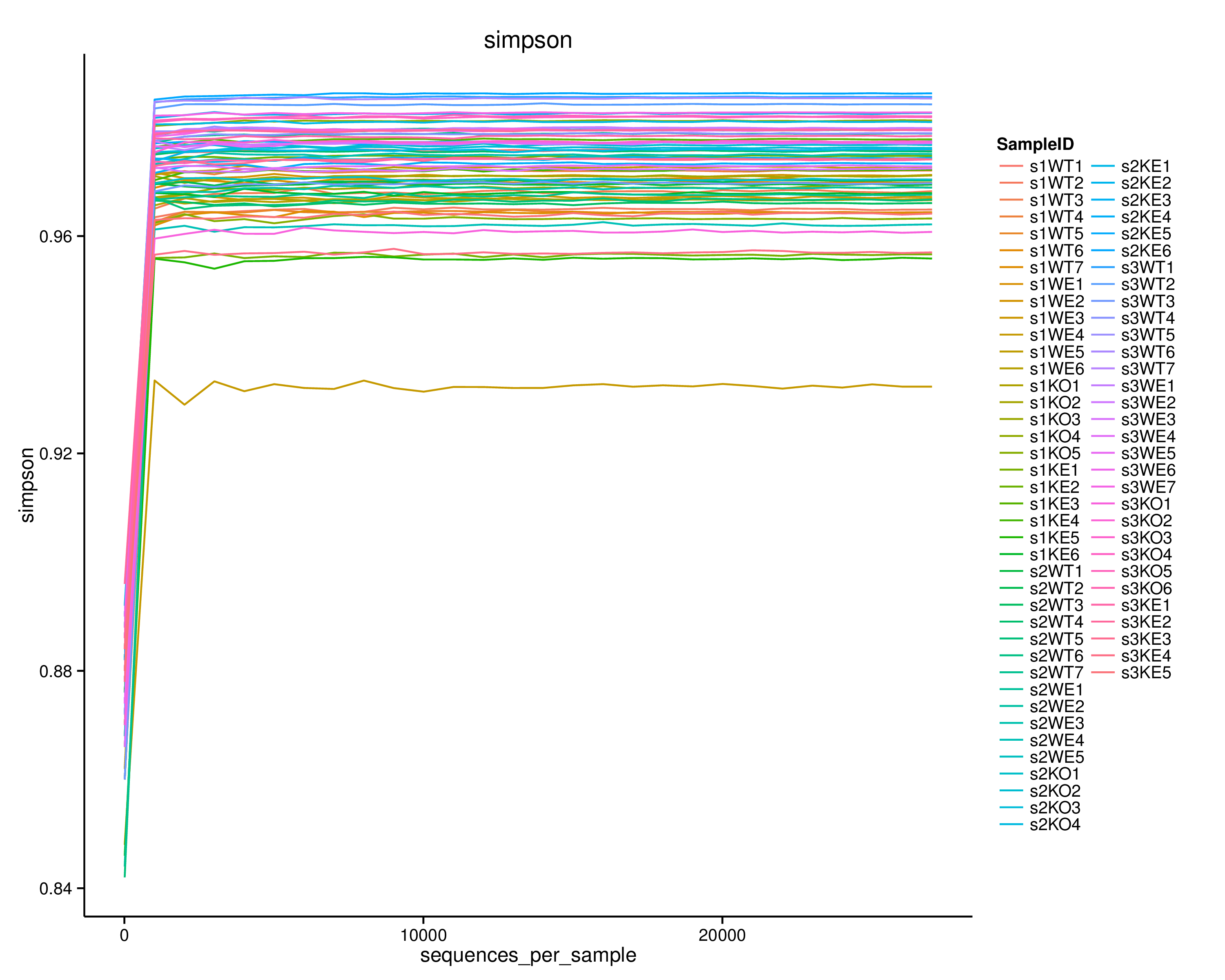

Supplement: Supplementary file 4 — Supplementary Data 1 [file 42003_2023_5520_MOESM4_ESM.zip › 4.Alpha_Diversity/alpha_rarefaction_plot/simpson.png]

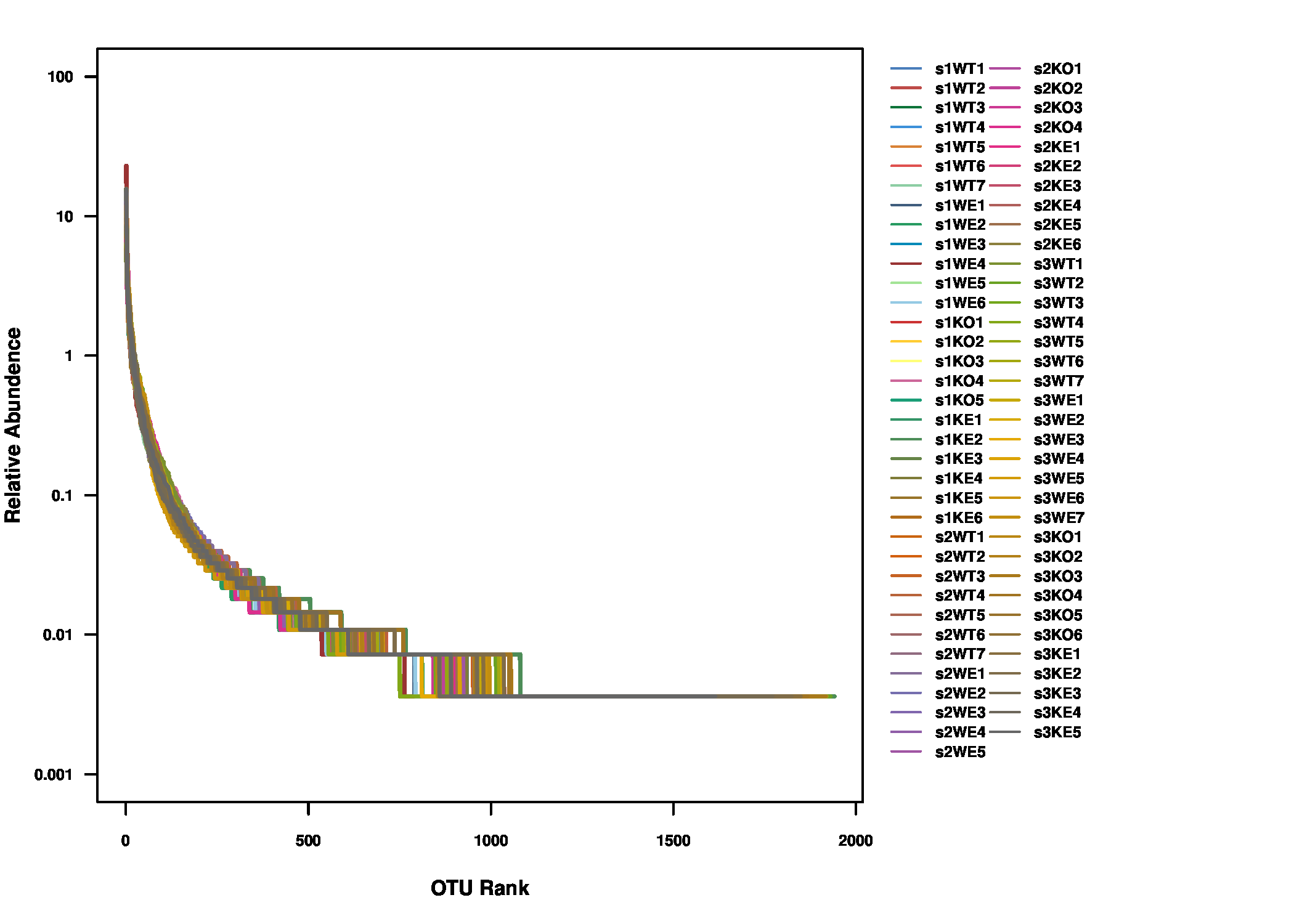

Supplement: Supplementary file 4 — Supplementary Data 1 [file 42003_2023_5520_MOESM4_ESM.zip › 4.Alpha_Diversity/rank_abundance/RankAbundance.png]

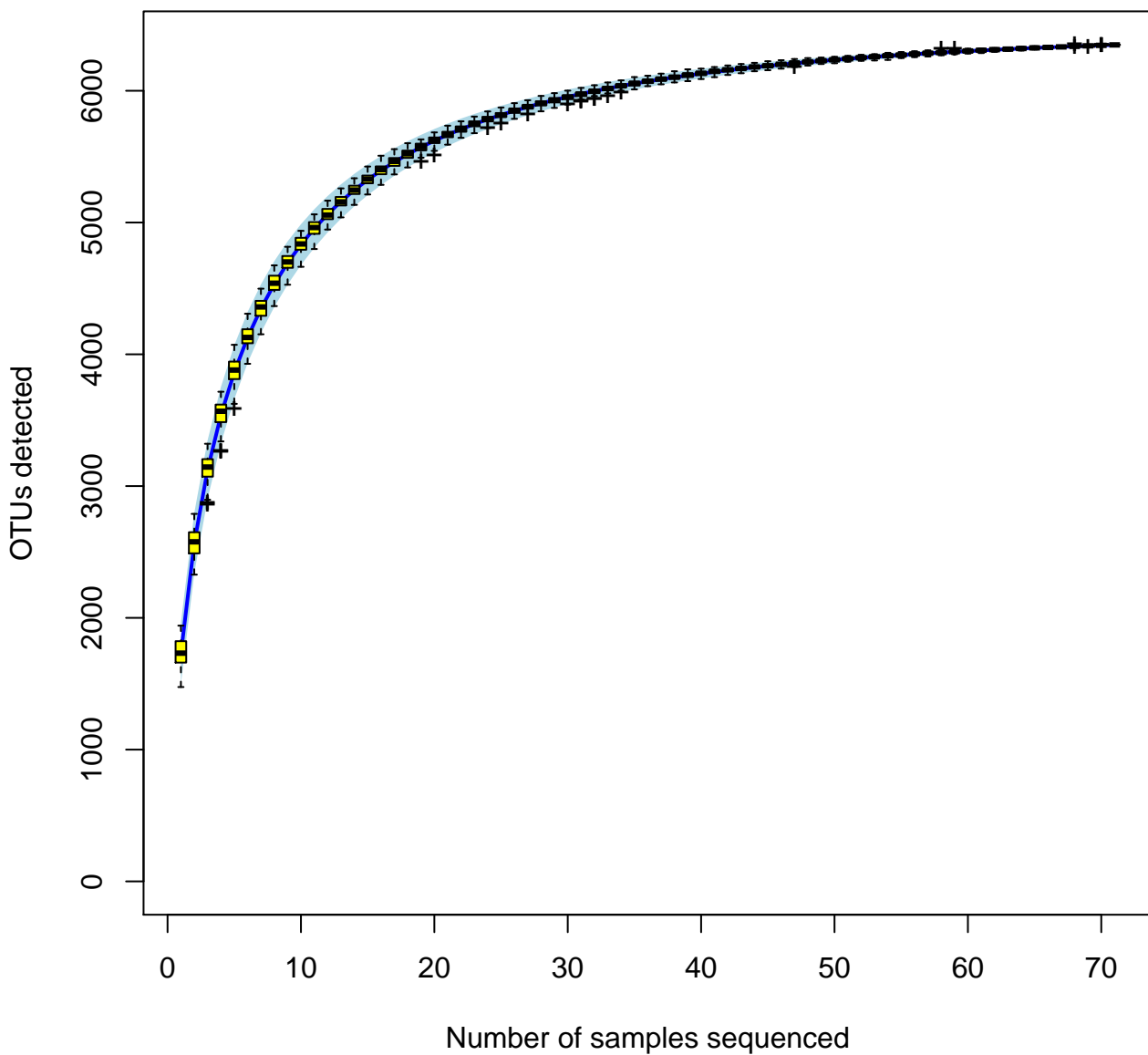

Supplement: Supplementary file 4 — Supplementary Data 1 [file 42003_2023_5520_MOESM4_ESM.zip › 4.Alpha_Diversity/specaccum/specaccum_All.pdf]

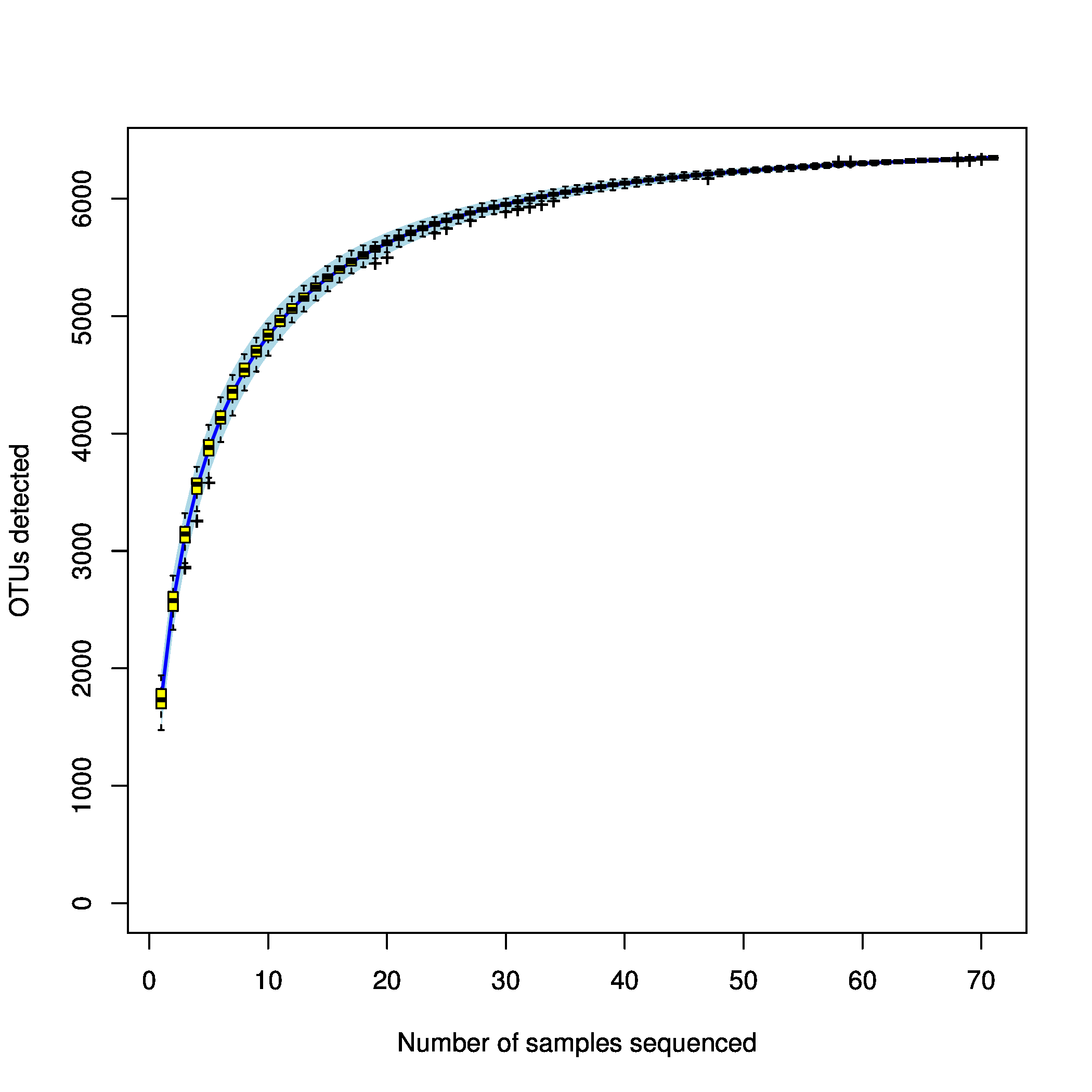

Supplement: Supplementary file 4 — Supplementary Data 1 [file 42003_2023_5520_MOESM4_ESM.zip › 4.Alpha_Diversity/specaccum/specaccum_All.png]

binary jaccard distance

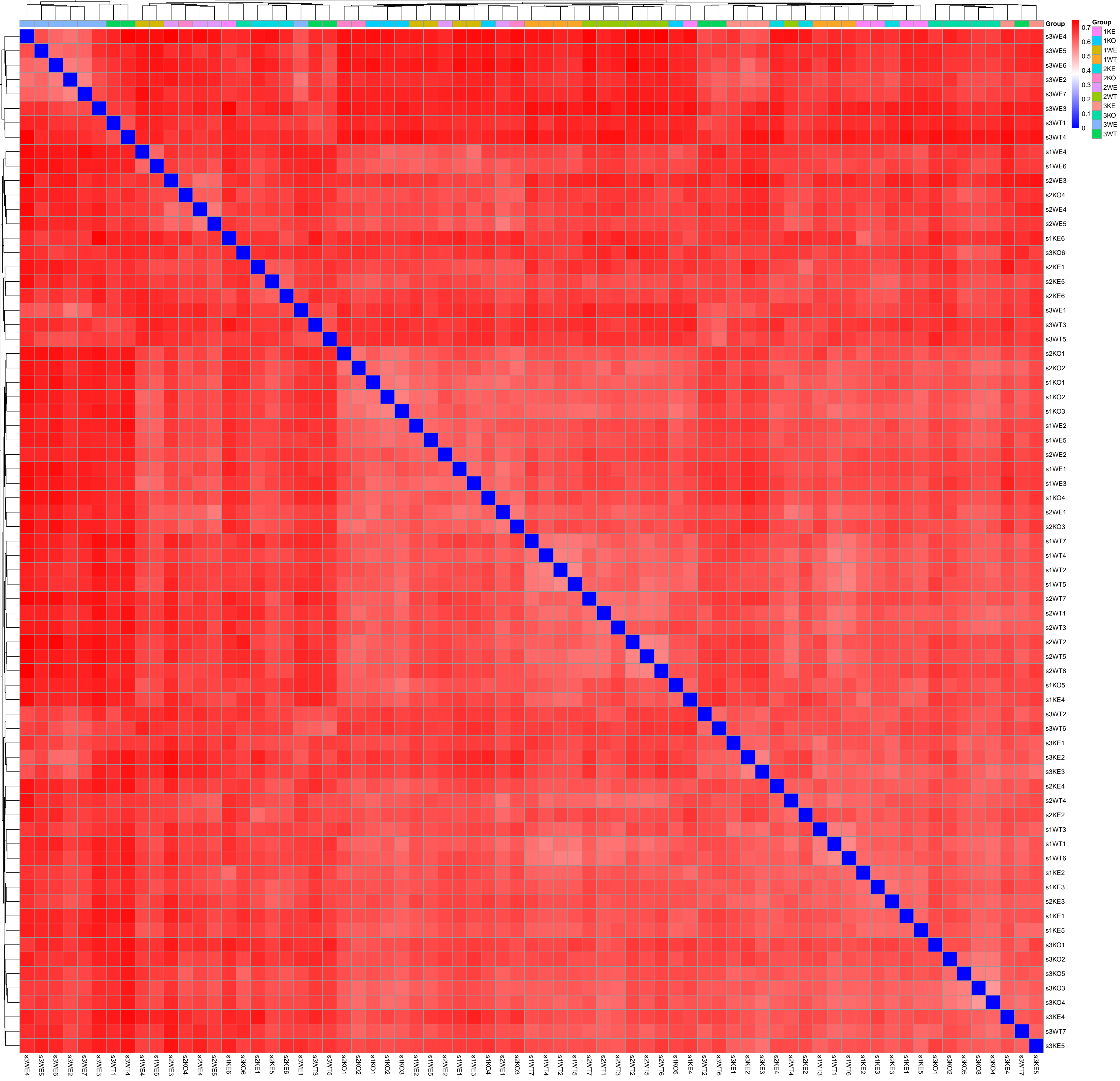

Supplement: Supplementary file 4 — Supplementary Data 1 [file 42003_2023_5520_MOESM4_ESM.zip › 5.Beta_Diversity/Distance/binary_jaccard_distance.pdf]

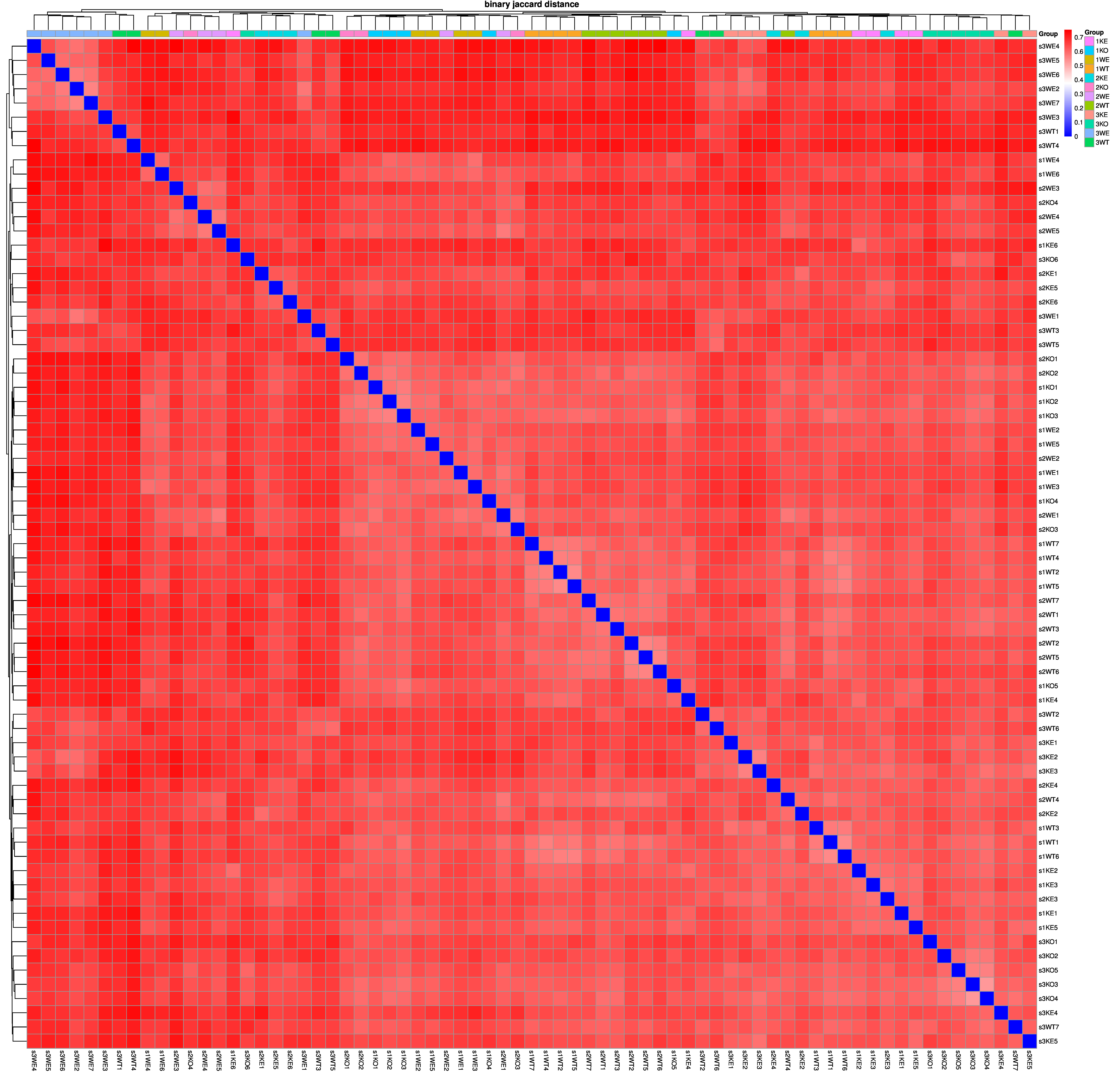

Supplement: Supplementary file 4 — Supplementary Data 1 [file 42003_2023_5520_MOESM4_ESM.zip › 5.Beta_Diversity/Distance/binary_jaccard_distance.png]
