# Supplementary material for: ASMT determines gut microbiota and increases neurobehavioral adaptability to exercise in female mice
Source: Commun Biol. 2023 Nov 7;6:1126. doi: 10.1038/s42003-023-05520-8 (PMC10630421; doi:10.1038/s42003-023-05520-8)
Supplement: Supplementary file 4 — Supplementary Data 1 [file 42003_2023_5520_MOESM4_ESM.zip › 5.Beta_Diversity/NMDS/nmds_binary_jaccard_nolabel_2d.pdf]

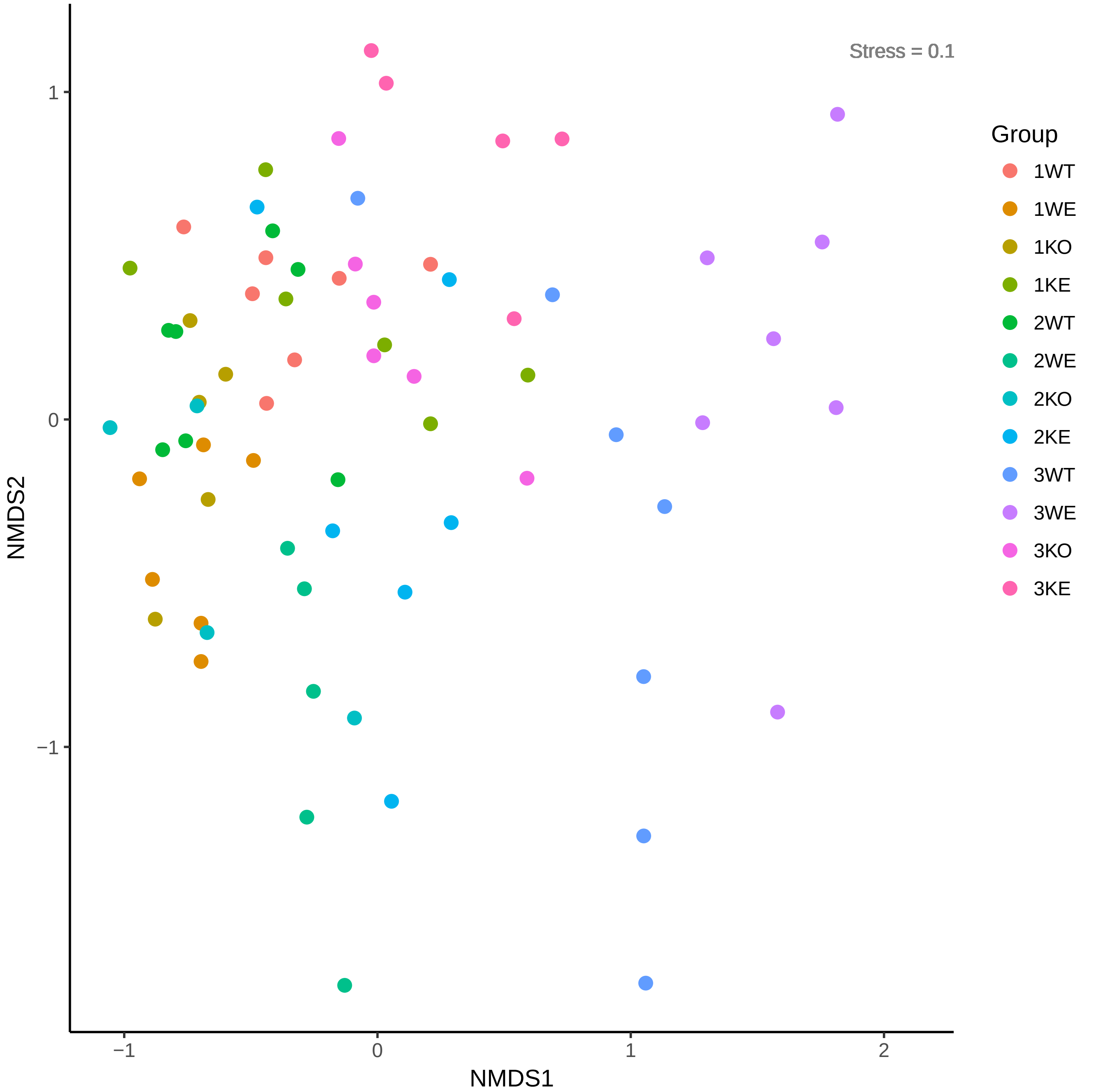

Stress = 0.1
NMDS2
NMDS1
1
0
−1
−1
0
1
2
Group
1WT
1WE
1KO
1KE
2WT
2WE
2KO
2KE
3WT
3WE
3KO
3KE
