# Supplementary material for: ASMT determines gut microbiota and increases neurobehavioral adaptability to exercise in female mice
Source: Commun Biol. 2023 Nov 7;6:1126. doi: 10.1038/s42003-023-05520-8 (PMC10630421; doi:10.1038/s42003-023-05520-8)
Supplement: Supplementary file 4 — Supplementary Data 1 [file 42003_2023_5520_MOESM4_ESM.zip › 5.Beta_Diversity/NMDS/nmds_unweighted_unifrac_nolabel_3d.pdf]

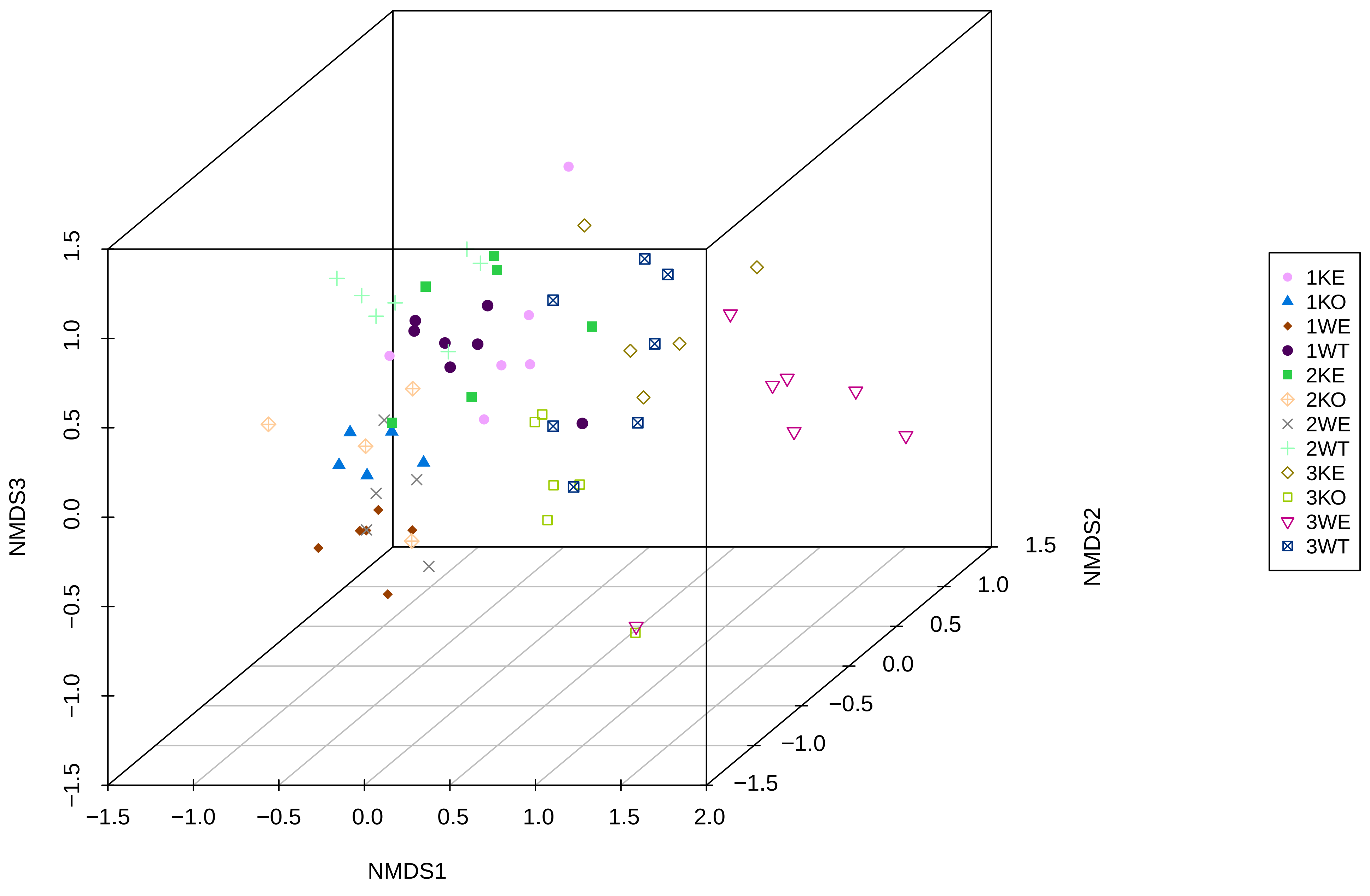

NMDS1
NMDS2
NMDS3
−1.5
−1.0
−0.5
0.0
0.5
1.0
1.5
2.0
1KE
1KO
1WE
1WT
2KE
2KO
2WE
2WT
3KE
3KO
3WE
3WT
