# Supplementary material for: ASMT determines gut microbiota and increases neurobehavioral adaptability to exercise in female mice
Source: Commun Biol. 2023 Nov 7;6:1126. doi: 10.1038/s42003-023-05520-8 (PMC10630421; doi:10.1038/s42003-023-05520-8)
Supplement: Supplementary file 4 — Supplementary Data 1 [file 42003_2023_5520_MOESM4_ESM.zip › 5.Beta_Diversity/PCA/PCA_without_3D_Plot.pdf]

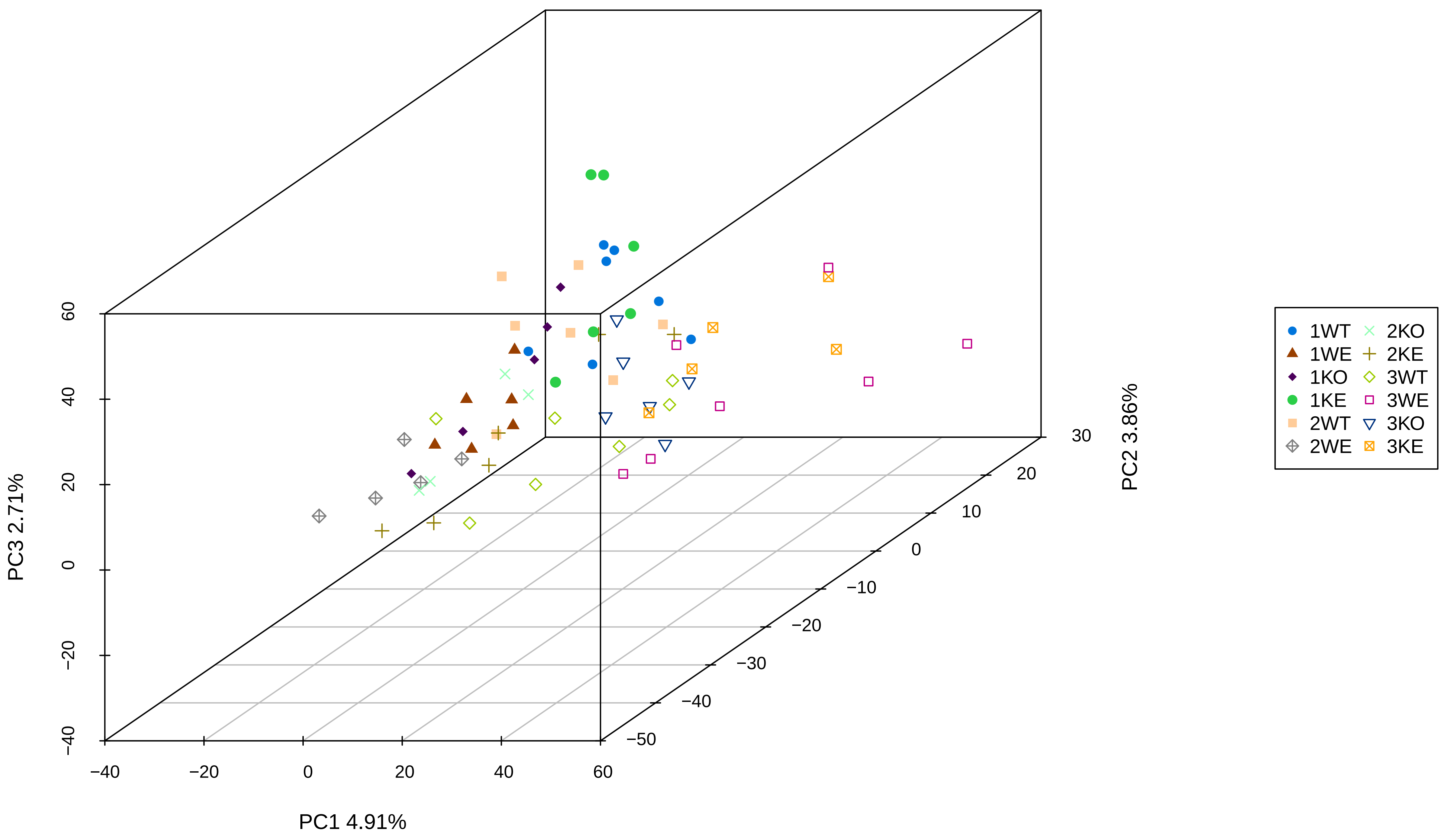
PC1 4.91%
PC2 3.86%
PC3 2.71%
−40
−20
0
20
40
60
−50
−40
−30
−20
−10
0
10
20
30
1WT
1WE
1KO
1KE
2WT
2WE
2KO
2KE
3WT
3WE
3KO
3KE
