# Supplementary material for: ASMT determines gut microbiota and increases neurobehavioral adaptability to exercise in female mice
Source: Commun Biol. 2023 Nov 7;6:1126. doi: 10.1038/s42003-023-05520-8 (PMC10630421; doi:10.1038/s42003-023-05520-8)
Supplement: Supplementary file 4 — Supplementary Data 1 [file 42003_2023_5520_MOESM4_ESM.zip › 5.Beta_Diversity/PCoA/pcoa_binary_jaccard_nolabel_3d.pdf]

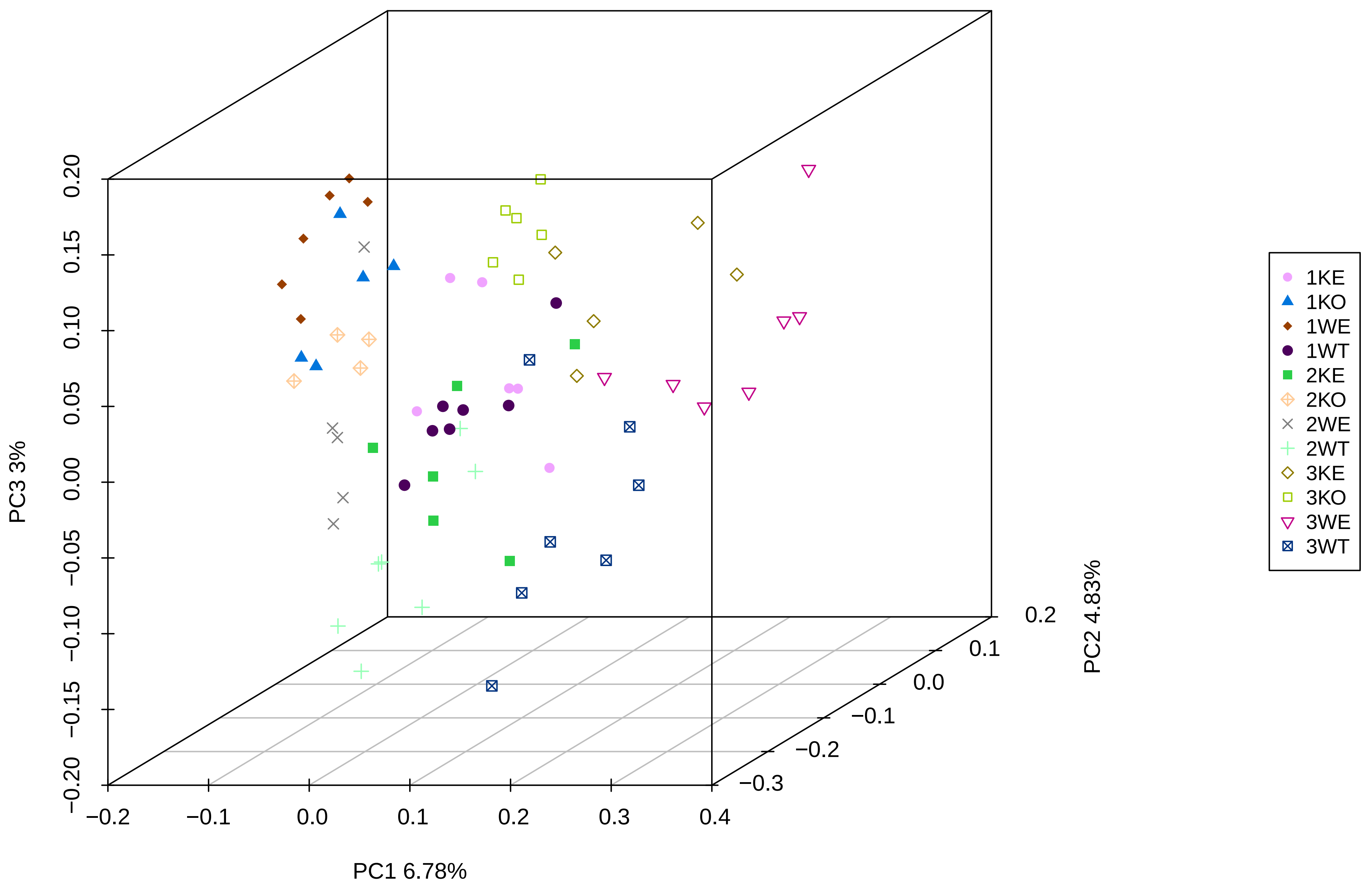

PC3 3%
0.20
0.15
0.10
0.05
0.00
−0.05
−0.10
−0.15
−0.20
−0.2
−0.1
0.0
0.1
0.2
0.3
0.4
PC1 6.78%
PC2 4.83%
0.2
0.1
0.0
−0.1
−0.2
−0.3
1KE
1KO
1WE
1WT
2KE
2KO
2WE
2WT
3KE
3KO
3WE
3WT
